# Proteomic Analysis of Host Cell Protein Dynamics in the Culture Supernatants of Antibody-Producing CHO Cells

**DOI:** 10.1038/srep44246

**Published:** 2017-03-10

**Authors:** Jin Hyoung Park, Jong Hwa Jin, Myung Sin Lim, Hyun Joo An, Jong Won Kim, Gyun Min Lee

**Affiliations:** 1 Department of Biological Sciences, KAIST, 291 Daehak-ro, Yuseong-gu, Daejeon 34141, Republic of Korea; 2 New Drug Development Center, 123 Osongsaengmyeng-ro, Cheongju-si, Chungbuk 28160, Republic of Korea; 3 Graduate School of Analytical Science & Technology, Chungnam National University, 99 Daehak-ro, Yuseong-gu, Daejon 34134, Republic of Korea

## Abstract

Chinese hamster ovary (CHO) cells are the most common cell line used for the production of therapeutic proteins including monoclonal antibodies (mAbs). Host cell proteins (HCPs), secreted and released from lysed cells, accumulate extracellularly during the cultures of recombinant CHO (rCHO) cells, potentially impairing product quality. In an effort to maintain good mAb quality during the cultures, HCPs accumulated extracellularly in batch and fed-batch cultures of a mAb-producing rCHO cell line were identified and quantified by nanoflow liquid chromatography-tandem mass spectrometry, followed by their gene ontology and functional analysis. Due to higher cell concentration and longer culture duration, more HCPs were identified and quantitated in fed-batch culture (2145 proteins identified and 1673 proteins quantified) than in batch culture (1934 proteins identified and 1486 proteins quantified). Clustering analysis of HCPs showed that the concentration profiles of HCPs affecting mAb quality (Lgmn, Ctsd, Gbl1, and B4galt1) correlated with changes in mAb quality attributes such as aggregation, charge variants, and *N*-glycosylation during the cultures. Taken together, the dataset of HCPs obtained in this study provides insights into determining the appropriate target proteins to be removed during both the cultures and purification steps for ensuring good mAb quality.

Chinese hamster ovary (CHO) cells are currently the primary choice for the industrial production of monoclonal antibodies (mAbs) because of their long history of use in commercial production of therapeutic proteins^1^. CHO cells are well adapted to growing in suspension with different media compositions^2^. For large-scale commercial production of mAbs, fed-batch culture has been widely used at large scales, up to 20,000 L working volume, because of its operational simplicity and high-titers. High titers of 3–5 g/L are now routinely achieved in fed-batch cultures^3^,^4^. However, one of the main challenges in fed-batch process development is to maintain high productivity while also ensuring good quality of mAb.

In fed-batch culture, periodic feeding of concentrated nutrients to avoid depletion of key media components prolongs culture longevity and productivity. Concomitantly, host cell proteins (HCPs) that are released from dead cells and secreted from viable cells accumulate extracellularly at a much higher level than they do in batch culture, thereby impairing product quality^5^,^6^. In particular, proteases^7^,^8^,^9^ and glycosidases^10^,^11^ that accumulate in culture medium negatively affect the quality of mAbs in recombinant CHO (rCHO) cell cultures. In addition, although the concentrations of HCPs in cell culture harvests are reduced to acceptable levels after a series of purification steps, certain HCPs escape an entire purification process and remain in the final mAb drug substance at levels that affect product quality and stability^12^,^13^. Therefore, it is paramount to characterize and quantify HCPs in fed-batch cultures to ensure an optimal mAb quality and perform targeted removal of HCPs during the purification steps.

Recently, secreted proteins from two particular CHO host cell lines (CHO DG44 and CHO-S) were identified and quantified using nanoflow liquid chromatography-tandem mass spectrometry (LC-MS/MS)^14^ and others from another CHO host cell line (CHO-K1) were characterized using LC-MS/MS and multiple bioinformatics tools^2^. In addition, HCPs in cell culture harvests from fed-batch cultures of a mAb-producing CHO GS cell line, after being partially purified by SDS-PAGE, were subjected to proteomic analysis to elucidate the correlation between secreted proteins and mAb productivity^15^. However, identification and quantification of HCPs, especially those affecting the mAb quality, in culture supernatants during fed-batch cultures of mAb-producing CHO cell lines, has not yet been performed.

In this study, in an effort to maintain good mAb quality in fed-batch cultures, HCPs accumulated extracellularly at different growth phases of a mAb-producing rCHO cell line were identified and quantified using LC-MS/MS. Prior to LC-MS/MS analysis, mAbs, predominantly present in the culture supernatants, were removed by protein A affinity chromatography instead of SDS-PAGE to minimize the loss of HCPs. Furthermore, the quality attributes of the mAbs (aggregation, charge variation, and *N*-glycosylation) were analyzed to understand the effects of HCPs present in the culture supernatants on their quality. A more complete analysis of HCPs in the culture supernatants will provide valuable information toward establishing effective methods allowing the production of high quality mAbs in fed-batch cultures and the removal of HCPs affecting their stability and quality throughout the purification process.

## Results

**Figure 1:**
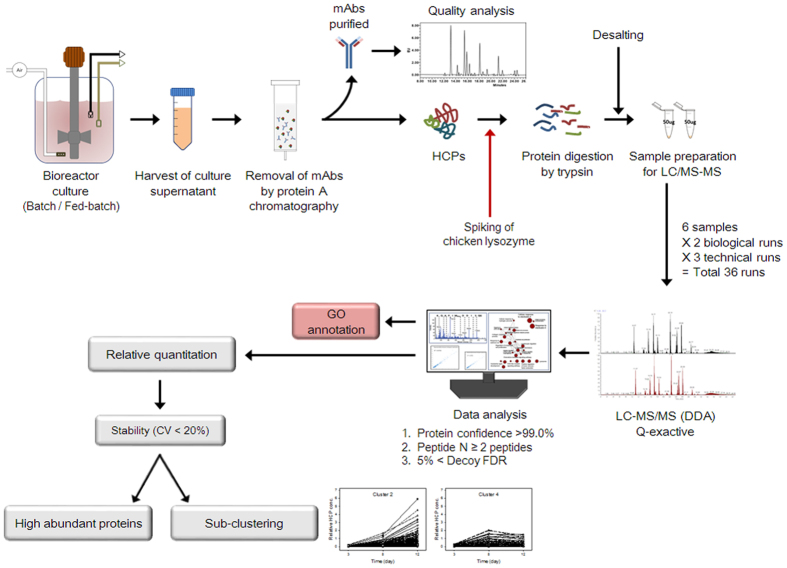
Workflow used to characterize the quality attributes of mAbs and identify HCPs in the culture supernatants.

In an aim to maintain good mAb quality in fed-batch cultures, such cultures of mAb-producing cells were performed in a bioreactor with pH and DO control. Batch cultures were also performed as a control. Culture supernatants were sampled at different growth phases on days 3, 5, and 8 in batch cultures and on days 3, 8, and 12 in fed-batch cultures. Cultures were performed three independent times. The workflow used to characterize the quality attributes of mAbs and identify HCPs in the culture supernatants is outlined in Fig. 1. Briefly, the mAbs present in the culture supernatants were removed by protein A affinity chromatography. Subsequently, the removed mAbs were subjected to mAb quality analysis in regard to aggregation, charge variation, and *N*-glycosylation. Since protein-free medium and protein-free nutrient cocktails were used in the cultures, the remaining proteins in the culture supernatants after this removal step are HCPs released from dead cells and secreted from viable cells. The HCPs were subjected to tryptic digestion and desalting, and were identified by LC-MS/MS. Subsequently, Gene ontology (GO) annotation was performed to categorize the proteins. Next, MS-based label-free quantitation was performed to compare the abundances of the HCPs at different growth phases. Highly abundant proteins were listed and clustering, according to their concentration patterns during the cultures, was also performed.

### Cell growth, mAb production, and HCPs in culture supernatants

**Figure 2:**
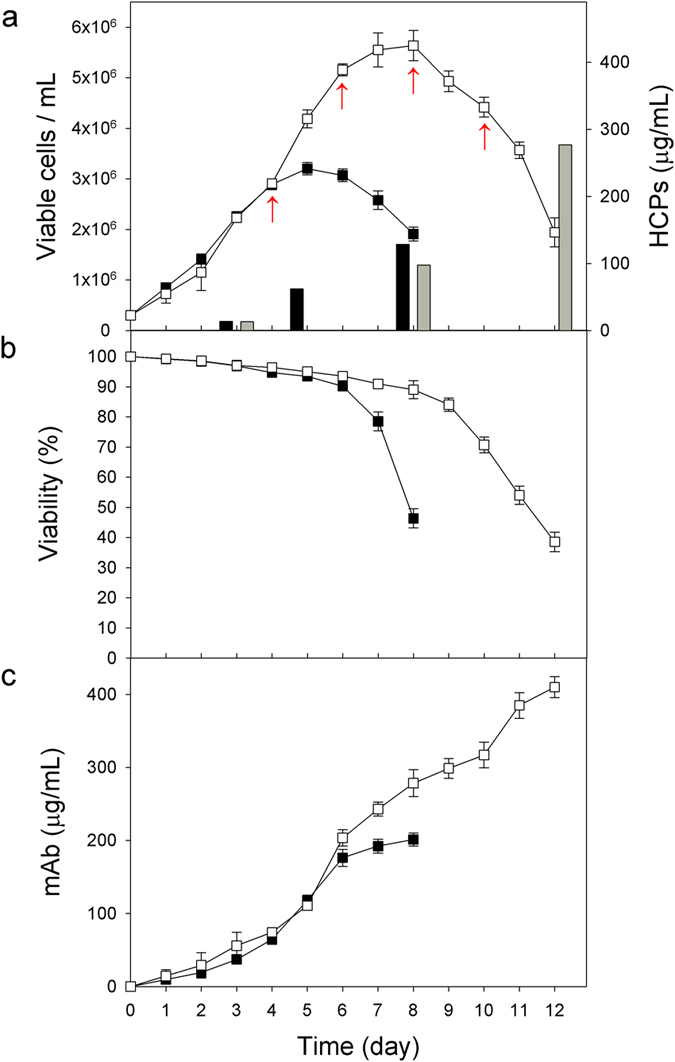
Profiles of (**a**) cell growth and HCPs concentration, (**b**) viability, and (**c**) mAb concentration during batch and fed-batch cultures. Batch culture (closed square and black bar) and fed-batch culture (open square and gray bar). Arrows correspond to feeding events. The error bars represent the SDs calculated from three independent experiments.

Figure 2 shows the profiles of cell growth, cell viability, mAb production, and HCP concentration in culture supernatants during batch and fed-batch cultures. In batch cultures, a maximum viable cell concentration (MVCC) of (3.2 ± 0.1) × 10^6^ cells/mL was achieved on day 5 (Fig. 2a). In fed-batch cultures, viable cell concentration, viability, and mAb concentrations were estimated and plotted daily before feeding nutrient cocktails, to avoid obtaining excessively complex profiles. The addition of nutrient cocktails further increased the MVCC and extended culture longevity (Fig. 2a,b). An MVCC of (5.6 ± 0.3) × 10^6^ cells/mL was achieved on day 8 in fed-batch cultures. Due to the increased MVCC and extended culture longevity, the maximum antibody concentration in fed-batch culture (410.1 ± 14.5 μg/mL) was significantly higher than that obtained in batch culture (210.3 ± 8.8 μg/mL) (*P* < 0.05, Fig. 2c).

To determine the HCP concentrations in the culture supernatants, the mAbs, which are the mainly represented proteins, were first removed using protein A affinity chromatography. In batch cultures, HCP concentrations significantly increased in the declining phase of growth, suggesting that a significant amount of HCPs was released from dead cells (*P* < 0.05) (Fig. 2a,b). The HCP concentrations on days 3 and 8 were 13.8 ± 0.8 and 128.4 ± 10.5 μg/mL, respectively. Likewise, the HCP concentrations significantly increased in the decline phase of growth in fed-batch cultures. However, due to the high MVCC, the HCP concentrations at the end of the fed-batch cultures (277.1 ± 7.3 μg/mL on day 12) were approximately 2.2 times higher than those observed in batch cultures (Fig. 2a).

### Analysis of mAb quality attributes with regard to aggregation, charge variation, and *N*-glycosylation

To analyze mAb quality, the mAbs present in the culture supernatants sampled at different growth phases in batch and fed-batch cultures (as shown in Fig. 2) were purified by protein A affinity chromatography. Subsequently, their aggregation, charge variants, and *N*-glycosylation levels were assessed by size exclusion-high performance liquid chromatography (SEC-HPLC), weak cation exchange-HPLC (WCX-HPLC), and ultra-performance liquid chromatography (UPLC), respectively. Prior to the analysis of *N*-glycosylation, the *N*-linked glycans from the mAbs were released using PNGase F enzyme.

**Figure 3:**
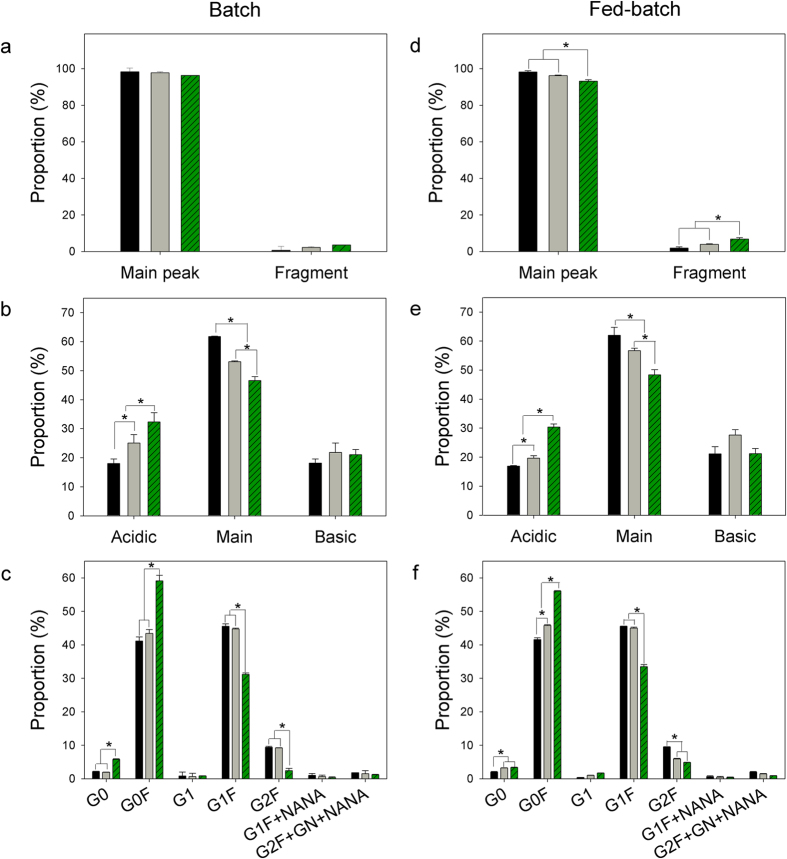
Profiles of aggregation (**a**,**d**), charge variation (**b**,**e**), and *N*-linked glycosylation analysis (**c**,**f**) of mAbs during batch (**a**–**c**) and fed-batch cultures (**d**–**f**). Day 3 (◾), day 5 (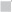), and day 8 (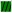) in batch culture. Day 3 (◾), day 8 (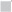), and day 12 (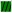) in fed-batch culture. G0, agalactosylated glycan without fucose; G0F, agalactosylated glycan with fucose; G1, monogalactosylated glycan without fucose; G1F, monogalactosylated glycan with fucose; G2F, digalactosylated glycan with fucose; G1F + NANA, monogalactosylated glycan with *N*-acetylneuramic acid; G2F + GN + NANA, digalactosylated glycan with *N*-acetylglucosamine and *N*-acetylneuramic acid.

Figure 3 shows the profiles of aggregation, charge variants, and *N*-linked glycan proportion of mAbs during the cultures. The SEC-HPLC analysis shows one main peak corresponding to the mAb monomers and one minor peak corresponding to smaller fragments, indicating that there were no mAb aggregates generated during neither the batch nor the fed-batch cultures (Fig. 3a,d). In fed-batch cultures, the portion of smaller fragments significantly increased from 1.9 ± 0.7% on day 3 to 6.8 ± 0.8% on day 12 (*P* < 0.05), as summarized in Supplementary Table S1. The WCX-HPLC analysis shows that mAbs produced in both batch and fed-batch cultures were separated into an acidic peak, a main peak, and a basic peak (Fig. 3b,e). In both batch and fed-batch cultures, acidic charge variants significantly increased with a concomitant decrease of the main peak with culture time (*P* < 0.05), as summarized in Supplementary Table S2. The portion of basic variants did not change significantly in both cases (*P* > 0.05). In fed-batch cultures, the portion of acidic variants increased from 16.9 ± 0.2% on day 3 to 30.4 ± 1.1% on day 12 while the main peak portion decreased from 60.0 ± 2.7% on day 3 to 48.4 ± 1.8% on day 12.

The UPLC analysis shows that most of the *N*-glycans of mAbs produced in both types of culture had a core fucose and varying terminal galactose contents (G0F, G1F, and G2F) (Fig. 3c,f). In both the batch and fed-batch cultures, G0F, G1F, and G2F represented 94–95% of the total glycan amount on day 3 (see Supplementary Table S3). In both types of culture, G1F and G2F then decreased, whereas G0F increased with culture time. In fed-batch cultures, G1F and G2F significantly decreased from 44.8 ± 0.0% and 9.4 ± 0.1% on day 3 to 33.3 ± 0.6% and 4.9 ± 0.1% on day 12, respectively (*P* < 0.05). In addition, small portions of monogalactosylated glycan with fucose and *N*-acetylneuramic acid (G1F + NANA, 0.4 ± 0.2%) and digalactosylated glycan with NANA (G2F + GN + NANA, 0.9 ± 0.3%) were observed on day 12 (see Supplementary Table S3).

### Identification of HCPs in culture supernatants

To characterize the HCPs in the culture supernatants, these were isolated from the culture supernatants sampled at different growth phases in batch and fed-batch cultures (as shown in Fig. 2), subsequently analyzed by high-resolution LC-MS/MS (Q-exactive), and finally subjected to data analysis using SEQUEST and X! Tandem, as outlined in Fig. 1. A total of 2460 HCPs in culture supernatants were identified at a minimum confidence level >99%, with at least two unique peptides, and a false discovery rate (FDR) <5%.

**Figure 4:**
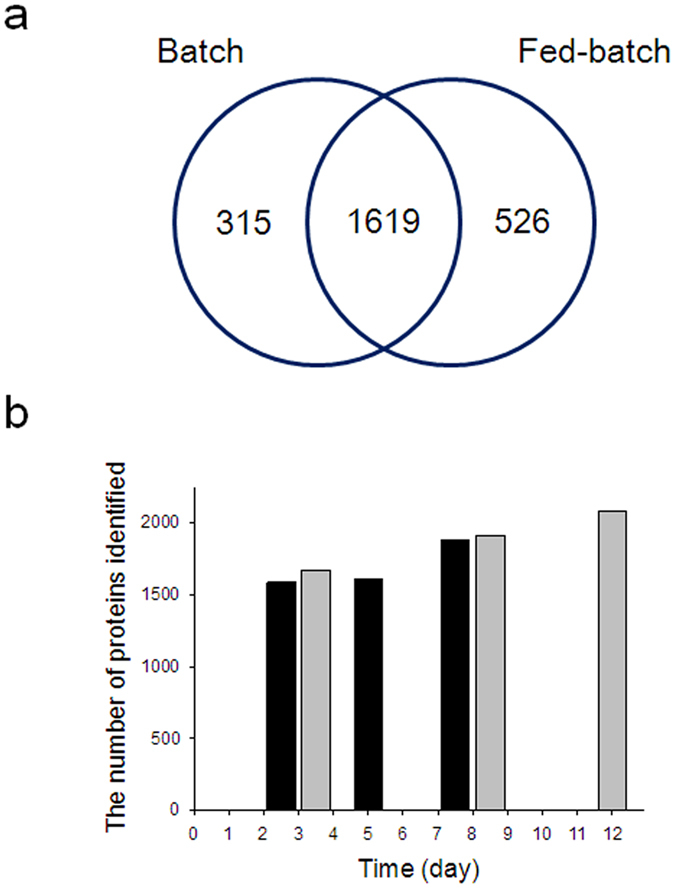
Number of identified HCPs in the culture supernatants during batch and fed-batch cultures. (**a**) Venn diagram representing total number of proteins. (**b**) The number of identified HCPs in the culture supernatants during batch (black) and fed-batch cultures (gray).

Figure 4 shows the number of identified HCPs in culture supernatants during the cultures. From a total of 2460 identified HCPs, 1619 were commonly identified in both batch and fed-batch cultures (Fig. 4a). A total of 1934 and 2145 HCPs were identified in batch and fed-batch cultures, respectively. Additionally, approximately 73–74% of them were identified at all three sampling times. In both types of culture, the number of identified HCPs increased toward the end of the culture process as cell viability decreased (Fig. 4b). In addition, more HCPs were identified in the fed-batch cultures, due to higher cell concentrations compared to those in batch culture (Fig. 2a). In fed-batch culture, 2074 HCPs were identified on day 12, while contrastingly, in batch culture, 1878 HCPs were identified on day 8. A complete list of the 2460 identified HCPs is provided in Supplementary Table S4.

To understand the biological roles of the identified HCPs, GO analysis of identified HCPs was performed using the Uniprot bioinformatics resource. GO terms of 1104 identified HCPs in batch culture and 1171 identified HCPs in fed-batch culture were successfully determined and functionally classified into cellular component (CC), molecular function (MF), and biological process (BP). The 3 sets of GO categorizations are overlapping, i.e., the proteins are placed into one or more categories.

**Figure 5:**
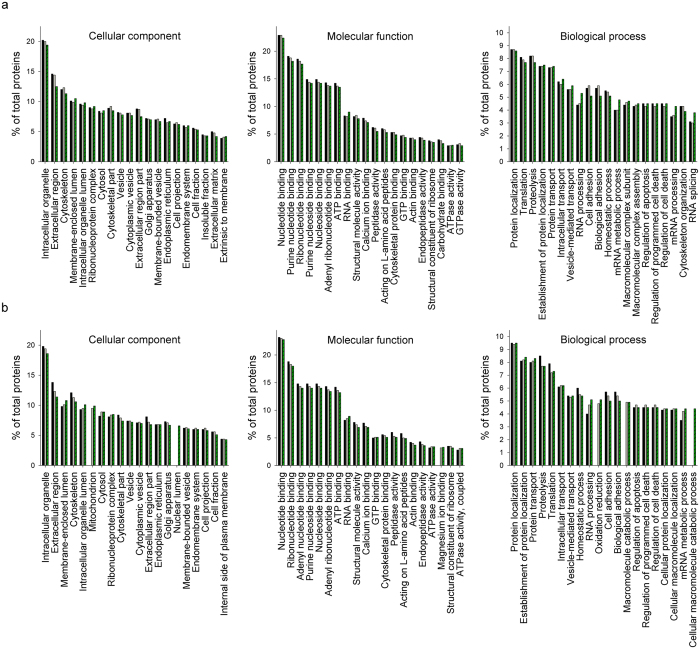
Top 20 enriched GO terms classified into CC, MF, and BP. (**a**) GO terms of the 1104 identified HCPs during batch culture; day 3 (◾), day 5 (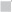), and day 8 (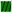). (**b**) GO terms of the 1171 identified HCPs during fed-batch culture; day 3 (◾), day 8 (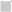), and day 12 (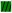).

Figure 5 shows the top 20 enriched GO terms classified into CC, MF, and BP during the cultures. Regarding CC, intracellular organelle was the most common (~20%) in both batch (Fig. 5a) and fed-batch cultures (Fig. 5b). However, unlike in the batch culture, mitochondrion (~10% on day12) and nuclear lumen (~7% on day12) became common at the end of the fed-batch culture (Fig. 5b). For MF, nucleotide binding was the most common (~23%) in both cultures. Regarding BP, protein localization was the most common (~9%) in both cultures. In addition, RNA processing and mRNA metabolic process increased toward the end of both cultures. Complete lists of GO analysis in batch and fed-batch cultures are provided in Supplementary Tables S5 and S6, respectively.

### Quantification of identified HCPs in culture supernatants

A total of 1486 and 1673 proteins, which were identified in all six LC-MS/MS runs (technical triplicate × biological duplicate) at each sampling time in batch and fed-batch cultures, respectively, were subjected to quantitation. The relative values of protein abundance were estimated by spectral counting method, followed by normalized spectral abundance factor (NSAF) method for reliable normalization^20^. Since large proteins usually tend to contribute more to the ‘peptide/spectra’ than small ones, the NSAF method is useful to minimize the variations originated from protein length. NSAF values of chicken lysozyme that was used as an internal control were satisfied with CV <1% in all 36 LC-MS/MS runs (data not shown). Furthermore, all linear correlation values (R^2^) between two biological experiments were greater than 0.96 in both batch and fed-batch cultures (see Supplementary Fig. S1), suggesting that semi-quantification of identified HCPs was highly reproducible.

To quantitate the identified HCPs in the culture supernatants, the normalized spectral count of each HCP was multiplied by the HCPs concentration of the culture supernatants shown in Fig. 2a. Then, the HCPs with CV < 20%, which was calculated from technical triplicates of each sample, were filtered out for reliable quantitation. As a result, a total of 530 and 569 proteins were quantitated in batch and fed-batch cultures, respectively, as summarized in Supplementary Table S7.

**Figure 6:**
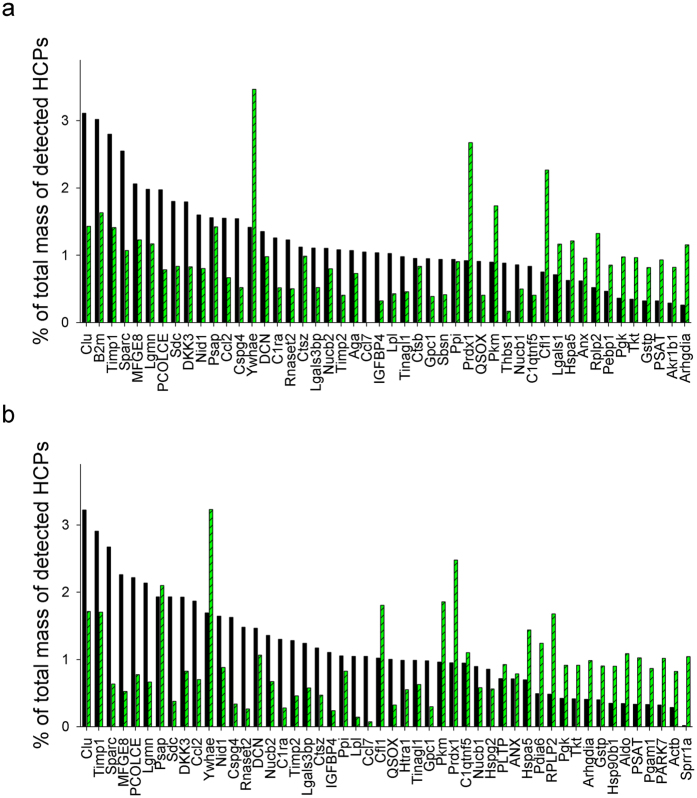
Percentage of total mass of detected HCPs. (**a**) Top 30 most abundant HCPs in culture supernatants sampled on day 3 (◾) and day 8 (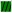) in batch culture. (**b**) Top 30 most abundant HCPs in culture supernatants sampled on day 3 (◾) and day 12 (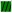) in fed-batch culture. The top 30 most abundant HCPs in the culture supernatants sampled in the exponential growth phase were plotted as % of total mass of detected HCPs in descending order.

The top 30 most abundant HCPs in the culture supernatants sampled in the exponential growth phase (day 3 in both batch and fed-batch cultures) and at the end of the culture (day 8 in batch culture and day 12 in fed-batch culture) were plotted as % of total mass of detected HCPs in Fig. 6. HCPs with higher quantities at each sampling time were very similar between batch and fed-batch cultures. However, in both types of culture, highly represented HCPs in the exponential growth phase were different from those observed at the end of the cultures. For example, high quantities of clusterin (Clu), metalloproteinase inhibitor 1 (Timp1), and sparc (Sparc) were observed in both the exponential and stationary phases of growth, whereas high quantities of 14-3-3 protein (Ywhae), peroxiredoxin 1 (Prdx1), and cofilin 1 (Cfl1) were observed at the end of the cultures (Fig. 6a,b).To understand the biological roles of these top 30 most abundant HCPs, GO analysis was performed using the Uniprot bioinformatics resource.

**Figure 7:**
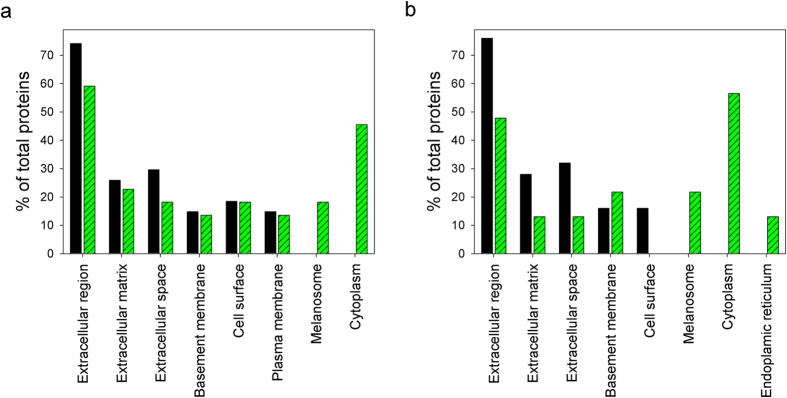
GO analysis of the top 30 most abundant HCPs classified into CC. (**a**) GO terms of the top 30 HCPs during batch culture; day 3 (◾) and day 8 (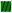). (**b**) GO terms of the top 30 most abundant HCPs during fed-batch culture; day 3 (◾) and day 12 (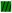).

Figure 7 shows the GO terms of the top 30 most abundant HCPs, which were classified into CC. In both batch and fed-batch cultures, HCPs with the highest percentages in the exponential growth phase were associated with the extracellular region (Fig. 7a,b). At the end of the fed-batch cultures, the percentage of HCPs associated with the extracellular region, extracellular matrix, and extracellular space was significantly lower due to the emergence of HCPs associated with the cytoplasm, melanosome, and ER. This result suggests that HCPs in the culture supernatants during the exponential growth phase include a high percentage of extracellular proteins secreted from viable cells, whereas HCPs at the end of the culture process involve a significant portion of intracellular proteins resulting from cell lysis.

### Cluster analysis of quantified HCPs

To evaluate the changes in HCP concentration in the culture supernatants during the cultures, relative concentration changes of quantified HCPs were visualized through a heat map. A compressed heat map provides an overview of all values in the dataset, in row-column format. Furthermore, clustering analysis of quantified HCPs was performed manually according to their concentration profiles during the cultures.

**Figure 8:**
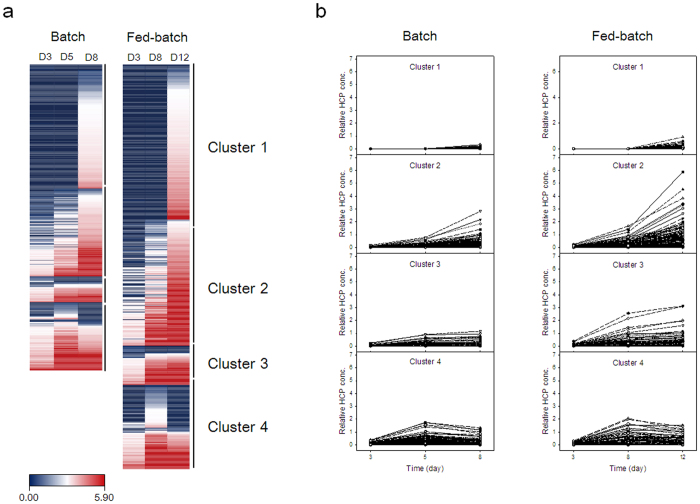
Heat map (**a**) and four established clusters of quantified HCPs (**b**) during batch and fed-batch cultures.

Figure 8 shows the heat map and 4 clusters of quantified HCPs during the cultures. In the heat map, blue corresponds to low abundances and red to high abundances. The scale extends from 0.00 (blue) to 5.90 (red) as relative concentrations. In both types of culture, more than half of the quantified HCPs (350 proteins in batch culture and 391 proteins in fed-batch culture) were hardly detected, reaching almost zero in the scale, in exponential growth phase. In general, the HCP concentration increased at the end of the cultures, indicating that a number of proteins were released from dead cells as a result of cell lysis. However, the concentrations of some HCPs tended to decrease at the end of the cultures (Fig. 8a). For further analysis, HCPs were classified in four clusters according to their concentration profiles during the cultures (Fig. 8b). Cluster 1 represents HCPs that were detected only at the end of the cultures. Cluster 2 and cluster 3 represent HCPs whose concentrations increased throughout the cultures, but either at a faster or slower rate toward the end of the cultures, respectively. Cluster 4 represents HCPs whose concentrations were the highest in the middle of culture process, but decreased in its later phase. As summarized in Supplementary Table S7, 215, 152, 45, and 118 proteins in batch culture and 218, 178, 55, and 118 proteins in fed-batch culture were grouped into clusters 1, 2, 3, and 4, respectively.

To understand the biological functions of the clustered HCPs, ClueGO analysis of the four clustered HCPs groups shown in Fig. 8B was performed to construct key functional and structural groups based on the KEGG pathway (see Supplementary Fig. S2). Many HCPs were associated with lysosome (33 proteins), glycan degradation (8 proteins), and *N*-glycan biosynthesis (5 proteins), which potentially affect the quality attributes of mAbs. These HCPs were themselves classified into various clusters. For example, cathepsin D (Ctsd) generates C-terminal heavy chain fragments by cleaving mAbs^12^. Ctsd and other cathepsin proteases including Ctsa, Ctsb, Ctsl, and Ctsz were associated with lysosome and classified in cluster 2 for both batch and fed-batch cultures. Legumain (Lgmn) is also a lysosomal protease and forms acidic charge variants by removing an amide functional group from the asparagine residues of mAbs^16^. Lgmn was classified into cluster 3 and 4 in batch and fed-batch cultures, respectively. Beta-galactosidase 1 (Glb1) and beta-1,4-galactosyltransferase (B4galt1) directly affect galactosylation of mAbs^17^. Glb1 was associated with lysosome in cluster 3 and B4galt1 was associated with *N*-glycan biosynthesis in cluster 4 in both batch and fed-batch cultures. In general, the concentration profiles of these HCPs correlated with changes in mAb quality during the cultures.

## Discussion

Maintaining good quality of mAbs in fed-batch cultures of rCHO cells is challenging because HCPs, including proteases and glycosidases, which are released from dead cells and secreted from viable cells, accumulate, reaching high enough levels to impair the mAb quality. Therefore, to characterize HCPs potentially affecting mAb quality, the detailed analysis of HCPs in the culture supernatant of fed-batch cultures of rCHO cells producing rituximab was performed in this study. Because of the masking effect generated by highly abundant mAbs, low abundant HCPs in the culture supernatants were difficult to characterize. However, after removal of the mAbs from the culture supernatants using protein A chromatography, HCPs in both batch and fed-batch cultures could be analyzed using LC-MS/MS and bioinformatics tools.

Due to higher cell concentration and longer culture duration, more HCPs were identified and quantitated in fed-batch cultures than in batch cultures. Among the HCPs identified in both batch (1934 proteins) and fed-batch cultures (2145 proteins), approximately 73–74% of them were identified throughout the cultures, including the exponential growth phase with a cell viability over 95%, suggesting that a number of HCPs were secreted from viable cells. In fact, when comparing the top 30 most abundant HCPs at each sampling time, eight HCPs (Clu, Timp1, Ywhae, decorin (Dcn), sulfated protein1 (Psap), dickkopf-related protein 3 (Dkk3), nidogen 1 (Nid1), and peptidyl-prolyl cis-trans isomerase (Ppi)) were identified conjointly in both the batch and fed-batch cultures (see Supplementary Table S7). These HCPs are located in either the cytoplasm or extracellular region and are secreted proteins. In addition to these 8 HCPs, 11 more HCPs (annexin (Anx), Rho GDP-dissociation inhibitor 1 (Arhgdia), Cfl1, glutathione S-transferase P (Gstp), 78 kDa glucose-regulated protein (Hspa5), phosphoglycerate kinase (Pgk), pyruvate kinase (Pkm), Prdx1, phosphserine aminotransferase (Psat), 60S acidic ribosomal protein P2 (Rplp2), and transketolase (Tkt)), which were among the top 30 most abundant HCPs at the end of both the batch and fed-batch cultures, are also secreted proteins. Although they were not among the top 30 most abundant HCPs, many HCPs such as collagens, tubulins, fibronectins, and proteoglycans were associated with membrane proteins including vesicle- and lysosome-related ones. These proteins are secreted by exocytosis after being sorted in the trans-Golgi network into transport vehicles^18^. Furthermore, the concentration of lactate dehydrogenase (Ldh), which is most widely used to quantify the degree of cell lysis, increased significantly toward the end of the cultures, but were not part of the top 30 most abundant HCPs at the end of neither the batch nor the fed-batch cultures. Accordingly, a significant portion of HCPs in the culture supernatants was secreted from viable cells.

ClueGO analysis of HCPs, classified into four clusters according to their concentration profiles during the cultures, provided further understanding of their cellular components or biological functions. As shown in Supplementary Fig. S2, abundant HCPs in cluster 1 were associated to transcription (spliceosome) and translation (RNA transport, aminoacyl-tRNA biosynthesis, and mRNA surveillance). In cluster 2, HCPs associated with carbohydrate metabolism and protein processing in ER, particularly molecular chaperones affecting protein folding and assembly such as Prdx1, Hspa5, and Pdi, were abundant. In addition, glutathione-related HCPs such as glutathione synthetase (Gss), gamma-glutamylcyclotransferase (Ggct), and glutathione S-transferases (Gstm, Gsto, Gstp, and Gstt) were abundant in cluster 2. In cluster 3, glycan degradation-related HCPs, including aspartylglucosaminidase (Aga), alpha-glucosidase (Gaa), Glb1, and beta-hexosaminidase b (Hexb), were highly represented. Furthermore, HCPs associated with lysosome were also abundant. In cluster 4, *N*-glycan biosynthesis-related HCPs, which affect terminal mannose and galactose residues of *N*-linked glycans, were abundant.

The mAb quality attributes, with regard to aggregation, charge variants, and *N*-glycosylation, varied during the cultures. The fragmentation of therapeutic proteins by HCPs in the culture supernatants has been observed in rCHO cells producing mAbs^9^, Fc-fusion proteins^7^,^19^,^20^, interferon γ^21^, factor VIII^8^, and erythropoietin (EPO)^22^. Cathepsins in the culture supernatants have been reported as enzymes that break down proteins^23^,^24^,^25^,^26^. In particular, Ctsd cleaves mAbs, generating C-terminal heavy chain fragments^12^. In this study, five different cathepsins (Ctsa, Ctsb, Ctsd, Ctsl, and Ctsz) were identified, with their concentrations gradually increasing during the cultures (see Supplementary Table S7). The amount of smaller fragments of mAbs also increased significantly during the culture, suggesting that the increased concentration of Ctsd is in part responsible for the increased fragmentation of mAbs.

MAbs were chemically and enzymatically modified during cultures, resulting in the formation of charge variants and the alteration of their isoelectric pH (pI) values^27^,^28^. Rituximab, in particular, suffered an increase in its amount of acidic charge variants over culture time^28^. In this study, acidic charge variants of mAbs also increased gradually during the cultures (Fig. 3b,e). It was reported that acidic charge variants are generated by a number of modifications such as deamidation of asparagine or glutamine^29^,^30^, sialylation^31^, reduced disulfide bonds, and C-terminal lysine cleavage^32^. Deamidation of asparagine residue, a common mechanism generating acidic charge variants, is mediated by asparagine endopeptidase^29^,^30^ and Lgmn^16^. In this study, Lgmn concentration increased during the cultures, suggesting that the increased acidic charge variants of mAbs was in part due to both asparagine deamidation by Lgmn and C-terminal cleavage by Ctsd.

The terminal galactose residues of mAbs affect the complement dependent cytotoxicity (CDC) by altering the binding affinity of mAbs to C1q in the complement system^33^. An increase in terminal galactose content of rituximab increased CDC activity^34^. Therefore, it is important to maintain galactosylation of rituximab during rCHO cell cultures. However, the proportion of terminal galactose residues (G1F and G2F) of mAbs decreased during the cultures (Fig. 3c,f). Glb1 and B4galt1 are directly involved in galactosylation of mAbs. Glb1 removes the terminal galactose linked to *N*-acetylglucosamine (GlcNAc) in the *N*-linked glycan^35^, and B4galt1 transfers the galactose efficiently to GlcNAc^36^. In this study, Glb1 was not among the top 30 most abundant HCPs, but its concentration in the culture supernatants increased during the cultures, which contributed in part to decreased terminal galactosylation of mAbs. In addition, HCPs related to *N*-linked glycan biosynthesis such as B4galt1, Hexb, and mannosidase alpha class 2A member 1 (Man2a1), mannosidase alpha class 2B member 1 (Man2b1), and fucosidase alpha-L-1 (Fuca1) were quantitated during batch and/or fed-batch cultures. These enzymes, which were classified into cluster 3 or 4, are involved in *N*-glycan chain extension, galactosylation, and *N*-glycan degradation^37^. In particular, B4galt1 concentration was almost zero at the end of both the batch and fed-batch cultures, suggesting that a low expression level of enzymes involved in *N*-glycan synthesis also contributed in part to the decreased galactosylation level of the mAbs at the end of the cultures.

Many other proteases have been reported to affect the quality of therapeutic proteins produced in rCHO cells. Metalloproteinase 3 (MMP3), MMP10, and MMP12 degrade the recombinant factor VIII, resulting in protein heterogeneity, denaturation, and attenuation of protein function^8^. Gelatinase, also known as MMP9, cleaves interferon-β^38^. Carboxypeptidase (Cpd) removes both the C-terminal lysine and arginine of IgG, EPO, and tissue plasminogen activator^39^,^40^, and serine and threonine class of proteases cleave the N-terminal residues of glucagon like peptide-1-antibody fusion proteins^41^. In addition, acidic protease, as well as serine and cysteine classes of proteases, cleave IgG1 and recombinant Fc-fusion proteins, respectively^42^. As summarized in Supplementary Table S8, all these proteases were detected and/or quantitated in the culture supernatants in both batch and fed-batch cultures. Furthermore, the concentration of these proteases in fed-batch cultures was much higher than that observed in batch cultures.

In the formulation of therapeutic proteins, surfactants such as polysorbates 20 (PS20) and 80 are commonly used for both preventing surface adsorption and as stabilizers against protein denaturation and aggregation^43^. Recently, PS80 degradation was observed during the long-term storage of CHO-derived purified mAbs with detectable quantities of endogenous lysosomal phospholipase A2 isomer X1 (Lpla2)^44^. PS degradation resulted in the generation of free fatty acids, which precipitated to form particles during long-term storage and impacted product quality^45^. In this study, Lpla2 was not identified in any of the culture supernatants. However, phospholipase A2 activating protein (Plaa) was identified and quantified in both batch and fed-batch cultures. Thus, Plaa should be removed during the mAb purification steps for long-term storage of mAbs formulated with PS.

CHO cell lines derived from the CHO-K1, DG44, and CHO-S lineages show several different phenotypic features due to genomic heterogeneity^46^. Therefore, the HCP concentration profiles during culture may differ among rCHO cell lines. It would be interesting to investigate the HCP concentration profiles during the cultures of other rCHO cell lines.

In conclusion, HCPs accumulated extracellularly in batch and fed-batch cultures of a mAb-producing rCHO cell line were identified and quantified using LC-MS/MS. Quantitative LC-MS/MS results were combined with GO and functional analysis of HCPs to characterize their effects on the quality attributes of mAbs. In general, the concentration profiles of HCPs affecting mAb quality correlated with changes in mAb quality during the cultures. The dataset of HCPs obtained in this study provides insights toward determining the proper target proteins and thereby maintain good mAb quality during the cultures through the addition of appropriate protease inhibitors and/or through CHO cell engineering.

## Methods

### Cell line and culture maintenance

The rCHO cell line producing rituximab (SM-0.025) was established by transfection of a vector containing glutamine synthetase (GS) and antibody genes into GS knockout CHO-K1 cells using Lipofectamine 2000 (Invitrogen, Carsbad, CA), following the manufacturer’s protocols, and subsequent selection using 25 μM methionine sulfoximine (MSX, Sigma-Aldrich, St. Louis, MO)^47^. SM-0.025 cells were adapted to grow in a serum-free suspension culture in 125 mL Erlenmeyer flasks (Corning, Corning, NY) containing 50 mL of culture medium and incubated in a climo-shaking incubator (ISF1-X, Adolf Kuhner AG, Birsfelden, Switzerland). The culture medium was protein-free, chemically defined PowerCHO2CD (Lonza, Basel, Switzerland) supplemented with 1 × GSEM (Sigma-Aldrich, St. Louis, MO) and 25 μM MSX.

### Cell culture

Exponentially growing cells were inoculated at 3.0 × 10^5^ cells/mL into a bioreactor (New Brunswick Scientific, Edison, NJ) containing 2.0 L of culture medium. Bioreactor cultures were maintained at 37 °C, pH 7.2, 50% air saturation, and an agitation speed of 50 rpm. Samples were taken every 24 h to determine viable cell concentrations. Culture supernatants were aliquoted and kept frozen at −70 °C for later analyses. For fed-batch cultures, three different feed supplements (protein-free, chemically defined Cell Boost 2, Cell Boost 5, and Cell Boost 6, Hyclone, Logan, UT) were mixed in a ratio of 1:1:1, and dissolved in water at a concentration of 70 g/L to prepare the stock solution. This solution was then added to the culture medium at a 5% v/v ratio once every other day from day 4, until day 10. Bioreactor cultures were performed three independent times.

### Viable cell concentration and mAb assays

The cell concentration was estimated using a CountessII^®^ FL automated cell counter (Invitrogen, Waltham, MA), and viable cells were distinguished from dead cells using the trypan blue dye exclusion method. The secreted mAb concentration was quantified using an enzyme-linked immunosorbent assay, as described previously^48^. The specific mAb productivity (*q*_mAb_) was evaluated from a plot displaying the mAb concentrations against the time integral values of the viable cell concentrations^49^.

### Purification of mAbs

Culture supernatants sampled from the bioreactor were centrifuged at 300 g for 5 min without damaging cells and then filtered gently through a 0.45 μm syringe filter (Millipore, Billerica, MA) to remove cells and cell debris. Subsequently, the secreted mAbs in the culture supernatants were purified by protein A affinity chromatography using MabSelect SuRe^TM^ (GE Healthcare, Uppsala, Sweden), according to the manufacturer’s protocol. Purified mAbs were stored at −70 °C until further analysis.

### SEC-HPLC analysis

Size variants of mAbs were analyzed by SEC-HPLC under isocratic conditions using an Agilent Bio-3 SEC column (4.6 mm × 300 mm, 3.0 μm particle size, Agilent, Santa Clara, CA) according to the manufacturer’s protocol. The signal was monitored at a wavelength of 280 nm.

### WCX-HPLC analysis

Acidic and basic charge variants of mAbs were analyzed by WCX-HPLC using an Agilent bio Mab column (4.6 mm × 250 mm, 5.0 μm particle size, Agilent), according to the manufacturer’s protocol. The mobile phase A (10 mM sodium phosphate, pH 7.0) and B (0.1 M sodium choloride, pH 7.0) were used. The signal was monitored at a wavelength of 220 nm.

### UPLC analysis

For *N*-glycosylation analysis, *N*-glycans were detached from mAbs via PNGase F (New England Biolabs, Ipswich, MA) and labeled by RapiFluor-MS (Waters, Millford, MA) according to the manufacturer’s protocol. *N*-glycans were analyzed using Acquity UPLC I-class system (Waters) with Acquity UPLC Glycan BEH amide column (Waters) according to the manufacturer’s protocol. The signal was monitored at 265 nm excitation and 425 nm emission wavelengths.

### Protein extraction and digestion

After centrifugation and filtration, mAbs in the culture supernatants were eliminated by protein A affinity chromatography. The remaining proteins in the culture supernatants (300 μg) were concentrated using an Amicon^®^ ultra-15 centrifugal 10 K filter (Millipore), precipitated with 100% cold acetone (4 volumes of sample) for 10 min at −70 °C, and then washed in 600 μL of 70% cold acetone by centrifugation at 14,000 g for 10 min at 4 °C. Protein pellets were solubilized with 100 μL of 100 mM Tris buffer (pH 8.0, Sigma-Aldrich) and the protein concentration was determined using Bradford assay. Then, 0.5 μg of chicken lysozyme (Sigma-Aldrich) was spiked into an aliquot of protein sample (100 μg and 30 μL) to generate an internal standard. The resulting protein samples were reduced, alkylated, and digested with minor modifications^50^. Briefly, after adding 60 μL of 9 M urea and 30 mM dithiothreitol (Sigma-Aldrich) in 10 mL of 100 mM Tris-base (pH 8.0) to each protein sample, samples were incubated for 30 min at 37 °C. After samples were allowed to cool at 20 °C, 9 μL of 500 mM iodoacetamide (Sigma-Aldrich) was added to each sample, followed by 20 min incubation at 20 °C. After diluting the urea down to a concentration of 0.6 M by adding 771 μL of 100 mM Tris buffer (pH 8.0), protein samples were digested with trypsin (Promega, Madison, WI) at a protein-to-enzyme ratio of 50:1 at 37 °C overnight. The digestion was quenched with 50 μL of 0.1% formic acid (FA). Then, the digested peptide mixture was applied to an HLB oasis cartridge (Waters) for desalting. The peptides were eluted twice with 1 mL of 40% acetonitrile (ACN) with 0.1% FA solution and 1 mL of 60% ACN with 0.1% FA solution.

### Protein identification by Q-Exactive analysis

To collect specific MS/MS spectra, digested peptides were analyzed by online nanoflow LC-MS/MS on a NanoAcquity UPLC system (Waters) that was connected to a Q-Exactive spectrometer (Thermo Scientific, Bremen, Germany) through a nanoelectrospray ion source. Briefly, digested peptides were loaded at a flow rate of 300 nL/min by an autosampler onto a precolumn (2 cm long; ID, 180 μm; particle size, 5 μm) and an analytical column (10 cm long; ID, 150 μm; particle size, 1.7 μm), which were both packed with reversed-phase C18 material. The peptides were separated on a linear ACN gradient from 3% to 40% for 120 min and from 40% to 60% for 60 min, and peptides were eluted between 3 min and 180 min.

The Q-Exactive instrument was operated in the data-dependent mode (DDA) to switch automatically between full-scan MS and MS/MS modes of acquisition. A survey of full-scan MS spectra (350–1600 m/z) was acquired on the Orbitrap with a resolution of 70,000 at 200 m/z after ions accumulated to a 3 × 10^6^ target value, based on predictive automatic gain control (AGC) from the previous full-scan. The dynamic exclusion was set to 30 s. The 10 most intense multiply charged ions (z ≥ 2) were sequentially isolated and fragmented by higher-energy collisional dissociation (HCD) in an octopole collision cell with a fixed injection time of 60 ms, an AGC target value of 5 × 10^4^, and a resolution of 17,500. The typical mass spectrometric conditions were as follows: S-lens RF level, 65; spray voltage, 2 kV; heated capillary temperature, 320 °C; and normalized HCD collision energy, 30%. The under fill ratio, MS/MS ion selection threshold, and isolation width were set to 1%, 8.3 × 10^3^ counts, and 2 m/z, respectively. The fixed first m/z was set to 100.

### Database searching and label-free quantitation

Database searching was performed using Proteome Discoverer (SEQUEST, Thermo Fischer Scientific, ver 1.4.0.288) and Scaffold (X! Tandem, version Scaffold_4.4.6, Proteome Software, Portland, OR). The MS/MS data were queried against the Chinese hamster database (UniProt-CHO) using the following parameters: MS accuracy, 10 ppm; MS/MS accuracy, 0.8 Da for HCD; trypsin digestion with 2 missed cleavages allowed; fixed carbamidomethyl modification of cysteine, +57.0215 Da; and variable modification of oxidized methionine, +15.9949 Da. The number of peptides and proteins in the protein groups was estimated using Scaffold program. Peptide identifications were accepted if they could be established at a probability, greater than 91.0% to achieve a FDR lower than 0.4% by the Scaffold Local FDR algorithm. Protein identifications were accepted if they could be established at a probability greater than 99.0%, and they contained at least two identified peptides.

For the label-free quantitation, summation of the spectral counts, validation of MS/MS-based peptides and protein identification, and annotation of peptide into proteins were performed using the Scaffold program. The spectral count result was reported by the Scaffold program, which provides an estimate of the relative values of protein abundance among time points. For reliable normalization among sample runs, a NSAF method was used. NSAF is defined as follows: NSAF = (SpC/Mw)/Σ(SpC/Mw)N, where “SpC” represents spectral counts, “Mw” represents the protein molecular weight in kDa, and “N” represents the total number of proteins^51^.

### GO and pathway analysis

GO terms in the protein datasets were determined using Uniprot bioinformatics resource (http://www.uniprot.org), which allows functional classification of the identified proteins into CC, MF, BP. Functional enrichment analysis was performed using ClueGO, which facilitates the visualization of functionally related genes displayed as a clustered network and pathway^52^,^53^. The statistical test used for the enrichment was based on the two-sided hypergeometric option with Bonferroni step-down correction and a kappa score of 0.4.

## Additional Information

**How to cite this article:** Park, J. H. *et al*. Proteomic Analysis of Host Cell Protein Dynamics in the Culture Supernatants of Antibody-Producing CHO Cells. *Sci. Rep.* **7**, 44246; doi: 10.1038/srep44246 (2017).

**Publisher's note:** Springer Nature remains neutral with regard to jurisdictional claims in published maps and institutional affiliations.

## Supplementary Material

Supplementary Information

## References

[b1] Chon J. H. & Zarbis-Papastoitsis G. Advances in the production and downstream processing of antibodies. Nat. Biotechnol. 28(5), 458–463 (2011). 10.1016/j.nbt.2011.03.015 21515428

[b2] Kumar A. . Elucidation of the CHO super-ome (CHO-SO) by proteoinformatics. J. Proteome Res. 14(11), 4687–4703 (2015). 26418914 10.1021/acs.jproteome.5b00588 PMC4721571

[b3] Li F., Vijayasankaran N., Shen A. Y., Kiss R. & Amanullah A. Cell culture processes for monoclonal antibody production. MAbs. 2(5), 466–477 (2010). 20622510 10.4161/mabs.2.5.12720 PMC2958569

[b4] Reinhart D., Damjanovic L., Kaisermayer C. & Kunert R. Benchmarking of commercially available CHO cell culture media for antibody production. Appl. Microbiol. Biotechnol. 99(11), 4645–4657 (2015). 25846330 10.1007/s00253-015-6514-4 PMC4435641

[b5] Huang Y. M. . Maximizing productivity of CHO cell-based fed-batch culture using chemically defined media conditions and typical manufacturing equipment. Biotechnol. Prog. 26(5), 1400–1410 (2010). 20945494 10.1002/btpr.436

[b6] Aboulaich N. . A novel approach to monitor clearance of host cell proteins associated with monoclonal antibodies. Biotechnol. Prog. 30(5), 1114–1124 (2014). 25044920 10.1002/btpr.1948 PMC4415537

[b7] Robert F. . Degradation of an Fc-fusion recombinant protein by host cell proteases: Identification of a CHO cathepsin D protease. Biotechnol. Bioeng. 104(6), 1132–1141 (2009). 19655395 10.1002/bit.22494

[b8] Sandberg H. . Mapping and partial characterization of proteases expressed by a CHO production cell line. Biotechnol. Bioeng. 95(5), 961–971 (2006). 16897737 10.1002/bit.21057

[b9] Gao S. X. . Fragmentation of a highly purified monoclonal antibody attributed to residual CHO cell protease activity. Biotechnol. Bioeng. 108(4), 977–982 (2011). 21404269 10.1002/bit.22982

[b10] Gramer M. J. & Goochee C. F. Glycosidase activities of the 293 and NS0 cell lines, and of an antibody-producing hybridoma cell line. Biotechnol. Bioeng. 43(5), 423–428 (1994). 18615725 10.1002/bit.260430510

[b11] Wong D. C. F., Wong K. T. K., Goh L. T., Heng C. K. & Yap M. G. S., Impact of dynamic online fed-batch strategies on metabolism, productivity and N-glycosylation quality in CHO cell cultures. Biotechnol. Bioeng. 89(2), 164–177 (2005). 15593097 10.1002/bit.20317

[b12] Bee J. S. . Trace levels of the CHO host cell protease cathepsin D caused particle formation in a monoclonal antibody product. Biotechnol. Prog. 31(5), 1360–1369 (2015). 26259961 10.1002/btpr.2150

[b13] Dixit N., Salamat-Miller N., Salinas P. A., Taylor K. D. & Basu S. K. Residual host cell protein promotes polysorbate 20 degradation in a sulfatase drug product leading to free fatty acid particles. J. Pharm. Sci. 105(5), 1657–1666 (2016). 27032893 10.1016/j.xphs.2016.02.029

[b14] Slade P. G., Hajivandi M., Bartel C. M. & Gorfien S. F. Identifying the CHO secretome using mucin-type O-linked glycosylation and click-chemistry. J. Proteome Res. 11(12), 6175–6186 (2012). 23140450 10.1021/pr300810f

[b15] Dorai H. . Proteomic analysis of bioreactor cultures of an antibody expressing CHOGS cell line that promotes high productivity. J. Proteomics Bioinform. 6(5), 98–108 (2013).

[b16] Dall E., Fegg J. C., Briza P. & Brandstetter H. Structure and mechanism of an aspartimide-dependent peptide ligase in human legumain. Angew. Chem. Int. Ed. Engl. 54(10), 2917–2921 (2015). 25630877 10.1002/anie.201409135 PMC4506564

[b17] Thomann M. . *In vitro* glycoengineering of IgG1 and its effect on Fc receptor binding and ADCC activity. PLoS One. 10(8) (2015). 10.1371/journal.pone.0134949 PMC4534130 26266936

[b18] Alberts B. . Transport from the trans Golgi network to lysosomes. MBoC. Vol. 4 (2002).

[b19] Tait A. S., Hogwood C. E., Smales C. M. & Bracewell D. G. Host cell protein dynamics in the supernatant of a mAb producing CHO cell line. Biotechnol. Bioeng. 109(4), 971–982 (2012). 22124969 10.1002/bit.24383

[b20] Dorai H. . Development of mammalian production cell lines expressing CNTO736, a glucagon like peptide-1-MIMETIBODY: factors that influence productivity and product quality. Biotechnol. Bioeng. 103(1), 162–176 (2009). 19137588 10.1002/bit.22217

[b21] Mols J., Peeters-Joris C., Wattiez R., Agathos S. N. & Schneider Y. J. Recombinant interferon-T secreted by Chinese hamster ovary-320 cells cultivated in suspension in protein-free media is protected against extracellular proteolysis by the expression of natural protease inhibitors and by the addition of plant protein hydrolysates to the culture medium. In Vitro Cell Dev-AN. 41 **(3–4)**, 83–91 (2005). 10.1290/0411075.1 16029078

[b22] Yang M. & Butler M. Enhanced erythropoietin heterogeneity in a CHO culture is caused by proteolytic degradation and can be eliminated by a high glutamine level. Cytotechnology. 34 **(1–2)**, 83–99 (2000). 19003383 10.1023/A:1008137712611 PMC3449730

[b23] Cuervo A. M., Mann L., Bonten E. J., d’Azzo A. & Dice J. F. Cathepsin A regulates chaperone-mediated autophagy through cleavage of the lysosomal receptor. EMBO J. 22(1), 47–59 (2003). 12505983 10.1093/emboj/cdg002 PMC140041

[b24] Foghsgaard L. . Cathepsin B acts as a dominant execution protease in tumor cell apoptosis induced by tumor necrosis factor. J. Cell. Biol. 153(5), 999–1009 (2001). 11381085 10.1083/jcb.153.5.999 PMC2174340

[b25] Salminen A. & Gottesman M. M. Inhibitor studies indicate that active cathepsin L is probably essential to its own processing in cultured fibroblasts. Biochem. J. 272(1), 39–44 (1990). 2264836 10.1042/bj2720039 PMC1149653

[b26] Bhutani N., Piccirillo R., Hourez R., Venkatraman P. & Goldberg A. L. Cathepsins L and Z are critical in degrading polyglutamine-containing proteins within lysosomes. J. Biol. Chem. 287(21), 17471–17482 (2012). 22451661 10.1074/jbc.M112.352781 PMC3366842

[b27] Vlasak J. & Ionescu R. Heterogeneity of monoclonal antibodies revealed by charge-sensitive methods. Curr. Pharm. Biotechnol. 9(6), 468–481 (2008). 19075686 10.2174/138920108786786402

[b28] Wang W., Singh S., Zeng D. L., King K. & Nema S. Antibody structure, instability, and formulation. J. Pharm. Sci. 96(1), 1–26 (2007). 16998873 10.1002/jps.20727

[b29] Dada O. O., Jaya N., Valliere-Douglass J. & Salas-Solano O. Characterization of acidic and basic variants of IgG1 therapeutic monoclonal antibodies based on non-denaturing IEF fractionation. Electrophoresis. 36(21), 2695–2702 (2015). 10.1002/elps.201500219 26289680

[b30] Moss C. X., Matthews S. P., Lamont D. J. & Watts C. Asparagine deamidation perturbs antigen presentation on class II major histocompatibility complex molecules. J. Biol. Chem. 280(18), 18498–18503 (2005). 15749706 10.1074/jbc.M501241200

[b31] Lyubarskaya Y., Houde D., Woodard J., Murphy D. & Mhatre R. Analysis of recombinant monoclonal antibody isoforms by electrospray ionization mass spectrometry as a strategy for streamlining characterization of recombinant monoclonal antibody charge heterogeneity. Anal. Biochem. 348(1), 24–39 (2006). 16289440 10.1016/j.ab.2005.10.003

[b32] Antes B. . Analysis of lysine clipping of a humanized Lewis-Y specific IgG antibody and its relation to Fc-mediated effector function. J. Chromatogr. B. 852 **(1–2)**, 250–256 (2007). 10.1016/j.jchromb.2007.01.024 17296336

[b33] Hodoniczky J., Zheng Y. Z. & James D. C. Control of recombinant monoclonal antibody effector functions by Fc N-glycan remodeling *in vitro*. Biotechnol. Prog. 21(6), 1644–1652 (2005). 16321047 10.1021/bp050228w

[b34] Raju T. S. Terminal sugars of Fc glycans influence antibody effector functions of IgGs. Curr. Opin. Immunol. 20(4), 471–478 (2008). 18606225 10.1016/j.coi.2008.06.007

[b35] Dragosits M. . Recombinant Aspergillus beta-galactosidases as a robust glycomic and biotechnological tool. Appl. Microbiol. Biotechnol. 98(8), 3553–3567 (2014). 24037406 10.1007/s00253-013-5192-3 PMC3973953

[b36] Lee J., Sundaram S., Shaper N. L., Raju T. S. & Stanley P. Chinese hamster ovary (CHO) cells may express six beta 4-galactosyltransferases (beta 4GalTs) - Consequences of the loss of functional beta 4GalT-1, beta 4GalT-6, or both in CHO glycosylation mutants. J. Biol. Chem. 276(17), 13924–13934 (2001). 11278604 10.1074/jbc.M010046200

[b37] Gramer M. J. & Goochee C. F. Glycosidase activities in Chinese-hamster ovary cell lysate and cell-culture supernatant. Biotechnol. Prog. 9(4), 366–373 (1993). 7763907 10.1021/bp00022a003

[b38] Nelissen I. . Gelatinase B/matrix metalloproteinase-9 cleaves interferon-beta and is a target for immunotherapy. Brain. 126, 1371–1381 (2003). 12764058 10.1093/brain/awg129

[b39] Depaolis A. & Sharma B. Tryptic map variation of erythropoietin resulting from carboxypeptidase B-like activity. J. Liq. Chromatogr. 17(13), 2777–2789 (1994).

[b40] Dick L. W., Qiu D. F., Mahon D., Adamo M. & Cheng K. C. C-terminal lysine variants in fully human monoclonal antibodies: Investigation of test methods and possible causes. Biotechnol. Bioeng. 100(6), 1132–1143 (2008). 18553400 10.1002/bit.21855

[b41] Dorai H. . Characterization of the proteases involved in the N-terminal clipping of glucagon-like-peptide-1-antibody fusion proteins. Biotechnol. Prog. 27(1), 220–231 (2011). 21312369 10.1002/btpr.537

[b42] Chakrabarti S., Barrow C. J., Kanwar R. K., Ramana V. & Kanwar J. R. Studies to prevent degradation of recombinant Fc-fusion protein expressed in mammalian cell line and protein characterization. Int. J. Mol. Sci. 17(6) (2016). 10.3390/ijms17060913 PMC4926446 27294920

[b43] Kerwin B. A. Polysorbates 20 and 80 used in the formulation of protein biotherapeutics: structure and degradation pathways. J. Pharm. Sci. 97(8), 2924–2935 (2008). 17973307 10.1002/jps.21190

[b44] Hall T., Sandefur S. L., Frye C. C., Tuley T. L. & Huang L. Polysorbates 20 and 80 degradation by group XV lysosomal phospholipase A2 isomer X1 in monoclonal antibody formulations. J. Pharm. Sci. 105(5), 1633–1642 (2016). 27056628 10.1016/j.xphs.2016.02.022

[b45] Tomlinson A., Demeule B., Lin B. & Yadav S. Polysorbate 20 degradation in biopharmaceutical dormulations: Quantification of free fatty acids, characterization of particulates, and insights into the degradation mechanism. Mol. Pharmacol. 12(11), 3805–3815 (2015). 10.1021/acs.molpharmaceut.5b00311 26419339

[b46] Lewis N. E. . Genomic landscapes of Chinese hamster ovary cell lines as revealed by the Cricetulus griseus draft genome. Nat. Biotechnol. 31(8), 759–765 (2013). 23873082 10.1038/nbt.2624

[b47] Park J. H., Noh S. M., Woo J. R., Kim J. W. & Lee G. M. Valeric acid induces cell cycle arrest at G1 phase in CHO cell cultures and improves recombinant antibody productivity. Biotechnol. J. 11(4), 487–496 (2016). 26663903 10.1002/biot.201500327

[b48] Ryu J. S., Kim T. K., Chung J. Y. & Lee G. M. Osmoprotective effect of glycine betaine on foreign protein production in hyperosmotic recombinant Chinese hamster ovary cell cultures differs among cell lines. Biotechnol. Bioeng. 70(2), 167–175 (2000). 10972928 10.1002/1097-0290(20001020)70:2<167::aid-bit6>3.0.co;2-p

[b49] Renard J. M., Spagnoli R., Mazier C., Salles M. F. & Mandine E. Evidence that monoclonal-antibody production kinetics is related to the integral of the viable cells curve in batch systems. Biotechnol. Lett. 10(2), 91–96 (1988).

[b50] Gundry R. L. . Preparation of proteins and peptides for mass spectrometry analysis in a bottom-up proteomics workflow. Curr. Protoc. Mol. Biol. Chapter 10, Unit 10, 25 (2009). 10.1002/0471142727.mb1025s88 PMC2905857 19816929

[b51] Alexova R., Haynes P. A., Ferrari B. C. & Neilan B. A. Comparative protein expression in different strains of the bloom-forming cyanobacterium Microcystis aeruginosa. Mol. Cell. Proteomics. 10(9), M110 003749 (2011). 10.1074/mcp.M110.003749 PMC3186190 21610102

[b52] Bindea G., Galon J. & Mlecnik B. CluePedia Cytoscape plugin: pathway insights using integrated experimental and in silico data. Bioinformatics. 29(5), 661–663 (2013). 23325622 10.1093/bioinformatics/btt019 PMC3582273

[b53] Bindea G. . ClueGO: a Cytoscape plug-in to decipher functionally grouped gene ontology and pathway annotation networks. Bioinformatics. 25(8), 1091–1093 (2009). 19237447 10.1093/bioinformatics/btp101 PMC2666812

