# Supplementary material for: Proteomic Analysis of Host Cell Protein Dynamics in the Culture Supernatants of Antibody-Producing CHO Cells
Source: Sci Rep. 2017 Mar 10;7:44246. doi: 10.1038/srep44246 (PMC5345005; doi:10.1038/srep44246)
Supplement: Supplementary Information [file srep44246-s1.pdf]

Supplementary Information

Proteomic Analysis of Host Cell Protein Dynamics in the Culture Supernatants of Antibody-producing CHO Cells

Jin Hyoung Park[1#], Jong Hwa Jin[2#], Myung Sin Lim[2], Hyun Joo An[3], Jong Won Kim[2*], and Gyun Min Lee[1*]

[1] Department of Biological Sciences, KAIST, 291 Daehak-ro, Yuseong-gu, Daejeon 34141, Republic of Korea

[2] New Drug Development Center, 123 Osongsaengmyeng-ro, Cheongju-si, Chungbuk 28160, Republic of Korea

[3] Graduate School of Analytical Science & Technology, Chungnam National University, 99 Daehak-ro, Yuseong-gu, Daejon 34134, Republic of Korea

* Corresponding. gyunminlee@kaist.ac.kr and jonwkim@kbio.kr

[#] These authors contributed equally to this work.

Contents:

Supplementary Figure S1 (page 4). Reproducibility between biological replications in batch and fed-batch cultures.

Supplementary Figure S2 (page 5). Functional and structural analysis of the clustered HCPs described in Figure 8 using ClueGO. The node size and color represent the term enrichment significance. (A) Batch cultures. (B) Fed-batch cultures.

Supplementary Table S1 (page 6). SEC-HPLC analysis of the aggregation of mAbs produced during the cultures shown in Figure 2.

Values are means ± standard deviations of two independent experiments.

The asterisks (*) indicate significant differences compared to day 3 (P<0.05).

Supplementary Table S2 (page 7). WCX-HPLC analysis of the charge variation of mAbs produced during the cultures shown in Figure 2.

Values are means ± standard deviations of two independent experiments.

The asterisks (*) indicate significant differences compared to day 3 (P<0.05).

Supplementary Table S3 (page 8). UPLC analysis of the *N*-linked glycosylation of mAbs produced during the cultures shown in Figure 2.

Values are means ± standard deviations of two independent experiments.

The asterisks (*) indicate significant differences compared to day 3 (P<0.05).

Supplementary Table S4 (page 9). A complete list of identified HCPs in the culture supernatants during batch and fed-batch cultures.

Supplementary Table S5 (page 65). GO analysis of identified HCPs in the culture

supernatants during batch culture.

Supplementary Table S6 (page 98). GO analysis of identified HCPs in the culture supernatants during fed-batch culture.

Supplementary Table S7 (page 129). A complete list of quantified HCPs in the culture supernatants during batch and fed-batch cultures.

Supplementary Table S8 (page 157). Proteases and glycosidases reported to affect the quality of therapeutic proteins produced from rCHO cells.

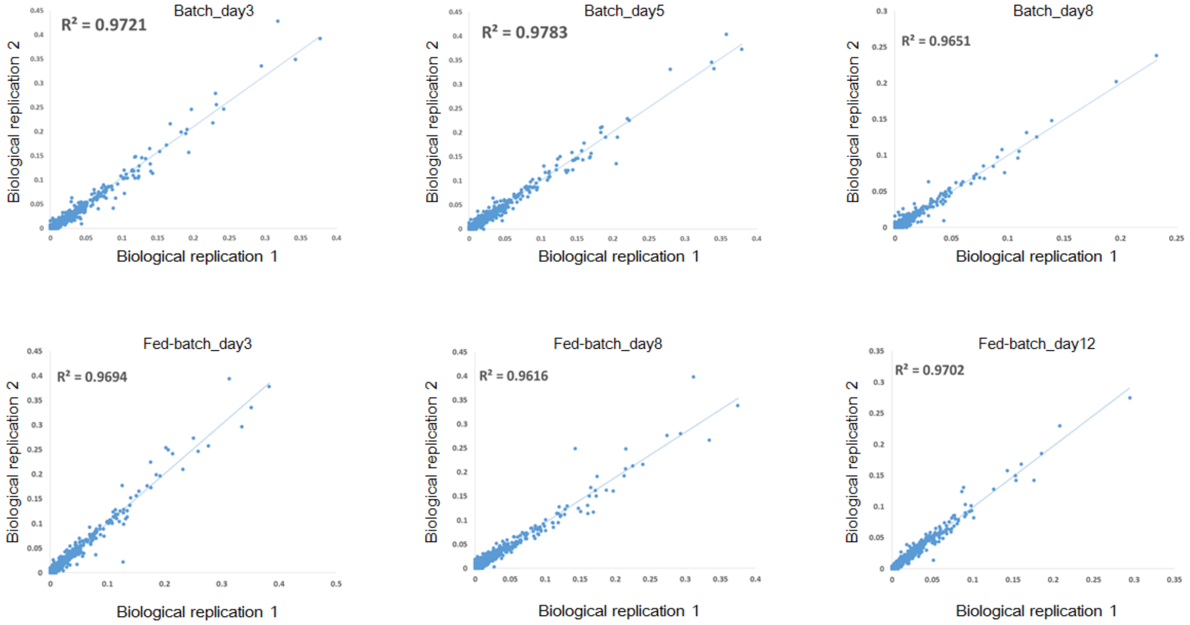

Supplementary Figure S1. Reproducibility between biological replications in batch and fed-batch cultures.

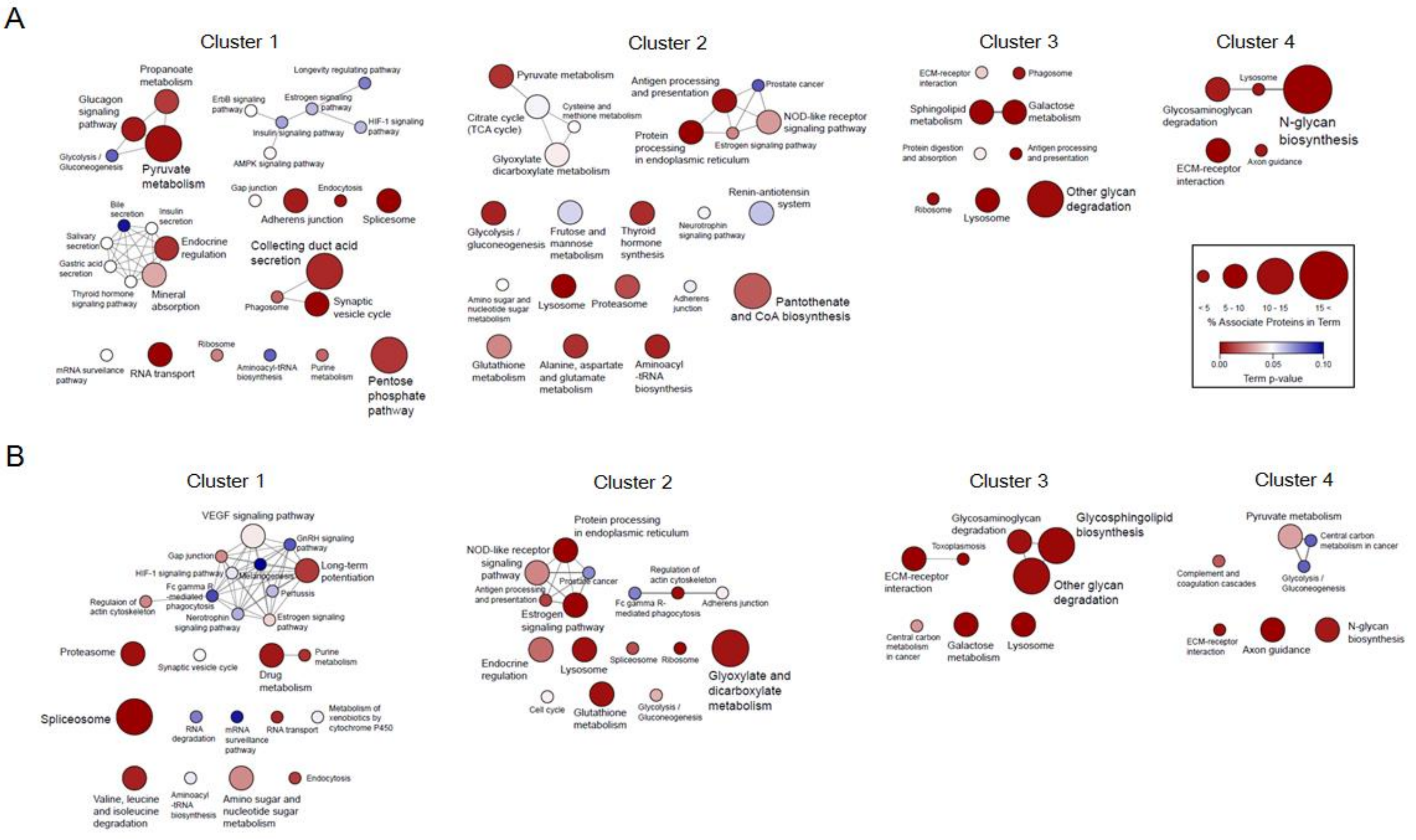

Supplementary Figure S2. Functional and structural analysis of clustered HCPs in Fig. 8 by using ClueGO. The node size and color represent the term enrichment significance. (A) Batch cultures. (B) Fed-batch cultures.

Supplementary Table S1. SEC-HPLC analysis on the aggregation of mAbs produced at bioreactor cultures shown in Fig. 2.

	Day	Main (%)	Fragment (%)
Batch cultures	Day 3	98.3 ± 1.1	0.7 ± 1.1
	Day 5	97.8 ± 0.5	2.2 ± 0.5
	Day 8	96.4 ± 0.1	3.7 ± 0.1
	Day	Main (%)	Fragment (%)
Fed-batch cultures	Day 3	98.2 ± 0.7	1.9 ± 0.7
	Day 8	96.1 ± 0.4	3.9 ± 0.4
	Day 12	93.2 ± 0.8*	6.8 ± 0.8*

Values are means ± standard deviations of two independent experiments.

The asterisk mark (*) indicates significant differences compared to Day 3 (*P*<0.05).

Supplementary Table S2. WCX-HPLC analysis on the charge variation of mAbs produced at bioreactor cultures shown in Fig. 2.

	Day	Acidic peak (%)	Main peak (%)	Basic peak (%)
Batch cultures	Day 3	18.1 ± 1.5	61.8 ± 0.1	20.1 ± 1.4
	Day 5	25.1 ± 2.9	53.1 ± 0.3*	21.9 ± 3.2
	Day 8	32.4 ± 3.1*	46.6 ± 1.3*	21.0 ± 1.8
	Day	Acidic peak (%)	Main peak (%)	Basic peak (%)
Fed-batch cultures	Day 3	16.9 ± 0.2	60.0 ± 2.7	21.1 ± 2.5
	Day 8	19.6 ± 0.9*	56.8 ± 0.9*	23.6 ± 1.9
	Day 12	30.4 ± 1.1*	48.4 ± 1.8*	21.2 ± 1.7

Values are means ± standard deviations of two independent experiments.

The asterisk mark (*) indicates significant differences compared to Day 3 ($P<0.05$).

Supplementary Table S3. UPLC analysis on the *N*-linked glycosylation of mAbs produced at bioreactor cultures shown in Fig. 2.

	Day	G0	G0F	G1	G1F	G2F	G1F+NANA	G2F+GN+NANA
Batch cultures	Day 3	2.1 ± 0.2	40.4 ± 1.2	0.8 ± 1.2	44.7 ± 0.8	9.3 ± 0.3	1.0 ± 0.5	1.8 ± 0.1
	Day 5	1.9 ± 0.1	41.5 ± 1.2	0.7 ± 0.1	44.8 ± 0.2	9.0 ± 0.1	0.7 ± 0.2	1.5 ± 1.0
	Day 8	5.8 ± 0.2*	58.6 ± 0.9*	0.9 ± 0.1	30.8 ± 0.5*	2.5 ± 0.6*	0.4 ± 0.2	1.2 ± 0.1
	Day	G0	G0F	G1	G1F	G2F	G1F+NANA	G2F+GN+NANA
Fed-batch cultures	Day 3	2.0 ± 0.2	40.9 ± 0.6	0.4 ± 0.1	44.8 ± 0.0	9.4 ± 0.1	0.6 ± 0.2	2.0 ± 0.8
	Day 8	3.2 ± 0.1	44.2 ± 0.2*	1.0 ± 0.1	44.0 ± 0.3	5.8 ± 0.2	0.5 ± 0.2*	1.4 ± 0.4
	Day 12	3.4 ± 0.1	55.6 ± 0.1*	1.7 ± 0.1	33.3 ± 0.6*	4.9 ± 0.1*	0.4 ± 0.2*	0.9 ± 0.3

Values are means ± standard deviations of two independent experiments.

The asterisk mark (*) indicates significant differences compared to Day 3 ($P<0.05$)

Supplementary Table S4. A complete list of identified HCPs in the culture supernatants during batch and fed-batch cultures.

Batch culture	
Accession	Protein Name
G3H0E4_CRIGR	Chondroitin sulfate proteoglycan 4
G3HGW6_CRIGR	Laminin subunit alpha-5
G3HIM1_CRIGR	Basement membrane-specific heparan sulfate proteoglycan core protein
G3HNJ3_CRIGR	Clusterin
G3HBI1_CRIGR	Peroxidasin-like
G3HG25_CRIGR	Laminin subunit gamma-1
G3HAC6_CRIGR	Titin
G3HCL6_CRIGR	Complement C3
G3I278_CRIGR	Laminin subunit beta-1
G3I664_CRIGR	Procollagen C-endopeptidase enhancer 1
G3GUR1_CRIGR	Complement C1r-A subcomponent
G3I1H5_CRIGR	Legumain
G3GR64_CRIGR	Inter-alpha-trypsin inhibitor heavy chain H5
G3I8R9_CRIGR	78 kDa glucose-regulated protein
G3H3E4_CRIGR	Galectin-3-binding protein
G3H3Q1_CRIGR	Pyruvate kinase
G3H584_CRIGR	SPARC
G3H4T5_CRIGR	Glypican-1
G3H7I6_CRIGR	Sulfhydryl oxidase
G3HWE4_CRIGR	Nidogen-1
G3HPT8_CRIGR	Suprabasin
G3HJG6_CRIGR	Decorin
G3HWE7_CRIGR	Dickkopf-related protein 3
G3HQM6_CRIGR	Endoplasmin
G3H8V1_CRIGR	Matrix metalloproteinase-9
G3GUR0_CRIGR	Calcium-dependent serine proteinase
G3HTE5_CRIGR	Lysosomal alpha-glucosidase
G3GUU5_CRIGR	Transketolase
G3IBH0_CRIGR	Metalloproteinase inhibitor 1
G3H6V7_CRIGR	Lipoprotein lipase
G3IF52_CRIGR	Nucleobindin-2
G3HHV4_CRIGR	Thrombospondin-1
G3I1Y9_CRIGR	Sulfated glycoprotein 1
G3IBK2_CRIGR	Filamin-A
G3H8C9_CRIGR	Semaphorin-3B
G3HMG4_CRIGR	Amyloid beta A4 protein
G3GXD7_CRIGR	Fatty acid synthase
G3I437_CRIGR	Agrin
G3I3U5_CRIGR	Nidogen-1
G3GUX9_CRIGR	Bone morphogenetic protein 1
B2MG_CRIGR	Beta-2-microglobulin
G3HGM6_CRIGR	N(4)-(Beta-N-acetylglucosaminyl)-L-asparaginase
PRDX1_CRIGR	Peroxiredoxin-1
G3H0L9_CRIGR	Cathepsin B
G3H1W4_CRIGR	Tubulointerstitial nephritis antigen-like
G3I9L7_CRIGR	EGF-containing fibulin-like extracellular matrix protein 1
GSLG1_CRIGR	Golgi apparatus protein 1
G3HRK9_CRIGR	Matrix metalloproteinase-19
G3H8V4_CRIGR	Phospholipid transfer protein
G3GXS2_CRIGR	EMILIN-1
G3I3K5_CRIGR	G-protein coupled receptor 56
G3H8V5_CRIGR	Lysosomal protective protein
G3HH34_CRIGR	Collagen alpha-1(XII) chain (Fragment)
PLEC_CRIGR	Plectin (Fragment)
Q9EPP7_CRIGR	Cathepsin Z
G3IE06_CRIGR	Transmembrane protein 132A
G3I4D4_CRIGR	Ribonuclease T2
G3HQD5_CRIGR	Peptidyl-glycine alpha-amidating monooxygenase B
G3I412_CRIGR	45 kDa calcium-binding protein
G3GWQ1_CRIGR	Vinculin
G3ILK7_CRIGR	Calsyntenin-1
G3H533_CRIGR	Peptidyl-prolyl cis-trans isomerase
G3H9N4_CRIGR	Polypeptide N-acetylgalactosaminyltransferase 1
G3H1K9_CRIGR	Alpha-actinin-1
G3ICD3_CRIGR	Lactadherin
G3HAI3_CRIGR	Follistatin-related protein 1
G3HLT3_CRIGR	Syndecan
G3HRF8_CRIGR	CD166 antigen
G3HN14_CRIGR	Transitional endoplasmic reticulum ATPase
G3H8F4_CRIGR	Dystroglycan

G3IDM2_CRIGR	Cofilin-1
G3HVZ2_CRIGR	Protocadherin Fat 1
G3IDT6_CRIGR	Protein disulfide-isomerase A4
G3IDU7_CRIGR	Beta-glucuronidase
G3HC84_CRIGR	Heat shock protein HSP 90-beta
G3GTX5_CRIGR	Amyloid-like protein 2
G3HN31_CRIGR	Semaphorin-3E
G3I2T9_CRIGR	3-phosphoinositide-dependent protein kinase 1
G3I5L3_CRIGR	Annexin
G3IIE7_CRIGR	Procollagen-lysine,2-oxoglutarate 5-dioxygenase 1
G3I064_CRIGR	Neutral alpha-glucosidase AB
G3HFM4_CRIGR	Filamin-B
G3I4H6_CRIGR	Fructose-bisphosphate aldolase
G3H092_CRIGR	Coiled-coil domain-containing protein 80
G3H2W6_CRIGR	Extracellular matrix protein 1
G3I4W7_CRIGR	Cathepsin D
G3HMI3_CRIGR	Semaphorin-3C
G3IM20_CRIGR	Collagen alpha-1(Ⅲ) chain
G3HXN7_CRIGR	Beta-hexosaminidase
G3IHY5_CRIGR	6-phosphogluconate dehydrogenase, decarboxylating
G3HEV3_CRIGR	Latent-transforming growth factor beta-binding protein 1
G3I5N6_CRIGR	Insulin-like growth factor-binding protein 4
G3H3E6_CRIGR	Metalloproteinase inhibitor 2
G3HG95_CRIGR	Lamin-A/C
PDIA1_CRIGR	Protein disulfide-isomerase
G3H559_CRIGR	Alpha-mannosidase 2
G3HEI6_CRIGR	Lysyl oxidase-like 1
G3HH30_CRIGR	Aldose reductase
G3IG05_CRIGR	Annexin
G3IKH9_CRIGR	Phosphoserine aminotransferase
G3HS71_CRIGR	Vasorin
G3IBF4_CRIGR	Serine protease HTRA1
HSP7C_CRIGR	Heat shock cognate 71 kDa protein
G3H3E3_CRIGR	Soluble calcium-activated nucleotidase 1
G3I692_CRIGR	Cytosolic non-specific dipeptidase
G3GZB2_CRIGR	Acid ceramidase
G3HQL6_CRIGR	Thioredoxin reductase 1, cytoplasmic
G3H1S2_CRIGR	UDP-glucose:glycoprotein glucosyltransferase 1
G3HUC4_CRIGR	Sushi, von Willebrand factor type A, EGF and pentraxin domain-containing protein 1
G3IDP5_CRIGR	Protein CYR61
G3I7U8_CRIGR	Lysyl oxidase-like 3
Q9ERF7_CRIGR	Intercellular adhesion molecule 1
G3HZD1_CRIGR	Tenascin-X
G3IFK8_CRIGR	MAM domain-containing protein 2
G3IID2_CRIGR	Complement C1q tumor necrosis factor-related protein 5
G3HH37_CRIGR	CD109 antigen
G3GWX6_CRIGR	Leukocyte elastase inhibitor A
G3GSG4_CRIGR	Renin receptor
G3GTT2_CRIGR	C-C motif chemokine 2
G3INC5_CRIGR	Cathepsin L1
G3HHM5_CRIGR	Obscurin
G3HJ89_CRIGR	Clathrin heavy chain
G3IAQ0_CRIGR	Alpha-enolase
G3HCV4_CRIGR	Lysosomal alpha-mannosidase
G3IBG3_CRIGR	Ubiquitin activating enzyme E1
G3IHT7_CRIGR	Semaphorin-4B
G3HB74_CRIGR	Amiloride-sensitive amine oxidase [copper-containing]
HS90A_CRIGR	Heat shock protein HSP 90-alpha
G3I1V4_CRIGR	Bifunctional purine biosynthesis protein PURH
G3IF80_CRIGR	Nucleolin
G3GXB0_CRIGR	Rho GDP-dissociation inhibitor 1
G3GY17_CRIGR	Cullin-associated NEDD8-dissociated protein 1
G3H3E9_CRIGR	Dynein heavy chain 17, axonemal
G3I1V3_CRIGR	Fibronectin
G3HZ42_CRIGR	T-complex protein 1 subunit beta
G3HAP7_CRIGR	Glutathione synthetase
G3IF62_CRIGR	Ras GTPase-activating-like protein IQGAP1
G3IH63_CRIGR	Myosin-9
G3IPU3_CRIGR	14-3-3 protein epsilon
G3HB04_CRIGR	Protein disulfide-isomerase A6
G3HGQ1_CRIGR	Cation-independent mannose-6-phosphate receptor
G3HQH7_CRIGR	Olfactomedin-like protein 3
G3GZZ0_CRIGR	Aspartate aminotransferase
LEG1_CRIGR	Galectin-1

G3H352_CRIGR	Cytoplasmic dynein 1 heavy chain 1 (Fragment)
G3I9X8_CRIGR	Spectrin alpha chain, brain
G3HKX9_CRIGR	N-acetylgalactosaminyltransferase 7
G3HNY5_CRIGR	Phosphoglycerate kinase
G3GZY4_CRIGR	Lysyl oxidase-like 4
G3GVX1_CRIGR	Ribosome-binding protein 1
G3HG83_CRIGR	T-complex protein 1 subunit gamma
G3I6T1_CRIGR	Putative phospholipase B-like 2
G3IEU2_CRIGR	Protein DJ-1
G3I3Y6_CRIGR	Glutathione S-transferase P
G3H1D5_CRIGR	Retinoid-inducible serine carboxypeptidase
G3IAS8_CRIGR	Kinesin-like protein KIF27
G3GX17_CRIGR	Polypeptide N-acetylgalactosaminyltransferase 2
G3I5Z5_CRIGR	Prostaglandin reductase 1
G3H5V0_CRIGR	Importin subunit beta-1
G3H902_CRIGR	Prostaglandin F2 receptor negative regulator
G3GR73_CRIGR	Rab GDP dissociation inhibitor beta
G3IFB0_CRIGR	Serine/arginine repetitive matrix protein 2
G3HAJ0_CRIGR	Golgin subfamily B member 1
G3H2I6_CRIGR	Neural cell adhesion molecule 1
G3I2H0_CRIGR	Dipeptidyl-peptidase 3
G3HSM3_CRIGR	Thimet oligopeptidase
G3HWC3_CRIGR	Ezrin
G3I255_CRIGR	L-lactate dehydrogenase
LDLR_CRIGR	Low-density lipoprotein receptor
G3H7K5_CRIGR	T-complex protein 1 subunit epsilon
G3GZW8_CRIGR	Phosphoglycerate mutase 1
G3IBK8_CRIGR	V-type proton ATPase subunit S1
ALD2_CRIGR	Aldose reductase-related protein 2
G3IK05_CRIGR	Lactadherin (Fragment)
G3HC49_CRIGR	Thrombospondin-3
G3I936_CRIGR	Epididymal secretory protein E1
G3GZ90_CRIGR	Calumenin
G3GTX3_CRIGR	Suppressor of tumorigenicity 14 protein homolog
G3GRE1_CRIGR	Bifunctional aminoacyl-tRNA synthetase (Fragment)
G3HGP4_CRIGR	Acetyl-CoA acetyltransferase, cytosolic
G3HDQ2_CRIGR	Malate dehydrogenase
G3GU60_CRIGR	Phosphatidylethanolamine-binding protein 1
G3HKZ1_CRIGR	14-3-3 protein zeta/delta
G3HSE4_CRIGR	Proteasome subunit alpha type
G3I5H3_CRIGR	Elongation factor 1-delta
G3GYY6_CRIGR	Catalase
G3HN02_CRIGR	Talin-1
TCPA_CRIGR	T-complex protein 1 subunit alpha
G3H8K2_CRIGR	Mannosyl-oligosaccharide 1,2-alpha-mannosidase IA
G3IHN6_CRIGR	4F2 cell-surface antigen heavy chain
G3IMH4_CRIGR	Sphingomyelin phosphodiesterase
G3I7W5_CRIGR	Myosin-XVIIIa
G3H7T9_CRIGR	E3 ubiquitin-protein ligase HUWE1 (Fragment)
G3IIB1_CRIGR	Sialate O-acetylesterase
G3IKC3_CRIGR	Glutathione S-transferase Mu 6
G3HFP1_CRIGR	Radixin
G3HEJ4_CRIGR	Neogenin
PLST_CRIGR	Plastin-3
G3HLS2_CRIGR	14-3-3 protein beta/alpha
G3IBF1_CRIGR	Nucleophosmin
G3GWV9_CRIGR	Nucleoprotein TPR
G3HPZ5_CRIGR	Macrophage-capping protein
G3HC47_CRIGR	Glucosylceramidase
G3H6Y6_CRIGR	NSFL1 cofactor p47
G3HM03_CRIGR	Synaptic vesicle membrane protein VAT-1-like
G3IDN7_CRIGR	Protein FAM3C
G3HW34_CRIGR	Prolow-density lipoprotein receptor-related protein 1
G3H2C4_CRIGR	Heat shock 70 kDa protein 4 (Fragment)
G3I3A8_CRIGR	Nuclear mitotic apparatus protein 1
G3HDL6_CRIGR	Elongation factor 1-beta
G3HJ24_CRIGR	Prolyl 4-hydroxylase subunit alpha-1
G3I676_CRIGR	Zonadhesin (Fragment)
G3GY44_CRIGR	Myosin-10
G3HZ57_CRIGR	Neuropilin-1
G3IKH4_CRIGR	Collagen alpha-2(V) chain
G3GU69_CRIGR	Citron Rho-interacting kinase
G3HYP6_CRIGR	Nuclear migration protein nudC
G3HJ15_CRIGR	E3 SUMO-protein ligase RanBP2

G3H4Z5_CRIGR	C-1-tetrahydrofolate synthase, cytoplasmic
G3HMQ0_CRIGR	FK506-binding protein 9
G6PI_CRIGR	Glucose-6-phosphate isomerase
G3HJM2_CRIGR	Glycyl-tRNA synthetase
G3GRU5_CRIGR	Zinc finger protein 469
G3HFP8_CRIGR	KDEL motif-containing protein 2
G3H4A6_CRIGR	Uncharacterized protein KIAA1683
G3H697_CRIGR	Glucosidase 2 subunit beta
G3H748_CRIGR	Endoplasmic reticulum aminopeptidase 1
G3IB69_CRIGR	Laminin subunit beta-2
G3HZX6_CRIGR	Nucleobindin-1
G3IKH3_CRIGR	Collagen alpha-2(V) chain
G3GWB3_CRIGR	Alpha-2-macroglobulin receptor-associated protein
G3I2M1_CRIGR	T-complex protein 1 subunit zeta
G3I0F7_CRIGR	Alpha-N-acetylgalactosaminidase
G3HVR6_CRIGR	Procollagen-lysine,2-oxoglutarate 5-dioxygenase 2
G3HNY0_CRIGR	Transcriptional regulator ATRX
G3HK00_CRIGR	Guanine nucleotide-binding protein subunit beta-2-like 1
G3H2J8_CRIGR	Heterogeneous nuclear ribonucleoproteins A2/B1
G3I2H6_CRIGR	N-acetyllactosaminide beta-1,3-N-acetylglucosaminyltransferase
G3GS70_CRIGR	Importin-5
G3IG23_CRIGR	Alanyl-tRNA synthetase, cytoplasmic
G3I3H2_CRIGR	60S acidic ribosomal protein P2
G3H6S8_CRIGR	Inorganic pyrophosphatase
G3IAW1_CRIGR	Golgi membrane protein 1
G3IA10_CRIGR	Heterogeneous nuclear ribonucleoprotein U-like protein 1
G3HG36_CRIGR	Glutamine synthetase
G3HTI7_CRIGR	Putative G-protein coupled receptor 116 (Fragment)
G3IH84_CRIGR	Arylsulfatase A
G3HF78_CRIGR	Adenomatous polyposis coli protein 2
G3I1R2_CRIGR	Purine nucleoside phosphorylase (Fragment)
G3H935_CRIGR	Tyrosyl-tRNA synthetase, cytoplasmic
G3HAN8_CRIGR	Adenosylhomocysteinase
G3GWE9_CRIGR	Solute carrier family 2, facilitated glucose transporter member 9
G3HFM7_CRIGR	Myosin-11
G3HR95_CRIGR	Carboxypeptidase D
G3I9G7_CRIGR	Proteasome subunit alpha type
G3GT06_CRIGR	T-complex protein 1 subunit theta
G3GWR8_CRIGR	Proteasome subunit alpha type-7 (Fragment)
G3IH96_CRIGR	Eukaryotic translation initiation factor 4B
G3HMV7_CRIGR	Tissue alpha-L-fucosidase
G3ILI3_CRIGR	Seryl-tRNA synthetase, cytoplasmic
G3ILN5_CRIGR	Reticulocalbin-3
G3GSF3_CRIGR	Sushi repeat-containing protein SRPX
G3GUS7_CRIGR	Dynein heavy chain 12, axonemal
G3HDQ9_CRIGR	UDP-GlcNAc:betaGal beta-1,3-N-acetylglucosaminyltransferase 2
G3H1W1_CRIGR	Collagen alpha-1(XVI) chain
G3GZQ0_CRIGR	Apolipoprotein B-100
G3HRJ2_CRIGR	182 kDa tankyrase-1-binding protein
G3GZ85_CRIGR	Nuclease domain-containing protein 1
PCNA_CRIGR	Proliferating cell nuclear antigen
G3H7Z2_CRIGR	Transgelin-2
G3GUI3_CRIGR	FK506-binding protein 4
G3HUU6_CRIGR	Protein S100-A11
G3HR08_CRIGR	Cytosol aminopeptidase
G3HIN8_CRIGR	Leucine zipper protein 1
G3HUI4_CRIGR	Lysosomal Pro-X carboxypeptidase
G3HAF4_CRIGR	Splicing factor 3B subunit 3
G3HU28_CRIGR	F-actin-capping protein subunit beta
G3HZF4_CRIGR	Chloride intracellular channel protein 1
G3HBI9_CRIGR	Leukotriene A-4 hydrolase
G3HIT7_CRIGR	Microtubule-associated protein 1B
PRDX2_CRIGR	Peroxiredoxin-2
G3HWS4_CRIGR	Chromodomain-helicase-DNA-binding protein 8
G3HQ45_CRIGR	Eukaryotic translation initiation factor 2 subunit 3
G3HIQ0_CRIGR	Threonyl-tRNA synthetase, cytoplasmic
G3HYB7_CRIGR	T-complex protein 1 subunit eta
G3HRJ5_CRIGR	Calcitonin gene-related peptide type 1 receptor
G3HW66_CRIGR	Multiple inositol polyphosphate phosphatase 1
G3H497_CRIGR	Inositol-3-phosphate synthase 1
G3HWP5_CRIGR	Chromodomain-helicase-DNA-binding protein 6
G3H470_CRIGR	Transmembrane 6 superfamily member 2
G3HRG8_CRIGR	Endoplasmic reticulum resident protein ERp44 (Fragment)
G3H284_CRIGR	Endoplasmic reticulum resident protein 29

G3H066_CRIGR	V-type proton ATPase catalytic subunit A
G3HYJ9_CRIGR	Moesin
G3I073_CRIGR	Elongation factor 1-gamma
G3IKN5_CRIGR	Glutathione S-transferase omega-1
G3GZ94_CRIGR	Filamin-C
G3I3V6_CRIGR	Discoidin, CUB and LCCL domain-containing protein 2
G3H6I5_CRIGR	Interleukin-1 receptor-like 1
G3GTA7_CRIGR	Ras-specific guanine nucleotide-releasing factor 1
G3HN88_CRIGR	Adenylyl cyclase-associated protein
G3H6B5_CRIGR	Interleukin enhancer-binding factor 3
G3I1P5_CRIGR	SH3 domain-binding glutamic acid-rich-like protein
G3II69_CRIGR	DNA damage-binding protein 1
G3H1M4_CRIGR	Eukaryotic translation initiation factor 2 subunit 1
G3IEB7_CRIGR	Out at first protein-like
G3IN86_CRIGR	Dipeptidyl-peptidase 2
G3HRK1_CRIGR	Receptor tyrosine-protein kinase erbB-3
G3H3G9_CRIGR	Septin-9
G3HHH6_CRIGR	Salivary plasminogen activator alpha 2
G3HBD3_CRIGR	Nucleoside diphosphate kinase
G3H177_CRIGR	Vesicular integral-membrane protein VIP36
G3H3X1_CRIGR	Apoptosis-inducing factor 1, mitochondrial
G3I7A8_CRIGR	Sortilin (Fragment)
G3HD97_CRIGR	Thioredoxin domain-containing protein 5
G3IEY9_CRIGR	14-3-3 protein theta
G3HLK3_CRIGR	Granulins
G3IIZ6_CRIGR	Structural maintenance of chromosomes protein 3
G3HZD3_CRIGR	Tenascin-X
G3H510_CRIGR	Talin-2
G3H638_CRIGR	Activator of 90 kDa heat shock protein ATPase-like 1
G3HSF3_CRIGR	Proteasome subunit alpha type
G3HP75_CRIGR	D-3-phosphoglycerate dehydrogenase
G3HA51_CRIGR	Procollagen-lysine,2-oxoglutarate 5-dioxygenase 3
G3H0C2_CRIGR	Proteasome subunit alpha type
G3H8A8_CRIGR	Mesencephalic astrocyte-derived neurotrophic factor
G3HKB0_CRIGR	Peptidyl-prolyl cis-trans isomerase
G3HEJ5_CRIGR	ADP-dependent glucokinase
G3IGL0_CRIGR	Putative heterogeneous nuclear ribonucleoprotein A1-like protein 3
G3HKQ7_CRIGR	Microtubule-associated protein
G3GRM2_CRIGR	Disintegrin and metalloproteinase domain-containing protein 10
G3HDE3_CRIGR	Proteasome activator complex subunit 2
G3HBD4_CRIGR	Nucleoside diphosphate kinase
G3HRK0_CRIGR	Proliferation-associated protein 2G4
G3H316_CRIGR	AP-3 complex subunit beta-1
G3HEE8_CRIGR	Alpha-L-iduronidase
IMDH2_CRIGR	Inosine-5'-monophosphate dehydrogenase 2
G3GZD2_CRIGR	Phosphoglucomutase-1
G3HCX3_CRIGR	Deoxyribonuclease-2-alpha
G3I8P7_CRIGR	N-acetylglucosamine-6-sulfatase
G3I2M2_CRIGR	Phosphoserine phosphatase
G3H3P8_CRIGR	Beta-hexosaminidase
SYRC_CRIGR	RecName: Full
G3H0S4_CRIGR	Sorbitol dehydrogenase
G3HK90_CRIGR	14-3-3 protein eta
G3IMD1_CRIGR	Alcohol dehydrogenase class-3
G3IEG2_CRIGR	Serpin B6
G3HEY8_CRIGR	Nucleotide exchange factor SIL1
G3HA54_CRIGR	Plasminogen activator inhibitor 1
G3I0L2_CRIGR	Beta/gamma crystallin domain-containing protein 3
G3H7B3_CRIGR	Galectin
G3HEB6_CRIGR	TGF-beta receptor type III
G3GT45_CRIGR	Interferon-alpha/beta receptor beta chain
G3HFY9_CRIGR	Ubiquitin-conjugating enzyme E2 L3
G3I740_CRIGR	Pantetheinase
G3GT05_CRIGR	T-complex protein 1 subunit theta
G3GTB7_CRIGR	Heterogeneous nuclear ribonucleoprotein Q
G3I8S7_CRIGR	Polyadenylate-binding protein 1
G3HMM0_CRIGR	Receptor-type tyrosine-protein phosphatase zeta
G3H928_CRIGR	Adenylate kinase 2, mitochondrial
G3HCL3_CRIGR	Tumor necrosis factor ligand superfamily member 9
G3H7F0_CRIGR	Tudor domain-containing protein 7
G3H604_CRIGR	Alpha-galactosidase A
G3HL16_CRIGR	Splicing factor 3B subunit 1
G3HBX0_CRIGR	N-acetylglucosamine-1-phosphotransferase subunit gamma
G3HRM8_CRIGR	Nascent polypeptide-associated complex subunit alpha, muscle-specific form

G3H4A9_CRIGR	Mpv17-like protein 2
G3HQK5_CRIGR	UDP-N-acetylhexosamine pyrophosphorylase
G3H7L5_CRIGR	Receptor-type tyrosine-protein phosphatase gamma (Fragment)
G3GZL7_CRIGR	Calcyclin-binding protein
CH60_CRIGR	60 kDa heat shock protein, mitochondrial
G3HNY4_CRIGR	Phosphoglycerate kinase
G3HY03_CRIGR	D-dopachrome decarboxylase
G3HF46_CRIGR	Polypyrimidine tract-binding protein 1
G3H9U3_CRIGR	Splicing factor, proline-and glutamine-rich
G3HXU0_CRIGR	Tubulin--tyrosine ligase-like protein 12
G3HNV4_CRIGR	Triosephosphate isomerase
G3HFJ1_CRIGR	Scavenger mRNA-decapping enzyme DcpS
G3HG69_CRIGR	Hepatoma-derived growth factor
G3H7Y0_CRIGR	Coatomer subunit alpha
G3H609_CRIGR	Glutathione reductase, mitochondrial
G3HRD9_CRIGR	Proteasome subunit beta type
G3IJU1_CRIGR	MAM domain-containing protein 2
UGPA_CRIGR	UTP--glucose-1-phosphate uridylyltransferase
G3HDR3_CRIGR	T-complex protein 1 subunit delta
G3GSZ1_CRIGR	Procollagen C-endopeptidase enhancer 2
G3HKG8_CRIGR	40S ribosomal protein S3a
G3HZ55_CRIGR	Integrin beta
G3H2E2_CRIGR	Heterogeneous nuclear ribonucleoprotein A/B
G3HJS0_CRIGR	Spectrin beta chain, brain 1
G3IB86_CRIGR	POM121-like protein 2
G3GRS9_CRIGR	N-acetylgalactosamine-6-sulfatase
G3I505_CRIGR	Eukaryotic translation initiation factor 3 subunit L
G3H0V1_CRIGR	Microtubule-associated protein 1A
G3H3W4_CRIGR	Insulin-degrading enzyme
G3I5H1_CRIGR	Glutathione S-transferase A4
G3H5D5_CRIGR	Acyl-CoA-binding protein
G3HSZ6_CRIGR	Aldose 1-epimerase
G3HC39_CRIGR	Farnesyl pyrophosphate synthetase
G3H9V0_CRIGR	Proteasome subunit beta type
G3IMZ5_CRIGR	Myosin-6
G3HE84_CRIGR	Microtubule-associated protein RP/EB family member 1
HPRT_CRIGR	Hypoxanthine-guanine phosphoribosyltransferase
G3GXN3_CRIGR	Intersectin-2
G3INU6_CRIGR	UDP-glucose 6-dehydrogenase
G3H873_CRIGR	ELKS/RAB6-interacting/CAST family member 1
G3H577_CRIGR	Ganglioside GM2 activator
A4URF0_CRIGR	C-X-C motif chemokine 3
G3I0I7_CRIGR	S-methyl-5'-thioadenosine phosphorylase
G3HC91_CRIGR	Vascular endothelial growth factor A
G3IHC7_CRIGR	Protein arginine N-methyltransferase 1
G3IC99_CRIGR	Septin-11
G3HTJ2_CRIGR	Septin-7 (Fragment)
G3HNT9_CRIGR	Proteasome subunit beta type-1
G3HW54_CRIGR	Protein OS-9
FRIH_CRIGR	Ferritin heavy chain
G3HJS1_CRIGR	Spectrin beta chain, brain 1
G3I729_CRIGR	Myosin-14
G3IBF7_CRIGR	Ubiquitin-conjugating enzyme E2 K
G3IDE4_CRIGR	Tripeptidyl-peptidase 1
G3HMU4_CRIGR	Chloride intracellular channel protein 4
G3H303_CRIGR	Proteasome subunit beta type
G3HI85_CRIGR	Acetyl-CoA carboxylase 2
G3I8H3_CRIGR	Vacuolar protein sorting-associated protein 35
G3HNK4_CRIGR	Dihydropyrimidinase-related protein 2
G3HPQ5_CRIGR	Hepatocyte growth factor
G3HU51_CRIGR	Isocitrate dehydrogenase [NADP]
MGT5A_CRIGR	Alpha-1,6-mannosylglycoprotein 6-beta-N-acetylglucosaminyltransferase A
G3HTA7_CRIGR	Lupus brain antigen 1 (Fragment)
G3I0S4_CRIGR	Protocadherin-7
G3GV64_CRIGR	Mammalian ependymin-related protein 1
G3HH02_CRIGR	Uncharacterized protein C20orf135-like
G3ILS7_CRIGR	26S proteasome non-ATPase regulatory subunit 5
G3H331_CRIGR	Ras GTPase-activating-like protein IQGAP2
G3H4S3_CRIGR	Heat shock 70 kDa protein 13
G3HEQ3_CRIGR	Ran-specific GTPase-activating protein
G3I863_CRIGR	Ephrin type-A receptor 2
G3H8X9_CRIGR	80 kDa MCM3-associated protein
G3H4V1_CRIGR	Septin-2
G3GXW9_CRIGR	SAM domain and HD domain-containing protein 1

G3HN89_CRIGR	Palmitoyl-protein thioesterase 1
G3H278_CRIGR	60S ribosomal protein L6
G3H6C5_CRIGR	Hsp90 co-chaperone Cdc37
G3H1K6_CRIGR	Disintegrin and metalloproteinase domain-containing protein 9
G3HWJ3_CRIGR	Putative uncharacterized protein
G3IHR6_CRIGR	Macrophage colony-stimulating factor 1
G3IBK9_CRIGR	Rab GDP dissociation inhibitor alpha
G3I3J3_CRIGR	A disintegrin and metalloproteinase with thrombospondin motifs 1
G3HIM4_CRIGR	Cell division control protein 42-like
G3I2F2_CRIGR	Splicing factor 3B subunit 2
G3I0G1_CRIGR	U3 small nucleolar RNA-associated protein 15-like
G3H6Z4_CRIGR	Phosphorylase
G3H8W5_CRIGR	6-phosphofructokinase
G3I5A4_CRIGR	Annexin
G3HCI6_CRIGR	Receptor-type tyrosine-protein phosphatase S
G3GXT2_CRIGR	CAD protein
G3HCH0_CRIGR	UPF0556 protein C19orf10-like
G3IBJ1_CRIGR	Dynamin-1
HMCS1_CRIGR	Hydroxymethylglutaryl-CoA synthase, cytoplasmic
G3H037_CRIGR	U5 small nuclear ribonucleoprotein 200 kDa helicase
Q91Z81_CRIGR	Protein disulfide-isomerase
G3HGL6_CRIGR	Vascular endothelial growth factor C
G3IDS2_CRIGR	F-actin-capping protein subunit alpha-1
G3HJJ1_CRIGR	Ribose-5-phosphate isomerase
G3H6T5_CRIGR	Hexokinase-1
G3I142_CRIGR	Splicing factor 3 subunit 1
G3I782_CRIGR	Heterogeneous nuclear ribonucleoprotein D0
G3HAQ2_CRIGR	Eukaryotic translation initiation factor 6
G3IBN9_CRIGR	Keratin, type II cytoskeletal 71
G3HV18_CRIGR	60S ribosomal protein L12
G3I3C7_CRIGR	Putative allantoicase
G3HIQ1_CRIGR	Peptidyl-prolyl cis-trans isomerase
G3IKX2_CRIGR	Pigment epithelium-derived factor
G3HN65_CRIGR	Ras suppressor protein 1
G3IFE7_CRIGR	Phosphoglucomutase-2
G3HTI5_CRIGR	Tumor necrosis factor receptor superfamily member 21
G3HRJ1_CRIGR	FACT complex subunit SSRP1
G3HIX6_CRIGR	Tryptophanyl-tRNA synthetase, cytoplasmic
G3GUP7_CRIGR	Ubiquitin carboxyl-terminal hydrolase
G3HZ28_CRIGR	Twisted gastrulation protein-like 1
G3HJB1_CRIGR	Alpha-actinin-4
G3I3F3_CRIGR	Ribonuclease inhibitor
G3H4D2_CRIGR	Glycosyltransferase 25 family member 1
G3I0C6_CRIGR	Cullin-5
G3I9G6_CRIGR	AT-rich interactive domain-containing protein 4A
G3HRX4_CRIGR	Disintegrin and metalloproteinase domain-containing protein 22
G3HWP7_CRIGR	FACT complex subunit SPT16
G3HQ74_CRIGR	Gamma-glutamyl hydrolase
G3GXQ2_CRIGR	Protein FAM59B
G3I2B6_CRIGR	Serine/threonine-protein phosphatase 2A 65 kDa regulatory subunit A alpha isoform (Fragment)
G3I737_CRIGR	40S ribosomal protein S12
G3IHM2_CRIGR	Atrial natriuretic factor
G3H6E1_CRIGR	Eukaryotic translation initiation factor 3 subunit G
G3I621_CRIGR	Desmin
G3I7Z8_CRIGR	Importin-7
G3I310_CRIGR	26S proteasome non-ATPase regulatory subunit 2
G3HI29_CRIGR	Ceroid-lipofuscinosis neuronal protein 5
Q60444_CRIGR	Type VII collagen (Fragment)
G3I3W4_CRIGR	S-formylglutathione hydrolase
G3H780_CRIGR	Splicing factor, arginine/serine-rich 17B
G3HZ17_CRIGR	Protein phosphatase 1 regulatory subunit 12
G3HS88_CRIGR	Ubiquitin-conjugating enzyme E2 N (Fragment)
G3GZU7_CRIGR	Tolloid-like protein 2
G3GW53_CRIGR	Epithelial cell transforming sequence 2 oncogene-like
G3HJB0_CRIGR	Alpha-actinin-4 (Fragment)
G3HVE1_CRIGR	Putative ATP-dependent RNA helicase YTHDC2
G3GTN0_CRIGR	Low-density lipoprotein receptor-related protein 8
G3H3I7_CRIGR	Neuron navigator 1
G3IIK9_CRIGR	Cornifin-A
G3H945_CRIGR	Eukaryotic translation initiation factor 3 subunit I
G3HF38_CRIGR	Basigin
G3IKI6_CRIGR	Keratin, type II cytoskeletal 1b
G3GUR3_CRIGR	Complement C1r subcomponent-like protein
G3IH46_CRIGR	Prothymosin alpha

G3GTA4_CRIGR	A disintegrin and metalloproteinase with thrombospondin motifs 7
G3HLV6_CRIGR	ATP-citrate synthase
G3GYB0_CRIGR	Proline-, glutamic acid-and leucine-rich protein 1
G3GZC9_CRIGR	VWFA and cache domain-containing protein 1
G3I6B7_CRIGR	Teneurin-4
G3GSM9_CRIGR	Leishmanolysin-like peptidase (Fragment)
G3H9I9_CRIGR	Hepatocyte growth factor receptor
G3II33_CRIGR	Heterogeneous nuclear ribonucleoprotein U-like protein 2
G3HTK4_CRIGR	Neuron navigator 3
G3IJD1_CRIGR	Putative ATP-dependent RNA helicase DDX17
G3I5S7_CRIGR	Coatomer subunit delta
G3HSE5_CRIGR	Coatomer subunit beta
G3I216_CRIGR	Triosephosphate isomerase
G3H0Y9_CRIGR	EH domain-containing protein 4
G3I2V6_CRIGR	Plexin-B2
G3GVY7_CRIGR	Nardilysin
G3HA23_CRIGR	Malate dehydrogenase (Fragment)
G3H750_CRIGR	Calpastatin
G3HU31_CRIGR	Neuroblastoma suppressor of tumorigenicity 1
G3H5N5_CRIGR	UDP-glucuronic acid decarboxylase 1
G3H1B3_CRIGR	Lamin-L(I)
G3I9P1_CRIGR	LDLR chaperone MESD
G3HP24_CRIGR	Isoleucyl-tRNA synthetase, cytoplasmic
G3H319_CRIGR	Tubulin-specific chaperone A
G3IKS9_CRIGR	Glutaredoxin-3
G3HGV7_CRIGR	tRNA-splicing ligase RtcB homolog
G3HD57_CRIGR	Syntenin-1
G3H2K2_CRIGR	Cytochrome c, somatic
G3IEP6_CRIGR	Laminin subunit alpha-2
G3HHZ9_CRIGR	Delta-aminolevulinic acid dehydratase
G3I7J6_CRIGR	Non-POU domain-containing octamer-binding protein
G3HID3_CRIGR	Coatomer subunit gamma
G3HIC5_CRIGR	RuvB-like 1
G3H4V0_CRIGR	Vigilin
G3I136_CRIGR	Leukemia inhibitory factor
G3HNA5_CRIGR	Ubiquitin carboxyl-terminal hydrolase isozyme L3 (Fragment)
G3HWH0_CRIGR	Zinc finger protein 142
G3ICC2_CRIGR	BAG family molecular chaperone regulator 3
G3HRX5_CRIGR	Protein DBF4-like A
G3ILF1_CRIGR	Glutathione S-transferase Mu 5
G3I3Z5_CRIGR	Coronin
G3GX59_CRIGR	Putative sodium-coupled neutral amino acid transporter 10
G3HQ69_CRIGR	Src substrate cortactin
G3HBJ9_CRIGR	Methionine aminopeptidase 2
G3I1P6_CRIGR	Nucleosome-binding protein 1
G3GXZ0_CRIGR	Protein-glutamine gamma-glutamyltransferase 2
G3HE67_CRIGR	Protein CREG1
G3GTH2_CRIGR	Na(+)/H(+) exchange regulatory cofactor NHE-RF1
G3HUL2_CRIGR	Aldehyde oxidase
G3GZB1_CRIGR	Eukaryotic translation initiation factor 3 subunit M
G3I9F1_CRIGR	Ras GTPase-activating protein-binding protein 2
G3HXV5_CRIGR	Nucleolar phosphoprotein p130
G3HR96_CRIGR	Bleomycin hydrolase
G3HXL1_CRIGR	Poly(RC)-binding protein 1
G3H5W0_CRIGR	Proteasome subunit beta type
G3HCK9_CRIGR	Far upstream element-binding protein 2
G3HC01_CRIGR	Tropomyosin alpha-3 chain
G3GVX2_CRIGR	Destrin
G3I7K9_CRIGR	Collagen alpha-1(IV) chain
G3IH35_CRIGR	Plasminogen activator inhibitor 1 RNA-binding protein
G3GSW3_CRIGR	Coatomer subunit beta'
G3I306_CRIGR	Eukaryotic translation initiation factor 4 gamma 1
G3HSL4_CRIGR	Elongation factor 2
G3HHR3_CRIGR	Vimentin
G3IGW0_CRIGR	Protein CTLA-2-beta
G3I151_CRIGR	Transcobalamin-2
G3GY95_CRIGR	Eukaryotic translation initiation factor 5A-1
G3HI96_CRIGR	Coronin
G3HVJ5_CRIGR	Squamous cell carcinoma antigen recognized by T-cells 3
G3IF83_CRIGR	A-kinase anchor protein SPHKAP
G3HWP9_CRIGR	Heterogeneous nuclear ribonucleoproteins C1/C2
G3HQT0_CRIGR	Proteasome subunit beta type
G3HTG9_CRIGR	Glyoxalase domain-containing protein 4
G3IGI9_CRIGR	STE20-like serine/threonine-protein kinase

G3HAG8_CRIGR	AP-1 complex subunit gamma-1
G3IIT6_CRIGR	Cysteinyl-tRNA synthetase, cytoplasmic
G3HX39_CRIGR	Phosphoglycerate kinase
G3HDD4_CRIGR	NEDD8
G3I2F5_CRIGR	Barrier-to-autointegration factor
G3GY75_CRIGR	Eukaryotic initiation factor 4A-I
G3HPT5_CRIGR	Glyceraldehyde-3-phosphate dehydrogenase
G3HT18_CRIGR	Nucleosome assembly protein 1-like 1
G3H2X6_CRIGR	Histone-lysine N-methyltransferase SETDB1
G3HPF1_CRIGR	Small nuclear ribonucleoprotein Sm D2
G3HTF5_CRIGR	Phosphatidylinositol transfer protein alpha isoform
G3H3I9_CRIGR	Cysteine and glycine-rich protein 1
G3GWP1_CRIGR	Protein transport protein Sec24C
G3HUM5_CRIGR	SUMO-activating enzyme subunit 2
G3I5Q4_CRIGR	Heterogeneous nuclear ribonucleoprotein M
G3HHG3_CRIGR	Complement component C7
AIMP1_CRIGR	Aminoacyl tRNA synthase complex-interacting multifunctional protein 1
G3ID62_CRIGR	Exostosin-like 2
G3HX58_CRIGR	Leucine-rich repeat transmembrane protein FLRT3
G3IG18_CRIGR	Alpha-mannosidase 2x
G3GZR1_CRIGR	Aspartyl-tRNA synthetase, cytoplasmic
G3GXU1_CRIGR	Protein phosphatase 1G
G3I4I9_CRIGR	Major vault protein
G3I620_CRIGR	Aspartyl aminopeptidase
G3GTC7_CRIGR	Malic enzyme (Fragment)
G3HNW2_CRIGR	Cadherin-9
G3HSA2_CRIGR	Myb-binding protein 1A
G3GZE6_CRIGR	Actin-related protein 3
G3HIS0_CRIGR	26S proteasome non-ATPase regulatory subunit 6
G3HD51_CRIGR	LisH domain and HEAT repeat-containing protein KIAA1468
G3HSM8_CRIGR	Lamin-B2
G3I6C1_CRIGR	Integrin alpha-5
G3HRR5_CRIGR	ELAV-like protein 1
G3I2I9_CRIGR	60S ribosomal protein L3
G3IDR6_CRIGR	Tumor necrosis factor-inducible gene 6 protein
G3HH63_CRIGR	Phospholipase A-2-activating protein
G3HEZ1_CRIGR	Eukaryotic peptide chain release factor subunit 1
G3H6M5_CRIGR	Fumarate hydratase, mitochondrial
G3IBB9_CRIGR	Nodal modulator 1 (Fragment)
G3HLP2_CRIGR	CAP-Gly domain-containing linker protein 2
G3IJ63_CRIGR	Protein FAM178A
G3HGU9_CRIGR	Oncostatin-M specific receptor subunit beta
G3IB32_CRIGR	Twinfilin-1
G3I1R6_CRIGR	Epididymal secretory protein E3-beta
G3H217_CRIGR	Pentraxin-related protein PTX3
G3HES3_CRIGR	Exportin-2
G3HFQ8_CRIGR	Acylphosphatase
G3I5R2_CRIGR	40S ribosomal protein S28
G3IJK4_CRIGR	Interferon-induced protein with tetratricopeptide repeats 3
MBTP1_CRIGR	Membrane-bound transcription factor site-1 protease
G3I6Y6_CRIGR	Sulfhydryl oxidase
G3HMN4_CRIGR	Omega-amidase NIT2
G3I798_CRIGR	AMP deaminase 2
G3HML5_CRIGR	Calcium-dependent secretion activator 2 (Fragment)
G3I0Q0_CRIGR	Non-muscle caldesmon
G3GRZ4_CRIGR	Plexin-A1
G3I6H5_CRIGR	Fermitin family-like 2
G3I9D3_CRIGR	Proteasome subunit beta type
G3HLN4_CRIGR	Peptidyl-prolyl cis-trans isomerase
G3H6Y5_CRIGR	Peptidyl-prolyl cis-trans isomerase
G3H576_CRIGR	Annexin
G3H8Y5_CRIGR	Collagen alpha-1(VI) chain
G3GYL9_CRIGR	Formin-binding protein 4
G3GTC6_CRIGR	Inactive serine protease 35
G3I4P0_CRIGR	Craniofacial development protein 1
G3IDD4_CRIGR	Serpin H1
G3I2N4_CRIGR	Lamina-associated polypeptide 2, isoforms alpha/zeta
G3H2N6_CRIGR	Programmed cell death 6-interacting protein
G3I2Y1_CRIGR	Aspartate aminotransferase
G3HTF8_CRIGR	Proto-oncogene C-crk
G3GYZ1_CRIGR	CD44 antigen
G3HM86_CRIGR	Breast cancer type 2 susceptibility protein-like
G3HCU8_CRIGR	26S protease regulatory subunit 8
G3IH34_CRIGR	Apoptosis inhibitor 5

G3H354_CRIGR	Heat shock protein HSP 90-alpha
G3H0T1_CRIGR	Eukaryotic translation initiation factor 3 subunit J
G3ICW1_CRIGR	Osteoclast-stimulating factor 1
G3H277_CRIGR	Tyrosine-protein phosphatase non-receptor type 11
G3HCI9_CRIGR	Scaffold attachment factor B1
G3HRP2_CRIGR	Protein canopy-like 2
G3GYC6_CRIGR	Profilin-1
G3GX96_CRIGR	Actin-related protein 2/3 complex subunit 4
G3HY08_CRIGR	Macrophage migration inhibitory factor
G3IE68_CRIGR	Laminin subunit alpha-2
G3ICL7_CRIGR	Voltage-gated potassium channel subunit beta-2
G3HR10_CRIGR	Dihydropteridine reductase
G3GSH5_CRIGR	ATP-dependent RNA helicase DDX3X
G3HCV0_CRIGR	ATP-dependent RNA helicase DDX42
G3HW48_CRIGR	Dynactin subunit 2
G3H8F9_CRIGR	Transforming protein RhoA
G3IKQ6_CRIGR	Cystatin-B
G3HS35_CRIGR	Beta-type platelet-derived growth factor receptor
G3IMZ0_CRIGR	Very low-density lipoprotein receptor
G3I2D3_CRIGR	40S ribosomal protein S20
G3I1H0_CRIGR	DNA replication licensing factor MCM3
G3HLV1_CRIGR	FK506-binding protein 10
G3HKP7_CRIGR	Protein shisa-5
G3IA12_CRIGR	Transforming growth factor beta-1
G3HXT3_CRIGR	Protein kinase C and casein kinase substrate in neurons protein 2
G3HQY6_CRIGR	Lipase
G3HCI0_CRIGR	Perilipin
G3IB71_CRIGR	Glutaminyl-tRNA synthetase
G3ILT7_CRIGR	Golgi membrane protein 1
G3HZV0_CRIGR	Ribosomal protein
Q99PC2_CRIGR	Beta-1,4-galactosyltransferase 1
G3II91_CRIGR	Voltage-dependent N-type calcium channel subunit alpha-1B
G3GRB9_CRIGR	Melanoma inhibitory activity protein 3
G3HCX8_CRIGR	Calreticulin
G3I2D8_CRIGR	Band 4.1-like protein 2
HMGA1_CRIGR	RecName: Full
G3INL9_CRIGR	CMP-N-acetylneuraminate-beta-galactosamide-alpha-2, 3-sialyltransferase
G3HAN5_CRIGR	Eukaryotic translation initiation factor 2 subunit 2
G3I4K1_CRIGR	Alpha-actinin-3
G3IJU2_CRIGR	Rho GTPase-activating protein 18
G3HM30_CRIGR	Zinc finger protein 622
G3GW11_CRIGR	Non-specific lipid-transfer protein
G3I6W5_CRIGR	Prefoldin subunit 6
G3GYP7_CRIGR	Alcohol dehydrogenase [NADP+]
G3IIT5_CRIGR	Nucleosome assembly protein 1-like 4
G3H3S4_CRIGR	Protein-L-isoaspartate(D-aspartate) O-methyltransferase
G3H6D2_CRIGR	Intercellular adhesion molecule 5
GRP75_CRIGR	Stress-70 protein, mitochondrial
G3HWS5_CRIGR	Putative leucyl-tRNA synthetase, mitochondrial
G3H1W0_CRIGR	Brain-specific angiogenesis inhibitor 2
G3GZG1_CRIGR	Toll-like receptor 9 (Fragment)
G3HYW3_CRIGR	Cullin-4B (Fragment)
G3IIR3_CRIGR	Transforming acidic coiled-coil-containing protein 2
G3H3I2_CRIGR	Importin-9
G3IAY3_CRIGR	SEC23-interacting protein
G3HLD0_CRIGR	Lactoylglutathione lyase
Q9R119_CRIGR	Urokinase plasminogen activator surface receptor (Fragment)
G3H958_CRIGR	A disintegrin and metalloproteinase with thrombospondin motifs 18
G3I3I0_CRIGR	AP-2 complex subunit alpha-2
G3H3H9_CRIGR	Aminopeptidase B
G3HRL6_CRIGR	CD63 antigen
G3IHE5_CRIGR	GTP-binding nuclear protein Ran
G3IMX9_CRIGR	von Willebrand factor A domain-containing protein 5A
G3GUY5_CRIGR	Protein KIAA1967-like
G3IL48_CRIGR	Protein transport protein Sec31A
G3I7Y5_CRIGR	Kinesin heavy chain isoform 5C
G3HX69_CRIGR	LIM domain and actin-binding protein 1
G3IPK9_CRIGR	Adenylate kinase 2, mitochondrial
G3I968_CRIGR	Histone H2A
G3H9B2_CRIGR	Myoferlin
G3H4Z8_CRIGR	Heat shock-related 70 kDa protein 2
G3IA94_CRIGR	Myosin regulatory light chain 12B
G3IDD9_CRIGR	40S ribosomal protein S3
G3GUC8_CRIGR	Obg-like ATPase 1

G3I413_CRIGR	Beta-1,3-galactosyltransferase 6
G3IJ29_CRIGR	Eukaryotic translation initiation factor 3 subunit D
ASNS_CRIGR	Asparagine synthetase [glutamine-hydrolyzing]
G3H6B2_CRIGR	Dynamin
G3H0V2_CRIGR	Tumor suppressor p53-binding protein 1
G3IJV7_CRIGR	Cytosolic acyl coenzyme A thioester hydrolase
G3H2B7_CRIGR	Septin-8
G3HP71_CRIGR	Heterogeneous nuclear ribonucleoprotein H (Fragment)
G3IEB2_CRIGR	Extracellular matrix protein FRAS1
G3H7M8_CRIGR	Heterogeneous nuclear ribonucleoprotein U
G3HZY1_CRIGR	RuvB-like 2
G3ID08_CRIGR	Putative pre-mRNA-splicing factor ATP-dependent RNA helicase DHX15
G3HNF5_CRIGR	Papilin (Fragment)
G3IGI6_CRIGR	Cytosolic purine 5'-nucleotidase
G3HKV9_CRIGR	Group XV phospholipase A2
G3HXW9_CRIGR	Alpha-centractin
G3IIA1_CRIGR	Methylosome protein 50 (Fragment)
G3I3H9_CRIGR	Chitinase domain-containing protein 1
G3HCB6_CRIGR	Putative tumor suppressor protein MN1
G3I710_CRIGR	AP-2 complex subunit alpha-1
G3HCI7_CRIGR	Scaffold attachment factor B2
G3GU41_CRIGR	Putative RNA-binding protein 19
G3I7W3_CRIGR	Dynactin subunit 1
G3I5T3_CRIGR	Putative ATP-dependent RNA helicase DDX6
G3HLZ2_CRIGR	Proteasome activator complex subunit 3
G3HV49_CRIGR	UPF0160 protein MYG1, mitochondrial
G3GUQ0_CRIGR	Gamma-enolase
G3IBZ4_CRIGR	Phosphoglycerate kinase
G3I027_CRIGR	Heat shock protein 75 kDa, mitochondrial
G3IDU4_CRIGR	Protein-tyrosine sulfotransferase 1
G3H9U6_CRIGR	Uncharacterized protein KIAA0319-like
G3GT01_CRIGR	RING finger protein 160
G3HWY2_CRIGR	Exostosin-2
G3HHE3_CRIGR	Leucyl-tRNA synthetase, cytoplasmic
G3GYY2_CRIGR	Caprin-1
G3H0U6_CRIGR	Protein disulfide-isomerase A3
G3HPZ6_CRIGR	ELMO domain-containing protein 3
G3IHH6_CRIGR	Fumarylacetoacetase
F7J0L2_CRIGR	Splicing factor, arginine/serine-rich 1 (Fragment)
G3I366_CRIGR	Prolyl endopeptidase
G3H2C9_CRIGR	S-phase kinase-associated protein 1
G3IGS8_CRIGR	Serrate RNA effector molecule-like
G3I7U9_CRIGR	Serine protease HTRA2, mitochondrial
G3IAX3_CRIGR	Pyruvate kinase
G3H7Y5_CRIGR	Sodium/potassium-transporting ATPase subunit alpha-2
G3H853_CRIGR	cAMP-dependent protein kinase type I-alpha regulatory subunit
G3GXW5_CRIGR	Protein NDRG3
G3H3D3_CRIGR	Branched-chain-amino-acid aminotransferase
G3HHE4_CRIGR	Leucyl-tRNA synthetase, cytoplasmic
G3I539_CRIGR	Hsc70-interacting protein
G3GU76_CRIGR	60S acidic ribosomal protein P0
Q8MHC1_CRIGR	MHC class I antigen Hm1-C5
G3HZE7_CRIGR	Heat shock 70 kDa protein 1L
G3H8Q4_CRIGR	Acidic leucine-rich nuclear phosphoprotein 32 family member A
G3HMM2_CRIGR	Peptidyl-prolyl cis-trans isomerase
G3HYJ8_CRIGR	10 kDa heat shock protein, mitochondrial
G3HKD6_CRIGR	Histone-binding protein RBBP7
G3HIP2_CRIGR	Heterogeneous nuclear ribonucleoprotein R
G3HCL8_CRIGR	Cdc42-interacting protein 4
G3HFY1_CRIGR	Protein phosphatase 1F
G3GYR1_CRIGR	Putative E3 ubiquitin-protein ligase HECTD3
G3H1G7_CRIGR	Proline synthetase co-transcribed bacterial-like protein
G3GZA9_CRIGR	Reticulocalbin-1 (Fragment)
G3HC31_CRIGR	Protein S100-A6
G3ILG2_CRIGR	THUMP domain-containing protein 1
G3HHV8_CRIGR	Histone deacetylase
G3IHH9_CRIGR	Isoamyl acetate-hydrolyzing esterase 1-like
Q9Z2J2_CRIGR	Apurinic/apyrimidinic endonuclease
G3HND2_CRIGR	Histone deacetylase 6
G3HTF0_CRIGR	N-sulphoglucosamine sulphohydrolase
G3I973_CRIGR	Hypoxia up-regulated protein 1
G3H4I2_CRIGR	Tropomyosin beta chain
G3HK37_CRIGR	Thrombospondin-2
G3IKI7_CRIGR	Keratin, type II cytoskeletal 2 oral

G3I3V5_CRIGR	Type 2 lactosamine alpha-2,3-sialyltransferase
G3GSZ0_CRIGR	Plastin-1
G3HX84_CRIGR	Gelsolin
G3GXN5_CRIGR	Nuclear receptor coactivator 1
G3H355_CRIGR	Envelope glycoprotein
G3GY86_CRIGR	Neuroligin-2
G3HEN3_CRIGR	Protein HIRA
G3II70_CRIGR	Bifunctional ATP-dependent dihydroxyacetone kinase/FAD-AMP lyase (Cyclizing)
G3HC17_CRIGR	Interleukin enhancer-binding factor 2
G3I757_CRIGR	ADP-ribosylation factor-like protein 3
G3HAT2_CRIGR	NMDA receptor-regulated protein 1
G3GSJ8_CRIGR	Disks large-like 1
G3IE48_CRIGR	Elongation factor 1-gamma
G3HQN3_CRIGR	H-2 class I histocompatibility antigen, D-D alpha chain
G3GYR9_CRIGR	40S ribosomal protein S8
G3H2P3_CRIGR	Beta-galactosidase (Fragment)
G3I881_CRIGR	Peptidyl-prolyl cis-trans isomerase
G3I952_CRIGR	EF-hand domain-containing protein D2
G3HX83_CRIGR	Ras-related protein Rab-14
G3HV92_CRIGR	H/ACA ribonucleoprotein complex subunit 4
G3GT78_CRIGR	Interleukin-1 receptor accessory protein
G3I198_CRIGR	Rab effector MyRIP
G3GWM0_CRIGR	Eukaryotic peptide chain release factor GTP-binding subunit ERF3B
G3HXY1_CRIGR	PDZ and LIM domain protein 1
G3HKY0_CRIGR	High mobility group protein B2
G3IJR3_CRIGR	Peripherin
G3HC41_CRIGR	Pyruvate kinase
G3HGK3_CRIGR	L-lactate dehydrogenase A chain
G3IGZ8_CRIGR	Torsin-1B
G3GV69_CRIGR	Cytochrome c
G3HYE4_CRIGR	Protocadherin gamma-C3
G3HKR3_CRIGR	Tyrosine-protein phosphatase non-receptor type 23
ACTB_CRIGR	Actin, cytoplasmic 1
G3HLL4_CRIGR	116 kDa U5 small nuclear ribonucleoprotein component
G3I4R7_CRIGR	Kallikrein-7
G3GYM1_CRIGR	Receptor-type tyrosine-protein phosphatase eta
G3H9L2_CRIGR	Ubiquitin carboxyl-terminal hydrolase
G3H2U4_CRIGR	BolA-like protein 1
G3I837_CRIGR	Mannose-6-phosphate isomerase
G3H9Q6_CRIGR	Stanniocalcin-2
G3HW36_CRIGR	Serine hydroxymethyltransferase
G3I6H3_CRIGR	Splicing factor 1
G3I4G1_CRIGR	Xanthine dehydrogenase/oxidase
G3HLR7_CRIGR	Adenosine deaminase
G3IDS7_CRIGR	Monocarboxylate transporter 1
G3HVL1_CRIGR	Cell division control protein 2-like
G3HQS8_CRIGR	Proteasome subunit beta type
H6ST1_CRIGR	Heparan-sulfate 6-O-sulfotransferase 1
G3HCS4_CRIGR	26S proteasome non-ATPase regulatory subunit 12
G3IEX5_CRIGR	Phosphopantothenate--cysteine ligase
G3HM98_CRIGR	Drebrin-like protein
G3I5T6_CRIGR	Serine/threonine-protein phosphatase 5
G3HDE5_CRIGR	Proteasome activator complex subunit 1
G3I072_CRIGR	Elongation factor 1-gamma
G3HCI4_CRIGR	Receptor-type tyrosine-protein phosphatase S
G3HFW9_CRIGR	Sorting nexin
G3HW49_CRIGR	Kinesin heavy chain isoform 5A
G3GVP7_CRIGR	ADP-sugar pyrophosphatase
G3HQS5_CRIGR	Bromodomain-containing protein 2
G3HIK1_CRIGR	Nuclear pore complex protein Nup93
G3HSM4_CRIGR	Small glutamine-rich tetratricopeptide repeat-containing protein alpha
G3I595_CRIGR	Histidyl-tRNA synthetase, cytoplasmic
G3IED5_CRIGR	Peptidyl-prolyl cis-trans isomerase A
Q8MHC4_CRIGR	MHC class I antigen Hm1-C2
G3IKD4_CRIGR	Keratin, type II cytoskeletal 79
G3GR90_CRIGR	Isopentenyl-diphosphate Delta-isomerase 1
G3IAG0_CRIGR	Xaa-Pro aminopeptidase 1
G3GTI1_CRIGR	Protein sidekick-2
G3HV83_CRIGR	Prefoldin subunit 3
G3H8H7_CRIGR	DnaJ-like subfamily C member 10
G3H283_CRIGR	Putative uncharacterized protein
G3I6P1_CRIGR	Glyceraldehyde-3-phosphate dehydrogenase
G3H9H7_CRIGR	Heterogeneous nuclear ribonucleoprotein G
G3HWJ4_CRIGR	Epiplakin

G3I2J3_CRIGR	Chromobox protein-like 6 (Fragment)
G3H1K2_CRIGR	Fibroblast growth factor receptor
G3IDJ8_CRIGR	6-phosphofructokinase
G3HBR6_CRIGR	Phosphoglycolate phosphatase
G3I319_CRIGR	AP-2 complex subunit mu-1
G3HZA8_CRIGR	Glyceraldehyde-3-phosphate dehydrogenase
G3II52_CRIGR	Oxysterol-binding protein (Fragment)
G3HV50_CRIGR	Prefoldin subunit 5
G3GXA9_CRIGR	Protein disulfide-isomerase
G3IBL4_CRIGR	Glucose-6-phosphate 1-dehydrogenase
G3H756_CRIGR	Glutaredoxin-1
G3HY23_CRIGR	Small nuclear ribonucleoprotein Sm D3
G3H381_CRIGR	Kinesin light chain 1
G3HXU8_CRIGR	Fibroblast growth factor
G3GVQ8_CRIGR	Selenide, water dikinase 1
G3HPE9_CRIGR	Echinoderm microtubule-associated protein-like 2
G3H9Z4_CRIGR	Serine-threonine kinase receptor-associated protein
Q8MHC2_CRIGR	MHC class I antigen Hm1-C4
G3HHA3_CRIGR	40S ribosomal protein S24
G3I4G0_CRIGR	EH domain-containing protein 3
G3HU18_CRIGR	A disintegrin and metalloproteinase with thrombospondin motifs 4
G3I230_CRIGR	Oligoribonuclease, mitochondrial
B4GT3_CRIGR	Beta-1,4-galactosyltransferase 3
G3HT77_CRIGR	Cytoplasmic FMR1-interacting protein 1
G3IBV7_CRIGR	Cytochrome b5
G3HXT4_CRIGR	Protein kinase C and casein kinase substrate in neurons protein 2
G3IFV3_CRIGR	Exportin-1
G3HSY7_CRIGR	V-type proton ATPase subunit H
G3HVH8_CRIGR	60S ribosomal protein L5
G3INF7_CRIGR	Glutathione S-transferase Mu 1
G3HID1_CRIGR	Ras-related protein Rab-7a
G3HXZ3_CRIGR	CD81 antigen
G3ID68_CRIGR	Selenoprotein P
G3GVR5_CRIGR	Cerebral dopamine neurotrophic factor
G3I015_CRIGR	NAD(P)H dehydrogenase [quinone] 1
G3GXB1_CRIGR	THO complex subunit 4
G3IPI0_CRIGR	Zinc finger protein 180
G3H6D0_CRIGR	Ribonucleoprotein PTB-binding 1
G3HCB0_CRIGR	PC4 and SFRS1-interacting protein
G3INA2_CRIGR	Tubulin alpha chain, nucleomorph
G3HRQ4_CRIGR	Myosin light polypeptide 6
G3I180_CRIGR	TRAF family member-associated NF-kappa-B activator
G3HQR5_CRIGR	H-2 class I histocompatibility antigen, D-37 alpha chain
G3IQ06_CRIGR	Pigment epithelium-derived factor
G3H2G5_CRIGR	Serine/threonine-protein phosphatase 2A 65 kDa regulatory subunit A beta isoform
G3I3X6_CRIGR	Proteasome subunit alpha type-6
G3HMI0_CRIGR	Semaphorin-3C
G3IAE3_CRIGR	UPF0568 protein C14orf166-like
G3I683_CRIGR	Plasminogen activator inhibitor 1 RNA-binding protein
G3HQL4_CRIGR	Carbohydrate sulfotransferase 11
G3GT95_CRIGR	Mannan-binding lectin serine protease 1 (Fragment)
G3HHL3_CRIGR	Eukaryotic translation initiation factor 5B
G3GVW1_CRIGR	Protein transport protein Sec23B
G3H204_CRIGR	Syntaxin-12
G3GRV0_CRIGR	Kelch domain-containing protein 4
G3HUD4_CRIGR	Proteasome activator complex subunit 1
G3HD18_CRIGR	ATP-dependent RNA helicase DDX39
G3I783_CRIGR	Heterogeneous nuclear ribonucleoprotein D-like
G3HR63_CRIGR	Protein canopy-like 3
G3INY3_CRIGR	Granulocyte colony-stimulating factor
G3HET8_CRIGR	Ubiquitin-conjugating enzyme E2 variant 1
G3IL75_CRIGR	Collagen alpha-1(V) chain
G3HMV2_CRIGR	60S ribosomal protein L11
G3HC59_CRIGR	Disintegrin and metalloproteinase domain-containing protein 15
G3GRX4_CRIGR	Coactosin-like protein
G3HU02_CRIGR	Olfactomedin-like protein 2B
G3H170_CRIGR	Drebrin
G3I1J6_CRIGR	[Protein ADP-ribosylarginine] hydrolase
G3GTC9_CRIGR	Phosphoacetylglucosamine mutase
G3I1Z7_CRIGR	Developmentally-regulated GTP-binding protein 1
G3GYG0_CRIGR	Vitamin K-dependent protein S
G3ID81_CRIGR	Beta-actin-like protein 2
G3HNJ6_CRIGR	60S ribosomal protein L5
G3I9Z3_CRIGR	Ribose-phosphate pyrophosphokinase 2

G3HYG2_CRIGR	Eukaryotic initiation factor 4A-II
G3HA24_CRIGR	Heat shock protein beta-1 (Fragment)
G3H5A6_CRIGR	Ferritin
G3I7T9_CRIGR	Guanine nucleotide-binding protein subunit gamma
G3H605_CRIGR	Heterogeneous nuclear ribonucleoprotein H2
G3H8Z9_CRIGR	Sodium/potassium-transporting ATPase subunit alpha-1
G3GVW2_CRIGR	Putative hydrolase RBBP9
G3I2Q7_CRIGR	Transcription intermediary factor 1-beta
G3HXT1_CRIGR	ADP-ribosylation factor GTPase-activating protein 3
G3H3B2_CRIGR	Cysteine-rich protein 2
G3H337_CRIGR	tRNA-nucleotidyltransferase 1, mitochondrial
G3I638_CRIGR	Eukaryotic translation initiation factor 3 subunit F
G3HUV6_CRIGR	Cysteine-rich motor neuron 1 protein
G3I6F9_CRIGR	EH domain-containing protein 1
G3H771_CRIGR	Septin-6
G3IN87_CRIGR	Endoplasmic reticulum mannosyl-oligosaccharide 1,2-alpha-mannosidase
G3GR86_CRIGR	6-phosphofructokinase
G3HLJ7_CRIGR	Ataxin-7-like protein 3
G3HPP2_CRIGR	4-trimethylaminobutyraldehyde dehydrogenase
G3I7E2_CRIGR	Focal adhesion kinase 1
G3GUG7_CRIGR	Protein DEK
G3H8N1_CRIGR	Plastin-2
G3I7L9_CRIGR	Eukaryotic translation initiation factor 3 subunit C
G3IDS3_CRIGR	Putative helicase MOV-10
G3HBP3_CRIGR	Hydroxyacylglutathione hydrolase, mitochondrial
G3H3S2_CRIGR	NKG2D ligand 4
G3GW88_CRIGR	Serine/threonine-protein phosphatase
G3IEE6_CRIGR	Spermidine synthase
G3HTV1_CRIGR	Protein unc-13-like D
G3I531_CRIGR	Ran GTPase-activating protein 1
G3I4E6_CRIGR	Inositol monophosphatase
G3H953_CRIGR	KH domain-containing, RNA-binding, signal transduction-associated protein 1
G3I004_CRIGR	40S ribosomal protein S11
G3HC56_CRIGR	Ephrin-A1
G3HAP1_CRIGR	Microtubule-associated proteins 1A/1B light chain 3A
G3HU10_CRIGR	Prefoldin subunit 2
G3H5I9_CRIGR	Pleiotropic regulator 1
G3HTE6_CRIGR	Eukaryotic initiation factor 4A-III
G3HWE9_CRIGR	Tubulin alpha-4A chain
G3H8D0_CRIGR	Guanine nucleotide-binding protein G(I), alpha-2 subunit
G3H7F8_CRIGR	Acidic leucine-rich nuclear phosphoprotein 32 family member B (Fragment)
G3HKX2_CRIGR	Nuclear transport factor 2
G3I5E0_CRIGR	Methylosome subunit pICln
G3HN77_CRIGR	Polyadenylate-binding protein 4
G3IFY1_CRIGR	Thymidylate synthase
Q6E6J8_CRIGR	Heterochromatin protein 1 gamma
G3HT06_CRIGR	L-lactate dehydrogenase A chain
G3GY52_CRIGR	Phosphoribosylformylglycinamidine synthase
G3I6E6_CRIGR	Actin, cytoplasmic 2
G3HVX7_CRIGR	Coiled-coil domain-containing protein 126
G3H937_CRIGR	Histone-binding protein RBBP4
G3I075_CRIGR	L-asparaginase
G3HEV1_CRIGR	Protein FAM98A
G3GSJ5_CRIGR	Arf-GAP with coiled-coil, ANK repeat and PH domain-containing protein 2
G3GXD5_CRIGR	COP9 signalosome complex subunit 1
G3H0N9_CRIGR	DnaJ-like subfamily B member 9
RS13_CRIGR	40S ribosomal protein S13
G3IA72_CRIGR	Profilin-2
G3GXX3_CRIGR	Dolichyl-diphosphooligosaccharide--protein glycosyltransferase subunit 2
G3HG54_CRIGR	Kin of IRRE-like protein 1
G3HQY2_CRIGR	Actin, aortic smooth muscle
G3I6J4_CRIGR	ADP-ribosylation factor 3
G3HHB8_CRIGR	Histone H3.3
G3IFL9_CRIGR	H-2 class I histocompatibility antigen, K-W28 alpha chain
G3I3D5_CRIGR	SH3 domain-binding glutamic acid-rich-like protein
G3HS15_CRIGR	Heat shock protein HSP 90-alpha
C4PFX6_CRIGR	Caspase 3
G3IJB6_CRIGR	Protein RCC2
G3H9Z3_CRIGR	Putative deoxyribose-phosphate aldolase
G3H194_CRIGR	Clathrin light chain B (Fragment)
G3HBY2_CRIGR	Hematological and neurological expressed 1-like protein
G3GRQ1_CRIGR	Tubulin beta-3 chain
G3IH98_CRIGR	Keratin, type II cytoskeletal 8
G3H565_CRIGR	Prolyl 4-hydroxylase subunit alpha-2

G3IBN8_CRIGR	Keratin, type II cytoskeletal 6A
G3HKA5_CRIGR	Sorting nexin-2
G3HQV2_CRIGR	Serine/threonine-protein kinase OSR1
G3H0L5_CRIGR	Tyrosine-protein kinase BLK
G3HBJ4_CRIGR	Netrin-4 (Fragment)
G3HWB6_CRIGR	NACHT, LRR and PYD domains-containing protein 9A
G3IE45_CRIGR	Enoyl-CoA hydratase domain-containing protein 1
G3GVF0_CRIGR	Beta-1,3-N-acetylglucosaminyltransferase lunatic fringe
COPE_CRIGR	Coatomer subunit epsilon
G6PD_CRIGR	Glucose-6-phosphate 1-dehydrogenase
G3ILF3_CRIGR	Glutathione S-transferase Mu 7
FBLN4_CRIGR	EGF-containing fibulin-like extracellular matrix protein 2
LAMP2_CRIGR	Lysosome-associated membrane glycoprotein 2
Q7M0H9_CRIGR	Ca2+/calmodulin-dependent protein kinase ERK1 (Fragment)
G3H511_CRIGR	Tropomyosin alpha-1 chain
PRIO_CRIGR	Major prion protein
G3HMI5_CRIGR	Di-N-acetylchitobiase
G3HW44_CRIGR	Methionyl-tRNA synthetase, cytoplasmic
G3H4H6_CRIGR	AP-1 complex subunit mu-1
G3HXA2_CRIGR	Basic leucine zipper and W2 domain-containing protein 2
G3GWE6_CRIGR	Cell growth-regulating nucleolar protein
G3HCT4_CRIGR	Putative ATP-dependent RNA helicase DDX5
G3HF76_CRIGR	DAZ-associated protein 1
G3IHQ2_CRIGR	H-2 class I histocompatibility antigen, L-D alpha chain
G3H8G0_CRIGR	Glutathione peroxidase
G3HSB7_CRIGR	Nuclear migration protein nudC
G3H9G4_CRIGR	ATP-binding cassette sub-family E member 1
G3HQY3_CRIGR	Tumor necrosis factor receptor superfamily member 6
G3HRN0_CRIGR	ATP synthase subunit beta
G3HXK8_CRIGR	Prenylcysteine oxidase
G3H5U1_CRIGR	Pyridoxine-5'-phosphate oxidase
TBB5_CRIGR	Tubulin beta-5 chain
G3I3X4_CRIGR	Fructose-bisphosphate aldolase
G3IIZ5_CRIGR	Tubulin alpha chain
G3I1D1_CRIGR	Guanine nucleotide-binding protein subunit beta-4
G3IC91_CRIGR	von Willebrand factor A domain-containing protein 5A
G3HWJ2_CRIGR	Plectin-1
G3HVP7_CRIGR	NHL repeat-containing protein 3
G3I7E1_CRIGR	Contactin-1
G3H9V4_CRIGR	Protein argonaute-4 (Fragment)
G3IG48_CRIGR	Torsin family protein C9orf167-like
G3IBP3_CRIGR	Keratin, type II cytoskeletal 2 epidermal
G3IHW0_CRIGR	Glyceraldehyde-3-phosphate dehydrogenase
G3IM13_CRIGR	Calmodulin
G3I2G7_CRIGR	EGF-containing fibulin-like extracellular matrix protein 2
G3H5W4_CRIGR	60S ribosomal protein L23
G3I7D8_CRIGR	Apoptosis-associated speck-like protein containing a CARD
G3GZ89_CRIGR	Inosine-5'-monophosphate dehydrogenase
G3HSA7_CRIGR	Thioredoxin domain-containing protein 17
G3HLX3_CRIGR	Alpha-N-acetylglucosaminidase
G3H6U1_CRIGR	Nucleolar RNA helicase 2
G3HBP8_CRIGR	40S ribosomal protein S2
G3GZ81_CRIGR	ADP-ribosylation factor 5
G3H740_CRIGR	Glyceraldehyde-3-phosphate dehydrogenase
G3H201_CRIGR	Replication protein A 32 kDa subunit
G3HDH2_CRIGR	Poly(RC)-binding protein 2
G3HYS9_CRIGR	Carbohydrate sulfotransferase 14
G3HP08_CRIGR	AP-1 complex subunit beta-1
G3IIY3_CRIGR	Beta-arrestin-1
G3HL33_CRIGR	Zyxin
G3HUQ9_CRIGR	Dentin matrix protein 4
G3HZW3_CRIGR	60S ribosomal protein L18
G3H6K1_CRIGR	Heterogeneous nuclear ribonucleoprotein G
G3HBG8_CRIGR	Calmodulin
G3GZF2_CRIGR	Peptidyl-prolyl cis-trans isomerase A
G3H601_CRIGR	Mitochondrial import inner membrane translocase subunit Tim8 A
G3I4J7_CRIGR	Leukosialin
G3GYB7_CRIGR	Proteasome subunit beta type-6
G3HAQ4_CRIGR	Ubiquinol-cytochrome c reductase complex chaperone CBP3-like
G3HVP2_CRIGR	Stathmin (Fragment)
G3I018_CRIGR	26S proteasome non-ATPase regulatory subunit 7
G3I3C3_CRIGR	Tetratricopeptide repeat protein 15
G3HZK7_CRIGR	Heat shock factor protein 4
STIP1_CRIGR	Stress-induced-phosphoprotein 1

G3HM99_CRIGR	Phosphoglycerate mutase 2
M1R995_CRIGR	Phosphoribosylaminoimidazole carboxylase/ phosphoribosylaminoimidazole succinocarboxamide synthetase
G3IJJ0_CRIGR	NHP2-like protein 1
G3HB42_CRIGR	Heme-binding protein 1
M1ZMN4_CRIGR	Aldehyde oxidase 3L1
G3IPV6_CRIGR	26S proteasome non-ATPase regulatory subunit 3
G3HNV7_CRIGR	Glyceraldehyde-3-phosphate dehydrogenase
G3I970_CRIGR	Porphobilinogen deaminase
G3HE31_CRIGR	26S protease regulatory subunit 7
G3GWV4_CRIGR	Protein SET
G3I4A0_CRIGR	Actin, gamma-enteric smooth muscle (Fragment)
G3I877_CRIGR	Stress-induced-phosphoprotein 1
G3I9A7_CRIGR	Calmodulin
G3I7X5_CRIGR	Liver carboxylesterase 4
G3I6A1_CRIGR	Selenoprotein N
G3GZJ9_CRIGR	Dynamin-3
G3HID7_CRIGR	Pre-mRNA-splicing factor ISY1-like
G3HYI7_CRIGR	Splicing factor U2AF 65 kDa subunit
AIMP2_CRIGR	Aminoacyl tRNA synthase complex-interacting multifunctional protein 2
G3H666_CRIGR	Protein C15orf2
CREL2_CRIGR	Cysteine-rich with EGF-like domain protein 2
G3IKE2_CRIGR	40S ribosomal protein S15a
G3GVB5_CRIGR	Ras-related C3 botulinum toxin substrate 1
G3H018_CRIGR	40S ribosomal protein S18
GROA_CRIGR	Growth-regulated alpha protein
G3HH71_CRIGR	Putative RNA-binding protein Luc7-like 2
G3I351_CRIGR	40S ribosomal protein S9
G3GTN6_CRIGR	Peptidyl-prolyl cis-trans isomerase
G3HF18_CRIGR	Tudor domain-containing protein 12
G3GSL6_CRIGR	UBX domain-containing protein 7
G3I6V9_CRIGR	Protein CutA
Q3HR08_CRIGR	Guanine nucleotide binding protein alpha 13
G3HMH5_CRIGR	Guanine nucleotide-binding protein G(I), alpha-1 subunit
G3IN81_CRIGR	Uncharacterized protein C10orf78-like
G3IFS9_CRIGR	Uncharacterized protein KIAA1602-like
G3HHM2_CRIGR	Histone H3.1t
G3HIY6_CRIGR	40S ribosomal protein S19
G3GTX7_CRIGR	Enolase
G3IB23_CRIGR	Protocadherin beta-6
G3IB24_CRIGR	Protocadherin beta-17
G3IGT5_CRIGR	Sjoegren syndrome/scleroderma autoantigen 1
G3I0H2_CRIGR	40S ribosomal protein S4
G3I792_CRIGR	Peptidyl-prolyl cis-trans isomerase
G3HGW7_CRIGR	40S ribosomal protein S21
G3H011_CRIGR	40S ribosomal protein S15a
G3HNQ5_CRIGR	Phospholipase D3
G3HCX7_CRIGR	Phenylalanyl-tRNA synthetase alpha chain
G3HCL2_CRIGR	Tubulin beta-4 chain
G3HKN1_CRIGR	Protein SET
G3IK44_CRIGR	Fructose-bisphosphate aldolase
PARP1_CRIGR	Poly [ADP-ribose] polymerase 1
Q9JJN7_CRIGR	Carbonyl reductase
G3H1U2_CRIGR	Heterogeneous nuclear ribonucleoprotein A3
G3HV67_CRIGR	Histone H3.3
G3HWX4_CRIGR	Elongation factor 1-alpha
G3GWA7_CRIGR	40S ribosomal protein S6 (Fragment)
G3GTJ4_CRIGR	Glyceraldehyde-3-phosphate dehydrogenase
G3HIY3_CRIGR	40S ribosomal protein S23
G3GYC7_CRIGR	Enolase
G3HPJ9_CRIGR	Regulator of differentiation 1
G3ILH4_CRIGR	von Willebrand factor A domain-containing protein 5A
G3HSH4_CRIGR	60S ribosomal protein L24
G3H5A2_CRIGR	U2 small nuclear ribonucleoprotein A'
G3I8B3_CRIGR	TATA-binding protein-associated factor 2N
G3H1F4_CRIGR	Ribosomal protein L15
G3GXH4_CRIGR	Cell division protein kinase 6
G3GX44_CRIGR	Protein SEC13-like
G3HTQ5_CRIGR	CMP-N-acetylneuraminate-poly-alpha-2, 8-sialyltransferase
CALR_CRIGR	Calreticulin
G3HDT6_CRIGR	Histone H2A
G3IKQ5_CRIGR	Pyridoxal kinase
G3HPI0_CRIGR	40S ribosomal protein S4
G3IDS4_CRIGR	Rho-related GTP-binding protein RhoC
G3GZ97_CRIGR	Glyceraldehyde-3-phosphate dehydrogenase

G3IJ43_CRIGR	Pyruvate kinase
G3IJ85_CRIGR	Ubiquitin-conjugating enzyme E2 D3
G3HY47_CRIGR	Putative uncharacterized protein
G3HPQ9_CRIGR	Ferritin
G3HA87_CRIGR	Peptidyl-prolyl cis-trans isomerase A
G3HNG2_CRIGR	Acyl-coenzyme A thioesterase 2, mitochondrial
G3IG19_CRIGR	HD domain-containing protein 3
G3HE98_CRIGR	Tyrosine-protein kinase HCK
D0UZH9_CRIGR	Mutant adenylosuccinate lyase
G3H6Y9_CRIGR	Poly(RC)-binding protein 2
Q3HR12_CRIGR	Guanine nucleotide binding protein alpha inhibiting 3
BAIP2_CRIGR	Brain-specific angiogenesis inhibitor 1-associated protein 2
G3HUJ1_CRIGR	40S ribosomal protein S2
G3IC90_CRIGR	von Willebrand factor A domain-containing protein 5A
G3H5H7_CRIGR	Cathepsin O
Q4PR36_CRIGR	Brca2 (Fragment)
G3HEW0_CRIGR	Protein dpy-30-like
RSSA_CRIGR	40S ribosomal protein SA
VIME_CRIGR	Vimentin (Fragment)
Q60455_CRIGR	Beta tubulin
G3I5Q9_CRIGR	Ras-related protein Rab-11B
G3H0C9_CRIGR	L-lactate dehydrogenase A chain
G3HCB4_CRIGR	40S ribosomal protein S2
CD44_CRIGR	CD44 antigen
G3I9W7_CRIGR	Ras-related protein Rab-6A (Fragment)
G3HS40_CRIGR	H-2 class II histocompatibility antigen gamma chain
G3HH97_CRIGR	60S ribosomal protein L5-A
G3I9A3_CRIGR	Neudesin
G3HKK5_CRIGR	Glyceraldehyde-3-phosphate dehydrogenase
G3GZ13_CRIGR	Glyceraldehyde-3-phosphate dehydrogenase
G3HLW5_CRIGR	Ras-related protein Rab-5C
G3HSC1_CRIGR	Cysteine-rich protein 2
G3HDT2_CRIGR	Histone H3
G3ILV6_CRIGR	Rab GDP dissociation inhibitor beta
G3GX75_CRIGR	Hepatocyte growth factor-regulated tyrosine kinase substrate
G3HWJ6_CRIGR	Poly(U)-binding-splicing factor PUF60
G3I668_CRIGR	Guanine nucleotide-binding protein G(I)/G(S)/G(T) subunit beta-2
G3IJ02_CRIGR	Leucine-rich repeat flightless-interacting protein 1
G3HJV4_CRIGR	Myosin light polypeptide 6
G3H298_CRIGR	Serine/threonine-protein phosphatase
G3I9H0_CRIGR	Glyceraldehyde-3-phosphate dehydrogenase
G3HUU8_CRIGR	40S ribosomal protein S13
G3GUS2_CRIGR	ADP-ribosylation factor 4
G3I403_CRIGR	Serine/threonine-protein phosphatase PP1-alpha catalytic subunit
G3H208_CRIGR	Glyceraldehyde-3-phosphate dehydrogenase
G3H4R9_CRIGR	60S ribosomal protein L5
G3IFA9_CRIGR	Transcription elongation factor B polypeptide 2
G3I948_CRIGR	Eukaryotic translation initiation factor 5A-2
G3HZH3_CRIGR	Spliceosome RNA helicase BAT1
LAYN_CRIGR	Layilin
G3H453_CRIGR	Small nuclear ribonucleoprotein-associated protein
G3HLP7_CRIGR	Peptidyl-prolyl cis-trans isomerase
G3H8Y6_CRIGR	Poly(RC)-binding protein 3
G3GY22_CRIGR	Ras-related protein Rap-1b
G3HV39_CRIGR	Poly(RC)-binding protein 2
G3HBJ5_CRIGR	Elongation factor 1-gamma
G3IBX5_CRIGR	V-type proton ATPase subunit B, kidney isoform
G3HES2_CRIGR	Keratin, type II cytoskeletal 8
M1ZMR5_CRIGR	Aldehyde oxidase 4
G3HEN9_CRIGR	Septin-5
EF2_CRIGR	Elongation factor 2
G3HGY8_CRIGR	Elongation factor 1-alpha
G3IHP6_CRIGR	Elongation factor 1-alpha 1
G3HD67_CRIGR	Ribose-phosphate pyrophosphokinase 1
G3H588_CRIGR	Tubulin alpha-1B chain
G3I9N7_CRIGR	Enolase
G3GXV8_CRIGR	Myosin regulatory light polypeptide 9
G3IJG4_CRIGR	Glyceraldehyde-3-phosphate dehydrogenase
G3INX6_CRIGR	Spliceosome RNA helicase BAT1
G3GZ17_CRIGR	60S ribosomal protein L7
G3HH39_CRIGR	Elongation factor 1-alpha 1
G3I3G8_CRIGR	Transaldolase
GLNA_CRIGR	Glutamine synthetase
G3HP89_CRIGR	GTP-binding nuclear protein Ran

G3IF39_CRIGR	Carbonyl reductase [NADPH] 1
G3GX23_CRIGR	Actin, alpha skeletal muscle
G3I4U7_CRIGR	Isocitrate dehydrogenase [NADP] cytoplasmic
G3HZE6_CRIGR	Heat shock 70 kDa protein 1A/1B
G3ILE5_CRIGR	Heat shock cognate 71 kDa protein
G3IL02_CRIGR	Ubiquitin thioesterase OTUB1
G3HE52_CRIGR	Transcription factor BTF3
G3H1C4_CRIGR	Protein SET
G3I6X3_CRIGR	60S ribosomal protein L23a
G3HDG2_CRIGR	Zinc finger homeobox protein 2
G3I4N3_CRIGR	Lysine--tRNA ligase
Q6W3F5_CRIGR	Tumor necrosis factor-alpha-converting enzyme mutant variant M2b
G3I1S5_CRIGR	Glyceraldehyde-3-phosphate dehydrogenase
D0UZH8_CRIGR	Adenylosuccinate lyase
Q4F888_CRIGR	Class V beta tubulin
G3HZE5_CRIGR	Heat shock 70 kDa protein 1A
G3GTJ3_CRIGR	Glyceraldehyde-3-phosphate dehydrogenase
G3IC16_CRIGR	Cysteine-rich with EGF-like domain protein 2
G3H215_CRIGR	40S ribosomal protein SA
Q8VHU7_CRIGR	Eukaryotic translation initiation factor 5A isoform II (Fragment)
G3IIF1_CRIGR	Keratin, type II cuticular Hb6
G3HHX9_CRIGR	Ferritin
G3HQ80_CRIGR	Tubulin alpha-8 chain
HS105_CRIGR	Heat shock protein 105 kDa
G3ILL5_CRIGR	Transaldolase
G3GT56_CRIGR	Trifunctional purine biosynthetic protein adenosine-3
G3HZP8_CRIGR	Serine/threonine-protein phosphatase (Fragment)
G3HIC1_CRIGR	40S ribosomal protein S2
G3H1E5_CRIGR	Dynein light chain 2, cytoplasmic
G3IB21_CRIGR	Protocadherin beta-5
G3HY27_CRIGR	U2 small nuclear ribonucleoprotein B''
G3H7S7_CRIGR	Histone H3.3
G3HL66_CRIGR	40S ribosomal protein S11
G3HY78_CRIGR	Transcription factor BTF3
G3GZA1_CRIGR	Putative uncharacterized protein (Fragment)
G3HW57_CRIGR	Cell division protein kinase 4
G3HDY7_CRIGR	60S acidic ribosomal protein P0
G3HKG9_CRIGR	60S acidic ribosomal protein P0
G3GWF4_CRIGR	Heat shock protein 105 kDa (Fragment)
G3IDL8_CRIGR	Heat shock cognate 71 kDa protein
G3H7L4_CRIGR	40S ribosomal protein S18
Q924V3_CRIGR	Carbonyl reductase 1
G3HSB4_CRIGR	60S ribosomal protein L27
G3GSR4_CRIGR	Histone H3.3
O88679_CRIGR	TRIP protein (Fragment)
V5QSN9_CRIGR	Adenylosuccinate synthetase isozyme 2
G3ILF2_CRIGR	Glutathione S-transferase Mu 2
G3GSB7_CRIGR	60S ribosomal protein L7a
G3GVM3_CRIGR	Tubulin alpha-1C chain (Fragment)
G3H125_CRIGR	Neutral amino acid transporter A
G3GWV3_CRIGR	Alpha-enolase
G3I0M9_CRIGR	Sodium/potassium-transporting ATPase subunit alpha-3
G3HDT8_CRIGR	Histone H1t
G3H8P4_CRIGR	Heterogeneous nuclear ribonucleoprotein A1
G3HZH7_CRIGR	Serine/threonine-protein kinase PAK 3
G3HKV0_CRIGR	Glyceraldehyde-3-phosphate dehydrogenase
G3GRE4_CRIGR	Peptidyl-prolyl cis-trans isomerase A
G3HVQ6_CRIGR	Low-density lipoprotein receptor
G3IAI3_CRIGR	60S ribosomal protein L17
G3HDS1_CRIGR	Histone H4
G3HPV7_CRIGR	Histone H4
G3H3C6_CRIGR	Tubulin alpha-3 chain
G3HJY9_CRIGR	Histone H2B type 1
G3HMW2_CRIGR	40S ribosomal protein S4
G3IFQ9_CRIGR	40S ribosomal protein S25
G3H2U0_CRIGR	Histone H2B
G3H627_CRIGR	Serine/threonine-protein phosphatase (Fragment)
G3HDV4_CRIGR	Histone H2B
Q924V2_CRIGR	Carbonyl reductase 2
G3IA30_CRIGR	60S acidic ribosomal protein P1
G3HXF7_CRIGR	Glyceraldehyde-3-phosphate dehydrogenase
G3HWN6_CRIGR	DNA topoisomerase 1 (Fragment)
G3GY47_CRIGR	60S ribosomal protein L26
G3HYZ5_CRIGR	Glyceraldehyde-3-phosphate dehydrogenase

G3HUE9_CRIGR	High mobility group protein B1
G3HYZ4_CRIGR	Glyceraldehyde-3-phosphate dehydrogenase
G3GVL9_CRIGR	60S ribosomal protein L30
G3H7J9_CRIGR	60S ribosomal protein L31
G3H061_CRIGR	High mobility group protein B1
Q8MHC3_CRIGR	MHC class I antigen Hm1-C3
G3H2G1_CRIGR	Layilin
G3HLB3_CRIGR	Glutamine synthetase
G3H2T7_CRIGR	Histone H3
G3I6W8_CRIGR	40S ribosomal protein S18
G3GWG3_CRIGR	High mobility group protein B1
G3GZL5_CRIGR	High mobility group protein B1
G3IBI0_CRIGR	Ras-related protein Rap-1A
G3IL84_CRIGR	Oligoribonuclease, mitochondrial
G3IHS0_CRIGR	Glyceraldehyde-3-phosphate dehydrogenase
RS14_CRIGR	40S ribosomal protein S14
G3HSC9_CRIGR	60S ribosomal protein L7
G3IGI0_CRIGR	60S ribosomal protein L23a
G3I1A7_CRIGR	Peptidyl-prolyl cis-trans isomerase A
G3GSD8_CRIGR	Glyceraldehyde-3-phosphate dehydrogenase
G3HYZ9_CRIGR	Actin-related protein 3B
G3H9M7_CRIGR	Glyceraldehyde-3-phosphate dehydrogenase
G3I583_CRIGR	40S ribosomal protein S26
G3I129_CRIGR	Ubiquitin
G3GUM6_CRIGR	Glyceraldehyde-3-phosphate dehydrogenase
O35080_CRIGR	Polyubiquitin
G3I364_CRIGR	40S ribosomal protein S25
G3HDS3_CRIGR	Histone H2A
G3I9E7_CRIGR	Pyridoxal kinase
Q99MH4_CRIGR	Lactate dehydrogenase (Fragment)
G3HCC0_CRIGR	60S ribosomal protein L27
G3I5T9_CRIGR	Calmodulin (Fragment)
G3GXN1_CRIGR	GTP-binding nuclear protein Ran
G3HBH4_CRIGR	Low molecular weight phosphotyrosine protein phosphatase
G3INM4_CRIGR	H-2 class I histocompatibility antigen, L-D alpha chain
G3HX34_CRIGR	60S ribosomal protein L7
G3IKQ4_CRIGR	Pyridoxal kinase
G3I7G4_CRIGR	40S ribosomal protein S2
G3HMW8_CRIGR	Tubulin beta-6 chain
G3H4X7_CRIGR	Calumenin
G3H5I3_CRIGR	Glyceraldehyde-3-phosphate dehydrogenase
G3GVM6_CRIGR	60S ribosomal protein L12
G3I0W1_CRIGR	Alpha-enolase
G3HDV5_CRIGR	Histone H2A
G3I8J8_CRIGR	60S ribosomal protein L23a
HYOU1_CRIGR	Hypoxia up-regulated protein 1
G3HZE3_CRIGR	Sialidase-1
Q9Z1W7_CRIGR	GP50
Q6W3F7_CRIGR	Tumor necrosis factor-alpha-converting enzyme mutant variant M1
EF1A1_CRIGR	Elongation factor 1-alpha 1
G3I1X8_CRIGR	Guanine nucleotide-binding protein G(I)/G(S)/G(T) subunit beta-1
G3HJW0_CRIGR	Glyceraldehyde-3-phosphate dehydrogenase
G3I044_CRIGR	Glyceraldehyde-3-phosphate dehydrogenase
SYK_CRIGR	Lysine--tRNA ligase
G3HFJ0_CRIGR	CMP-N-acetylneuraminate-beta-galactosamide-alpha-2, 3-sialyltransferase
G3HUD3_CRIGR	Proteasome activator complex subunit 1
TALDO_CRIGR	Transaldolase
Q6TMK7_CRIGR	Ribosomal protein Rps2
G3I0B0_CRIGR	Ras-related protein Rab-11A
G3HYV9_CRIGR	40S ribosomal protein S2
G3IDL7_CRIGR	Heat shock cognate 71 kDa protein
G3HV73_CRIGR	Poly [ADP-ribose] polymerase 1
G3IBY4_CRIGR	Plastin-3
G3GR87_CRIGR	Protein SET
G3HQ39_CRIGR	60S ribosomal protein L23a
G3I3E3_CRIGR	Ubiquitin-conjugating enzyme E2 variant 2
G3GU30_CRIGR	60S ribosomal protein L6
G3HHK3_CRIGR	60S ribosomal protein L31
G3INF1_CRIGR	40S ribosomal protein S26
B8Y440_CRIGR	Sialidase I
G3HJD8_CRIGR	40S ribosomal protein S16
G3I5Y2_CRIGR	40S ribosomal protein S16
GBB1_CRIGR	Guanine nucleotide-binding protein G(I)/G(S)/G(T) subunit beta-1
B2ZA78_CRIGR	Phosphoribosylglycinamide transformylase

G3H2T4_CRIGR	Histone H2B
Q60454_CRIGR	Beta tubulin
G3GZ08_CRIGR	Tubulin beta-2 chain
G3GXC8_CRIGR	Ras-related C3 botulinum toxin substrate 3
TOP1_CRIGR	DNA topoisomerase 1
G3INP0_CRIGR	Spliceosome RNA helicase Bat1
M1R372_CRIGR	Phosphoribosylaminoimidazole carboxylase/phosphoribosylaminoimidazole succinocarboxamide synthetase
Q7M080_CRIGR	DnaK-type molecular chaperone (Fragments)
G3HDU3_CRIGR	Histone H2B
G3IH36_CRIGR	Small ubiquitin-related modifier 2
KAPCA_CRIGR	cAMP-dependent protein kinase catalytic subunit alpha
G3H7M3_CRIGR	Adenylosuccinate synthetase
G3H5Q0_CRIGR	Actin, alpha cardiac muscle 1
G3H1U3_CRIGR	40S ribosomal protein S15a
G3IDC2_CRIGR	Heat shock cognate 71 kDa protein
G3I979_CRIGR	40S ribosomal protein S25 (Fragment)
G3HQX0_CRIGR	40S ribosomal protein SA
G3IF43_CRIGR	Carbonyl reductase [NADPH] 1
G3IPD7_CRIGR	14-3-3 protein gamma
G3HA25_CRIGR	14-3-3 protein gamma
G3IAB9_CRIGR	Eukaryotic translation initiation factor 4E
G3ILP4_CRIGR	60S ribosomal protein L9
G3HDT9_CRIGR	Histone H4
G3HPV6_CRIGR	Histone H2A type 1
G3GX66_CRIGR	Actin, cytoplasmic 2
G3P_CRIGR	Glyceraldehyde-3-phosphate dehydrogenase
G3HD12_CRIGR	cAMP-dependent protein kinase catalytic subunit alpha
G3HPV1_CRIGR	Histone H2B
G3HXX5_CRIGR	Eukaryotic translation initiation factor 5A-1
G3GY37_CRIGR	60S ribosomal protein L9
G3HIC0_CRIGR	40S ribosomal protein S2
G3HPH9_CRIGR	CMP-N-acetylneuraminate-beta-galactosamide-alpha-2, 3-sialyltransferase
G3IHH8_CRIGR	Disintegrin and metalloproteinase domain-containing protein 17 (Fragment)
G3IF46_CRIGR	Carbonyl reductase [NADPH] 3
G3GTT3_CRIGR	C-C motif chemokine 7
G3IA26_CRIGR	Heparanase
G3IAP3_CRIGR	Connective tissue growth factor
G3I2U1_CRIGR	Serine protease 27
G3HYE5_CRIGR	Protocadherin gamma-C3
G3INI9_CRIGR	NKG2D ligand 1
Q80WN6_CRIGR	Beta-1,4-galactosyltransferase 5
G3HCG5_CRIGR	Semaphorin-6B
G3HLR8_CRIGR	WNT1-inducible-signaling pathway protein 2
G3HHC1_CRIGR	Protein phosphatase 1L
G3HUR5_CRIGR	Tsukushin
G3HXN5_CRIGR	Beta-hexosaminidase subunit beta
G3H893_CRIGR	Abhydrolase domain-containing protein 14A
G3HFY2_CRIGR	60S ribosomal protein L12
Q80WL0_CRIGR	Gal beta-1,3/4-GlcNAc alpha-2,3-sialyltransferase St3Gal IV
G3I369_CRIGR	Golgi apparatus protein 1
G3I0Q1_CRIGR	40S ribosomal protein S26
G3IB22_CRIGR	Protocadherin-3
G3HV98_CRIGR	Ribose-phosphate pyrophosphokinase 1
A0PA08_CRIGR	Perlecan (Fragment)
G3HRK4_CRIGR	Ras-related protein Rab-5B
G3H7L1_CRIGR	60S ribosomal protein L23a
G3IF44_CRIGR	Carbonyl reductase [NADPH] 1
G3I9F0_CRIGR	General vesicular transport factor p115
G3GVT2_CRIGR	Far upstream element-binding protein 1
G3HGF9_CRIGR	Protein transport protein Sec23A
G3IEK1_CRIGR	Actin-related protein 2/3 complex subunit 2
G3IK13_CRIGR	Eukaryotic translation initiation factor 3 subunit C
G3GXY4_CRIGR	Regulation of nuclear pre-mRNA domain-containing protein 1B
G3HRD3_CRIGR	Protein arginine N-methyltransferase 5
APT_CRIGR	Adenine phosphoribosyltransferase
G3H2V3_CRIGR	Acidic leucine-rich nuclear phosphoprotein 32 family member E
G3HDG6_CRIGR	Paraspeckle component 1
G3HAU1_CRIGR	Eukaryotic translation initiation factor 3 subunit E
G3I7Q5_CRIGR	Zinc finger protein ZPR1
G3HCV7_CRIGR	Deoxyhypusine synthase
G3IE04_CRIGR	Pre-mRNA-processing factor 19
G3I873_CRIGR	Ubiquitin thioesterase OTUB1
G3I0M2_CRIGR	Platelet-activating factor acetylhydrolase IB subunit gamma
G3HUU7_CRIGR	Protein S100-A10

G3I8D0_CRIGR	Suppressor of G2 allele of SKP1-like
G3H892_CRIGR	Aminoacylase-1A
G3HRM9_CRIGR	Prostaglandin E synthase 3 (Fragment)
G3I249_CRIGR	Selenocysteine lyase
G3GUK0_CRIGR	Putative fructose-2,6-bisphosphatase TIGAR
G3H5W5_CRIGR	LIM and SH3 domain protein 1
G3INN8_CRIGR	Microtubule-associated protein 6
G3IP86_CRIGR	Replication protein A 70 kDa DNA-binding subunit
G3GVW9_CRIGR	Sorting nexin-5
G3H6W0_CRIGR	V-type proton ATPase subunit B, brain isoform (Fragment)
G3H9C9_CRIGR	Transcription elongation factor B polypeptide 1
G3HYT5_CRIGR	2'-deoxynucleoside 5'-phosphate N-hydrolase 1
G3HAR2_CRIGR	Copine-1
G3HNV5_CRIGR	U6 snRNA-associated Sm-like protein LSm8
G3IKQ9_CRIGR	Osteopontin
G3GWQ3_CRIGR	Adenosine kinase
G3I000_CRIGR	Aldehyde dehydrogenase family 16 member A1
G3I0Q4_CRIGR	Platelet-activating factor acetylhydrolase IB subunit beta
G3I7M1_CRIGR	Ataxin-2-like protein
G3GVT1_CRIGR	DnaJ-like subfamily B member 4
G3I1P0_CRIGR	Isochorismatase domain-containing protein 1
MCA3_CRIGR	Eukaryotic translation elongation factor 1 epsilon-1
G3GX38_CRIGR	Putative uncharacterized protein
G3I784_CRIGR	Enolase-phosphatase E1
G3GRT8_CRIGR	Diphosphomevalonate decarboxylase
G3HE59_CRIGR	TIP41-like protein
G3I2G0_CRIGR	UPF0696 protein C11orf68-like
G3I8T9_CRIGR	60S ribosomal protein L6
G3HHV6_CRIGR	Protein FAM98B
G3ILW0_CRIGR	Serine/threonine-protein kinase PAK 1
G3H7R2_CRIGR	Eukaryotic translation initiation factor 3 subunit A
G3GY31_CRIGR	Cleavage and polyadenylation specificity factor subunit 6
G3HEL2_CRIGR	Glucosamine-6-phosphate isomerase 1 (Fragment)
G3GRH9_CRIGR	Tropomodulin-3
G3IE25_CRIGR	Clathrin light chain A
G3IJD6_CRIGR	Putative D-tyrosyl-tRNA(Tyr) deacylase 2
G3HQ40_CRIGR	Eukaryotic peptide chain release factor GTP-binding subunit ERF3B
G3I388_CRIGR	28 kDa heat-and acid-stable phosphoprotein
G3HIQ3_CRIGR	Activated RNA polymerase II transcriptional coactivator p15
G3GU92_CRIGR	Lupus La protein-like
G3H2A5_CRIGR	Vacuolar protein sorting-associated protein 29
G3HMB9_CRIGR	Histone H2A
G3I308_CRIGR	26S proteasome non-ATPase regulatory subunit 2
G3H6T8_CRIGR	Vacuolar protein sorting-associated protein 26A
G3HTF7_CRIGR	Myosin-Ic
G3H1M5_CRIGR	V-type proton ATPase subunit D
G3H6K9_CRIGR	Four and a half LIM domains protein 1
G3HVC7_CRIGR	Diphthamide biosynthesis protein 1
G3IIF4_CRIGR	Keratin, type II cuticular Hb2
G3I0N6_CRIGR	Coiled-coil domain-containing protein 6
G3H9Y7_CRIGR	NudC domain-containing protein 2
G3II47_CRIGR	Aconitate hydratase, mitochondrial
G3H265_CRIGR	Diablo-like, mitochondrial
G3HJD6_CRIGR	Pleckstrin-likey domain-containing family G member 2
G3HQ99_CRIGR	Programmed cell death protein 10
G3HAJ9_CRIGR	Coiled-coil domain-containing protein 58
G3HS58_CRIGR	Putative E3 ubiquitin-protein ligase MGRN1
G3HUK8_CRIGR	NIF3-like protein 1
G3I4N5_CRIGR	Gamma-aminobutyric acid receptor-associated protein-like 2
G3IJB9_CRIGR	UDP-N-acetylhexosamine pyrophosphorylase-like protein 1
GNAS_CRIGR	Guanine nucleotide-binding protein G(s) subunit alpha
G3GSK4_CRIGR	Serine/threonine-protein kinase PAK 2
G3HFG7_CRIGR	Putative hydroxypyruvate isomerase
G3HJH9_CRIGR	Replication protein A 14 kDa subunit
G3HDU1_CRIGR	Putative uncharacterized protein
G3HNP6_CRIGR	26S protease regulatory subunit 6B (Fragment)
G3GRE0_CRIGR	3'(2'),5'-bisphosphate nucleotidase 1
G3I7K0_CRIGR	Neuroligin-3
G3IBG4_CRIGR	Serine/threonine-protein kinase PCTAIRE-1
G3HHY9_CRIGR	V-type proton ATPase subunit G 1
G3IIF2_CRIGR	Keratin, type II cuticular Hb5
G3H2A8_CRIGR	Actin-related protein 2/3 complex subunit 3 (Fragment)
G3HID6_CRIGR	Cellular nucleic acid-binding protein
G3HL89_CRIGR	Serine/threonine-protein kinase PFTAIRE-1

G3IDF4_CRIGR	Arfaptin-2
G3I8V8_CRIGR	DnaJ-like subfamily A member 1
A9XHW5_CRIGR	CCHC-type zinc finger (Fragment)
G3HP90_CRIGR	40S ribosomal protein S2
G3HMJ8_CRIGR	60S ribosomal protein L7
G3HDS7_CRIGR	Histone H1.3
G3HEK4_CRIGR	Protein SET
G3IH03_CRIGR	Fructose-bisphosphate aldolase
G3HKT3_CRIGR	Heterogeneous nuclear ribonucleoprotein F
G3HPU9_CRIGR	60S ribosomal protein L23a
G3H1G2_CRIGR	Eukaryotic translation initiation factor 4E-binding protein 1
G3GWT7_CRIGR	Guanine nucleotide-binding protein G(S) subunit alpha isoforms XLas
G3HRU8_CRIGR	Heterogeneous nuclear ribonucleoprotein A3
G3GSH7_CRIGR	60S ribosomal protein L23a
G3I2N7_CRIGR	60S ribosomal protein L23a
G3H954_CRIGR	Putative uncharacterized protein
G3H8L7_CRIGR	Ras-related protein Rab-5A
HMGB1_CRIGR	High mobility group protein B1 (Fragment)
G3IMT7_CRIGR	Keratin, type II cuticular Hb3
P70098_CRIGR	BRCA2 (Fragment)
G3HEC7_CRIGR	Ubiquitin-conjugating enzyme E2 D2B
G3GRH2_CRIGR	60S ribosomal protein L23a
Q3LRD4_CRIGR	Guanine nucleotide binding protein alpha inhibiting 1
G3IQD3_CRIGR	Spliceosome RNA helicase Bat1
G3I100_CRIGR	60S ribosomal protein L27
BST2_CRIGR	Bone marrow stromal antigen 2
G3H4E0_CRIGR	Bone marrow stromal antigen 2
G3HF33_CRIGR	60S acidic ribosomal protein P1
G3I3J5_CRIGR	40S ribosomal protein S25
G3IGQ2_CRIGR	Heat shock cognate 71 kDa protein
G3H3K6_CRIGR	60S ribosomal protein L23a
G3IFR0_CRIGR	60S ribosomal protein L7
G3IGL4_CRIGR	40S ribosomal protein S21
G3I9M5_CRIGR	Dynein light chain 1, cytoplasmic
AK1A1_CRIGR	Alcohol dehydrogenase [NADP(+)] (Fragment)
G3HK42_CRIGR	60S ribosomal protein L30
Q80ZC6_CRIGR	Neutral amino acid transporter type 1
G3I1I8_CRIGR	Small nuclear ribonucleoprotein-associated protein
G3H2D0_CRIGR	Serine/threonine-protein phosphatase
G3I8X0_CRIGR	60S ribosomal protein L19
G3HND4_CRIGR	40S ribosomal protein S15a
G3IAI6_CRIGR	Heme oxygenase 1
G3HVB8_CRIGR	Platelet-activating factor acetylhydrolase IB subunit alpha
G3I8H9_CRIGR	Cysteine and histidine-rich domain-containing protein 1
G3HYG7_CRIGR	DnaJ-like subfamily B member 11
G3H229_CRIGR	GMP synthase [glutamine-hydrolyzing]
G3HTI3_CRIGR	Putative G-protein coupled receptor 111
G3HWJ5_CRIGR	Poly(U)-binding-splicing factor PUF60
G3HE04_CRIGR	Splicing factor 3A subunit 3
G3H4D3_CRIGR	6-phosphogluconolactonase
G3IK83_CRIGR	Protein transport protein Sec24D
G3HDD1_CRIGR	Geranylgeranyl transferase type-2 subunit alpha
G3GXB5_CRIGR	Ethanolamine-phosphate cytidylyltransferase
G3HHX0_CRIGR	Interleukin-6 receptor subunit beta
G3HC25_CRIGR	Protein S100-A13
G3HX09_CRIGR	FK506-binding protein 14
G3HKJ6_CRIGR	Myc box-dependent-interacting protein 1 (Fragment)
G3H3P5_CRIGR	Kinesin-1 heavy chain
G3IM40_CRIGR	26S proteasome non-ATPase regulatory subunit 1
G3HPY5_CRIGR	U4/U6.U5 tri-snRNP-associated protein 2
OFUT1_CRIGR	GDP-fucose protein O-fucosyltransferase 1
G3HRT4_CRIGR	Fibrillin-1
G3HIY9_CRIGR	Glutamate--cysteine ligase catalytic subunit
G3H530_CRIGR	Sorting nexin-1
G3H8F2_CRIGR	Acylamino-acid-releasing enzyme
G3I5F4_CRIGR	Protein canopy-like 4
G3H6F2_CRIGR	Peptidyl-prolyl cis-trans isomerase NIMA-interacting 1
G3HXR8_CRIGR	Phosphatidylinositol-binding clathrin assembly protein
G3H7C1_CRIGR	Glia maturation factor beta
G3HJB6_CRIGR	Ras and Rab interactor-like protein
G3HUP4_CRIGR	Uncharacterized protein C7orf50-like
G3HA77_CRIGR	Stonin-2
G3I7X7_CRIGR	Liver carboxylesterase 1
G3HJM5_CRIGR	Gamma-glutamylcyclotransferase

SORCN_CRIGR	Sorcin
C6H0P7_CRIGR	Antrax toxin receptor 2 (Fragment)
G3IFX0_CRIGR	Programmed cell death protein 6
G3HQ89_CRIGR	V-type proton ATPase subunit E 1
G3I7D7_CRIGR	RNA-binding protein FUS
G3H7F1_CRIGR	Nuclear cap-binding protein subunit 1
G3GTV9_CRIGR	Vacuolar protein sorting-associated protein 26B
G3H9I6_CRIGR	Testin
G3I245_CRIGR	Integrin-linked kinase-associated serine/threonine phosphatase 2C
G3I5F0_CRIGR	COP9 signalosome complex subunit 6
G3IN18_CRIGR	60S ribosomal protein L8
G3GUB2_CRIGR	Cytoplasmic dynein 1 intermediate chain 2 (Fragment)
G3HDD5_CRIGR	Magnesium-dependent phosphatase 1
G3IND2_CRIGR	26S proteasome non-ATPase regulatory subunit 13
G3HLM6_CRIGR	Protein HEXIM1
G3GTU8_CRIGR	Notchless protein-like 1
G3H1Y8_CRIGR	Regulator of chromosome condensation
G3HN43_CRIGR	High mobility group protein B1
G3I390_CRIGR	Actin-related protein 2/3 complex subunit 1B
G3HUE5_CRIGR	UPF0368 protein Cxorf26-like
G3HBI7_CRIGR	Serine/threonine-protein kinase PCTAIRE-2
G3HNR3_CRIGR	U1 small nuclear ribonucleoprotein A
G3H9M6_CRIGR	Glyceraldehyde-3-phosphate dehydrogenase
G3H4K0_CRIGR	High mobility group protein B1
G3HE39_CRIGR	Oxidoreductase HTATIP2
G3H8Q0_CRIGR	ADP-ribosylation factor 1
G3H1Q6_CRIGR	Beta-centractin
G3HM00_CRIGR	60S ribosomal protein L27
G3I8Z5_CRIGR	Sulfiredoxin
G3HHM7_CRIGR	Guanylate kinase
G3HNP4_CRIGR	rRNA 2'-O-methyltransferase fibrillarin
G3I0Z2_CRIGR	Alpha-soluble NSF attachment protein
Q925U5_CRIGR	Adenosine kinase (Fragment)
G3GS93_CRIGR	Stress-induced-phosphoprotein 1
G3H8Z7_CRIGR	Ubiquitin-conjugating enzyme E2 G2 (Fragment)
G3HMS8_CRIGR	60S ribosomal protein L17
G3I5L0_CRIGR	Liver carboxylesterase 4
G3I3W6_CRIGR	60S ribosomal protein L27a
G3I9X3_CRIGR	Protein SET
G3HGH1_CRIGR	High mobility group protein B1
G3ICQ8_CRIGR	60S ribosomal protein L7
G3HRX0_CRIGR	Sorcin (Fragment)
PYRG1_CRIGR	CTP synthase 1 (Fragment)
G3IFT9_CRIGR	Actin-related protein 2
G3HZH5_CRIGR	H-2 class I histocompatibility antigen, L-D alpha chain
G3HV32_CRIGR	Histone H3.3 type 1
G3GZV3_CRIGR	60S acidic ribosomal protein P2
G3I7W4_CRIGR	Dynactin subunit 1
G3HMB6_CRIGR	Putative uncharacterized protein
TSN_CRIGR	Translin
G3HL93_CRIGR	Muscleblind-like protein 1
G3HQH8_CRIGR	Tubulin alpha-1C chain
G3HCN2_CRIGR	CTP synthase
G3H3V7_CRIGR	Prefoldin subunit 3
G3HTC5_CRIGR	40S ribosomal protein S23
G3HZY6_CRIGR	Lin-7-like B
G3GR58_CRIGR	60S ribosomal protein L26
G3I5T4_CRIGR	Septin-7
G3H9A3_CRIGR	60S ribosomal protein L7a
G3GTK2_CRIGR	60S ribosomal protein L12
G3HPW0_CRIGR	Histone H2A
G3HUT1_CRIGR	Carbonyl reductase [NADPH] 1
G3IF40_CRIGR	Carbonyl reductase [NADPH] 1
G3I5N9_CRIGR	Peptidyl-prolyl cis-trans isomerase
G3IGQ4_CRIGR	Heat shock cognate 71 kDa protein
G3H4Q1_CRIGR	Histone H2A
G3HK68_CRIGR	Glyceraldehyde-3-phosphate dehydrogenase
G3HNG3_CRIGR	Acyl-coenzyme A thioesterase 1
G3GWM6_CRIGR	Peptidyl-prolyl cis-trans isomerase
G3H3Y8_CRIGR	60S ribosomal protein L32
G3GR77_CRIGR	Protein SET (Fragment)
G3HI14_CRIGR	Eukaryotic translation initiation factor 1
G3IM75_CRIGR	60S ribosomal protein L32
G3I535_CRIGR	40S ribosomal protein S2

G3GSM5_CRIGR	Transferrin receptor protein 1
TFR1_CRIGR	Transferrin receptor protein 1
SUMO2_CRIGR	Small ubiquitin-related modifier 2
G3IIH3_CRIGR	Eukaryotic translation initiation factor 4E
G3HTU9_CRIGR	Histone H3.3
G3HDJ3_CRIGR	Syndecan
G3HVW6_CRIGR	Interleukin-6
G3HEI9_CRIGR	CD276 antigen
G3HJP6_CRIGR	Prohibitin
O55077_CRIGR	Cyclin-dependent kinase 2 (CDK2L)
G3H667_CRIGR	Polyadenylate-binding protein 1
G3H5K5_CRIGR	60S ribosomal protein L19
G3GW02_CRIGR	60S ribosomal protein L18
G3HJV9_CRIGR	Glyceraldehyde-3-phosphate dehydrogenase
G3IC50_CRIGR	High mobility group protein B2 (Fragment)
G3H5G9_CRIGR	Putative uncharacterized protein
G3H6R4_CRIGR	60S ribosomal protein L23a
G3IM64_CRIGR	40S ribosomal protein S8
G3I1A1_CRIGR	40S ribosomal protein SA
G3HQI9_CRIGR	60S ribosomal protein L23a
G3HMB8_CRIGR	Peptidyl-prolyl cis-trans isomerase
G3IHV9_CRIGR	Poly(RC)-binding protein 2
G3GS76_CRIGR	Heparan-sulfate 6-O-sulfotransferase 3
G3HQ11_CRIGR	40S ribosomal protein SA
G3HXP3_CRIGR	Acyl-CoA-binding protein (Fragment)
G3IF49_CRIGR	40S ribosomal protein S4
G3ICC1_CRIGR	60S ribosomal protein L6
Q60456_CRIGR	Beta tubulin (Fragment)
G3HUE3_CRIGR	Isocitrate dehydrogenase [NADP] cytoplasmic
G3IIE9_CRIGR	Keratin, type II cuticular Hb3
G3ICB6_CRIGR	Elongation factor 1-alpha 1
G3IQ10_CRIGR	Proteasome subunit beta type-3
G3GWQ0_CRIGR	40S ribosomal protein S15a
G3IEG6_CRIGR	Tubulin beta-2A chain
G3GSG3_CRIGR	40S ribosomal protein S2
G3GVL0_CRIGR	Ubiquitin
G3GV60_CRIGR	High mobility group protein 1-like 10
G3H216_CRIGR	60S ribosomal protein L23a
G3I0R3_CRIGR	Nicotinamide phosphoribosyltransferase (Fragment)
G3HFI6_CRIGR	ATP-dependent RNA helicase DDX1
G3I8B9_CRIGR	AP-2 complex subunit beta
G3GTN1_CRIGR	Protein mago nashi-like
G3I887_CRIGR	Peroxiredoxin-5, mitochondrial
G3GUM9_CRIGR	Putative ribosomal RNA methyltransferase NOP2
G3IA32_CRIGR	COP9 signalosome complex subunit 4
G3GTE4_CRIGR	Growth factor receptor-bound protein 2
G3IEY5_CRIGR	Dynamin-1-like protein
G3HG78_CRIGR	Apolipoprotein A-I-binding protein
G3H1Z4_CRIGR	DnaJ-like subfamily C member 8
G3IG96_CRIGR	Glutamate--cysteine ligase regulatory subunit
G3H0F1_CRIGR	Alpha-mannosidase 2C1
G3H0A7_CRIGR	Eukaryotic translation initiation factor 3 subunit H (Fragment)
G3IL63_CRIGR	Caspase-7
G3H894_CRIGR	Abhydrolase domain-containing protein 14B
G3ID93_CRIGR	WD repeat-containing protein 5
G3I5V6_CRIGR	Mitotic checkpoint protein BUB3
G3ILH7_CRIGR	26S proteasome non-ATPase regulatory subunit 1
G3IFL1_CRIGR	Amidophosphoribosyltransferase
G3H0T9_CRIGR	Huntingtin-interacting protein K
G3HCF8_CRIGR	Endophilin-A2
G3H7F9_CRIGR	Sialic acid synthase
G3HQ86_CRIGR	BH3-interacting domain death agonist
G3I5W2_CRIGR	TAR DNA-binding protein 43
G3HAP6_CRIGR	Acetyl-coenzyme A synthetase, cytoplasmic
Q6E6J6_CRIGR	Chromobox protein-like 5
G3HU11_CRIGR	Nitrilase-like 1
G3GYJ1_CRIGR	Rho GTPase-activating protein 1
G3HZY5_CRIGR	U1 small nuclear ribonucleoprotein 70 kDa
G3H5M8_CRIGR	HEAT repeat-containing protein 3
G3H2Z7_CRIGR	26S proteasome non-ATPase regulatory subunit 4
G3HZG2_CRIGR	Casein kinase II subunit beta
G3IEX2_CRIGR	Nuclease-sensitive element-binding protein 1
G3H2S5_CRIGR	RNA-binding protein 8A
G3GYV4_CRIGR	Ribosome biogenesis protein WDR12

MP2K1_CRIGR	Dual specificity mitogen-activated protein kinase kinase 1
G3H2U6_CRIGR	Splicing factor 3B subunit 4
G3IGN7_CRIGR	BRCA2 and CDKN1A-interacting protein
G3HFF0_CRIGR	Solute carrier family 2, facilitated glucose transporter member 1 (Fragment)
G3HJA4_CRIGR	26S proteasome non-ATPase regulatory subunit 8
G3I2X3_CRIGR	Casein kinase II subunit alpha'
G3I8Z3_CRIGR	TBC1 domain family member 20
G3H8Q6_CRIGR	Importin subunit alpha
G3HV97_CRIGR	Ribose-phosphate pyrophosphokinase 1
G3I501_CRIGR	Histone H1.0
G3HSZ7_CRIGR	Heterogeneous nuclear ribonucleoprotein L-like
G3II02_CRIGR	Serine/threonine-protein phosphatase 2A regulatory subunit B' (Fragment)
G3HYG9_CRIGR	Mitochondrial import inner membrane translocase subunit Tim13
G3I581_CRIGR	Prefoldin subunit 1
G3HH92_CRIGR	Pirin
G3I3Y7_CRIGR	Glutathione S-transferase P 2
G3H1W2_CRIGR	Peflin
G3HJA9_CRIGR	Eukaryotic translation initiation factor 3 subunit K (Fragment)
G3GXD0_CRIGR	L-xylulose reductase
G3HY21_CRIGR	Beta-ureidopropionase
G3ICB9_CRIGR	Nucleolysin TIAR
G3HMX7_CRIGR	Polyadenylate-binding protein-interacting protein 1
G3I6E3_CRIGR	Lin-7-like C
G3HVZ6_CRIGR	Small nuclear ribonucleoprotein G
G3HFY6_CRIGR	Stromal cell-derived factor 2-like protein 1
G3II12_CRIGR	Calcium-binding protein 39
G3I3C4_CRIGR	1,2-dihydroxy-3-keto-5-methylthiopentene dioxygenase
G3I054_CRIGR	Proteasome assembly chaperone 1
G3GVZ1_CRIGR	Transcription factor BTF3-like 4
G3II46_CRIGR	PHD finger-like domain-containing protein 5A
G3GZ09_CRIGR	Proteasome assembly chaperone 4
G3H6X4_CRIGR	Splicing factor 3B subunit 5
G3IEG3_CRIGR	Ribosyldihydronicotinamide dehydrogenase [quinone]
G3HHK4_CRIGR	Phosducin-like protein 3
G3GS73_CRIGR	Heterogeneous nuclear ribonucleoprotein A3-like 1
G3IKR0_CRIGR	dCTP pyrophosphatase 1
G3HXH2_CRIGR	Microtubule-associated proteins 1A/1B light chain 3B
G3HZE8_CRIGR	U6 snRNA-associated Sm-like protein LSm2 (Fragment)
G3II56_CRIGR	Actin-related protein 2
G3IIV0_CRIGR	Ubiquitin domain-containing protein UBFD1
G3GZ95_CRIGR	V-type proton ATPase subunit F
G3HY07_CRIGR	Glutathione S-transferase theta-2
G3I2L7_CRIGR	Coiled-coil-helix-coiled-coil-helix domain-containing protein 2, mitochondrial
G3GVM9_CRIGR	Calcium-regulated heat stable protein 1
G3HED6_CRIGR	Protein Dr1
G3HXS2_CRIGR	Uncharacterized protein C11orf73-like
G3IHB4_CRIGR	Endothelial differentiation-related factor 1
G3HEF9_CRIGR	CB1 cannabinoid receptor-interacting protein 1
G3H3W5_CRIGR	Nuclear ubiquitous casein and cyclin-dependent kinases substrate
G3ILJ8_CRIGR	Actin-related protein 2
G3HDG0_CRIGR	Thiamine-triphosphatase
G3I3C2_CRIGR	Protein TSSC1
G3IK70_CRIGR	PDZ and LIM domain protein 5
G3H5F7_CRIGR	Asparaginyl-tRNA synthetase, cytoplasmic
G3HLM2_CRIGR	Glycylpeptide N-tetradecanoyltransferase
G3I2F9_CRIGR	Dr1-associated corepressor
G3HWN3_CRIGR	Actin-related protein 2/3 complex subunit 1B
G3HWZ3_CRIGR	60S ribosomal protein L23a
G3IAQ1_CRIGR	60S ribosomal protein L7a
G3GS74_CRIGR	Muscleblind-like protein 2
G3HN60_CRIGR	60S ribosomal protein L19
G3GSY1_CRIGR	Sodium/potassium-transporting ATPase subunit beta-3
G3IPR4_CRIGR	Pre-mRNA-processing-splicing factor 8
G3I334_CRIGR	Hydroxyacyl-coenzyme A dehydrogenase, mitochondrial
G3I0N5_CRIGR	Programmed cell death protein 5
G3HLU5_CRIGR	Eukaryotic translation initiation factor 1
G3H5C9_CRIGR	40S ribosomal protein S27
G3HBJ3_CRIGR	Small nuclear ribonucleoprotein F
G3HC18_CRIGR	Interleukin enhancer-binding factor 2
G3H1F2_CRIGR	Ubiquitin-conjugating enzyme E2 E1
G3HZ82_CRIGR	Glycylpeptide N-tetradecanoyltransferase
G3HQ15_CRIGR	S-adenosylmethionine synthase
G3I179_CRIGR	26S proteasome non-ATPase regulatory subunit 14
G3I359_CRIGR	Adenosylhomocysteinase

G3H7R4_CRIGR	Thioredoxin-dependent peroxide reductase, mitochondrial
G3HB69_CRIGR	Cell division protein kinase 5
G3HP80_CRIGR	Vesicle-trafficking protein SEC22b
G3HYW4_CRIGR	Lysosome-associated membrane glycoprotein 2
G3HN76_CRIGR	Polyadenylate-binding protein 4
G3H9W6_CRIGR	Trafficking protein particle complex subunit 3
G3HHC3_CRIGR	Importin subunit alpha
G3HPT1_CRIGR	Cytochrome c oxidase subunit 6B1
G3INC8_CRIGR	Actin-related protein 2
G3HJT6_CRIGR	60S ribosomal protein L32
G3HPV5_CRIGR	Histone H1.5
G3H2J7_CRIGR	Chromobox protein-like 3
G3HRK8_CRIGR	Partner of Y14 and mago
G3GYD5_CRIGR	Complement component 1 Q subcomponent-binding protein, mitochondrial
G3HAN2_CRIGR	Polyadenylate-binding protein 1
G3HEC9_CRIGR	60S ribosomal protein L5
G3HNI3_CRIGR	Uncharacterized protein C14orf45-like
G3I9C4_CRIGR	Cytochrome c oxidase subunit 6B1
G3I3A2_CRIGR	40S ribosomal protein SA
G3HWZ9_CRIGR	Nuclear migration protein nudC
G3HGD7_CRIGR	60S ribosomal protein L23a
G3I8Y4_CRIGR	Nucleosome-binding protein 1
G3HDU9_CRIGR	Histone H1.1
G3I3V3_CRIGR	Coproporphyrinogen-III oxidase, mitochondrial
G3IHF4_CRIGR	Glyceraldehyde-3-phosphate dehydrogenase
G3HKP5_CRIGR	Collagen alpha-1(VII) chain
G3HMU5_CRIGR	Runt-related transcription factor 3
G3I094_CRIGR	60S ribosomal protein L36a
G3I9M2_CRIGR	Aconitate hydratase, mitochondrial
G3HSX8_CRIGR	Biglycan
G3HFK6_CRIGR	60S ribosomal protein L5
G3I3I5_CRIGR	60S ribosomal protein L7a
SRRT_CRIGR	Serrate RNA effector molecule homolog (Fragment)
G3I8N5_CRIGR	V-type proton ATPase subunit E 2
G3HTI6_CRIGR	60S ribosomal protein L7a
G3HDP9_CRIGR	Ubiquitin-conjugating enzyme E2 E3
G3GTY5_CRIGR	60S ribosomal protein L23a
G3HHS0_CRIGR	Zinc finger CCCH domain-containing protein 15
G3HGI4_CRIGR	Hypoxanthine-guanine phosphoribosyltransferase
G3IH19_CRIGR	60S ribosomal protein L36a
G3HT42_CRIGR	Protein FAM59A
G3HH35_CRIGR	60S ribosomal protein L36a
G3HWH8_CRIGR	Transcription factor BTF3
G3HTW4_CRIGR	Cell division protein kinase 3
G3IGC1_CRIGR	60S ribosomal protein L7a
G3IMG8_CRIGR	Eukaryotic translation initiation factor 2 subunit 2
G3IHD5_CRIGR	Alpha-centractin
G3HIJ0_CRIGR	Guanine nucleotide-binding protein G(O) subunit alpha (Fragment)
G3HW76_CRIGR	Peptidyl-prolyl cis-trans isomerase A
G3IMT9_CRIGR	40S ribosomal protein S3a
G3HAK9_CRIGR	60S ribosomal protein L17
G3H133_CRIGR	Translin
A9XHW4_CRIGR	Sjogren syndrome antigen B (Fragment)
G3GX55_CRIGR	Brain-specific angiogenesis inhibitor 1-associated protein 2
G3ID55_CRIGR	Glyceraldehyde-3-phosphate dehydrogenase
G3GSD7_CRIGR	60S ribosomal protein L9
G3I4C8_CRIGR	60S ribosomal protein L18
G3I8N9_CRIGR	Eukaryotic translation initiation factor 1
G3I4R0_CRIGR	60S ribosomal protein L7a
G3H753_CRIGR	Regulator of chromosome condensation
G3IF45_CRIGR	Carbonyl reductase [NADPH] 1
G3GX00_CRIGR	60S ribosomal protein L17
G3IM57_CRIGR	Spermidine synthase
G3I3F0_CRIGR	60S ribosomal protein L32
G3HUQ4_CRIGR	cAMP-dependent protein kinase type I-beta regulatory subunit
G3HTS3_CRIGR	60S ribosomal protein L32
G3HJV0_CRIGR	Protein canopy-like 2
G3HVK3_CRIGR	60S ribosomal protein L23a
G3HH76_CRIGR	40S ribosomal protein S21
G3H6Z3_CRIGR	40S ribosomal protein S2
G3I2B4_CRIGR	Putative uncharacterized protein
G3I5G6_CRIGR	PHD finger-like domain-containing protein 5A
Q9QVA0_CRIGR	Glutathione S-transferase PI (Fragment)
G3HTP6_CRIGR	Alpha-enolase

G3I741_CRIGR	GRB2-associated-binding protein 2
Q4PNS8_CRIGR	Glucosamine-6-phosphate isomerase (Fragment)
G3H1F1_CRIGR	Ubiquitin-conjugating enzyme E2 E1
G3II62_CRIGR	40S ribosomal protein S2
G3IEW6_CRIGR	Ras-related C3 botulinum toxin substrate 2
G3IHP9_CRIGR	60S ribosomal protein L7
Q91Y62_CRIGR	Ezrin binding protein 50 (Fragment)
G3IBA0_CRIGR	ATP-citrate synthase
G3IN60_CRIGR	Septin-1 (Fragment)
G3ICR6_CRIGR	Eukaryotic translation initiation factor 4 gamma 1
G3H4N3_CRIGR	RuvB-like 2
G3HTQ8_CRIGR	F-actin-capping protein subunit beta
G3HRK5_CRIGR	Cell division protein kinase 2
G3HV08_CRIGR	Cell division protein kinase 9

Fed-batch	
Accession	Protein Name
G3HYJ8_CRIGR	10 kDa heat shock protein, mitochondrial
G3HLL4_CRIGR	116 kDa U5 small nuclear ribonucleoprotein component
G3HLS2_CRIGR	14-3-3 protein beta/alpha
G3IPU3_CRIGR	14-3-3 protein epsilon
G3HK90_CRIGR	14-3-3 protein eta
G3HA25_CRIGR	14-3-3 protein gamma
G3H132_CRIGR	14-3-3 protein theta
G3IEY9_CRIGR	14-3-3 protein theta
G3HKZ1_CRIGR	14-3-3 protein zeta/delta
G3IFQ8_CRIGR	15 kDa selenoprotein
G3HRJ2_CRIGR	182 kDa tankyrase-1-binding protein
G3HBG5_CRIGR	26S protease regulatory subunit 4
G3HNP6_CRIGR	26S protease regulatory subunit 6B (Fragment)
G3HE31_CRIGR	26S protease regulatory subunit 7
G3HCU8_CRIGR	26S protease regulatory subunit 8
G3IM40_CRIGR	26S proteasome non-ATPase regulatory subunit 1
G3HCS4_CRIGR	26S proteasome non-ATPase regulatory subunit 12
G3I310_CRIGR	26S proteasome non-ATPase regulatory subunit 2
G3IPV6_CRIGR	26S proteasome non-ATPase regulatory subunit 3
G3ILS7_CRIGR	26S proteasome non-ATPase regulatory subunit 5
G3HIS0_CRIGR	26S proteasome non-ATPase regulatory subunit 6
G3H256_CRIGR	26S proteasome non-ATPase regulatory subunit 9
G3I2S4_CRIGR	3,2-trans-enoyl-CoA isomerase, mitochondrial
G3I2T9_CRIGR	3-phosphoinositide-dependent protein kinase 1
G3I004_CRIGR	40S ribosomal protein S11
G3I737_CRIGR	40S ribosomal protein S12
RS13_CRIGR	40S ribosomal protein S13
G3H011_CRIGR	40S ribosomal protein S15a
G3H1U3_CRIGR	40S ribosomal protein S15a
G3IKE2_CRIGR	40S ribosomal protein S15a
G3HJD8_CRIGR	40S ribosomal protein S16
G3I5Y2_CRIGR	40S ribosomal protein S16
RS17_CRIGR	40S ribosomal protein S17
G3H018_CRIGR	40S ribosomal protein S18
G3H7L4_CRIGR	40S ribosomal protein S18
G3I6W8_CRIGR	40S ribosomal protein S18
G3HBP8_CRIGR	40S ribosomal protein S2
G3HCB4_CRIGR	40S ribosomal protein S2
G3HIC1_CRIGR	40S ribosomal protein S2
G3HP90_CRIGR	40S ribosomal protein S2
G3HUJ1_CRIGR	40S ribosomal protein S2
G3HYV9_CRIGR	40S ribosomal protein S2
G3I7G4_CRIGR	40S ribosomal protein S2
G3I2D3_CRIGR	40S ribosomal protein S20
G3HGW7_CRIGR	40S ribosomal protein S21
G3HH76_CRIGR	40S ribosomal protein S21
G3HIY3_CRIGR	40S ribosomal protein S23
G3HTC5_CRIGR	40S ribosomal protein S23
G3HHA3_CRIGR	40S ribosomal protein S24
G3I583_CRIGR	40S ribosomal protein S26
G3HC03_CRIGR	40S ribosomal protein S27
G3I5R2_CRIGR	40S ribosomal protein S28
G3IDD9_CRIGR	40S ribosomal protein S3
G3HKG8_CRIGR	40S ribosomal protein S3a
G3HMW2_CRIGR	40S ribosomal protein S4
G3HPI0_CRIGR	40S ribosomal protein S4

G3HUW8_CRIGR	40S ribosomal protein S4
G3I0H2_CRIGR	40S ribosomal protein S4
G3IF49_CRIGR	40S ribosomal protein S4
G3H0Q0_CRIGR	40S ribosomal protein S6
G3HAB4_CRIGR	40S ribosomal protein S6
G3IMV1_CRIGR	40S ribosomal protein S6
G3GYR9_CRIGR	40S ribosomal protein S8
G3I351_CRIGR	40S ribosomal protein S9
G3H215_CRIGR	40S ribosomal protein SA
G3HQX0_CRIGR	40S ribosomal protein SA
RSSA_CRIGR	40S ribosomal protein SA
G3I412_CRIGR	45 kDa calcium-binding protein
G3IHN6_CRIGR	4F2 cell-surface antigen heavy chain
G3GR86_CRIGR	6-phosphofructokinase
G3H8W5_CRIGR	6-phosphofructokinase
G3IDJ8_CRIGR	6-phosphofructokinase
G3IHY5_CRIGR	6-phosphogluconate dehydrogenase, decarboxylating
G3HUN3_CRIGR	60 kDa SS-A/Ro ribonucleoprotein
CH60_CRIGR	60 kDa heat shock protein, mitochondrial
G3GU76_CRIGR	60S acidic ribosomal protein P0
G3HDY7_CRIGR	60S acidic ribosomal protein P0
G3HKG9_CRIGR	60S acidic ribosomal protein P0
G3I3H2_CRIGR	60S acidic ribosomal protein P2
G3HMV2_CRIGR	60S ribosomal protein L11
G3GTK2_CRIGR	60S ribosomal protein L12
G3GVM6_CRIGR	60S ribosomal protein L12
G3HV18_CRIGR	60S ribosomal protein L12
G3HZW3_CRIGR	60S ribosomal protein L18
G3H4C1_CRIGR	60S ribosomal protein L18a
G3H5W4_CRIGR	60S ribosomal protein L23
G3HGD7_CRIGR	60S ribosomal protein L23a
G3IGI0_CRIGR	60S ribosomal protein L23a
G3HSH4_CRIGR	60S ribosomal protein L24
G3GY47_CRIGR	60S ribosomal protein L26
G3HM00_CRIGR	60S ribosomal protein L27
G3HSB4_CRIGR	60S ribosomal protein L27
G3I3W6_CRIGR	60S ribosomal protein L27a
G3I2I9_CRIGR	60S ribosomal protein L3
G3IM94_CRIGR	60S ribosomal protein L30
G3H7J9_CRIGR	60S ribosomal protein L31
G3H3Y8_CRIGR	60S ribosomal protein L32
G3IM75_CRIGR	60S ribosomal protein L32
G3H8N3_CRIGR	60S ribosomal protein L4
G3HNJ6_CRIGR	60S ribosomal protein L5
G3HVH8_CRIGR	60S ribosomal protein L5
G3HH97_CRIGR	60S ribosomal protein L5-A
G3GU30_CRIGR	60S ribosomal protein L6
G3H278_CRIGR	60S ribosomal protein L6
G3I8T9_CRIGR	60S ribosomal protein L6
G3GZ17_CRIGR	60S ribosomal protein L7
G3HSC9_CRIGR	60S ribosomal protein L7
G3H9A3_CRIGR	60S ribosomal protein L7a
G3HTQ7_CRIGR	60S ribosomal protein L7a
G3I3I5_CRIGR	60S ribosomal protein L7a
G3I9S3_CRIGR	60S ribosomal protein L7a
G3ILP4_CRIGR	60S ribosomal protein L9
G3I8R9_CRIGR	78 kDa glucose-regulated protein
G3I3J3_CRIGR	A disintegrin and metalloproteinase with thrombospondin motifs 1
G3HU18_CRIGR	A disintegrin and metalloproteinase with thrombospondin motifs 4
G3GU12_CRIGR	A-kinase anchor protein 12
G3I358_CRIGR	A-kinase anchor protein 13
G3GWS7_CRIGR	A-kinase anchor protein 8-like
G3IF83_CRIGR	A-kinase anchor protein SPHKAP
G3HEJ5_CRIGR	ADP-dependent glucokinase
G3I6J4_CRIGR	ADP-ribosylation factor 3
G3GUS2_CRIGR	ADP-ribosylation factor 4
G3GZ81_CRIGR	ADP-ribosylation factor 5
G3IMU0_CRIGR	ADP-ribosylation factor 6
G3I757_CRIGR	ADP-ribosylation factor-like protein 3
G3H2C2_CRIGR	AF4/FMR2 family member 4
G3I798_CRIGR	AMP deaminase 2
G3HP08_CRIGR	AP-1 complex subunit beta-1
G3HAG8_CRIGR	AP-1 complex subunit gamma-1
G3I710_CRIGR	AP-2 complex subunit alpha-1

G3I3I0_CRIGR	AP-2 complex subunit alpha-2
G3I8B9_CRIGR	AP-2 complex subunit beta
G3I319_CRIGR	AP-2 complex subunit mu-1
G3H316_CRIGR	AP-3 complex subunit beta-1
G3HFA8_CRIGR	AP-3 complex subunit delta
G3HRN0_CRIGR	ATP synthase subunit beta
G3GTZ6_CRIGR	ATP-binding cassette sub-family A member 1
G3H9G4_CRIGR	ATP-binding cassette sub-family E member 1
G3HLV6_CRIGR	ATP-citrate synthase
G3HFI6_CRIGR	ATP-dependent RNA helicase DDX1
G3IG25_CRIGR	ATP-dependent RNA helicase DDX19A
G3HD18_CRIGR	ATP-dependent RNA helicase DDX39
G3GSH5_CRIGR	ATP-dependent RNA helicase DDX3X
G3HCW3_CRIGR	ATPase Asna1
G3HT41_CRIGR	Abl interactor 1
G3HGP4_CRIGR	Acetyl-CoA acetyltransferase, cytosolic
G3I0C8_CRIGR	Acetyl-CoA acetyltransferase, mitochondrial
M5AJ86_CRIGR	Acetyl-CoA carboxylase 1
G3HI85_CRIGR	Acetyl-CoA carboxylase 2
G3GZB2_CRIGR	Acid ceramidase
G3H8Q4_CRIGR	Acidic leucine-rich nuclear phosphoprotein 32 family member A
G3H7F8_CRIGR	Acidic leucine-rich nuclear phosphoprotein 32 family member B (Fragment)
G3GUD8_CRIGR	Acidic leucine-rich nuclear phosphoprotein 32 family member B
G3I9M2_CRIGR	Aconitate hydratase, mitochondrial
G3II47_CRIGR	Aconitate hydratase, mitochondrial
G3H5Q0_CRIGR	Actin, alpha cardiac muscle 1
G3GX23_CRIGR	Actin, alpha skeletal muscle
G3HQY2_CRIGR	Actin, aortic smooth muscle
ACTB_CRIGR	Actin, cytoplasmic 1
G3I6E6_CRIGR	Actin, cytoplasmic 2
G3I4A0_CRIGR	Actin, gamma-enteric smooth muscle (Fragment)
G3I1D2_CRIGR	Actin-like protein 6A
G3I390_CRIGR	Actin-related protein 2/3 complex subunit 1B
G3IEK1_CRIGR	Actin-related protein 2/3 complex subunit 2
G3GX96_CRIGR	Actin-related protein 2/3 complex subunit 4
G3GZE6_CRIGR	Actin-related protein 3
G3HIQ3_CRIGR	Activated RNA polymerase II transcriptional coactivator p15
G3H638_CRIGR	Activator of 90 kDa heat shock protein ATPase-like 1
G3H5D5_CRIGR	Acyl-CoA-binding protein
G3HNG3_CRIGR	Acyl-coenzyme A thioesterase 1
G3HNG2_CRIGR	Acyl-coenzyme A thioesterase 2, mitochondrial
G3HF78_CRIGR	Adenomatous polyposis coli protein 2
G3HF07_CRIGR	Adenomatous polyposis coli protein
G3HLR7_CRIGR	Adenosine deaminase
Q925U5_CRIGR	Adenosine kinase (Fragment)
G3HAN8_CRIGR	Adenosylhomocysteinase
G3IHR8_CRIGR	Adenosylhomocysteinase
G3H928_CRIGR	Adenylate kinase 2, mitochondrial
G3IPK9_CRIGR	Adenylate kinase 2, mitochondrial
D0UZH8_CRIGR	Adenylosuccinate lyase
G3HN88_CRIGR	Adenylyl cyclase-associated protein
G3I437_CRIGR	Agrin
G3IG23_CRIGR	Alanyl-tRNA synthetase, cytoplasmic
AK1A1_CRIGR	Alcohol dehydrogenase [NADP(+)] (Fragment)
G3GYP7_CRIGR	Alcohol dehydrogenase [NADP+]
G3IMD1_CRIGR	Alcohol dehydrogenase class-3
G3I000_CRIGR	Aldehyde dehydrogenase family 16 member A1
M1ZMN4_CRIGR	Aldehyde oxidase 3L1
G3HUL2_CRIGR	Aldehyde oxidase
G3IE22_CRIGR	Aldo-keto reductase family 1 member B10
G3HSZ6_CRIGR	Aldose 1-epimerase
G3HH30_CRIGR	Aldose reductase
ALD2_CRIGR	Aldose reductase-related protein 2
MGT5A_CRIGR	Alpha-1,6-mannosylglycoprotein 6-beta-N-acetylglucosaminyltransferase A
G3GWB3_CRIGR	Alpha-2-macroglobulin receptor-associated protein
G3HEE8_CRIGR	Alpha-L-iduronidase
G3I0F7_CRIGR	Alpha-N-acetylgalactosaminidase
G3HLX3_CRIGR	Alpha-N-acetylglucosaminidase
G3H1K9_CRIGR	Alpha-actinin-1
G3I4K1_CRIGR	Alpha-actinin-3
G3HJB0_CRIGR	Alpha-actinin-4 (Fragment)
G3HJB1_CRIGR	Alpha-actinin-4
G3HXW9_CRIGR	Alpha-centractin
G3GWV3_CRIGR	Alpha-enolase

G3IAQ0_CRIGR	Alpha-enolase
G3H604_CRIGR	Alpha-galactosidase A
G3H559_CRIGR	Alpha-mannosidase 2
G3H0F1_CRIGR	Alpha-mannosidase 2C1
G3IG18_CRIGR	Alpha-mannosidase 2x
G3HB74_CRIGR	Amiloride-sensitive amine oxidase [copper-containing]
AIMP1_CRIGR	Aminoacyl tRNA synthase complex-interacting multifunctional protein 1
AIMP2_CRIGR	Aminoacyl tRNA synthase complex-interacting multifunctional protein 2
G3H3H9_CRIGR	Aminopeptidase B
G3HMG4_CRIGR	Amyloid beta A4 protein
G3GTX5_CRIGR	Amyloid-like protein 2
G3HKF3_CRIGR	Ankycorbin
G3IF65_CRIGR	Ankyrin-2
G3H576_CRIGR	Annexin
G3HHA9_CRIGR	Annexin
G3HXL6_CRIGR	Annexin
G3I5A4_CRIGR	Annexin
G3I5L3_CRIGR	Annexin
G3IG05_CRIGR	Annexin
G3IFZ0_CRIGR	Antigen KI-67
C6H0P7_CRIGR	Antrax toxin receptor 2 (Fragment)
G3IK15_CRIGR	Apolipoprotein B-100 receptor
G3IH34_CRIGR	Apoptosis inhibitor 5
G3I7D8_CRIGR	Apoptosis-associated speck-like protein containing a CARD
G3H3X1_CRIGR	Apoptosis-inducing factor 1, mitochondrial
G3HRE2_CRIGR	Apoptotic chromatin condensation inducer in the nucleus
G3GSJ5_CRIGR	Arf-GAP with coiled-coil, ANK repeat and PH domain-containing protein 2
G3HQA3_CRIGR	Arfaptin-1
G3IH84_CRIGR	Arylsulfatase A
ASNS_CRIGR	Asparagine synthetase [glutamine-hydrolyzing]
G3GV42_CRIGR	Asparaginyl-tRNA synthetase, cytoplasmic
G3GZZ0_CRIGR	Aspartate aminotransferase
G3I2Y1_CRIGR	Aspartate aminotransferase
G3I620_CRIGR	Aspartyl aminopeptidase
G3GZR1_CRIGR	Aspartyl-tRNA synthetase, cytoplasmic
G3ILI0_CRIGR	Aspartyl-tRNA synthetase, cytoplasmic
G3IHM2_CRIGR	Atrial natriuretic factor
G3ICC2_CRIGR	BAG family molecular chaperone regulator 3
G3HPL6_CRIGR	BAT2 domain-containing protein 1
G3HVD5_CRIGR	BRISC complex subunit Abro1
G3HEV6_CRIGR	Baculoviral IAP repeat-containing protein 6
G3I2D8_CRIGR	Band 4.1-like protein 2
G3I2F5_CRIGR	Barrier-to-autointegration factor
G3HIM1_CRIGR	Basement membrane-specific heparan sulfate proteoglycan core protein
BASI_CRIGR	Basigin (Fragment)
G3HF38_CRIGR	Basigin
G3H0N2_CRIGR	Bcl-2-associated transcription factor 1
Q60455_CRIGR	Beta tubulin
Q60454_CRIGR	Beta tubulin
G3I413_CRIGR	Beta-1,3-galactosyltransferase 6
Q99PC2_CRIGR	Beta-1,4-galactosyltransferase 1
G3HET2_CRIGR	Beta-1,4-galactosyltransferase 5
Q80WN6_CRIGR	Beta-1,4-galactosyltransferase 5
B2MG_CRIGR	Beta-2-microglobulin
G3ID82_CRIGR	Beta-actin-like protein 2
G3H1Q6_CRIGR	Beta-centractin
G3H2P3_CRIGR	Beta-galactosidase (Fragment)
G3IDU7_CRIGR	Beta-glucuronidase
G3H3P8_CRIGR	Beta-hexosaminidase
G3HXN7_CRIGR	Beta-hexosaminidase
G3HXN5_CRIGR	Beta-hexosaminidase subunit beta
G3HS35_CRIGR	Beta-type platelet-derived growth factor receptor
G3II70_CRIGR	Bifunctional ATP-dependent dihydroxyacetone kinase/FAD-AMP lyase (Cyclizing)
G3GRE1_CRIGR	Bifunctional aminoacyl-tRNA synthetase (Fragment)
G3I4A7_CRIGR	Bifunctional methylenetetrahydrofolate dehydrogenase/cyclohydrolase, mitochondrial
G3I1V4_CRIGR	Bifunctional purine biosynthesis protein PURH
G3HR96_CRIGR	Bleomycin hydrolase
BST2_CRIGR	Bone marrow stromal antigen 2
G3H4E0_CRIGR	Bone marrow stromal antigen 2
G3GUX9_CRIGR	Bone morphogenetic protein 1
G3H3D3_CRIGR	Branched-chain-amino-acid aminotransferase
G3H4Z5_CRIGR	C-1-tetrahydrofolate synthase, cytoplasmic
G3GTT2_CRIGR	C-C motif chemokine 2
G3GTT3_CRIGR	C-C motif chemokine 7

A4URF0_CRIGR	C-X-C motif chemokine 3
G3HBD6_CRIGR	C-jun-amino-terminal kinase-interacting protein 4
G3GXT2_CRIGR	CAD protein
G3H268_CRIGR	CAP-Gly domain-containing linker protein 1
G3HH37_CRIGR	CD109 antigen
G3HRF8_CRIGR	CD166 antigen
CD44_CRIGR	CD44 antigen
G3GYZ1_CRIGR	CD44 antigen
G3HRL6_CRIGR	CD63 antigen
G3HXZ3_CRIGR	CD81 antigen
G3HFJ0_CRIGR	CMP-N-acetylneuraminate-beta-galactosamide-alpha-2, 3-sialyltransferase
G3INL9_CRIGR	CMP-N-acetylneuraminate-beta-galactosamide-alpha-2, 3-sialyltransferase
G3GXD5_CRIGR	COP9 signalosome complex subunit 1
G3HHP3_CRIGR	COP9 signalosome complex subunit 3
G3IH48_CRIGR	COP9 signalosome complex subunit 7b
G3HCN2_CRIGR	CTP synthase
G3H919_CRIGR	CUB and sushi domain-containing protein 2 (Fragment)
Q7M0H9_CRIGR	Ca2+/calmodulin-dependent protein kinase ERK1 (Fragment)
G3I1Y8_CRIGR	Cadherin-23
G3HNW2_CRIGR	Cadherin-9
G3HRJ5_CRIGR	Calcitonin gene-related peptide type 1 receptor
G3IP10_CRIGR	Calcium-binding and coiled-coil domain-containing protein 1 (Fragment)
G3GUR0_CRIGR	Calcium-dependent serine proteinase
G3GZL7_CRIGR	Calcyclin-binding protein
G3I5T9_CRIGR	Calmodulin (Fragment)
G3HBG8_CRIGR	Calmodulin
G3I9A7_CRIGR	Calmodulin
G3IM13_CRIGR	Calmodulin
G3ILW1_CRIGR	Calpain-1 catalytic subunit
G3H750_CRIGR	Calpastatin
G3HF56_CRIGR	Calponin-2
CALR_CRIGR	Calreticulin
G3HCX8_CRIGR	Calreticulin
G3ILK7_CRIGR	Calsyntenin-1
G3GZ90_CRIGR	Calumenin
G3HQL4_CRIGR	Carbohydrate sulfotransferase 11
Q924V2_CRIGR	Carbonyl reductase 2
Q9JJN7_CRIGR	Carbonyl reductase
G3IF39_CRIGR	Carbonyl reductase [NADPH] 1
G3IF40_CRIGR	Carbonyl reductase [NADPH] 1
G3IF44_CRIGR	Carbonyl reductase [NADPH] 1
G3HR95_CRIGR	Carboxypeptidase D
C4PFX6_CRIGR	Caspase 3
Q6JH80_CRIGR	Caspase 3
G3GYY6_CRIGR	Catalase
G3H0L9_CRIGR	Cathepsin B
G3I4W7_CRIGR	Cathepsin D
G3I4K2_CRIGR	Cathepsin F
G3INC5_CRIGR	Cathepsin L1
Q9EPP7_CRIGR	Cathepsin Z
G3HGQ1_CRIGR	Cation-independent mannose-6-phosphate receptor
G3HCL8_CRIGR	Cdc42-interacting protein 4
G3HVL1_CRIGR	Cell division control protein 2-like
G3HIM4_CRIGR	Cell division control protein 42-like
G3GWE6_CRIGR	Cell growth-regulating nucleolar protein
G3HI29_CRIGR	Ceroid-lipofuscinosis neuronal protein 5
G3HAN1_CRIGR	Charged multivesicular body protein 4b
G3I3H9_CRIGR	Chitinase domain-containing protein 1
G3HZF4_CRIGR	Chloride intracellular channel protein 1
G3HMU4_CRIGR	Chloride intracellular channel protein 4
G3H0E4_CRIGR	Chondroitin sulfate proteoglycan 4
G3I2J3_CRIGR	Chromobox protein-like 6 (Fragment)
G3GSE1_CRIGR	Chromodomain-helicase-DNA-binding protein 2
Q4F888_CRIGR	Class V beta tubulin
G3HJ89_CRIGR	Clathrin heavy chain
G3H194_CRIGR	Clathrin light chain B (Fragment)
G3GYX4_CRIGR	Cleavage stimulation factor 77 kDa subunit
G3HNJ3_CRIGR	Clusterin
G3GRX4_CRIGR	Coactosin-like protein
G3H7Y0_CRIGR	Coatomer subunit alpha
G3HSE5_CRIGR	Coatomer subunit beta
G3GSW3_CRIGR	Coatomer subunit beta'
G3I5S7_CRIGR	Coatomer subunit delta
COPE_CRIGR	Coatomer subunit epsilon

G3GZ72_CRIGR	Coatomer subunit gamma
G3HID3_CRIGR	Coatomer subunit gamma
G3IDM2_CRIGR	Cofilin-1
G3HAJ9_CRIGR	Coiled-coil domain-containing protein 58
G3H092_CRIGR	Coiled-coil domain-containing protein 80
G3IM20_CRIGR	Collagen alpha-1(III) chain
G3I7K9_CRIGR	Collagen alpha-1(IV) chain
G3IL75_CRIGR	Collagen alpha-1(V) chain
G3H8Y5_CRIGR	Collagen alpha-1(VI) chain
G3HH34_CRIGR	Collagen alpha-1(XII) chain (Fragment)
G3H1W1_CRIGR	Collagen alpha-1(XVI) chain
G3IKH3_CRIGR	Collagen alpha-2(V) chain
G3IKH4_CRIGR	Collagen alpha-2(V) chain
G3H6E6_CRIGR	Collagen alpha-3(V) chain (Fragment)
G3GZJ0_CRIGR	Collagen alpha-5(VI) chain
G3IID2_CRIGR	Complement C1q tumor necrosis factor-related protein 5
G3GUR3_CRIGR	Complement C1r subcomponent-like protein
G3GUR1_CRIGR	Complement C1r-A subcomponent
G3HCL6_CRIGR	Complement C3
G3I3T4_CRIGR	Complement component C1q receptor
G3HHG0_CRIGR	Complement component C6
G3HHG3_CRIGR	Complement component C7
G3IAP3_CRIGR	Connective tissue growth factor
G3I7E1_CRIGR	Contactin-1
G3I9G3_CRIGR	Copine-3
G3H585_CRIGR	Copper transport protein ATOX1
G3IIK9_CRIGR	Cornifin-A
G3HI96_CRIGR	Coronin
G3HS70_CRIGR	Coronin
G3I3Z5_CRIGR	Coronin
G3I4P0_CRIGR	Craniofacial development protein 1
G3GXG0_CRIGR	Cullin-1
G3HT32_CRIGR	Cullin-2
G3ICS3_CRIGR	Cullin-3
G3HYW3_CRIGR	Cullin-4B (Fragment)
G3I0C6_CRIGR	Cullin-5
G3GY17_CRIGR	Cullin-associated NEDD8-dissociated protein 1
G3H3I9_CRIGR	Cysteine and glycine-rich protein 1
G3HUV6_CRIGR	Cysteine-rich motor neuron 1 protein
G3H3B2_CRIGR	Cysteine-rich protein 2
CREL2_CRIGR	Cysteine-rich with EGF-like domain protein 2
G3IC16_CRIGR	Cysteine-rich with EGF-like domain protein 2
G3IIT6_CRIGR	Cysteinyl-tRNA synthetase, cytoplasmic
G3H2K2_CRIGR	Cytochrome c, somatic
G3HT77_CRIGR	Cytoplasmic FMR1-interacting protein 1
G3HQZ8_CRIGR	Cytoplasmic aconitate hydratase
G3H352_CRIGR	Cytoplasmic dynein 1 heavy chain 1 (Fragment)
G3H656_CRIGR	Cytoplasmic dynein 1 light intermediate chain 2
G3GUV0_CRIGR	Cytoplasmic dynein 2 heavy chain 1
G3GYJ5_CRIGR	Cytoskeleton-associated protein 5
G3HR08_CRIGR	Cytosol aminopeptidase
G3IJV7_CRIGR	Cytosolic acyl coenzyme A thioester hydrolase
G3I692_CRIGR	Cytosolic non-specific dipeptidase
G3IGI6_CRIGR	Cytosolic purine 5'-nucleotidase
G3HP75_CRIGR	D-3-phosphoglycerate dehydrogenase
G3HY03_CRIGR	D-dopachrome decarboxylase
G3II69_CRIGR	DNA damage-binding protein 1
G3I1H0_CRIGR	DNA replication licensing factor MCM3
G3HWN6_CRIGR	DNA topoisomerase 1 (Fragment)
TOP1_CRIGR	DNA topoisomerase 1
TOP2A_CRIGR	DNA topoisomerase 2-alpha
Q76M93_CRIGR	DNA-directed RNA polymerase
G3HJG6_CRIGR	Decorin
G3HHZ9_CRIGR	Delta-aminolevulinic acid dehydratase
G3HUQ9_CRIGR	Dentin matrix protein 4
G3HCX3_CRIGR	Deoxyribonuclease-2-alpha
G3I621_CRIGR	Desmin
G3GVX2_CRIGR	Destrin
G3HMI5_CRIGR	Di-N-acetylchitobiase
G3HKN5_CRIGR	Diacylglycerol kinase
G3HWE7_CRIGR	Dickkopf-related protein 3
Q2MH30_CRIGR	Dihydrofolate reductase
G3HR10_CRIGR	Dihydropteridine reductase
G3HNK4_CRIGR	Dihydropyrimidinase-related protein 2

G3IN86_CRIGR	Dipeptidyl-peptidase 2
G3I2H0_CRIGR	Dipeptidyl-peptidase 3
G3HQK4_CRIGR	Discoidin domain-containing receptor 2
G3I3V6_CRIGR	Discoidin, CUB and LCCL domain-containing protein 2
Q99P57_CRIGR	Discoidin-1 domain receptor-2 (Fragment)
G3GRM2_CRIGR	Disintegrin and metalloproteinase domain-containing protein 10
G3HC59_CRIGR	Disintegrin and metalloproteinase domain-containing protein 15
G3HRX4_CRIGR	Disintegrin and metalloproteinase domain-containing protein 22
G3H1K6_CRIGR	Disintegrin and metalloproteinase domain-containing protein 9
G3GSJ8_CRIGR	Disks large-like 1
G3HYG7_CRIGR	DnaJ-like subfamily B member 11
G3H0N9_CRIGR	DnaJ-like subfamily B member 9
G3H8H7_CRIGR	DnaJ-like subfamily C member 10
G3GS78_CRIGR	DnaJ-like subfamily C member 3
G3H1Z4_CRIGR	DnaJ-like subfamily C member 8
G3I2P6_CRIGR	DnaJ-like subfamily C member 9
Q7M080_CRIGR	DnaK-type molecular chaperone (Fragments)
G3GXX3_CRIGR	Dolichyl-diphosphooligosaccharide--protein glycosyltransferase subunit 2
G3H170_CRIGR	Drebrin
G3HM98_CRIGR	Drebrin-like protein
G3I7W3_CRIGR	Dynactin subunit 1
G3HW48_CRIGR	Dynactin subunit 2
G3H6B2_CRIGR	Dynamin
G3IBJ1_CRIGR	Dynamin-1
G3IEY5_CRIGR	Dynamin-1-like protein
G3HWY6_CRIGR	Dynamin-like 120 kDa protein, mitochondrial
G3H1E5_CRIGR	Dynein light chain 2, cytoplasmic
G3H8F4_CRIGR	Dystroglycan
G3HJ15_CRIGR	E3 SUMO-protein ligase RanBP2
G3I905_CRIGR	E3 ubiquitin-protein ligase UBR4
G3I952_CRIGR	EF-hand domain-containing protein D2
G3I9L7_CRIGR	EGF-containing fibulin-like extracellular matrix protein 1
FBLN4_CRIGR	EGF-containing fibulin-like extracellular matrix protein 2
G3I2G7_CRIGR	EGF-containing fibulin-like extracellular matrix protein 2
G3I6F9_CRIGR	EH domain-containing protein 1
G3I4G0_CRIGR	EH domain-containing protein 3
G3H0Y9_CRIGR	EH domain-containing protein 4
G3HRR5_CRIGR	ELAV-like protein 1
G3H873_CRIGR	ELKS/RAB6-interacting/CAST family member 1
G3HPZ6_CRIGR	ELMO domain-containing protein 3
G3GXS2_CRIGR	EMILIN-1
G3I600_CRIGR	Early endosome antigen 1
G3HPE9_CRIGR	Echinoderm microtubule-associated protein-like 2
G3GV30_CRIGR	Echinoderm microtubule-associated protein-like 4 (Fragment)
EF1A1_CRIGR	Elongation factor 1-alpha 1
G3HH39_CRIGR	Elongation factor 1-alpha 1
G3IHP6_CRIGR	Elongation factor 1-alpha 1
G3HGY8_CRIGR	Elongation factor 1-alpha
G3HWX4_CRIGR	Elongation factor 1-alpha
G3HDL6_CRIGR	Elongation factor 1-beta
G3I5H3_CRIGR	Elongation factor 1-delta
G3HBJ5_CRIGR	Elongation factor 1-gamma
G3I072_CRIGR	Elongation factor 1-gamma
G3I073_CRIGR	Elongation factor 1-gamma
G3IE48_CRIGR	Elongation factor 1-gamma
EF2_CRIGR	Elongation factor 2
G3HSL4_CRIGR	Elongation factor 2
G3H748_CRIGR	Endoplasmic reticulum aminopeptidase 1
G3IN87_CRIGR	Endoplasmic reticulum mannosyl-oligosaccharide 1,2-alpha-mannosidase
G3H284_CRIGR	Endoplasmic reticulum resident protein 29
G3HRG8_CRIGR	Endoplasmic reticulum resident protein ERp44 (Fragment)
G3HQM6_CRIGR	Endoplasmin
G3GTX7_CRIGR	Enolase
G3GWV2_CRIGR	Enolase
G3I9N7_CRIGR	Enolase
G3H355_CRIGR	Envelope glycoprotein
G3I863_CRIGR	Ephrin type-A receptor 2
G3HC56_CRIGR	Ephrin-A1
G3H4H4_CRIGR	Epidermal growth factor receptor substrate 15-like 1
G3I936_CRIGR	Epididymal secretory protein E1
G3I1R6_CRIGR	Epididymal secretory protein E3-beta
G3HWJ4_CRIGR	Epiplakin
G3GW53_CRIGR	Epithelial cell transforming sequence 2 oncogene-like
G3GY75_CRIGR	Eukaryotic initiation factor 4A-I

G3HYG2_CRIGR	Eukaryotic initiation factor 4A-II
G3HQ40_CRIGR	Eukaryotic peptide chain release factor GTP-binding subunit ERF3B
G3HEZ1_CRIGR	Eukaryotic peptide chain release factor subunit 1
MCA3_CRIGR	Eukaryotic translation elongation factor 1 epsilon-1
G3H1M4_CRIGR	Eukaryotic translation initiation factor 2 subunit 1
G3HQ45_CRIGR	Eukaryotic translation initiation factor 2 subunit 3
G3HG40_CRIGR	Eukaryotic translation initiation factor 2A
G3IK13_CRIGR	Eukaryotic translation initiation factor 3 subunit C
G3IJ29_CRIGR	Eukaryotic translation initiation factor 3 subunit D
G3I638_CRIGR	Eukaryotic translation initiation factor 3 subunit F
G3H6E1_CRIGR	Eukaryotic translation initiation factor 3 subunit G
G3H945_CRIGR	Eukaryotic translation initiation factor 3 subunit I
G3H0T1_CRIGR	Eukaryotic translation initiation factor 3 subunit J
G3I505_CRIGR	Eukaryotic translation initiation factor 3 subunit L
G3GZB1_CRIGR	Eukaryotic translation initiation factor 3 subunit M
G3I306_CRIGR	Eukaryotic translation initiation factor 4 gamma 1
G3IH96_CRIGR	Eukaryotic translation initiation factor 4B
G3IAB9_CRIGR	Eukaryotic translation initiation factor 4E
G3IIH3_CRIGR	Eukaryotic translation initiation factor 4E
G3GY95_CRIGR	Eukaryotic translation initiation factor 5A-1
G3I948_CRIGR	Eukaryotic translation initiation factor 5A-2
G3HHL3_CRIGR	Eukaryotic translation initiation factor 5B
G3HAQ2_CRIGR	Eukaryotic translation initiation factor 6
G3H5A9_CRIGR	Exosome complex exonuclease RRP44
G3ID62_CRIGR	Exostosin-like 2
G3HES3_CRIGR	Exportin-2
G3HYU7_CRIGR	Exportin-5
G3H9J8_CRIGR	Exportin-T
G3H2W6_CRIGR	Extracellular matrix protein 1
G3HWC3_CRIGR	Ezrin
G3IDS2_CRIGR	F-actin-capping protein subunit alpha-1
G3HU28_CRIGR	F-actin-capping protein subunit beta
G3HU71_CRIGR	F-box only protein 43
G3HWP7_CRIGR	FACT complex subunit SPT16
G3HRJ1_CRIGR	FACT complex subunit SSRP1
G3HLV1_CRIGR	FK506-binding protein 10
G3GUI3_CRIGR	FK506-binding protein 4
G3HMQ0_CRIGR	FK506-binding protein 9
G3HCK9_CRIGR	Far upstream element-binding protein 2
G3HC39_CRIGR	Farnesyl pyrophosphate synthetase
Q8R4V9_CRIGR	Fatty acid synthase (Fragment)
G3GXD7_CRIGR	Fatty acid synthase
G3I6H5_CRIGR	Fermitin family-like 2
G3H5A6_CRIGR	Ferritin
G3HPQ9_CRIGR	Ferritin
FRIH_CRIGR	Ferritin heavy chain
G3HXU8_CRIGR	Fibroblast growth factor
G3I1V3_CRIGR	Fibronectin
G3IBK2_CRIGR	Filamin-A
G3HFM4_CRIGR	Filamin-B
G3GZ94_CRIGR	Filamin-C
G3I7E2_CRIGR	Focal adhesion kinase 1
G3HAI3_CRIGR	Follistatin-related protein 1
G3GYL9_CRIGR	Formin-binding protein 4
G3I3X4_CRIGR	Fructose-bisphosphate aldolase
G3I4H6_CRIGR	Fructose-bisphosphate aldolase
G3IH03_CRIGR	Fructose-bisphosphate aldolase
G3IK44_CRIGR	Fructose-bisphosphate aldolase
G3H6M5_CRIGR	Fumarate hydratase, mitochondrial
G3IHH6_CRIGR	Fumarylacetoacetase
G3I3K5_CRIGR	G-protein coupled receptor 56
FCL_CRIGR	GDP-L-fucose synthase
G3H229_CRIGR	GMP synthase [glutamine-hydrolyzing]
Q9Z1W7_CRIGR	GP50
G3GW97_CRIGR	GPN-loop GTPase 1
G3GXN1_CRIGR	GTP-binding nuclear protein Ran
G3HP89_CRIGR	GTP-binding nuclear protein Ran
G3IHE5_CRIGR	GTP-binding nuclear protein Ran
G3IKJ4_CRIGR	GTP-binding nuclear protein Ran
G3HEB0_CRIGR	GTP-binding protein REM 1
G3HTU8_CRIGR	Galactokinase
G3H7B3_CRIGR	Galectin
LEG1_CRIGR	Galectin-1
G3H3E4_CRIGR	Galectin-3-binding protein

G3GUQ0_CRIGR	Gamma-enolase
G3HQ74_CRIGR	Gamma-glutamyl hydrolase
G3H577_CRIGR	Ganglioside GM2 activator
G3HX84_CRIGR	Gelsolin
G3HCE7_CRIGR	Gephyrin
G3HDD1_CRIGR	Geranylgeranyl transferase type-2 subunit alpha
G3IG52_CRIGR	Glucocorticoid receptor DNA-binding factor 1
G6PD_CRIGR	Glucose-6-phosphate 1-dehydrogenase
G3IBL4_CRIGR	Glucose-6-phosphate 1-dehydrogenase
G6PI_CRIGR	Glucose-6-phosphate isomerase
G3H697_CRIGR	Glucosidase 2 subunit beta
G3HC47_CRIGR	Glucosylceramidase
GLNA_CRIGR	Glutamine synthetase
G3HG36_CRIGR	Glutamine synthetase
G3HLB3_CRIGR	Glutamine synthetase
G3IB71_CRIGR	Glutaminyl-tRNA synthetase
G3IKS9_CRIGR	Glutaredoxin-3
G3I5H1_CRIGR	Glutathione S-transferase A4
G3INF7_CRIGR	Glutathione S-transferase Mu 1
G3ILF2_CRIGR	Glutathione S-transferase Mu 2
G3ILF1_CRIGR	Glutathione S-transferase Mu 5
G3IKC3_CRIGR	Glutathione S-transferase Mu 6
G3ILF3_CRIGR	Glutathione S-transferase Mu 7
G3I3Y6_CRIGR	Glutathione S-transferase P
G3IKN5_CRIGR	Glutathione S-transferase omega-1
G3H8G0_CRIGR	Glutathione peroxidase
G3HF60_CRIGR	Glutathione peroxidase
G3H609_CRIGR	Glutathione reductase, mitochondrial
G3HAP7_CRIGR	Glutathione synthetase
G3P_CRIGR	Glyceraldehyde-3-phosphate dehydrogenase
G3GTJ3_CRIGR	Glyceraldehyde-3-phosphate dehydrogenase
G3GTJ4_CRIGR	Glyceraldehyde-3-phosphate dehydrogenase
G3GZ13_CRIGR	Glyceraldehyde-3-phosphate dehydrogenase
G3GZ97_CRIGR	Glyceraldehyde-3-phosphate dehydrogenase
G3H208_CRIGR	Glyceraldehyde-3-phosphate dehydrogenase
G3H5I3_CRIGR	Glyceraldehyde-3-phosphate dehydrogenase
G3H740_CRIGR	Glyceraldehyde-3-phosphate dehydrogenase
G3H9M6_CRIGR	Glyceraldehyde-3-phosphate dehydrogenase
G3H9M7_CRIGR	Glyceraldehyde-3-phosphate dehydrogenase
G3HJW0_CRIGR	Glyceraldehyde-3-phosphate dehydrogenase
G3HKA3_CRIGR	Glyceraldehyde-3-phosphate dehydrogenase
G3HKK5_CRIGR	Glyceraldehyde-3-phosphate dehydrogenase
G3HKV0_CRIGR	Glyceraldehyde-3-phosphate dehydrogenase
G3HNV7_CRIGR	Glyceraldehyde-3-phosphate dehydrogenase
G3HPT5_CRIGR	Glyceraldehyde-3-phosphate dehydrogenase
G3HXF7_CRIGR	Glyceraldehyde-3-phosphate dehydrogenase
G3HYZ4_CRIGR	Glyceraldehyde-3-phosphate dehydrogenase
G3HZA8_CRIGR	Glyceraldehyde-3-phosphate dehydrogenase
G3I044_CRIGR	Glyceraldehyde-3-phosphate dehydrogenase
G3I1S5_CRIGR	Glyceraldehyde-3-phosphate dehydrogenase
G3I6P1_CRIGR	Glyceraldehyde-3-phosphate dehydrogenase
G3I9H0_CRIGR	Glyceraldehyde-3-phosphate dehydrogenase
G3IHF4_CRIGR	Glyceraldehyde-3-phosphate dehydrogenase
G3IHW0_CRIGR	Glyceraldehyde-3-phosphate dehydrogenase
G3IJG4_CRIGR	Glyceraldehyde-3-phosphate dehydrogenase
G3I0A8_CRIGR	Glycine cleavage system H protein, mitochondrial
G3HZY0_CRIGR	Glycogen [starch] synthase, muscle
G3H4D2_CRIGR	Glycosyltransferase 25 family member 1
G3HJM2_CRIGR	Glycyl-tRNA synthetase
G3HTG9_CRIGR	Glyoxalase domain-containing protein 4
G3H4T5_CRIGR	Glypican-1
GSLG1_CRIGR	Golgi apparatus protein 1
G3IAW1_CRIGR	Golgi membrane protein 1
G3ILT7_CRIGR	Golgi membrane protein 1
G3IB53_CRIGR	Golgi-associated plant pathogenesis-related protein 1
G3I1H4_CRIGR	Golgin subfamily A member 5
G3HAJ0_CRIGR	Golgin subfamily B member 1
G3HLK3_CRIGR	Granulins
G3INY3_CRIGR	Granulocyte colony-stimulating factor
G3HKV9_CRIGR	Group XV phospholipase A2
G3GTE4_CRIGR	Growth factor receptor-bound protein 2
GROA_CRIGR	Growth-regulated alpha protein
G3HZS9_CRIGR	GrpE protein homolog
Q3LRD4_CRIGR	Guanine nucleotide binding protein alpha inhibiting 1

Q3HR13_CRIGR	Guanine nucleotide binding protein alpha inhibiting 2
Q3HR12_CRIGR	Guanine nucleotide binding protein alpha inhibiting 3
G3HMH5_CRIGR	Guanine nucleotide-binding protein G(I), alpha-1 subunit
G3H8D0_CRIGR	Guanine nucleotide-binding protein G(I), alpha-2 subunit
G3I1X8_CRIGR	Guanine nucleotide-binding protein G(I)/G(S)/G(T) subunit beta-1
G3I668_CRIGR	Guanine nucleotide-binding protein G(I)/G(S)/G(T) subunit beta-2
G3HIJ0_CRIGR	Guanine nucleotide-binding protein G(O) subunit alpha (Fragment)
GNAS_CRIGR	Guanine nucleotide-binding protein G(s) subunit alpha
G3HK00_CRIGR	Guanine nucleotide-binding protein subunit beta-2-like 1
G3I1D1_CRIGR	Guanine nucleotide-binding protein subunit beta-4
G3I7T9_CRIGR	Guanine nucleotide-binding protein subunit gamma
G3HQR1_CRIGR	Guanine nucleotide-binding protein-like 1 (Fragment)
G3HQR5_CRIGR	H-2 class I histocompatibility antigen, D-37 alpha chain
G3HQN3_CRIGR	H-2 class I histocompatibility antigen, D-D alpha chain
G3INM4_CRIGR	H-2 class I histocompatibility antigen, L-D alpha chain
G3HS40_CRIGR	H-2 class II histocompatibility antigen gamma chain
G3HV92_CRIGR	H/ACA ribonucleoprotein complex subunit 4
G3IG19_CRIGR	HD domain-containing protein 3
G3H4S3_CRIGR	Heat shock 70 kDa protein 13
G3HZE5_CRIGR	Heat shock 70 kDa protein 1A
G3HZE6_CRIGR	Heat shock 70 kDa protein 1A/1B
G3HZE7_CRIGR	Heat shock 70 kDa protein 1L
G3H2C4_CRIGR	Heat shock 70 kDa protein 4 (Fragment)
HSP7C_CRIGR	Heat shock cognate 71 kDa protein
G3IDL7_CRIGR	Heat shock cognate 71 kDa protein
G3IDL8_CRIGR	Heat shock cognate 71 kDa protein
G3ILE5_CRIGR	Heat shock cognate 71 kDa protein
G3GWF4_CRIGR	Heat shock protein 105 kDa (Fragment)
HS105_CRIGR	Heat shock protein 105 kDa
G3I027_CRIGR	Heat shock protein 75 kDa, mitochondrial
HS90A_CRIGR	Heat shock protein HSP 90-alpha
G3H354_CRIGR	Heat shock protein HSP 90-alpha
G3HS15_CRIGR	Heat shock protein HSP 90-alpha
G3HC84_CRIGR	Heat shock protein HSP 90-beta
G3HA24_CRIGR	Heat shock protein beta-1 (Fragment)
G3H4Z8_CRIGR	Heat shock-related 70 kDa protein 2
G3IAI6_CRIGR	Heme oxygenase 1
H6ST1_CRIGR	Heparan-sulfate 6-O-sulfotransferase 1
G3IA26_CRIGR	Heparanase
G3HPQ5_CRIGR	Hepatocyte growth factor
G3H9I9_CRIGR	Hepatocyte growth factor receptor
G3HG69_CRIGR	Hepatoma-derived growth factor
Q6E6J8_CRIGR	Heterochromatin protein 1 gamma
G3I486_CRIGR	Heterochromatin protein 1-binding protein 3
G3H2E2_CRIGR	Heterogeneous nuclear ribonucleoprotein A/B
G3H1U2_CRIGR	Heterogeneous nuclear ribonucleoprotein A3
G3HRU8_CRIGR	Heterogeneous nuclear ribonucleoprotein A3
G3I783_CRIGR	Heterogeneous nuclear ribonucleoprotein D-like
G3I782_CRIGR	Heterogeneous nuclear ribonucleoprotein D0
G3H6K1_CRIGR	Heterogeneous nuclear ribonucleoprotein G
G3H9H7_CRIGR	Heterogeneous nuclear ribonucleoprotein G
G3HP71_CRIGR	Heterogeneous nuclear ribonucleoprotein H (Fragment)
G3H605_CRIGR	Heterogeneous nuclear ribonucleoprotein H2
G3I5Q4_CRIGR	Heterogeneous nuclear ribonucleoprotein M
G3GTB7_CRIGR	Heterogeneous nuclear ribonucleoprotein Q
G3HIP2_CRIGR	Heterogeneous nuclear ribonucleoprotein R
G3H7M8_CRIGR	Heterogeneous nuclear ribonucleoprotein U
G3IA10_CRIGR	Heterogeneous nuclear ribonucleoprotein U-like protein 1
G3II33_CRIGR	Heterogeneous nuclear ribonucleoprotein U-like protein 2
G3H2J8_CRIGR	Heterogeneous nuclear ribonucleoproteins A2/B1
G3HWP9_CRIGR	Heterogeneous nuclear ribonucleoproteins C1/C2
G4Y367_CRIGR	Hexokinase 1 (Fragment)
G4Y368_CRIGR	Hexokinase 2 (Fragment)
G3H6T5_CRIGR	Hexokinase-1
G3IGL3_CRIGR	Hexokinase-2
G3GWG3_CRIGR	High mobility group protein B1
G3GZL5_CRIGR	High mobility group protein B1
G3HGH1_CRIGR	High mobility group protein B1
G3HI51_CRIGR	High mobility group protein B1
G3HN43_CRIGR	High mobility group protein B1
G3HUE9_CRIGR	High mobility group protein B1
G3HKY0_CRIGR	High mobility group protein B2
G3I595_CRIGR	Histidyl-tRNA synthetase, cytoplasmic
G3HDT8_CRIGR	Histone H1t

G3H4Q1_CRIGR	Histone H2A
G3HDS3_CRIGR	Histone H2A
G3HDT6_CRIGR	Histone H2A
G3HMB9_CRIGR	Histone H2A
G3I968_CRIGR	Histone H2A
G3H2T4_CRIGR	Histone H2B
G3H2U0_CRIGR	Histone H2B
G3HDT0_CRIGR	Histone H2B
G3HDU3_CRIGR	Histone H2B
G3HDV4_CRIGR	Histone H2B
G3HJY9_CRIGR	Histone H2B type 1
G3H2T7_CRIGR	Histone H3
G3HDT2_CRIGR	Histone H3
G3HHM2_CRIGR	Histone H3.1t
G3GSR4_CRIGR	Histone H3.3
G3HV67_CRIGR	Histone H3.3
G3HDS1_CRIGR	Histone H4
G3HDT9_CRIGR	Histone H4
G3HPV7_CRIGR	Histone H4
G3HND2_CRIGR	Histone deacetylase 6
G3HHV8_CRIGR	Histone deacetylase
G3H937_CRIGR	Histone-binding protein RBBP4
G3HKD6_CRIGR	Histone-binding protein RBBP7
G3I539_CRIGR	Hsc70-interacting protein
G3H6C5_CRIGR	Hsp90 co-chaperone Cdc37
G3H0T9_CRIGR	Huntingtin-interacting protein K
G3HBP3_CRIGR	Hydroxyacylglutathione hydrolase, mitochondrial
HMCS1_CRIGR	Hydroxymethylglutaryl-CoA synthase, cytoplasmic
G3HMY0_CRIGR	Hydroxymethylglutaryl-CoA synthase, cytoplasmic
HPRT_CRIGR	Hypoxanthine-guanine phosphoribosyltransferase
HYOU1_CRIGR	Hypoxia up-regulated protein 1
G3I973_CRIGR	Hypoxia up-regulated protein 1
G3HCT1_CRIGR	Importin subunit alpha
G3H5V0_CRIGR	Importin subunit beta-1
G3HDD8_CRIGR	Importin-4
G3GS70_CRIGR	Importin-5
G3I7Z8_CRIGR	Importin-7
G3H3I2_CRIGR	Importin-9
G3GTC6_CRIGR	Inactive serine protease 35
G3IK71_CRIGR	Inner centromere protein
G3H6S8_CRIGR	Inorganic pyrophosphatase
IMDH2_CRIGR	Inosine-5'-monophosphate dehydrogenase 2
G3GZ89_CRIGR	Inosine-5'-monophosphate dehydrogenase
G3I4E6_CRIGR	Inositol monophosphatase
G3H497_CRIGR	Inositol-3-phosphate synthase 1
G3H3W4_CRIGR	Insulin-degrading enzyme
G3I5N6_CRIGR	Insulin-like growth factor-binding protein 4
G3I6C1_CRIGR	Integrin alpha-5
G3HZ55_CRIGR	Integrin beta
G3GR64_CRIGR	Inter-alpha-trypsin inhibitor heavy chain H5
Q9ERF7_CRIGR	Intercellular adhesion molecule 1
G3H6D2_CRIGR	Intercellular adhesion molecule 5
G3GT45_CRIGR	Interferon-alpha/beta receptor beta chain
G3GW63_CRIGR	Interferon-gamma receptor alpha chain
G3IJK4_CRIGR	Interferon-induced protein with tetratricopeptide repeats 3
G3IDL3_CRIGR	Interferon-inducible protein
G3HC17_CRIGR	Interleukin enhancer-binding factor 2
G3H6B5_CRIGR	Interleukin enhancer-binding factor 3
G3GT78_CRIGR	Interleukin-1 receptor accessory protein
G3H6I5_CRIGR	Interleukin-1 receptor-like 1
G3HHX0_CRIGR	Interleukin-6 receptor subunit beta
G3IHH9_CRIGR	Isoamyl acetate-hydrolyzing esterase 1-like
G3HU51_CRIGR	Isocitrate dehydrogenase [NADP]
G3I4U7_CRIGR	Isocitrate dehydrogenase [NADP] cytoplasmic
G3HP24_CRIGR	Isoleucyl-tRNA synthetase, cytoplasmic
G3GR90_CRIGR	Isopentenyl-diphosphate Delta-isomerase 1
G3HFP8_CRIGR	KDEL motif-containing protein 2
G3IPN2_CRIGR	Keratin, type I cytoskeletal 10 (Fragment)
G3I8F6_CRIGR	Keratin, type I cytoskeletal 15
G3IKI6_CRIGR	Keratin, type II cytoskeletal 1b
G3IBP3_CRIGR	Keratin, type II cytoskeletal 2 epidermal
G3IKI7_CRIGR	Keratin, type II cytoskeletal 2 oral
G3IBN9_CRIGR	Keratin, type II cytoskeletal 71
G3H7A8_CRIGR	Kinectin

G3HW49_CRIGR	Kinesin heavy chain isoform 5A
G3I7Y5_CRIGR	Kinesin heavy chain isoform 5C
G3H381_CRIGR	Kinesin light chain 1
G3H3P5_CRIGR	Kinesin-1 heavy chain
G3IAS8_CRIGR	Kinesin-like protein KIF27
G3I075_CRIGR	L-asparaginase
G3H0C9_CRIGR	L-lactate dehydrogenase A chain
G3HGK3_CRIGR	L-lactate dehydrogenase A chain
G3HH14_CRIGR	L-lactate dehydrogenase
G3I255_CRIGR	L-lactate dehydrogenase
G3I9P1_CRIGR	LDLR chaperone MESD
G3H5W5_CRIGR	LIM and SH3 domain protein 1
G3HX69_CRIGR	LIM domain and actin-binding protein 1
G3IK05_CRIGR	Lactadherin (Fragment)
G3ICD3_CRIGR	Lactadherin
Q99MH4_CRIGR	Lactate dehydrogenase (Fragment)
G3HLD0_CRIGR	Lactoylglutathione lyase
G3HG95_CRIGR	Lamin-A/C
G3HSM8_CRIGR	Lamin-B2
G3H1B3_CRIGR	Lamin-L(I)
G3I2N4_CRIGR	Lamina-associated polypeptide 2, isoforms alpha/zeta
G3IE68_CRIGR	Laminin subunit alpha-2
G3IEP6_CRIGR	Laminin subunit alpha-2
G3HGW6_CRIGR	Laminin subunit alpha-5
G3I278_CRIGR	Laminin subunit beta-1
G3IB69_CRIGR	Laminin subunit beta-2
G3HG25_CRIGR	Laminin subunit gamma-1
G3H8Y0_CRIGR	Lanosterol synthase
G3HZG6_CRIGR	Large proline-rich protein BAT3
G3HEV3_CRIGR	Latent-transforming growth factor beta-binding protein 1
G3H2G1_CRIGR	Layilin
LAYN_CRIGR	Layilin
G3I1H5_CRIGR	Legumain
G3II57_CRIGR	Leucine carboxyl methyltransferase 1
G3HIN8_CRIGR	Leucine zipper protein 1
G3IJ02_CRIGR	Leucine-rich repeat flightless-interacting protein 1
G3HX58_CRIGR	Leucine-rich repeat transmembrane protein FLRT3
G3HHE3_CRIGR	Leucyl-tRNA synthetase, cytoplasmic
G3HHE4_CRIGR	Leucyl-tRNA synthetase, cytoplasmic
G3I136_CRIGR	Leukemia inhibitory factor
G3GWX6_CRIGR	Leukocyte elastase inhibitor A
G3I4J7_CRIGR	Leukosialin
G3HBI9_CRIGR	Leukotriene A-4 hydrolase
G3I6E3_CRIGR	Lin-7-like C
G3HQY6_CRIGR	Lipase
G3H6V7_CRIGR	Lipoprotein lipase
G3HQ70_CRIGR	Liprin-alpha-1
G3I0V9_CRIGR	Liprin-beta-1
G3I7X7_CRIGR	Liver carboxylesterase 1
G3I5L0_CRIGR	Liver carboxylesterase 4
G3I7X5_CRIGR	Liver carboxylesterase 4
G3HBH4_CRIGR	Low molecular weight phosphotyrosine protein phosphatase
LDLR_CRIGR	Low-density lipoprotein receptor
G3GTN0_CRIGR	Low-density lipoprotein receptor-related protein 8
G3GU92_CRIGR	Lupus La protein-like
G3I4N3_CRIGR	Lysine--tRNA ligase
SYK_CRIGR	Lysine--tRNA ligase
G3I411_CRIGR	Lysine-specific demethylase 2A
G3H246_CRIGR	Lysine-specific demethylase 2B
G3HUI4_CRIGR	Lysosomal Pro-X carboxypeptidase
G3HTE5_CRIGR	Lysosomal alpha-glucosidase
G3HCV4_CRIGR	Lysosomal alpha-mannosidase
G3H8V5_CRIGR	Lysosomal protective protein
G3HZC7_CRIGR	Lysosomal thioesterase PPT2
LAMP1_CRIGR	Lysosome-associated membrane glycoprotein 1
LAMP2_CRIGR	Lysosome-associated membrane glycoprotein 2
G3HEI6_CRIGR	Lysyl oxidase-like 1
G3I7U8_CRIGR	Lysyl oxidase-like 3
G3GZY4_CRIGR	Lysyl oxidase-like 4
G3IFK8_CRIGR	MAM domain-containing protein 2
G3IJU1_CRIGR	MAM domain-containing protein 2
Q8MHC4_CRIGR	MHC class I antigen Hm1-C2
Q8MHC2_CRIGR	MHC class I antigen Hm1-C4
Q8MHC1_CRIGR	MHC class I antigen Hm1-C5

G3IHR6_CRIGR	Macrophage colony-stimulating factor 1
G3GUV3_CRIGR	Macrophage metalloelastase
G3HY08_CRIGR	Macrophage migration inhibitory factor
G3HPZ5_CRIGR	Macrophage-capping protein
PRIO_CRIGR	Major prion protein
G3I4I9_CRIGR	Major vault protein
G3HDQ2_CRIGR	Malate dehydrogenase
G3GTC7_CRIGR	Malic enzyme (Fragment)
G3GV64_CRIGR	Mammalian ependymin-related protein 1
G3H8K2_CRIGR	Mannosyl-oligosaccharide 1,2-alpha-mannosidase IA
G3H910_CRIGR	Mannosyl-oligosaccharide 1,2-alpha-mannosidase IB
G3HRK9_CRIGR	Matrix metalloproteinase-19
G3H8V1_CRIGR	Matrix metalloproteinase-9
G3GRB9_CRIGR	Melanoma inhibitory activity protein 3
MBTP1_CRIGR	Membrane-bound transcription factor site-1 protease
G3H8A8_CRIGR	Mesencephalic astrocyte-derived neurotrophic factor
G3IBH0_CRIGR	Metalloproteinase inhibitor 1
G3H3E6_CRIGR	Metalloproteinase inhibitor 2
G3HBJ9_CRIGR	Methionine aminopeptidase 2
G3HWD4_CRIGR	Methionine synthase
G3HW44_CRIGR	Methionyl-tRNA synthetase, cytoplasmic
G3I5E0_CRIGR	Methylosome subunit pICln
G3HN72_CRIGR	Microtubule-actin cross-linking factor 1, isoform 4
G3H0V1_CRIGR	Microtubule-associated protein 1A
G3HIT7_CRIGR	Microtubule-associated protein 1B
G3HKQ7_CRIGR	Microtubule-associated protein
G3HE84_CRIGR	Microtubule-associated protein RP/EB family member 1
G3HAP1_CRIGR	Microtubule-associated proteins 1A/1B light chain 3A
G3H601_CRIGR	Mitochondrial import inner membrane translocase subunit Tim8 A
G3HJK8_CRIGR	Mitochondrial inner membrane protein
G3HSH0_CRIGR	Mitochondrial ribonuclease P protein 1
G3HFY3_CRIGR	Mitogen-activated protein kinase 1
G3I4H1_CRIGR	Mitogen-activated protein kinase 3
G3HYJ9_CRIGR	Moesin
G3IDS7_CRIGR	Monocarboxylate transporter 1
G3H4A9_CRIGR	Mpv17-like protein 2
MDR1_CRIGR	Multidrug resistance protein 1
MDR2_CRIGR	Multidrug resistance protein 2
G3HW66_CRIGR	Multiple inositol polyphosphate phosphatase 1
D0UZH9_CRIGR	Mutant adenylosuccinate lyase
G3HSA2_CRIGR	Myb-binding protein 1A
G3HKJ6_CRIGR	Myc box-dependent-interacting protein 1 (Fragment)
G3HJV4_CRIGR	Myosin light polypeptide 6
G3HRQ4_CRIGR	Myosin light polypeptide 6
G3IA94_CRIGR	Myosin regulatory light chain 12B
G3GXV8_CRIGR	Myosin regulatory light polypeptide 9
G3GY44_CRIGR	Myosin-10
G3HFM7_CRIGR	Myosin-11
G3I729_CRIGR	Myosin-14
G3IH63_CRIGR	Myosin-9
G3HTF7_CRIGR	Myosin-Ic
G3HQI8_CRIGR	Myosin-Vb
G3I7W5_CRIGR	Myosin-XVIIIa
G3H680_CRIGR	Myosin-binding protein C, slow-type
G3HGM6_CRIGR	N(4)-(Beta-N-acetylglucosaminyl)-L-asparaginase
G3GRS9_CRIGR	N-acetylgalactosamine-6-sulfatase
G3HKX9_CRIGR	N-acetylgalactosaminyltransferase 7
G3HBX0_CRIGR	N-acetylglucosamine-1-phosphotransferase subunit gamma
G3I8P7_CRIGR	N-acetylglucosamine-6-sulfatase
G3I2H6_CRIGR	N-acetyllactosaminide beta-1,3-N-acetylglucosaminyltransferase
G3HH11_CRIGR	N-acylneuraminate cytidylyltransferase
G3I015_CRIGR	NAD(P)H dehydrogenase [quinone] 1
G3HLE5_CRIGR	NADH dehydrogenase [ubiquinone] flavoprotein 3, mitochondrial
G3HDD4_CRIGR	NEDD8
G3H3S2_CRIGR	NKG2D ligand 4
G3H6Y6_CRIGR	NSFL1 cofactor p47
G3GTH2_CRIGR	Na(+)/H(+) exchange regulatory cofactor NHE-RF1
G3GVY7_CRIGR	Nardilysin
G3HRM8_CRIGR	Nascent polypeptide-associated complex subunit alpha, muscle-specific form
G3H8H3_CRIGR	Nck-associated protein 1 (Fragment)
G3HEJ4_CRIGR	Neogenin
G3HBJ4_CRIGR	Netrin-4 (Fragment)
G3I9A3_CRIGR	Neudesin
G3HJR1_CRIGR	Neurabin-2

G3H2I6_CRIGR	Neural cell adhesion molecule 1
G3HU31_CRIGR	Neuroblastoma suppressor of tumorigenicity 1
G3GY86_CRIGR	Neuroligin-2
G3I7K0_CRIGR	Neuroligin-3
G3H4M6_CRIGR	Neurolysin, mitochondrial
G3H3I7_CRIGR	Neuron navigator 1
G3HZ57_CRIGR	Neuropilin-1
G3I064_CRIGR	Neutral alpha-glucosidase AB
G3HWE4_CRIGR	Nidogen-1
G3I3U5_CRIGR	Nidogen-1
J9T0K1_CRIGR	Niemann-Pick type C2 protein (Fragment)
G3HU11_CRIGR	Nitrilase-like 1
G3IBB9_CRIGR	Nodal modulator 1 (Fragment)
G3I7J6_CRIGR	Non-POU domain-containing octamer-binding protein
G3I0Q0_CRIGR	Non-muscle caldesmon
G3GW11_CRIGR	Non-specific lipid-transfer protein
G3HSB7_CRIGR	Nuclear migration protein nudC
G3HYP6_CRIGR	Nuclear migration protein nudC
G3I3A8_CRIGR	Nuclear mitotic apparatus protein 1
G3HIK1_CRIGR	Nuclear pore complex protein Nup93
G3I8G6_CRIGR	Nuclear receptor coactivator 2
G3GZ85_CRIGR	Nuclease domain-containing protein 1
G3HZX6_CRIGR	Nucleobindin-1
G3IF52_CRIGR	Nucleobindin-2
G3H6U1_CRIGR	Nucleolar RNA helicase 2
G3H451_CRIGR	Nucleolar protein 56
G3GYX2_CRIGR	Nucleolar protein 58
G3IF80_CRIGR	Nucleolin
G3ICB9_CRIGR	Nucleolysin TIAR
G3IBF1_CRIGR	Nucleophosmin
G3GWV9_CRIGR	Nucleoprotein TPR
G3HBD3_CRIGR	Nucleoside diphosphate kinase
G3HBD4_CRIGR	Nucleoside diphosphate kinase
G3HBN6_CRIGR	Nucleoside diphosphate kinase
G3HT18_CRIGR	Nucleosome assembly protein 1-like 1
G3IIT5_CRIGR	Nucleosome assembly protein 1-like 4
G3I1P6_CRIGR	Nucleosome-binding protein 1
G3I8Y4_CRIGR	Nucleosome-binding protein 1
G3HEY8_CRIGR	Nucleotide exchange factor SIL1
G3GUC8_CRIGR	Obg-like ATPase 1
G3HHM5_CRIGR	Obscurin
G3HU02_CRIGR	Olfactomedin-like protein 2B
G3HQH7_CRIGR	Olfactomedin-like protein 3
G3ICI3_CRIGR	Olfactory receptor 2T2
G3I230_CRIGR	Oligoribonuclease, mitochondrial
G3IL84_CRIGR	Oligoribonuclease, mitochondrial
G3HGU9_CRIGR	Oncostatin-M specific receptor subunit beta
G3IEB7_CRIGR	Out at first protein-like
G3HE39_CRIGR	Oxidoreductase HTATIP2
G3HAC1_CRIGR	Oxysterol-binding protein-related protein 6
G3HXY1_CRIGR	PDZ and LIM domain protein 1
G3I6W0_CRIGR	PHD finger protein 1
G3HFC9_CRIGR	PR domain zinc finger protein 15
G3HN89_CRIGR	Palmitoyl-protein thioesterase 1
G3I740_CRIGR	Pantetheinase
G3HNF5_CRIGR	Papilin (Fragment)
G3H217_CRIGR	Pentraxin-related protein PTX3
G3HQD5_CRIGR	Peptidyl-glycine alpha-amidating monooxygenase B
G3GZF2_CRIGR	Peptidyl-prolyl cis-trans isomerase A
G3HA87_CRIGR	Peptidyl-prolyl cis-trans isomerase A
G3I1A7_CRIGR	Peptidyl-prolyl cis-trans isomerase A
G3IED5_CRIGR	Peptidyl-prolyl cis-trans isomerase A
PPIA_CRIGR	Peptidyl-prolyl cis-trans isomerase A
G3HN81_CRIGR	Peptidyl-prolyl cis-trans isomerase E
G3GTN6_CRIGR	Peptidyl-prolyl cis-trans isomerase
G3H533_CRIGR	Peptidyl-prolyl cis-trans isomerase
G3H6Y5_CRIGR	Peptidyl-prolyl cis-trans isomerase
G3HKB0_CRIGR	Peptidyl-prolyl cis-trans isomerase
G3HLN4_CRIGR	Peptidyl-prolyl cis-trans isomerase
G3HLP7_CRIGR	Peptidyl-prolyl cis-trans isomerase
G3HMB8_CRIGR	Peptidyl-prolyl cis-trans isomerase
G3HMM2_CRIGR	Peptidyl-prolyl cis-trans isomerase
G3I792_CRIGR	Peptidyl-prolyl cis-trans isomerase
G3I881_CRIGR	Peptidyl-prolyl cis-trans isomerase

G3HCI0_CRIGR	Perilipin
G3HJ76_CRIGR	Peripheral-type benzodiazepine receptor-associated protein 1
G3IJR3_CRIGR	Peripherin
G3HBI1_CRIGR	Peroxidasin-like
PRDX1_CRIGR	Peroxiredoxin-1
PRDX2_CRIGR	Peroxiredoxin-2
G3I887_CRIGR	Peroxiredoxin-5, mitochondrial
G3HCX7_CRIGR	Phenylalanyl-tRNA synthetase alpha chain
G3IBQ9_CRIGR	Phenylalanyl-tRNA synthetase beta chain
G3GU60_CRIGR	Phosphatidylethanolamine-binding protein 1
G3HTF5_CRIGR	Phosphatidylinositol transfer protein alpha isoform
G3HXR8_CRIGR	Phosphatidylinositol-binding clathrin assembly protein
G3GZD2_CRIGR	Phosphoglucomutase-1
G3IFE7_CRIGR	Phosphoglucomutase-2
G3HNY4_CRIGR	Phosphoglycerate kinase
G3HNY5_CRIGR	Phosphoglycerate kinase
G3HX39_CRIGR	Phosphoglycerate kinase
G3IBZ4_CRIGR	Phosphoglycerate kinase
G3GZW8_CRIGR	Phosphoglycerate mutase 1
G3HM99_CRIGR	Phosphoglycerate mutase 2
G3HBR6_CRIGR	Phosphoglycolate phosphatase
G3HWN7_CRIGR	Phosphoinositide phospholipase C (Fragment)
G3HH63_CRIGR	Phospholipase A-2-activating protein
G3H8V4_CRIGR	Phospholipid transfer protein
G3IEX5_CRIGR	Phosphopantothenate--cysteine ligase
M1R995_CRIGR	Phosphoribosylaminoimidazole carboxylase/ phosphoribosylaminoimidazole succinocarboxamide synthetase
M1R372_CRIGR	Phosphoribosylaminoimidazole carboxylase/phosphoribosylaminoimidazole succinocarboxamide synthetase
G3GY52_CRIGR	Phosphoribosylformylglycinamidine synthase
B2ZA78_CRIGR	Phosphoribosylglycinamide transformylase
G3H6Z4_CRIGR	Phosphorylase
G3ILF9_CRIGR	Phosphorylase
G3IKH9_CRIGR	Phosphoserine aminotransferase
G3I2M2_CRIGR	Phosphoserine phosphatase
G3IKX2_CRIGR	Pigment epithelium-derived factor
G3IQ06_CRIGR	Pigment epithelium-derived factor
G3HA54_CRIGR	Plasminogen activator inhibitor 1
G3I683_CRIGR	Plasminogen activator inhibitor 1 RNA-binding protein
G3GSZ0_CRIGR	Plastin-1
G3H8N1_CRIGR	Plastin-2
G3IBY4_CRIGR	Plastin-3
PLST_CRIGR	Plastin-3
G3HVB8_CRIGR	Platelet-activating factor acetylhydrolase IB subunit alpha
G3I5S9_CRIGR	Pleckstrin-likey-like domain family B member 1
PLEC_CRIGR	Plectin (Fragment)
G3HWJ2_CRIGR	Plectin-1
G3GRZ4_CRIGR	Plexin-A1
G3I2V6_CRIGR	Plexin-B2
G3IP74_CRIGR	Plexin-B2
G3HV73_CRIGR	Poly [ADP-ribose] polymerase 1
PARP1_CRIGR	Poly [ADP-ribose] polymerase 1
G3H9W3_CRIGR	Poly(ADP-ribose) glycohydrolase ARH3
G3HXL1_CRIGR	Poly(RC)-binding protein 1
G3H6Y9_CRIGR	Poly(RC)-binding protein 2
G3HV39_CRIGR	Poly(RC)-binding protein 2
G3H8Y6_CRIGR	Poly(RC)-binding protein 3
G3HWJ5_CRIGR	Poly(U)-binding-splicing factor PUF60
G3HWJ6_CRIGR	Poly(U)-binding-splicing factor PUF60
G3I8S7_CRIGR	Polyadenylate-binding protein 1
G3HN77_CRIGR	Polyadenylate-binding protein 4
G3H9N4_CRIGR	Polypeptide N-acetylgalactosaminyltransferase 1
G3GX17_CRIGR	Polypeptide N-acetylgalactosaminyltransferase 2
G3HF46_CRIGR	Polypyrimidine tract-binding protein 1
G3IHX5_CRIGR	Polypyrimidine tract-binding protein 1
O35080_CRIGR	Polyubiquitin
G3HPT4_CRIGR	Potassium-transporting ATPase alpha chain 1
G3IPB5_CRIGR	Pre-mRNA-processing-splicing factor 8
G3HU10_CRIGR	Prefoldin subunit 2
G3HV83_CRIGR	Prefoldin subunit 3
G3I6W5_CRIGR	Prefoldin subunit 6
G3HXK8_CRIGR	Prenylcysteine oxidase
G3I664_CRIGR	Procollagen C-endopeptidase enhancer 1
G3GSZ1_CRIGR	Procollagen C-endopeptidase enhancer 2
G3IIE7_CRIGR	Procollagen-lysine,2-oxoglutarate 5-dioxygenase 1
G3HVR6_CRIGR	Procollagen-lysine,2-oxoglutarate 5-dioxygenase 2

G3HA51_CRIGR	Procollagen-lysine,2-oxoglutarate 5-dioxygenase 3
G3GYC6_CRIGR	Profilin-1
G3H2N6_CRIGR	Programmed cell death 6-interacting protein
PCNA_CRIGR	Proliferating cell nuclear antigen
G3HRK0_CRIGR	Proliferation-associated protein 2G4
G3H1G7_CRIGR	Proline synthetase co-transcribed bacterial-like protein
G3GYB0_CRIGR	Proline-, glutamic acid-and leucine-rich protein 1
G3HW34_CRIGR	Prolow-density lipoprotein receptor-related protein 1
G3HJ24_CRIGR	Prolyl 4-hydroxylase subunit alpha-1
G3I366_CRIGR	Prolyl endopeptidase
G3H902_CRIGR	Prostaglandin F2 receptor negative regulator
G3I5Z5_CRIGR	Prostaglandin reductase 1
G3HDE5_CRIGR	Proteasome activator complex subunit 1
G3HUD3_CRIGR	Proteasome activator complex subunit 1
G3HUD4_CRIGR	Proteasome activator complex subunit 1
G3HDE3_CRIGR	Proteasome activator complex subunit 2
G3HFL0_CRIGR	Proteasome activator complex subunit 4
G3H0C2_CRIGR	Proteasome subunit alpha type
G3HSE4_CRIGR	Proteasome subunit alpha type
G3HSF3_CRIGR	Proteasome subunit alpha type
G3I9G7_CRIGR	Proteasome subunit alpha type
G3I3X6_CRIGR	Proteasome subunit alpha type-6
G3GWR8_CRIGR	Proteasome subunit alpha type-7 (Fragment)
G3H303_CRIGR	Proteasome subunit beta type
G3H5W0_CRIGR	Proteasome subunit beta type
G3H9V0_CRIGR	Proteasome subunit beta type
G3HQS8_CRIGR	Proteasome subunit beta type
G3HQT0_CRIGR	Proteasome subunit beta type
G3HRD9_CRIGR	Proteasome subunit beta type
G3I9D3_CRIGR	Proteasome subunit beta type
G3HNT9_CRIGR	Proteasome subunit beta type-1
G3GYB7_CRIGR	Proteasome subunit beta type-6
G3I5Z8_CRIGR	Proteasome-associated protein ECM29-like
G3HE67_CRIGR	Protein CREG1
G3IGW0_CRIGR	Protein CTLA-2-beta
G3IDP5_CRIGR	Protein CYR61
G3I6V9_CRIGR	Protein CutA
G3HRX5_CRIGR	Protein DBF4-like A
G3GUG7_CRIGR	Protein DEK
G3IEU2_CRIGR	Protein DJ-1
G3H5H0_CRIGR	Protein FAM38B
G3IDN7_CRIGR	Protein FAM3C
G3GXQ2_CRIGR	Protein FAM59B
G3HEN3_CRIGR	Protein HIRA
G3GUY5_CRIGR	Protein KIAA1967-like
G3GXW5_CRIGR	Protein NDRG3
G3H9S0_CRIGR	Protein Niban
G3GYN7_CRIGR	Protein O-linked-mannose beta-1,2-N-acetylglucosaminyltransferase 1
G3HW54_CRIGR	Protein OS-9
G3HUU6_CRIGR	Protein S100-A11
G3HC31_CRIGR	Protein S100-A6
G3GR87_CRIGR	Protein SET
G3GWV4_CRIGR	Protein SET
G3H1C4_CRIGR	Protein SET
G3HEK4_CRIGR	Protein SET
G3HKN1_CRIGR	Protein SET
G3I9X3_CRIGR	Protein SET
G3IHC7_CRIGR	Protein arginine N-methyltransferase 1
G3I7E3_CRIGR	Protein argonaute-2
G3HJV0_CRIGR	Protein canopy-like 2
G3HRP2_CRIGR	Protein canopy-like 2
G3HR63_CRIGR	Protein canopy-like 3
G3I5F4_CRIGR	Protein canopy-like 4
G3H0U6_CRIGR	Protein disulfide-isomerase A3
G3IDT6_CRIGR	Protein disulfide-isomerase A4
G3HB04_CRIGR	Protein disulfide-isomerase A6
G3GXA9_CRIGR	Protein disulfide-isomerase
PDIA1_CRIGR	Protein disulfide-isomerase
Q91Z81_CRIGR	Protein disulfide-isomerase
G3HXT3_CRIGR	Protein kinase C and casein kinase substrate in neurons protein 2
G3HXT4_CRIGR	Protein kinase C and casein kinase substrate in neurons protein 2
G3H3M6_CRIGR	Protein phosphatase 1 regulatory subunit 12
G3HZ17_CRIGR	Protein phosphatase 1 regulatory subunit 12
G3HFY1_CRIGR	Protein phosphatase 1F

G3GXU1_CRIGR	Protein phosphatase 1G
G3I9G1_CRIGR	Protein piccolo
G3HKP7_CRIGR	Protein shisa-5
G3GVW1_CRIGR	Protein transport protein Sec23B
G3IL48_CRIGR	Protein transport protein Sec31A
G3IG20_CRIGR	Protein unc-45-like A
G3H3S4_CRIGR	Protein-L-isoaspartate(D-aspartate) O-methyltransferase
G3GXZ0_CRIGR	Protein-glutamine gamma-glutamyltransferase 2
G3IDU4_CRIGR	Protein-tyrosine sulfotransferase 1
G3IH46_CRIGR	Prothymosin alpha
G3HTF8_CRIGR	Proto-oncogene C-crk
G3GXX7_CRIGR	Proto-oncogene tyrosine-protein kinase Src
G3IFY3_CRIGR	Proto-oncogene tyrosine-protein kinase Yes
G3HVZ2_CRIGR	Protocadherin Fat 1
G3H6N7_CRIGR	Protocadherin Fat 3 (Fragment)
G3IB24_CRIGR	Protocadherin beta-17
G3IB21_CRIGR	Protocadherin beta-5
G3IB23_CRIGR	Protocadherin beta-6
G3HYE4_CRIGR	Protocadherin gamma-C3
G3I0S4_CRIGR	Protocadherin-7
G3I1R2_CRIGR	Purine nucleoside phosphorylase (Fragment)
G3I6K0_CRIGR	Putative ATP-dependent RNA helicase DDX23
G3I5T3_CRIGR	Putative ATP-dependent RNA helicase DDX6
G3HXU5_CRIGR	Putative ATP-dependent RNA helicase DHX36
G3HVE1_CRIGR	Putative ATP-dependent RNA helicase YTHDC2
G3HTI7_CRIGR	Putative G-protein coupled receptor 116 (Fragment)
G3HE92_CRIGR	Putative Polycomb group protein ASXL1
G3GZ56_CRIGR	Putative adenosylhomocysteinase 3
G3I3C7_CRIGR	Putative allantoicase
G3I474_CRIGR	Putative bifunctional methylenetetrahydrofolate dehydrogenase/cyclohydrolase 2
G3H9Z3_CRIGR	Putative deoxyribose-phosphate aldolase
G3IGL0_CRIGR	Putative heterogeneous nuclear ribonucleoprotein A1-like protein 3
G3GVW2_CRIGR	Putative hydrolase RBBP9
G3I6T1_CRIGR	Putative phospholipase B-like 2
G3ID08_CRIGR	Putative pre-mRNA-splicing factor ATP-dependent RNA helicase DHX15
G3GX59_CRIGR	Putative sodium-coupled neutral amino acid transporter 10
G3GWL9_CRIGR	Putative uncharacterized protein
G3H283_CRIGR	Putative uncharacterized protein
G3HDU1_CRIGR	Putative uncharacterized protein
G3HKI0_CRIGR	Putative uncharacterized protein
G3HWJ3_CRIGR	Putative uncharacterized protein
G3HY47_CRIGR	Putative uncharacterized protein
G3I0G4_CRIGR	Putative uncharacterized protein
G3IAF3_CRIGR	Putative uncharacterized protein
G3IFA2_CRIGR	Putative uncharacterized protein
G3IKQ5_CRIGR	Pyridoxal kinase
G3HFN7_CRIGR	Pyridoxal-dependent decarboxylase domain-containing protein 1
G3H3Q1_CRIGR	Pyruvate kinase
G3HC41_CRIGR	Pyruvate kinase
G3IAX3_CRIGR	Pyruvate kinase
G3IJ43_CRIGR	Pyruvate kinase
G3HJD2_CRIGR	RNA polymerase II-associated factor 1-like
G3HAR7_CRIGR	RNA-binding protein 39
G3HP03_CRIGR	RNA-binding protein EWS
G3GZW7_CRIGR	RRP12-like protein
G3IBK9_CRIGR	Rab GDP dissociation inhibitor alpha
G3GR73_CRIGR	Rab GDP dissociation inhibitor beta
G3ILV6_CRIGR	Rab GDP dissociation inhibitor beta
G3I198_CRIGR	Rab effector MyRIP
G3HFP1_CRIGR	Radixin
G3I531_CRIGR	Ran GTPase-activating protein 1
G3HEQ3_CRIGR	Ran-specific GTPase-activating protein
G3I9F1_CRIGR	Ras GTPase-activating protein-binding protein 2
G3IF62_CRIGR	Ras GTPase-activating-like protein IQGAP1
G3H331_CRIGR	Ras GTPase-activating-like protein IQGAP2
G3HG79_CRIGR	Ras GTPase-activating-like protein IQGAP3
G3HJB6_CRIGR	Ras and Rab interactor-like protein
G3HN65_CRIGR	Ras suppressor protein 1
G3GVB5_CRIGR	Ras-related C3 botulinum toxin substrate 1
G3I0B0_CRIGR	Ras-related protein Rab-11A
G3I5Q9_CRIGR	Ras-related protein Rab-11B
G3HX83_CRIGR	Ras-related protein Rab-14
G3I2E9_CRIGR	Ras-related protein Rab-1B
G3GU73_CRIGR	Ras-related protein Rab-35

G3HLW5_CRIGR	Ras-related protein Rab-5C
G3I9W7_CRIGR	Ras-related protein Rab-6A (Fragment)
G3GZG4_CRIGR	Ras-related protein Rab-6B
G3HID1_CRIGR	Ras-related protein Rab-7a
G3H4I1_CRIGR	Ras-related protein Rab-8A
G3H515_CRIGR	Ras-related protein Rab-8B
G3IBI0_CRIGR	Ras-related protein Rap-1A
G3GY22_CRIGR	Ras-related protein Rap-1b
G3GTA7_CRIGR	Ras-specific guanine nucleotide-releasing factor 1
SYRC_CRIGR	RecName: Full
HMGA1_CRIGR	RecName: Full
G3HRK1_CRIGR	Receptor tyrosine-protein kinase erbB-3
G3HCI4_CRIGR	Receptor-type tyrosine-protein phosphatase S
G3HCI6_CRIGR	Receptor-type tyrosine-protein phosphatase S
G3H7L5_CRIGR	Receptor-type tyrosine-protein phosphatase gamma (Fragment)
G3HMM0_CRIGR	Receptor-type tyrosine-protein phosphatase zeta
G3HPJ9_CRIGR	Regulator of differentiation 1
G3GSG4_CRIGR	Renin receptor
G3H201_CRIGR	Replication protein A 32 kDa subunit
G3IP86_CRIGR	Replication protein A 70 kDa DNA-binding subunit
G3GZA9_CRIGR	Reticulocalbin-1 (Fragment)
G3ILN5_CRIGR	Reticulocalbin-3
G3H1D5_CRIGR	Retinoid-inducible serine carboxypeptidase
G3GXB0_CRIGR	Rho GDP-dissociation inhibitor 1
G3IJU2_CRIGR	Rho GTPase-activating protein 18
G3I1U6_CRIGR	Rho guanine nucleotide exchange factor 7
G3IDS4_CRIGR	Rho-related GTP-binding protein RhoC
G3I4D4_CRIGR	Ribonuclease T2
G3I3F3_CRIGR	Ribonuclease inhibitor
G3H6D0_CRIGR	Ribonucleoprotein PTB-binding 1
G3HJJ1_CRIGR	Ribose-5-phosphate isomerase
G3HD67_CRIGR	Ribose-phosphate pyrophosphokinase 1
G3HV97_CRIGR	Ribose-phosphate pyrophosphokinase 1
G3I9Z3_CRIGR	Ribose-phosphate pyrophosphokinase 2
G3GWL7_CRIGR	Ribosomal L1 domain-containing protein 1
G3H1F4_CRIGR	Ribosomal protein L15
G3HZV0_CRIGR	Ribosomal protein
G3GVX1_CRIGR	Ribosome-binding protein 1
G3IEG3_CRIGR	Ribosyldihydronicotinamide dehydrogenase [quinone]
G3HIC5_CRIGR	RuvB-like 1
G3HZY1_CRIGR	RuvB-like 2
G3HNS9_CRIGR	Ryanodine receptor 2
G3I3W4_CRIGR	S-formylglutathione hydrolase
G3I0I7_CRIGR	S-methyl-5'-thioadenosine phosphorylase
G3H2C9_CRIGR	S-phase kinase-associated protein 1
G3GXW9_CRIGR	SAM domain and HD domain-containing protein 1
G3IAY3_CRIGR	SEC23-interacting protein
G3I1P5_CRIGR	SH3 domain-binding glutamic acid-rich-like protein
G3I3D5_CRIGR	SH3 domain-binding glutamic acid-rich-like protein
G3HJ18_CRIGR	SH3 domain-containing RING finger protein 3 (Fragment)
G3H584_CRIGR	SPARC
G3IGI9_CRIGR	STE20-like serine/threonine-protein kinase
G3HUM5_CRIGR	SUMO-activating enzyme subunit 2
G3HRY8_CRIGR	SWI/SNF-related matrix-associated actin-dependent regulator of chromatin subfamily A member 5
G3HHH6_CRIGR	Salivary plasminogen activator alpha 2
G3HCI9_CRIGR	Scaffold attachment factor B1
G3HCI7_CRIGR	Scaffold attachment factor B2
G3HFJ1_CRIGR	Scavenger mRNA-decapping enzyme DcpS
G3HYI3_CRIGR	Scavenger receptor cysteine-rich domain-containing protein LOC284297-like
G3H4J6_CRIGR	Sec1 family domain-containing protein 1
G3GYA1_CRIGR	Segment polarity protein dishevelled-like DVL-2
G3GVQ8_CRIGR	Selenide, water dikinase 1
G3I249_CRIGR	Selenocysteine lyase
G3ID68_CRIGR	Selenoprotein P
G3H8C9_CRIGR	Semaphorin-3B
G3HMI3_CRIGR	Semaphorin-3C
G3HN31_CRIGR	Semaphorin-3E
G3IHT7_CRIGR	Semaphorin-4B
G3HCG5_CRIGR	Semaphorin-6B
G3IC99_CRIGR	Septin-11
G3H4V1_CRIGR	Septin-2
G3H771_CRIGR	Septin-6
G3HTJ2_CRIGR	Septin-7 (Fragment)
G3H2B7_CRIGR	Septin-8

G3H3G9_CRIGR	Septin-9
G3GVI3_CRIGR	Serine hydroxymethyltransferase
G3HW36_CRIGR	Serine hydroxymethyltransferase
G3I2U1_CRIGR	Serine protease 27
G3IBF4_CRIGR	Serine protease HTRA1
G3H9Z4_CRIGR	Serine-threonine kinase receptor-associated protein
G3IFB0_CRIGR	Serine/arginine repetitive matrix protein 2
G3HQV2_CRIGR	Serine/threonine-protein kinase OSR1
G3GSK4_CRIGR	Serine/threonine-protein kinase PAK 2
G3GU61_CRIGR	Serine/threonine-protein kinase TAO3
G3H627_CRIGR	Serine/threonine-protein phosphatase (Fragment)
G3HZP8_CRIGR	Serine/threonine-protein phosphatase (Fragment)
G3HY75_CRIGR	Serine/threonine-protein phosphatase 2A 55 kDa regulatory subunit B
G3I2B6_CRIGR	Serine/threonine-protein phosphatase 2A 65 kDa regulatory subunit A alpha isoform (Fragment)
G3H2G5_CRIGR	Serine/threonine-protein phosphatase 2A 65 kDa regulatory subunit A beta isoform
G3HJT2_CRIGR	Serine/threonine-protein phosphatase 4 regulatory subunit 3B
G3I5T6_CRIGR	Serine/threonine-protein phosphatase 5
G3I5B3_CRIGR	Serine/threonine-protein phosphatase 6 regulatory subunit 3
G3GW88_CRIGR	Serine/threonine-protein phosphatase
G3H298_CRIGR	Serine/threonine-protein phosphatase
G3IEG2_CRIGR	Serpin B6
G3IDD4_CRIGR	Serpin H1
G3IGS8_CRIGR	Serrate RNA effector molecule-like
G3ILI3_CRIGR	Seryl-tRNA synthetase, cytoplasmic
G3IIB1_CRIGR	Sialate O-acetylesterase
B8Y440_CRIGR	Sialidase I
G3HZE3_CRIGR	Sialidase-1
G3IFL3_CRIGR	Signal recognition particle 72 kDa protein
G3IBF6_CRIGR	Sister chromatid cohesion protein PDS5-like A
G3IGT5_CRIGR	Sjoegren syndrome/scleroderma autoantigen 1
G3H453_CRIGR	Small nuclear ribonucleoprotein-associated protein
G3I1I8_CRIGR	Small nuclear ribonucleoprotein-associated protein
SUMO2_CRIGR	Small ubiquitin-related modifier 2
G3H8Z9_CRIGR	Sodium/potassium-transporting ATPase subunit alpha-1
G3H7Y5_CRIGR	Sodium/potassium-transporting ATPase subunit alpha-2
G3I0M9_CRIGR	Sodium/potassium-transporting ATPase subunit alpha-3
G3H3E3_CRIGR	Soluble calcium-activated nucleotidase 1
G3GWE9_CRIGR	Solute carrier family 2, facilitated glucose transporter member 9
G3H0S4_CRIGR	Sorbitol dehydrogenase
G3HRX0_CRIGR	Sorcin (Fragment)
SORCN_CRIGR	Sorcin
G3I7A8_CRIGR	Sortilin (Fragment)
G3HFW9_CRIGR	Sorting nexin
G3H530_CRIGR	Sorting nexin-1
G3HKA5_CRIGR	Sorting nexin-2
G3GVW9_CRIGR	Sorting nexin-5
G3I9X6_CRIGR	Spectrin alpha chain, brain
G3I9X8_CRIGR	Spectrin alpha chain, brain
G3HJS0_CRIGR	Spectrin beta chain, brain 1
G3HJS1_CRIGR	Spectrin beta chain, brain 1
G3HLH5_CRIGR	Spermatid perinuclear RNA-binding protein
G3HTL1_CRIGR	Spermatogenesis-associated protein 5
G3IEE6_CRIGR	Spermidine synthase
G3IMH4_CRIGR	Sphingomyelin phosphodiesterase
G3HZH3_CRIGR	Spliceosome RNA helicase BAT1
G3INX6_CRIGR	Spliceosome RNA helicase BAT1
G3I142_CRIGR	Splicing factor 3 subunit 1
G3HE04_CRIGR	Splicing factor 3A subunit 3
G3HL16_CRIGR	Splicing factor 3B subunit 1
G3I2F2_CRIGR	Splicing factor 3B subunit 2
G3HAF4_CRIGR	Splicing factor 3B subunit 3
G3H2U6_CRIGR	Splicing factor 3B subunit 4
G3HYI7_CRIGR	Splicing factor U2AF 65 kDa subunit
G3H9U3_CRIGR	Splicing factor, proline-and glutamine-rich
G3HVJ5_CRIGR	Squamous cell carcinoma antigen recognized by T-cells 3
G3HQ69_CRIGR	Src substrate cortactin
G3H9Q6_CRIGR	Stanniocalcin-2
GRP75_CRIGR	Stress-70 protein, mitochondrial
G3I877_CRIGR	Stress-induced-phosphoprotein 1
STIP1_CRIGR	Stress-induced-phosphoprotein 1
G3IIZ6_CRIGR	Structural maintenance of chromosomes protein 3
G3H7U2_CRIGR	Structural maintenance of chromosomes protein
G3H343_CRIGR	Sulfatase-modifying factor 1
G3I1Y9_CRIGR	Sulfated glycoprotein 1

G3H7I6_CRIGR	Sulfhydryl oxidase
G3I6Y6_CRIGR	Sulfhydryl oxidase
G3I8Z5_CRIGR	Sulfiredoxin
G3HHX7_CRIGR	Superkiller viralicidic activity 2-like 2
G3I8D0_CRIGR	Suppressor of G2 allele of SKP1-like
G3H6E2_CRIGR	Suppressor of SWI4 1-like
G3GTX3_CRIGR	Suppressor of tumorigenicity 14 protein homolog
G3HPT8_CRIGR	Suprabasin
G3GSF3_CRIGR	Sushi repeat-containing protein SRPX
G3HUC4_CRIGR	Sushi, von Willebrand factor type A, EGF and pentraxin domain-containing protein 1
G3HM03_CRIGR	Synaptic vesicle membrane protein VAT-1-like
G3HDJ3_CRIGR	Syndecan
G3HLT3_CRIGR	Syndecan
G3H204_CRIGR	Syntaxin-12
G3HD57_CRIGR	Syntenin-1
TCPA_CRIGR	T-complex protein 1 subunit alpha
G3HZ42_CRIGR	T-complex protein 1 subunit beta
G3HDR3_CRIGR	T-complex protein 1 subunit delta
G3H7K5_CRIGR	T-complex protein 1 subunit epsilon
G3HYB7_CRIGR	T-complex protein 1 subunit eta
G3HG83_CRIGR	T-complex protein 1 subunit gamma
G3GT05_CRIGR	T-complex protein 1 subunit theta
G3GT06_CRIGR	T-complex protein 1 subunit theta
G3I2M1_CRIGR	T-complex protein 1 subunit zeta
G3H9Y3_CRIGR	TBC1 domain family member 5
G3HEB6_CRIGR	TGF-beta receptor type III
G3IKT2_CRIGR	THO complex subunit 2
G3GXB1_CRIGR	THO complex subunit 4
G3I180_CRIGR	TRAF family member-associated NF-kappa-B activator
O88679_CRIGR	TRIP protein (Fragment)
G3HN02_CRIGR	Talin-1
G3H510_CRIGR	Talin-2
G3HZD1_CRIGR	Tenascin-X
G3HZD3_CRIGR	Tenascin-X
G3IEV8_CRIGR	Testin-2
G3HSM3_CRIGR	Thimet oligopeptidase
G3HSA7_CRIGR	Thioredoxin domain-containing protein 17
G3HD97_CRIGR	Thioredoxin domain-containing protein 5
G3HQL6_CRIGR	Thioredoxin reductase 1, cytoplasmic
G3HIQ0_CRIGR	Threonyl-tRNA synthetase, cytoplasmic
G3HHV4_CRIGR	Thrombospondin-1
G3HK37_CRIGR	Thrombospondin-2
G3HC49_CRIGR	Thrombospondin-3
G3HXA5_CRIGR	Thrombospondin-4 (Fragment)
G3IFY1_CRIGR	Thymidylate synthase
G3GRU3_CRIGR	Thyrotropin-releasing hormone receptor
G3IMK2_CRIGR	Tight junction protein ZO-2
G3HMV7_CRIGR	Tissue alpha-L-fucosidase
G3HAC6_CRIGR	Titin
G3GZG1_CRIGR	Toll-like receptor 9 (Fragment)
G3GZU7_CRIGR	Tolloid-like protein 2
G3IGZ8_CRIGR	Torsin-1B
G3I3G8_CRIGR	Transaldolase
G3ILL5_CRIGR	Transaldolase
TALDO_CRIGR	Transaldolase
G3I151_CRIGR	Transcobalamin-2
G3IFA9_CRIGR	Transcription elongation factor B polypeptide 2
G3IGH6_CRIGR	Transcription elongation factor SPT6
G3GSM5_CRIGR	Transferrin receptor protein 1
G3IA12_CRIGR	Transforming growth factor beta-1
G3H8F9_CRIGR	Transforming protein RhoA
G3H7Z2_CRIGR	Transgelin-2
G3HN14_CRIGR	Transitional endoplasmic reticulum ATPase
G3GUU5_CRIGR	Transketolase
G3GU75_CRIGR	Translational activator GCN1
G3H470_CRIGR	Transmembrane 6 superfamily member 2
G3IE06_CRIGR	Transmembrane protein 132A
G3HCW0_CRIGR	Transportin-2
G3GT56_CRIGR	Trifunctional purine biosynthetic protein adenosine-3
G3HIL0_CRIGR	Triosephosphate isomerase
G3HNV4_CRIGR	Triosephosphate isomerase
G3I216_CRIGR	Triosephosphate isomerase
G3IDE4_CRIGR	Tripeptidyl-peptidase 1
G3H511_CRIGR	Tropomyosin alpha-1 chain

G3H116_CRIGR	Tropomyosin alpha-3 chain
G3HC01_CRIGR	Tropomyosin alpha-3 chain
G3H4I2_CRIGR	Tropomyosin beta chain
G3HIX6_CRIGR	Tryptophanyl-tRNA synthetase, cytoplasmic
G3IIZ5_CRIGR	Tubulin alpha chain
G3INA2_CRIGR	Tubulin alpha chain, nucleomorph
G3H588_CRIGR	Tubulin alpha-1B chain
TBA1C_CRIGR	Tubulin alpha-1C chain
G3H3C6_CRIGR	Tubulin alpha-3 chain
G3HWE9_CRIGR	Tubulin alpha-4A chain
G3HQ80_CRIGR	Tubulin alpha-8 chain
G3GZ08_CRIGR	Tubulin beta-2 chain
G3IEG6_CRIGR	Tubulin beta-2A chain
G3GRQ1_CRIGR	Tubulin beta-3 chain
G3HCL2_CRIGR	Tubulin beta-4 chain
TBB5_CRIGR	Tubulin beta-5 chain
G3HXU0_CRIGR	Tubulin--tyrosine ligase-like protein 12
G3H319_CRIGR	Tubulin-specific chaperone A
G3I449_CRIGR	Tubulin-specific chaperone D
G3H1W4_CRIGR	Tubulointerstitial nephritis antigen-like
G3HF18_CRIGR	Tudor domain-containing protein 12
G3HCL3_CRIGR	Tumor necrosis factor ligand superfamily member 9
G3IL01_CRIGR	Tumor necrosis factor receptor superfamily member 1B
G3HTI5_CRIGR	Tumor necrosis factor receptor superfamily member 21
G3HQY3_CRIGR	Tumor necrosis factor receptor superfamily member 6
Q6W3F7_CRIGR	Tumor necrosis factor-alpha-converting enzyme mutant variant M1
Q6W3F6_CRIGR	Tumor necrosis factor-alpha-converting enzyme mutant variant M2a
Q6W3F5_CRIGR	Tumor necrosis factor-alpha-converting enzyme mutant variant M2b
G3IDR6_CRIGR	Tumor necrosis factor-inducible gene 6 protein
G3H0V2_CRIGR	Tumor suppressor p53-binding protein 1
G3IB32_CRIGR	Twinfilin-1
G3HZ28_CRIGR	Twisted gastrulation protein-like 1
G3H1Q1_CRIGR	Type I inositol-3,4-bisphosphate 4-phosphatase
Q60444_CRIGR	Type VII collagen (Fragment)
G3H277_CRIGR	Tyrosine-protein phosphatase non-receptor type 11
G3HKR3_CRIGR	Tyrosine-protein phosphatase non-receptor type 23
G3H935_CRIGR	Tyrosyl-tRNA synthetase, cytoplasmic
G3HZY5_CRIGR	U1 small nuclear ribonucleoprotein 70 kDa
G3HNR3_CRIGR	U1 small nuclear ribonucleoprotein A
G3H897_CRIGR	U3 small nucleolar RNA-interacting protein 2
G3I2F8_CRIGR	U4/U6.U5 tri-snRNP-associated protein 1
G3HPY5_CRIGR	U4/U6.U5 tri-snRNP-associated protein 2
G3H037_CRIGR	U5 small nuclear ribonucleoprotein 200 kDa helicase
G3GSL6_CRIGR	UBX domain-containing protein 7
G3HDQ9_CRIGR	UDP-GlcNAc:betaGal beta-1,3-N-acetylglucosaminyltransferase 2
G3HQK5_CRIGR	UDP-N-acetylhexosamine pyrophosphorylase
G3IJB9_CRIGR	UDP-N-acetylhexosamine pyrophosphorylase-like protein 1
G3INU6_CRIGR	UDP-glucose 6-dehydrogenase
G3H1S2_CRIGR	UDP-glucose:glycoprotein glucosyltransferase 1
G3H5N5_CRIGR	UDP-glucuronic acid decarboxylase 1
G3HCH0_CRIGR	UPF0556 protein C19orf10-like
UGPA_CRIGR	UTP--glucose-1-phosphate uridylyltransferase
G3HAQ4_CRIGR	Ubiquinol-cytochrome c reductase complex chaperone CBP3-like
G3I129_CRIGR	Ubiquitin
G3IBG3_CRIGR	Ubiquitin activating enzyme E1
G3GSH2_CRIGR	Ubiquitin carboxyl-terminal hydrolase
G3GUP7_CRIGR	Ubiquitin carboxyl-terminal hydrolase
G3H9L2_CRIGR	Ubiquitin carboxyl-terminal hydrolase
G3HNA5_CRIGR	Ubiquitin carboxyl-terminal hydrolase isozyme L3 (Fragment)
G3I873_CRIGR	Ubiquitin thioesterase OTUB1
G3HVI1_CRIGR	Ubiquitin-conjugating enzyme E2 D2
G3IJ85_CRIGR	Ubiquitin-conjugating enzyme E2 D3
G3H8Z7_CRIGR	Ubiquitin-conjugating enzyme E2 G2 (Fragment)
G3IBF7_CRIGR	Ubiquitin-conjugating enzyme E2 K
G3HFY9_CRIGR	Ubiquitin-conjugating enzyme E2 L3
G3HS88_CRIGR	Ubiquitin-conjugating enzyme E2 N (Fragment)
G3HET8_CRIGR	Ubiquitin-conjugating enzyme E2 variant 1
G3HH02_CRIGR	Uncharacterized protein C20orf135-like
G3H9U6_CRIGR	Uncharacterized protein KIAA0319-like
Q9R119_CRIGR	Urokinase plasminogen activator surface receptor (Fragment)
G3HYY3_CRIGR	Urokinase plasminogen activator surface receptor
G3H066_CRIGR	V-type proton ATPase catalytic subunit A
G3H6W0_CRIGR	V-type proton ATPase subunit B, brain isoform (Fragment)
G3IBX5_CRIGR	V-type proton ATPase subunit B, kidney isoform

G3H1M5_CRIGR	V-type proton ATPase subunit D
G3HQ89_CRIGR	V-type proton ATPase subunit E 1
G3IBK8_CRIGR	V-type proton ATPase subunit S1
G3GZC9_CRIGR	VWFA and cache domain-containing protein 1
G3ID64_CRIGR	Vacuolar protein sorting-associated protein 13A (Fragment)
G3H6T8_CRIGR	Vacuolar protein sorting-associated protein 26A
G3H2A5_CRIGR	Vacuolar protein sorting-associated protein 29
G3I8H3_CRIGR	Vacuolar protein sorting-associated protein 35
G3H2U9_CRIGR	Vacuolar protein sorting-associated protein 45
G3HZE9_CRIGR	Valyl-tRNA synthetase
G3HC91_CRIGR	Vascular endothelial growth factor A
G3HS71_CRIGR	Vasorin
G3IMZ0_CRIGR	Very low-density lipoprotein receptor
NSF_CRIGR	Vesicle-fusing ATPase
G3H177_CRIGR	Vesicular integral-membrane protein VIP36
G3H4V0_CRIGR	Vigilin
VIME_CRIGR	Vimentin (Fragment)
G3HHR3_CRIGR	Vimentin
G3GWQ1_CRIGR	Vinculin
G3GUY3_CRIGR	Vinexin (Fragment)
G3GYG0_CRIGR	Vitamin K-dependent protein S
G3ICL7_CRIGR	Voltage-gated potassium channel subunit beta-2
FAM21_CRIGR	WASH complex subunit FAM21
G3IB76_CRIGR	WD repeat-containing protein 6
G3HLR8_CRIGR	WNT1-inducible-signaling pathway protein 2
G3IAG0_CRIGR	Xaa-Pro aminopeptidase 1
G3I4G1_CRIGR	Xanthine dehydrogenase/oxidase
G3I1B9_CRIGR	Xin actin-binding repeat-containing protein 2
G3I281_CRIGR	Zinc finger homeobox protein 3
G3GRU5_CRIGR	Zinc finger protein 469
G3I0Z1_CRIGR	Zinc finger protein 541
KAPCA_CRIGR	cAMP-dependent protein kinase catalytic subunit alpha
G3H853_CRIGR	cAMP-dependent protein kinase type I-alpha regulatory subunit
G3HSQ5_CRIGR	mRNA cap guanine-N7 methyltransferase
G3HC95_CRIGR	tRNA (Cytosine-5-)-methyltransferase NSUN2
G3I7Y9_CRIGR	tRNA guanosine-2'-O-methyltransferase TRM11-like
G3HCJ8_CRIGR	tRNA-dihydrouridine(47) synthase [NAD(P)(+)]
G3H337_CRIGR	tRNA-nucleotidyltransferase 1, mitochondrial
G3HGV7_CRIGR	tRNA-splicing ligase RtcB homolog
G3IC90_CRIGR	von Willebrand factor A domain-containing protein 5A
G3IC91_CRIGR	von Willebrand factor A domain-containing protein 5A
G3IFU4_CRIGR	von Willebrand factor A domain-containing protein 5A
G3IMX9_CRIGR	von Willebrand factor A domain-containing protein 5A
G3GWQ0_CRIGR	40S ribosomal protein S15a
G3GZV3_CRIGR	60S acidic ribosomal protein P2
G3GTY5_CRIGR	60S ribosomal protein L23a
G3H7L1_CRIGR	60S ribosomal protein L23a
G3GVL9_CRIGR	60S ribosomal protein L30
G3GTA4_CRIGR	A disintegrin and metalloproteinase with thrombospondin motifs 7
G3H893_CRIGR	Abhydrolase domain-containing protein 14A
G3GVF0_CRIGR	Beta-1,3-N-acetylglucosaminyltransferase lunatic fringe
G3HEI9_CRIGR	CD276 antigen
G3HPH9_CRIGR	CMP-N-acetylneuraminate-beta-galactosamide-alpha-2, 3-sialyltransferase
G3HTQ5_CRIGR	CMP-N-acetylneuraminate-poly-alpha-2, 8-sialyltransferase
G3GVR5_CRIGR	Cerebral dopamine neurotrophic factor
G3HQN8_CRIGR	Corneodesmosin
G3HUV7_CRIGR	Cysteine-rich motor neuron 1 protein
G3HM68_CRIGR	Cytochrome P450 4F6
G3GSU6_CRIGR	Ectonucleotide pyrophosphatase/phosphodiesterase family member 6
G3HWY2_CRIGR	Exostosin-2
Q80WL0_CRIGR	Gal beta-1,3/4-GlcNAc alpha-2,3-sialyltransferase St3Gal IV
G3IA95_CRIGR	Homeobox protein TGIF1
G3IH68_CRIGR	Integral membrane protein 2C
G3I7Y3_CRIGR	Lysosome-associated membrane glycoprotein 1
G3HVP7_CRIGR	NHL repeat-containing protein 3
G3INI9_CRIGR	NKG2D ligand 1
G3IEU3_CRIGR	Peptidyl-prolyl cis-trans isomerase A
G3I5N9_CRIGR	Peptidyl-prolyl cis-trans isomerase
G3HKY3_CRIGR	Plasma membrane calcium-transporting ATPase 1
G3HHC1_CRIGR	Protein phosphatase 1L
G3HYE5_CRIGR	Protocadherin gamma-C3
G3HS58_CRIGR	Putative E3 ubiquitin-protein ligase MGRN1
G3GZA1_CRIGR	Putative uncharacterized protein (Fragment)
G3HMI0_CRIGR	Semaphorin-3C

G3H9K5_CRIGR	Transmembrane protein 5
G3HUR5_CRIGR	Tsukushin
G3I3V5_CRIGR	Type 2 lactosamine alpha-2,3-sialyltransferase
G3IHB0_CRIGR	14 kDa phosphohistidine phosphatase
G3HYT5_CRIGR	2'-deoxynucleoside 5'-phosphate N-hydrolase 1
G3GYK8_CRIGR	26S protease regulatory subunit 6A
G3GTS6_CRIGR	26S proteasome non-ATPase regulatory subunit 11
G3IND2_CRIGR	26S proteasome non-ATPase regulatory subunit 13
G3I308_CRIGR	26S proteasome non-ATPase regulatory subunit 2
G3I018_CRIGR	26S proteasome non-ATPase regulatory subunit 7
G3GRE0_CRIGR	3'(2'),5'-bisphosphate nucleotidase 1
G3HPP2_CRIGR	4-trimethylaminobutyraldehyde dehydrogenase
RS14_CRIGR	40S ribosomal protein S14
G3HND4_CRIGR	40S ribosomal protein S15a
G3H4D3_CRIGR	6-phosphogluconolactonase
RL13_CRIGR	60S ribosomal protein L13
G3I196_CRIGR	60S ribosomal protein L14
G3GX00_CRIGR	60S ribosomal protein L17
G3H6V1_CRIGR	60S ribosomal protein L17
G3HAK9_CRIGR	60S ribosomal protein L17
G3HMS8_CRIGR	60S ribosomal protein L17
G3HQJ1_CRIGR	60S ribosomal protein L17
G3HPU9_CRIGR	60S ribosomal protein L23a
G3HWZ3_CRIGR	60S ribosomal protein L23a
G3I2N7_CRIGR	60S ribosomal protein L23a
G3GR58_CRIGR	60S ribosomal protein L26
G3HK42_CRIGR	60S ribosomal protein L30
G3HHK3_CRIGR	60S ribosomal protein L31
G3HJT6_CRIGR	60S ribosomal protein L32
G3HFG4_CRIGR	60S ribosomal protein L4
G3HTP4_CRIGR	60S ribosomal protein L4
G3HFK6_CRIGR	60S ribosomal protein L5
G3HAD3_CRIGR	60S ribosomal protein L6
G3HMJ8_CRIGR	60S ribosomal protein L7
G3GSB7_CRIGR	60S ribosomal protein L7a
G3H0N4_CRIGR	60S ribosomal protein L7a
G3H7V4_CRIGR	60S ribosomal protein L7a
G3HVW8_CRIGR	60S ribosomal protein L7a
G3ILW8_CRIGR	60S ribosomal protein L7a
G3IN18_CRIGR	60S ribosomal protein L8
G3GVP7_CRIGR	ADP-sugar pyrophosphatase
G3IG24_CRIGR	ATP-dependent RNA helicase DDX19B
G3IFT9_CRIGR	Actin-related protein 2
G3II56_CRIGR	Actin-related protein 2
G3H2A8_CRIGR	Actin-related protein 2/3 complex subunit 3 (Fragment)
APT_CRIGR	Adenine phosphoribosyltransferase
G3GWQ3_CRIGR	Adenosine kinase
G3I359_CRIGR	Adenosylhomocysteinase
M1ZMR5_CRIGR	Aldehyde oxidase 4
G3I0Z2_CRIGR	Alpha-soluble NSF attachment protein
G3H892_CRIGR	Aminoacylase-1A
G3HG78_CRIGR	Apolipoprotein A-I-binding protein
Q9Z2J2_CRIGR	Apurinic/apyrimidinic endonuclease
G3H5F7_CRIGR	Asparaginyl-tRNA synthetase, cytoplasmic
G3HQ86_CRIGR	BH3-interacting domain death agonist
G3IGN7_CRIGR	BRCA2 and CDKN1A-interacting protein
Q60456_CRIGR	Beta tubulin (Fragment)
G3GXY1_CRIGR	Beta-catenin-like protein 1
G3HY21_CRIGR	Beta-ureidopropionase
G3H2U4_CRIGR	BolA-like protein 1
G3I4G8_CRIGR	BolA-like protein 2
BAIP2_CRIGR	Brain-specific angiogenesis inhibitor 1-associated protein 2
G3GX55_CRIGR	Brain-specific angiogenesis inhibitor 1-associated protein 2
G3HQS5_CRIGR	Bromodomain-containing protein 2
G3HEF9_CRIGR	CB1 cannabinoid receptor-interacting protein 1
G3IA32_CRIGR	COP9 signalosome complex subunit 4
G3H4X7_CRIGR	Calumenin
G3GYY2_CRIGR	Caprin-1
Q924V3_CRIGR	Carbonyl reductase 1
G3IF45_CRIGR	Carbonyl reductase [NADPH] 1
G3I2X3_CRIGR	Casein kinase II subunit alpha'
G3GXH4_CRIGR	Cell division protein kinase 6
G3IDA1_CRIGR	Charged multivesicular body protein 2b (Fragment)
G3H5T7_CRIGR	Chromobox protein-like 1

G3HRP3_CRIGR	Citrate synthase
G3GY31_CRIGR	Cleavage and polyadenylation specificity factor subunit 6
G3I9L6_CRIGR	Coiled-coil domain-containing protein 85A
G3HQH1_CRIGR	Cold shock domain-containing protein E1 (Fragment)
G3HAR2_CRIGR	Copine-1
G3HFZ9_CRIGR	Crk-like protein
G3I8H9_CRIGR	Cysteine and histidine-rich domain-containing protein 1
G3HM70_CRIGR	Cytochrome P450 4F4
G3HM72_CRIGR	Cytochrome P450 4F4
G3IBV7_CRIGR	Cytochrome b5
G3I4G5_CRIGR	Cytoplasmic FMR1-interacting protein 2
G3HCV7_CRIGR	Deoxyhypusine synthase
G3I1Z7_CRIGR	Developmentally-regulated GTP-binding protein 1
G3I277_CRIGR	Dihydrolipoyl dehydrogenase
G3GRT8_CRIGR	Diphosphomevalonate decarboxylase
G3HVC7_CRIGR	Diphthamide biosynthesis protein 1
G3HD21_CRIGR	DnaJ-like subfamily B member 1
MP2K1_CRIGR	Dual specificity mitogen-activated protein kinase kinase 1
G3I9M5_CRIGR	Dynein light chain 1, cytoplasmic
G3I784_CRIGR	Enolase-phosphatase E1
G3HLH2_CRIGR	Envelope glycoprotein
G3HTE6_CRIGR	Eukaryotic initiation factor 4A-III
G3GWM0_CRIGR	Eukaryotic peptide chain release factor GTP-binding subunit ERF3B
G3IMG8_CRIGR	Eukaryotic translation initiation factor 2 subunit 2
G3I7L9_CRIGR	Eukaryotic translation initiation factor 3 subunit C
G3HAU1_CRIGR	Eukaryotic translation initiation factor 3 subunit E
G3I5A2_CRIGR	Exosome complex exonuclease RRP45
G3IFV3_CRIGR	Exportin-1
G3GUX2_CRIGR	Exportin-7 (Fragment)
Q9EQS1_CRIGR	FancG
G3GVT2_CRIGR	Far upstream element-binding protein 1
G3HHX9_CRIGR	Ferritin
G3HNR0_CRIGR	Flavin reductase
OFUT1_CRIGR	GDP-fucose protein O-fucosyltransferase 1
G3H3C9_CRIGR	GTPase KRas
G3HJM5_CRIGR	Gamma-glutamylcyclotransferase
G3I9F0_CRIGR	General vesicular transport factor p115
G3H7C1_CRIGR	Glia maturation factor beta
G3HEL2_CRIGR	Glucosamine-6-phosphate isomerase 1 (Fragment)
G4Y366_CRIGR	Glucose transporter 1
G3IG96_CRIGR	Glutamate--cysteine ligase regulatory subunit
G3GSD8_CRIGR	Glyceraldehyde-3-phosphate dehydrogenase
G3HKA4_CRIGR	Glyceraldehyde-3-phosphate dehydrogenase
G3ID55_CRIGR	Glyceraldehyde-3-phosphate dehydrogenase
G3IHS0_CRIGR	Glyceraldehyde-3-phosphate dehydrogenase
G3HLM3_CRIGR	Glycylpeptide N-tetradecanoyltransferase
G3I369_CRIGR	Golgi apparatus protein 1
Q3HR14_CRIGR	Guanine nucleotide binding protein alpha O
G3H5M8_CRIGR	HEAT repeat-containing protein 3
G3HZK7_CRIGR	Heat shock factor protein 4
G3GU64_CRIGR	Heat shock protein beta-8
G3HBY2_CRIGR	Hematological and neurological expressed 1-like protein
Q6E6J7_CRIGR	Heterochromatin protein 1 beta
G3H8P4_CRIGR	Heterogeneous nuclear ribonucleoprotein A1
G3GTK0_CRIGR	Heterogeneous nuclear ribonucleoprotein A3
G3IJ98_CRIGR	Heterogeneous nuclear ribonucleoprotein A3
G3HKT3_CRIGR	Heterogeneous nuclear ribonucleoprotein F
G3I2K6_CRIGR	Hippocalcin-like protein 1
G3I501_CRIGR	Histone H1.0
G3HDU9_CRIGR	Histone H1.1
G3HDS7_CRIGR	Histone H1.3
G3HPV5_CRIGR	Histone H1.5
G3H4K5_CRIGR	Histone H2A
G3HDV5_CRIGR	Histone H2A
G3HPW0_CRIGR	Histone H2A
G3IK55_CRIGR	Importin subunit alpha
G3HC18_CRIGR	Interleukin enhancer-binding factor 2
G3I1P0_CRIGR	Isochorismatase domain-containing protein 1
G3HUE3_CRIGR	Isocitrate dehydrogenase [NADP] cytoplasmic
G3H450_CRIGR	Isocitrate dehydrogenase [NAD] subunit beta, mitochondrial
G3GRU9_CRIGR	Large neutral amino acids transporter small subunit 1
G3HZ87_CRIGR	Lin-7-like A
G3HVQ6_CRIGR	Low-density lipoprotein receptor
G3HDD5_CRIGR	Magnesium-dependent phosphatase 1

G3HA23_CRIGR	Malate dehydrogenase (Fragment)
G3I837_CRIGR	Mannose-6-phosphate isomerase
G3H7S8_CRIGR	Melanoma-associated antigen D2
G3IIA1_CRIGR	Methylosome protein 50 (Fragment)
G3HYG9_CRIGR	Mitochondrial import inner membrane translocase subunit Tim13
G3HTF0_CRIGR	N-sulphoglucosamine sulphohydrolase
G3HZ25_CRIGR	NADH dehydrogenase [ubiquinone] flavoprotein 2, mitochondrial
G3I0D8_CRIGR	NADH-cytochrome b5 reductase 3
G3GS62_CRIGR	NEDD8-activating enzyme E1 catalytic subunit
G3HAT2_CRIGR	NMDA receptor-regulated protein 1
G3H125_CRIGR	Neutral amino acid transporter A
Q80ZC6_CRIGR	Neutral amino acid transporter type 1
G3I0R3_CRIGR	Nicotinamide phosphoribosyltransferase (Fragment)
G3GTU8_CRIGR	Notchless protein-like 1
G3GSK1_CRIGR	Nuclear cap-binding protein subunit 2
G3GX68_CRIGR	Nuclear protein localization protein 4-like
G3HXV5_CRIGR	Nucleolar phosphoprotein p130
G3HMN4_CRIGR	Omega-amidase NIT2
G3ICW1_CRIGR	Osteoclast-stimulating factor 1
G3IKQ9_CRIGR	Osteopontin
G3II52_CRIGR	Oxysterol-binding protein (Fragment)
G3IMX6_CRIGR	PEST proteolytic signal-containing nuclear protein
G3HZ16_CRIGR	PRKC apoptosis WT1 regulator protein
G3HDG6_CRIGR	Paraspeckle component 1
G3H1W2_CRIGR	Peflin
G3GRE4_CRIGR	Peptidyl-prolyl cis-trans isomerase A
G3I4J9_CRIGR	Peptidyl-prolyl cis-trans isomerase A
G3HHK4_CRIGR	Phosducin-like protein 3
G3GTC9_CRIGR	Phosphoacetylglucosamine mutase
G3I830_CRIGR	Phosphopantothenoylcysteine decarboxylase
G3I0M2_CRIGR	Platelet-activating factor acetylhydrolase IB subunit gamma
G3IE04_CRIGR	Pre-mRNA-processing factor 19
G3IJU9_CRIGR	Pre-mRNA-splicing factor 38B
G3HV50_CRIGR	Prefoldin subunit 5
G3I1Q8_CRIGR	Probable tRNA N6-adenosine threonylcarbamoyltransferase
G3HRM9_CRIGR	Prostaglandin E synthase 3 (Fragment)
G3HGW5_CRIGR	Proteasomal ubiquitin receptor ADRM1
G3HLZ2_CRIGR	Proteasome activator complex subunit 3
G3HCK1_CRIGR	Protein ENL
G3GVR4_CRIGR	Protein FAM107B
G3I563_CRIGR	Protein FAM26D
G3IA76_CRIGR	Protein LZIC
G3IMP5_CRIGR	Protein NDRG1
G3HUU7_CRIGR	Protein S100-A10
G3HC25_CRIGR	Protein S100-A13
G3HRD3_CRIGR	Protein arginine N-methyltransferase 5
G3H1H6_CRIGR	Protein farnesyltransferase/geranylgeranyltransferase type-1 subunit alpha
G3H8M2_CRIGR	Protein quaking (Fragment)
G3HGF9_CRIGR	Protein transport protein Sec23A
G3GWP1_CRIGR	Protein transport protein Sec24C
G3HMR9_CRIGR	Proto-oncogene tyrosine-protein kinase Fyn
G3IJD1_CRIGR	Putative ATP-dependent RNA helicase DDX17
G3HB38_CRIGR	Putative ATP-dependent RNA helicase DDX47
G3HCT4_CRIGR	Putative ATP-dependent RNA helicase DDX5
G3IJD6_CRIGR	Putative D-tyrosyl-tRNA(Tyr) deacylase 2
G3HTI3_CRIGR	Putative G-protein coupled receptor 111
G3GUK0_CRIGR	Putative fructose-2,6-bisphosphatase TIGAR
G3HFG7_CRIGR	Putative hydroxypyruvate isomerase
G3GUM9_CRIGR	Putative ribosomal RNA methyltransferase NOP2
G3HK86_CRIGR	Putative uncharacterized protein
G3HVD1_CRIGR	Putative uncharacterized protein
G3GXC8_CRIGR	Ras-related C3 botulinum toxin substrate 3
G3HSE8_CRIGR	Ras-related protein R-Ras2 (Fragment)
G3H3P1_CRIGR	Ras-related protein Rab-18
G3HWS3_CRIGR	Ras-related protein Rab-2B
G3HRK4_CRIGR	Ras-related protein Rab-5B
G3GV75_CRIGR	Ras-related protein Ral-A
G3GXY4_CRIGR	Regulation of nuclear pre-mRNA domain-containing protein 1B
G3I5L5_CRIGR	Retinal dehydrogenase 1
G3GYJ1_CRIGR	Rho GTPase-activating protein 1
G3HPZ2_CRIGR	S-adenosylmethionine synthase
G3HC68_CRIGR	SHC-transforming protein 1
G3I7U9_CRIGR	Serine protease HTRA2, mitochondrial
G3ILW0_CRIGR	Serine/threonine-protein kinase PAK 1

G3HHD9_CRIGR	Serine/threonine-protein phosphatase 2A 55 kDa regulatory subunit B (Fragment)
G3II02_CRIGR	Serine/threonine-protein phosphatase 2A regulatory subunit B' (Fragment)
G3HZI9_CRIGR	Serine/threonine-protein phosphatase 6 catalytic subunit
G3I4H5_CRIGR	Serine/threonine-protein phosphatase
G3I403_CRIGR	Serine/threonine-protein phosphatase PP1-alpha catalytic subunit
G3HSM4_CRIGR	Small glutamine-rich tetratricopeptide repeat-containing protein alpha
G3HGH4_CRIGR	Small nuclear ribonucleoprotein Sm D1
G3HY23_CRIGR	Small nuclear ribonucleoprotein Sm D3
G3GSY1_CRIGR	Sodium/potassium-transporting ATPase subunit beta-3
G3HFF0_CRIGR	Solute carrier family 2, facilitated glucose transporter member 1 (Fragment)
G3I6H3_CRIGR	Splicing factor 1
G3HHF3_CRIGR	Succinyl-CoA:3-ketoacid-coenzyme A transferase
G3HWB8_CRIGR	Superoxide dismutase
G3I1W1_CRIGR	Synapse-associated protein 1
G3HMA7_CRIGR	Synaptobrevin-like YKT6
G3IES9_CRIGR	T-complex protein 1 subunit eta
G3I5W2_CRIGR	TAR DNA-binding protein 43
G3I8B3_CRIGR	TATA-binding protein-associated factor 2N
G3ILG2_CRIGR	THUMP domain-containing protein 1
G3HE59_CRIGR	TIP41-like protein
G3H9I6_CRIGR	Testin
G3H9C9_CRIGR	Transcription elongation factor B polypeptide 1
G3GVZ1_CRIGR	Transcription factor BTF3-like 4
G3HMC0_CRIGR	Transcriptional activator protein Pur-beta
TSN_CRIGR	Translin
G3GZ54_CRIGR	Transportin-3
G3HS38_CRIGR	Treacle protein
G3GRH9_CRIGR	Tropomodulin-3
G3H5A2_CRIGR	U2 small nuclear ribonucleoprotein A'
G3HY27_CRIGR	U2 small nuclear ribonucleoprotein B''
G3HV49_CRIGR	UPF0160 protein MYG1, mitochondrial
G3HWU6_CRIGR	Ubiquilin-2
G3IL02_CRIGR	Ubiquitin thioesterase OTUB1
G3I3E3_CRIGR	Ubiquitin-conjugating enzyme E2 variant 2
G3HZH2_CRIGR	V-type proton ATPase subunit G 2
G3HSY7_CRIGR	V-type proton ATPase subunit H
G3IE90_CRIGR	Vascular endothelial growth factor receptor 2
G3HP80_CRIGR	Vesicle-trafficking protein SEC22b
G3I853_CRIGR	WD repeat-containing protein 40A (Fragment)
G3ID93_CRIGR	WD repeat-containing protein 5
G3H0B9_CRIGR	WD repeat-containing protein 61
G3I7Q5_CRIGR	Zinc finger protein ZPR1
G3I1J6_CRIGR	[Protein ADP-ribosylarginine] hydrolase
G3HMJ0_CRIGR	cAMP-dependent protein kinase catalytic subunit beta
KAPCB_CRIGR	cAMP-dependent protein kinase catalytic subunit beta
G3ILH4_CRIGR	von Willebrand factor A domain-containing protein 5A
G3ILX2_CRIGR	26S proteasome non-ATPase regulatory subunit 13
G3I4P8_CRIGR	40S ribosomal protein S2
G3I535_CRIGR	40S ribosomal protein S2
G3GSH7_CRIGR	60S ribosomal protein L23a
G3HQ39_CRIGR	60S ribosomal protein L23a
G3HCC0_CRIGR	60S ribosomal protein L27
G3H8Q0_CRIGR	ADP-ribosylation factor 1
G3HNG5_CRIGR	Acyl-coenzyme A thioesterase 1
G3H8F2_CRIGR	Acylamino-acid-releasing enzyme
V5QSN9_CRIGR	Adenylosuccinate synthetase isozyme 2
G3HU24_CRIGR	Aflatoxin B1 aldehyde reductase member 2
G3H4F0_CRIGR	BRCA1-A complex subunit MERIT40
PYRG1_CRIGR	CTP synthase 1 (Fragment)
G3GRB1_CRIGR	Calpain-2 catalytic subunit
G3HUT1_CRIGR	Carbonyl reductase [NADPH] 1
G3HV08_CRIGR	Cell division protein kinase 9
G3ICB6_CRIGR	Elongation factor 1-alpha 1
G3HAN5_CRIGR	Eukaryotic translation initiation factor 2 subunit 2
Q8VHU7_CRIGR	Eukaryotic translation initiation factor 5A isoform II (Fragment)
G3IBA9_CRIGR	Glutamate-rich WD repeat-containing protein 1
G3HZ74_CRIGR	Glutaredoxin-related protein 5
G3I3Y7_CRIGR	Glutathione S-transferase P 2
Q9QVA0_CRIGR	Glutathione S-transferase PI (Fragment)
G3HK68_CRIGR	Glyceraldehyde-3-phosphate dehydrogenase
G3HQR6_CRIGR	H-2 class I histocompatibility antigen, L-D alpha chain
G3H6K5_CRIGR	HIV Tat-specific factor 1-like
G3H942_CRIGR	Histone deacetylase
G3I1G3_CRIGR	Leucine-rich repeat and fibronectin type-III domain-containing protein 2

Q6VVX1_CRIGR	Lipid-transfer protein CERTL
G3I5V6_CRIGR	Mitotic checkpoint protein BUB3
G3GYX1_CRIGR	Nucleolar protein 58
G3HCB0_CRIGR	PC4 and SFRS1-interacting protein
G3GVN0_CRIGR	Phosphomannomutase
G3HCE1_CRIGR	Protein farnesyltransferase subunit beta
G3H0R3_CRIGR	Protein fto
G3I597_CRIGR	Putative histidyl-tRNA synthetase, mitochondrial
G3I7D7_CRIGR	RNA-binding protein FUS
G3I3B5_CRIGR	Rho-related GTP-binding protein RhoG
G3H4N3_CRIGR	RuvB-like 2
G3I2Q1_CRIGR	Serine/threonine-protein phosphatase 2B catalytic subunit beta isoform
G3HTW7_CRIGR	Signal recognition particle 68 kDa protein
F7J0L2_CRIGR	Splicing factor, arginine/serine-rich 1 (Fragment)
G3HSZ4_CRIGR	Splicing factor, arginine/serine-rich 7 (Fragment)
G3I801_CRIGR	Switch-associated protein 70
G3I321_CRIGR	Translation initiation factor eIF-2B subunit epsilon
G3I2G0_CRIGR	UPF0696 protein C11orf68-like
G3HUN1_CRIGR	Ubiquitin carboxyl-terminal hydrolase isozyme L5
G3HA07_CRIGR	Uncharacterized methyltransferase WBSCR22
G3GSP0_CRIGR	Uridine 5'-monophosphate synthase
G3HHY9_CRIGR	V-type proton ATPase subunit G 1
Q91Y61_CRIGR	Ezrin binding protein 50 (Fragment)
G3HVW6_CRIGR	Interleukin-6
G3HZY6_CRIGR	Lin-7-like B
G3I1B8_CRIGR	Phosphoglycerate kinase
G3H667_CRIGR	Polyadenylate-binding protein 1
G3I0C5_CRIGR	Ras-related protein Rab-39A
G3INF1_CRIGR	40S ribosomal protein S26
G3I607_CRIGR	40S ribosomal protein S3
G3GRH2_CRIGR	60S ribosomal protein L23a
G3H6R4_CRIGR	60S ribosomal protein L23a
G3ICC1_CRIGR	60S ribosomal protein L6
Q8R4V6_CRIGR	Acetyl-CoA carboxylase (Fragment)
G3H9D1_CRIGR	Acetyl-CoA carboxylase 1
G3H855_CRIGR	Arylsulfatase G
B4GT3_CRIGR	Beta-1,4-galactosyltransferase 3
G3GY78_CRIGR	DNA-directed RNA polymerase
G3I3F4_CRIGR	GTPase HRas
G3I1V1_CRIGR	Gamma-aminobutyric acid receptor-associated protein-like 1
G3GTD5_CRIGR	Glyceraldehyde-3-phosphate dehydrogenase
G3HJV9_CRIGR	Glyceraldehyde-3-phosphate dehydrogenase
G3ILW7_CRIGR	Glyceraldehyde-3-phosphate dehydrogenase
G3HNP1_CRIGR	GrpE protein homolog
G3I7A0_CRIGR	Guanine nucleotide-binding protein G(T) subunit alpha-2
G3H3H8_CRIGR	Histone H2A
G3HK56_CRIGR	Lysosome-associated membrane glycoprotein 1
G3HYW4_CRIGR	Lysosome-associated membrane glycoprotein 2
G3GWM6_CRIGR	Peptidyl-prolyl cis-trans isomerase
A0PA08_CRIGR	Perlecan (Fragment)
G3HWR4_CRIGR	Plexin-B2
G3IHV7_CRIGR	Poly(RC)-binding protein 2
G3IQ10_CRIGR	Proteasome subunit beta type-3
G3H5G9_CRIGR	Putative uncharacterized protein
G3H8L7_CRIGR	Ras-related protein Rab-5A
SCRB1_CRIGR	Scavenger receptor class B member 1
G3H2Y9_CRIGR	Semaphorin-6C
G3I5T4_CRIGR	Septin-7
G3HMW8_CRIGR	Tubulin beta-6 chain
G3I6I1_CRIGR	26S protease regulatory subunit S10B
G3ILH7_CRIGR	26S proteasome non-ATPase regulatory subunit 1
G3H2Z7_CRIGR	26S proteasome non-ATPase regulatory subunit 4
G3HJA4_CRIGR	26S proteasome non-ATPase regulatory subunit 8
G3I388_CRIGR	28 kDa heat-and acid-stable phosphoprotein
G3H7U0_CRIGR	3-hydroxyacyl-CoA dehydrogenase type-2
G3H3T4_CRIGR	40S ribosomal protein S16
G3II62_CRIGR	40S ribosomal protein S2
G3IGL4_CRIGR	40S ribosomal protein S21
G3H5C9_CRIGR	40S ribosomal protein S27
G3HPZ9_CRIGR	60S ribosomal protein L17
G3HW09_CRIGR	60S ribosomal protein L23
G3I5G8_CRIGR	60S ribosomal protein L23
G3I100_CRIGR	60S ribosomal protein L27
G3I3F0_CRIGR	60S ribosomal protein L32

G3HK69_CRIGR	60S ribosomal protein L6
G3HZP6_CRIGR	60S ribosomal protein L6
G3ICQ8_CRIGR	60S ribosomal protein L7
G3IHP9_CRIGR	60S ribosomal protein L7
G3IIG4_CRIGR	60S ribosomal protein L7
G3GSC3_CRIGR	60S ribosomal protein L7a
G3H4N9_CRIGR	60S ribosomal protein L7a
G3H7D6_CRIGR	60S ribosomal protein L7a
G3HMP4_CRIGR	60S ribosomal protein L7a
G3HTS2_CRIGR	60S ribosomal protein L7a
G3I4R0_CRIGR	60S ribosomal protein L7a
G3IAQ1_CRIGR	60S ribosomal protein L7a
G3IGC1_CRIGR	60S ribosomal protein L7a
G3I3Z1_CRIGR	AH receptor-interacting protein
G3HAE2_CRIGR	ATP synthase subunit alpha, mitochondrial
Q6PW16_CRIGR	ATPase 3 (Fragment)
G3H894_CRIGR	Abhydrolase domain-containing protein 14B
G3H2V3_CRIGR	Acidic leucine-rich nuclear phosphoprotein 32 family member E
G3IIU8_CRIGR	Acyl carrier protein
G3HXP3_CRIGR	Acyl-CoA-binding protein (Fragment)
G3HNG8_CRIGR	Acyl-coenzyme A thioesterase 6
G3I4Q6_CRIGR	Adaptin ear-binding coat-associated protein 1
G3H7M3_CRIGR	Adenylosuccinate synthetase
G3HFP0_CRIGR	Adrenodoxin, mitochondrial
G3H289_CRIGR	Aldehyde dehydrogenase, mitochondrial
G3HIL4_CRIGR	Anamorsin
G3IDF4_CRIGR	Arfaptin-2
G3GW47_CRIGR	Arginyl-tRNA synthetase, cytoplasmic
G3I3Y2_CRIGR	Aspartoacylase-2
G3HXA2_CRIGR	Basic leucine zipper and W2 domain-containing protein 2
A9XHW5_CRIGR	CCHC-type zinc finger (Fragment)
G3I5F0_CRIGR	COP9 signalosome complex subunit 6
G3IHZ2_CRIGR	COP9 signalosome complex subunit 8
G3IF42_CRIGR	Carbonyl reductase [NADPH] 1
G3HZG2_CRIGR	Casein kinase II subunit beta
G3HID6_CRIGR	Cellular nucleic acid-binding protein
G3H2J7_CRIGR	Chromobox protein-like 3
Q6E6J6_CRIGR	Chromobox protein-like 5
G3IE25_CRIGR	Clathrin light chain A
G3HKP5_CRIGR	Collagen alpha-1(VII) chain
G3I3V3_CRIGR	Coproporphyrinogen-III oxidase, mitochondrial
G3I7I0_CRIGR	DCN1-like protein
G3HYU6_CRIGR	DNA-directed RNA polymerases I and III subunit RPAC1
G3I0B8_CRIGR	Dual specificity mitogen-activated protein kinase kinase 1
G3HCF8_CRIGR	Endophilin-A2
G3I0X4_CRIGR	Epoxide hydrolase 1
B5TSQ7_CRIGR	Eukaryotic translation elongation factor 1 alpha 2 (Fragment)
G3HLU5_CRIGR	Eukaryotic translation initiation factor 1
G3HJA9_CRIGR	Eukaryotic translation initiation factor 3 subunit K (Fragment)
G3ICR6_CRIGR	Eukaryotic translation initiation factor 4 gamma 1
G3H375_CRIGR	Eukaryotic translation initiation factor 5
G3IN52_CRIGR	Fumarylacetoacetase
SAR1B_CRIGR	GTP-binding protein SAR1b
G3HS21_CRIGR	GTP:AMP phosphotransferase mitochondrial
G3I4N5_CRIGR	Gamma-aminobutyric acid receptor-associated protein-like 2
G3I6H6_CRIGR	Glucosamine 6-phosphate N-acetyltransferase
G3IL43_CRIGR	Glucosamine-6-phosphate isomerase 2
G3HY07_CRIGR	Glutathione S-transferase theta-2
G3GTD6_CRIGR	Glyceraldehyde-3-phosphate dehydrogenase
G3HY36_CRIGR	Glyceraldehyde-3-phosphate dehydrogenase
G3HYZ5_CRIGR	Glyceraldehyde-3-phosphate dehydrogenase
G3I3S0_CRIGR	Glyceraldehyde-3-phosphate dehydrogenase
G3HPU8_CRIGR	Glycine cleavage system H protein, mitochondrial
G3GVS3_CRIGR	Glycylpeptide N-tetradecanoyltransferase
G3HLM2_CRIGR	Glycylpeptide N-tetradecanoyltransferase
G3HZ81_CRIGR	Glycylpeptide N-tetradecanoyltransferase
G3HZ82_CRIGR	Glycylpeptide N-tetradecanoyltransferase
G3HHM7_CRIGR	Guanylate kinase
G3H2E1_CRIGR	H/ACA ribonucleoprotein complex subunit 2
G3IDC2_CRIGR	Heat shock cognate 71 kDa protein
G3IGQ4_CRIGR	Heat shock cognate 71 kDa protein
G3HB42_CRIGR	Heme-binding protein 1
G3IEG9_CRIGR	Heterogeneous nuclear ribonucleoprotein A3
HMGB1_CRIGR	High mobility group protein B1 (Fragment)

G3H154_CRIGR	Histone H2A
G3IID7_CRIGR	Histone H3.3
G3HSE6_CRIGR	Histone-binding protein RBBP4
G3I334_CRIGR	Hydroxyacyl-coenzyme A dehydrogenase, mitochondrial
G3H8Q6_CRIGR	Importin subunit alpha
G3H951_CRIGR	Importin subunit alpha
G3HHC3_CRIGR	Importin subunit alpha
G3H0B5_CRIGR	Isocitrate dehydrogenase [NAD] subunit alpha, mitochondrial
G3ILE4_CRIGR	Kynurenine--oxoglutarate transaminase 3
G3HPL3_CRIGR	L-lactate dehydrogenase A chain
G3HT05_CRIGR	L-lactate dehydrogenase A chain
G3GXD0_CRIGR	L-xylulose reductase
G3GXR7_CRIGR	Microtubule-associated protein RP/EB family member 3
G3HFC2_CRIGR	Mitochondrial import inner membrane translocase subunit Tim13
G3IMX4_CRIGR	Mitochondrial import inner membrane translocase subunit Tim9
G3IFL2_CRIGR	Multifunctional protein ADE2
G3I2Q4_CRIGR	NEDD8-conjugating enzyme Ubc12
G3HXM0_CRIGR	NFU1 iron-sulfur cluster scaffold-like, mitochondrial
G3IJJ0_CRIGR	NHP2-like protein 1
G3IEX2_CRIGR	Nuclease-sensitive element-binding protein 1
G3H9Y7_CRIGR	NudC domain-containing protein 2
G3IK70_CRIGR	PDZ and LIM domain protein 5
G3GZH9_CRIGR	PEST proteolytic signal-containing nuclear protein
G3GZI0_CRIGR	PEST proteolytic signal-containing nuclear protein
G3HTX6_CRIGR	Phosphoribosyl pyrophosphate synthetase-associated protein 1
G3GVJ8_CRIGR	Phosphoribosyl pyrophosphate synthetase-associated protein 2
G3HA55_CRIGR	Plasminogen activator inhibitor 1
G3HMX7_CRIGR	Polyadenylate-binding protein-interacting protein 1
G3I970_CRIGR	Porphobilinogen deaminase
G3HQ99_CRIGR	Programmed cell death protein 10
G3I0N5_CRIGR	Programmed cell death protein 5
G3IIM4_CRIGR	Proteasome activator complex subunit 3
G3I054_CRIGR	Proteasome assembly chaperone 1
G3IGW1_CRIGR	Protein CTLA-2-beta
G3HED6_CRIGR	Protein Dr1
G3HHV6_CRIGR	Protein FAM98B
G3HLM6_CRIGR	Protein HEXIM1
G3INR2_CRIGR	Protein NDRG1
G3IJB6_CRIGR	Protein RCC2
G3GX44_CRIGR	Protein SEC13-like
G3GR77_CRIGR	Protein SET (Fragment)
G3H938_CRIGR	Protein archease
G3GTN1_CRIGR	Protein mago nashi-like
G3ID73_CRIGR	Putative proline racemase
G3HNE4_CRIGR	Putative ribosomal RNA methyltransferase 1
G3HCP5_CRIGR	Putative uncharacterized protein
G3I9E7_CRIGR	Pyridoxal kinase
G3HS22_CRIGR	RNA 3'-terminal phosphate cyclase-like protein
G3H2S5_CRIGR	RNA-binding protein 8A
G3HG97_CRIGR	Ras-related protein Rab-25
G3HJH9_CRIGR	Replication protein A 14 kDa subunit
G3H0D2_CRIGR	Reticulocalbin-2
G3GYV4_CRIGR	Ribosome biogenesis protein WDR12
Q9QVY2_CRIGR	SAR1B protein promoting vesicle budding from the endoplasmic reticulum
G3IFW7_CRIGR	Serine/threonine-protein phosphatase 2A 55 kDa regulatory subunit B delta isoform
SRRT_CRIGR	Serrate RNA effector molecule homolog (Fragment)
G3H7F9_CRIGR	Sialic acid synthase
G3I9L2_CRIGR	Sialidase-2
G3HGL0_CRIGR	Single-stranded DNA-binding protein
A9XHW4_CRIGR	Sjogren syndrome antigen B (Fragment)
G3HMF9_CRIGR	Small acidic protein
G3HBJ3_CRIGR	Small nuclear ribonucleoprotein F
G3I8L4_CRIGR	Sorting nexin-6 (Fragment)
G3I2M0_CRIGR	Sulfatase-modifying factor 2
G3I5W3_CRIGR	TAR DNA-binding protein 43
G3HDG0_CRIGR	Thiamine-triphosphatase
G3H7R4_CRIGR	Thioredoxin-dependent peroxide reductase, mitochondrial
G3H5G6_CRIGR	Thioredoxin-like protein 1
G3HJ65_CRIGR	Transcription elongation factor B polypeptide 1
G3H2J4_CRIGR	Transcription elongation factor B polypeptide 2
G3IGW5_CRIGR	Transcription elongation factor B polypeptide 2
G3HVX3_CRIGR	Transmembrane glycoprotein NMB
G3HIU2_CRIGR	Transportin-1
G3ICG5_CRIGR	Tripeptidyl-peptidase 2

G3HPR6_CRIGR	Tubulin-folding cofactor B
G3H4A5_CRIGR	U6 snRNA-associated Sm-like protein LSm4
G3I775_CRIGR	UPF0195 protein FAM96B
G3HUQ0_CRIGR	UPF0363 protein C7orf20-like
G3HUE5_CRIGR	UPF0368 protein Cxorf26-like
G3IAS6_CRIGR	Ubiquilin-1
G3HGA1_CRIGR	Ubiquilin-4
G3IIV0_CRIGR	Ubiquitin domain-containing protein UBFD1
G3H1F1_CRIGR	Ubiquitin-conjugating enzyme E2 E1
G3H1F2_CRIGR	Ubiquitin-conjugating enzyme E2 E1
G3HDP9_CRIGR	Ubiquitin-conjugating enzyme E2 E3
G3I3N5_CRIGR	V-type proton ATPase subunit C 1
G3I8N5_CRIGR	V-type proton ATPase subunit E 2
G3GZ95_CRIGR	V-type proton ATPase subunit F
G3HZM3_CRIGR	V-type proton ATPase subunit d 1
G3HAV4_CRIGR	Vacuolar protein sorting-associated protein VTA1-like
G3IQ91_CRIGR	Vesicle-fusing ATPase

Supplementary Table S5. GO analysis of identified HCPs in the culture supernatants during batch culture.

Batch culture_raw data				
Day3_CC				
Term	Count	%	P-Value	Benjamini
intracellular non-membrane-bounded organelle	223	20.2	1.80E-16	3.40E-14
non-membrane-bounded organelle	223	20.2	1.80E-16	3.40E-14
extracellular region	161	14.6	6.60E-06	7.90E-05
cytoskeleton	133	12	2.70E-10	7.40E-09
membrane-enclosed lumen	111	10.1	4.40E-04	3.60E-03
intracellular organelle lumen	106	9.6	8.30E-04	6.40E-03
organelle lumen	106	9.6	9.10E-04	6.90E-03
ribonucleoprotein complex	99	9	8.80E-25	4.00E-22
cytoskeletal part	98	8.9	3.50E-09	8.10E-08
extracellular region part	97	8.8	7.30E-09	1.60E-07
cytosol	93	8.4	5.70E-16	4.20E-14
vesicle	90	8.2	3.70E-16	3.00E-14
cytoplasmic vesicle	89	8.1	3.10E-16	3.80E-14
Golgi apparatus	80	7.2	2.80E-06	4.00E-05
endoplasmic reticulum	80	7.2	2.40E-03	1.60E-02
cytoplasmic membrane-bounded vesicle	77	7	1.50E-15	8.20E-14
membrane-bounded vesicle	77	7	3.30E-15	1.70E-13
cell projection	70	6.3	3.60E-06	4.90E-05
endomembrane system	66	6	4.50E-06	5.90E-05
cell fraction	62	5.6	1.10E-03	8.10E-03
extracellular matrix	55	5	1.50E-10	4.40E-09
proteinaceous extracellular matrix	54	4.9	1.00E-10	3.10E-09
extracellular space	53	4.8	2.90E-03	1.90E-02
vacuole	51	4.6	1.10E-15	7.20E-14
insoluble fraction	50	4.5	2.00E-02	8.80E-02
microtubule cytoskeleton	48	4.3	2.70E-03	1.80E-02
lysosome	46	4.2	8.80E-15	4.00E-13
lytic vacuole	46	4.2	1.10E-14	4.60E-13
internal side of plasma membrane	46	4.2	1.50E-07	2.80E-06
ribosome	45	4.1	7.30E-13	2.60E-11
actin cytoskeleton	45	4.1	7.90E-12	2.60E-10
membrane fraction	45	4.1	7.10E-02	2.50E-01
extrinsic to membrane	43	3.9	5.20E-02	2.00E-01
melanosome	38	3.4	3.00E-21	6.80E-19
pigment granule	38	3.4	3.00E-21	6.80E-19
endoplasmic reticulum part	37	3.4	3.10E-06	4.30E-05
cell surface	37	3.4	1.00E-03	7.50E-03
Golgi apparatus part	34	3.1	3.50E-05	3.60E-04
microtubule	33	3	2.40E-04	2.00E-03
neuron projection	29	2.6	5.60E-03	3.30E-02
endoplasmic reticulum lumen	28	2.5	6.70E-14	2.60E-12
cell cortex	28	2.5	9.20E-08	1.80E-06
extracellular matrix part	26	2.4	1.60E-09	3.90E-08
spliceosome	27	2.4	2.50E-07	4.40E-06
nuclear envelope	27	2.4	1.50E-05	1.70E-04
external side of plasma membrane	25	2.3	7.90E-03	4.40E-02
cell leading edge	23	2.1	6.50E-06	8.20E-05
basement membrane	22	2	1.10E-08	2.30E-07
membrane coat	21	1.9	7.00E-10	1.80E-08
coated membrane	21	1.9	7.00E-10	1.80E-08
contractile fiber	20	1.8	2.10E-05	2.20E-04
cytosolic part	19	1.7	1.40E-07	2.60E-06

cell cortex part	19	1.7	1.50E-06	2.30E-05
contractile fiber part	19	1.7	1.80E-05	1.90E-04
soluble fraction	19	1.7	1.30E-04	1.10E-03
myofibril	18	1.6	1.40E-04	1.20E-03
coated vesicle	18	1.6	4.90E-03	2.90E-02
proteasome complex	17	1.5	1.10E-06	1.80E-05
ribosomal subunit	17	1.5	7.30E-06	8.50E-05
cell soma	17	1.5	6.30E-03	3.60E-02
sarcomere	15	1.4	1.00E-03	7.70E-03
axon	15	1.4	1.50E-02	7.30E-02
apical part of cell	16	1.4	4.70E-02	1.80E-01
Golgi membrane	16	1.4	5.00E-02	1.90E-01
basolateral plasma membrane	16	1.4	6.10E-02	2.20E-01
vesicle membrane	14	1.3	1.50E-02	7.30E-02
ruffle	13	1.2	5.80E-05	5.90E-04
cytoplasmic vesicle membrane	13	1.2	1.60E-02	7.40E-02
cytoplasmic vesicle part	13	1.2	3.00E-02	1.20E-01
nuclear pore	12	1.1	2.00E-03	1.40E-02
pore complex	12	1.1	1.10E-02	5.70E-02
stress fiber	11	1	1.10E-06	1.90E-05
actin filament bundle	11	1	1.80E-06	2.70E-05
actomyosin	11	1	6.50E-06	8.10E-05
clathrin coat	11	1	6.50E-05	6.50E-04
Golgi-associated vesicle	11	1	1.10E-04	1.00E-03
cortical cytoskeleton	11	1	5.40E-04	4.30E-03
I band	11	1	2.90E-03	1.90E-02
myosin complex	11	1	8.30E-03	4.50E-02
vesicle coat	10	0.9	7.00E-05	6.70E-04
small ribosomal subunit	10	0.9	9.50E-05	8.90E-04
coated pit	10	0.9	2.20E-04	1.90E-03
Z disc	10	0.9	3.60E-03	2.20E-02
coated vesicle membrane	10	0.9	9.50E-03	5.10E-02
extrinsic to plasma membrane	10	0.9	1.20E-02	6.30E-02
focal adhesion	10	0.9	1.50E-02	7.30E-02
cell-substrate adherens junction	10	0.9	2.30E-02	1.00E-01
lamellipodium	10	0.9	2.80E-02	1.20E-01
cell-substrate junction	10	0.9	3.60E-02	1.50E-01
microtubule associated complex	10	0.9	7.60E-02	2.70E-01
cytosolic ribosome	9	0.8	1.10E-05	1.30E-04
nuclear periphery	9	0.8	1.80E-02	8.10E-02
AP-type membrane coat adaptor complex	8	0.7	1.10E-03	8.20E-03
clathrin adaptor complex	8	0.7	1.10E-03	8.20E-03
transport vesicle	8	0.7	2.90E-03	1.90E-02
actin filament	8	0.7	3.60E-03	2.20E-02
nuclear membrane	8	0.7	1.40E-02	7.00E-02
large ribosomal subunit	8	0.7	1.60E-02	7.40E-02
chaperonin-containing T-complex	7	0.6	6.90E-07	1.20E-05
eukaryotic translation initiation factor 3 complex	7	0.6	6.80E-05	6.60E-04
septin complex	7	0.6	1.90E-04	1.70E-03
septin cytoskeleton	7	0.6	1.90E-04	1.70E-03
Golgi-associated vesicle membrane	7	0.6	2.30E-03	1.60E-02
cytosolic large ribosomal subunit	5	0.5	6.60E-04	5.20E-03
ciliary rootlet	5	0.5	3.40E-03	2.10E-02
COPI vesicle coat	5	0.5	3.40E-03	2.10E-02
COPI coated vesicle membrane	5	0.5	3.40E-03	2.10E-02
proton-transporting two-sector ATPase complex, catalytic domain	6	0.5	4.70E-03	2.80E-02
COPI-coated vesicle	5	0.5	7.10E-03	4.00E-02

basal lamina	5	0.5	9.70E-03	5.10E-02
neuromuscular junction	6	0.5	1.50E-02	7.30E-02
filamentous actin	5	0.5	1.70E-02	7.60E-02
transport vesicle membrane	5	0.5	2.10E-02	9.30E-02
trans-Golgi network transport vesicle	5	0.5	3.10E-02	1.30E-01
collagen	5	0.5	3.80E-02	1.50E-01
uropod	4	0.4	2.90E-03	1.90E-02
proteasome accessory complex	4	0.4	2.90E-03	1.90E-02
trailing edge	4	0.4	2.90E-03	1.90E-02
nuclear lamina	4	0.4	1.40E-02	7.00E-02
proton-transporting V-type ATPase, V1 domain	4	0.4	1.40E-02	7.00E-02
kinesin complex	4	0.4	4.50E-02	1.80E-01
immunological synapse	4	0.4	5.50E-02	2.10E-01
polysome	4	0.4	6.70E-02	2.40E-01
nuclear inner membrane	4	0.4	8.00E-02	2.70E-01
proteasome activator complex	3	0.3	1.40E-02	6.90E-02
lamin filament	3	0.3	2.60E-02	1.10E-01
vacuolar lumen	3	0.3	4.10E-02	1.60E-01
clathrin coat of coated pit	3	0.3	5.90E-02	2.20E-01
ER to Golgi transport vesicle membrane	3	0.3	7.90E-02	2.70E-01
COPII vesicle coat	3	0.3	7.90E-02	2.70E-01
cell division site	3	0.3	7.90E-02	2.70E-01
striated muscle thin filament	3	0.3	7.90E-02	2.70E-01
cell division site part	3	0.3	7.90E-02	2.70E-01
laminin complex	3	0.3	7.90E-02	2.70E-01
Day3_MF				
Term	Count	%	P-Value	Benjamini
nucleotide binding	253	22.9	7.80E-19	3.50E-16
purine nucleotide binding	211	19.1	6.50E-14	1.20E-11
ribonucleotide binding	205	18.6	5.20E-14	1.20E-11
purine ribonucleotide binding	205	18.6	5.20E-14	1.20E-11
adenyl nucleotide binding	164	14.9	6.00E-09	4.50E-07
purine nucleoside binding	164	14.9	1.10E-08	7.00E-07
nucleoside binding	164	14.9	1.70E-08	1.00E-06
adenyl ribonucleotide binding	158	14.3	5.00E-09	4.10E-07
ATP binding	157	14.2	4.00E-09	3.60E-07
RNA binding	92	8.3	2.80E-10	3.10E-08
structural molecule activity	91	8.2	8.10E-21	7.30E-18
calcium ion binding	87	7.9	1.30E-04	4.10E-03
peptidase activity	69	6.2	1.40E-04	4.20E-03
peptidase activity, acting on L-amino acid peptides	66	6	2.10E-04	5.90E-03
cytoskeletal protein binding	59	5.3	1.70E-07	8.20E-06
GTP binding	52	4.7	4.10E-07	1.80E-05
guanyl nucleotide binding	52	4.7	8.90E-07	3.60E-05
guanyl ribonucleotide binding	52	4.7	8.90E-07	3.60E-05
endopeptidase activity	49	4.4	4.10E-04	1.10E-02
actin binding	47	4.3	7.20E-08	4.10E-06
carbohydrate binding	44	4	1.60E-05	5.90E-04
structural constituent of ribosome	42	3.8	1.10E-14	3.40E-12
GTPase activity	34	3.1	2.20E-11	2.80E-09
ATPase activity	32	2.9	6.60E-03	1.10E-01
ATPase activity, coupled	29	2.6	1.50E-03	3.50E-02
identical protein binding	29	2.6	4.60E-02	4.20E-01
pattern binding	28	2.5	1.40E-07	7.10E-06
polysaccharide binding	28	2.5	1.40E-07	7.10E-06
translation factor activity, nucleic acid binding	26	2.4	7.30E-09	5.10E-07
metallopeptidase activity	26	2.4	1.10E-03	2.70E-02

unfolded protein binding	25	2.3	1.70E-11	2.60E-09
glycosaminoglycan binding	25	2.3	7.20E-07	3.10E-05
protein domain specific binding	25	2.3	3.40E-03	6.50E-02
ligase activity, forming carbon-nitrogen bonds	24	2.2	7.30E-03	1.20E-01
manganese ion binding	22	2	1.30E-03	3.00E-02
motor activity	21	1.9	1.30E-03	3.00E-02
protein homodimerization activity	21	1.9	4.50E-02	4.30E-01
serine-type peptidase activity	21	1.9	9.60E-02	6.50E-01
serine hydrolase activity	21	1.9	1.00E-01	6.60E-01
ligase activity, forming carbon-oxygen bonds	19	1.7	1.10E-09	1.10E-07
aminoacyl-tRNA ligase activity	19	1.7	1.10E-09	1.10E-07
ligase activity, forming aminoacyl-tRNA and related compounds	19	1.7	1.10E-09	1.10E-07
translation initiation factor activity	19	1.7	1.10E-07	5.90E-06
heparin binding	19	1.7	1.10E-05	4.40E-04
helicase activity	19	1.7	3.30E-03	6.50E-02
coenzyme binding	18	1.6	5.00E-02	4.50E-01
growth factor binding	17	1.5	2.50E-05	8.90E-04
growth factor activity	17	1.5	3.40E-02	3.60E-01
metalloendopeptidase activity	16	1.4	1.20E-02	1.70E-01
protein transporter activity	14	1.3	5.00E-04	1.30E-02
exopeptidase activity	14	1.3	8.90E-04	2.30E-02
ATP-dependent helicase activity	14	1.3	5.90E-03	1.00E-01
purine NTP-dependent helicase activity	14	1.3	5.90E-03	1.00E-01
actin filament binding	13	1.2	9.30E-05	3.20E-03
structural constituent of cytoskeleton	10	0.9	9.80E-05	3.30E-03
transferase activity, transferring alkyl or aryl (other than methyl) groups	10	0.9	9.70E-03	1.50E-01
cysteine-type endopeptidase activity	10	0.9	2.80E-02	3.20E-01
rRNA binding	9	0.8	1.40E-04	4.20E-03
extracellular matrix binding	9	0.8	1.90E-04	5.50E-03
ribonucleoprotein binding	9	0.8	1.80E-03	4.00E-02
intramolecular oxidoreductase activity	9	0.8	3.80E-03	7.10E-02
NAD or NADH binding	9	0.8	9.60E-03	1.50E-01
Ras GTPase binding	8	0.7	9.60E-02	6.50E-01
insulin-like growth factor binding	7	0.6	3.10E-03	6.20E-02
integrin binding	7	0.6	6.10E-03	1.00E-01
glutathione transferase activity	7	0.6	7.40E-03	1.20E-01
aminopeptidase activity	7	0.6	1.10E-02	1.60E-01
extracellular matrix structural constituent	7	0.6	1.50E-02	2.00E-01
translation elongation factor activity	7	0.6	1.80E-02	2.30E-01
peptidyl-prolyl cis-trans isomerase activity	7	0.6	3.10E-02	3.40E-01
cis-trans isomerase activity	7	0.6	4.00E-02	3.90E-01
hormone receptor binding	7	0.6	4.90E-02	4.40E-01
intramolecular oxidoreductase activity, interconverting keto- and enol-groups	5	0.5	2.20E-03	4.60E-02
proton-transporting ATPase activity, rotational mechanism	6	0.5	2.60E-03	5.50E-02
intramolecular transferase activity, phosphotransferases	5	0.5	3.40E-03	6.60E-02
ribosome binding	6	0.5	4.80E-03	8.60E-02
tRNA binding	6	0.5	1.00E-02	1.50E-01
intramolecular transferase activity	6	0.5	2.20E-02	2.70E-01
cation-transporting ATPase activity	6	0.5	2.60E-02	3.00E-01
oxidoreductase activity, acting on the CH-NH2 group of donors, oxygen as ac	5	0.5	3.20E-02	3.40E-01
L-ascorbic acid binding	5	0.5	4.50E-02	4.20E-01
oxidoreductase activity, acting on the CH-NH2 group of donors	5	0.5	5.30E-02	4.60E-01
oxidoreductase activity, acting on sulfur group of donors	6	0.5	7.40E-02	5.70E-01
protein serine/threonine phosphatase activity	6	0.5	8.20E-02	6.00E-01
actin-dependent ATPase activity	4	0.4	5.60E-03	9.80E-02
intramolecular oxidoreductase activity, transposing S-S bonds	4	0.4	1.40E-02	1.90E-01
protein disulfide isomerase activity	4	0.4	1.40E-02	1.90E-01

carbon-nitrogen ligase activity, with glutamine as amido-N-donor	4	0.4	2.00E-02	2.50E-01
platelet-derived growth factor binding	4	0.4	2.00E-02	2.50E-01
hydrogen ion transporting ATP synthase activity, rotational mechanism	4	0.4	3.60E-02	3.70E-01
protein kinase C binding	4	0.4	3.60E-02	3.70E-01
sulfuric ester hydrolase activity	4	0.4	5.60E-02	4.70E-01
disulfide oxidoreductase activity	4	0.4	8.00E-02	5.90E-01
mannosidase activity	4	0.4	8.00E-02	5.90E-01
palmitoyl-CoA hydrolase activity	4	0.4	8.00E-02	5.90E-01
hexosaminidase activity	4	0.4	9.40E-02	6.40E-01
phosphoglucomutase activity	3	0.3	1.40E-02	1.90E-01
procollagen-lysine 5-dioxygenase activity	3	0.3	1.40E-02	1.90E-01
proteasome activator activity	3	0.3	2.60E-02	3.00E-01
proteasome regulator activity	3	0.3	2.60E-02	3.00E-01
ADP binding	3	0.3	2.60E-02	3.00E-01
peptidyl-lysine 5-dioxygenase activity	3	0.3	2.60E-02	3.00E-01
serine-type exopeptidase activity	3	0.3	4.10E-02	4.00E-01
GDP-dissociation inhibitor activity	3	0.3	4.10E-02	4.00E-01
serine-type carboxypeptidase activity	3	0.3	4.10E-02	4.00E-01
peroxiredoxin activity	3	0.3	7.90E-02	5.90E-01
microfilament motor activity	3	0.3	7.90E-02	5.90E-01
Day3_BP				
Term	Count	%	P-Value	Benjamini
translation	89	8.1	7.10E-32	2.10E-28
protein folding	36	3.3	2.60E-13	3.80E-10
intracellular transport	69	6.2	2.20E-11	2.20E-08
tRNA aminoacylation	19	1.7	2.90E-10	2.10E-07
tRNA aminoacylation for protein translation	19	1.7	2.90E-10	2.10E-07
amino acid activation	19	1.7	2.90E-10	2.10E-07
actin filament-based process	38	3.4	3.30E-10	1.90E-07
protein localization	96	8.7	9.60E-10	4.70E-07
cellular protein complex assembly	26	2.4	3.30E-08	1.40E-05
actin cytoskeleton organization	33	3	4.00E-08	1.40E-05
establishment of protein localization	82	7.4	4.80E-08	1.50E-05
intracellular protein transport	45	4.1	6.10E-08	1.80E-05
protein transport	81	7.3	7.20E-08	1.90E-05
cellular protein localization	47	4.3	9.10E-08	2.20E-05
cellular macromolecule localization	47	4.3	1.10E-07	2.50E-05
vesicle-mediated transport	62	5.6	3.20E-07	6.60E-05
cellular macromolecular complex assembly	37	3.4	3.60E-07	6.90E-05
ribonucleoside monophosphate biosynthetic process	10	0.9	3.90E-07	7.00E-05
actin filament organization	17	1.5	4.70E-07	8.00E-05
cytoskeleton organization	48	4.3	4.70E-07	7.60E-05
cellular macromolecular complex subunit organization	39	3.5	9.80E-07	1.50E-04
ribonucleoside monophosphate metabolic process	10	0.9	1.30E-06	1.90E-04
RNA splicing	34	3.1	1.40E-06	1.90E-04
regulation of cytoskeleton organization	22	2	1.90E-06	2.50E-04
mRNA metabolic process	44	4	2.00E-06	2.60E-04
cell redox homeostasis	17	1.5	2.10E-06	2.50E-04
protein complex assembly	36	3.3	3.00E-06	3.50E-04
protein complex biogenesis	36	3.3	3.00E-06	3.50E-04
macromolecular complex assembly	47	4.3	3.20E-06	3.50E-04
regulation of actin polymerization or depolymerization	15	1.4	3.20E-06	3.40E-04
regulation of actin filament length	15	1.4	4.10E-06	4.30E-04
mRNA processing	39	3.5	5.10E-06	5.10E-04
macromolecular complex subunit organization	49	4.4	5.90E-06	5.70E-04
regulation of actin cytoskeleton organization	16	1.4	6.70E-06	6.30E-04
purine ribonucleoside monophosphate biosynthetic process	8	0.7	7.10E-06	6.40E-04

purine nucleoside monophosphate biosynthetic process	8	0.7	7.10E-06	6.40E-04
regulation of actin filament-based process	16	1.4	8.30E-06	7.30E-04
regulation of organelle organization	27	2.4	1.10E-05	9.20E-04
ribonucleotide biosynthetic process	22	2	1.30E-05	1.00E-03
membrane invagination	30	2.7	2.10E-05	1.70E-03
endocytosis	30	2.7	2.10E-05	1.70E-03
carbohydrate catabolic process	18	1.6	2.10E-05	1.60E-03
purine nucleoside monophosphate metabolic process	8	0.7	2.40E-05	1.80E-03
purine ribonucleoside monophosphate metabolic process	8	0.7	2.40E-05	1.80E-03
ribonucleotide metabolic process	23	2.1	2.60E-05	1.90E-03
protein polymerization	12	1.1	4.40E-05	3.20E-03
tRNA metabolic process	21	1.9	5.50E-05	3.90E-03
hexose metabolic process	27	2.4	5.70E-05	3.90E-03
nucleobase, nucleoside and nucleotide biosynthetic process	28	2.5	5.80E-05	3.90E-03
nucleobase, nucleoside, nucleotide and nucleic acid biosynthetic process	28	2.5	5.80E-05	3.90E-03
cell adhesion	63	5.7	6.20E-05	4.10E-03
biological adhesion	63	5.7	6.50E-05	4.20E-03
regulation of protein polymerization	14	1.3	7.70E-05	4.90E-03
purine ribonucleotide biosynthetic process	20	1.8	8.00E-05	4.90E-03
regulation of protein complex assembly	15	1.4	8.10E-05	4.90E-03
regulation of protein complex disassembly	12	1.1	9.00E-05	5.30E-03
nucleotide biosynthetic process	27	2.4	9.30E-05	5.40E-03
branching involved in salivary gland morphogenesis	8	0.7	9.80E-05	5.50E-03
regulation of vesicle-mediated transport	17	1.5	1.10E-04	5.90E-03
purine nucleotide biosynthetic process	23	2.1	1.10E-04	5.90E-03
regulation of actin filament depolymerization	9	0.8	1.10E-04	5.90E-03
glucose catabolic process	13	1.2	1.30E-04	6.80E-03
regulation of endocytosis	13	1.2	1.30E-04	6.80E-03
hexose catabolic process	13	1.2	1.30E-04	6.80E-03
cellular carbohydrate catabolic process	14	1.3	1.40E-04	7.00E-03
regulation of actin filament polymerization	12	1.1	1.40E-04	7.10E-03
glutamine metabolic process	8	0.7	1.50E-04	7.30E-03
positive regulation of cellular component organization	21	1.9	1.70E-04	8.10E-03
regulation of cellular component size	25	2.3	1.80E-04	8.60E-03
monosaccharide catabolic process	13	1.2	1.90E-04	8.90E-03
regulation of cellular component biogenesis	17	1.5	2.50E-04	1.20E-02
nitrogen compound biosynthetic process	38	3.4	2.60E-04	1.20E-02
actin filament bundle formation	7	0.6	2.60E-04	1.20E-02
salivary gland morphogenesis	9	0.8	2.80E-04	1.20E-02
neuromuscular process controlling balance	10	0.9	3.00E-04	1.30E-02
vacuole organization	10	0.9	3.00E-04	1.30E-02
glycoprotein metabolic process	23	2.1	3.10E-04	1.30E-02
membrane organization	35	3.2	3.20E-04	1.30E-02
purine ribonucleotide metabolic process	20	1.8	3.40E-04	1.40E-02
extracellular matrix organization	18	1.6	3.60E-04	1.50E-02
cellular homeostasis	41	3.7	4.20E-04	1.70E-02
monosaccharide metabolic process	27	2.4	4.30E-04	1.70E-02
RNA processing	49	4.4	4.70E-04	1.90E-02
cell proliferation	32	2.9	5.30E-04	2.10E-02
homeostatic process	61	5.5	5.70E-04	2.20E-02
nucleocytoplasmic transport	17	1.5	6.00E-04	2.30E-02
nucleoside monophosphate biosynthetic process	11	1	6.40E-04	2.40E-02
nuclear transport	17	1.5	7.60E-04	2.80E-02
salivary gland development	9	0.8	7.70E-04	2.80E-02
extracellular structure organization	22	2	9.40E-04	3.30E-02
purine nucleotide metabolic process	23	2.1	1.00E-03	3.50E-02
ncRNA metabolic process	27	2.4	1.00E-03	3.50E-02

microtubule-based movement	17	1.5	1.10E-03	3.60E-02
alcohol catabolic process	13	1.2	1.10E-03	3.80E-02
IMP metabolic process	4	0.4	1.10E-03	3.80E-02
IMP biosynthetic process	4	0.4	1.10E-03	3.80E-02
cytoskeleton-dependent intracellular transport	9	0.8	1.20E-03	4.00E-02
negative regulation of cellular component organization	16	1.4	1.20E-03	4.10E-02
sulfur metabolic process	16	1.4	1.40E-03	4.50E-02
nuclear import	12	1.1	1.40E-03	4.50E-02
oligosaccharide metabolic process	7	0.6	1.50E-03	4.80E-02
hydrogen peroxide metabolic process	7	0.6	1.50E-03	4.80E-02
muscle organ development	24	2.2	1.60E-03	4.80E-02
negative regulation of protein complex assembly	8	0.7	1.60E-03	4.90E-02
negative regulation of protein polymerization	8	0.7	1.60E-03	4.90E-02
muscle tissue development	20	1.8	1.80E-03	5.40E-02
glutamine family amino acid metabolic process	10	0.9	1.90E-03	5.60E-02
striated muscle tissue development	19	1.7	2.00E-03	5.90E-02
muscle cell differentiation	18	1.6	2.00E-03	5.80E-02
negative regulation of actin filament depolymerization	7	0.6	2.00E-03	5.80E-02
aminoglycan metabolic process	11	1	2.10E-03	6.00E-02
cell projection assembly	13	1.2	2.20E-03	6.20E-02
glycolysis	10	0.9	2.20E-03	6.20E-02
striated muscle cell differentiation	15	1.4	2.30E-03	6.30E-02
protein import into nucleus, docking	6	0.5	2.30E-03	6.30E-02
lysosome organization	7	0.6	2.60E-03	7.10E-02
translational initiation	9	0.8	3.20E-03	8.50E-02
regulation of cell adhesion	15	1.4	3.80E-03	1.00E-01
microtubule-based process	26	2.4	3.90E-03	1.00E-01
negative regulation of cytoskeleton organization	10	0.9	4.10E-03	1.10E-01
nucleic acid transport	12	1.1	4.10E-03	1.00E-01
establishment of RNA localization	12	1.1	4.10E-03	1.00E-01
RNA transport	12	1.1	4.10E-03	1.00E-01
small GTPase mediated signal transduction	30	2.7	4.20E-03	1.10E-01
regulation of protein catabolic process	8	0.7	4.50E-03	1.10E-01
RNA localization	12	1.1	4.60E-03	1.10E-01
glycoprotein biosynthetic process	17	1.5	4.90E-03	1.20E-01
tissue morphogenesis	28	2.5	5.00E-03	1.20E-01
regulation of cell motion	16	1.4	5.00E-03	1.20E-01
regulation of cell morphogenesis	15	1.4	5.10E-03	1.20E-01
positive regulation of protein complex disassembly	4	0.4	5.10E-03	1.20E-01
exocrine system development	9	0.8	5.20E-03	1.20E-01
negative regulation of actin filament polymerization	7	0.6	5.20E-03	1.20E-01
negative regulation of apoptosis	28	2.5	5.30E-03	1.20E-01
nucleoside monophosphate metabolic process	11	1	5.40E-03	1.20E-01
proteolysis	91	8.2	5.50E-03	1.20E-01
glucose metabolic process	19	1.7	5.70E-03	1.30E-01
positive regulation of kinase activity	18	1.6	6.00E-03	1.30E-01
myotube differentiation	5	0.5	6.40E-03	1.40E-01
cellular response to hydrogen peroxide	5	0.5	6.40E-03	1.40E-01
hydrogen peroxide catabolic process	5	0.5	6.40E-03	1.40E-01
gland development	24	2.2	6.50E-03	1.40E-01
negative regulation of programmed cell death	28	2.5	7.00E-03	1.50E-01
cellular component morphogenesis	37	3.4	7.00E-03	1.50E-01
actin filament capping	6	0.5	7.00E-03	1.50E-01
myofibril assembly	6	0.5	7.00E-03	1.50E-01
positive regulation of organelle organization	10	0.9	7.10E-03	1.50E-01
negative regulation of cell death	28	2.5	7.30E-03	1.50E-01
protein targeting	18	1.6	7.50E-03	1.50E-01

angiogenesis	18	1.6	7.50E-03	1.50E-01
negative regulation of protein complex disassembly	8	0.7	7.60E-03	1.50E-01
cytokinesis	7	0.6	7.80E-03	1.60E-01
polysaccharide metabolic process	14	1.3	8.10E-03	1.60E-01
positive regulation of transferase activity	18	1.6	8.70E-03	1.70E-01
localization of cell	31	2.8	8.80E-03	1.70E-01
cell motility	31	2.8	8.80E-03	1.70E-01
regulation of cell cycle process	12	1.1	8.90E-03	1.70E-01
striated muscle cell development	10	0.9	9.10E-03	1.70E-01
regulation of kinase activity	23	2.1	9.50E-03	1.80E-01
nucleobase, nucleoside, nucleotide and nucleic acid transport	12	1.1	9.90E-03	1.90E-01
pigment metabolic process	8	0.7	1.00E-02	1.90E-01
cell migration	27	2.4	1.00E-02	1.90E-01
collagen fibril organization	6	0.5	1.10E-02	2.00E-01
protein import into nucleus	10	0.9	1.10E-02	2.10E-01
regulation of cell shape	9	0.8	1.20E-02	2.10E-01
'de novo' IMP biosynthetic process	3	0.3	1.30E-02	2.30E-01
myoblast proliferation	3	0.3	1.30E-02	2.30E-01
pentose-phosphate shunt	4	0.4	1.30E-02	2.30E-01
regulation of intracellular protein transport	7	0.6	1.30E-02	2.30E-01
cell division	30	2.7	1.30E-02	2.30E-01
blood vessel morphogenesis	23	2.1	1.30E-02	2.30E-01
cell motion	37	3.4	1.40E-02	2.30E-01
positive regulation of molecular function	32	2.9	1.40E-02	2.30E-01
anti-apoptosis	13	1.2	1.40E-02	2.40E-01
regulation of transferase activity	23	2.1	1.40E-02	2.30E-01
cell projection organization	33	3	1.40E-02	2.40E-01
actin filament-based movement	5	0.5	1.50E-02	2.50E-01
mesenchymal cell differentiation	9	0.8	1.50E-02	2.50E-01
glycosaminoglycan metabolic process	8	0.7	1.60E-02	2.50E-01
positive regulation of catalytic activity	28	2.5	1.60E-02	2.60E-01
mesenchyme development	9	0.8	1.70E-02	2.70E-01
regulation of catabolic process	9	0.8	1.70E-02	2.70E-01
neuromuscular process	10	0.9	1.80E-02	2.70E-01
muscle contraction	10	0.9	1.80E-02	2.70E-01
positive regulation of T cell differentiation	7	0.6	1.80E-02	2.80E-01
protein amino acid glycosylation	13	1.2	1.80E-02	2.80E-01
biopolymer glycosylation	13	1.2	1.80E-02	2.80E-01
glycosylation	13	1.2	1.80E-02	2.80E-01
positive regulation of protein kinase activity	16	1.4	1.90E-02	2.80E-01
nicotinamide metabolic process	6	0.5	1.90E-02	2.90E-01
response to hydrogen peroxide	6	0.5	1.90E-02	2.90E-01
alkaloid metabolic process	6	0.5	1.90E-02	2.90E-01
nicotinamide nucleotide metabolic process	6	0.5	1.90E-02	2.90E-01
protein localization in nucleus	10	0.9	2.00E-02	2.90E-01
muscle cell development	10	0.9	2.00E-02	2.90E-01
cofactor metabolic process	21	1.9	2.00E-02	2.90E-01
morphogenesis of a branching structure	16	1.4	2.00E-02	2.90E-01
regulation of intracellular transport	8	0.7	2.00E-02	3.00E-01
posttranscriptional regulation of gene expression	18	1.6	2.00E-02	3.00E-01
proteoglycan metabolic process	7	0.6	2.10E-02	3.00E-01
mRNA transport	10	0.9	2.20E-02	3.10E-01
negative regulation of organelle organization	10	0.9	2.20E-02	3.10E-01
blood vessel development	26	2.4	2.20E-02	3.10E-01
positive regulation of cell adhesion	8	0.7	2.30E-02	3.20E-01
actomyosin structure organization	6	0.5	2.30E-02	3.20E-01
antigen processing and presentation of peptide antigen via MHC class I	5	0.5	2.40E-02	3.30E-01

regulation of epithelial cell proliferation	10	0.9	2.40E-02	3.20E-01
negative regulation of leukocyte activation	9	0.8	2.40E-02	3.20E-01
negative regulation of cell activation	9	0.8	2.40E-02	3.20E-01
positive regulation of endocytosis	7	0.6	2.40E-02	3.20E-01
positive regulation of lymphocyte differentiation	7	0.6	2.40E-02	3.20E-01
gland morphogenesis	12	1.1	2.40E-02	3.20E-01
regulation of protein kinase activity	21	1.9	2.40E-02	3.30E-01
N-glycan processing	3	0.3	2.50E-02	3.30E-01
cell morphogenesis	31	2.8	2.60E-02	3.40E-01
regulation of mitotic cell cycle	13	1.2	2.60E-02	3.40E-01
regulation of apoptosis	50	4.5	2.70E-02	3.50E-01
positive regulation of cytoskeleton organization	6	0.5	2.70E-02	3.50E-01
regulation of nucleocytoplasmic transport	7	0.6	2.70E-02	3.50E-01
regulation of T cell activation	14	1.3	2.80E-02	3.60E-01
cellular response to reactive oxygen species	5	0.5	2.90E-02	3.60E-01
regulation of microtubule-based process	8	0.7	2.90E-02	3.60E-01
vasculature development	26	2.4	2.90E-02	3.60E-01
positive regulation of developmental process	23	2.1	3.00E-02	3.70E-01
negative regulation of immune system process	11	1	3.00E-02	3.60E-01
response to oxidative stress	12	1.1	3.00E-02	3.70E-01
mesoderm formation	7	0.6	3.10E-02	3.70E-01
cellular component assembly involved in morphogenesis	7	0.6	3.10E-02	3.70E-01
positive regulation of cell-substrate adhesion	6	0.5	3.10E-02	3.80E-01
regulation of programmed cell death	50	4.5	3.30E-02	3.90E-01
mannose metabolic process	4	0.4	3.30E-02	3.90E-01
muscle system process	10	0.9	3.40E-02	3.90E-01
regulation of nitric oxide biosynthetic process	5	0.5	3.50E-02	4.00E-01
cardiac muscle tissue development	9	0.8	3.50E-02	4.00E-01
regulation of cell death	50	4.5	3.50E-02	4.00E-01
cell recognition	8	0.7	3.60E-02	4.00E-01
mesenchymal cell development	8	0.7	3.60E-02	4.00E-01
pyridine nucleotide metabolic process	6	0.5	3.60E-02	4.10E-01
antigen processing and presentation of exogenous antigen	6	0.5	3.60E-02	4.10E-01
regulation of lymphocyte differentiation	9	0.8	3.80E-02	4.30E-01
peptide metabolic process	7	0.6	3.90E-02	4.30E-01
mesoderm morphogenesis	7	0.6	3.90E-02	4.30E-01
ruffle organization	3	0.3	3.90E-02	4.30E-01
dichotomous subdivision of terminal units involved in salivary gland branching	3	0.3	3.90E-02	4.30E-01
skeletal myofibril assembly	3	0.3	3.90E-02	4.30E-01
regulation of T cell differentiation	8	0.7	3.90E-02	4.30E-01
positive regulation of signal transduction	19	1.7	4.00E-02	4.30E-01
cell-cell recognition	5	0.5	4.10E-02	4.40E-01
chaperone mediated protein folding requiring cofactor	4	0.4	4.20E-02	4.40E-01
mesoderm development	9	0.8	4.20E-02	4.40E-01
positive regulation of MAP kinase activity	9	0.8	4.20E-02	4.40E-01
response to inorganic substance	12	1.1	4.30E-02	4.50E-01
cellular amide metabolic process	7	0.6	4.40E-02	4.50E-01
cellular component disassembly	7	0.6	4.40E-02	4.50E-01
positive regulation of cell differentiation	19	1.7	4.60E-02	4.70E-01
pigment biosynthetic process	6	0.5	4.70E-02	4.80E-01
proteasomal ubiquitin-dependent protein catabolic process	6	0.5	4.70E-02	4.80E-01
proteasomal protein catabolic process	6	0.5	4.70E-02	4.80E-01
regulation of transforming growth factor beta receptor signaling pathway	6	0.5	4.70E-02	4.80E-01
regulation of heart contraction	8	0.7	4.80E-02	4.80E-01
activation of MAPK activity	8	0.7	4.80E-02	4.80E-01
regulation of phosphorus metabolic process	29	2.6	4.80E-02	4.80E-01
regulation of phosphate metabolic process	29	2.6	4.80E-02	4.80E-01

formation of primary germ layer	7	0.6	4.80E-02	4.80E-01
purine nucleoside triphosphate biosynthetic process	12	1.1	4.90E-02	4.90E-01
positive regulation of cell communication	20	1.8	5.00E-02	4.90E-01
skeletal muscle tissue development	10	0.9	5.00E-02	4.90E-01
regulation of cell cycle	22	2	5.00E-02	4.90E-01
purine nucleoside triphosphate metabolic process	13	1.2	5.10E-02	4.90E-01
regulation of phosphorylation	28	2.5	5.10E-02	4.90E-01
ER-associated protein catabolic process	4	0.4	5.20E-02	4.90E-01
membrane protein proteolysis	4	0.4	5.20E-02	4.90E-01
cortical cytoskeleton organization	4	0.4	5.20E-02	4.90E-01
membrane protein ectodomain proteolysis	4	0.4	5.20E-02	4.90E-01
nucleoside triphosphate biosynthetic process	12	1.1	5.20E-02	5.00E-01
regulation of leukocyte activation	17	1.5	5.30E-02	5.00E-01
positive regulation of epithelial cell proliferation	6	0.5	5.30E-02	5.00E-01
lactation	6	0.5	5.30E-02	5.00E-01
sulfur compound biosynthetic process	7	0.6	5.40E-02	5.00E-01
adult locomotory behavior	9	0.8	5.40E-02	5.00E-01
innate immune response	13	1.2	5.40E-02	5.00E-01
cellular response to oxidative stress	5	0.5	5.60E-02	5.10E-01
purine deoxyribonucleotide metabolic process	3	0.3	5.60E-02	5.10E-01
purine deoxyribonucleoside triphosphate metabolic process	3	0.3	5.60E-02	5.10E-01
cardiac myofibril assembly	3	0.3	5.60E-02	5.10E-01
dichotomous subdivision of an epithelial terminal unit	3	0.3	5.60E-02	5.10E-01
ribosomal small subunit biogenesis	3	0.3	5.60E-02	5.10E-01
glycolipid catabolic process	3	0.3	5.60E-02	5.10E-01
negative regulation of lymphocyte activation	8	0.7	5.70E-02	5.20E-01
gastrulation	10	0.9	5.80E-02	5.20E-01
skeletal muscle organ development	10	0.9	5.80E-02	5.20E-01
regulation of cell activation	17	1.5	5.80E-02	5.20E-01
regulation of lymphocyte activation	16	1.4	5.90E-02	5.20E-01
negative regulation of T cell activation	7	0.6	5.90E-02	5.20E-01
mammary gland development	11	1	6.10E-02	5.30E-01
response to wounding	32	2.9	6.10E-02	5.30E-01
'de novo' protein folding	4	0.4	6.30E-02	5.40E-01
chondroitin sulfate metabolic process	4	0.4	6.30E-02	5.40E-01
cell-substrate junction assembly	4	0.4	6.30E-02	5.40E-01
vascular endothelial growth factor receptor signaling pathway	4	0.4	6.30E-02	5.40E-01
'de novo' posttranslational protein folding	4	0.4	6.30E-02	5.40E-01
NADP metabolic process	4	0.4	6.30E-02	5.40E-01
neural crest cell migration	5	0.5	6.40E-02	5.50E-01
deoxyribonucleotide metabolic process	5	0.5	6.40E-02	5.50E-01
positive regulation of T cell activation	9	0.8	7.30E-02	5.90E-01
hippocampus development	5	0.5	7.30E-02	5.90E-01
positive regulation of nitric oxide biosynthetic process	4	0.4	7.40E-02	6.00E-01
regulation of centrosome cycle	3	0.3	7.50E-02	6.00E-01
L-serine metabolic process	3	0.3	7.50E-02	6.00E-01
positive regulation of mitotic cell cycle	3	0.3	7.50E-02	6.00E-01
regulation of protein localization	11	1	7.80E-02	6.10E-01
nucleoside metabolic process	8	0.7	7.90E-02	6.20E-01
regulation of adaptive immune response	8	0.7	7.90E-02	6.20E-01
regulation of adaptive immune response based on somatic recombination of i	8	0.7	7.90E-02	6.20E-01
antigen processing and presentation of peptide antigen	6	0.5	8.20E-02	6.30E-01
regulation of morphogenesis of a branching structure	5	0.5	8.20E-02	6.30E-01
regulation of phagocytosis	5	0.5	8.20E-02	6.30E-01
nucleoside triphosphate metabolic process	13	1.2	8.30E-02	6.30E-01
cellular cation homeostasis	16	1.4	8.50E-02	6.40E-01
nucleus organization	7	0.6	8.50E-02	6.40E-01

lamellipodium assembly	4	0.4	8.70E-02	6.50E-01
chondroitin sulfate proteoglycan metabolic process	4	0.4	8.70E-02	6.50E-01
negative regulation of transforming growth factor beta receptor signaling path	4	0.4	8.70E-02	6.50E-01
protein amino acid O-linked glycosylation	4	0.4	8.70E-02	6.50E-01
2'-deoxyribonucleotide metabolic process	4	0.4	8.70E-02	6.50E-01
regulation of cell growth	11	1	8.70E-02	6.50E-01
regulation of cell migration	11	1	8.70E-02	6.50E-01
response to reactive oxygen species	6	0.5	9.00E-02	6.50E-01
triglyceride metabolic process	6	0.5	9.00E-02	6.50E-01
regulation of microtubule cytoskeleton organization	6	0.5	9.00E-02	6.50E-01
tissue remodeling	7	0.6	9.20E-02	6.60E-01
oxygen and reactive oxygen species metabolic process	7	0.6	9.20E-02	6.60E-01
branching morphogenesis of a tube	11	1	9.20E-02	6.60E-01
ribonucleoside triphosphate biosynthetic process	11	1	9.20E-02	6.60E-01
purine ribonucleoside triphosphate biosynthetic process	11	1	9.20E-02	6.60E-01
adult behavior	11	1	9.20E-02	6.60E-01
neuron projection development	21	1.9	9.40E-02	6.70E-01
regulation of lipid kinase activity	3	0.3	9.60E-02	6.70E-01
cerebral cortex radially oriented cell migration	3	0.3	9.60E-02	6.70E-01
regulation of protein kinase cascade	16	1.4	9.60E-02	6.70E-01
protein import	10	0.9	9.70E-02	6.80E-01
positive regulation of immune system process	20	1.8	9.70E-02	6.70E-01
positive regulation of lymphocyte activation	11	1	9.80E-02	6.70E-01
immune response	40	3.6	9.80E-02	6.70E-01
Ras protein signal transduction	8	0.7	9.80E-02	6.70E-01
coenzyme metabolic process	15	1.4	9.80E-02	6.70E-01
ATP synthesis coupled proton transport	6	0.5	9.90E-02	6.70E-01
cerebral cortex development	6	0.5	9.90E-02	6.70E-01
energy coupled proton transport, down electrochemical gradient	6	0.5	9.90E-02	6.70E-01

Day5_CC				
Term	Count	%	P-Value	Benjamini
intracellular non-membrane-bounded organelle	224	20	1.70E-16	1.70E-14
non-membrane-bounded organelle	224	20	1.70E-16	1.70E-14
extracellular region	161	14.4	8.50E-06	9.70E-05
cytoskeleton	138	12.3	1.10E-11	4.00E-10
membrane-enclosed lumen	111	9.9	5.10E-04	4.10E-03
intracellular organelle lumen	105	9.4	1.40E-03	1.00E-02
organelle lumen	105	9.4	1.50E-03	1.10E-02
cytoskeletal part	103	9.2	1.00E-10	2.80E-09
ribonucleoprotein complex	99	8.8	1.20E-24	5.60E-22
extracellular region part	97	8.7	9.40E-09	1.90E-07
cytoplasmic vesicle	91	8.1	2.80E-17	4.20E-15
vesicle	91	8.1	1.10E-16	1.20E-14
cytosol	91	8.1	5.80E-15	2.90E-13
cytoplasmic membrane-bounded vesicle	79	7.1	1.20E-16	1.00E-14
membrane-bounded vesicle	79	7.1	3.90E-16	2.80E-14
Golgi apparatus	79	7.1	6.20E-06	7.50E-05
cell projection	73	6.5	5.30E-07	7.90E-06
endoplasmic reticulum	73	6.5	3.20E-02	1.20E-01
endomembrane system	64	5.7	2.00E-05	2.20E-04
cell fraction	60	5.4	3.20E-03	2.00E-02
extracellular matrix	54	4.8	5.50E-10	1.40E-08
extracellular space	54	4.8	1.90E-03	1.30E-02
proteinaceous extracellular matrix	53	4.7	3.80E-10	9.90E-09
vacuole	51	4.6	1.20E-15	6.80E-14

actin cytoskeleton	49	4.4	3.20E-14	1.20E-12
insoluble fraction	48	4.3	4.50E-02	1.60E-01
internal side of plasma membrane	47	4.2	6.60E-08	1.20E-06
microtubule cytoskeleton	47	4.2	4.90E-03	2.80E-02
lysosome	46	4.1	1.00E-14	4.70E-13
lytic vacuole	46	4.1	1.30E-14	5.30E-13
extrinsic to membrane	46	4.1	1.80E-02	7.30E-02
ribosome	43	3.8	1.40E-11	4.60E-10
pigment granule	40	3.6	2.70E-23	6.10E-21
melanosome	40	3.6	2.70E-23	6.10E-21
Golgi apparatus part	36	3.2	6.00E-06	7.50E-05
cell surface	35	3.1	4.00E-03	2.30E-02
endoplasmic reticulum part	34	3	5.40E-05	5.40E-04
microtubule	32	2.9	5.60E-04	4.40E-03
cell cortex	29	2.6	2.60E-08	4.80E-07
neuron projection	29	2.6	6.00E-03	3.20E-02
cell leading edge	28	2.5	7.10E-09	1.60E-07
spliceosome	28	2.5	7.10E-08	1.20E-06
nuclear envelope	26	2.3	4.70E-05	4.80E-04
endoplasmic reticulum lumen	25	2.2	3.40E-11	1.00E-09
extracellular matrix part	25	2.2	9.20E-09	2.00E-07
external side of plasma membrane	23	2.1	2.80E-02	1.10E-01
basement membrane	22	2	1.20E-08	2.30E-07
membrane coat	20	1.8	5.40E-09	1.30E-07
coated membrane	20	1.8	5.40E-09	1.30E-07
cell cortex part	20	1.8	3.40E-07	5.20E-06
Golgi membrane	19	1.7	6.30E-03	3.20E-02
cytosolic part	18	1.6	8.00E-07	1.10E-05
contractile fiber	18	1.6	2.50E-04	2.10E-03
coated vesicle	18	1.6	5.10E-03	2.90E-02
basolateral plasma membrane	18	1.6	1.80E-02	7.40E-02
proteasome complex	17	1.5	1.20E-06	1.60E-05
contractile fiber part	17	1.5	2.40E-04	2.10E-03
soluble fraction	17	1.5	1.20E-03	8.80E-03
cell soma	17	1.5	6.60E-03	3.40E-02
intermediate filament	17	1.5	2.70E-02	1.10E-01
intermediate filament cytoskeleton	17	1.5	3.20E-02	1.20E-01
ruffle	16	1.4	3.30E-07	5.20E-06
ribosomal subunit	16	1.4	3.40E-05	3.50E-04
myofibril	16	1.4	1.40E-03	9.80E-03
axon	16	1.4	6.70E-03	3.40E-02
apical part of cell	15	1.3	8.70E-02	2.70E-01
sarcomere	13	1.2	8.60E-03	4.20E-02
vesicle membrane	14	1.2	1.60E-02	6.90E-02
cytoplasmic vesicle membrane	13	1.2	1.60E-02	7.00E-02
cytoplasmic vesicle part	13	1.2	3.00E-02	1.20E-01
adherens junction	14	1.2	3.10E-02	1.20E-01
anchoring junction	14	1.2	8.30E-02	2.60E-01
Golgi-associated vesicle	12	1.1	2.00E-05	2.10E-04
cortical cytoskeleton	12	1.1	1.20E-04	1.10E-03
focal adhesion	12	1.1	1.60E-03	1.10E-02
nuclear pore	12	1.1	2.10E-03	1.40E-02
cell-substrate adherens junction	12	1.1	2.80E-03	1.80E-02
lamellipodium	12	1.1	3.60E-03	2.10E-02
cell-substrate junction	12	1.1	5.20E-03	2.90E-02
pore complex	12	1.1	1.10E-02	5.20E-02
stress fiber	11	1	1.20E-06	1.60E-05

actin filament bundle	11	1	1.90E-06	2.40E-05
actomyosin	11	1	6.80E-06	8.00E-05
vesicle coat	11	1	9.90E-06	1.10E-04
coated vesicle membrane	11	1	3.00E-03	1.90E-02
extrinsic to plasma membrane	11	1	4.00E-03	2.30E-02
myosin complex	11	1	8.60E-03	4.20E-02
small ribosomal subunit	10	0.9	9.90E-05	9.60E-04
coated pit	10	0.9	2.30E-04	2.00E-03
clathrin coat	10	0.9	3.80E-04	3.10E-03
nuclear periphery	10	0.9	5.80E-03	3.10E-02
I band	10	0.9	9.80E-03	4.60E-02
keratin filament	10	0.9	3.10E-02	1.20E-01
eukaryotic translation initiation factor 3 complex	9	0.8	2.00E-07	3.30E-06
transport vesicle	9	0.8	5.90E-04	4.60E-03
actin filament	9	0.8	7.60E-04	5.70E-03
Z disc	9	0.8	1.30E-02	5.80E-02
cytosolic ribosome	8	0.7	1.20E-04	1.10E-03
Golgi-associated vesicle membrane	8	0.7	3.60E-04	3.10E-03
nuclear membrane	8	0.7	1.40E-02	6.30E-02
site of polarized growth	8	0.7	6.00E-02	2.00E-01
growth cone	8	0.7	6.00E-02	2.00E-01
chaperonin-containing T-complex	7	0.6	7.10E-07	1.00E-05
septin cytoskeleton	7	0.6	2.00E-04	1.80E-03
septin complex	7	0.6	2.00E-04	1.80E-03
proton-transporting two-sector ATPase complex, catalytic domain	7	0.6	6.90E-04	5.30E-03
clathrin adaptor complex	7	0.6	6.10E-03	3.20E-02
AP-type membrane coat adaptor complex	7	0.6	6.10E-03	3.20E-02
large ribosomal subunit	7	0.6	5.00E-02	1.70E-01
nuclear matrix	7	0.6	6.70E-02	2.20E-01
proton-transporting two-sector ATPase complex	7	0.6	6.70E-02	2.20E-01
filamentous actin	6	0.5	2.60E-03	1.70E-02
transport vesicle membrane	6	0.5	3.60E-03	2.20E-02
trans-Golgi network transport vesicle	6	0.5	6.30E-03	3.30E-02
neuromuscular junction	6	0.5	1.50E-02	6.70E-02
endocytic vesicle	6	0.5	4.10E-02	1.50E-01
heterotrimeric G-protein complex	6	0.5	9.10E-02	2.80E-01
proton-transporting V-type ATPase, V1 domain	5	0.4	1.30E-03	9.30E-03
uropod	4	0.4	3.00E-03	1.90E-02
trailing edge	4	0.4	3.00E-03	1.90E-02
COPI coated vesicle membrane	5	0.4	3.40E-03	2.10E-02
COPI vesicle coat	5	0.4	3.40E-03	2.10E-02
clathrin coat of coated pit	4	0.4	5.60E-03	3.10E-02
COPI-coated vesicle	5	0.4	7.20E-03	3.60E-02
cytosolic large ribosomal subunit	4	0.4	9.40E-03	4.50E-02
basal lamina	5	0.4	9.80E-03	4.60E-02
nuclear lamina	4	0.4	1.40E-02	6.30E-02
ciliary rootlet	4	0.4	2.70E-02	1.10E-01
proton-transporting V-type ATPase complex	5	0.4	3.20E-02	1.20E-01
collagen	5	0.4	3.80E-02	1.40E-01
proteasome core complex	5	0.4	4.50E-02	1.60E-01
immunological synapse	4	0.4	5.60E-02	1.90E-01
polysome	4	0.4	6.80E-02	2.20E-01
nuclear inner membrane	4	0.4	8.00E-02	2.60E-01
proteasome activator complex	3	0.3	1.40E-02	6.20E-02
aminoacyl-tRNA synthetase multienzyme complex	3	0.3	1.40E-02	6.20E-02
lamin filament	3	0.3	2.60E-02	1.10E-01
vacuolar lumen	3	0.3	4.20E-02	1.50E-01

proteasome accessory complex	3	0.3	4.20E-02	1.50E-01
Arp2/3 protein complex	3	0.3	5.90E-02	2.00E-01
laminin complex	3	0.3	8.00E-02	2.60E-01
cell division site	3	0.3	8.00E-02	2.60E-01
striated muscle thin filament	3	0.3	8.00E-02	2.60E-01
ER to Golgi transport vesicle membrane	3	0.3	8.00E-02	2.60E-01
clathrin coat of trans-Golgi network vesicle	3	0.3	8.00E-02	2.60E-01
cell division site part	3	0.3	8.00E-02	2.60E-01
COPII vesicle coat	3	0.3	8.00E-02	2.60E-01
Day5_MF				
Term	Count	%	P-Value	Benjamini
nucleotide binding	256	22.9	1.90E-19	8.60E-17
purine nucleotide binding	210	18.8	2.20E-13	4.00E-11
purine ribonucleotide binding	204	18.2	1.80E-13	5.30E-11
ribonucleotide binding	204	18.2	1.80E-13	5.30E-11
adenyl nucleotide binding	161	14.4	4.50E-08	2.90E-06
purine nucleoside binding	161	14.4	8.00E-08	4.50E-06
nucleoside binding	161	14.4	1.20E-07	6.10E-06
adenyl ribonucleotide binding	155	13.8	4.00E-08	2.80E-06
ATP binding	153	13.7	5.50E-08	3.30E-06
structural molecule activity	94	8.4	2.30E-22	2.10E-19
RNA binding	92	8.2	3.70E-10	4.10E-08
calcium ion binding	83	7.4	9.70E-04	2.30E-02
peptidase activity	68	6.1	2.70E-04	8.20E-03
peptidase activity, acting on L-amino acid peptides	65	5.8	4.20E-04	1.20E-02
cytoskeletal protein binding	59	5.3	2.10E-07	9.40E-06
GTP binding	54	4.8	8.10E-08	4.30E-06
guanyl nucleotide binding	54	4.8	1.90E-07	8.80E-06
guanyl ribonucleotide binding	54	4.8	1.90E-07	8.80E-06
actin binding	48	4.3	3.10E-08	2.30E-06
endopeptidase activity	48	4.3	8.30E-04	2.10E-02
carbohydrate binding	43	3.8	3.90E-05	1.30E-03
structural constituent of ribosome	40	3.6	3.60E-13	4.60E-11
GTPase activity	37	3.3	1.80E-13	4.00E-11
ATPase activity	33	2.9	3.90E-03	7.60E-02
ATPase activity, coupled	30	2.7	8.00E-04	2.10E-02
unfolded protein binding	27	2.4	3.20E-13	4.80E-11
translation factor activity, nucleic acid binding	27	2.4	1.70E-09	1.50E-07
pattern binding	27	2.4	5.80E-07	2.50E-05
polysaccharide binding	27	2.4	5.80E-07	2.50E-05
metallopeptidase activity	26	2.3	1.20E-03	2.70E-02
glycosaminoglycan binding	25	2.2	7.90E-07	3.20E-05
protein domain specific binding	24	2.1	7.30E-03	1.20E-01
ligase activity, forming carbon-nitrogen bonds	22	2	2.70E-02	3.10E-01
translation initiation factor activity	20	1.8	2.00E-08	1.60E-06
manganese ion binding	20	1.8	6.80E-03	1.10E-01
ligase activity, forming carbon-oxygen bonds	19	1.7	1.20E-09	1.20E-07
aminoacyl-tRNA ligase activity	19	1.7	1.20E-09	1.20E-07
ligase activity, forming aminoacyl-tRNA and related compounds	19	1.7	1.20E-09	1.20E-07
heparin binding	19	1.7	1.20E-05	4.50E-04
helicase activity	19	1.7	3.50E-03	6.90E-02
motor activity	19	1.7	7.10E-03	1.20E-01
growth factor binding	18	1.6	6.30E-06	2.50E-04
coenzyme binding	17	1.5	9.00E-02	6.50E-01
metalloendopeptidase activity	16	1.4	1.20E-02	1.70E-01
actin filament binding	14	1.2	2.00E-05	7.30E-04
protein transporter activity	14	1.2	5.20E-04	1.40E-02

exopeptidase activity	14	1.2	9.40E-04	2.30E-02
ATP-dependent helicase activity	14	1.2	6.20E-03	1.10E-01
purine NTP-dependent helicase activity	14	1.2	6.20E-03	1.10E-01
intramolecular oxidoreductase activity	11	1	2.00E-04	6.10E-03
extracellular matrix binding	10	0.9	2.70E-05	9.30E-04
structural constituent of cytoskeleton	10	0.9	1.00E-04	3.30E-03
transferase activity, transferring alkyl or aryl (other than methyl) groups	10	0.9	1.00E-02	1.50E-01
cysteine-type endopeptidase activity	10	0.9	2.90E-02	3.30E-01
ATPase activity, coupled to transmembrane movement of ions	10	0.9	4.80E-02	4.60E-01
ribonucleoprotein binding	9	0.8	1.90E-03	4.20E-02
NAD or NADH binding	9	0.8	9.90E-03	1.50E-01
rRNA binding	8	0.7	9.30E-04	2.30E-02
Ras GTPase binding	8	0.7	9.80E-02	6.80E-01
proton-transporting ATPase activity, rotational mechanism	7	0.6	3.20E-04	9.30E-03
insulin-like growth factor binding	7	0.6	3.10E-03	6.50E-02
cation-transporting ATPase activity	7	0.6	6.20E-03	1.10E-01
integrin binding	7	0.6	6.20E-03	1.10E-01
glutathione transferase activity	7	0.6	7.60E-03	1.20E-01
aminopeptidase activity	7	0.6	1.10E-02	1.60E-01
translation elongation factor activity	7	0.6	1.80E-02	2.30E-01
hormone receptor binding	7	0.6	5.10E-02	4.70E-01
ribosome binding	6	0.5	4.90E-03	9.20E-02
tRNA binding	6	0.5	1.00E-02	1.50E-01
intramolecular transferase activity	6	0.5	2.20E-02	2.70E-01
extracellular matrix structural constituent	6	0.5	5.40E-02	4.90E-01
oxidoreductase activity, acting on sulfur group of donors	6	0.5	7.50E-02	6.00E-01
intramolecular oxidoreductase activity, interconverting keto- and enol-groups	5	0.4	2.20E-03	4.70E-02
platelet-derived growth factor binding	5	0.4	2.20E-03	4.70E-02
intramolecular transferase activity, phosphotransferases	5	0.4	3.50E-03	7.00E-02
hydrogen ion transporting ATP synthase activity, rotational mechanism	5	0.4	5.10E-03	9.40E-02
actin-dependent ATPase activity	4	0.4	5.70E-03	1.00E-01
intramolecular oxidoreductase activity, transposing S-S bonds	4	0.4	1.40E-02	1.90E-01
protein disulfide isomerase activity	4	0.4	1.40E-02	1.90E-01
intramolecular oxidoreductase activity, interconverting aldoses and ketoses	4	0.4	2.00E-02	2.60E-01
oxidoreductase activity, acting on the CH-NH2 group of donors, oxygen as ac	5	0.4	3.20E-02	3.50E-01
protein kinase C binding	4	0.4	3.60E-02	3.80E-01
L-ascorbic acid binding	5	0.4	4.60E-02	4.50E-01
threonine-type peptidase activity	5	0.4	5.40E-02	4.90E-01
threonine-type endopeptidase activity	5	0.4	5.40E-02	4.90E-01
oxidoreductase activity, acting on the CH-NH2 group of donors	5	0.4	5.40E-02	4.90E-01
sulfuric ester hydrolase activity	4	0.4	5.70E-02	5.00E-01
mannosidase activity	4	0.4	8.10E-02	6.20E-01
disulfide oxidoreductase activity	4	0.4	8.10E-02	6.20E-01
hexosaminidase activity	4	0.4	9.50E-02	6.70E-01
procollagen-lysine 5-dioxygenase activity	3	0.3	1.40E-02	1.90E-01
phosphoglucomutase activity	3	0.3	1.40E-02	1.90E-01
proteasome regulator activity	3	0.3	2.60E-02	3.10E-01
proteasome activator activity	3	0.3	2.60E-02	3.10E-01
peptidyl-lysine 5-dioxygenase activity	3	0.3	2.60E-02	3.10E-01
serine-type carboxypeptidase activity	3	0.3	4.20E-02	4.20E-01
serine-type exopeptidase activity	3	0.3	4.20E-02	4.20E-01
GDP-dissociation inhibitor activity	3	0.3	4.20E-02	4.20E-01
peroxiredoxin activity	3	0.3	8.00E-02	6.20E-01
microfilament motor activity	3	0.3	8.00E-02	6.20E-01
Day5_BP				
Term	Count	%	P-Value	Benjamini
protein localization	97	8.7	5.90E-10	2.80E-07

proteolysis	92	8.2	4.60E-03	1.20E-01
translation	89	7.9	1.10E-31	3.10E-28
establishment of protein localization	83	7.4	2.90E-08	1.20E-05
protein transport	82	7.3	4.30E-08	1.40E-05
intracellular transport	67	6	2.10E-10	2.00E-07
cell adhesion	66	5.9	1.10E-05	9.50E-04
biological adhesion	66	5.9	1.20E-05	9.50E-04
vesicle-mediated transport	63	5.6	1.70E-07	3.30E-05
homeostatic process	61	5.4	6.50E-04	2.60E-02
macromolecular complex subunit organization	51	4.6	1.40E-06	1.50E-04
RNA processing	50	4.5	2.90E-04	1.30E-02
macromolecular complex assembly	49	4.4	6.60E-07	7.80E-05
cytoskeleton organization	48	4.3	5.50E-07	6.90E-05
regulation of apoptosis	48	4.3	5.70E-02	5.50E-01
regulation of programmed cell death	48	4.3	6.60E-02	5.80E-01
regulation of cell death	48	4.3	7.20E-02	6.10E-01
cellular protein localization	46	4.1	2.80E-07	4.50E-05
cellular macromolecule localization	46	4.1	3.40E-07	4.90E-05
mRNA metabolic process	45	4	9.60E-07	1.10E-04
intracellular protein transport	44	3.9	2.00E-07	3.50E-05
cellular macromolecular complex subunit organization	41	3.7	1.50E-07	3.00E-05
mRNA processing	40	3.6	2.30E-06	2.40E-04
cellular homeostasis	40	3.6	9.00E-04	3.30E-02
cellular macromolecular complex assembly	39	3.5	4.50E-08	1.20E-05
nitrogen compound biosynthetic process	39	3.5	1.40E-04	7.60E-03
actin filament-based process	38	3.4	3.80E-10	2.20E-07
cell motion	38	3.4	9.00E-03	1.80E-01
cellular component morphogenesis	37	3.3	7.60E-03	1.70E-01
protein complex assembly	36	3.2	3.40E-06	3.30E-04
protein complex biogenesis	36	3.2	3.40E-06	3.30E-04
protein folding	35	3.1	1.70E-12	2.40E-09
RNA splicing	34	3	1.60E-06	1.70E-04
cell proliferation	34	3	1.20E-04	6.70E-03
membrane organization	34	3	7.20E-04	2.80E-02
cell projection organization	34	3	9.10E-03	1.80E-01
actin cytoskeleton organization	33	2.9	4.50E-08	1.30E-05
positive regulation of molecular function	33	2.9	8.70E-03	1.80E-01
cell motility	31	2.8	9.50E-03	1.80E-01
localization of cell	31	2.8	9.50E-03	1.80E-01
cell morphogenesis	31	2.8	2.70E-02	3.70E-01
response to wounding	31	2.8	9.50E-02	6.80E-01
small GTPase mediated signal transduction	29	2.6	8.40E-03	1.70E-01
positive regulation of catalytic activity	29	2.6	9.80E-03	1.90E-01
generation of precursor metabolites and energy	29	2.6	9.80E-03	1.90E-01
regulation of organelle organization	28	2.5	3.90E-06	3.70E-04
nucleobase, nucleoside, nucleotide and nucleic acid biosynthetic process	28	2.5	6.40E-05	3.90E-03
nucleobase, nucleoside and nucleotide biosynthetic process	28	2.5	6.40E-05	3.90E-03
endocytosis	28	2.5	1.50E-04	7.70E-03
membrane invagination	28	2.5	1.50E-04	7.70E-03
tissue morphogenesis	28	2.5	5.40E-03	1.30E-01
negative regulation of apoptosis	28	2.5	5.70E-03	1.40E-01
negative regulation of programmed cell death	28	2.5	7.50E-03	1.60E-01
negative regulation of cell death	28	2.5	7.90E-03	1.70E-01
cell division	28	2.5	3.90E-02	4.60E-01
regulation of cellular component size	27	2.4	2.70E-05	1.80E-03
hexose metabolic process	27	2.4	6.20E-05	3.90E-03
monosaccharide metabolic process	27	2.4	4.60E-04	1.90E-02

neuron development	27	2.4	8.80E-02	6.60E-01
cellular protein complex assembly	26	2.3	3.70E-08	1.30E-05
nucleotide biosynthetic process	26	2.3	2.60E-04	1.20E-02
ncRNA metabolic process	26	2.3	2.30E-03	6.70E-02
cell migration	26	2.3	2.00E-02	3.10E-01
microtubule-based process	25	2.2	8.10E-03	1.70E-01
in utero embryonic development	25	2.2	9.00E-02	6.70E-01
regulation of cytoskeleton organization	23	2.1	5.10E-07	6.60E-05
ribonucleotide metabolic process	23	2.1	2.80E-05	1.80E-03
purine nucleotide biosynthetic process	23	2.1	1.20E-04	6.60E-03
extracellular structure organization	24	2.1	1.50E-04	7.80E-03
purine nucleotide metabolic process	23	2.1	1.10E-03	3.80E-02
muscle organ development	24	2.1	1.70E-03	5.30E-02
positive regulation of developmental process	23	2.1	3.10E-02	4.00E-01
regulation of growth	24	2.1	9.60E-02	6.80E-01
blood vessel development	23	2.1	9.90E-02	6.90E-01
ribonucleotide biosynthetic process	22	2	1.40E-05	1.10E-03
glycoprotein metabolic process	22	2	8.40E-04	3.10E-02
regulation of kinase activity	22	2	1.90E-02	3.00E-01
gland development	22	2	2.50E-02	3.50E-01
regulation of transferase activity	22	2	2.70E-02	3.70E-01
neuron projection development	22	2	6.20E-02	5.60E-01
positive regulation of cellular component organization	21	1.9	1.80E-04	8.90E-03
purine ribonucleotide biosynthetic process	20	1.8	8.60E-05	5.00E-03
tRNA metabolic process	20	1.8	1.80E-04	8.90E-03
purine ribonucleotide metabolic process	20	1.8	3.60E-04	1.60E-02
muscle tissue development	20	1.8	1.90E-03	5.80E-02
regulation of protein kinase activity	20	1.8	4.50E-02	4.90E-01
positive regulation of cell communication	20	1.8	5.20E-02	5.20E-01
blood vessel morphogenesis	20	1.8	7.50E-02	6.20E-01
amino acid activation	19	1.7	3.20E-10	2.30E-07
tRNA aminoacylation	19	1.7	3.20E-10	2.30E-07
tRNA aminoacylation for protein translation	19	1.7	3.20E-10	2.30E-07
striated muscle tissue development	19	1.7	2.10E-03	6.30E-02
glucose metabolic process	19	1.7	6.00E-03	1.40E-01
positive regulation of signal transduction	19	1.7	4.10E-02	4.70E-01
positive regulation of cell differentiation	19	1.7	4.80E-02	5.00E-01
transmembrane receptor protein tyrosine kinase signaling pathway	19	1.7	9.70E-02	6.80E-01
regulation of actin cytoskeleton organization	18	1.6	2.60E-07	4.30E-05
regulation of actin filament-based process	18	1.6	3.30E-07	5.00E-05
carbohydrate catabolic process	18	1.6	2.20E-05	1.50E-03
regulation of cellular component biogenesis	18	1.6	7.80E-05	4.70E-03
extracellular matrix organization	18	1.6	3.90E-04	1.60E-02
positive regulation of kinase activity	18	1.6	6.30E-03	1.50E-01
protein targeting	18	1.6	7.90E-03	1.70E-01
positive regulation of transferase activity	18	1.6	9.20E-03	1.80E-01
posttranscriptional regulation of gene expression	18	1.6	2.10E-02	3.20E-01
regulation of actin polymerization or depolymerization	17	1.5	8.80E-08	2.10E-05
regulation of actin filament length	17	1.5	1.20E-07	2.60E-05
actin filament organization	17	1.5	5.10E-07	6.90E-05
negative regulation of cellular component organization	17	1.5	4.40E-04	1.80E-02
microtubule-based movement	17	1.5	1.10E-03	3.90E-02
regulation of cell motion	17	1.5	2.10E-03	6.30E-02
muscle cell differentiation	17	1.5	5.20E-03	1.30E-01
regulation of cell development	17	1.5	7.00E-02	6.00E-01
cell redox homeostasis	16	1.4	1.10E-05	9.30E-04
regulation of protein complex assembly	16	1.4	2.00E-05	1.40E-03

striated muscle cell differentiation	16	1.4	8.20E-04	3.10E-02
sulfur metabolic process	16	1.4	1.50E-03	4.80E-02
regulation of cell adhesion	16	1.4	1.50E-03	4.80E-02
glycoprotein biosynthetic process	16	1.4	1.20E-02	2.20E-01
positive regulation of protein kinase activity	16	1.4	1.90E-02	3.00E-01
cellular cation homeostasis	16	1.4	8.80E-02	6.60E-01
regulation of protein polymerization	15	1.3	1.80E-05	1.30E-03
nucleocytoplasmic transport	15	1.3	4.80E-03	1.20E-01
regulation of cell morphogenesis	15	1.3	5.30E-03	1.30E-01
nuclear transport	15	1.3	5.80E-03	1.40E-01
morphogenesis of a branching structure	15	1.3	4.20E-02	4.70E-01
angiogenesis	15	1.3	6.40E-02	5.70E-01
carboxylic acid biosynthetic process	15	1.3	9.30E-02	6.80E-01
organic acid biosynthetic process	15	1.3	9.30E-02	6.80E-01
regulation of actin filament polymerization	14	1.2	5.20E-06	4.80E-04
regulation of protein complex disassembly	13	1.2	1.80E-05	1.30E-03
cellular carbohydrate catabolic process	14	1.2	1.40E-04	7.50E-03
monosaccharide catabolic process	13	1.2	2.00E-04	9.60E-03
alcohol catabolic process	13	1.2	1.20E-03	3.90E-02
cell projection assembly	13	1.2	2.30E-03	6.70E-02
regulation of vesicle-mediated transport	14	1.2	3.50E-03	9.40E-02
polysaccharide metabolic process	14	1.2	8.50E-03	1.70E-01
anti-apoptosis	13	1.2	1.50E-02	2.50E-01
purine nucleoside triphosphate biosynthetic process	13	1.2	2.40E-02	3.40E-01
nucleoside triphosphate biosynthetic process	13	1.2	2.50E-02	3.50E-01
purine nucleoside triphosphate metabolic process	14	1.2	2.60E-02	3.50E-01
nucleoside triphosphate metabolic process	14	1.2	4.50E-02	4.90E-01
innate immune response	13	1.2	5.60E-02	5.40E-01
protein polymerization	12	1.1	4.60E-05	2.90E-03
glucose catabolic process	12	1.1	5.70E-04	2.30E-02
hexose catabolic process	12	1.1	5.70E-04	2.30E-02
protein amino acid glycosylation	12	1.1	4.20E-02	4.70E-01
biopolymer glycosylation	12	1.1	4.20E-02	4.70E-01
glycosylation	12	1.1	4.20E-02	4.70E-01
purine ribonucleoside triphosphate biosynthetic process	12	1.1	4.80E-02	5.00E-01
ribonucleoside triphosphate biosynthetic process	12	1.1	4.80E-02	5.00E-01
regulation of mitotic cell cycle	12	1.1	5.80E-02	5.50E-01
developmental growth	12	1.1	7.30E-02	6.10E-01
purine ribonucleoside triphosphate metabolic process	12	1.1	7.70E-02	6.20E-01
ribonucleoside triphosphate metabolic process	12	1.1	8.10E-02	6.40E-01
negative regulation of cytoskeleton organization	11	1	1.10E-03	3.90E-02
regulation of endocytosis	11	1	2.20E-03	6.40E-02
striated muscle cell development	11	1	2.90E-03	8.00E-02
nuclear import	11	1	5.00E-03	1.30E-01
muscle cell development	11	1	7.10E-03	1.60E-01
negative regulation of organelle organization	11	1	8.00E-03	1.70E-01
nucleic acid transport	11	1	1.20E-02	2.20E-01
establishment of RNA localization	11	1	1.20E-02	2.20E-01
RNA transport	11	1	1.20E-02	2.20E-01
RNA localization	11	1	1.40E-02	2.40E-01
regulation of cell cycle process	11	1	2.40E-02	3.40E-01
nucleobase, nucleoside, nucleotide and nucleic acid transport	11	1	2.60E-02	3.60E-01
gland morphogenesis	11	1	5.50E-02	5.40E-01
regulation of cell growth	11	1	9.00E-02	6.70E-01
regulation of cell migration	11	1	9.00E-02	6.70E-01
branching morphogenesis of a tube	11	1	9.50E-02	6.80E-01
adult behavior	11	1	9.50E-02	6.80E-01

regulation of actin filament depolymerization	10	0.9	1.40E-05	1.10E-03
neuromuscular process controlling balance	10	0.9	3.10E-04	1.40E-02
vacuole organization	10	0.9	3.10E-04	1.40E-02
translational initiation	10	0.9	7.50E-04	2.90E-02
glycolysis	10	0.9	2.30E-03	6.70E-02
nucleoside monophosphate biosynthetic process	10	0.9	2.70E-03	7.50E-02
positive regulation of organelle organization	10	0.9	7.30E-03	1.60E-01
aminoglycan metabolic process	10	0.9	7.30E-03	1.60E-01
protein import into nucleus	10	0.9	1.20E-02	2.20E-01
nucleoside monophosphate metabolic process	10	0.9	1.60E-02	2.70E-01
neuromuscular process	10	0.9	1.80E-02	2.90E-01
muscle contraction	10	0.9	1.80E-02	2.90E-01
protein localization in nucleus	10	0.9	2.00E-02	3.10E-01
muscle system process	10	0.9	3.50E-02	4.30E-01
skeletal muscle tissue development	10	0.9	5.20E-02	5.20E-01
gastrulation	10	0.9	6.00E-02	5.60E-01
skeletal muscle organ development	10	0.9	6.00E-02	5.60E-01
ATP biosynthetic process	10	0.9	9.90E-02	6.90E-01
protein import	10	0.9	9.90E-02	6.90E-01
ribonucleoside monophosphate biosynthetic process	9	0.8	5.80E-06	5.10E-04
ribonucleoside monophosphate metabolic process	9	0.8	1.60E-05	1.20E-03
negative regulation of protein complex assembly	9	0.8	2.90E-04	1.30E-02
negative regulation of protein polymerization	9	0.8	2.90E-04	1.30E-02
negative regulation of protein complex disassembly	9	0.8	1.90E-03	5.90E-02
positive regulation of cell adhesion	9	0.8	7.20E-03	1.60E-01
regulation of cell shape	9	0.8	1.20E-02	2.20E-01
mesenchymal cell differentiation	9	0.8	1.60E-02	2.70E-01
mesenchyme development	9	0.8	1.80E-02	2.90E-01
nucleoside metabolic process	9	0.8	3.30E-02	4.10E-01
cardiac muscle tissue development	9	0.8	3.60E-02	4.30E-01
mesoderm development	9	0.8	4.30E-02	4.80E-01
positive regulation of MAP kinase activity	9	0.8	4.30E-02	4.80E-01
adult locomotory behavior	9	0.8	5.50E-02	5.40E-01
mRNA transport	9	0.8	5.50E-02	5.40E-01
regulation of epithelial cell proliferation	9	0.8	6.00E-02	5.60E-01
negative regulation of actin filament depolymerization	8	0.7	3.10E-04	1.40E-02
negative regulation of actin filament polymerization	8	0.7	1.00E-03	3.60E-02
salivary gland morphogenesis	8	0.7	1.70E-03	5.30E-02
salivary gland development	8	0.7	3.90E-03	1.00E-01
cytoskeleton-dependent intracellular transport	8	0.7	5.60E-03	1.40E-01
pigment metabolic process	8	0.7	1.10E-02	2.00E-01
glycosaminoglycan metabolic process	8	0.7	1.60E-02	2.70E-01
exocrine system development	8	0.7	1.80E-02	3.00E-01
regulation of intracellular transport	8	0.7	2.10E-02	3.20E-01
cell recognition	8	0.7	3.70E-02	4.40E-01
mesenchymal cell development	8	0.7	3.70E-02	4.40E-01
activation of MAPK activity	8	0.7	4.90E-02	5.10E-01
regulation of heart contraction	8	0.7	4.90E-02	5.10E-01
Golgi vesicle transport	8	0.7	5.80E-02	5.50E-01
regulation of lymphocyte differentiation	8	0.7	9.30E-02	6.80E-01
purine ribonucleoside monophosphate biosynthetic process	7	0.6	1.00E-04	5.90E-03
purine nucleoside monophosphate biosynthetic process	7	0.6	1.00E-04	5.90E-03
purine nucleoside monophosphate metabolic process	7	0.6	2.70E-04	1.30E-02
purine ribonucleoside monophosphate metabolic process	7	0.6	2.70E-04	1.30E-02
actin filament bundle formation	7	0.6	2.70E-04	1.30E-02
branching involved in salivary gland morphogenesis	7	0.6	8.40E-04	3.10E-02
actin filament capping	7	0.6	1.20E-03	3.90E-02

oligosaccharide metabolic process	7	0.6	1.60E-03	5.10E-02
hydrogen peroxide metabolic process	7	0.6	1.60E-03	5.10E-02
lysosome organization	7	0.6	2.70E-03	7.50E-02
cytokinesis	7	0.6	8.00E-03	1.70E-01
positive regulation of cell-substrate adhesion	7	0.6	8.00E-03	1.70E-01
regulation of intracellular protein transport	7	0.6	1.40E-02	2.40E-01
pigment biosynthetic process	7	0.6	1.40E-02	2.40E-01
proteoglycan metabolic process	7	0.6	2.10E-02	3.20E-01
positive regulation of endocytosis	7	0.6	2.40E-02	3.50E-01
regulation of nucleocytoplasmic transport	7	0.6	2.80E-02	3.70E-01
mesoderm formation	7	0.6	3.10E-02	4.00E-01
cellular component assembly involved in morphogenesis	7	0.6	3.10E-02	4.00E-01
energy coupled proton transport, down electrochemical gradient	7	0.6	3.60E-02	4.30E-01
ATP synthesis coupled proton transport	7	0.6	3.60E-02	4.30E-01
mesoderm morphogenesis	7	0.6	4.00E-02	4.60E-01
muscle fiber development	7	0.6	4.00E-02	4.60E-01
dendrite development	7	0.6	4.50E-02	4.90E-01
cellular component disassembly	7	0.6	4.50E-02	4.90E-01
regulation of cell-substrate adhesion	7	0.6	5.00E-02	5.10E-01
formation of primary germ layer	7	0.6	5.00E-02	5.10E-01
sulfur compound biosynthetic process	7	0.6	5.50E-02	5.40E-01
ion transmembrane transport	7	0.6	6.10E-02	5.60E-01
glutamine family amino acid metabolic process	7	0.6	6.70E-02	5.80E-01
cellular amino acid biosynthetic process	7	0.6	7.30E-02	6.10E-01
regulation of microtubule-based process	7	0.6	8.00E-02	6.30E-01
nucleus organization	7	0.6	8.70E-02	6.60E-01
single fertilization	7	0.6	8.70E-02	6.60E-01
tissue remodeling	7	0.6	9.40E-02	6.80E-01
oxygen and reactive oxygen species metabolic process	7	0.6	9.40E-02	6.80E-01
protein import into nucleus, docking	6	0.5	2.30E-03	6.60E-02
myofibril assembly	6	0.5	7.20E-03	1.60E-01
glutamine metabolic process	6	0.5	7.20E-03	1.60E-01
steroid hormone receptor signaling pathway	6	0.5	1.10E-02	2.10E-01
collagen fibril organization	6	0.5	1.10E-02	2.10E-01
response to hydrogen peroxide	6	0.5	2.00E-02	3.10E-01
actomyosin structure organization	6	0.5	2.40E-02	3.40E-01
positive regulation of cytoskeleton organization	6	0.5	2.80E-02	3.70E-01
antigen processing and presentation of exogenous antigen	6	0.5	3.70E-02	4.40E-01
regulation of transforming growth factor beta receptor signaling pathway	6	0.5	4.80E-02	5.00E-01
positive regulation of T cell differentiation	6	0.5	6.10E-02	5.60E-01
regulation of protein catabolic process	6	0.5	6.10E-02	5.60E-01
intracellular receptor-mediated signaling pathway	6	0.5	6.10E-02	5.60E-01
ribonucleoprotein complex assembly	6	0.5	7.50E-02	6.20E-01
positive regulation of lymphocyte differentiation	6	0.5	7.50E-02	6.20E-01
antigen processing and presentation of peptide antigen	6	0.5	8.30E-02	6.50E-01
response to reactive oxygen species	6	0.5	9.20E-02	6.70E-01
triglyceride metabolic process	6	0.5	9.20E-02	6.70E-01
IMP biosynthetic process	4	0.4	1.20E-03	4.00E-02
IMP metabolic process	4	0.4	1.20E-03	4.00E-02
positive regulation of protein complex disassembly	4	0.4	5.20E-03	1.30E-01
hydrogen peroxide catabolic process	5	0.4	6.50E-03	1.50E-01
cellular response to hydrogen peroxide	5	0.4	6.50E-03	1.50E-01
myotube differentiation	5	0.4	6.50E-03	1.50E-01
cortical cytoskeleton organization	5	0.4	8.90E-03	1.80E-01
actin filament-based movement	5	0.4	1.50E-02	2.60E-01
nucleobase biosynthetic process	4	0.4	1.90E-02	3.00E-01
antigen processing and presentation of peptide antigen via MHC class I	5	0.4	2.40E-02	3.40E-01

cellular response to reactive oxygen species	5	0.4	2.90E-02	3.80E-01
purine base metabolic process	4	0.4	3.30E-02	4.20E-01
mannose metabolic process	4	0.4	3.30E-02	4.20E-01
nucleobase metabolic process	5	0.4	3.50E-02	4.30E-01
regulation of nitric oxide biosynthetic process	5	0.4	3.50E-02	4.30E-01
cell-cell recognition	5	0.4	4.20E-02	4.70E-01
chaperone mediated protein folding requiring cofactor	4	0.4	4.20E-02	4.70E-01
peptidyl-lysine modification	4	0.4	4.20E-02	4.70E-01
membrane protein ectodomain proteolysis	4	0.4	5.20E-02	5.30E-01
membrane protein proteolysis	4	0.4	5.20E-02	5.30E-01
cellular response to oxidative stress	5	0.4	5.70E-02	5.50E-01
cell-substrate junction assembly	4	0.4	6.30E-02	5.70E-01
'de novo' protein folding	4	0.4	6.30E-02	5.70E-01
chondroitin sulfate metabolic process	4	0.4	6.30E-02	5.70E-01
vascular endothelial growth factor receptor signaling pathway	4	0.4	6.30E-02	5.70E-01
'de novo' posttranslational protein folding	4	0.4	6.30E-02	5.70E-01
neural crest cell migration	5	0.4	6.50E-02	5.80E-01
deoxyribonucleotide metabolic process	5	0.4	6.50E-02	5.80E-01
purine nucleoside metabolic process	5	0.4	7.40E-02	6.20E-01
hippocampus development	5	0.4	7.40E-02	6.20E-01
alkaloid metabolic process	5	0.4	7.40E-02	6.20E-01
nicotinamide nucleotide metabolic process	5	0.4	7.40E-02	6.20E-01
purine ribonucleoside metabolic process	5	0.4	7.40E-02	6.20E-01
nicotinamide metabolic process	5	0.4	7.40E-02	6.20E-01
positive regulation of nitric oxide biosynthetic process	4	0.4	7.50E-02	6.20E-01
regulation of phagocytosis	5	0.4	8.40E-02	6.50E-01
regulation of morphogenesis of a branching structure	5	0.4	8.40E-02	6.50E-01
chondroitin sulfate proteoglycan metabolic process	4	0.4	8.80E-02	6.60E-01
lamellipodium assembly	4	0.4	8.80E-02	6.60E-01
2'-deoxyribonucleotide metabolic process	4	0.4	8.80E-02	6.60E-01
negative regulation of transforming growth factor beta receptor signaling path	4	0.4	8.80E-02	6.60E-01
myoblast proliferation	3	0.3	1.30E-02	2.30E-01
'de novo' IMP biosynthetic process	3	0.3	1.30E-02	2.30E-01
N-glycan processing	3	0.3	2.50E-02	3.50E-01
purine base biosynthetic process	3	0.3	4.00E-02	4.60E-01
induction of apoptosis by oxidative stress	3	0.3	4.00E-02	4.60E-01
ruffle organization	3	0.3	4.00E-02	4.60E-01
skeletal myofibril assembly	3	0.3	4.00E-02	4.60E-01
dichotomous subdivision of terminal units involved in salivary gland branching	3	0.3	4.00E-02	4.60E-01
cellular metabolic compound salvage	3	0.3	4.00E-02	4.60E-01
purine deoxyribonucleotide metabolic process	3	0.3	5.70E-02	5.40E-01
peptidyl-lysine modification to hypusine	3	0.3	5.70E-02	5.40E-01
hypusine metabolic process	3	0.3	5.70E-02	5.40E-01
cardiac myofibril assembly	3	0.3	5.70E-02	5.40E-01
glycolipid catabolic process	3	0.3	5.70E-02	5.40E-01
purine deoxyribonucleoside triphosphate metabolic process	3	0.3	5.70E-02	5.40E-01
dichotomous subdivision of an epithelial terminal unit	3	0.3	5.70E-02	5.40E-01
ribosomal small subunit biogenesis	3	0.3	5.70E-02	5.40E-01
purine ribonucleoside biosynthetic process	3	0.3	7.60E-02	6.20E-01
purine nucleoside biosynthetic process	3	0.3	7.60E-02	6.20E-01
L-serine metabolic process	3	0.3	7.60E-02	6.20E-01
regulation of centrosome cycle	3	0.3	7.60E-02	6.20E-01
positive regulation of mitotic cell cycle	3	0.3	7.60E-02	6.20E-01
nucleoside biosynthetic process	3	0.3	7.60E-02	6.20E-01
androgen receptor signaling pathway	3	0.3	7.60E-02	6.20E-01
ribonucleoside biosynthetic process	3	0.3	7.60E-02	6.20E-01
positive regulation of actin filament polymerization	3	0.3	9.70E-02	6.80E-01

pentose-phosphate shunt	3	0.3	9.70E-02	6.80E-01
cerebral cortex radially oriented cell migration	3	0.3	9.70E-02	6.80E-01
regulation of lipid kinase activity	3	0.3	9.70E-02	6.80E-01

Day8_CC				
Term	Count	%	P-Value	Benjamini
non-membrane-bounded organelle	247	19.4	4.70E-18	5.60E-16
intracellular non-membrane-bounded organelle	247	19.4	4.70E-18	5.60E-16
extracellular region	159	12.5	3.60E-03	2.10E-02
cytoskeleton	144	11.3	4.90E-10	1.30E-08
membrane-enclosed lumen	134	10.5	2.30E-06	2.90E-05
intracellular organelle lumen	125	9.8	2.70E-05	2.70E-04
organelle lumen	125	9.8	3.00E-05	2.80E-04
ribonucleoprotein complex	118	9.2	1.70E-32	8.10E-30
cytosol	109	8.5	1.50E-20	2.40E-18
cytoskeletal part	108	8.5	1.10E-09	2.80E-08
vesicle	100	7.8	6.10E-18	5.80E-16
cytoplasmic vesicle	98	7.7	1.30E-17	1.00E-15
extracellular region part	96	7.5	2.80E-06	3.50E-05
Golgi apparatus	89	7	8.40E-07	1.20E-05
membrane-bounded vesicle	86	6.7	3.90E-17	2.70E-15
cytoplasmic membrane-bounded vesicle	85	6.7	5.10E-17	3.10E-15
endoplasmic reticulum	86	6.7	4.70E-03	2.60E-02
cell projection	79	6.2	5.10E-07	7.60E-06
endomembrane system	77	6	1.10E-07	1.90E-06
cell fraction	67	5.3	1.60E-03	1.10E-02
insoluble fraction	55	4.3	1.90E-02	8.20E-02
proteinaceous extracellular matrix	53	4.2	1.60E-08	3.40E-07
extracellular matrix	54	4.2	2.40E-08	4.70E-07
extrinsic to membrane	54	4.2	3.50E-03	2.10E-02
extracellular space	53	4.2	2.30E-02	9.70E-02
vacuole	50	3.9	3.80E-13	1.60E-11
actin cytoskeleton	50	3.9	4.60E-13	1.80E-11
microtubule cytoskeleton	50	3.9	8.60E-03	4.20E-02
membrane fraction	50	3.9	6.30E-02	2.20E-01
ribosome	48	3.8	5.50E-13	2.00E-11
internal side of plasma membrane	49	3.8	2.40E-07	3.80E-06
lysosome	45	3.5	2.10E-12	7.30E-11
lytic vacuole	45	3.5	2.60E-12	8.30E-11
pigment granule	42	3.3	1.10E-23	2.70E-21
melanosome	42	3.3	1.10E-23	2.70E-21
spliceosome	39	3.1	4.80E-14	2.50E-12
Golgi apparatus part	40	3.1	1.60E-06	2.20E-05
endoplasmic reticulum part	39	3.1	6.40E-06	7.10E-05
microtubule	34	2.7	8.10E-04	6.10E-03
cell surface	35	2.7	1.90E-02	8.20E-02
nucleolus	34	2.7	3.60E-02	1.40E-01
cell leading edge	30	2.4	4.00E-09	9.40E-08
cell cortex	30	2.4	6.80E-08	1.30E-06
neuron projection	31	2.4	7.70E-03	3.90E-02
endoplasmic reticulum lumen	29	2.3	1.30E-13	6.30E-12
nuclear envelope	29	2.3	1.40E-05	1.50E-04
extracellular matrix part	26	2	1.60E-08	3.40E-07
membrane coat	24	1.9	1.30E-11	3.80E-10
coated membrane	24	1.9	1.30E-11	3.80E-10
basement membrane	22	1.7	7.80E-08	1.40E-06

Golgi membrane	22	1.7	1.80E-03	1.20E-02
proteasome complex	21	1.6	5.00E-09	1.10E-07
cytosolic part	20	1.6	1.40E-07	2.40E-06
cell cortex part	21	1.6	3.80E-07	5.80E-06
contractile fiber part	20	1.6	2.30E-05	2.30E-04
contractile fiber	21	1.6	3.00E-05	2.80E-04
coated vesicle	20	1.6	3.00E-03	1.80E-02
basolateral plasma membrane	21	1.6	5.70E-03	3.00E-02
myofibril	19	1.5	1.70E-04	1.40E-03
soluble fraction	19	1.5	5.10E-04	3.90E-03
chromatin	19	1.5	8.50E-02	2.70E-01
cell soma	18	1.4	8.20E-03	4.10E-02
nuclear body	18	1.4	4.60E-02	1.70E-01
ruffle	17	1.3	2.10E-07	3.50E-06
ribosomal subunit	17	1.3	3.00E-05	2.80E-04
sarcomere	16	1.3	1.00E-03	7.50E-03
vesicle membrane	17	1.3	2.90E-03	1.80E-02
axon	17	1.3	7.70E-03	3.90E-02
apical part of cell	17	1.3	5.90E-02	2.10E-01
intermediate filament	17	1.3	6.20E-02	2.20E-01
intermediate filament cytoskeleton	17	1.3	7.30E-02	2.40E-01
cytoplasmic vesicle membrane	15	1.2	6.50E-03	3.40E-02
cytoplasmic vesicle part	15	1.2	1.40E-02	6.30E-02
adherens junction	15	1.2	3.30E-02	1.30E-01
anchoring junction	15	1.2	9.10E-02	2.90E-01
clathrin coat	13	1	4.60E-06	5.30E-05
focal adhesion	13	1	1.10E-03	8.00E-03
nuclear pore	13	1	1.60E-03	1.10E-02
cell-substrate adherens junction	13	1	2.10E-03	1.40E-02
lamellipodium	13	1	2.80E-03	1.70E-02
cell-substrate junction	13	1	4.20E-03	2.30E-02
pore complex	13	1	9.50E-03	4.60E-02
eukaryotic translation initiation factor 3 complex	11	0.9	4.10E-10	1.10E-08
stress fiber	11	0.9	3.20E-06	3.90E-05
vesicle coat	12	0.9	3.40E-06	4.10E-05
actin filament bundle	11	0.9	5.10E-06	5.80E-05
coated pit	12	0.9	1.50E-05	1.60E-04
actomyosin	11	0.9	1.80E-05	1.80E-04
Golgi-associated vesicle	12	0.9	5.40E-05	4.70E-04
cortical cytoskeleton	12	0.9	3.10E-04	2.50E-03
I band	12	0.9	2.00E-03	1.30E-02
coated vesicle membrane	12	0.9	2.00E-03	1.30E-02
Z disc	11	0.9	2.20E-03	1.40E-02
extrinsic to plasma membrane	12	0.9	2.80E-03	1.70E-02
nuclear periphery	11	0.9	3.70E-03	2.10E-02
myosin complex	11	0.9	1.70E-02	7.70E-02
proton-transporting two-sector ATPase complex, catalytic domain	10	0.8	1.30E-06	1.80E-05
transport vesicle	10	0.8	2.30E-04	1.90E-03
small ribosomal subunit	10	0.8	2.30E-04	1.90E-03
proton-transporting two-sector ATPase complex	10	0.8	4.10E-03	2.30E-02
keratin filament	10	0.8	5.60E-02	2.00E-01
cytosolic ribosome	9	0.7	2.60E-05	2.60E-04
AP-type membrane coat adaptor complex	9	0.7	4.00E-04	3.20E-03
clathrin adaptor complex	9	0.7	4.00E-04	3.20E-03
actin filament	9	0.7	1.50E-03	1.10E-02
organelle envelope lumen	9	0.7	1.00E-02	4.80E-02
site of polarized growth	9	0.7	4.00E-02	1.50E-01

growth cone	9	0.7	4.00E-02	1.50E-01
septin complex	8	0.6	3.40E-05	3.00E-04
septin cytoskeleton	8	0.6	3.40E-05	3.00E-04
Golgi-associated vesicle membrane	8	0.6	7.00E-04	5.40E-03
nuclear membrane	8	0.6	2.40E-02	1.00E-01
large ribosomal subunit	8	0.6	2.80E-02	1.10E-01
nuclear matrix	8	0.6	4.00E-02	1.50E-01
sarcolemma	8	0.6	6.70E-02	2.30E-01
chaperonin-containing T-complex	7	0.5	1.40E-06	1.80E-05
proton-transporting V-type ATPase, V1 domain	6	0.5	1.20E-04	1.10E-03
transport vesicle membrane	7	0.5	8.40E-04	6.30E-03
filamentous actin	6	0.5	4.20E-03	2.30E-02
neuromuscular junction	7	0.5	5.30E-03	2.80E-02
proton-transporting V-type ATPase complex	6	0.5	9.90E-03	4.80E-02
trans-Golgi network transport vesicle	6	0.5	9.90E-03	4.80E-02
mitochondrial intermembrane space	7	0.5	2.50E-02	1.00E-01
heterotrimeric G-protein complex	7	0.5	4.90E-02	1.80E-01
proteasome accessory complex	5	0.4	1.60E-04	1.40E-03
cytosolic large ribosomal subunit	5	0.4	1.00E-03	7.40E-03
COPI vesicle coat	5	0.4	5.10E-03	2.70E-02
ciliary rootlet	5	0.4	5.10E-03	2.70E-02
COPI coated vesicle membrane	5	0.4	5.10E-03	2.70E-02
COPI-coated vesicle	5	0.4	1.10E-02	5.00E-02
basal lamina	5	0.4	1.40E-02	6.50E-02
collagen	5	0.4	5.40E-02	1.90E-01
filopodium	5	0.4	8.40E-02	2.70E-01
trailing edge	4	0.3	4.10E-03	2.30E-02
uropod	4	0.3	4.10E-03	2.30E-02
clathrin coat of coated pit	4	0.3	7.70E-03	3.90E-02
Arp2/3 protein complex	4	0.3	7.70E-03	3.90E-02
ER to Golgi transport vesicle membrane	4	0.3	1.30E-02	5.90E-02
COPII vesicle coat	4	0.3	1.30E-02	5.90E-02
ER to Golgi transport vesicle	4	0.3	1.90E-02	8.20E-02
nuclear lamina	4	0.3	1.90E-02	8.20E-02
cyclin-dependent protein kinase holoenzyme complex	4	0.3	2.70E-02	1.10E-01
prefoldin complex	4	0.3	3.60E-02	1.40E-01
kinesin complex	4	0.3	5.90E-02	2.10E-01
small nuclear ribonucleoprotein complex	4	0.3	5.90E-02	2.10E-01
immunological synapse	4	0.3	7.30E-02	2.40E-01
polysome	4	0.3	8.80E-02	2.80E-01
aminoacyl-tRNA synthetase multienzyme complex	3	0.2	1.70E-02	7.60E-02
proteasome activator complex	3	0.2	1.70E-02	7.60E-02
lamin filament	3	0.2	3.20E-02	1.30E-01
vacuolar lumen	3	0.2	5.10E-02	1.90E-01
cell division site part	3	0.2	9.60E-02	3.00E-01
cell division site	3	0.2	9.60E-02	3.00E-01
clathrin coat of trans-Golgi network vesicle	3	0.2	9.60E-02	3.00E-01
laminin complex	3	0.2	9.60E-02	3.00E-01
striated muscle thin filament	3	0.2	9.60E-02	3.00E-01
Day8_MF				
Term	Count	%	P-Value	Benjamini
nucleotide binding	286	22.4	1.10E-20	5.40E-18
purine nucleotide binding	232	18.2	2.00E-13	2.70E-11
purine ribonucleotide binding	226	17.7	9.60E-14	1.80E-11
ribonucleotide binding	226	17.7	9.60E-14	1.80E-11
adenyl nucleotide binding	181	14.2	1.10E-08	8.20E-07
purine nucleoside binding	181	14.2	2.00E-08	1.30E-06

nucleoside binding	181	14.2	3.30E-08	2.00E-06
adenyl ribonucleotide binding	175	13.7	6.10E-09	4.90E-07
ATP binding	172	13.5	1.30E-08	9.00E-07
RNA binding	115	9	1.40E-15	4.60E-13
structural molecule activity	99	7.8	3.00E-21	2.90E-18
calcium ion binding	90	7.1	2.20E-03	5.00E-02
peptidase activity	70	5.5	2.90E-03	5.90E-02
peptidase activity, acting on L-amino acid peptides	67	5.3	3.70E-03	6.80E-02
cytoskeletal protein binding	63	4.9	6.00E-07	3.00E-05
GTP binding	56	4.4	8.10E-07	3.90E-05
guanyl nucleotide binding	56	4.4	1.80E-06	8.10E-05
guanyl ribonucleotide binding	56	4.4	1.80E-06	8.10E-05
actin binding	51	4	8.10E-08	4.60E-06
endopeptidase activity	50	3.9	3.70E-03	6.90E-02
structural constituent of ribosome	45	3.5	8.40E-15	2.00E-12
carbohydrate binding	42	3.3	1.10E-03	2.80E-02
ATPase activity	38	3	1.40E-03	3.20E-02
GTPase activity	37	2.9	7.40E-12	8.90E-10
ATPase activity, coupled	35	2.7	1.50E-04	5.00E-03
translation factor activity, nucleic acid binding	30	2.4	2.10E-10	2.30E-08
unfolded protein binding	29	2.3	9.90E-14	1.60E-11
protein domain specific binding	28	2.2	2.30E-03	5.00E-02
ligase activity, forming carbon-nitrogen bonds	28	2.2	2.50E-03	5.30E-02
polysaccharide binding	27	2.1	6.00E-06	2.60E-04
pattern binding	27	2.1	6.00E-06	2.60E-04
metallopeptidase activity	27	2.1	3.20E-03	6.20E-02
cofactor binding	25	2	8.20E-02	6.10E-01
glycosaminoglycan binding	24	1.9	2.30E-05	9.20E-04
translation initiation factor activity	23	1.8	6.90E-10	6.60E-08
aminoacyl-tRNA ligase activity	20	1.6	1.20E-09	1.00E-07
ligase activity, forming aminoacyl-tRNA and related compounds	20	1.6	1.20E-09	1.00E-07
ligase activity, forming carbon-oxygen bonds	20	1.6	1.20E-09	1.00E-07
motor activity	21	1.6	5.70E-03	9.90E-02
helicase activity	20	1.6	5.80E-03	9.90E-02
manganese ion binding	21	1.6	1.20E-02	1.80E-01
coenzyme binding	21	1.6	2.70E-02	3.20E-01
protein transporter activity	17	1.3	3.90E-05	1.50E-03
growth factor binding	16	1.3	4.10E-04	1.30E-02
heparin binding	17	1.3	6.60E-04	1.90E-02
metalloendopeptidase activity	16	1.3	3.40E-02	3.70E-01
purine NTP-dependent helicase activity	15	1.2	7.00E-03	1.10E-01
ATP-dependent helicase activity	15	1.2	7.00E-03	1.10E-01
actin filament binding	14	1.1	7.50E-05	2.70E-03
ATPase activity, coupled to transmembrane movement of ions	14	1.1	2.60E-03	5.30E-02
exopeptidase activity	14	1.1	2.90E-03	5.90E-02
monovalent inorganic cation transmembrane transporter activity	14	1.1	1.80E-02	2.50E-01
ATPase activity, coupled to movement of substances	14	1.1	4.10E-02	4.10E-01
hydrolase activity, acting on acid anhydrides, catalyzing transmembrane mover	14	1.1	4.10E-02	4.10E-01
ATPase activity, coupled to transmembrane movement of substances	14	1.1	4.10E-02	4.10E-01
P-P-bond-hydrolysis-driven transmembrane transporter activity	14	1.1	7.70E-02	6.00E-01
primary active transmembrane transporter activity	14	1.1	8.20E-02	6.10E-01
transferase activity, transferring alkyl or aryl (other than methyl) groups	13	1	6.90E-04	1.90E-02
extracellular matrix binding	11	0.9	9.60E-06	4.00E-04
intramolecular oxidoreductase activity	11	0.9	5.40E-04	1.60E-02
cysteine-type endopeptidase activity	11	0.9	2.50E-02	3.10E-01
proton-transporting ATPase activity, rotational mechanism	10	0.8	3.70E-07	2.00E-05
cation-transporting ATPase activity	10	0.8	7.30E-05	2.70E-03

structural constituent of cytoskeleton	10	0.8	2.70E-04	8.80E-03
ribonucleoprotein binding	10	0.8	9.80E-04	2.50E-02
NAD or NADH binding	10	0.8	6.50E-03	1.10E-01
rRNA binding	9	0.7	3.40E-04	1.10E-02
glutathione transferase activity	9	0.7	6.30E-04	1.80E-02
antioxidant activity	8	0.6	3.50E-02	3.80E-01
hydrogen ion transporting ATP synthase activity, rotational mechanism	6	0.5	9.30E-04	2.50E-02
ribosome binding	7	0.5	1.40E-03	3.20E-02
tRNA binding	6	0.5	1.70E-02	2.50E-01
aminopeptidase activity	7	0.5	2.00E-02	2.70E-01
extracellular matrix structural constituent	7	0.5	2.70E-02	3.30E-01
translation elongation factor activity	7	0.5	3.20E-02	3.60E-01
intramolecular transferase activity	6	0.5	3.60E-02	3.90E-01
integrin binding	6	0.5	4.30E-02	4.20E-01
peptidyl-prolyl cis-trans isomerase activity	7	0.5	5.40E-02	4.80E-01
peroxidase activity	6	0.5	6.50E-02	5.50E-01
oxidoreductase activity, acting on peroxide as acceptor	6	0.5	6.50E-02	5.50E-01
cis-trans isomerase activity	7	0.5	6.70E-02	5.60E-01
hormone receptor binding	7	0.5	8.30E-02	6.10E-01
cyclin-dependent protein kinase activity	6	0.5	8.30E-02	6.00E-01
peroxiredoxin activity	5	0.4	1.10E-03	2.70E-02
intramolecular oxidoreductase activity, interconverting keto- and enol-groups	5	0.4	3.50E-03	6.70E-02
platelet-derived growth factor binding	5	0.4	3.50E-03	6.70E-02
intramolecular transferase activity, phosphotransferases	5	0.4	5.50E-03	9.70E-02
mannosidase activity	5	0.4	2.60E-02	3.20E-01
oxidoreductase activity, acting on the CH-NH2 group of donors, oxygen as acc	5	0.4	4.80E-02	4.60E-01
L-ascorbic acid binding	5	0.4	6.70E-02	5.60E-01
oxidoreductase activity, acting on the CH-NH2 group of donors	5	0.4	7.80E-02	6.00E-01
insulin-like growth factor binding	5	0.4	9.00E-02	6.30E-01
actin-dependent ATPase activity	4	0.3	8.10E-03	1.30E-01
intramolecular oxidoreductase activity, transposing S-S bonds	4	0.3	2.00E-02	2.70E-01
p53 binding	4	0.3	2.00E-02	2.70E-01
protein disulfide isomerase activity	4	0.3	2.00E-02	2.70E-01
alpha-mannosidase activity	4	0.3	2.00E-02	2.70E-01
intramolecular oxidoreductase activity, interconverting aldoses and ketoses	4	0.3	2.90E-02	3.40E-01
carbon-nitrogen ligase activity, with glutamine as amido-N-donor	4	0.3	2.90E-02	3.40E-01
sodium:potassium-exchanging ATPase activity	4	0.3	3.80E-02	4.00E-01
protein kinase C binding	4	0.3	5.00E-02	4.70E-01
sulfuric ester hydrolase activity	4	0.3	7.70E-02	6.00E-01
procollagen-lysine 5-dioxygenase activity	3	0.2	1.80E-02	2.50E-01
phosphoglucomutase activity	3	0.2	1.80E-02	2.50E-01
ADP binding	3	0.2	3.30E-02	3.70E-01
peptidyl-lysine 5-dioxygenase activity	3	0.2	3.30E-02	3.70E-01
proteasome regulator activity	3	0.2	3.30E-02	3.70E-01
proteasome activator activity	3	0.2	3.30E-02	3.70E-01
GDP-dissociation inhibitor activity	3	0.2	5.30E-02	4.80E-01
serine-type exopeptidase activity	3	0.2	5.30E-02	4.80E-01
serine-type carboxypeptidase activity	3	0.2	5.30E-02	4.80E-01
microfilament motor activity	3	0.2	1.00E-01	6.70E-01
Day8_BP				
Term	Count	%	P-Value	Benjamini
protein localization	110	8.6	3.70E-11	1.60E-08
translation	98	7.7	4.20E-34	1.20E-30
proteolysis	98	7.7	2.00E-02	2.60E-01
establishment of protein localization	96	7.5	7.10E-10	2.10E-07
protein transport	95	7.4	1.00E-09	2.50E-07
intracellular transport	82	6.4	1.90E-14	2.80E-11

vesicle-mediated transport	75	5.9	1.10E-09	2.50E-07
RNA processing	68	5.3	2.80E-08	4.20E-06
cell adhesion	65	5.1	8.00E-04	3.00E-02
biological adhesion	65	5.1	8.30E-04	3.00E-02
homeostatic process	65	5.1	2.20E-03	6.00E-02
mRNA metabolic process	61	4.8	4.70E-12	2.80E-09
macromolecule catabolic process	61	4.8	7.90E-02	6.10E-01
macromolecular complex subunit organization	60	4.7	3.50E-08	4.90E-06
macromolecular complex assembly	58	4.5	1.10E-08	1.60E-06
regulation of apoptosis	57	4.5	1.90E-02	2.60E-01
regulation of programmed cell death	57	4.5	2.30E-02	2.90E-01
regulation of cell death	57	4.5	2.50E-02	3.00E-01
cell cycle	57	4.5	9.20E-02	6.50E-01
mRNA processing	55	4.3	1.30E-11	6.30E-09
cellular protein localization	55	4.3	2.20E-09	4.30E-07
cellular macromolecule localization	55	4.3	2.80E-09	5.20E-07
intracellular protein transport	53	4.2	9.40E-10	2.50E-07
cytoskeleton organization	50	3.9	3.70E-06	2.80E-04
RNA splicing	49	3.8	6.70E-13	5.00E-10
cellular macromolecular complex subunit organization	49	3.8	1.10E-09	2.30E-07
nitrogen compound biosynthetic process	49	3.8	9.40E-07	8.70E-05
cellular macromolecular complex assembly	47	3.7	1.60E-10	6.00E-08
cellular homeostasis	44	3.4	9.10E-04	3.20E-02
cell motion	42	3.3	9.20E-03	1.70E-01
protein folding	39	3.1	9.20E-14	9.10E-11
actin filament-based process	39	3.1	3.70E-09	6.40E-07
protein complex assembly	40	3.1	1.40E-06	1.10E-04
protein complex biogenesis	40	3.1	1.40E-06	1.10E-04
membrane organization	40	3.1	1.00E-04	5.20E-03
cellular component morphogenesis	39	3.1	1.90E-02	2.60E-01
cell proliferation	37	2.9	1.30E-04	6.50E-03
cell projection organization	37	2.9	1.20E-02	2.00E-01
nucleobase, nucleoside and nucleotide biosynthetic process	36	2.8	2.10E-07	2.50E-05
nucleobase, nucleoside, nucleotide and nucleic acid biosynthetic process	36	2.8	2.10E-07	2.50E-05
positive regulation of molecular function	36	2.8	1.10E-02	1.90E-01
actin cytoskeleton organization	34	2.7	2.60E-07	3.00E-05
cell motility	34	2.7	1.10E-02	1.80E-01
localization of cell	34	2.7	1.10E-02	1.80E-01
nucleotide biosynthetic process	33	2.6	2.70E-06	2.20E-04
small GTPase mediated signal transduction	33	2.6	4.60E-03	1.00E-01
cell division	33	2.6	1.50E-02	2.30E-01
cell morphogenesis	33	2.6	5.00E-02	4.80E-01
endocytosis	32	2.5	3.80E-05	2.10E-03
membrane invagination	32	2.5	3.80E-05	2.10E-03
generation of precursor metabolites and energy	32	2.5	9.60E-03	1.70E-01
positive regulation of catalytic activity	32	2.5	9.60E-03	1.70E-01
regulation of phosphate metabolic process	32	2.5	5.60E-02	5.10E-01
regulation of phosphorus metabolic process	32	2.5	5.60E-02	5.10E-01
regulation of organelle organization	30	2.4	4.90E-06	3.40E-04
hexose metabolic process	31	2.4	1.20E-05	7.30E-04
monosaccharide metabolic process	31	2.4	1.20E-04	6.10E-03
ncRNA metabolic process	30	2.4	7.40E-04	2.90E-02
in utero embryonic development	30	2.4	3.50E-02	3.80E-01
regulation of phosphorylation	31	2.4	5.60E-02	5.10E-01
regulation of cellular protein metabolic process	30	2.4	6.00E-02	5.30E-01
cellular protein complex assembly	29	2.3	6.20E-09	1.00E-06
purine nucleotide biosynthetic process	29	2.3	1.30E-06	1.10E-04

purine nucleotide metabolic process	29	2.3	2.90E-05	1.70E-03
tissue morphogenesis	29	2.3	1.50E-02	2.30E-01
negative regulation of apoptosis	29	2.3	1.60E-02	2.30E-01
negative regulation of programmed cell death	29	2.3	2.00E-02	2.70E-01
negative regulation of cell death	29	2.3	2.10E-02	2.70E-01
M phase	29	2.3	9.70E-02	6.70E-01
ribonucleotide metabolic process	28	2.2	6.60E-07	6.30E-05
regulation of cellular component size	28	2.2	8.70E-05	4.50E-03
muscle organ development	28	2.2	3.90E-04	1.70E-02
cell migration	28	2.2	2.80E-02	3.30E-01
ribonucleotide biosynthetic process	27	2.1	2.00E-07	2.40E-05
cofactor metabolic process	27	2.1	1.50E-03	4.80E-02
regulation of cytoskeleton organization	25	2	2.90E-07	3.20E-05
purine ribonucleotide biosynthetic process	25	2	1.30E-06	1.20E-04
purine ribonucleotide metabolic process	25	2	9.50E-06	6.10E-04
regulation of kinase activity	26	2	6.20E-03	1.30E-01
regulation of transferase activity	26	2	9.60E-03	1.70E-01
microtubule-based process	26	2	1.90E-02	2.50E-01
regulation of cell cycle	26	2	2.20E-02	2.80E-01
blood vessel development	26	2	8.30E-02	6.20E-01
glycoprotein metabolic process	24	1.9	7.80E-04	3.00E-02
extracellular structure organization	24	1.9	9.40E-04	3.30E-02
regulation of protein kinase activity	24	1.9	1.50E-02	2.30E-01
positive regulation of developmental process	24	1.9	6.10E-02	5.40E-01
neuron projection development	24	1.9	7.20E-02	5.80E-01
posttranscriptional regulation of gene expression	23	1.8	2.00E-03	5.60E-02
gland development	23	1.8	4.70E-02	4.70E-01
sulfur metabolic process	22	1.7	6.40E-06	4.30E-04
tRNA metabolic process	22	1.7	1.20E-04	5.90E-03
positive regulation of cellular component organization	22	1.7	3.60E-04	1.60E-02
muscle tissue development	22	1.7	1.50E-03	4.80E-02
blood vessel morphogenesis	22	1.7	8.00E-02	6.10E-01
tRNA aminoacylation	20	1.6	3.00E-10	9.90E-08
amino acid activation	20	1.6	3.00E-10	9.90E-08
tRNA aminoacylation for protein translation	20	1.6	3.00E-10	9.90E-08
regulation of cellular component biogenesis	20	1.6	3.50E-05	2.00E-03
striated muscle tissue development	21	1.6	1.50E-03	4.70E-02
protein targeting	21	1.6	2.70E-03	7.20E-02
positive regulation of kinase activity	20	1.6	4.70E-03	1.00E-01
glucose metabolic process	21	1.6	4.90E-03	1.10E-01
positive regulation of transferase activity	20	1.6	7.00E-03	1.40E-01
coenzyme metabolic process	20	1.6	1.30E-02	2.10E-01
positive regulation of cell differentiation	20	1.6	7.70E-02	6.00E-01
regulation of actin cytoskeleton organization	19	1.5	3.00E-07	3.10E-05
regulation of actin filament-based process	19	1.5	3.90E-07	4.00E-05
cell redox homeostasis	19	1.5	5.10E-07	5.10E-05
carbohydrate catabolic process	19	1.5	3.10E-05	1.80E-03
muscle cell differentiation	19	1.5	3.30E-03	8.30E-02
ribonucleoprotein complex biogenesis	19	1.5	1.70E-02	2.40E-01
regulation of actin polymerization or depolymerization	18	1.4	8.20E-08	1.10E-05
regulation of actin filament length	18	1.4	1.10E-07	1.50E-05
regulation of protein complex assembly	18	1.4	5.20E-06	3.50E-04
nucleocytoplasmic transport	18	1.4	9.00E-04	3.30E-02
nuclear transport	18	1.4	1.10E-03	3.90E-02
extracellular matrix organization	18	1.4	1.60E-03	4.90E-02
purine nucleoside triphosphate metabolic process	18	1.4	2.80E-03	7.30E-02
regulation of cell motion	18	1.4	3.00E-03	7.70E-02

nucleoside triphosphate metabolic process	18	1.4	6.40E-03	1.30E-01
positive regulation of protein kinase activity	18	1.4	1.30E-02	2.10E-01
actin filament organization	17	1.3	2.80E-06	2.20E-04
regulation of protein polymerization	17	1.3	3.60E-06	2.70E-04
regulation of vesicle-mediated transport	17	1.3	4.80E-04	2.00E-02
negative regulation of cellular component organization	17	1.3	1.70E-03	5.10E-02
purine nucleoside triphosphate biosynthetic process	17	1.3	1.90E-03	5.60E-02
nucleoside triphosphate biosynthetic process	17	1.3	2.20E-03	6.10E-02
striated muscle cell differentiation	16	1.3	2.90E-03	7.60E-02
polysaccharide metabolic process	16	1.3	4.10E-03	9.60E-02
microtubule-based movement	17	1.3	4.10E-03	9.50E-02
ribonucleoside triphosphate biosynthetic process	16	1.3	4.50E-03	1.00E-01
purine ribonucleoside triphosphate biosynthetic process	16	1.3	4.50E-03	1.00E-01
regulation of cell adhesion	16	1.3	5.00E-03	1.10E-01
regulation of translation	16	1.3	8.90E-03	1.70E-01
purine ribonucleoside triphosphate metabolic process	16	1.3	9.70E-03	1.70E-01
ribonucleoside triphosphate metabolic process	16	1.3	1.10E-02	1.80E-01
glycoprotein biosynthetic process	17	1.3	1.60E-02	2.40E-01
chromatin assembly or disassembly	16	1.3	1.90E-02	2.60E-01
angiogenesis	17	1.3	4.70E-02	4.70E-01
morphogenesis of a branching structure	16	1.3	5.40E-02	5.00E-01
cellular amino acid derivative metabolic process	17	1.3	7.30E-02	5.80E-01
regulation of actin filament polymerization	15	1.2	3.80E-06	2.80E-04
cellular carbohydrate catabolic process	15	1.2	1.30E-04	6.40E-03
regulation of cell morphogenesis	15	1.2	1.50E-02	2.30E-01
monosaccharide catabolic process	14	1.1	1.70E-04	7.60E-03
alcohol catabolic process	14	1.1	1.10E-03	3.90E-02
RNA transport	14	1.1	1.30E-03	4.30E-02
nucleic acid transport	14	1.1	1.30E-03	4.30E-02
establishment of RNA localization	14	1.1	1.30E-03	4.30E-02
RNA localization	14	1.1	1.50E-03	4.80E-02
cell projection assembly	14	1.1	2.30E-03	6.10E-02
nucleobase, nucleoside, nucleotide and nucleic acid transport	14	1.1	3.80E-03	9.10E-02
ATP biosynthetic process	14	1.1	9.10E-03	1.70E-01
response to oxidative stress	14	1.1	1.50E-02	2.30E-01
anti-apoptosis	14	1.1	1.60E-02	2.40E-01
ATP metabolic process	14	1.1	1.90E-02	2.60E-01
response to inorganic substance	14	1.1	2.30E-02	2.80E-01
ribosome biogenesis	14	1.1	8.60E-02	6.30E-01
translational initiation	13	1	1.60E-05	9.80E-04
regulation of protein complex disassembly	13	1	6.30E-05	3.30E-03
hexose catabolic process	13	1	4.40E-04	1.90E-02
glucose catabolic process	13	1	4.40E-04	1.90E-02
nuclear import	13	1	1.30E-03	4.20E-02
protein import	13	1	2.20E-02	2.80E-01
glycosylation	13	1	4.40E-02	4.50E-01
biopolymer glycosylation	13	1	4.40E-02	4.50E-01
protein amino acid glycosylation	13	1	4.40E-02	4.50E-01
regulation of mitotic cell cycle	13	1	6.20E-02	5.40E-01
protein localization in organelle	13	1	6.60E-02	5.60E-01
protein polymerization	12	0.9	1.50E-04	6.90E-03
pigment metabolic process	11	0.9	3.40E-04	1.50E-02
nucleoside monophosphate biosynthetic process	12	0.9	4.50E-04	1.90E-02
aminoglycan metabolic process	12	0.9	1.60E-03	4.90E-02
regulation of endocytosis	12	0.9	1.60E-03	4.90E-02
negative regulation of cytoskeleton organization	11	0.9	3.00E-03	7.60E-02
nucleoside monophosphate metabolic process	12	0.9	4.60E-03	1.00E-01

positive regulation of organelle organization	11	0.9	5.40E-03	1.10E-01
Golgi vesicle transport	11	0.9	5.40E-03	1.10E-01
mRNA transport	12	0.9	6.80E-03	1.40E-01
striated muscle cell development	11	0.9	7.20E-03	1.40E-01
protein import into nucleus	11	0.9	9.30E-03	1.70E-01
protein localization in nucleus	11	0.9	1.70E-02	2.40E-01
muscle cell development	11	0.9	1.70E-02	2.40E-01
negative regulation of organelle organization	11	0.9	1.90E-02	2.60E-01
skeletal muscle tissue development	12	0.9	2.00E-02	2.70E-01
regulation of cell cycle process	12	0.9	2.20E-02	2.80E-01
skeletal muscle organ development	12	0.9	2.50E-02	3.00E-01
placenta development	12	0.9	6.70E-02	5.60E-01
regulation of protein localization	12	0.9	8.20E-02	6.20E-01
regulation of cell migration	12	0.9	9.20E-02	6.50E-01
adult behavior	12	0.9	9.80E-02	6.70E-01
ribonucleoside monophosphate biosynthetic process	10	0.8	1.20E-06	1.10E-04
ribonucleoside monophosphate metabolic process	10	0.8	4.00E-06	2.80E-04
regulation of actin filament depolymerization	10	0.8	4.00E-05	2.20E-03
vacuole organization	10	0.8	7.90E-04	3.00E-02
neuromuscular process controlling balance	10	0.8	7.90E-04	3.00E-02
ATP synthesis coupled proton transport	10	0.8	1.50E-03	4.80E-02
energy coupled proton transport, down electrochemical gradient	10	0.8	1.50E-03	4.80E-02
ion transmembrane transport	10	0.8	3.90E-03	9.30E-02
glutamine family amino acid metabolic process	10	0.8	4.60E-03	1.00E-01
glycolysis	10	0.8	5.40E-03	1.10E-01
proton transport	10	0.8	1.60E-02	2.40E-01
hydrogen transport	10	0.8	1.80E-02	2.50E-01
oxidative phosphorylation	10	0.8	2.50E-02	3.00E-01
nucleoside metabolic process	10	0.8	2.50E-02	3.00E-01
positive regulation of MAP kinase activity	10	0.8	3.40E-02	3.80E-01
muscle contraction	10	0.8	3.80E-02	4.00E-01
neuromuscular process	10	0.8	3.80E-02	4.00E-01
regulation of epithelial cell proliferation	10	0.8	4.90E-02	4.80E-01
muscle system process	10	0.8	6.80E-02	5.60E-01
negative regulation of protein polymerization	9	0.7	6.80E-04	2.70E-02
negative regulation of protein complex assembly	9	0.7	6.80E-04	2.70E-02
pigment biosynthetic process	9	0.7	1.50E-03	4.70E-02
cytoskeleton-dependent intracellular transport	9	0.7	2.80E-03	7.40E-02
negative regulation of protein complex disassembly	9	0.7	4.20E-03	9.60E-02
RNA splicing, via transesterification reactions	9	0.7	6.00E-03	1.20E-01
RNA splicing, via transesterification reactions with bulged adenosine as nucleo	9	0.7	6.00E-03	1.20E-01
nuclear mRNA splicing, via spliceosome	9	0.7	6.00E-03	1.20E-01
peptide metabolic process	9	0.7	7.10E-03	1.40E-01
glycosaminoglycan metabolic process	9	0.7	9.80E-03	1.70E-01
sulfur compound biosynthetic process	9	0.7	1.10E-02	1.90E-01
regulation of intracellular transport	9	0.7	1.30E-02	2.10E-01
positive regulation of cell adhesion	9	0.7	1.50E-02	2.30E-01
regulation of cell shape	9	0.7	2.50E-02	3.00E-01
mesenchymal cell differentiation	9	0.7	3.10E-02	3.50E-01
regulation of heart contraction	9	0.7	3.50E-02	3.80E-01
mesenchyme development	9	0.7	3.50E-02	3.80E-01
regulation of catabolic process	9	0.7	3.50E-02	3.80E-01
activation of MAPK activity	9	0.7	3.50E-02	3.80E-01
cardiac muscle tissue development	9	0.7	6.70E-02	5.60E-01
mesoderm development	9	0.7	7.90E-02	6.10E-01
Ras protein signal transduction	9	0.7	7.90E-02	6.10E-01
adult locomotory behavior	9	0.7	9.90E-02	6.70E-01

purine ribonucleoside monophosphate biosynthetic process	8	0.6	1.70E-05	1.00E-03
purine nucleoside monophosphate biosynthetic process	8	0.6	1.70E-05	1.00E-03
purine nucleoside monophosphate metabolic process	8	0.6	5.60E-05	3.00E-03
purine ribonucleoside monophosphate metabolic process	8	0.6	5.60E-05	3.00E-03
glutamine metabolic process	8	0.6	3.30E-04	1.50E-02
hydrogen peroxide metabolic process	8	0.6	4.80E-04	2.00E-02
negative regulation of actin filament depolymerization	8	0.6	6.70E-04	2.70E-02
negative regulation of actin filament polymerization	8	0.6	2.10E-03	6.00E-02
salivary gland morphogenesis	8	0.6	3.40E-03	8.40E-02
regulation of intracellular protein transport	8	0.6	6.40E-03	1.30E-01
salivary gland development	8	0.6	7.80E-03	1.50E-01
regulation of protein catabolic process	8	0.6	9.30E-03	1.70E-01
proteoglycan metabolic process	8	0.6	1.10E-02	1.90E-01
ribonucleoprotein complex assembly	8	0.6	1.30E-02	2.10E-01
regulation of nucleocytoplasmic transport	8	0.6	1.50E-02	2.30E-01
dendrite development	8	0.6	2.70E-02	3.20E-01
cellular amide metabolic process	8	0.6	2.70E-02	3.20E-01
exocrine system development	8	0.6	3.40E-02	3.80E-01
cellular amino acid biosynthetic process	8	0.6	4.80E-02	4.70E-01
regulation of microtubule-based process	8	0.6	5.30E-02	5.00E-01
mesenchymal cell development	8	0.6	6.50E-02	5.50E-01
oxygen and reactive oxygen species metabolic process	8	0.6	6.50E-02	5.50E-01
cell recognition	8	0.6	6.50E-02	5.50E-01
actin filament bundle formation	7	0.5	5.40E-04	2.20E-02
cellular response to hydrogen peroxide	6	0.5	1.30E-03	4.30E-02
hydrogen peroxide catabolic process	6	0.5	1.30E-03	4.30E-02
branching involved in salivary gland morphogenesis	7	0.5	1.60E-03	4.90E-02
actin filament capping	7	0.5	2.30E-03	6.20E-02
oligosaccharide metabolic process	7	0.5	3.00E-03	7.70E-02
protein import into nucleus, docking	6	0.5	4.10E-03	9.50E-02
lysosome organization	7	0.5	5.10E-03	1.10E-01
response to hydrogen peroxide	7	0.5	8.10E-03	1.50E-01
nicotinamide nucleotide metabolic process	7	0.5	8.10E-03	1.50E-01
nicotinamide metabolic process	7	0.5	8.10E-03	1.50E-01
alkaloid metabolic process	7	0.5	8.10E-03	1.50E-01
cellular response to reactive oxygen species	6	0.5	9.60E-03	1.70E-01
positive regulation of cytoskeleton organization	7	0.5	1.20E-02	2.00E-01
nucleobase metabolic process	6	0.5	1.20E-02	2.00E-01
myofibril assembly	6	0.5	1.20E-02	2.00E-01
positive regulation of cell-substrate adhesion	7	0.5	1.50E-02	2.30E-01
cytokinesis	7	0.5	1.50E-02	2.30E-01
pyridine nucleotide metabolic process	7	0.5	1.70E-02	2.40E-01
collagen fibril organization	6	0.5	1.90E-02	2.50E-01
steroid hormone receptor signaling pathway	6	0.5	1.90E-02	2.50E-01
ER to Golgi vesicle-mediated transport	7	0.5	2.10E-02	2.70E-01
cellular response to oxidative stress	6	0.5	2.30E-02	2.80E-01
glutathione metabolic process	6	0.5	3.30E-02	3.60E-01
hippocampus development	6	0.5	3.30E-02	3.60E-01
actomyosin structure organization	6	0.5	3.80E-02	4.00E-01
positive regulation of endocytosis	7	0.5	4.20E-02	4.30E-01
nuclear-transcribed mRNA catabolic process, nonsense-mediated decay	6	0.5	4.40E-02	4.50E-01
negative regulation of epithelial cell proliferation	6	0.5	5.10E-02	4.90E-01
mesoderm formation	7	0.5	5.40E-02	5.00E-01
triglyceride metabolic process	7	0.5	5.40E-02	5.00E-01
response to reactive oxygen species	7	0.5	5.40E-02	5.00E-01
cellular component assembly involved in morphogenesis	7	0.5	5.40E-02	5.00E-01
antigen processing and presentation of exogenous antigen	6	0.5	5.90E-02	5.20E-01

cerebral cortex development	7	0.5	6.00E-02	5.30E-01
muscle fiber development	7	0.5	6.70E-02	5.60E-01
mesoderm morphogenesis	7	0.5	6.70E-02	5.60E-01
oxidoreduction coenzyme metabolic process	7	0.5	6.70E-02	5.60E-01
cellular component disassembly	7	0.5	7.40E-02	5.90E-01
proteasomal ubiquitin-dependent protein catabolic process	6	0.5	7.50E-02	5.90E-01
regulation of transforming growth factor beta receptor signaling pathway	6	0.5	7.50E-02	5.90E-01
proteasomal protein catabolic process	6	0.5	7.50E-02	5.90E-01
formation of primary germ layer	7	0.5	8.20E-02	6.20E-01
regulation of cell-substrate adhesion	7	0.5	8.20E-02	6.20E-01
lactation	6	0.5	8.40E-02	6.20E-01
intracellular receptor-mediated signaling pathway	6	0.5	9.40E-02	6.60E-01
positive regulation of T cell differentiation	6	0.5	9.40E-02	6.60E-01
mRNA catabolic process	7	0.5	9.80E-02	6.70E-01
nucleobase biosynthetic process	5	0.4	3.10E-03	7.90E-02
purine base metabolic process	5	0.4	7.30E-03	1.40E-01
mannose metabolic process	5	0.4	7.30E-03	1.40E-01
myotube differentiation	5	0.4	1.00E-02	1.80E-01
cortical cytoskeleton organization	5	0.4	1.40E-02	2.20E-01
chondroitin sulfate metabolic process	5	0.4	1.80E-02	2.50E-01
NADP metabolic process	5	0.4	1.80E-02	2.50E-01
actin filament-based movement	5	0.4	2.40E-02	2.90E-01
chondroitin sulfate proteoglycan metabolic process	5	0.4	3.00E-02	3.40E-01
antigen processing and presentation of peptide antigen via MHC class I	5	0.4	3.60E-02	3.90E-01
heme metabolic process	5	0.4	5.20E-02	4.90E-01
cell-cell recognition	5	0.4	6.20E-02	5.40E-01
aspartate family amino acid metabolic process	5	0.4	8.30E-02	6.20E-01
neural crest cell migration	5	0.4	9.40E-02	6.60E-01
sulfur amino acid metabolic process	5	0.4	9.40E-02	6.60E-01
deoxyribonucleotide metabolic process	5	0.4	9.40E-02	6.60E-01
IMP biosynthetic process	4	0.3	1.70E-03	5.00E-02
IMP metabolic process	4	0.3	1.70E-03	5.00E-02
purine base biosynthetic process	4	0.3	4.00E-03	9.50E-02
cellular metabolic compound salvage	4	0.3	4.00E-03	9.50E-02
positive regulation of protein complex disassembly	4	0.3	7.50E-03	1.40E-01
cerebral cortex radially oriented cell migration	4	0.3	1.90E-02	2.60E-01
pentose-phosphate shunt	4	0.3	1.90E-02	2.60E-01
chaperone mediated protein folding requiring cofactor	4	0.3	5.80E-02	5.20E-01
peptidyl-lysine modification	4	0.3	5.80E-02	5.20E-01
receptor clustering	4	0.3	7.20E-02	5.80E-01
ER-associated protein catabolic process	4	0.3	7.20E-02	5.80E-01
membrane protein proteolysis	4	0.3	7.20E-02	5.80E-01
membrane protein ectodomain proteolysis	4	0.3	7.20E-02	5.80E-01
'de novo' protein folding	4	0.3	8.60E-02	6.30E-01
vascular endothelial growth factor receptor signaling pathway	4	0.3	8.60E-02	6.30E-01
cell-substrate junction assembly	4	0.3	8.60E-02	6.30E-01
'de novo' posttranslational protein folding	4	0.3	8.60E-02	6.30E-01
'de novo' IMP biosynthetic process	3	0.2	1.70E-02	2.40E-01
myoblast proliferation	3	0.2	1.70E-02	2.40E-01
layer formation in the cerebral cortex	3	0.2	1.70E-02	2.40E-01
N-glycan processing	3	0.2	3.20E-02	3.60E-01
cerebral cortex radial glia guided migration	3	0.2	3.20E-02	3.60E-01
skeletal myofibril assembly	3	0.2	5.00E-02	4.80E-01
induction of apoptosis by oxidative stress	3	0.2	5.00E-02	4.80E-01
ruffle organization	3	0.2	5.00E-02	4.80E-01
dichotomous subdivision of terminal units involved in salivary gland branching	3	0.2	5.00E-02	4.80E-01
cardiac myofibril assembly	3	0.2	7.10E-02	5.80E-01

peptidyl-lysine modification to hypusine	3	0.2	7.10E-02	5.80E-01
purine deoxyribonucleotide metabolic process	3	0.2	7.10E-02	5.80E-01
hypusine metabolic process	3	0.2	7.10E-02	5.80E-01
dichotomous subdivision of an epithelial terminal unit	3	0.2	7.10E-02	5.80E-01
ribosomal small subunit biogenesis	3	0.2	7.10E-02	5.80E-01
purine deoxyribonucleoside triphosphate metabolic process	3	0.2	7.10E-02	5.80E-01
glycolipid catabolic process	3	0.2	7.10E-02	5.80E-01
L-serine metabolic process	3	0.2	9.50E-02	6.60E-01
purine nucleoside biosynthetic process	3	0.2	9.50E-02	6.60E-01
regulation of centrosome cycle	3	0.2	9.50E-02	6.60E-01
ribonucleoside biosynthetic process	3	0.2	9.50E-02	6.60E-01
regulation of protein export from nucleus	3	0.2	9.50E-02	6.60E-01
androgen receptor signaling pathway	3	0.2	9.50E-02	6.60E-01
nucleoside biosynthetic process	3	0.2	9.50E-02	6.60E-01
purine ribonucleoside biosynthetic process	3	0.2	9.50E-02	6.60E-01
positive regulation of mitotic cell cycle	3	0.2	9.50E-02	6.60E-01

Supplementary Table S6. GO analysis of identified HCPs in the culture supernatants during fed-batch culture.

Fed-batch culture				
Day3_CC				
Term	Count	%	P-Value	Benjamini
intracellular non-membrane-bounded organelle	232	19.8	2.20E-16	3.30E-14
non-membrane-bounded organelle	232	19.8	2.20E-16	3.30E-14
extracellular region	161	13.8	1.40E-04	1.40E-03
cytoskeleton	142	12.1	2.90E-11	9.20E-10
plasma membrane part	136	11.6	6.00E-02	2.30E-01
membrane-enclosed lumen	115	9.8	6.90E-04	5.50E-03
intracellular organelle lumen	109	9.3	1.80E-03	1.30E-02
organelle lumen	109	9.3	1.90E-03	1.40E-02
cytoskeletal part	98	8.4	5.80E-08	1.20E-06
cytosol	96	8.2	6.90E-16	7.50E-14
ribonucleoprotein complex	95	8.1	1.00E-20	4.50E-18
extracellular region part	95	8.1	4.10E-07	7.40E-06
vesicle	86	7.4	6.50E-13	3.70E-11
Golgi apparatus	85	7.3	9.90E-07	1.60E-05
cytoplasmic vesicle	83	7.1	3.80E-12	1.50E-10
endoplasmic reticulum	80	6.8	9.80E-03	5.60E-02
membrane-bounded vesicle	73	6.2	4.60E-12	1.70E-10
cytoplasmic membrane-bounded vesicle	72	6.2	6.40E-12	2.20E-10
endomembrane system	70	6	1.90E-06	2.90E-05
cell projection	70	6	2.30E-05	2.60E-04
cell fraction	66	5.6	6.20E-04	5.00E-03
extracellular matrix	54	4.6	3.30E-09	8.80E-08
insoluble fraction	54	4.6	1.00E-02	5.70E-02
proteinaceous extracellular matrix	53	4.5	2.20E-09	6.30E-08
vacuole	51	4.4	9.80E-15	6.30E-13
internal side of plasma membrane	51	4.4	5.40E-09	1.30E-07
microtubule cytoskeleton	52	4.4	1.00E-03	7.70E-03
extracellular space	52	4.4	1.30E-02	6.60E-02
membrane fraction	49	4.2	3.90E-02	1.70E-01
lysosome	48	4.1	2.90E-15	2.60E-13
lytic vacuole	48	4.1	3.80E-15	2.80E-13
actin cytoskeleton	47	4	3.50E-12	1.60E-10
extrinsic to membrane	45	3.8	5.40E-02	2.20E-01
ribosome	43	3.7	7.30E-11	2.20E-09
pigment granule	38	3.2	2.00E-20	4.50E-18
melanosome	38	3.2	2.00E-20	4.50E-18
endoplasmic reticulum part	38	3.2	4.30E-06	6.00E-05
cell surface	36	3.1	4.80E-03	3.00E-02
Golgi apparatus part	35	3	4.30E-05	4.70E-04
microtubule	34	2.9	2.90E-04	2.50E-03
nucleolus	32	2.7	4.40E-02	1.90E-01
cell cortex	31	2.6	4.70E-09	1.20E-07
neuron projection	29	2.5	1.20E-02	6.10E-02
nuclear envelope	28	2.4	1.40E-05	1.70E-04
endoplasmic reticulum lumen	27	2.3	2.00E-12	9.90E-11
extracellular matrix part	25	2.1	2.50E-08	5.30E-07
cell leading edge	24	2.1	4.60E-06	6.30E-05
spliceosome	24	2.1	2.60E-05	3.00E-04
external side of plasma membrane	24	2.1	2.60E-02	1.20E-01
basement membrane	21	1.8	1.50E-07	2.80E-06
contractile fiber	21	1.8	1.30E-05	1.60E-04
proteasome complex	20	1.7	1.20E-08	2.80E-07
cell cortex part	20	1.7	7.30E-07	1.30E-05
contractile fiber part	20	1.7	1.00E-05	1.30E-04
membrane coat	19	1.6	7.70E-08	1.50E-06
coated membrane	19	1.6	7.70E-08	1.50E-06
myofibril	19	1.6	8.20E-05	8.40E-04
Golgi membrane	19	1.6	1.00E-02	5.70E-02

basolateral plasma membrane	19	1.6	1.40E-02	6.90E-02
cytosolic part	17	1.5	7.70E-06	1.00E-04
soluble fraction	17	1.5	2.00E-03	1.40E-02
sarcomere	16	1.4	5.70E-04	4.70E-03
apical part of cell	16	1.4	6.90E-02	2.60E-01
ribosomal subunit	15	1.3	2.30E-04	2.10E-03
nuclear pore	14	1.2	2.60E-04	2.30E-03
pore complex	14	1.2	2.10E-03	1.50E-02
axon	14	1.2	4.70E-02	2.00E-01
cortical cytoskeleton	13	1.1	3.80E-05	4.20E-04
extrinsic to plasma membrane	13	1.1	4.90E-04	4.10E-03
adherens junction	13	1.1	8.40E-02	3.00E-01
lamellipodium	12	1	5.30E-03	3.20E-02
stress fiber	11	0.9	1.90E-06	2.90E-05
actin filament bundle	11	0.9	3.00E-06	4.40E-05
actomyosin	11	0.9	1.10E-05	1.30E-04
coated pit	11	0.9	5.80E-05	6.10E-04
small ribosomal subunit	10	0.9	1.50E-04	1.40E-03
Golgi-associated vesicle	11	0.9	1.80E-04	1.60E-03
clathrin coat	10	0.9	5.50E-04	4.60E-03
ruffle	11	0.9	1.70E-03	1.30E-02
I band	11	0.9	4.40E-03	2.80E-02
Z disc	10	0.9	5.30E-03	3.20E-02
focal adhesion	11	0.9	7.50E-03	4.50E-02
nuclear periphery	10	0.9	8.10E-03	4.70E-02
cell-substrate adherens junction	11	0.9	1.20E-02	6.20E-02
myosin complex	11	0.9	1.20E-02	6.20E-02
cell-substrate junction	11	0.9	2.00E-02	9.50E-02
microtubule associated complex	10	0.9	1.00E-01	3.30E-01
cytosolic ribosome	8	0.7	1.60E-04	1.50E-03
vesicle coat	8	0.7	3.20E-03	2.20E-02
growth cone	8	0.7	7.50E-02	2.70E-01
site of polarized growth	8	0.7	7.50E-02	2.70E-01
coated vesicle membrane	8	0.7	8.80E-02	3.10E-01
chaperonin-containing T-complex	7	0.6	9.60E-07	1.60E-05
eukaryotic translation initiation factor 3 complex	7	0.6	9.20E-05	9.20E-04
proton-transporting two-sector ATPase complex, catalytic domain	7	0.6	9.00E-04	7.00E-03
Golgi-associated vesicle membrane	7	0.6	3.10E-03	2.10E-02
AP-type membrane coat adaptor complex	7	0.6	7.80E-03	4.60E-02
clathrin adaptor complex	7	0.6	7.80E-03	4.60E-02
actin filament	7	0.6	1.90E-02	9.20E-02
nuclear membrane	7	0.6	5.50E-02	2.20E-01
nuclear matrix	7	0.6	8.20E-02	2.90E-01
proton-transporting two-sector ATPase complex	7	0.6	8.20E-02	2.90E-01
late endosome	7	0.6	9.70E-02	3.30E-01
septin complex	6	0.5	2.30E-03	1.60E-02
septin cytoskeleton	6	0.5	2.30E-03	1.60E-02
collagen	6	0.5	9.90E-03	5.50E-02
neuromuscular junction	6	0.5	1.90E-02	9.10E-02
transport vesicle	6	0.5	5.60E-02	2.20E-01
ciliary rootlet	5	0.4	4.10E-03	2.70E-02
COPI vesicle coat	5	0.4	4.10E-03	2.70E-02
COPI coated vesicle membrane	5	0.4	4.10E-03	2.70E-02
COPI-coated vesicle	5	0.4	8.60E-03	4.90E-02
basal lamina	5	0.4	1.20E-02	6.10E-02
trans-Golgi network transport vesicle	5	0.4	3.80E-02	1.70E-01
filopodium	5	0.4	7.10E-02	2.70E-01
cortical actin cytoskeleton	5	0.4	7.10E-02	2.70E-01
trailing edge	4	0.3	3.40E-03	2.30E-02
uropod	4	0.3	3.40E-03	2.30E-02
cytosolic large ribosomal subunit	4	0.3	1.10E-02	5.80E-02
aminoacyl-tRNA synthetase multienzyme complex	3	0.3	1.50E-02	7.50E-02
proton-transporting V-type ATPase, V1 domain	4	0.3	1.60E-02	8.00E-02

nuclear lamina	4	0.3	1.60E-02	8.00E-02
lamin filament	3	0.3	2.90E-02	1.30E-01
proteasome accessory complex	3	0.3	4.60E-02	1.90E-01
vacuolar lumen	3	0.3	4.60E-02	1.90E-01
kinesin complex	4	0.3	5.20E-02	2.10E-01
immunological synapse	4	0.3	6.30E-02	2.50E-01
clathrin coat of coated pit	3	0.3	6.50E-02	2.50E-01
Arp2/3 protein complex	3	0.3	6.50E-02	2.50E-01
fibrillar collagen	3	0.3	8.70E-02	3.10E-01
cell division site part	3	0.3	8.70E-02	3.10E-01
cell division site	3	0.3	8.70E-02	3.10E-01
laminin complex	3	0.3	8.70E-02	3.10E-01
striated muscle thin filament	3	0.3	8.70E-02	3.10E-01
filamentous actin	4	0.3	9.10E-02	3.10E-01
nuclear inner membrane	4	0.3	9.10E-02	3.10E-01
Day3_MF				
Term	Count	%	P-Value	Benjamini
nucleotide binding	271	23.2	2.90E-21	2.70E-18
purine nucleotide binding	226	19.3	9.50E-16	2.30E-13
ribonucleotide binding	220	18.8	6.30E-16	2.10E-13
purine ribonucleotide binding	220	18.8	6.30E-16	2.10E-13
adenyl nucleotide binding	173	14.8	1.90E-09	1.40E-07
purine nucleoside binding	173	14.8	3.50E-09	2.50E-07
nucleoside binding	173	14.8	5.70E-09	3.50E-07
adenyl ribonucleotide binding	167	14.3	1.30E-09	1.10E-07
ATP binding	166	14.2	9.90E-10	9.20E-08
RNA binding	96	8.2	1.70E-10	2.00E-08
structural molecule activity	91	7.8	2.60E-19	1.20E-16
calcium ion binding	90	7.7	1.80E-04	5.50E-03
peptidase activity	70	6	3.60E-04	9.70E-03
peptidase activity, acting on L-amino acid peptides	68	5.8	3.10E-04	8.50E-03
cytoskeletal protein binding	65	5.6	6.00E-09	3.50E-07
GTP binding	59	5	3.60E-09	2.40E-07
guanyl ribonucleotide binding	59	5	9.30E-09	5.10E-07
guanyl nucleotide binding	59	5	9.30E-09	5.10E-07
endopeptidase activity	50	4.3	7.10E-04	1.70E-02
actin binding	49	4.2	4.80E-08	2.30E-06
carbohydrate binding	42	3.6	2.20E-04	6.50E-03
structural constituent of ribosome	41	3.5	3.30E-13	6.10E-11
ATPase activity	37	3.2	6.30E-04	1.50E-02
GTPase activity	34	2.9	8.60E-11	1.10E-08
ATPase activity, coupled	33	2.8	1.70E-04	5.50E-03
pattern binding	27	2.3	1.40E-06	6.20E-05
polysaccharide binding	27	2.3	1.40E-06	6.20E-05
translation factor activity, nucleic acid binding	26	2.2	2.10E-08	1.10E-06
metallopeptidase activity	26	2.2	2.30E-03	4.80E-02
unfolded protein binding	24	2.1	3.40E-10	3.50E-08
glycosaminoglycan binding	25	2.1	1.80E-06	7.60E-05
cofactor binding	24	2.1	6.40E-02	5.40E-01
ligase activity, forming carbon-nitrogen bonds	23	2	2.40E-02	2.90E-01
ligase activity, forming carbon-oxygen bonds	22	1.9	3.40E-12	5.30E-10
ligase activity, forming aminoacyl-tRNA and related compounds	22	1.9	3.40E-12	5.30E-10
aminoacyl-tRNA ligase activity	22	1.9	3.40E-12	5.30E-10
protein domain specific binding	21	1.8	6.60E-02	5.50E-01
motor activity	20	1.7	5.30E-03	9.70E-02
manganese ion binding	20	1.7	1.10E-02	1.70E-01
translation initiation factor activity	19	1.6	2.40E-07	1.10E-05
heparin binding	19	1.6	2.30E-05	8.40E-04
coenzyme binding	19	1.6	4.30E-02	4.30E-01
protein transporter activity	18	1.5	3.30E-06	1.30E-04
growth factor binding	16	1.4	1.70E-04	5.50E-03
metalloendopeptidase activity	16	1.4	1.80E-02	2.50E-01
helicase activity	16	1.4	4.80E-02	4.60E-01

exopeptidase activity	14	1.2	1.40E-03	3.20E-02
calmodulin binding	14	1.2	7.10E-02	5.60E-01
actin filament binding	13	1.1	1.50E-04	5.30E-03
ATPase activity, coupled to transmembrane movement of ions	13	1.1	3.80E-03	7.40E-02
purine NTP-dependent helicase activity	13	1.1	2.10E-02	2.70E-01
ATP-dependent helicase activity	13	1.1	2.10E-02	2.70E-01
ATPase activity, coupled to movement of substances	13	1.1	4.90E-02	4.50E-01
hydrolase activity, acting on acid anhydrides	13	1.1	4.90E-02	4.50E-01
ATPase activity, coupled to transmembrane movement of substances	13	1.1	4.90E-02	4.50E-01
P-P-bond-hydrolysis-driven transmembrane transporter activity	13	1.1	8.80E-02	6.20E-01
primary active transmembrane transporter activity	13	1.1	9.30E-02	6.40E-01
transferase activity, transferring alkyl or aryl (other than methyl) groups	12	1	1.30E-03	2.90E-02
cysteine-type endopeptidase activity	12	1	5.30E-03	9.70E-02
structural constituent of cytoskeleton	11	0.9	2.20E-05	8.60E-04
ribonucleoprotein binding	10	0.9	5.50E-04	1.40E-02
microtubule binding	10	0.9	3.10E-02	3.50E-01
tubulin binding	11	0.9	4.80E-02	4.50E-01
monovalent inorganic cation transmembrane transporter activity	11	0.9	1.00E-01	6.50E-01
tRNA binding	9	0.8	4.40E-05	1.60E-03
rRNA binding	9	0.8	2.00E-04	5.90E-03
cation-transporting ATPase activity	9	0.8	2.70E-04	7.60E-03
extracellular matrix binding	9	0.8	2.70E-04	7.60E-03
copper ion binding	9	0.8	7.30E-02	5.70E-01
glutathione transferase activity	8	0.7	2.00E-03	4.40E-02
intramolecular oxidoreductase activity	8	0.7	1.80E-02	2.50E-01
proton-transporting ATPase activity, rotational mechanism	7	0.6	4.10E-04	1.10E-02
insulin-like growth factor binding	7	0.6	4.00E-03	7.60E-02
integrin binding	7	0.6	7.80E-03	1.30E-01
aminopeptidase activity	7	0.6	1.40E-02	2.10E-01
extracellular matrix structural constituent	7	0.6	1.90E-02	2.50E-01
translation elongation factor activity	7	0.6	2.20E-02	2.80E-01
antioxidant activity	7	0.6	6.70E-02	5.50E-01
hydro-lyase activity	7	0.6	8.20E-02	6.10E-01
NAD or NADH binding	7	0.6	9.70E-02	6.50E-01
mannosidase activity	6	0.5	3.30E-03	6.60E-02
ribosome binding	6	0.5	6.00E-03	1.10E-01
intramolecular transferase activity	6	0.5	2.70E-02	3.10E-01
oxidoreductase activity, acting on paired donors, with incorporation or reduction of molecular o	6	0.5	6.30E-02	5.40E-01
oxidoreductase activity, acting on sulfur group of donors	6	0.5	8.80E-02	6.30E-01
intramolecular oxidoreductase activity, interconverting keto- and enol-groups	5	0.4	2.60E-03	5.40E-02
hydrogen ion transporting ATP synthase activity, rotational mechanism	5	0.4	6.10E-03	1.10E-01
ubiquitin protein ligase binding	5	0.4	1.50E-02	2.20E-01
phosphoprotein binding	5	0.4	3.80E-02	3.90E-01
actin-dependent ATPase activity	4	0.3	6.50E-03	1.10E-01
peroxiredoxin activity	4	0.3	1.10E-02	1.70E-01
procollagen-lysine 5-dioxygenase activity	3	0.3	1.50E-02	2.20E-01
alpha-mannosidase activity	4	0.3	1.60E-02	2.30E-01
intramolecular oxidoreductase activity, transposing S-S bonds	4	0.3	1.60E-02	2.30E-01
protein disulfide isomerase activity	4	0.3	1.60E-02	2.30E-01
platelet-derived growth factor binding	4	0.3	2.30E-02	2.80E-01
carbon-nitrogen ligase activity, with glutamine as amido-N-donor	4	0.3	2.30E-02	2.80E-01
peptidyl-lysine 5-dioxygenase activity	3	0.3	2.90E-02	3.30E-01
intramolecular transferase activity, phosphotransferases	4	0.3	3.10E-02	3.50E-01
protein kinase C binding	4	0.3	4.10E-02	4.20E-01
serine-type carboxypeptidase activity	3	0.3	4.60E-02	4.40E-01
GDP-dissociation inhibitor activity	3	0.3	4.60E-02	4.40E-01
serine-type exopeptidase activity	3	0.3	4.60E-02	4.40E-01
profilin binding	3	0.3	6.50E-02	5.40E-01
microfilament motor activity	3	0.3	8.70E-02	6.30E-01
oxidoreductase activity, acting on the CH-NH group of donors, NAD or NADP as acceptor	4	0.3	9.10E-02	6.30E-01
palmitoyl-CoA hydrolase activity	4	0.3	9.10E-02	6.30E-01
disulfide oxidoreductase activity	4	0.3	9.10E-02	6.30E-01
Day3_BP				

Term	Count	%	P-Value	Benjamini
protein localization	111	9.5	1.30E-13	1.90E-10
proteolysis	99	8.5	1.80E-03	5.90E-02
establishment of protein localization	95	8.1	2.70E-11	2.00E-08
protein transport	94	8	4.20E-11	1.80E-08
translation	92	7.9	3.40E-32	1.00E-28
intracellular transport	71	6.1	4.20E-11	1.50E-08
homeostatic process	70	6	1.80E-05	1.60E-03
cell adhesion	67	5.7	3.20E-05	2.30E-03
biological adhesion	67	5.7	3.30E-05	2.30E-03
vesicle-mediated transport	63	5.4	1.10E-06	1.70E-04
regulation of apoptosis	53	4.5	2.20E-02	3.20E-01
regulation of programmed cell death	53	4.5	2.80E-02	3.60E-01
regulation of cell death	53	4.5	3.00E-02	3.70E-01
cytoskeleton organization	51	4.4	1.70E-07	3.90E-05
cellular protein localization	50	4.3	2.80E-08	8.20E-06
cellular macromolecule localization	50	4.3	3.50E-08	9.30E-06
protein catabolic process	49	4.2	9.00E-02	6.60E-01
intracellular protein transport	47	4	4.40E-08	1.10E-05
cellular homeostasis	47	4	2.10E-05	1.80E-03
macromolecular complex subunit organization	47	4	1.10E-04	7.10E-03
RNA processing	47	4	4.60E-03	1.20E-01
macromolecular complex assembly	45	3.8	6.60E-05	4.40E-03
nitrogen compound biosynthetic process	43	3.7	2.00E-05	1.80E-03
actin filament-based process	41	3.5	2.90E-11	1.70E-08
mRNA metabolic process	41	3.5	9.60E-05	6.10E-03
cell motion	41	3.5	4.40E-03	1.20E-01
cellular component morphogenesis	39	3.3	6.00E-03	1.40E-01
cellular macromolecular complex subunit organization	37	3.2	2.40E-05	2.00E-03
small GTPase mediated signal transduction	37	3.2	7.30E-05	4.80E-03
actin cytoskeleton organization	36	3.1	3.60E-09	1.20E-06
mRNA processing	36	3.1	2.20E-04	1.10E-02
cellular macromolecular complex assembly	35	3	1.00E-05	1.10E-03
protein complex assembly	35	3	2.70E-05	2.10E-03
protein complex biogenesis	35	3	2.70E-05	2.10E-03
protein folding	34	2.9	3.60E-11	1.80E-08
chemical homeostasis	34	2.9	9.00E-02	6.50E-01
membrane organization	33	2.8	3.30E-03	9.40E-02
localization of cell	33	2.8	6.30E-03	1.50E-01
cell motility	33	2.8	6.30E-03	1.50E-01
cell morphogenesis	33	2.8	2.00E-02	3.10E-01
cell projection organization	33	2.8	3.00E-02	3.70E-01
response to wounding	33	2.8	7.60E-02	6.10E-01
nucleobase, nucleoside, nucleotide and nucleic acid biosynthetic process	32	2.7	3.10E-06	4.10E-04
nucleobase, nucleoside and nucleotide biosynthetic process	32	2.7	3.10E-06	4.10E-04
nucleotide biosynthetic process	31	2.6	4.80E-06	6.20E-04
RNA splicing	30	2.6	2.00E-04	1.00E-02
cell proliferation	30	2.6	5.20E-03	1.30E-01
regulation of phosphate metabolic process	31	2.6	3.70E-02	4.20E-01
regulation of phosphorus metabolic process	31	2.6	3.70E-02	4.20E-01
regulation of phosphorylation	30	2.6	3.90E-02	4.30E-01
positive regulation of molecular function	31	2.6	4.40E-02	4.60E-01
tissue morphogenesis	29	2.5	5.80E-03	1.40E-01
cell migration	29	2.5	6.50E-03	1.50E-01
ncRNA metabolic process	28	2.4	1.10E-03	4.00E-02
negative regulation of apoptosis	28	2.4	1.10E-02	2.10E-01
negative regulation of programmed cell death	28	2.4	1.40E-02	2.50E-01
negative regulation of cell death	28	2.4	1.50E-02	2.60E-01
ion homeostasis	28	2.4	9.80E-02	6.80E-01
endocytosis	27	2.3	8.00E-04	3.30E-02
membrane invagination	27	2.3	8.00E-04	3.30E-02
generation of precursor metabolites and energy	27	2.3	5.00E-02	4.90E-01
positive regulation of catalytic activity	27	2.3	5.00E-02	4.90E-01

ribonucleotide metabolic process	26	2.2	1.90E-06	2.80E-04
muscle organ development	26	2.2	6.80E-04	2.80E-02
microtubule-based process	26	2.2	8.00E-03	1.70E-01
cellular ion homeostasis	26	2.2	7.80E-02	6.10E-01
cellular chemical homeostasis	26	2.2	9.70E-02	6.80E-01
cellular protein complex assembly	25	2.1	4.20E-07	8.10E-05
ribonucleotide biosynthetic process	25	2.1	7.10E-07	1.20E-04
tRNA metabolic process	25	2.1	1.00E-06	1.60E-04
purine nucleotide biosynthetic process	25	2.1	3.20E-05	2.30E-03
regulation of organelle organization	25	2.1	2.10E-04	1.10E-02
extracellular structure organization	24	2.1	3.30E-04	1.50E-02
purine nucleotide metabolic process	25	2.1	3.90E-04	1.70E-02
regulation of cellular component size	24	2.1	1.00E-03	3.80E-02
hexose metabolic process	24	2.1	1.90E-03	6.30E-02
monosaccharide metabolic process	25	2.1	4.50E-03	1.20E-01
positive regulation of developmental process	25	2.1	1.70E-02	2.80E-01
purine ribonucleotide biosynthetic process	23	2	4.90E-06	6.00E-04
purine ribonucleotide metabolic process	23	2	2.90E-05	2.20E-03
protein targeting	23	2	1.60E-04	9.00E-03
regulation of kinase activity	23	2	1.80E-02	2.90E-01
regulation of transferase activity	23	2	2.60E-02	3.50E-01
tRNA aminoacylation for protein translation	22	1.9	7.70E-13	7.60E-10
amino acid activation	22	1.9	7.70E-13	7.60E-10
tRNA aminoacylation	22	1.9	7.70E-13	7.60E-10
positive regulation of cellular component organization	22	1.9	1.30E-04	7.60E-03
glycoprotein metabolic process	22	1.9	1.60E-03	5.60E-02
positive regulation of cell differentiation	22	1.9	1.30E-02	2.30E-01
cofactor metabolic process	22	1.9	1.90E-02	2.90E-01
cation homeostasis	22	1.9	2.10E-02	3.10E-01
gland development	22	1.9	4.00E-02	4.40E-01
regulation of cytoskeleton organization	21	1.8	1.70E-05	1.60E-03
regulation of protein kinase activity	21	1.8	4.10E-02	4.40E-01
positive regulation of cell communication	21	1.8	4.80E-02	4.80E-01
muscle tissue development	20	1.7	3.40E-03	9.50E-02
cellular cation homeostasis	20	1.7	1.10E-02	2.10E-01
positive regulation of signal transduction	20	1.7	3.60E-02	4.20E-01
nucleocytoplasmic transport	19	1.6	1.20E-04	7.60E-03
nuclear transport	19	1.6	1.60E-04	8.90E-03
extracellular matrix organization	19	1.6	2.40E-04	1.10E-02
muscle cell differentiation	19	1.6	1.50E-03	5.20E-02
striated muscle tissue development	19	1.6	3.70E-03	1.00E-01
regulation of leukocyte activation	19	1.6	2.50E-02	3.40E-01
regulation of cell activation	19	1.6	2.80E-02	3.60E-01
actin filament organization	18	1.5	1.80E-07	3.80E-05
carbohydrate catabolic process	17	1.5	1.50E-04	8.70E-03
regulation of cellular component biogenesis	17	1.5	4.80E-04	2.00E-02
striated muscle cell differentiation	17	1.5	4.80E-04	2.00E-02
microtubule-based movement	18	1.5	7.10E-04	2.90E-02
regulation of cell adhesion	17	1.5	8.90E-04	3.60E-02
positive regulation of kinase activity	17	1.5	2.20E-02	3.20E-01
coenzyme metabolic process	18	1.5	2.50E-02	3.40E-01
regulation of lymphocyte activation	18	1.5	2.70E-02	3.50E-01
positive regulation of transferase activity	17	1.5	3.00E-02	3.70E-01
posttranscriptional regulation of gene expression	18	1.5	3.30E-02	4.00E-01
glucose metabolic process	17	1.5	4.00E-02	4.40E-01
regulation of protein kinase cascade	17	1.5	8.40E-02	6.30E-01
regulation of actin cytoskeleton organization	16	1.4	1.40E-05	1.40E-03
regulation of actin filament-based process	16	1.4	1.70E-05	1.70E-03
cell redox homeostasis	16	1.4	2.10E-05	1.80E-03
negative regulation of cellular component organization	16	1.4	2.20E-03	6.90E-02
purine nucleoside triphosphate metabolic process	16	1.4	7.80E-03	1.70E-01
regulation of cell motion	16	1.4	8.50E-03	1.80E-01
nucleoside triphosphate metabolic process	16	1.4	1.60E-02	2.70E-01

glycoprotein biosynthetic process	16	1.4	1.80E-02	2.90E-01
cellular di-, tri-valent inorganic cation homeostasis	16	1.4	5.30E-02	5.10E-01
ubiquitin-dependent protein catabolic process	16	1.4	7.50E-02	6.00E-01
di-, tri-valent inorganic cation homeostasis	16	1.4	9.50E-02	6.70E-01
regulation of actin polymerization or depolymerization	15	1.3	6.30E-06	7.40E-04
regulation of actin filament length	15	1.3	8.20E-06	9.20E-04
regulation of protein complex assembly	15	1.3	1.50E-04	8.70E-03
regulation of protein localization	15	1.3	4.30E-03	1.10E-01
sulfur metabolic process	15	1.3	6.40E-03	1.50E-01
purine nucleoside triphosphate biosynthetic process	15	1.3	6.40E-03	1.50E-01
nucleoside triphosphate biosynthetic process	15	1.3	7.00E-03	1.60E-01
regulation of T cell activation	15	1.3	2.00E-02	3.10E-01
positive regulation of protein kinase activity	15	1.3	5.70E-02	5.30E-01
morphogenesis of a branching structure	15	1.3	6.00E-02	5.40E-01
regulation of protein polymerization	14	1.2	1.40E-04	8.20E-03
nuclear import	14	1.2	1.70E-04	8.80E-03
regulation of vesicle-mediated transport	14	1.2	5.50E-03	1.30E-01
purine ribonucleoside triphosphate biosynthetic process	14	1.2	1.40E-02	2.50E-01
ribonucleoside triphosphate biosynthetic process	14	1.2	1.40E-02	2.50E-01
protein localization in organelle	14	1.2	1.90E-02	3.00E-01
regulation of cell morphogenesis	14	1.2	1.90E-02	3.00E-01
purine ribonucleoside triphosphate metabolic process	14	1.2	2.60E-02	3.50E-01
ribonucleoside triphosphate metabolic process	14	1.2	2.80E-02	3.60E-01
homeostasis of number of cells	14	1.2	5.70E-02	5.30E-01
T cell activation	14	1.2	6.80E-02	5.70E-01
regulation of protein complex disassembly	13	1.1	3.00E-05	2.30E-03
cellular carbohydrate catabolic process	13	1.1	9.00E-04	3.50E-02
negative regulation of immune system process	13	1.1	7.00E-03	1.50E-01
anti-apoptosis	13	1.1	2.10E-02	3.10E-01
biopolymer glycosylation	13	1.1	2.70E-02	3.60E-01
protein amino acid glycosylation	13	1.1	2.70E-02	3.60E-01
glycosylation	13	1.1	2.70E-02	3.60E-01
polysaccharide metabolic process	13	1.1	2.90E-02	3.70E-01
epithelial tube morphogenesis	13	1.1	9.50E-02	6.70E-01
regulation of actin filament polymerization	12	1	2.40E-04	1.10E-02
glucose catabolic process	12	1	8.90E-04	3.50E-02
hexose catabolic process	12	1	8.90E-04	3.50E-02
monosaccharide catabolic process	12	1	1.20E-03	4.50E-02
protein import into nucleus	12	1	1.70E-03	5.80E-02
muscle contraction	12	1	3.00E-03	8.80E-02
protein localization in nucleus	12	1	3.40E-03	9.60E-02
alcohol catabolic process	12	1	5.70E-03	1.40E-01
muscle system process	12	1	7.20E-03	1.60E-01
cell projection assembly	12	1	9.90E-03	2.00E-01
gastrulation	12	1	1.50E-02	2.60E-01
ATP biosynthetic process	12	1	3.00E-02	3.70E-01
protein import	12	1	3.00E-02	3.70E-01
anatomical structure homeostasis	12	1	3.20E-02	3.90E-01
gland morphogenesis	12	1	3.50E-02	4.10E-01
response to protein stimulus	12	1	4.40E-02	4.60E-01
response to oxidative stress	12	1	4.40E-02	4.60E-01
ATP metabolic process	12	1	5.30E-02	5.10E-01
regulation of cell growth	12	1	6.10E-02	5.40E-01
positive regulation of lymphocyte activation	12	1	6.90E-02	5.80E-01
positive regulation of leukocyte activation	12	1	9.20E-02	6.60E-01
cellular metal ion homeostasis	12	1	9.70E-02	6.80E-01
ribonucleoside monophosphate biosynthetic process	10	0.9	6.40E-07	1.20E-04
ribonucleoside monophosphate metabolic process	10	0.9	2.10E-06	3.00E-04
protein polymerization	11	0.9	3.70E-04	1.70E-02
vacuole organization	10	0.9	4.60E-04	2.00E-02
nucleoside monophosphate biosynthetic process	11	0.9	1.00E-03	3.80E-02
negative regulation of cytoskeleton organization	11	0.9	1.70E-03	5.70E-02
positive regulation of cell adhesion	10	0.9	2.80E-03	8.20E-02

positive regulation of organelle organization	11	0.9	3.20E-03	9.20E-02
regulation of endocytosis	11	0.9	3.20E-03	9.20E-02
glycolysis	10	0.9	3.30E-03	9.40E-02
striated muscle cell development	11	0.9	4.20E-03	1.10E-01
nucleoside monophosphate metabolic process	11	0.9	8.10E-03	1.70E-01
muscle cell development	11	0.9	1.00E-02	2.00E-01
negative regulation of organelle organization	11	0.9	1.10E-02	2.10E-01
negative regulation of cell activation	10	0.9	1.20E-02	2.20E-01
negative regulation of leukocyte activation	10	0.9	1.20E-02	2.20E-01
RNA transport	11	0.9	1.70E-02	2.80E-01
nucleic acid transport	11	0.9	1.70E-02	2.80E-01
establishment of RNA localization	11	0.9	1.70E-02	2.80E-01
RNA localization	11	0.9	1.90E-02	3.00E-01
regulation of lymphocyte differentiation	10	0.9	2.00E-02	3.10E-01
mesoderm development	10	0.9	2.20E-02	3.20E-01
nucleobase, nucleoside, nucleotide and nucleic acid transport	11	0.9	3.60E-02	4.10E-01
positive regulation of T cell activation	10	0.9	4.30E-02	4.50E-01
skeletal muscle tissue development	10	0.9	6.80E-02	5.70E-01
negative regulation of transport	10	0.9	6.80E-02	5.70E-01
skeletal muscle organ development	10	0.9	7.80E-02	6.10E-01
regulation of DNA binding	10	0.9	7.80E-02	6.10E-01
regulation of actin filament depolymerization	9	0.8	1.70E-04	8.80E-03
cytoskeleton-dependent intracellular transport	9	0.8	1.80E-03	5.90E-02
neuromuscular process controlling balance	9	0.8	2.20E-03	6.80E-02
negative regulation of protein complex disassembly	9	0.8	2.60E-03	8.00E-02
translational initiation	9	0.8	4.50E-03	1.20E-01
glutamine family amino acid metabolic process	9	0.8	9.80E-03	2.00E-01
regulation of cell shape	9	0.8	1.70E-02	2.80E-01
regulation of T cell differentiation	9	0.8	1.90E-02	3.00E-01
regulation of heart contraction	9	0.8	2.40E-02	3.30E-01
negative regulation of lymphocyte activation	9	0.8	2.90E-02	3.70E-01
aminoglycan metabolic process	9	0.8	2.90E-02	3.70E-01
nucleoside metabolic process	9	0.8	4.30E-02	4.50E-01
cardiac muscle tissue development	9	0.8	4.70E-02	4.80E-01
regulation of transcription factor activity	9	0.8	5.60E-02	5.20E-01
neuromuscular process	9	0.8	6.10E-02	5.40E-01
adult locomotory behavior	9	0.8	7.10E-02	5.90E-01
regulation of epithelial cell proliferation	9	0.8	7.60E-02	6.00E-01
regulation of protein transport	9	0.8	8.80E-02	6.50E-01
purine ribonucleoside monophosphate biosynthetic process	8	0.7	1.00E-05	1.10E-03
purine nucleoside monophosphate biosynthetic process	8	0.7	1.00E-05	1.10E-03
purine ribonucleoside monophosphate metabolic process	8	0.7	3.50E-05	2.40E-03
purine nucleoside monophosphate metabolic process	8	0.7	3.50E-05	2.40E-03
glutamine metabolic process	8	0.7	2.10E-04	1.10E-02
salivary gland morphogenesis	8	0.7	2.20E-03	6.90E-02
negative regulation of protein polymerization	8	0.7	2.20E-03	6.90E-02
negative regulation of protein complex assembly	8	0.7	2.20E-03	6.90E-02
salivary gland development	8	0.7	5.20E-03	1.30E-01
positive regulation of T cell differentiation	8	0.7	6.20E-03	1.50E-01
positive regulation of lymphocyte differentiation	8	0.7	8.80E-03	1.80E-01
mesoderm formation	8	0.7	1.20E-02	2.20E-01
mesoderm morphogenesis	8	0.7	1.60E-02	2.70E-01
formation of primary germ layer	8	0.7	2.10E-02	3.10E-01
exocrine system development	8	0.7	2.40E-02	3.30E-01
ion transmembrane transport	8	0.7	2.70E-02	3.60E-01
regulation of intracellular transport	8	0.7	2.70E-02	3.60E-01
cellular amino acid biosynthetic process	8	0.7	3.40E-02	4.00E-01
activation of MAPK activity	8	0.7	6.20E-02	5.50E-01
regulation of catabolic process	8	0.7	6.20E-02	5.50E-01
proton transport	8	0.7	7.30E-02	6.00E-01
hydrogen transport	8	0.7	8.00E-02	6.20E-01
protein import into nucleus, docking	7	0.6	3.60E-04	1.70E-02
actin filament bundle formation	7	0.6	3.60E-04	1.70E-02

branching involved in salivary gland morphogenesis	7	0.6	1.10E-03	4.10E-02
hydrogen peroxide metabolic process	7	0.6	2.10E-03	6.60E-02
oligosaccharide metabolic process	7	0.6	2.10E-03	6.60E-02
negative regulation of actin filament depolymerization	7	0.6	2.70E-03	8.20E-02
lysosome organization	7	0.6	3.50E-03	9.60E-02
actomyosin structure organization	7	0.6	6.90E-03	1.60E-01
negative regulation of actin filament polymerization	7	0.6	6.90E-03	1.60E-01
positive regulation of cytoskeleton organization	7	0.6	8.50E-03	1.80E-01
cytokinesis	7	0.6	1.00E-02	2.00E-01
regulation of intracellular protein transport	7	0.6	1.70E-02	2.80E-01
proteasomal protein catabolic process	7	0.6	1.70E-02	2.80E-01
proteasomal ubiquitin-dependent protein catabolic process	7	0.6	1.70E-02	2.80E-01
regulation of protein catabolic process	7	0.6	2.30E-02	3.30E-01
positive regulation of endocytosis	7	0.6	3.10E-02	3.70E-01
cellular component assembly involved in morphogenesis	7	0.6	3.90E-02	4.30E-01
energy coupled proton transport, down electrochemical gradient	7	0.6	4.40E-02	4.60E-01
ATP synthesis coupled proton transport	7	0.6	4.40E-02	4.60E-01
peptide metabolic process	7	0.6	5.00E-02	4.90E-01
muscle fiber development	7	0.6	5.00E-02	4.90E-01
cellular component disassembly	7	0.6	5.50E-02	5.20E-01
glycosaminoglycan metabolic process	7	0.6	6.10E-02	5.50E-01
negative regulation of T cell activation	7	0.6	7.40E-02	6.00E-01
myotube differentiation	6	0.5	9.30E-04	3.60E-02
actin filament capping	6	0.5	9.00E-03	1.80E-01
myofibril assembly	6	0.5	9.00E-03	1.80E-01
nucleobase metabolic process	6	0.5	9.00E-03	1.80E-01
collagen fibril organization	6	0.5	1.40E-02	2.50E-01
steroid hormone receptor signaling pathway	6	0.5	1.40E-02	2.50E-01
response to hydrogen peroxide	6	0.5	2.40E-02	3.40E-01
negative regulation of secretion	6	0.5	3.90E-02	4.30E-01
positive regulation of cell-substrate adhesion	6	0.5	3.90E-02	4.30E-01
antigen processing and presentation of exogenous antigen	6	0.5	4.50E-02	4.60E-01
regulation of transforming growth factor beta receptor signaling pathway	6	0.5	5.80E-02	5.30E-01
establishment or maintenance of cell polarity	6	0.5	6.50E-02	5.60E-01
lactation	6	0.5	6.50E-02	5.60E-01
intracellular receptor-mediated signaling pathway	6	0.5	7.30E-02	6.00E-01
T cell differentiation in the thymus	6	0.5	7.30E-02	6.00E-01
negative regulation of lymphocyte proliferation	6	0.5	9.90E-02	6.80E-01
negative regulation of leukocyte proliferation	6	0.5	9.90E-02	6.80E-01
negative regulation of mononuclear cell proliferation	6	0.5	9.90E-02	6.80E-01
regulation of nucleocytoplasmic transport	6	0.5	9.90E-02	6.80E-01
hydrogen peroxide catabolic process	5	0.4	7.90E-03	1.70E-01
cellular response to hydrogen peroxide	5	0.4	7.90E-03	1.70E-01
cortical cytoskeleton organization	5	0.4	1.10E-02	2.10E-01
actin filament-based movement	5	0.4	1.80E-02	2.90E-01
lamellipodium assembly	5	0.4	2.30E-02	3.30E-01
T cell homeostasis	5	0.4	3.50E-02	4.10E-01
cellular response to reactive oxygen species	5	0.4	3.50E-02	4.10E-01
regulation of nitric oxide biosynthetic process	5	0.4	4.20E-02	4.50E-01
positive regulation of alpha-beta T cell differentiation	5	0.4	4.20E-02	4.50E-01
serine family amino acid metabolic process	5	0.4	6.60E-02	5.70E-01
lung alveolus development	5	0.4	6.60E-02	5.70E-01
cellular response to oxidative stress	5	0.4	6.60E-02	5.70E-01
regulation of alpha-beta T cell differentiation	5	0.4	6.60E-02	5.70E-01
negative regulation of inflammatory response	5	0.4	6.60E-02	5.70E-01
deoxyribonucleotide metabolic process	5	0.4	7.60E-02	6.10E-01
positive regulation of myeloid cell differentiation	5	0.4	9.70E-02	6.80E-01
regulation of phagocytosis	5	0.4	9.70E-02	6.80E-01
protein amino acid N-linked glycosylation	5	0.4	9.70E-02	6.80E-01
IMP metabolic process	4	0.3	1.40E-03	4.80E-02
IMP biosynthetic process	4	0.3	1.40E-03	4.80E-02
positive regulation of protein complex disassembly	4	0.3	6.10E-03	1.40E-01
'de novo' IMP biosynthetic process	3	0.3	1.40E-02	2.50E-01

myoblast proliferation	3	0.3	1.40E-02	2.50E-01
nucleobase biosynthetic process	4	0.3	2.20E-02	3.20E-01
ribosomal protein import into nucleus	3	0.3	2.70E-02	3.60E-01
N-glycan processing	3	0.3	2.70E-02	3.60E-01
cellular copper ion homeostasis	4	0.3	2.90E-02	3.70E-01
mannose metabolic process	4	0.3	3.80E-02	4.30E-01
copper ion homeostasis	4	0.3	3.80E-02	4.30E-01
dichotomous subdivision of terminal units involved in salivary gland branching	3	0.3	4.40E-02	4.60E-01
skeletal myofibril assembly	3	0.3	4.40E-02	4.60E-01
ruffle organization	3	0.3	4.40E-02	4.60E-01
glycine metabolic process	4	0.3	4.80E-02	4.80E-01
chaperone mediated protein folding requiring cofactor	4	0.3	4.80E-02	4.80E-01
membrane protein ectodomain proteolysis	4	0.3	6.00E-02	5.40E-01
membrane protein proteolysis	4	0.3	6.00E-02	5.40E-01
ER-associated protein catabolic process	4	0.3	6.00E-02	5.40E-01
positive regulation of myeloid leukocyte differentiation	4	0.3	6.00E-02	5.40E-01
cardiac myofibril assembly	3	0.3	6.20E-02	5.50E-01
dichotomous subdivision of an epithelial terminal unit	3	0.3	6.20E-02	5.50E-01
purine deoxyribonucleotide metabolic process	3	0.3	6.20E-02	5.50E-01
glycolipid catabolic process	3	0.3	6.20E-02	5.50E-01
notochord morphogenesis	3	0.3	6.20E-02	5.50E-01
purine deoxyribonucleoside triphosphate metabolic process	3	0.3	6.20E-02	5.50E-01
cell-substrate junction assembly	4	0.3	7.20E-02	5.90E-01
'de novo' posttranslational protein folding	4	0.3	7.20E-02	5.90E-01
'de novo' protein folding	4	0.3	7.20E-02	5.90E-01
syncytium formation by plasma membrane fusion	3	0.3	8.30E-02	6.30E-01
antigen processing and presentation via MHC class Ib	3	0.3	8.30E-02	6.30E-01
myoblast fusion	3	0.3	8.30E-02	6.30E-01
L-serine metabolic process	3	0.3	8.30E-02	6.30E-01
positive regulation of smooth muscle cell proliferation	4	0.3	8.60E-02	6.40E-01
positive regulation of translation	4	0.3	8.60E-02	6.40E-01
positive regulation of nitric oxide biosynthetic process	4	0.3	8.60E-02	6.40E-01
Day8_CC				
Term	Count	%	P-Value	Benjamini
intracellular non-membrane-bounded organelle	258	19.4	3.40E-18	4.00E-16
non-membrane-bounded organelle	258	19.4	3.40E-18	4.00E-16
extracellular region	164	12.3	7.70E-03	4.20E-02
cytoskeleton	151	11.3	2.80E-10	7.80E-09
plasma membrane part	149	11.2	8.30E-02	2.80E-01
membrane-enclosed lumen	134	10.1	3.80E-05	4.30E-04
intracellular organelle lumen	126	9.5	2.00E-04	2.00E-03
organelle lumen	126	9.5	2.30E-04	2.10E-03
mitochondrion	126	9.5	4.00E-02	1.60E-01
cytosol	119	8.9	9.10E-24	1.40E-21
ribonucleoprotein complex	112	8.4	1.30E-26	6.00E-24
cytoskeletal part	105	7.9	1.60E-07	3.00E-06
vesicle	99	7.4	1.00E-15	7.90E-14
cytoplasmic vesicle	96	7.2	5.60E-15	3.80E-13
extracellular region part	96	7.2	2.70E-05	3.20E-04
Golgi apparatus	95	7.1	2.00E-07	3.60E-06
endoplasmic reticulum	91	6.8	3.60E-03	2.40E-02
membrane-bounded vesicle	84	6.3	1.40E-14	8.00E-13
cytoplasmic membrane-bounded vesicle	83	6.2	1.70E-14	9.10E-13
endomembrane system	82	6.2	2.70E-08	6.40E-07
cell projection	83	6.2	3.10E-07	5.20E-06
cell fraction	74	5.6	2.50E-04	2.20E-03
insoluble fraction	60	4.5	7.70E-03	4.30E-02
internal side of plasma membrane	59	4.4	5.90E-11	1.70E-09
vacuole	56	4.2	6.00E-16	5.20E-14
extracellular matrix	55	4.1	5.70E-08	1.20E-06
microtubule cytoskeleton	55	4.1	2.40E-03	1.70E-02
membrane fraction	55	4.1	2.70E-02	1.20E-01

proteinaceous extracellular matrix	53	4	9.50E-08	1.90E-06
extracellular space	53	4	5.50E-02	2.00E-01
extrinsic to membrane	52	3.9	2.20E-02	1.00E-01
actin cytoskeleton	51	3.8	9.20E-13	3.60E-11
lysosome	49	3.7	4.60E-14	2.20E-12
lytic vacuole	49	3.7	5.80E-14	2.50E-12
ribosome	48	3.6	3.90E-12	1.40E-10
melanosome	45	3.4	5.50E-26	1.30E-23
pigment granule	45	3.4	5.50E-26	1.30E-23
Golgi apparatus part	41	3.1	2.40E-06	3.70E-05
endoplasmic reticulum part	41	3.1	3.80E-06	5.60E-05
nucleolus	39	2.9	7.20E-03	4.10E-02
microtubule	36	2.7	5.10E-04	4.10E-03
cell surface	36	2.7	2.40E-02	1.10E-01
spliceosome	34	2.6	6.30E-10	1.70E-08
neuron projection	34	2.6	2.80E-03	1.90E-02
cell cortex	31	2.3	6.10E-08	1.20E-06
nuclear envelope	30	2.3	1.40E-05	1.70E-04
cell leading edge	29	2.2	5.50E-08	1.20E-06
endoplasmic reticulum lumen	27	2	2.50E-11	7.90E-10
proteasome complex	25	1.9	4.80E-12	1.60E-10
extracellular matrix part	25	1.9	2.10E-07	3.60E-06
Golgi membrane	24	1.8	6.60E-04	5.20E-03
basolateral plasma membrane	22	1.7	4.90E-03	3.10E-02
coated membrane	21	1.6	1.30E-08	3.10E-07
membrane coat	21	1.6	1.30E-08	3.10E-07
basement membrane	21	1.6	9.00E-07	1.50E-05
cell cortex part	20	1.5	3.90E-06	5.60E-05
contractile fiber	20	1.5	2.00E-04	2.00E-03
soluble fraction	20	1.5	3.50E-04	2.90E-03
cytosolic part	18	1.4	7.40E-06	1.00E-04
contractile fiber part	19	1.4	1.60E-04	1.60E-03
myofibril	18	1.4	9.60E-04	7.30E-03
apical part of cell	18	1.4	5.00E-02	1.90E-01
coated vesicle	17	1.3	4.40E-02	1.80E-01
nuclear pore	16	1.2	5.40E-05	5.90E-04
ribosomal subunit	16	1.2	2.10E-04	2.00E-03
pore complex	16	1.2	6.70E-04	5.20E-03
cell soma	16	1.2	5.20E-02	2.00E-01
anchoring junction	16	1.2	7.40E-02	2.60E-01
ruffle	15	1.1	1.40E-05	1.70E-04
extrinsic to plasma membrane	14	1.1	3.70E-04	3.00E-03
lamellipodium	14	1.1	1.40E-03	1.10E-02
sarcomere	15	1.1	4.80E-03	3.00E-02
cytoplasmic vesicle part	15	1.1	2.10E-02	9.90E-02
cytoplasmic vesicle membrane	14	1.1	2.30E-02	1.10E-01
vesicle membrane	15	1.1	2.50E-02	1.10E-01
adherens junction	15	1.1	4.80E-02	1.90E-01
axon	15	1.1	5.20E-02	2.00E-01
cortical cytoskeleton	13	1	1.10E-04	1.20E-03
focal adhesion	13	1	1.80E-03	1.30E-02
cell-substrate adherens junction	13	1	3.30E-03	2.20E-02
cell-substrate junction	13	1	6.40E-03	3.70E-02
stress fiber	11	0.8	5.20E-06	7.30E-05
actin filament bundle	11	0.8	8.30E-06	1.10E-04
actomyosin	11	0.8	2.90E-05	3.30E-04
coated pit	11	0.8	1.50E-04	1.60E-03
vesicle coat	10	0.8	2.50E-04	2.30E-03
small ribosomal subunit	10	0.8	3.40E-04	2.90E-03
Golgi-associated vesicle	11	0.8	4.40E-04	3.60E-03
clathrin coat	10	0.8	1.20E-03	9.20E-03
nuclear periphery	11	0.8	5.40E-03	3.20E-02
coated vesicle membrane	11	0.8	9.50E-03	5.10E-02

I band	11	0.8	9.50E-03	5.10E-02
Z disc	10	0.8	1.10E-02	5.70E-02
site of polarized growth	10	0.8	2.10E-02	9.70E-02
growth cone	10	0.8	2.10E-02	9.70E-02
myosin complex	11	0.8	2.50E-02	1.10E-01
protein-DNA complex	11	0.8	8.20E-02	2.80E-01
actin filament	9	0.7	2.20E-03	1.50E-02
proton-transporting two-sector ATPase complex	9	0.7	1.80E-02	8.90E-02
eukaryotic translation initiation factor 3 complex	8	0.6	1.30E-05	1.60E-04
proton-transporting two-sector ATPase complex, catalytic domain	8	0.6	2.20E-04	2.10E-03
cytosolic ribosome	8	0.6	3.30E-04	2.90E-03
transport vesicle	8	0.6	7.40E-03	4.20E-02
organelle envelope lumen	8	0.6	4.10E-02	1.60E-01
nuclear matrix	8	0.6	5.10E-02	2.00E-01
chaperonin-containing T-complex	7	0.5	1.90E-06	2.90E-05
septin cytoskeleton	6	0.5	3.80E-03	2.50E-02
septin complex	6	0.5	3.80E-03	2.50E-02
filamentous actin	6	0.5	5.30E-03	3.30E-02
Golgi-associated vesicle membrane	7	0.5	5.40E-03	3.20E-02
proton-transporting V-type ATPase complex	6	0.5	1.20E-02	6.50E-02
AP-type membrane coat adaptor complex	7	0.5	1.30E-02	6.80E-02
clathrin adaptor complex	7	0.5	1.30E-02	6.80E-02
collagen	6	0.5	1.60E-02	7.80E-02
filopodium	6	0.5	2.90E-02	1.20E-01
neuromuscular junction	6	0.5	2.90E-02	1.20E-01
vacuolar part	7	0.5	6.10E-02	2.20E-01
heterotrimeric G-protein complex	7	0.5	6.10E-02	2.20E-01
endocytic vesicle	6	0.5	7.30E-02	2.50E-01
nuclear membrane	7	0.5	8.50E-02	2.80E-01
mitochondrial intermembrane space	6	0.5	9.20E-02	3.00E-01
large ribosomal subunit	7	0.5	9.30E-02	3.00E-01
proton-transporting V-type ATPase, V1 domain	5	0.4	2.30E-03	1.60E-02
COPI vesicle coat	5	0.4	6.20E-03	3.60E-02
ciliary rootlet	5	0.4	6.20E-03	3.60E-02
COPI coated vesicle membrane	5	0.4	6.20E-03	3.60E-02
COPI-coated vesicle	5	0.4	1.30E-02	6.60E-02
basal lamina	5	0.4	1.70E-02	8.40E-02
transport vesicle membrane	5	0.4	3.60E-02	1.50E-01
trans-Golgi network transport vesicle	5	0.4	5.30E-02	2.00E-01
proteasome core complex	5	0.4	7.40E-02	2.60E-01
cortical actin cytoskeleton	5	0.4	9.90E-02	3.10E-01
trailing edge	4	0.3	4.70E-03	3.00E-02
proteasome accessory complex	4	0.3	4.70E-03	3.00E-02
uropod	4	0.3	4.70E-03	3.00E-02
Arp2/3 protein complex	4	0.3	8.90E-03	4.90E-02
cytosolic large ribosomal subunit	4	0.3	1.50E-02	7.40E-02
nuclear lamina	4	0.3	2.20E-02	1.00E-01
kinesin complex	4	0.3	6.80E-02	2.40E-01
immunological synapse	4	0.3	8.30E-02	2.80E-01
proteasome activator complex	3	0.2	1.90E-02	9.00E-02
aminoacyl-tRNA synthetase multienzyme complex	3	0.2	1.90E-02	9.00E-02
lamin filament	3	0.2	3.60E-02	1.50E-01
vacuolar lumen	3	0.2	5.60E-02	2.10E-01
DNA replication factor A complex	3	0.2	5.60E-02	2.10E-01
clathrin coat of coated pit	3	0.2	8.00E-02	2.70E-01
Day8_MF				
Term	Count	%	P-Value	Benjamini
nucleotide binding	307	23	6.20E-25	6.30E-22
purine nucleotide binding	252	18.9	1.60E-17	5.40E-15
purine ribonucleotide binding	244	18.3	2.10E-17	5.40E-15
ribonucleotide binding	244	18.3	2.10E-17	5.40E-15
adenyl nucleotide binding	191	14.3	8.10E-10	6.90E-08
purine nucleoside binding	191	14.3	1.70E-09	1.10E-07

nucleoside binding	191	14.3	2.70E-09	1.60E-07
adenyl ribonucleotide binding	183	13.7	1.20E-09	9.10E-08
ATP binding	181	13.6	1.40E-09	9.50E-08
RNA binding	113	8.5	9.90E-14	1.70E-11
structural molecule activity	98	7.4	1.20E-19	6.20E-17
calcium ion binding	95	7.1	8.80E-04	2.20E-02
cytoskeletal protein binding	72	5.4	1.20E-09	8.60E-08
peptidase activity	70	5.3	6.60E-03	1.30E-01
GTP binding	68	5.1	4.60E-11	5.20E-09
guanyl nucleotide binding	68	5.1	1.40E-10	1.50E-08
guanyl ribonucleotide binding	68	5.1	1.40E-10	1.50E-08
peptidase activity, acting on L-amino acid peptides	67	5	8.20E-03	1.40E-01
actin binding	53	4	3.60E-08	2.10E-06
endopeptidase activity	50	3.8	7.00E-03	1.30E-01
structural constituent of ribosome	46	3.5	5.80E-15	1.20E-12
ATPase activity	45	3.4	1.90E-05	7.90E-04
carbohydrate binding	42	3.2	2.10E-03	5.00E-02
magnesium ion binding	43	3.2	7.00E-02	5.50E-01
ATPase activity, coupled	41	3.1	1.60E-06	7.50E-05
GTPase activity	39	2.9	8.80E-13	1.30E-10
cofactor binding	32	2.4	2.90E-03	6.70E-02
unfolded protein binding	27	2	1.30E-11	1.60E-09
translation factor activity, nucleic acid binding	26	2	1.90E-07	9.80E-06
polysaccharide binding	27	2	1.10E-05	5.00E-04
pattern binding	27	2	1.10E-05	5.00E-04
coenzyme binding	27	2	5.30E-04	1.40E-02
ligase activity, forming carbon-nitrogen bonds	27	2	7.70E-03	1.40E-01
metallopeptidase activity	26	2	9.70E-03	1.60E-01
glycosaminoglycan binding	25	1.9	1.30E-05	5.40E-04
manganese ion binding	24	1.8	2.00E-03	4.80E-02
protein domain specific binding	24	1.8	4.00E-02	4.10E-01
ligase activity, forming aminoacyl-tRNA and related compounds	21	1.6	2.60E-10	2.40E-08
aminoacyl-tRNA ligase activity	21	1.6	2.60E-10	2.40E-08
ligase activity, forming carbon-oxygen bonds	21	1.6	2.60E-10	2.40E-08
protein transporter activity	21	1.6	1.50E-07	8.30E-06
helicase activity	21	1.6	3.90E-03	8.40E-02
motor activity	20	1.5	1.70E-02	2.30E-01
translation initiation factor activity	19	1.4	1.30E-06	6.30E-05
heparin binding	19	1.4	1.00E-04	3.30E-03
purine NTP-dependent helicase activity	18	1.4	5.00E-04	1.40E-02
ATP-dependent helicase activity	18	1.4	5.00E-04	1.40E-02
growth factor binding	17	1.3	1.80E-04	5.70E-03
ATPase activity, coupled to transmembrane movement of ions	16	1.2	3.70E-04	1.10E-02
hydrolase activity, acting on acid anhydrides	16	1.2	1.20E-02	1.80E-01
ATPase activity, coupled to movement of substances	16	1.2	1.20E-02	1.80E-01
ATPase activity, coupled to transmembrane movement of substances	16	1.2	1.20E-02	1.80E-01
P-P-bond-hydrolysis-driven transmembrane transporter activity	16	1.2	2.70E-02	3.20E-01
primary active transmembrane transporter activity	16	1.2	2.90E-02	3.40E-01
metalloendopeptidase activity	16	1.2	4.40E-02	4.20E-01
exopeptidase activity	14	1.1	3.90E-03	8.30E-02
actin filament binding	13	1	4.40E-04	1.30E-02
tubulin binding	13	1	2.00E-02	2.60E-01
monovalent inorganic cation transmembrane transporter activity	13	1	4.90E-02	4.60E-01
NAD or NADH binding	12	0.9	6.30E-04	1.70E-02
transferase activity, transferring alkyl or aryl (other than methyl) groups	12	0.9	3.20E-03	7.10E-02
structural constituent of cytoskeleton	11	0.8	5.90E-05	2.30E-03
rRNA binding	10	0.8	6.60E-05	2.50E-03
cation-transporting ATPase activity	10	0.8	9.50E-05	3.30E-03
extracellular matrix binding	10	0.8	9.50E-05	3.30E-03
ribonucleoprotein binding	11	0.8	2.70E-04	8.20E-03
intramolecular oxidoreductase activity	11	0.8	7.10E-04	1.80E-02
microtubule binding	11	0.8	2.50E-02	3.00E-01
cysteine-type endopeptidase activity	11	0.8	3.00E-02	3.40E-01

copper ion binding	10	0.8	5.80E-02	4.90E-01
tRNA binding	9	0.7	9.90E-05	3.30E-03
mRNA binding	9	0.7	7.00E-02	5.60E-01
ATPase activity, coupled to transmembrane movement of ions	9	0.7	9.00E-02	6.40E-01
proton-transporting ATPase activity, rotational mechanism	8	0.6	8.50E-05	3.10E-03
antioxidant activity	8	0.6	4.10E-02	4.10E-01
mannosidase activity	6	0.5	5.40E-03	1.10E-01
insulin-like growth factor binding	7	0.5	6.90E-03	1.30E-01
ribosome binding	6	0.5	9.70E-03	1.60E-01
intramolecular transferase activity	7	0.5	1.10E-02	1.70E-01
integrin binding	7	0.5	1.30E-02	2.00E-01
glutathione transferase activity	7	0.5	1.60E-02	2.30E-01
aminopeptidase activity	7	0.5	2.30E-02	2.90E-01
extracellular matrix structural constituent	7	0.5	3.20E-02	3.50E-01
translation elongation factor activity	7	0.5	3.70E-02	3.80E-01
oxidoreductase activity, acting on the CH-NH group of donors	6	0.5	4.80E-02	4.50E-01
carboxy-lyase activity	6	0.5	6.40E-02	5.30E-01
oxidoreductase activity	6	0.5	9.30E-02	6.40E-01
intramolecular oxidoreductase activity, interconverting keto- and enol-groups	5	0.4	4.00E-03	8.30E-02
intramolecular transferase activity, phosphotransferases	5	0.4	6.20E-03	1.20E-01
hydrogen ion transporting ATP synthase activity, rotational mechanism	5	0.4	9.10E-03	1.50E-01
sulfuric ester hydrolase activity	5	0.4	1.70E-02	2.40E-01
ubiquitin protein ligase binding	5	0.4	2.30E-02	2.90E-01
oxidoreductase activity, acting on the CH-NH group of donors, NAD or NADP as acceptor	5	0.4	2.90E-02	3.30E-01
phosphoprotein binding	5	0.4	5.40E-02	4.80E-01
threonine-type endopeptidase activity	5	0.4	8.70E-02	6.20E-01
threonine-type peptidase activity	5	0.4	8.70E-02	6.20E-01
actin-dependent ATPase activity	4	0.3	9.00E-03	1.50E-01
peroxiredoxin activity	4	0.3	1.50E-02	2.20E-01
intramolecular oxidoreductase activity, transposing S-S bonds	4	0.3	2.20E-02	2.90E-01
protein disulfide isomerase activity	4	0.3	2.20E-02	2.90E-01
alpha-mannosidase activity	4	0.3	2.20E-02	2.90E-01
carbon-nitrogen ligase activity, with glutamine as amido-N-donor	4	0.3	3.10E-02	3.50E-01
intramolecular oxidoreductase activity, interconverting aldoses and ketoses	4	0.3	3.10E-02	3.50E-01
platelet-derived growth factor binding	4	0.3	3.10E-02	3.50E-01
sodium:potassium-exchanging ATPase activity	4	0.3	4.20E-02	4.10E-01
protein kinase C binding	4	0.3	5.40E-02	4.80E-01
telomeric DNA binding	4	0.3	5.40E-02	4.80E-01
phosphoglucomutase activity	3	0.2	1.90E-02	2.50E-01
procollagen-lysine 5-dioxygenase activity	3	0.2	1.90E-02	2.50E-01
ADP binding	3	0.2	3.60E-02	3.80E-01
peptidyl-lysine 5-dioxygenase activity	3	0.2	3.60E-02	3.80E-01
proteasome regulator activity	3	0.2	3.60E-02	3.80E-01
proteasome activator activity	3	0.2	3.60E-02	3.80E-01
serine-type exopeptidase activity	3	0.2	5.60E-02	4.90E-01
GDP-dissociation inhibitor activity	3	0.2	5.60E-02	4.90E-01
serine-type carboxypeptidase activity	3	0.2	5.60E-02	4.90E-01
oxidoreductase activity, acting on sulfur group of donors, NAD or NADP as acceptor	3	0.2	8.00E-02	6.00E-01
profilin binding	3	0.2	8.00E-02	6.00E-01
transferase activity, transferring acyl groups, acyl groups converted into alkyl on transfer	3	0.2	8.00E-02	6.00E-01
Day8_BP				
Term	Count	%	P-Value	Benjamini
protein localization	125	9.4	1.40E-15	2.00E-12
establishment of protein localization	109	8.2	1.20E-13	1.20E-10
protein transport	108	8.1	1.70E-13	1.00E-10
proteolysis	103	7.7	1.20E-02	2.00E-01
translation	96	7.2	2.50E-31	7.80E-28
intracellular signaling cascade	87	6.5	5.50E-02	5.20E-01
intracellular transport	82	6.2	1.40E-13	1.10E-10
homeostatic process	73	5.5	1.10E-04	4.90E-03
cell adhesion	72	5.4	5.30E-05	2.70E-03
biological adhesion	72	5.4	5.70E-05	2.80E-03
vesicle-mediated transport	71	5.3	1.30E-07	2.00E-05

macromolecule catabolic process	65	4.9	4.60E-02	4.70E-01
oxidation reduction	64	4.8	9.40E-02	6.50E-01
RNA processing	63	4.7	4.90E-06	4.10E-04
regulation of apoptosis	62	4.7	5.30E-03	1.20E-01
regulation of programmed cell death	62	4.7	6.90E-03	1.40E-01
regulation of cell death	62	4.7	7.70E-03	1.50E-01
macromolecular complex subunit organization	60	4.5	1.30E-07	2.00E-05
cellular protein localization	58	4.4	3.70E-10	1.30E-07
cellular macromolecule localization	58	4.4	4.80E-10	1.30E-07
macromolecular complex assembly	57	4.3	9.50E-08	1.50E-05
mRNA metabolic process	56	4.2	4.20E-09	9.30E-07
intracellular protein transport	55	4.1	4.00E-10	1.20E-07
cytoskeleton organization	55	4.1	1.60E-07	2.30E-05
protein catabolic process	54	4.1	9.30E-02	6.50E-01
mRNA processing	50	3.8	1.20E-08	2.10E-06
cellular homeostasis	49	3.7	8.10E-05	3.70E-03
cellular macromolecular complex subunit organization	48	3.6	1.10E-08	1.90E-06
cellular macromolecular complex assembly	46	3.5	1.70E-09	4.40E-07
nitrogen compound biosynthetic process	46	3.5	2.90E-05	1.60E-03
cell motion	47	3.5	1.30E-03	4.00E-02
small GTPase mediated signal transduction	45	3.4	1.00E-06	1.10E-04
actin filament-based process	43	3.2	6.20E-11	2.70E-08
RNA splicing	43	3.2	4.80E-09	9.80E-07
protein complex biogenesis	41	3.1	1.30E-06	1.40E-04
protein complex assembly	41	3.1	1.30E-06	1.40E-04
cellular component morphogenesis	41	3.1	1.30E-02	2.10E-01
cellular response to stress	41	3.1	8.60E-02	6.30E-01
membrane organization	40	3	2.20E-04	9.60E-03
actin cytoskeleton organization	38	2.9	5.30E-09	1.00E-06
cell motility	38	2.9	1.90E-03	5.40E-02
localization of cell	38	2.9	1.90E-03	5.40E-02
cell projection organization	38	2.9	1.30E-02	2.10E-01
chemical homeostasis	39	2.9	5.20E-02	5.00E-01
generation of precursor metabolites and energy	37	2.8	7.90E-04	2.80E-02
protein folding	36	2.7	3.40E-11	1.70E-08
cell proliferation	36	2.7	5.60E-04	2.20E-02
nucleobase, nucleoside, nucleotide and nucleic acid biosynthetic process	34	2.6	4.10E-06	3.50E-04
nucleobase, nucleoside and nucleotide biosynthetic process	34	2.6	4.10E-06	3.50E-04
regulation of phosphorylation	34	2.6	2.30E-02	3.10E-01
regulation of phosphate metabolic process	35	2.6	2.30E-02	3.10E-01
regulation of phosphorus metabolic process	35	2.6	2.30E-02	3.10E-01
cell morphogenesis	35	2.6	3.30E-02	3.80E-01
positive regulation of molecular function	34	2.6	4.40E-02	4.60E-01
monosaccharide metabolic process	33	2.5	4.20E-05	2.20E-03
cell migration	33	2.5	2.60E-03	6.80E-02
hexose metabolic process	32	2.4	8.80E-06	6.40E-04
nucleotide biosynthetic process	32	2.4	1.60E-05	1.10E-03
negative regulation of apoptosis	32	2.4	4.60E-03	1.00E-01
negative regulation of programmed cell death	32	2.4	6.20E-03	1.30E-01
negative regulation of cell death	32	2.4	6.50E-03	1.30E-01
ion homeostasis	32	2.4	6.20E-02	5.50E-01
cellular protein complex assembly	30	2.3	3.20E-09	7.50E-07
ncRNA metabolic process	30	2.3	1.30E-03	4.00E-02
tissue morphogenesis	30	2.3	1.40E-02	2.20E-01
positive regulation of catalytic activity	30	2.3	4.10E-02	4.40E-01
neuron development	31	2.3	8.70E-02	6.30E-01
regulation of organelle organization	29	2.2	2.80E-05	1.60E-03
muscle organ development	29	2.2	3.00E-04	1.30E-02
endocytosis	29	2.2	8.90E-04	3.00E-02
membrane invagination	29	2.2	8.90E-04	3.00E-02
cellular ion homeostasis	29	2.2	6.30E-02	5.50E-01
cellular chemical homeostasis	29	2.2	8.20E-02	6.20E-01
ribonucleotide metabolic process	28	2.1	1.30E-06	1.40E-04

cofactor metabolic process	28	2.1	1.20E-03	3.70E-02
regulation of kinase activity	28	2.1	2.60E-03	6.70E-02
regulation of transferase activity	28	2.1	4.20E-03	9.80E-02
microtubule-based process	28	2.1	9.20E-03	1.70E-01
ribonucleotide biosynthetic process	27	2	4.00E-07	5.40E-05
protein targeting	27	2	1.40E-05	1.00E-03
purine nucleotide biosynthetic process	27	2	2.50E-05	1.50E-03
purine nucleotide metabolic process	27	2	3.50E-04	1.50E-02
regulation of cellular component size	26	2	9.00E-04	3.00E-02
regulation of protein kinase activity	26	2	6.50E-03	1.30E-01
positive regulation of developmental process	27	2	1.90E-02	2.80E-01
blood vessel development	27	2	7.60E-02	6.00E-01
vasculature development	27	2	9.50E-02	6.50E-01
regulation of cytoskeleton organization	25	1.9	5.70E-07	7.30E-05
purine ribonucleotide biosynthetic process	25	1.9	2.50E-06	2.40E-04
purine ribonucleotide metabolic process	25	1.9	1.80E-05	1.10E-03
extracellular structure organization	25	1.9	6.60E-04	2.40E-02
tRNA metabolic process	24	1.8	2.20E-05	1.30E-03
coenzyme metabolic process	24	1.8	8.70E-04	3.00E-02
glycoprotein metabolic process	24	1.8	1.30E-03	4.00E-02
positive regulation of cell differentiation	24	1.8	1.10E-02	2.00E-01
cation homeostasis	24	1.8	2.00E-02	2.80E-01
gland development	24	1.8	4.00E-02	4.30E-01
nucleocytoplasmic transport	22	1.7	1.60E-05	1.10E-03
nuclear transport	22	1.7	2.20E-05	1.40E-03
positive regulation of cellular component organization	23	1.7	2.20E-04	9.70E-03
glucose metabolic process	22	1.7	3.40E-03	8.20E-02
tRNA aminoacylation	21	1.6	6.40E-11	2.40E-08
amino acid activation	21	1.6	6.40E-11	2.40E-08
tRNA aminoacylation for protein translation	21	1.6	6.40E-11	2.40E-08
muscle tissue development	21	1.6	5.30E-03	1.20E-01
cellular cation homeostasis	21	1.6	1.70E-02	2.60E-01
carbohydrate catabolic process	20	1.5	1.40E-05	1.00E-03
regulation of cellular component biogenesis	20	1.5	5.70E-05	2.80E-03
muscle cell differentiation	20	1.5	2.10E-03	5.70E-02
striated muscle tissue development	20	1.5	5.40E-03	1.20E-01
positive regulation of kinase activity	20	1.5	6.90E-03	1.40E-01
positive regulation of transferase activity	20	1.5	1.00E-02	1.80E-01
posttranscriptional regulation of gene expression	20	1.5	2.50E-02	3.30E-01
positive regulation of signal transduction	20	1.5	8.90E-02	6.40E-01
actin filament organization	18	1.4	8.80E-07	1.00E-04
regulation of actin cytoskeleton organization	18	1.4	2.60E-06	2.40E-04
regulation of actin filament-based process	18	1.4	3.30E-06	3.00E-04
cell redox homeostasis	18	1.4	4.20E-06	3.60E-04
regulation of protein complex assembly	18	1.4	8.40E-06	6.30E-04
striated muscle cell differentiation	18	1.4	5.60E-04	2.20E-02
microtubule-based movement	19	1.4	9.20E-04	3.00E-02
extracellular matrix organization	19	1.4	9.20E-04	3.00E-02
regulation of cell adhesion	18	1.4	1.10E-03	3.40E-02
sulfur metabolic process	18	1.4	1.10E-03	3.40E-02
protein localization in organelle	18	1.4	1.50E-03	4.60E-02
purine nucleoside triphosphate metabolic process	18	1.4	4.00E-03	9.30E-02
regulation of cell motion	18	1.4	4.40E-03	1.00E-01
nucleoside triphosphate metabolic process	18	1.4	9.20E-03	1.70E-01
positive regulation of protein kinase activity	18	1.4	1.90E-02	2.70E-01
regulation of leukocyte activation	19	1.4	6.30E-02	5.50E-01
regulation of lymphocyte activation	18	1.4	6.40E-02	5.50E-01
regulation of cell activation	19	1.4	6.90E-02	5.70E-01
di-, tri-valent inorganic cation homeostasis	18	1.4	7.10E-02	5.80E-01
regulation of actin polymerization or depolymerization	17	1.3	8.30E-07	1.00E-04
regulation of actin filament length	17	1.3	1.10E-06	1.20E-04
regulation of protein polymerization	17	1.3	5.70E-06	4.50E-04
negative regulation of cellular component organization	17	1.3	2.50E-03	6.70E-02

purine nucleoside triphosphate biosynthetic process	17	1.3	2.80E-03	7.10E-02
nucleoside triphosphate biosynthetic process	17	1.3	3.10E-03	7.70E-02
glycoprotein biosynthetic process	17	1.3	2.20E-02	3.00E-01
angiogenesis	17	1.3	6.20E-02	5.50E-01
cellular di-, tri-valent inorganic cation homeostasis	17	1.3	6.50E-02	5.60E-01
carboxylic acid biosynthetic process	17	1.3	9.30E-02	6.50E-01
ubiquitin-dependent protein catabolic process	17	1.3	9.30E-02	6.50E-01
organic acid biosynthetic process	17	1.3	9.30E-02	6.50E-01
nuclear import	16	1.2	3.30E-05	1.80E-03
cellular carbohydrate catabolic process	16	1.2	5.00E-05	2.60E-03
protein import	16	1.2	1.80E-03	5.30E-02
regulation of vesicle-mediated transport	16	1.2	2.10E-03	5.70E-02
ribonucleoside triphosphate biosynthetic process	16	1.2	6.30E-03	1.30E-01
purine ribonucleoside triphosphate biosynthetic process	16	1.2	6.30E-03	1.30E-01
purine ribonucleoside triphosphate metabolic process	16	1.2	1.30E-02	2.20E-01
ribonucleoside triphosphate metabolic process	16	1.2	1.40E-02	2.30E-01
MAPKKK cascade	16	1.2	3.60E-02	4.10E-01
morphogenesis of a branching structure	16	1.2	7.00E-02	5.70E-01
regulation of protein complex disassembly	14	1.1	1.80E-05	1.10E-03
regulation of actin filament polymerization	14	1.1	3.10E-05	1.70E-03
monosaccharide catabolic process	15	1.1	5.80E-05	2.80E-03
hexose catabolic process	14	1.1	1.60E-04	7.10E-03
glucose catabolic process	14	1.1	1.60E-04	7.10E-03
protein import into nucleus	14	1.1	3.50E-04	1.50E-02
alcohol catabolic process	15	1.1	4.80E-04	1.90E-02
nucleic acid transport	15	1.1	5.60E-04	2.20E-02
establishment of RNA localization	15	1.1	5.60E-04	2.20E-02
RNA transport	15	1.1	5.60E-04	2.20E-02
RNA localization	15	1.1	6.60E-04	2.40E-02
protein localization in nucleus	14	1.1	8.40E-04	3.00E-02
nucleobase, nucleoside, nucleotide and nucleic acid transport	15	1.1	1.80E-03	5.30E-02
cell projection assembly	14	1.1	3.10E-03	7.70E-02
response to oxidative stress	15	1.1	8.30E-03	1.60E-01
anti-apoptosis	15	1.1	9.20E-03	1.70E-01
regulation of protein localization	15	1.1	1.10E-02	1.90E-01
ATP biosynthetic process	14	1.1	1.20E-02	2.00E-01
polysaccharide metabolic process	15	1.1	1.30E-02	2.20E-01
regulation of cell morphogenesis	15	1.1	2.10E-02	2.90E-01
ATP metabolic process	14	1.1	2.50E-02	3.30E-01
glycosylation	14	1.1	2.70E-02	3.40E-01
protein amino acid glycosylation	14	1.1	2.70E-02	3.40E-01
biopolymer glycosylation	14	1.1	2.70E-02	3.40E-01
energy derivation by oxidation of organic compounds	14	1.1	4.60E-02	4.70E-01
regulation of T cell activation	15	1.1	4.60E-02	4.70E-01
chromatin assembly or disassembly	15	1.1	5.00E-02	4.90E-01
DNA packaging	14	1.1	5.60E-02	5.20E-01
protein polymerization	13	1	4.10E-05	2.20E-03
regulation of endocytosis	13	1	6.20E-04	2.30E-02
muscle system process	13	1	6.00E-03	1.30E-01
gastrulation	13	1	1.30E-02	2.20E-01
negative regulation of immune system process	13	1	1.60E-02	2.50E-01
anatomical structure homeostasis	13	1	3.00E-02	3.70E-01
response to inorganic substance	13	1	6.00E-02	5.40E-01
negative regulation of cytoskeleton organization	12	0.9	1.10E-03	3.50E-02
positive regulation of organelle organization	12	0.9	2.20E-03	6.00E-02
striated muscle cell development	12	0.9	3.00E-03	7.50E-02
muscle contraction	12	0.9	7.00E-03	1.40E-01
muscle cell development	12	0.9	7.90E-03	1.50E-01
mRNA transport	12	0.9	8.90E-03	1.70E-01
negative regulation of organelle organization	12	0.9	8.90E-03	1.70E-01
regulation of MAP kinase activity	12	0.9	6.80E-02	5.70E-01
response to protein stimulus	12	0.9	8.30E-02	6.20E-01
response to drug	12	0.9	8.80E-02	6.30E-01

ribonucleoside monophosphate biosynthetic process	10	0.8	1.60E-06	1.60E-04
ribonucleoside monophosphate metabolic process	10	0.8	5.40E-06	4.40E-04
regulation of actin filament depolymerization	10	0.8	5.30E-05	2.70E-03
neuromuscular process controlling balance	10	0.8	1.00E-03	3.40E-02
vacuole organization	10	0.8	1.00E-03	3.40E-02
negative regulation of protein complex disassembly	10	0.8	1.30E-03	4.00E-02
positive regulation of cell adhesion	11	0.8	1.60E-03	4.80E-02
glycolysis	11	0.8	2.00E-03	5.50E-02
nucleoside monophosphate biosynthetic process	11	0.8	2.30E-03	6.30E-02
activation of MAPK activity	11	0.8	5.30E-03	1.20E-01
glutamine family amino acid metabolic process	10	0.8	5.80E-03	1.20E-01
negative regulation of leukocyte activation	11	0.8	8.00E-03	1.60E-01
negative regulation of cell activation	11	0.8	8.00E-03	1.60E-01
regulation of cell shape	10	0.8	1.10E-02	1.90E-01
regulation of catabolic process	10	0.8	1.60E-02	2.40E-01
nucleoside monophosphate metabolic process	11	0.8	1.70E-02	2.50E-01
positive regulation of MAP kinase activity	11	0.8	1.70E-02	2.50E-01
proton transport	10	0.8	2.00E-02	2.90E-01
negative regulation of lymphocyte activation	10	0.8	2.00E-02	2.90E-01
hydrogen transport	10	0.8	2.30E-02	3.00E-01
nucleoside metabolic process	10	0.8	3.10E-02	3.70E-01
mesoderm development	10	0.8	4.20E-02	4.50E-01
Ras protein signal transduction	10	0.8	4.20E-02	4.50E-01
neuromuscular process	10	0.8	4.60E-02	4.70E-01
response to steroid hormone stimulus	10	0.8	4.60E-02	4.70E-01
adult locomotory behavior	10	0.8	5.50E-02	5.20E-01
skeletal muscle tissue development	11	0.8	5.70E-02	5.30E-01
regulation of epithelial cell proliferation	10	0.8	6.00E-02	5.40E-01
nucleosome assembly	11	0.8	6.20E-02	5.50E-01
skeletal muscle organ development	11	0.8	6.70E-02	5.60E-01
chromatin assembly	11	0.8	7.20E-02	5.80E-01
protein-DNA complex assembly	11	0.8	7.70E-02	6.00E-01
nucleosome organization	11	0.8	7.70E-02	6.00E-01
protein modification by small protein conjugation	11	0.8	9.50E-02	6.50E-01
protein import into nucleus, docking	9	0.7	6.00E-06	4.60E-04
negative regulation of protein polymerization	9	0.7	8.70E-04	3.00E-02
negative regulation of protein complex assembly	9	0.7	8.70E-04	3.00E-02
proteasomal ubiquitin-dependent protein catabolic process	9	0.7	1.90E-03	5.30E-02
proteasomal protein catabolic process	9	0.7	1.90E-03	5.30E-02
regulation of protein catabolic process	9	0.7	2.90E-03	7.30E-02
cytoskeleton-dependent intracellular transport	9	0.7	3.60E-03	8.40E-02
translational initiation	9	0.7	8.80E-03	1.60E-01
ion transmembrane transport	9	0.7	1.60E-02	2.50E-01
regulation of intracellular transport	9	0.7	1.60E-02	2.50E-01
cellular amino acid biosynthetic process	9	0.7	2.10E-02	2.90E-01
nucleus organization	9	0.7	2.70E-02	3.40E-01
tissue homeostasis	9	0.7	4.20E-02	4.50E-01
regulation of heart contraction	9	0.7	4.20E-02	4.50E-01
aminoglycan metabolic process	9	0.7	5.10E-02	5.00E-01
Golgi vesicle transport	9	0.7	5.10E-02	5.00E-01
oxidative phosphorylation	9	0.7	7.30E-02	5.90E-01
cellular carbohydrate biosynthetic process	9	0.7	7.30E-02	5.90E-01
cardiac muscle tissue development	9	0.7	8.00E-02	6.10E-01
regulation of lymphocyte differentiation	9	0.7	8.60E-02	6.30E-01
cellular respiration	9	0.7	9.30E-02	6.50E-01
regulation of transcription factor activity	9	0.7	9.30E-02	6.50E-01
purine nucleoside monophosphate biosynthetic process	8	0.6	2.20E-05	1.40E-03
purine ribonucleoside monophosphate biosynthetic process	8	0.6	2.20E-05	1.40E-03
purine nucleoside monophosphate metabolic process	8	0.6	7.10E-05	3.40E-03
purine ribonucleoside monophosphate metabolic process	8	0.6	7.10E-05	3.40E-03
glutamine metabolic process	8	0.6	4.20E-04	1.70E-02
hydrogen peroxide metabolic process	8	0.6	6.00E-04	2.30E-02
negative regulation of actin filament depolymerization	8	0.6	8.40E-04	3.00E-02

negative regulation of actin filament polymerization	8	0.6	2.60E-03	6.80E-02
positive regulation of cytoskeleton organization	8	0.6	3.40E-03	8.10E-02
mesoderm formation	8	0.6	2.10E-02	2.90E-01
ATP synthesis coupled proton transport	8	0.6	2.50E-02	3.20E-01
energy coupled proton transport, down electrochemical gradient	8	0.6	2.50E-02	3.20E-01
muscle fiber development	8	0.6	2.80E-02	3.50E-01
mesoderm morphogenesis	8	0.6	2.80E-02	3.50E-01
peptide metabolic process	8	0.6	2.80E-02	3.50E-01
cellular component disassembly	8	0.6	3.20E-02	3.80E-01
formation of primary germ layer	8	0.6	3.60E-02	4.10E-01
sulfur compound biosynthetic process	8	0.6	4.10E-02	4.40E-01
negative regulation of T cell activation	8	0.6	4.60E-02	4.70E-01
regulation of microtubule-based process	8	0.6	6.30E-02	5.50E-01
oxygen and reactive oxygen species metabolic process	8	0.6	7.60E-02	6.00E-01
cell recognition	8	0.6	7.60E-02	6.00E-01
regulation of T cell differentiation	8	0.6	8.30E-02	6.20E-01
actin filament bundle formation	7	0.5	6.60E-04	2.50E-02
actin filament capping	7	0.5	2.70E-03	7.00E-02
oligosaccharide metabolic process	7	0.5	3.60E-03	8.50E-02
steroid hormone receptor signaling pathway	7	0.5	4.70E-03	1.10E-01
lysosome organization	7	0.5	6.10E-03	1.30E-01
response to hydrogen peroxide	7	0.5	9.60E-03	1.70E-01
branching involved in salivary gland morphogenesis	6	0.5	1.10E-02	1.90E-01
cellular response to reactive oxygen species	6	0.5	1.10E-02	1.90E-01
actomyosin structure organization	7	0.5	1.20E-02	2.00E-01
myofibril assembly	6	0.5	1.40E-02	2.30E-01
nucleobase metabolic process	6	0.5	1.40E-02	2.30E-01
salivary gland morphogenesis	7	0.5	1.70E-02	2.60E-01
cytokinesis	7	0.5	1.70E-02	2.60E-01
positive regulation of cell-substrate adhesion	7	0.5	1.70E-02	2.60E-01
aerobic respiration	7	0.5	1.70E-02	2.60E-01
collagen fibril organization	6	0.5	2.20E-02	3.00E-01
serine family amino acid metabolic process	6	0.5	2.60E-02	3.40E-01
lung alveolus development	6	0.5	2.60E-02	3.40E-01
cellular response to oxidative stress	6	0.5	2.60E-02	3.40E-01
regulation of intracellular protein transport	7	0.5	2.80E-02	3.50E-01
tricarboxylic acid cycle	6	0.5	3.20E-02	3.80E-01
acetyl-CoA metabolic process	7	0.5	3.30E-02	3.80E-01
lactation	7	0.5	3.30E-02	3.80E-01
salivary gland development	7	0.5	3.30E-02	3.80E-01
acetyl-CoA catabolic process	6	0.5	3.70E-02	4.20E-01
nicotinamide metabolic process	6	0.5	3.70E-02	4.20E-01
purine nucleoside metabolic process	6	0.5	3.70E-02	4.20E-01
nicotinamide nucleotide metabolic process	6	0.5	3.70E-02	4.20E-01
purine ribonucleoside metabolic process	6	0.5	3.70E-02	4.20E-01
alkaloid metabolic process	6	0.5	3.70E-02	4.20E-01
intracellular receptor-mediated signaling pathway	7	0.5	3.80E-02	4.20E-01
positive regulation of T cell differentiation	7	0.5	3.80E-02	4.20E-01
cofactor catabolic process	7	0.5	3.80E-02	4.20E-01
regulation of phagocytosis	6	0.5	4.40E-02	4.60E-01
positive regulation of lymphocyte differentiation	7	0.5	4.90E-02	4.90E-01
positive regulation of endocytosis	7	0.5	4.90E-02	4.90E-01
negative regulation of lymphocyte proliferation	7	0.5	5.50E-02	5.20E-01
regulation of nucleocytoplasmic transport	7	0.5	5.50E-02	5.20E-01
negative regulation of leukocyte proliferation	7	0.5	5.50E-02	5.20E-01
negative regulation of mononuclear cell proliferation	7	0.5	5.50E-02	5.20E-01
nuclear export	6	0.5	5.80E-02	5.30E-01
negative regulation of epithelial cell proliferation	6	0.5	5.80E-02	5.30E-01
triglyceride metabolic process	7	0.5	6.20E-02	5.50E-01
regulation of microtubule cytoskeleton organization	7	0.5	6.20E-02	5.50E-01
cellular component assembly involved in morphogenesis	7	0.5	6.20E-02	5.50E-01
response to reactive oxygen species	7	0.5	6.20E-02	5.50E-01
negative regulation of T cell proliferation	6	0.5	6.70E-02	5.60E-01

pyridine nucleotide metabolic process	6	0.5	6.70E-02	5.60E-01
antigen processing and presentation of exogenous antigen	6	0.5	6.70E-02	5.60E-01
pigment metabolic process	7	0.5	6.90E-02	5.70E-01
coenzyme catabolic process	6	0.5	7.60E-02	6.00E-01
polysaccharide biosynthetic process	6	0.5	8.50E-02	6.30E-01
regulation of transforming growth factor beta receptor signaling pathway	6	0.5	8.50E-02	6.30E-01
pigment biosynthetic process	6	0.5	8.50E-02	6.30E-01
ribonucleoside metabolic process	7	0.5	8.50E-02	6.30E-01
cellular amide metabolic process	7	0.5	8.50E-02	6.30E-01
regulation of cell-substrate adhesion	7	0.5	9.40E-02	6.50E-01
glycosaminoglycan metabolic process	7	0.5	9.40E-02	6.50E-01
establishment or maintenance of cell polarity	6	0.5	9.50E-02	6.50E-01
mannose metabolic process	5	0.4	8.30E-03	1.60E-01
cellular response to hydrogen peroxide	5	0.4	1.20E-02	2.00E-01
chaperone mediated protein folding requiring cofactor	5	0.4	1.20E-02	2.00E-01
myotube differentiation	5	0.4	1.20E-02	2.00E-01
hydrogen peroxide catabolic process	5	0.4	1.20E-02	2.00E-01
cortical cytoskeleton organization	5	0.4	1.60E-02	2.40E-01
ER-associated protein catabolic process	5	0.4	1.60E-02	2.40E-01
'de novo' protein folding	5	0.4	2.10E-02	2.90E-01
'de novo' posttranslational protein folding	5	0.4	2.10E-02	2.90E-01
actin filament-based movement	5	0.4	2.70E-02	3.40E-01
protein amino acid O-linked glycosylation	5	0.4	3.30E-02	3.90E-01
lamellipodium assembly	5	0.4	3.30E-02	3.90E-01
apoptotic nuclear changes	5	0.4	5.90E-02	5.30E-01
regulation of nitric oxide biosynthetic process	5	0.4	5.90E-02	5.30E-01
positive regulation of protein polymerization	5	0.4	5.90E-02	5.30E-01
regulation of smooth muscle cell proliferation	5	0.4	6.90E-02	5.70E-01
DNA catabolic process	5	0.4	8.00E-02	6.10E-01
regulation of steroid metabolic process	5	0.4	9.20E-02	6.50E-01
negative regulation of inflammatory response	5	0.4	9.20E-02	6.50E-01
aspartate family amino acid metabolic process	5	0.4	9.20E-02	6.50E-01
IMP biosynthetic process	4	0.3	1.90E-03	5.30E-02
IMP metabolic process	4	0.3	1.90E-03	5.30E-02
positive regulation of protein complex disassembly	4	0.3	8.30E-03	1.60E-01
pentose-phosphate shunt	4	0.3	2.10E-02	2.90E-01
nucleobase biosynthetic process	4	0.3	2.90E-02	3.60E-01
asymmetric protein localization	4	0.3	3.90E-02	4.30E-01
cellular copper ion homeostasis	4	0.3	3.90E-02	4.30E-01
copper ion homeostasis	4	0.3	5.10E-02	5.00E-01
purine base metabolic process	4	0.3	5.10E-02	5.00E-01
peptidyl-lysine modification	4	0.3	6.40E-02	5.50E-01
glycine metabolic process	4	0.3	6.40E-02	5.50E-01
membrane protein proteolysis	4	0.3	7.80E-02	6.00E-01
DNA fragmentation involved in apoptosis	4	0.3	7.80E-02	6.00E-01
membrane protein ectodomain proteolysis	4	0.3	7.80E-02	6.00E-01
positive regulation of myeloid leukocyte differentiation	4	0.3	7.80E-02	6.00E-01
heart process	4	0.3	9.40E-02	6.50E-01
cell-substrate junction assembly	4	0.3	9.40E-02	6.50E-01
vascular endothelial growth factor receptor signaling pathway	4	0.3	9.40E-02	6.50E-01
NADP metabolic process	4	0.3	9.40E-02	6.50E-01
heart contraction	4	0.3	9.40E-02	6.50E-01
'de novo' IMP biosynthetic process	3	0.2	1.80E-02	2.60E-01
myoblast proliferation	3	0.2	1.80E-02	2.60E-01
N-glycan processing	3	0.2	3.40E-02	3.90E-01
ribosomal protein import into nucleus	3	0.2	3.40E-02	3.90E-01
cellular metabolic compound salvage	3	0.2	5.40E-02	5.10E-01
purine base biosynthetic process	3	0.2	5.40E-02	5.10E-01
dichotomous subdivision of terminal units involved in salivary gland branching	3	0.2	5.40E-02	5.10E-01
skeletal myofibril assembly	3	0.2	5.40E-02	5.10E-01
ruffle organization	3	0.2	5.40E-02	5.10E-01
notochord morphogenesis	3	0.2	7.60E-02	6.00E-01
blood vessel endothelial cell migration	3	0.2	7.60E-02	6.00E-01

ribosomal small subunit biogenesis	3	0.2	7.60E-02	6.00E-01
peptidyl-lysine modification to hypusine	3	0.2	7.60E-02	6.00E-01
dichotomous subdivision of an epithelial terminal unit	3	0.2	7.60E-02	6.00E-01
neurotransmitter receptor metabolic process	3	0.2	7.60E-02	6.00E-01
hypusine metabolic process	3	0.2	7.60E-02	6.00E-01
purine deoxyribonucleoside triphosphate metabolic process	3	0.2	7.60E-02	6.00E-01
cardiac myofibril assembly	3	0.2	7.60E-02	6.00E-01
purine deoxyribonucleotide metabolic process	3	0.2	7.60E-02	6.00E-01
glycolipid catabolic process	3	0.2	7.60E-02	6.00E-01
Day12_CC				
Term	Count	%	P-Value	Benjamini
non-membrane-bounded organelle	264	18.6	1.90E-17	2.20E-15
intracellular non-membrane-bounded organelle	264	18.6	1.90E-17	2.20E-15
extracellular region	162	11.4	4.80E-02	1.90E-01
membrane-enclosed lumen	153	10.8	3.80E-08	8.50E-07
cytoskeleton	151	10.6	5.70E-09	1.40E-07
intracellular organelle lumen	144	10.1	4.70E-07	8.50E-06
organelle lumen	144	10.1	5.40E-07	9.60E-06
mitochondrion	140	9.9	3.80E-03	2.40E-02
cytosol	126	8.9	7.30E-26	1.70E-23
ribonucleoprotein complex	120	8.5	8.80E-30	4.20E-27
cytoskeletal part	105	7.4	1.30E-06	1.90E-05
vesicle	102	7.2	8.40E-16	8.50E-14
cytoplasmic vesicle	99	7	4.00E-15	3.20E-13
extracellular region part	96	6.8	1.40E-04	1.20E-03
endoplasmic reticulum	97	6.8	1.40E-03	9.30E-03
Golgi apparatus	95	6.7	1.40E-06	2.10E-05
nuclear lumen	93	6.6	2.20E-02	9.80E-02
membrane-bounded vesicle	87	6.1	6.00E-15	4.10E-13
cytoplasmic membrane-bounded vesicle	86	6.1	7.40E-15	4.40E-13
endomembrane system	85	6	1.90E-08	4.50E-07
cell projection	82	5.8	3.30E-06	4.40E-05
cell fraction	73	5.1	1.40E-03	9.20E-03
internal side of plasma membrane	61	4.3	3.60E-11	1.00E-09
insoluble fraction	59	4.2	2.60E-02	1.10E-01
extrinsic to membrane	57	4	6.50E-03	3.50E-02
vacuole	55	3.9	1.60E-14	8.60E-13
microtubule cytoskeleton	55	3.9	6.00E-03	3.40E-02
extracellular matrix	54	3.8	5.50E-07	9.40E-06
membrane fraction	54	3.8	7.30E-02	2.60E-01
extracellular space	54	3.8	7.40E-02	2.60E-01
proteinaceous extracellular matrix	52	3.7	8.70E-07	1.40E-05
actin cytoskeleton	51	3.6	4.50E-12	1.40E-10
lysosome	49	3.5	2.30E-13	9.80E-12
lytic vacuole	49	3.5	2.80E-13	1.10E-11
ribosome	48	3.4	1.70E-11	5.20E-10
nucleolus	46	3.2	2.60E-04	2.10E-03
melanosome	44	3.1	3.70E-24	5.90E-22
pigment granule	44	3.1	3.70E-24	5.90E-22
endoplasmic reticulum part	43	3	1.80E-06	2.50E-05
spliceosome	39	2.7	9.90E-13	3.60E-11
Golgi apparatus part	39	2.7	3.80E-05	3.80E-04
microtubule	36	2.5	1.10E-03	7.90E-03
neuron projection	34	2.4	5.40E-03	3.10E-02
nuclear envelope	32	2.3	4.00E-06	5.20E-05
cell cortex	31	2.2	1.50E-07	3.20E-06
endosome	30	2.1	5.70E-02	2.20E-01
proteasome complex	28	2	1.80E-14	8.50E-13
endoplasmic reticulum lumen	29	2	1.40E-12	4.90E-11
cell leading edge	29	2	1.30E-07	2.90E-06
extracellular matrix part	25	1.8	4.50E-07	8.50E-06
coated membrane	23	1.6	6.40E-10	1.70E-08

membrane coat	23	1.6	6.40E-10	1.70E-08
contractile fiber	22	1.6	3.70E-05	3.80E-04
Golgi membrane	22	1.6	5.70E-03	3.30E-02
basolateral plasma membrane	22	1.6	7.90E-03	4.10E-02
basement membrane	21	1.5	1.80E-06	2.50E-05
contractile fiber part	21	1.5	2.60E-05	2.70E-04
cell cortex part	20	1.4	7.20E-06	8.60E-05
myofibril	20	1.4	1.90E-04	1.60E-03
soluble fraction	20	1.4	5.90E-04	4.40E-03
nuclear pore	18	1.3	4.90E-06	6.20E-05
cytosolic part	18	1.3	1.30E-05	1.40E-04
pore complex	18	1.3	1.00E-04	9.60E-04
coated vesicle	19	1.3	1.70E-02	8.00E-02
apical part of cell	18	1.3	6.90E-02	2.50E-01
sarcomere	17	1.2	9.80E-04	7.10E-03
cytoplasmic vesicle part	17	1.2	6.00E-03	3.40E-02
vesicle membrane	17	1.2	7.40E-03	3.80E-02
ribosomal subunit	16	1.1	3.40E-04	2.70E-03
lamellipodium	15	1.1	6.70E-04	5.00E-03
cytoplasmic vesicle membrane	16	1.1	6.20E-03	3.40E-02
adherens junction	15	1.1	6.40E-02	2.40E-01
axon	15	1.1	6.80E-02	2.50E-01
cell soma	16	1.1	7.00E-02	2.50E-01
ruffle	14	1	1.00E-04	9.50E-04
coated pit	13	0.9	6.00E-06	7.40E-05
proton-transporting two-sector ATPase complex	13	0.9	1.30E-04	1.20E-03
cortical cytoskeleton	13	0.9	1.70E-04	1.50E-03
coated vesicle membrane	13	0.9	1.30E-03	9.40E-03
extrinsic to plasma membrane	13	0.9	1.90E-03	1.30E-02
focal adhesion	13	0.9	2.60E-03	1.70E-02
cell-substrate adherens junction	13	0.9	4.70E-03	2.80E-02
cell-substrate junction	13	0.9	8.90E-03	4.50E-02
proton-transporting two-sector ATPase complex, catalytic domain	11	0.8	2.10E-07	4.20E-06
stress fiber	11	0.8	7.70E-06	8.90E-05
vesicle coat	12	0.8	8.70E-06	9.90E-05
actin filament bundle	11	0.8	1.20E-05	1.30E-04
actomyosin	11	0.8	4.10E-05	4.10E-04
clathrin coat	12	0.8	7.20E-05	7.00E-04
Golgi-associated vesicle	12	0.8	1.30E-04	1.20E-03
I band	12	0.8	4.30E-03	2.70E-02
Z disc	11	0.8	4.50E-03	2.80E-02
nuclear periphery	11	0.8	7.20E-03	3.80E-02
myosin complex	11	0.8	3.20E-02	1.30E-01
small ribosomal subunit	10	0.7	4.70E-04	3.60E-03
growth cone	10	0.7	2.60E-02	1.10E-01
site of polarized growth	10	0.7	2.60E-02	1.10E-01
eukaryotic translation initiation factor 3 complex	9	0.6	9.60E-07	1.50E-05
proton-transporting V-type ATPase complex	8	0.6	4.30E-04	3.40E-03
cytosolic ribosome	8	0.6	4.30E-04	3.40E-03
Golgi-associated vesicle membrane	8	0.6	1.30E-03	8.90E-03
transport vesicle	9	0.6	2.20E-03	1.50E-02
actin filament	9	0.6	2.80E-03	1.80E-02
organelle envelope lumen	9	0.6	1.80E-02	8.10E-02
nuclear matrix	8	0.6	6.20E-02	2.30E-01
late endosome	8	0.6	7.60E-02	2.60E-01
chaperonin-containing T-complex	7	0.5	2.40E-06	3.30E-05
clathrin adaptor complex	7	0.5	1.60E-02	7.70E-02
AP-type membrane coat adaptor complex	7	0.5	1.60E-02	7.70E-02
mitochondrial intermembrane space	7	0.5	3.80E-02	1.50E-01
vacuolar part	7	0.5	7.20E-02	2.50E-01
nuclear membrane	7	0.5	9.90E-02	3.20E-01
proton-transporting V-type ATPase, V1 domain	6	0.4	2.00E-04	1.60E-03
proteasome accessory complex	5	0.4	2.40E-04	2.00E-03

septin cytoskeleton	6	0.4	4.60E-03	2.80E-02
septin complex	6	0.4	4.60E-03	2.80E-02
filamentous actin	6	0.4	6.40E-03	3.50E-02
ciliary rootlet	5	0.4	7.20E-03	3.80E-02
COPI vesicle coat	5	0.4	7.20E-03	3.80E-02
COPI coated vesicle membrane	5	0.4	7.20E-03	3.80E-02
transport vesicle membrane	6	0.4	8.60E-03	4.40E-02
trans-Golgi network transport vesicle	6	0.4	1.50E-02	7.20E-02
COPI-coated vesicle	5	0.4	1.50E-02	7.10E-02
collagen	6	0.4	1.90E-02	8.50E-02
basal lamina	5	0.4	2.00E-02	9.00E-02
neuromuscular junction	6	0.4	3.40E-02	1.40E-01
filopodium	6	0.4	3.40E-02	1.40E-01
uropod	4	0.3	5.40E-03	3.20E-02
trailing edge	4	0.3	5.40E-03	3.20E-02
clathrin coat of coated pit	4	0.3	1.00E-02	5.00E-02
Arp2/3 protein complex	4	0.3	1.00E-02	5.00E-02
cytosolic large ribosomal subunit	4	0.3	1.60E-02	7.80E-02
nuclear lamina	4	0.3	2.50E-02	1.10E-01
signalosome	4	0.3	4.70E-02	1.80E-01
clathrin vesicle coat	4	0.3	6.00E-02	2.30E-01
kinesin complex	4	0.3	7.50E-02	2.60E-01
immunological synapse	4	0.3	9.20E-02	3.00E-01
proteasome activator complex	3	0.2	2.00E-02	9.10E-02
aminoacyl-tRNA synthetase multienzyme complex	3	0.2	2.00E-02	9.10E-02
eukaryotic translation elongation factor 1 complex	3	0.2	3.90E-02	1.60E-01
lamin filament	3	0.2	3.90E-02	1.60E-01
DNA replication factor A complex	3	0.2	6.10E-02	2.30E-01
vacuolar lumen	3	0.2	6.10E-02	2.30E-01
chromocenter	3	0.2	8.60E-02	2.90E-01

Day12_MF

Term	Count	%	P-Value	Benjamini
nucleotide binding	323	22.8	5.10E-26	5.20E-23
purine nucleotide binding	263	18.5	1.20E-17	3.20E-15
ribonucleotide binding	255	18	1.30E-17	2.70E-15
purine ribonucleotide binding	255	18	1.30E-17	2.70E-15
adenyl nucleotide binding	198	14	1.70E-09	1.40E-07
purine nucleoside binding	198	14	3.60E-09	2.30E-07
nucleoside binding	198	14	5.90E-09	3.40E-07
adenyl ribonucleotide binding	190	13.4	2.00E-09	1.50E-07
ATP binding	188	13.2	2.30E-09	1.60E-07
RNA binding	127	8.9	7.60E-18	2.60E-15
structural molecule activity	98	6.9	4.90E-18	2.50E-15
calcium ion binding	98	6.9	1.60E-03	3.40E-02
GTP binding	73	5.1	3.10E-12	4.50E-10
guanyl nucleotide binding	73	5.1	1.10E-11	1.10E-09
guanyl ribonucleotide binding	73	5.1	1.10E-11	1.10E-09
cytoskeletal protein binding	72	5.1	1.20E-08	6.30E-07
peptidase activity	72	5.1	1.00E-02	1.50E-01
peptidase activity, acting on L-amino acid peptides	69	4.9	1.20E-02	1.70E-01
actin binding	53	3.7	2.10E-07	9.70E-06
endopeptidase activity	51	3.6	1.20E-02	1.70E-01
ATPase activity	48	3.4	7.10E-06	2.90E-04
magnesium ion binding	47	3.3	3.60E-02	3.70E-01
structural constituent of ribosome	46	3.2	4.20E-14	7.20E-12
ATPase activity, coupled	44	3.1	3.70E-07	1.70E-05
carbohydrate binding	41	2.9	9.10E-03	1.40E-01
GTPase activity	38	2.7	2.10E-11	2.00E-09
cofactor binding	34	2.4	1.80E-03	4.00E-02
translation factor activity, nucleic acid binding	31	2.2	3.60E-10	3.10E-08
ligase activity, forming carbon-nitrogen bonds	30	2.1	2.20E-03	4.50E-02
unfolded protein binding	28	2	6.30E-12	8.20E-10
coenzyme binding	29	2	2.20E-04	6.10E-03

pattern binding	27	1.9	2.90E-05	1.10E-03
polysaccharide binding	27	1.9	2.90E-05	1.10E-03
glycosaminoglycan binding	25	1.8	3.10E-05	1.10E-03
metallopeptidase activity	25	1.8	3.10E-02	3.30E-01
manganese ion binding	24	1.7	3.90E-03	7.40E-02
protein domain specific binding	24	1.7	6.60E-02	5.20E-01
ligase activity, forming carbon-oxygen bonds	23	1.6	9.40E-12	1.10E-09
ligase activity, forming aminoacyl-tRNA and related compounds	23	1.6	9.40E-12	1.10E-09
aminoacyl-tRNA ligase activity	23	1.6	9.40E-12	1.10E-09
translation initiation factor activity	23	1.6	3.60E-09	2.20E-07
protein transporter activity	22	1.6	7.40E-08	3.60E-06
helicase activity	21	1.5	7.00E-03	1.20E-01
acid-amino acid ligase activity	21	1.5	7.80E-02	5.80E-01
motor activity	20	1.4	2.70E-02	3.00E-01
ATPase activity, coupled to transmembrane movement of ions	19	1.3	1.50E-05	5.80E-04
growth factor binding	18	1.3	9.90E-05	3.20E-03
heparin binding	19	1.3	2.00E-04	5.60E-03
purine NTP-dependent helicase activity	18	1.3	9.20E-04	2.20E-02
ATP-dependent helicase activity	18	1.3	9.20E-04	2.20E-02
ATPase activity, coupled to transmembrane movement of substances	19	1.3	1.40E-03	3.30E-02
hydrolase activity, acting on acid anhydrides, catalyzing transmembrane movement of substanc	19	1.3	1.40E-03	3.30E-02
ATPase activity, coupled to movement of substances	19	1.3	1.40E-03	3.30E-02
P-P-bond-hydrolysis-driven transmembrane transporter activity	19	1.3	4.30E-03	8.00E-02
primary active transmembrane transporter activity	19	1.3	4.80E-03	8.60E-02
monovalent inorganic cation transmembrane transporter activity	17	1.2	2.80E-03	5.40E-02
inorganic cation transmembrane transporter activity	17	1.2	7.90E-02	5.80E-01
transferase activity, transferring alkyl or aryl (other than methyl) groups	16	1.1	2.60E-05	1.00E-03
exopeptidase activity	15	1.1	2.30E-03	4.60E-02
tubulin binding	14	1	1.20E-02	1.70E-01
hydrogen ion transmembrane transporter activity	14	1	2.20E-02	2.60E-01
NAD or NADH binding	13	0.9	2.50E-04	6.50E-03
actin filament binding	13	0.9	7.20E-04	1.80E-02
proton-transporting ATPase activity, rotational mechanism	11	0.8	4.30E-08	2.20E-06
cation-transporting ATPase activity	12	0.8	2.60E-06	1.10E-04
ribonucleoprotein binding	12	0.8	8.20E-05	2.80E-03
structural constituent of cytoskeleton	11	0.8	9.40E-05	3.10E-03
intramolecular oxidoreductase activity	12	0.8	2.50E-04	6.40E-03
microtubule binding	12	0.8	1.40E-02	1.90E-01
cysteine-type endopeptidase activity	12	0.8	1.80E-02	2.20E-01
rRNA binding	10	0.7	1.00E-04	3.10E-03
extracellular matrix binding	10	0.7	1.40E-04	4.30E-03
mRNA binding	10	0.7	4.00E-02	3.80E-01
ATPase activity, coupled to transmembrane movement of ions, phosphorylative mechanism	10	0.7	5.30E-02	4.70E-01
copper ion binding	10	0.7	7.60E-02	5.70E-01
tRNA binding	9	0.6	1.40E-04	4.10E-03
glutathione transferase activity	9	0.6	1.10E-03	2.70E-02
aminopeptidase activity	8	0.6	8.10E-03	1.30E-01
translation elongation factor activity	8	0.6	1.40E-02	1.90E-01
antioxidant activity	9	0.6	1.90E-02	2.30E-01
hydrogen ion transporting ATP synthase activity, rotational mechanism	7	0.5	1.30E-04	3.90E-03
ribosome binding	7	0.5	2.20E-03	4.60E-02
insulin-like growth factor binding	7	0.5	9.00E-03	1.40E-01
intramolecular transferase activity	7	0.5	1.40E-02	1.90E-01
integrin binding	7	0.5	1.70E-02	2.20E-01
carboxy-lyase activity	7	0.5	2.50E-02	2.80E-01
extracellular matrix structural constituent	7	0.5	4.00E-02	3.80E-01
oxidoreductase activity, acting on sulfur group of donors	7	0.5	6.00E-02	5.00E-01
peroxiredoxin activity	5	0.4	1.50E-03	3.50E-02
intramolecular oxidoreductase activity, interconverting keto- and enol-groups	5	0.4	4.80E-03	8.60E-02
intramolecular oxidoreductase activity, interconverting aldoses and ketoses	5	0.4	4.80E-03	8.60E-02
platelet-derived growth factor binding	5	0.4	4.80E-03	8.60E-02
mannosidase activity	6	0.4	6.80E-03	1.10E-01
intramolecular transferase activity, phosphotransferases	5	0.4	7.50E-03	1.20E-01

ubiquitin protein ligase binding	5	0.4	2.70E-02	3.00E-01
hydrogen-exporting ATPase activity, phosphorylative mechanism	5	0.4	2.70E-02	3.00E-01
palmitoyl-CoA hydrolase activity	5	0.4	3.50E-02	3.60E-01
oxidoreductase activity, acting on the CH-NH group of donors, NAD or NADP as acceptor	5	0.4	3.50E-02	3.60E-01
oxidoreductase activity, acting on the CH-NH group of donors	6	0.4	5.90E-02	5.00E-01
phosphoprotein binding	5	0.4	6.30E-02	5.10E-01
acyl-CoA thioesterase activity	5	0.4	8.70E-02	6.10E-01
oxidoreductase activity, acting on peroxide as acceptor	6	0.4	8.80E-02	6.10E-01
peroxidase activity	6	0.4	8.80E-02	6.10E-01
ribose phosphate diphosphokinase activity	4	0.3	2.40E-03	4.70E-02
diphosphotransferase activity	4	0.3	5.60E-03	9.60E-02
actin-dependent ATPase activity	4	0.3	1.00E-02	1.50E-01
intramolecular oxidoreductase activity, transposing S-S bonds	4	0.3	2.60E-02	2.90E-01
protein disulfide isomerase activity	4	0.3	2.60E-02	2.90E-01
alpha-mannosidase activity	4	0.3	2.60E-02	2.90E-01
carbon-nitrogen ligase activity, with glutamine as amido-N-donor	4	0.3	3.60E-02	3.70E-01
sodium:potassium-exchanging ATPase activity	4	0.3	4.80E-02	4.40E-01
telomeric DNA binding	4	0.3	6.20E-02	5.10E-01
protein kinase C binding	4	0.3	6.20E-02	5.10E-01
sulfuric ester hydrolase activity	4	0.3	9.50E-02	6.40E-01
phosphoglucomutase activity	3	0.2	2.10E-02	2.50E-01
procollagen-lysine 5-dioxygenase activity	3	0.2	2.10E-02	2.50E-01
proteasome activator activity	3	0.2	4.00E-02	3.90E-01
peptidyl-lysine 5-dioxygenase activity	3	0.2	4.00E-02	3.90E-01
ADP binding	3	0.2	4.00E-02	3.90E-01
proteasome regulator activity	3	0.2	4.00E-02	3.90E-01
serine-type carboxypeptidase activity	3	0.2	6.20E-02	5.10E-01
GDP-dissociation inhibitor activity	3	0.2	6.20E-02	5.10E-01
serine-type exopeptidase activity	3	0.2	6.20E-02	5.10E-01
isocitrate dehydrogenase activity	3	0.2	8.80E-02	6.10E-01
transferase activity, transferring acyl groups, acyl groups converted into alkyl on transfer	3	0.2	8.80E-02	6.10E-01
profilin binding	3	0.2	8.80E-02	6.10E-01
oxidoreductase activity, acting on sulfur group of donors, NAD or NADP as acceptor	3	0.2	8.80E-02	6.10E-01
Day12_BP				
Term	Count	%	P-Value	Benjamini
protein localization	135	9.5	1.70E-17	2.60E-14
establishment of protein localization	119	8.4	1.20E-15	1.20E-12
protein transport	118	8.3	1.70E-15	1.30E-12
proteolysis	109	7.7	1.30E-02	2.00E-01
translation	103	7.3	4.40E-34	1.40E-30
intracellular transport	88	6.2	1.00E-14	6.10E-12
vesicle-mediated transport	76	5.4	3.90E-08	5.10E-06
homeostatic process	76	5.4	1.70E-04	7.10E-03
RNA processing	73	5.1	2.70E-08	4.00E-06
oxidation reduction	73	5.1	2.30E-02	3.10E-01
cell adhesion	71	5	6.40E-04	2.10E-02
biological adhesion	71	5	6.70E-04	2.20E-02
macromolecule catabolic process	70	4.9	3.30E-02	3.90E-01
regulation of apoptosis	64	4.5	9.50E-03	1.80E-01
regulation of programmed cell death	64	4.5	1.20E-02	2.00E-01
regulation of cell death	64	4.5	1.40E-02	2.10E-01
cellular protein localization	62	4.4	7.00E-11	2.10E-08
cellular macromolecule localization	62	4.4	9.30E-11	2.60E-08
mRNA metabolic process	62	4.4	1.10E-10	2.80E-08
cellular macromolecule catabolic process	62	4.4	9.70E-02	6.80E-01
intracellular protein transport	59	4.2	6.00E-11	2.00E-08
macromolecular complex subunit organization	60	4.2	1.20E-06	1.10E-04
macromolecular complex assembly	58	4.1	3.60E-07	3.80E-05
mRNA processing	56	3.9	2.00E-10	4.30E-08
nitrogen compound biosynthetic process	56	3.9	4.20E-08	5.30E-06
cytoskeleton organization	55	3.9	1.30E-06	1.20E-04
cellular homeostasis	53	3.7	2.70E-05	1.40E-03
RNA splicing	49	3.5	2.70E-11	1.20E-08

cellular macromolecular complex subunit organization	49	3.5	2.90E-08	4.10E-06
cellular macromolecular complex assembly	47	3.3	4.40E-09	7.90E-07
small GTPase mediated signal transduction	47	3.3	9.80E-07	9.70E-05
cell motion	45	3.2	1.20E-02	2.00E-01
actin filament-based process	44	3.1	1.30E-10	3.10E-08
generation of precursor metabolites and energy	44	3.1	1.70E-05	1.00E-03
nucleobase, nucleoside and nucleotide biosynthetic process	42	3	2.80E-09	5.80E-07
nucleobase, nucleoside, nucleotide and nucleic acid biosynthetic process	42	3	2.80E-09	5.80E-07
protein complex assembly	42	3	2.70E-06	2.00E-04
protein complex biogenesis	42	3	2.70E-06	2.00E-04
cellular response to stress	43	3	9.80E-02	6.80E-01
nucleotide biosynthetic process	40	2.8	1.30E-08	2.10E-06
membrane organization	40	2.8	8.00E-04	2.60E-02
actin cytoskeleton organization	39	2.7	9.10E-09	1.50E-06
cellular component morphogenesis	39	2.7	7.10E-02	6.00E-01
protein folding	37	2.6	4.70E-11	1.80E-08
cofactor metabolic process	37	2.6	1.20E-06	1.10E-04
localization of cell	37	2.6	9.60E-03	1.80E-01
cell motility	37	2.6	9.60E-03	1.80E-01
monosaccharide metabolic process	36	2.5	1.00E-05	6.50E-04
ncRNA metabolic process	36	2.5	3.50E-05	1.80E-03
cell proliferation	36	2.5	1.80E-03	5.00E-02
regulation of phosphorylation	35	2.5	3.40E-02	3.90E-01
regulation of phosphate metabolic process	36	2.5	3.50E-02	4.00E-01
regulation of phosphorus metabolic process	36	2.5	3.50E-02	4.00E-01
cell projection organization	36	2.5	7.00E-02	6.00E-01
negative regulation of apoptosis	34	2.4	3.60E-03	8.20E-02
negative regulation of programmed cell death	34	2.4	5.00E-03	1.00E-01
negative regulation of cell death	34	2.4	5.30E-03	1.10E-01
positive regulation of molecular function	34	2.4	9.10E-02	6.70E-01
purine nucleotide biosynthetic process	32	2.3	3.20E-07	3.50E-05
purine nucleotide metabolic process	32	2.3	1.00E-05	6.70E-04
hexose metabolic process	33	2.3	1.20E-05	7.60E-04
cell migration	32	2.3	1.20E-02	2.00E-01
ion homeostasis	33	2.3	8.40E-02	6.40E-01
cellular protein complex assembly	31	2.2	3.30E-09	6.30E-07
ribonucleotide metabolic process	31	2.2	1.20E-07	1.50E-05
ribonucleotide biosynthetic process	30	2.1	2.70E-08	4.20E-06
positive regulation of catalytic activity	30	2.1	8.00E-02	6.30E-01
cellular ion homeostasis	30	2.1	8.00E-02	6.30E-01
in utero embryonic development	30	2.1	1.00E-01	6.80E-01
purine ribonucleotide biosynthetic process	28	2	1.80E-07	2.00E-05
purine ribonucleotide metabolic process	28	2	1.70E-06	1.50E-04
protein targeting	29	2	5.30E-06	3.70E-04
coenzyme metabolic process	29	2	2.20E-05	1.20E-03
regulation of organelle organization	29	2	8.80E-05	4.20E-03
muscle organ development	29	2	8.60E-04	2.70E-02
membrane invagination	29	2	2.40E-03	6.00E-02
endocytosis	29	2	2.40E-03	6.00E-02
regulation of kinase activity	29	2	3.20E-03	7.60E-02
regulation of transferase activity	29	2	5.40E-03	1.10E-01
tissue morphogenesis	28	2	7.30E-02	6.10E-01
tRNA metabolic process	27	1.9	2.00E-06	1.60E-04
regulation of protein kinase activity	27	1.9	7.70E-03	1.50E-01
regulation of cytoskeleton organization	25	1.8	1.80E-06	1.60E-04
posttranscriptional regulation of gene expression	25	1.8	1.50E-03	4.40E-02
extracellular structure organization	25	1.8	1.60E-03	4.70E-02
regulation of cellular component size	26	1.8	2.20E-03	5.70E-02
microtubule-based process	26	1.8	5.50E-02	5.30E-01
heart development	26	1.8	9.10E-02	6.70E-01
positive regulation of developmental process	25	1.8	9.60E-02	6.80E-01
cation homeostasis	24	1.7	3.80E-02	4.20E-01
gland development	24	1.7	7.20E-02	6.00E-01

amino acid activation	23	1.6	2.40E-12	1.20E-09
tRNA aminoacylation	23	1.6	2.40E-12	1.20E-09
tRNA aminoacylation for protein translation	23	1.6	2.40E-12	1.20E-09
nucleocytoplasmic transport	23	1.6	1.30E-05	7.90E-04
nuclear transport	23	1.6	1.80E-05	1.00E-03
positive regulation of cellular component organization	23	1.6	5.50E-04	1.90E-02
glucose metabolic process	23	1.6	3.40E-03	8.00E-02
positive regulation of cell differentiation	22	1.6	6.60E-02	5.80E-01
cell redox homeostasis	21	1.5	9.10E-08	1.10E-05
purine nucleoside triphosphate metabolic process	21	1.5	5.30E-04	1.80E-02
nucleoside triphosphate metabolic process	21	1.5	1.50E-03	4.40E-02
muscle cell differentiation	21	1.5	1.90E-03	5.20E-02
muscle tissue development	21	1.5	1.10E-02	1.80E-01
glycoprotein metabolic process	21	1.5	2.30E-02	3.10E-01
cellular cation homeostasis	21	1.5	3.20E-02	3.90E-01
carbohydrate catabolic process	20	1.4	3.50E-05	1.80E-03
regulation of cellular component biogenesis	20	1.4	1.40E-04	5.70E-03
purine nucleoside triphosphate biosynthetic process	20	1.4	2.90E-04	1.10E-02
sulfur metabolic process	20	1.4	2.90E-04	1.10E-02
nucleoside triphosphate biosynthetic process	20	1.4	3.30E-04	1.30E-02
protein localization in organelle	20	1.4	4.40E-04	1.60E-02
striated muscle tissue development	20	1.4	1.10E-02	1.80E-01
positive regulation of kinase activity	20	1.4	1.30E-02	2.10E-01
positive regulation of transferase activity	20	1.4	1.90E-02	2.80E-01
actin filament organization	18	1.3	2.20E-06	1.70E-04
regulation of actin cytoskeleton organization	18	1.3	6.20E-06	4.30E-04
regulation of actin filament-based process	18	1.3	7.90E-06	5.40E-04
regulation of protein complex assembly	18	1.3	2.00E-05	1.10E-03
striated muscle cell differentiation	19	1.3	4.10E-04	1.50E-02
protein import	18	1.3	4.40E-04	1.60E-02
ribonucleoside triphosphate biosynthetic process	19	1.3	7.20E-04	2.30E-02
purine ribonucleoside triphosphate biosynthetic process	19	1.3	7.20E-04	2.30E-02
extracellular matrix organization	19	1.3	1.90E-03	5.30E-02
purine ribonucleoside triphosphate metabolic process	19	1.3	1.90E-03	5.30E-02
ribonucleoside triphosphate metabolic process	19	1.3	2.20E-03	5.70E-02
regulation of cell adhesion	18	1.3	2.20E-03	5.70E-02
microtubule-based movement	18	1.3	4.70E-03	1.00E-01
positive regulation of protein kinase activity	18	1.3	3.30E-02	3.90E-01
ribonucleoprotein complex biogenesis	19	1.3	4.10E-02	4.40E-01
ubiquitin-dependent protein catabolic process	18	1.3	8.80E-02	6.60E-01
regulation of actin polymerization or depolymerization	17	1.2	2.00E-06	1.60E-04
regulation of actin filament length	17	1.2	2.70E-06	2.00E-04
regulation of protein polymerization	17	1.2	1.30E-05	7.90E-04
nuclear import	17	1.2	1.70E-05	9.90E-04
nucleic acid transport	17	1.2	9.40E-05	4.30E-03
establishment of RNA localization	17	1.2	9.40E-05	4.30E-03
RNA transport	17	1.2	9.40E-05	4.30E-03
RNA localization	17	1.2	1.10E-04	5.00E-03
nucleobase, nucleoside, nucleotide and nucleic acid transport	17	1.2	3.90E-04	1.40E-02
ATP biosynthetic process	17	1.2	1.30E-03	3.80E-02
anti-apoptosis	17	1.2	2.70E-03	6.70E-02
ATP metabolic process	17	1.2	3.40E-03	7.90E-02
negative regulation of cellular component organization	17	1.2	4.80E-03	1.00E-01
regulation of translation	17	1.2	9.70E-03	1.80E-01
regulation of cell motion	17	1.2	1.80E-02	2.60E-01
ribosome biogenesis	17	1.2	2.70E-02	3.40E-01
angiogenesis	17	1.2	9.70E-02	6.80E-01
cellular carbohydrate catabolic process	16	1.1	1.10E-04	4.80E-03
monosaccharide catabolic process	15	1.1	1.20E-04	5.10E-03
protein import into nucleus	15	1.1	1.80E-04	7.20E-03
protein localization in nucleus	15	1.1	4.70E-04	1.60E-02
alcohol catabolic process	15	1.1	9.20E-04	2.90E-02
response to oxidative stress	16	1.1	6.10E-03	1.20E-01

regulation of vesicle-mediated transport	15	1.1	9.60E-03	1.80E-01
regulation of protein localization	15	1.1	1.90E-02	2.70E-01
response to inorganic substance	15	1.1	2.30E-02	3.10E-01
polysaccharide metabolic process	15	1.1	2.30E-02	3.10E-01
regulation of cell morphogenesis	15	1.1	3.40E-02	3.90E-01
energy derivation by oxidation of organic compounds	15	1.1	3.60E-02	4.10E-01
chromatin assembly or disassembly	16	1.1	4.20E-02	4.40E-01
regulation of protein complex disassembly	14	1	3.60E-05	1.80E-03
regulation of actin filament polymerization	14	1	6.10E-05	2.90E-03
glucose catabolic process	14	1	3.00E-04	1.20E-02
hexose catabolic process	14	1	3.00E-04	1.20E-02
proton transport	14	1	3.00E-04	1.20E-02
hydrogen transport	14	1	3.70E-04	1.40E-02
mRNA transport	14	1	1.80E-03	5.00E-02
DNA packaging	14	1	8.50E-02	6.40E-01
translational initiation	13	0.9	4.40E-05	2.20E-03
ion transmembrane transport	13	0.9	1.30E-04	5.50E-03
oxidative phosphorylation	13	0.9	2.20E-03	5.70E-02
nucleoside metabolic process	13	0.9	2.20E-03	5.70E-02
muscle system process	13	0.9	9.90E-03	1.80E-01
cell projection assembly	13	0.9	1.40E-02	2.20E-01
negative regulation of immune system process	13	0.9	2.60E-02	3.30E-01
carbohydrate biosynthetic process	13	0.9	4.70E-02	4.70E-01
response to protein stimulus	13	0.9	6.30E-02	5.70E-01
response to drug	13	0.9	6.70E-02	5.90E-01
ATP synthesis coupled proton transport	12	0.8	1.70E-04	6.90E-03
energy coupled proton transport, down electrochemical gradient	12	0.8	1.70E-04	6.90E-03
protein polymerization	12	0.8	3.60E-04	1.40E-02
nucleoside monophosphate biosynthetic process	12	0.8	1.10E-03	3.30E-02
negative regulation of cytoskeleton organization	12	0.8	1.90E-03	5.20E-02
positive regulation of cell adhesion	11	0.8	2.70E-03	6.60E-02
glycolysis	11	0.8	3.20E-03	7.50E-02
regulation of endocytosis	12	0.8	3.70E-03	8.30E-02
positive regulation of organelle organization	12	0.8	3.70E-03	8.30E-02
striated muscle cell development	12	0.8	5.00E-03	1.00E-01
activation of MAPK activity	11	0.8	8.30E-03	1.60E-01
nucleoside monophosphate metabolic process	12	0.8	9.90E-03	1.80E-01
Golgi vesicle transport	11	0.8	1.10E-02	1.90E-01
muscle contraction	12	0.8	1.10E-02	1.90E-01
muscle cell development	12	0.8	1.30E-02	2.00E-01
negative regulation of organelle organization	12	0.8	1.40E-02	2.20E-01
Ras protein signal transduction	11	0.8	2.50E-02	3.30E-01
positive regulation of MAP kinase activity	11	0.8	2.50E-02	3.30E-01
gastrulation	12	0.8	4.70E-02	4.70E-01
skeletal muscle tissue development	11	0.8	8.10E-02	6.40E-01
nucleosome assembly	11	0.8	8.80E-02	6.60E-01
anatomical structure homeostasis	12	0.8	9.10E-02	6.70E-01
skeletal muscle organ development	11	0.8	9.40E-02	6.70E-01
regulation of MAP kinase activity	12	0.8	9.70E-02	6.80E-01
protein import into nucleus, docking	10	0.7	6.90E-07	7.00E-05
ribonucleoside monophosphate biosynthetic process	10	0.7	2.90E-06	2.10E-04
ribonucleoside monophosphate metabolic process	10	0.7	9.30E-06	6.10E-04
regulation of actin filament depolymerization	10	0.7	8.90E-05	4.20E-03
neuromuscular process controlling balance	10	0.7	1.60E-03	4.70E-02
vacuole organization	10	0.7	1.60E-03	4.70E-02
negative regulation of protein complex disassembly	10	0.7	2.00E-03	5.50E-02
pigment metabolic process	10	0.7	3.10E-03	7.40E-02
peptide metabolic process	10	0.7	3.80E-03	8.40E-02
glutamine family amino acid metabolic process	10	0.7	8.90E-03	1.70E-01
regulation of cell shape	10	0.7	1.60E-02	2.40E-01
regulation of catabolic process	10	0.7	2.30E-02	3.10E-01
negative regulation of cell activation	10	0.7	3.30E-02	3.90E-01
negative regulation of leukocyte activation	10	0.7	3.30E-02	3.90E-01

cellular carbohydrate biosynthetic process	10	0.7	4.50E-02	4.60E-01
cellular respiration	10	0.7	5.90E-02	5.50E-01
response to steroid hormone stimulus	10	0.7	6.50E-02	5.70E-01
neuromuscular process	10	0.7	6.50E-02	5.70E-01
adult locomotory behavior	10	0.7	7.60E-02	6.20E-01
regulation of epithelial cell proliferation	10	0.7	8.30E-02	6.40E-01
purine nucleoside monophosphate biosynthetic process	8	0.6	3.40E-05	1.80E-03
purine ribonucleoside monophosphate biosynthetic process	8	0.6	3.40E-05	1.80E-03
purine nucleoside monophosphate metabolic process	8	0.6	1.10E-04	4.80E-03
purine ribonucleoside monophosphate metabolic process	8	0.6	1.10E-04	4.80E-03
hydrogen peroxide metabolic process	9	0.6	1.30E-04	5.60E-03
glutamine metabolic process	8	0.6	6.30E-04	2.10E-02
negative regulation of actin filament depolymerization	8	0.6	1.20E-03	3.80E-02
negative regulation of protein polymerization	9	0.6	1.30E-03	4.00E-02
negative regulation of protein complex assembly	9	0.6	1.30E-03	4.00E-02
proteasomal protein catabolic process	9	0.6	2.80E-03	6.80E-02
proteasomal ubiquitin-dependent protein catabolic process	9	0.6	2.80E-03	6.80E-02
response to hydrogen peroxide	8	0.6	3.00E-03	7.10E-02
negative regulation of actin filament polymerization	8	0.6	3.80E-03	8.40E-02
regulation of protein catabolic process	9	0.6	4.30E-03	9.50E-02
positive regulation of cytoskeleton organization	8	0.6	4.80E-03	1.00E-01
cytoskeleton-dependent intracellular transport	9	0.6	5.30E-03	1.10E-01
aerobic respiration	8	0.6	6.10E-03	1.20E-01
pigment biosynthetic process	8	0.6	1.10E-02	1.90E-01
acetyl-CoA metabolic process	8	0.6	1.30E-02	2.10E-01
cofactor catabolic process	8	0.6	1.60E-02	2.40E-01
regulation of intracellular transport	9	0.6	2.30E-02	3.10E-01
response to reactive oxygen species	8	0.6	2.90E-02	3.60E-01
cellular amino acid biosynthetic process	9	0.6	3.00E-02	3.70E-01
nucleus organization	9	0.6	3.80E-02	4.20E-01
muscle fiber development	8	0.6	3.80E-02	4.20E-01
oxygen and reactive oxygen species metabolic process	9	0.6	4.20E-02	4.50E-01
cellular component disassembly	8	0.6	4.30E-02	4.50E-01
cellular amide metabolic process	8	0.6	4.30E-02	4.50E-01
regulation of heart contraction	9	0.6	5.80E-02	5.40E-01
aminoglycan metabolic process	9	0.6	7.00E-02	6.00E-01
negative regulation of lymphocyte activation	9	0.6	7.00E-02	6.00E-01
blastocyst development	9	0.6	8.30E-02	6.40E-01
actin filament bundle formation	7	0.5	9.40E-04	2.90E-02
cellular response to reactive oxygen species	7	0.5	2.80E-03	6.90E-02
nucleobase metabolic process	7	0.5	3.80E-03	8.50E-02
actin filament capping	7	0.5	3.80E-03	8.50E-02
oligosaccharide metabolic process	7	0.5	5.00E-03	1.00E-01
steroid hormone receptor signaling pathway	7	0.5	6.50E-03	1.30E-01
lysosome organization	7	0.5	8.40E-03	1.60E-01
cellular response to oxidative stress	7	0.5	8.40E-03	1.60E-01
tricarboxylic acid cycle	7	0.5	1.10E-02	1.80E-01
nicotinamide nucleotide metabolic process	7	0.5	1.30E-02	2.10E-01
nicotinamide metabolic process	7	0.5	1.30E-02	2.10E-01
acetyl-CoA catabolic process	7	0.5	1.30E-02	2.10E-01
glutathione metabolic process	7	0.5	1.30E-02	2.10E-01
alkaloid metabolic process	7	0.5	1.30E-02	2.10E-01
actomyosin structure organization	7	0.5	1.60E-02	2.40E-01
positive regulation of cell-substrate adhesion	7	0.5	2.30E-02	3.10E-01
cytokinesis	7	0.5	2.30E-02	3.10E-01
salivary gland morphogenesis	7	0.5	2.30E-02	3.10E-01
pyridine nucleotide metabolic process	7	0.5	2.70E-02	3.50E-01
coenzyme catabolic process	7	0.5	3.20E-02	3.90E-01
regulation of intracellular protein transport	7	0.5	3.70E-02	4.20E-01
lactation	7	0.5	4.30E-02	4.50E-01
salivary gland development	7	0.5	4.30E-02	4.50E-01
positive regulation of T cell differentiation	7	0.5	4.90E-02	4.90E-01
intracellular receptor-mediated signaling pathway	7	0.5	4.90E-02	4.90E-01

positive regulation of endocytosis	7	0.5	6.40E-02	5.70E-01
positive regulation of lymphocyte differentiation	7	0.5	6.40E-02	5.70E-01
regulation of nucleocytoplasmic transport	7	0.5	7.10E-02	6.00E-01
regulation of microtubule cytoskeleton organization	7	0.5	8.00E-02	6.30E-01
triglyceride metabolic process	7	0.5	8.00E-02	6.30E-01
mesoderm formation	7	0.5	8.00E-02	6.30E-01
cellular component assembly involved in morphogenesis	7	0.5	8.00E-02	6.30E-01
oxidoreduction coenzyme metabolic process	7	0.5	9.80E-02	6.80E-01
mesoderm morphogenesis	7	0.5	9.80E-02	6.80E-01
myotube differentiation	6	0.4	2.10E-03	5.50E-02
cellular response to hydrogen peroxide	6	0.4	2.10E-03	5.50E-02
hydrogen peroxide catabolic process	6	0.4	2.10E-03	5.50E-02
nucleobase biosynthetic process	5	0.4	4.60E-03	9.90E-02
mannose metabolic process	5	0.4	1.00E-02	1.80E-01
branching involved in salivary gland morphogenesis	6	0.4	1.50E-02	2.20E-01
chaperone mediated protein folding requiring cofactor	5	0.4	1.50E-02	2.20E-01
myofibril assembly	6	0.4	1.80E-02	2.70E-01
ER-associated protein catabolic process	5	0.4	2.00E-02	2.80E-01
cortical cytoskeleton organization	5	0.4	2.00E-02	2.80E-01
'de novo' posttranslational protein folding	5	0.4	2.60E-02	3.30E-01
'de novo' protein folding	5	0.4	2.60E-02	3.30E-01
NADP metabolic process	5	0.4	2.60E-02	3.30E-01
collagen fibril organization	6	0.4	2.80E-02	3.50E-01
actin filament-based movement	5	0.4	3.30E-02	3.90E-01
lung alveolus development	6	0.4	3.40E-02	3.90E-01
serine family amino acid metabolic process	6	0.4	3.40E-02	3.90E-01
lamellipodium assembly	5	0.4	4.10E-02	4.40E-01
protein amino acid O-linked glycosylation	5	0.4	4.10E-02	4.40E-01
purine ribonucleoside metabolic process	6	0.4	4.80E-02	4.80E-01
purine nucleoside metabolic process	6	0.4	4.80E-02	4.80E-01
T cell homeostasis	5	0.4	6.00E-02	5.60E-01
nuclear-transcribed mRNA catabolic process, nonsense-mediated decay	6	0.4	6.40E-02	5.70E-01
heme metabolic process	5	0.4	7.10E-02	6.00E-01
apoptotic nuclear changes	5	0.4	7.10E-02	6.00E-01
amino sugar metabolic process	5	0.4	7.10E-02	6.00E-01
positive regulation of protein polymerization	5	0.4	7.10E-02	6.00E-01
negative regulation of epithelial cell proliferation	6	0.4	7.30E-02	6.10E-01
nuclear export	6	0.4	7.30E-02	6.10E-01
antigen processing and presentation of exogenous antigen	6	0.4	8.30E-02	6.50E-01
regulation of smooth muscle cell proliferation	5	0.4	8.40E-02	6.40E-01
ER to Golgi vesicle-mediated transport	6	0.4	9.40E-02	6.70E-01
DNA catabolic process	5	0.4	9.70E-02	6.80E-01
IMP biosynthetic process	4	0.3	2.30E-03	5.80E-02
IMP metabolic process	4	0.3	2.30E-03	5.80E-02
positive regulation of protein complex disassembly	4	0.3	1.00E-02	1.80E-01
pentose-phosphate shunt	4	0.3	2.50E-02	3.30E-01
cellular copper ion homeostasis	4	0.3	4.60E-02	4.70E-01
asymmetric protein localization	4	0.3	4.60E-02	4.70E-01
glucosamine metabolic process	4	0.3	6.00E-02	5.50E-01
purine base metabolic process	4	0.3	6.00E-02	5.50E-01
copper ion homeostasis	4	0.3	6.00E-02	5.50E-01
N-acetylglucosamine metabolic process	4	0.3	6.00E-02	5.50E-01
glycine metabolic process	4	0.3	7.50E-02	6.10E-01
peptidyl-lysine modification	4	0.3	7.50E-02	6.10E-01
positive regulation of myeloid leukocyte differentiation	4	0.3	9.20E-02	6.70E-01
DNA fragmentation involved in apoptosis	4	0.3	9.20E-02	6.70E-01
membrane protein proteolysis	4	0.3	9.20E-02	6.70E-01
membrane protein ectodomain proteolysis	4	0.3	9.20E-02	6.70E-01
'de novo' IMP biosynthetic process	3	0.2	2.00E-02	2.90E-01
myoblast proliferation	3	0.2	2.00E-02	2.90E-01
N-glycan processing	3	0.2	3.80E-02	4.20E-01
ribosomal protein import into nucleus	3	0.2	3.80E-02	4.20E-01
amino sugar biosynthetic process	3	0.2	3.80E-02	4.20E-01

response to arsenic	3	0.2	3.80E-02	4.20E-01
skeletal myofibril assembly	3	0.2	6.00E-02	5.50E-01
ruffle organization	3	0.2	6.00E-02	5.50E-01
dichotomous subdivision of terminal units involved in salivary gland branching	3	0.2	6.00E-02	5.50E-01
cellular metabolic compound salvage	3	0.2	6.00E-02	5.50E-01
purine base biosynthetic process	3	0.2	6.00E-02	5.50E-01
hypusine metabolic process	3	0.2	8.60E-02	6.50E-01
purine deoxyribonucleoside triphosphate metabolic process	3	0.2	8.60E-02	6.50E-01
purine deoxyribonucleotide metabolic process	3	0.2	8.60E-02	6.50E-01
ribosomal small subunit biogenesis	3	0.2	8.60E-02	6.50E-01
peptidyl-lysine modification to hypusine	3	0.2	8.60E-02	6.50E-01
glycolipid catabolic process	3	0.2	8.60E-02	6.50E-01
protein neddylation	3	0.2	8.60E-02	6.50E-01
neurotransmitter receptor metabolic process	3	0.2	8.60E-02	6.50E-01
dichotomous subdivision of an epithelial terminal unit	3	0.2	8.60E-02	6.50E-01
cardiac myofibril assembly	3	0.2	8.60E-02	6.50E-01
notochord morphogenesis	3	0.2	8.60E-02	6.50E-01

Supplementary Table S7. A complete list of quantified HCPs in the culture supernatants during batch and fed-batch cultures.

Batch culture				
Identified Proteins_CV<20% (530)	Protein ID	day3	day5	day8
Clusterin	Clu	0.385	1.569	1.162
Beta-2-microglobulin	B2m	0.373	1.726	1.328
Metalloproteinase inhibitor 1	Timp1	0.346	1.750	1.148
SPARC	Sparc	0.315	1.545	0.871
Lactadherin	MFGE8	0.255	1.405	0.999
Legumain	Lgmn	0.245	0.882	0.951
Procollagen C-endopeptidase enhancer 1	PCOLCE	0.244	1.029	0.639
Syndecan	Sdc	0.223	0.913	0.681
Dickkopf-related protein 3	DKK3	0.222	1.031	0.675
Nidogen-1	Nid1	0.198	0.910	0.653
Sulfated glycoprotein 1	Psap	0.193	0.904	1.158
C-C motif chemokine 2	Ccl2	0.192	0.691	0.544
Chondroitin sulfate proteoglycan 4	Cspg4	0.191	0.733	0.424
14-3-3 protein epsilon	Ywhae	0.175	0.778	2.821
Decorin	DCN	0.167	0.873	0.796
Complement C1r-A subcomponent	C1ra	0.156	0.682	0.420
Ribonuclease T2	Rnaset2	0.152	0.560	0.408
Cathepsin Z	Ctsz	0.139	0.302	0.803
Galectin-3-binding protein	Lgals3bp	0.137	0.778	0.423
Nucleobindin-2	Nucb2	0.137	0.594	0.650
Metalloproteinase inhibitor 2	Timp2	0.134	0.730	0.330
N(4)-(Beta-N-acetylglucosaminyl)-L-asparaginase	Aga	0.132	0.580	0.593
C-C motif chemokine 7	Ccl7	0.129	0.316	0.000
Insulin-like growth factor-binding protein 4	IGFBP4	0.128	0.637	0.261
Lipoprotein lipase	Lpl	0.127	0.749	0.348
Tubulointerstitial nephritis antigen-like	Tinagl1	0.121	0.435	0.374
Cathepsin B	Ctsb	0.118	0.619	0.679
Glypican-1	Gpc1	0.117	0.388	0.317
Suprabasin	Sbsn	0.116	0.588	0.336
Peptidyl-prolyl cis-trans isomerase	Ppi	0.116	0.677	0.736
Peroxiredoxin-1	Prdx1	0.114	0.612	2.176
Sulfhydryl oxidase	QSOX	0.113	0.658	0.331
Nidogen-1	Nid1	0.112	0.500	0.452
Pyruvate kinase	Pkm	0.111	0.399	1.411
Thrombospondin-1	Thbs1	0.109	0.583	0.135
Nucleobindin-1	Nucb1	0.106	0.664	0.406
Peroxidasin-like	Pxdnl	0.104	0.416	0.289
Laminin subunit gamma-1	Lamc1	0.104	0.264	0.297
EGF-containing fibulin-like extracellular matrix pr	Efemp1	0.103	0.507	0.255
Complement C1q tumor necrosis factor-related	C1qtnf5	0.103	0.693	0.330
Laminin subunit alpha-5	Lama5	0.102	0.299	0.274
Cofilin-1	Cfl1	0.093	0.723	1.843
Galectin-1	Lgals1	0.088	0.515	0.948
Calcium-dependent serine proteinase	I79_001431	0.086	0.482	0.223
Inter-alpha-trypsin inhibitor heavy chain H5	Itih5	0.086	0.374	0.318
45 kDa calcium-binding protein	SDF4	0.086	0.403	0.173
Basement membrane-specific heparan sulfate pr	Hspg2	0.084	0.464	0.404
Phospholipid transfer protein	PLTP	0.083	0.522	0.325
CD166 antigen	Alcam	0.083	0.229	0.177
Serine protease HTRA1	Htra1	0.082	0.541	0.474
Laminin subunit beta-1	Lamb1	0.081	0.234	0.252
Follistatin-related protein 1	Fstl1	0.079	0.318	0.147
Semaphorin-3C	Sema3c	0.079	0.219	0.104
Matrix metalloproteinase-9	Mmp9	0.078	0.404	0.360
Lactadherin (Fragment)	MFGE8	0.078	0.382	0.315
78 kDa glucose-regulated protein	Hspa5	0.077	0.302	0.986
Epididymal secretory protein E1	Npc2	0.077	0.340	0.482
Annexin	Anx	0.077	0.464	0.779
Complement C3	C3	0.075	0.410	0.106
Amyloid beta A4 protein	App	0.072	0.440	0.493
C-X-C motif chemokine 3	Cxcr3	0.072	0.378	0.388
Peptidyl-prolyl cis-trans isomerase	Ppi	0.071	0.252	0.265
Soluble calcium-activated nucleotidase 1	Cant1	0.071	0.307	0.133
Lysosomal protective protein	Ctsa	0.070	0.271	0.531
MAM domain-containing protein 2	Mamdc2	0.070	0.258	0.088
Renin receptor	ATP6AP2	0.069	0.422	0.375
G-protein coupled receptor 56	Gpr56	0.068	0.343	0.179
Beta-hexosaminidase	Hexb	0.067	0.254	0.102
Semaphorin-3B	Sema3b	0.066	0.354	0.179
60S acidic ribosomal protein P2	Rplp2	0.064	0.375	1.076
Protein CYR61	Cyr61	0.064	0.177	0.127
Cathepsin D	Ctsd	0.064	0.224	0.439
Acid ceramidase	Asah1	0.062	0.197	0.302
Cathepsin L1	Ctsl	0.062	0.226	0.328
Extracellular matrix protein 1	ECM1	0.060	0.300	0.097
Lysosomal alpha-glucosidase	Gaa	0.058	0.297	0.309
Phosphatidylethanolamine-binding protein 1	Pebp1	0.057	0.329	0.696
Heat shock protein HSP 90-beta	Hsp90ab1	0.054	0.210	0.581
Calsyntenin-1	Clstn1	0.052	0.238	0.114
Interleukin-1 receptor-like 1	Il1rl1	0.051	0.123	0.000
Lysyl oxidase-like 4	LOXL4	0.051	0.077	0.024

Protein ID	day3	Protein ID	day5	Protein ID	day8
Clu	0.38	Timp1	1.75	Ywhae	2.82
B2m	0.37	B2m	1.73	Prdx1	2.18
Timp1	0.35	Clu	1.57	Cfl1	1.84
Sparc	0.32	Sparc	1.54	Pkm	1.41
MFGE8	0.25	MFGE8	1.41	B2m	1.33
Lgmn	0.24	DKK3	1.03	Clu	1.16
PCOLCE	0.24	PCOLCE	1.03	Psap	1.16
Sdc	0.22	Sdc	0.91	Timp1	1.15
DKK3	0.22	Nid1	0.91	Rplp2	1.08
Nid1	0.20	Psap	0.90	MFGE8	1.00
Psap	0.19	Lgmn	0.88	Hspa5	0.99
Ccl2	0.19	DCN	0.87	Lgmn	0.95
Cspg4	0.19	Lgals3bp	0.78	Lgals1	0.95
Ywhae	0.18	Ywhae	0.78	Arhgdia	0.94
DCN	0.17	Lpl	0.75	Sparc	0.87
C1ra	0.16	Cspg4	0.73	Ctsz	0.80
Rnaset2	0.15	Timp2	0.73	DCN	0.80
Ctsz	0.14	Cfl1	0.72	Pgk	0.79
Lgals3bp	0.14	C1qtnf5	0.69	Tkt	0.79
Nucb2	0.14	Ccl2	0.69	Anx	0.78
Timp2	0.13	C1ra	0.68	PSAT	0.76
Aga	0.13	Ppi	0.68	Ppi	0.74
Ccl7	0.13	Nucb1	0.66	Pebp1	0.70
IGFBP4	0.13	QSOX	0.66	Sdc	0.68
Lpl	0.13	IGFBP4	0.64	Anx	0.68
Tinagl1	0.12	Ctsb	0.62	Ctsb	0.68
Ctsb	0.12	Prdx1	0.61	DKK3	0.68
Gpc1	0.12	Nucb2	0.59	Akr1b1	0.67
Sbsn	0.12	Sbsn	0.59	Gstp	0.66
Ppi	0.12	Thbs1	0.58	Nid1	0.65
Prdx1	0.11	Aga	0.58	Nucb2	0.65
QSOX	0.11	Rnaset2	0.56	Hsp90b1	0.64
Nid1	0.11	Htra1	0.54	PCOLCE	0.64
Pkm	0.11	PLTP	0.52	Aga	0.59
Thbs1	0.11	Lgals1	0.51	Hsp90ab1	0.58
Nucb1	0.11	Efemp1	0.51	VAT1L	0.57
Pxdnl	0.10	Nid1	0.50	Pdia4	0.55
Lamc1	0.10	I79_001431	0.48	Ccl2	0.54
Efemp1	0.10	Anx	0.46	Tbca	0.54
C1qtnf5	0.10	Hspg2	0.46	Ctsa	0.53
Lama5	0.10	App	0.44	6Pgd	0.52
Cfl1	0.09	Tinagl1	0.43	App	0.49
Lgals1	0.09	ATP6AP2	0.42	Npc2	0.48
I79_001431	0.09	Pxdnl	0.42	Htra1	0.47
Itih5	0.09	C3	0.41	Ak2	0.47
SDF4	0.09	Mmp9	0.40	Psma7	0.46
Hspg2	0.08	SDF4	0.40	Gss	0.46
PLTP	0.08	Pkm	0.40	Nid1	0.45
Alcam	0.08	Gpc1	0.39	Ctsd	0.44
Htra1	0.08	MFGE8	0.38	RecName: Full	0.44
Lamb1	0.08	Cxcr3	0.38	Serpinb1a	0.43
Fstl1	0.08	Rplp2	0.37	Ast	0.43
Sema3c	0.08	Itih5	0.37	Cspg4	0.42
Mmp9	0.08	Sema3b	0.35	Lgals3bp	0.42
MFGE8	0.08	Gpr56	0.34	C1ra	0.42
Hspa5	0.08	Npc2	0.34	Eno1	0.42
Npc2	0.08	Oaf	0.34	Rnaset2	0.41
Anx	0.08	Pebp1	0.33	Nucb1	0.41
C3	0.08	Fstl1	0.32	Hspg2	0.40
App	0.07	Ccl7	0.32	Tpi1	0.40
Cxcr3	0.07	LOXL1	0.31	Cxcr3	0.39
Ppi	0.07	Cant1	0.31	Capg	0.38
Cant1	0.07	Hspa5	0.30	Ncl	0.38
Ctsa	0.07	Ctsz	0.30	ATP6AP2	0.37
Mamdc2	0.07	ECM1	0.30	Tinagl1	0.37
ATP6AP2	0.07	Lama5	0.30	Pdi	0.37
Gpr56	0.07	Gaa	0.30	Mmp9	0.36
Hexb	0.07	Anx	0.28	Hnrnpa2b1	0.35
Sema3b	0.07	Ctsa	0.27	Lpl	0.35
Rplp2	0.06	Pdpk1	0.27	Ldh	0.35
Cyr61	0.06	Lamc1	0.26	Eef1g	0.34
Ctsd	0.06	Mamdc2	0.26	Nme1	0.34
Asah1	0.06	Hexb	0.25	Sbsn	0.34
Ctsl	0.06	Arhgdia	0.25	Actn1	0.34
ECM1	0.06	Ppi	0.25	QSOX	0.33
Gaa	0.06	pam-b	0.25	Timp2	0.33
Pebp1	0.06	Clstn1	0.24	C1qtnf5	0.33
Hsp90ab1	0.05	Pgk	0.24	Ctsl	0.33
Clstn1	0.05	Lamb1	0.23	PLTP	0.32
Il1rl1	0.05	Alcam	0.23	Hmox1	0.32
LOXL4	0.05	Ctsl	0.23	Nudc	0.32

EMILIN-1	Emilin1	0.050	0.186	0.115
Amyloid-like protein 2	Aplp2	0.049	0.208	0.205
Beta-glucuronidase	Gusb	0.049	0.208	0.167
Vasorin	Vasn	0.047	0.209	0.121
Intercellular adhesion molecule 1	Icam1	0.047	0.199	0.212
Out at first protein-like	Oaf	0.046	0.337	0.175
Endoplasmin	Hsp90b1	0.046	0.165	0.643
Semaphorin-3E	Sema3e	0.046	0.155	0.084
Lysyl oxidase-like 1	LOXL1	0.045	0.311	0.174
V-type proton ATPase subunit S1	Atp6ap1	0.045	0.191	0.223
Phosphoglycerate kinase	Pgk	0.045	0.237	0.794
Protein disulfide-isomerase	Pdi	0.045	0.133	0.368
Procollagen-lysine,2-oxoglutarate 5-dioxygenase	Plod1	0.044	0.162	0.207
Retinoid-inducible serine carboxypeptidase	Scpep1	0.044	0.144	0.148
Leukocyte elastase inhibitor A	Serpinb1a	0.044	0.215	0.432
Transketolase	Tkt	0.043	0.208	0.785
Bone morphogenetic protein 1	BMP1	0.041	0.212	0.084
Triosephosphate isomerase	Tpi1	0.041	0.190	0.402
Annexin	Anx	0.041	0.280	0.680
Dystroglycan	Dag1	0.041	0.185	0.159
Nucleoside diphosphate kinase	Nme1	0.041	0.174	0.339
Glutathione S-transferase P	Gstp	0.040	0.194	0.665
Phosphoserine aminotransferase	PSAT	0.040	0.192	0.758
3-phosphoinositide-dependent protein kinase 1	Pdpk1	0.039	0.268	0.109
Peptidyl-glycine alpha-amidating monooxygenas	pam-b	0.039	0.247	0.194
Alpha-enolase	Eno1	0.036	0.190	0.415
Aldose reductase	Akr1b1	0.036	0.176	0.668
14-3-3 protein theta	Ywhaq	0.033	0.137	0.298
Agrin	Agrn	0.033	0.052	0.099
Rho GDP-dissociation inhibitor 1	Arhgdia	0.032	0.253	0.939
6-phosphogluconate dehydrogenase, decarboxyl	6Pgd	0.032	0.131	0.516
L-lactate dehydrogenase	Ldh	0.031	0.094	0.345
Tissue alpha-L-fucosidase	Fuca1	0.031	0.137	0.048
Twisted gastrulation protein-like 1	Twsg1	0.030	0.116	0.157
Elongation factor 1-gamma	Eef1g	0.029	0.103	0.343
N-acetylgalactosaminyltransferase 7	Galnt7	0.029	0.129	0.057
Lysyl oxidase-like 3	LOXL3	0.027	0.164	0.061
Alpha-galactosidase A	Gla	0.027	0.097	0.096
Proteasome subunit alpha type-7 (Fragment)	Psma7	0.027	0.089	0.461
Adenylate kinase 2, mitochondrial	Ak2	0.027	0.072	0.468
Sialate O-acetylesterase	Siae	0.027	0.137	0.031
Neutral alpha-glucosidase AB	Ganab	0.026	0.086	0.193
Golgi apparatus protein 1	GLG1	0.026	0.192	0.117
N-acetyllactosaminide beta-1,3-N-acetylglucosan	B3GNT	0.026	0.165	0.069
Reticulocalbin-3	Rcn3	0.026	0.099	0.251
Nuclear migration protein nudC	Nudc	0.026	0.098	0.323
Cell division control protein 42-like	Cdc42	0.025	0.099	0.289
Nucleolin	Ncl	0.024	0.140	0.375
Coiled-coil domain-containing protein 80	Ccdc80	0.023	0.179	0.054
Glutathione S-transferase omega-1	Gsto1	0.023	0.074	0.244
Mpv17-like protein 2	Mpv17l	0.022	0.043	0.038
Nascent polypeptide-associated complex subunit	Naca	0.022	0.084	0.235
Pentraxin-related protein PTX3	Ptx3	0.022	0.013	0.003
Thioredoxin reductase 1, cytoplasmic	Txnrd1	0.022	0.111	0.299
Aspartate aminotransferase	Ast	0.022	0.096	0.425
Glutamine synthetase	Glul	0.022	0.163	0.313
Alpha-actinin-1	Actn1	0.021	0.118	0.336
Lysosomal alpha-mannosidase	Man2b1	0.021	0.129	0.042
40S ribosomal protein S3a	Rps3a	0.020	0.102	0.124
Alpha-mannosidase 2	Man2a1	0.019	0.142	0.059
Inorganic pyrophosphatase	Ppa	0.018	0.103	0.279
Prostaglandin F2 receptor negative regulator	Ptgfrn	0.018	0.113	0.071
Heat shock cognate 71 kDa protein	Hspa8	0.018	0.085	0.150
NSFL1 cofactor p47	Nsfl1c	0.018	0.075	0.316
Synaptic vesicle membrane protein VAT-1-like	VAT1L	0.018	0.158	0.572
Tumor necrosis factor ligand superfamily membe	Tnfsf9	0.017	0.132	0.165
Pantetheinase	Vnn1	0.017	0.053	0.088
Protein disulfide-isomerase A4	Pdia4	0.017	0.064	0.552
Glutathione synthetase	Gss	0.016	0.094	0.457
Low-density lipoprotein receptor	Lrp	0.016	0.048	0.105
Heat shock protein HSP 90-alpha	Hsp90aa1	0.016	0.073	0.253
Calumenin	Calu	0.016	0.071	0.130
Collagen alpha-1(III) chain	COL3A1	0.015	0.213	0.054
Collagen alpha-1(XII) chain (Fragment)	Col12a1	0.015	0.042	0.047
Glucosidase 2 subunit beta	Prkcsh	0.014	0.044	0.141
Ubiquitin-conjugating enzyme E2 N (Fragment)	Ube2n	0.013	0.075	0.288
CD109 antigen	Cd109	0.013	0.070	0.043
Macrophage-capping protein	Capg	0.013	0.111	0.383
FK506-binding protein 9	Fkbp9	0.013	0.035	0.166
CD63 antigen	Cd63	0.012	0.035	0.092
Protein disulfide-isomerase	Pdi	0.012	0.059	0.057
Lipase	Lpl	0.012	0.037	0.003
Disintegrin and metalloproteinase domain-conta	Adam10	0.011	0.067	0.002
CD44 antigen	Cd44	0.010	0.044	0.043
Ran-specific GTPase-activating protein	Ranbp1	0.010	0.033	0.145
Heterogeneous nuclear ribonucleoproteins A2/B	Hnrnpa2b1	0.010	0.060	0.353

Emilin1	0.05	Ctsd	0.22	Itih5	0.32
Aplp2	0.05	Sema3c	0.22	Gpc1	0.32
Gusb	0.05	Serpinb1a	0.21	Nsfl1c	0.32
Vasn	0.05	COL3A1	0.21	MFGE8	0.32
Icam1	0.05	BMP1	0.21	Glul	0.31
Oaf	0.05	Hsp90ab1	0.21	Gaa	0.31
Hsp90b1	0.05	Vasn	0.21	Asah1	0.30
Sema3e	0.05	Aplp2	0.21	Txnrd1	0.30
LOXL1	0.05	Gusb	0.21	Ywhaq	0.30
Atp6ap1	0.05	Tkt	0.21	Lamc1	0.30
Pgk	0.05	Icam1	0.20	Pxdnl	0.29
Pdi	0.04	Asah1	0.20	Cdc42	0.29
Plod1	0.04	Gstp	0.19	Ube2n	0.29
Scpep1	0.04	GLG1	0.19	Lap3	0.28
Serpinb1a	0.04	PSAT	0.19	St13	0.28
Tkt	0.04	Atp6ap1	0.19	Ppa	0.28
BMP1	0.04	Tpi1	0.19	Lama5	0.27
Tpi1	0.04	Eno1	0.19	Ppi	0.26
Anx	0.04	Emilin1	0.19	IGFBP4	0.26
Dag1	0.04	Dag1	0.18	Efemp1	0.26
Nme1	0.04	Ccdc80	0.18	Hsp90aa1	0.25
Gstp	0.04	Cyr61	0.18	Lamb1	0.25
PSAT	0.04	Akr1b1	0.18	Pfdn6	0.25
Pdpk1	0.04	Nme1	0.17	Rcn3	0.25
pam-b	0.04	B3GNT	0.16	Gsto1	0.24
Eno1	0.04	Hsp90b1	0.16	Naca	0.24
Akr1b1	0.04	LOXL3	0.16	Mdh1	0.23
Ywhaq	0.03	Glul	0.16	I79_001431	0.22
Agrn	0.03	Plod1	0.16	Atp6ap1	0.22
Arhgdia	0.03	VAT1L	0.16	Snrpd3	0.21
6Pgd	0.03	Sema3e	0.16	Icam1	0.21
Ldh	0.03	Scpep1	0.14	Pgls	0.21
Fuca1	0.03	Man2a1	0.14	Cyb5	0.21
Twsg1	0.03	Ncl	0.14	Lsm8	0.21
Eef1g	0.03	Fuca1	0.14	Plod1	0.21
Galnt7	0.03	Ywhaq	0.14	Aplp2	0.20
LOXL3	0.03	Siae	0.14	Yars	0.20
Gla	0.03	Pdi	0.13	Thop1	0.20
Psma7	0.03	Tnfsf9	0.13	pam-b	0.19
Ak2	0.03	6Pgd	0.13	Ganab	0.19
Siae	0.03	Galnt7	0.13	Glrx3	0.19
Ganab	0.03	Man2b1	0.13	Fasn	0.19
GLG1	0.03	Il1rl1	0.12	Sema3b	0.18
B3GNT	0.03	Actn1	0.12	Gpr56	0.18
Rcn3	0.03	Twsg1	0.12	Nit2	0.18
Nudc	0.03	Ptgfrn	0.11	Alcam	0.18
Cdc42	0.02	Txnrd1	0.11	Oaf	0.18
Ncl	0.02	Capg	0.11	LOXL1	0.17
Ccdc80	0.02	Ppa	0.10	Flna	0.17
Gsto1	0.02	Eef1g	0.10	Impdh2	0.17
Mpv17l	0.02	Rps3a	0.10	SDF4	0.17
Naca	0.02	Rcn3	0.10	S100a6	0.17
Ptx3	0.02	Cdc42	0.10	Gusb	0.17
Txnrd1	0.02	Nudc	0.10	Fkbp9	0.17
Ast	0.02	Gla	0.10	Tnfsf9	0.16
Glul	0.02	Ast	0.10	Dag1	0.16
Actn1	0.02	Gss	0.09	Twsg1	0.16
Man2b1	0.02	Ldh	0.09	S100a13	0.15
Rps3a	0.02	Psma7	0.09	Hspa8	0.15
Man2a1	0.02	Ganab	0.09	Ruvbl1	0.15
Ppa	0.02	Hspa8	0.09	Scpep1	0.15
Ptgfrn	0.02	Naca	0.08	Fstl1	0.15
Hspa8	0.02	LOXL4	0.08	Sub1	0.15
Nsfl1c	0.02	Ube2n	0.08	Ranbp1	0.14
VAT1L	0.02	Snrpd3	0.08	Isoc1	0.14
Tnfsf9	0.02	Nsfl1c	0.07	Nucb1	0.14
Vnn1	0.02	Gsto1	0.07	Hnrnpa3	0.14
Pdia4	0.02	Hsp90aa1	0.07	Rprd1b	0.14
Gss	0.02	Ak2	0.07	Atp6v1a	0.14
Lrp	0.02	Calu	0.07	Prkcsh	0.14
Hsp90aa1	0.02	Cd109	0.07	Pafah1b1	0.14
Calu	0.02	Adam10	0.07	Thbs1	0.14
COL3A1	0.02	Pdia4	0.06	Ptges3	0.13
Col12a1	0.02	Hnrnpa2b1	0.06	Cant1	0.13
Prkcsh	0.01	Pdi	0.06	Tars	0.13
Ube2n	0.01	Eif3i	0.06	Grb2	0.13
Cd109	0.01	SGSH	0.05	Fkbp14	0.13
Capg	0.01	Vnn1	0.05	Calu	0.13
Fkbp9	0.01	Agrn	0.05	Cyr61	0.13
Cd63	0.01	Lrp	0.05	Otub1	0.13
Pdi	0.01	Cd44	0.04	Uap1	0.12
Lpl	0.01	Prkcsh	0.04	Rps3a	0.12
Adam10	0.01	Mpv17l	0.04	PSME3	0.12
Cd44	0.01	Col12a1	0.04	Abhd14b	0.12
Ranbp1	0.01	Lpl	0.04	Iah1	0.12
Hnrnpa2b1	0.01	LOC100689010	0.04	ETF1	0.12

Eukaryotic translation initiation factor 3 subunit	Eif3i	0.010	0.058	0.117
MHC class I antigen Hm1-C2	LOC100689010	0.009	0.036	0.000
Thimet oligopeptidase	Thop1	0.008	0.029	0.195
Putative uncharacterized protein	Nrg1	0.008	0.000	0.000
Protein OS-9	Os9	0.007	0.033	0.002
Beta-1,4-galactosyltransferase 1	B4galt1	0.007	0.017	0.030
Protocadherin Fat 1	FAT1	0.007	0.020	0.002
EGF-containing fibulin-like extracellular matrix p	Efemp2	0.007	0.031	0.000
UDP-N-acetylhexosamine pyrophosphorylase	Uap1	0.006	0.016	0.125
NKG2D ligand 4	Nkg2d4	0.006	0.029	0.007
Poly(RC)-binding protein 1	Pcbp1	0.006	0.028	0.113
Lamina-associated polypeptide 2, isoforms alpha	Tmpo	0.006	0.032	0.106
Syndecan	Sdc	0.005	0.000	0.000
Beta-galactosidase (Fragment)	Glb1	0.005	0.009	0.010
Cysteine-rich motor neuron 1 protein	Crim1	0.005	0.021	0.022
Fatty acid synthase	Fasn	0.005	0.012	0.188
Cytosol aminopeptidase	Lap3	0.004	0.025	0.282
Selenoprotein N	Sepn	0.004	0.003	0.000
Protein kinase C and casein kinase substrate in	Pacsin2	0.004	0.011	0.081
Eukaryotic translation initiation factor 2 subunit	Eif2s2	0.004	0.019	0.082
Heterochromatin protein 1 gamma	Hp1g	0.003	0.000	0.099
Filamin-A	Flna	0.003	0.035	0.174
Calreticulin	Calr	0.003	0.017	0.066
Pyridoxal kinase	Pdxk	0.003	0.006	0.017
Sortilin (Fragment)	Sort1	0.003	0.017	0.054
Tubulin beta-5 chain	Tubb5	0.003	0.014	0.019
Glycosyltransferase 25 family member 1	GLT25D1	0.003	0.000	0.000
Tubulin-specific chaperone A	Tbca	0.003	0.028	0.538
Small nuclear ribonucleoprotein Sm D3	Snrpd3	0.002	0.075	0.214
Netrin-4 (Fragment)	Ntn4	0.002	0.002	0.000
40S ribosomal protein S2	Rps2	0.002	0.000	0.000
FK506-binding protein 14	Fkbp14	0.002	0.000	0.131
Tyrosyl-tRNA synthetase, cytoplasmic	Yars	0.002	0.014	0.197
60S acidic ribosomal protein P1	Rplp1	0.002	0.000	0.000
60S ribosomal protein L12	Rpl12	0.002	0.000	0.019
Replication protein A 32 kDa subunit	Rpa2	0.002	0.000	0.039
Prefoldin subunit 6	Pfdn6	0.002	0.000	0.251
Plectin (Fragment)	Plec	0.002	0.011	0.023
Glyceraldehyde-3-phosphate dehydrogenase	Gapdh	0.002	0.000	0.000
Isoamyl acetate-hydrolyzing esterase 1-like	Iah1	0.001	0.000	0.121
AP-1 complex subunit beta-1	Ap1b1	0.001	0.004	0.051
Clathrin light chain B (Fragment)	Cltb	0.001	0.006	0.086
Cytochrome b5	Cyb5	0.001	0.013	0.211
60S ribosomal protein L27a	Rpl27a	0.001	0.000	0.042
Glyceraldehyde-3-phosphate dehydrogenase	Gapdh	0.001	0.000	0.007
Magnesium-dependent phosphatase 1	Mdp1	0.001	0.000	0.065
Contactin-1	Cntn1	0.001	0.002	0.000
Ubiquitin thioesterase OTUB1	Otub1	0.001	0.012	0.000
Thioredoxin domain-containing protein 17	Txndc17	0.001	0.006	0.103
Glutaredoxin-3	Glrx3	0.001	0.012	0.192
Nucleosome assembly protein 1-like 4	Nap1l4	0.001	0.003	0.092
Fibroblast growth factor	Fgfr	0.001	0.003	0.059
60S ribosomal protein L7	Rpl7	0.001	0.000	0.000
ADP-ribosylation factor 4	Arf4	0.001	0.006	0.000
cAMP-dependent protein kinase type I-alpha re	Prkar1a	0.001	0.008	0.081
Liver carboxylesterase 4	Ces4	0.001	0.000	0.003
Uncharacterized protein C7orf50-like	Cg050	0.001	0.000	0.040
Collagen alpha-1(VI) chain	COL6A1	0.001	0.019	0.000
Keratin, type II cytoskeletal 2 epidermal	Krt2	0.001	0.002	0.005
Protein SET	Set	0.001	0.000	0.014
ADP-sugar pyrophosphatase	Nudt5	0.001	0.004	0.113
26S proteasome non-ATPase regulatory subunit	Psmd2	0.001	0.001	0.046
Methylosome subunit pICln	Clns1a	0.001	0.021	0.081
H-2 class I histocompatibility antigen, K-W28 al	H2-K1	0.001	0.003	0.000
Zinc finger homeobox protein 2	Zeb2	0.001	0.000	0.000
A disintegrin and metalloproteinase with thromb	Adamts18	0.001	0.001	0.003
Fumarate hydratase, mitochondrial	Fh	0.001	0.000	0.094
Omega-amidase NIT2	Nit2	0.001	0.019	0.177
Guanine nucleotide-binding protein G(I)/G(S)/G(	Gnb2	0.001	0.000	0.011
Malate dehydrogenase (Fragment)	Mdh1	0.001	0.000	0.234
Phosphopantothenate--cysteine ligase	Ppcs	0.001	0.000	0.088
Serine-threonine kinase receptor-associated prot	Strap	0.001	0.003	0.069
Histone H3.1t	Hist3h3	0.001	0.000	0.000
Ataxin-7-like protein 3	Atxn7l3	0.001	0.003	0.012
PDZ and LIM domain protein 1	Pdlim1	0.001	0.004	0.071
Voltage-gated potassium channel subunit beta-2	Kcnab2	0.001	0.003	0.089
Calcium-dependent secretion activator 2 (Fragm	Cadps2	0.001	0.000	0.000
Bromodomain-containing protein 2	Brd2	0.001	0.003	0.002
Chitinase domain-containing protein 1	Chid1	0.001	0.010	0.080
Layilin	Layn	0.001	0.006	0.011
H-2 class I histocompatibility antigen, D-D alpha	H2-D1	0.001	0.001	0.001
Heterogeneous nuclear ribonucleoprotein G	Hnrnpg	0.001	0.013	0.022
Heat shock cognate 71 kDa protein	Hspa8	0.001	0.000	0.005
Protein C15orf2	C15orf2	0.000	0.000	0.000
Drebrin-like protein	Dbnl	0.000	0.000	0.040
Bleomycin hydrolase	Blmh	0.000	0.004	0.102

Eif3i	0.01	Cd63	0.04	Vasn	0.12
LOC100689010	0.01	Flna	0.03	Eif3i	0.12
Thop1	0.01	Fkbp9	0.03	GLG1	0.12
Nrg1	0.01	Os9	0.03	Emilin1	0.12
Os9	0.01	Ranbp1	0.03	Clstn1	0.11
B4galt1	0.01	RecName: Full	0.03	Nudt5	0.11
FAT1	0.01	Tmpo	0.03	Pcbp1	0.11
Efemp2	0.01	Efemp2	0.03	Tardbp	0.11
Uap1	0.01	Thop1	0.03	Fubp1	0.11
Nkg2d4	0.01	Nkg2d4	0.03	Bid	0.11
Pcbp1	0.01	Tbca	0.03	Pdpk1	0.11
Tmpo	0.01	Pcbp1	0.03	C3	0.11
Sdc	0.01	Lap3	0.03	Tmpo	0.11
Glb1	0.00	S100a6	0.02	Lrp	0.10
Crim1	0.00	Clns1a	0.02	Calm	0.10
Fasn	0.00	Crim1	0.02	Dhps	0.10
Lap3	0.00	FAT1	0.02	Arcn1	0.10
Sepn	0.00	Nit2	0.02	Sema3c	0.10
Pacsin2	0.00	Fah	0.02	Txndc17	0.10
Eif2s2	0.00	Eif2s2	0.02	Blmh	0.10
Hp1g	0.00	COL6A1	0.02	Hexb	0.10
Flna	0.00	B4galt1	0.03	Hp1g	0.10
Calr	0.00	Calr	0.02	Agrn	0.10
Pdxk	0.00	Sort1	0.02	Gabarapl2	0.10
Sort1	0.00	Uap1	0.02	Clta	0.10
Tubb5	0.00	Tubb5	0.01	Myg1	0.10
GLT25D1	0.00	Yars	0.01	Fah	0.10
Tbca	0.00	Ptx3	0.01	Eif3e	0.10
Snrpd3	0.00	Cyb5	0.01	Dnph1	0.10
Ntn4	0.00	Hnrnpg	0.01	ECM1	0.10
Rps2	0.00	Fasn	0.01	Gla	0.10
Fkbp14	0.00	Fubp1	0.01	Bag3	0.09
Yars	0.00	Otub1	0.01	Fh	0.09
Rplp1	0.00	Glrx3	0.01	Sirt1	0.09
Rpl12	0.00	Pacsin2	0.01	Nap1l4	0.09
Rpa2	0.00	Plec	0.01	Cd63	0.09
Pfdn6	0.00	Chid1	0.01	Vbp1	0.09
Plec	0.00	Rab6a	0.01	Crip2	0.09
Gapdh	0.00	Fkbp1a	0.01	Eef1e1	0.09
Iah1	0.00	Pafah1b1	0.01	Kcnab2	0.09
Ap1b1	0.00	Glb1	0.01	Vnn1	0.09
Cltb	0.00	Khsrp	0.01	Ppcs	0.09
Cyb5	0.00	Hist1h2b	0.01	Mamdc2	0.09
Rpl27a	0.00	Prkar1a	0.01	Chordc1	0.09
Gapdh	0.00	PSME3	0.01	Sri	0.09
Mdp1	0.00	Arcn1	0.01	Cltb	0.09
Cntn1	0.00	Cltb	0.01	BMP1	0.08
Otub1	0.00	Pdxk	0.01	Acly	0.08
Txndc17	0.00	Txndc17	0.01	Sema3e	0.08
Glrx3	0.00	Arf4	0.01	Iqgap1	0.08
Nap1l4	0.00	Layn	0.01	Dnajc8	0.08
Fgfr	0.00	FERMT2	0.01	Eif2s2	0.08
Rpl7	0.00	Hist1h4	0.01	Pacsin2	0.08
Arf4	0.00	Iqgap1	0.00	Clns1a	0.08
Prkar1a	0.00	Gapdh	0.00	Prkar1a	0.08
Ces4	0.00	Tars	0.00	Chid1	0.08
Cg050	0.00	Nudt5	0.00	Csnk2b	0.08
COL6A1	0.00	St8sia4	0.00	Dars	0.08
Krt2	0.00	Blmh	0.00	Ada	0.08
Set	0.00	Bag3	0.00	Hnrnpll	0.07
Nudt5	0.00	Otub1	0.00	Pdlim1	0.07
Psmd2	0.00	Plekhg2	0.00	Ptgfrn	0.07
Clns1a	0.00	Pdlim1	0.00	FERMT2	0.07
H2-K1	0.00	Dars	0.00	Pafah1b1	0.07
Zeb2	0.00	Gnpda1	0.00	Khsrp	0.07
Adamts18	0.00	Krt82	0.00	B3GNT	0.07
Fh	0.00	Ap1b1	0.00	Strap	0.07
Nit2	0.00	Ddx5	0.00	Gmfb	0.07
Gnb2	0.00	Kcnab2	0.00	Uso1	0.07
Mdh1	0.00	Rprd1b	0.00	Htra2	0.07
Ppcs	0.00	Pcbp2	0.00	Calr	0.07
Strap	0.00	Sepn	0.00	Mdp1	0.06
Hist3h3	0.00	Strap	0.00	Adprh	0.06
Atxn7l3	0.00	Atxn7l3	0.00	Rpl7a	0.06
Pdlim1	0.00	Psmd7	0.00	Cycs	0.06
Kcnab2	0.00	Nap1l4	0.00	Prpf19	0.06
Cadps2	0.00	Fgfr	0.00	Ppm1f	0.06
Brd2	0.00	Des	0.00	Prmt5	0.06
Chid1	0.00	Brd2	0.00	LOXL3	0.06
Layn	0.00	Ptprj	0.00	Imnl1	0.06
H2-D1	0.00	H2-K1	0.00	Man2a1	0.06
Hnrnpg	0.00	Atp1a1	0.00	Fgfr	0.06
Hspa8	0.00	Ssb	0.00	Scly	0.06
Protein C15orf2	0.00	Krt2	0.00	Psmg4	0.06
Dbnl	0.00	Cntn1	0.00	Zpr1	0.06
Blmh	0.00	Tuba8	0.00	Rnpep	0.06

CMP-N-acetylneuraminate-poly-alpha-2, 8-sialylt	St8sia4	0.000	0.004	0.000
Elongation factor 1-alpha 1	Eef1a1	0.000	0.000	0.000
Aspartyl-tRNA synthetase, cytoplasmic	Dars	0.000	0.004	0.077
Keratin, type II cytoskeletal 8	Krt8	0.000	0.000	0.000
4-trimethylaminobutyraldehyde dehydrogenase	Aldh9a1	0.000	0.000	0.017
Coatomer subunit delta	Arcn1	0.000	0.007	0.104
N-sulphoglucosamine sulphohydrolase	SGSH	0.000	0.053	0.002
Phospholipase D3	Pld3	0.000	0.000	0.015
Brain-specific angiogenesis inhibitor 1-associated	Baiap2	0.000	0.000	0.010
BAG family molecular chaperone regulator 3	Bag3	0.000	0.004	0.095
Protein FAM178A	Slf2	0.000	0.000	0.001
Histone-lysine N-methyltransferase SETDB1	Setdb1	0.000	0.000	0.000
Heat shock-related 70 kDa protein 2	Hspa2	0.000	0.000	0.012
Putative ATP-dependent RNA helicase DDX5	Ddx5	0.000	0.004	0.004
V-type proton ATPase catalytic subunit A	Atp6v1a	0.000	0.002	0.141
Aminopeptidase B	Rnpep	0.000	0.000	0.059
Leucyl-tRNA synthetase, cytoplasmic	Lars	0.000	0.000	0.028
Mannan-binding lectin serine protease 1 (Fragm	Masp1	0.000	0.000	0.000
U5 small nuclear ribonucleoprotein 200 kDa heli	Snrnp200	0.000	0.000	0.017
Sodium/potassium-transporting ATPase subunit	Atp1a1	0.000	0.003	0.020
Splicing factor 3 subunit 1	Sf3a1	0.000	0.001	0.057
Spectrin beta chain, brain 1	Sptbn1	0.000	0.000	0.026
Phospholipase A-2-activating protein	Plaa	0.000	0.001	0.037
Golgin subfamily B member 1	Golgb1	0.000	0.001	0.000
Brain-specific angiogenesis inhibitor 2	Bai2	0.000	0.000	0.000
Serrate RNA effector molecule-like	Srrt	0.000	0.000	0.005
RING finger protein 160	Ltn1	0.000	0.000	0.000
Myosin-10	Myh10	0.000	0.000	0.005
Trifunctional purine biosynthetic protein adenosi	Gart	0.000	0.000	0.000
Tudor domain-containing protein 7	Tdrd7	0.000	0.001	0.001
Transcriptional regulator ATRX	Atrx	0.000	0.001	0.000
Sodium/potassium-transporting ATPase subunit	Atp1a2	0.000	0.000	0.000
Uncharacterized protein KIAA1602-like	KIAA1602-like	0.000	0.000	0.002
Fibrillin-1	Fbn1	0.000	0.000	0.000
Type VII collagen (Fragment)	Col7a1	0.000	0.002	0.002
Myosin-XVIIIa	Myo18a	0.000	0.000	0.002
Acetyl-CoA carboxylase 2	Acacb	0.000	0.000	0.001
Zinc finger protein ZPR1	Zpr1	0.000	0.000	0.059
Zinc finger CCCH domain-containing protein 15	Zc3h15	0.000	0.000	0.015
Putative ATP-dependent RNA helicase YTHDC2	Ythdc2	0.000	0.001	0.002
von Willebrand factor A domain-containing prot	Vwa5a	0.000	0.001	0.006
Vacuolar protein sorting-associated protein 35	Vps35	0.000	0.001	0.038
Vacuolar protein sorting-associated protein 26A	Vps26a	0.000	0.000	0.033
Vimentin (Fragment)	Vim	0.000	0.000	0.002
Prefoldin subunit 3	Vbp1	0.000	0.000	0.011
Prefoldin subunit 3	Vbp1	0.000	0.000	0.091
General vesicular transport factor p115	Uso1	0.000	0.000	0.067
Uncharacterized protein C14orf45-like	C14orf45-like	0.000	0.000	0.010
Uncharacterized protein C10orf78-like	C10orf78-like	0.000	0.000	0.006
Ubiquitin	Ubiquitin	0.000	0.000	0.002
Ubiquitin-conjugating enzyme E2 D3	Ube2d3	0.000	0.000	0.022
Tubulin beta-4 chain	Tubb4	0.000	0.000	0.002
Tubulin beta-3 chain	Tubb3	0.000	0.000	0.001
Tubulin alpha-8 chain	Tuba8	0.000	0.002	0.000
Tetratricopeptide repeat protein 15	Ttc15	0.000	0.000	0.003
Translin	Tsn	0.000	0.000	0.019
Cdc42-interacting protein 4	Trip10	0.000	0.000	0.033
Trafficking protein particle complex subunit 3	Trappc3	0.000	0.000	0.017
Heat shock protein 75 kDa, mitochondrial	Trap1	0.000	0.000	0.002
Tumor suppressor p53-binding protein 1	Tp53bp1	0.000	0.000	0.001
182 kDa tankyrase-1-binding protein	Tnks1bp1	0.000	0.001	0.010
Toll-like receptor 9 (Fragment)	Tlr9	0.000	0.000	0.013
THUMP domain-containing protein 1	Thumpd1	0.000	0.000	0.032
Thiamine-triphosphatase	Thtpa	0.000	0.000	0.028
Teneurin-4	Tenm4	0.000	0.000	0.001
Transcription elongation factor B polypeptide 2	Tceb2	0.000	0.000	0.012
Threonyl-tRNA synthetase, cytoplasmic	Tars	0.000	0.004	0.132
TAR DNA-binding protein 43	Tardbp	0.000	0.000	0.111
Transaldolase	Taldo1	0.000	0.000	0.007
Activated RNA polymerase II transcriptional coac	Sub1	0.000	0.000	0.145
Stress-induced-phosphoprotein 1	Stip1	0.000	0.000	0.010
CMP-N-acetylneuraminate-beta-galactosamide-a	St3gal	0.000	0.000	0.003
Hsc70-interacting protein	St13	0.000	0.000	0.282
Lupus La protein-like	Ssb	0.000	0.003	0.028
Sorcin	Sri	0.000	0.000	0.086
Sorting nexin-5	Snx5	0.000	0.000	0.044
Sorting nexin-2	Snx2	0.000	0.000	0.008
U2 small nuclear ribonucleoprotein B''	Snrpb2	0.000	0.000	0.006
Solute carrier family 2, facilitated glucose transp	Slc2a1	0.000	0.000	0.030
Nicotinamide phosphoribosyltransferase (Fragme	Sirt1	0.000	0.000	0.092
Splicing factor 3B subunit 1	Sf3b1	0.000	0.000	0.028
Splicing factor 1	Sf1	0.000	0.000	0.053
Protein transport protein Sec24C	Sec24c	0.000	0.000	0.036
Protein transport protein Sec23B	Sec23b	0.000	0.000	0.024
Vesicle-trafficking protein SEC22b	Sec22b	0.000	0.000	0.016
Protein sidekick-2	Sdk2	0.000	0.001	0.000

St8sia4	0.00	Ruvbl1	0.00	Sf3a1	0.06
Eef1a1	0.00	Imnl1	0.00	Pdi	0.06
Dars	0.00	Pak2	0.00	Galnt7	0.06
Krt8	0.00	Ntn4	0.00	Ccdc80	0.05
Aldh9a1	0.00	Atp6v1a	0.00	COL3A1	0.05
Arcn1	0.00	Asns	0.00	Sort1	0.05
SGSH	0.00	Col7a1	0.00	Pdap1	0.05
Pld3	0.00	Kars	0.00	Psmd7	0.05
Baiap2	0.00	Psmd2	0.00	Sf1	0.05
Bag3	0.00	Vps35	0.00	Ap1b1	0.05
Slf2	0.00	Cacna1b	0.00	Rps27	0.05
Setdb1	0.00	Vwa5a	0.00	Atp6v1b2	0.05
Hspa2	0.00	Plaa	0.00	Atp6v1e1	0.05
Ddx5	0.00	H2-D1	0.00	Copa	0.05
Atp6v1a	0.00	Sf3a1	0.00	Fuca1	0.05
Rnpep	0.00	Nlgn3	0.00	Dctpp1	0.05
Lars	0.00	Nucb1	0.00	Col12a1	0.05
Masp1	0.00	Adamts18	0.00	Puf60	0.05
Snrnp200	0.00	Lars2	0.00	Psmd2	0.05
Atp1a1	0.00	Chd6	0.00	Cnrip1	0.05
Sf3a1	0.00	Epb41l2	0.00	Nt5c2	0.05
Sptbn1	0.00	Ap3b1	0.00	Eif4a2	0.04
Plaa	0.00	Sdk2	0.00	Snx5	0.04
Golgb1	0.00	Ythdc2	0.00	Rpa3	0.04
Bai2	0.00	Brca2	0.00	Cd44	0.04
Srrt	0.00	C7	0.00	Cd109	0.04
Ltn1	0.00	Crybg3	0.00	Rpl27a	0.04
Myh10	0.00	Huwe1	0.00	Dr1	0.04
Gart	0.00	Golgb1	0.00	Man2b1	0.04
Tdrd7	0.00	Apob	0.00	Pgm3	0.04
Atrx	0.00	Tnks1bp1	0.00	Ap1g1	0.04
Atp1a2	0.00	Kiaa1468	0.00	Map	0.04
KIAA1602-like	0.00	Atrx	0.00	Cg050	0.04
Fbn1	0.00	Pcdhb6	0.00	Dbnl	0.04
Col7a1	0.00	Tdrd7	0.00	Rpa2	0.04
Myo18a	0.00	Flnc	0.00	Mpv17l	0.04
Acacb	0.00	Rpl6l	0.00	Vps35	0.04
Zpr1	0.00	Zpr1	0.00	Plaa	0.04
Zc3h15	0.00	Zeb2	0.00	Eif3c	0.04
Ythdc2	0.00	Zc3h15	0.00	Fus	0.04
Vwa5a	0.00	Vps26a	0.00	Nrg1	0.04
Vps35	0.00	Vim	0.00	Sec24c	0.04
Vps26a	0.00	Vbp1	0.00	Dnm	0.04
Vim	0.00	Vbp1	0.00	Sart3	0.04
Vbp1	0.00	Uso1	0.00	Gstt2	0.04
Vbp1	0.00	Uncharacterized p	0.00	Actr2	0.03
Uso1	0.00	Uncharacterized p	0.00	Nono	0.03
Uncharacterized p	0.00	Ubiquitin	0.00	Dera	0.03
Uncharacterized p	0.00	Ube2d3	0.00	Fam98b	0.03
Ubiquitin	0.00	Tubb4	0.00	Trip10	0.03
Ube2d3	0.00	Tubb3	0.00	Vps26a	0.03
Tubb4	0.00	Ttc15	0.00	Ruvbl2	0.03
Tubb3	0.00	Tsn	0.00	Nqo2	0.03
Tuba8	0.00	Trip10	0.00	Thumpd1	0.03
Ttc15	0.00	Trappc3	0.00	Kpna2	0.03
Tsn	0.00	Trap1	0.00	Pdcl3	0.03
Trip10	0.00	Tp53bp1	0.00	Siae	0.03
Trappc3	0.00	Tlr9	0.00	Nucks	0.03
Trap1	0.00	Thumpd1	0.00	B4galt1	0.00
Tp53bp1	0.00	Thtpa	0.00	Cnbp	0.03
Tnks1bp1	0.00	Tenm4	0.00	Slc2a1	0.03
Tlr9	0.00	Tceb2	0.00	Pin1	0.03
Thumpd1	0.00	Tardbp	0.00	Gnpda1	0.03
Thtpa	0.00	Taldo1	0.00	Atp6v1d	0.03
Tenm4	0.00	Sub1	0.00	Adi1	0.03
Tceb2	0.00	Stip1	0.00	Ssb	0.03
Tars	0.00	St3gal	0.00	Qars	0.03
Tardbp	0.00	St13	0.00	Sf3b1	0.03
Taldo1	0.00	Srrt	0.00	Rps14	0.03
Sub1	0.00	Sri	0.00	Thtpa	0.03
Stip1	0.00	Sptbn1	0.00	Lars	0.03
St3gal	0.00	Snx5	0.00	Hnrnpdl	0.03
St13	0.00	Snx2	0.00	Hexim1	0.03
Ssb	0.00	Snrpb2	0.00	Sptbn1	0.03
Sri	0.00	Snrnp200	0.00	Mybbp1a	0.02
Snx5	0.00	Slf2	0.00	Bin1	0.02
Snx2	0.00	Slc2a1	0.00	Arfgap3	0.02
Snrpb2	0.00	Sirt1	0.00	LOXL4	0.02
Slc2a1	0.00	Sf3b1	0.00	Sec23b	0.02
Sirt1	0.00	Sf1	0.00	Plec	0.02
Sf3b1	0.00	Setdb1	0.00	Fhl1	0.02
Sf1	0.00	Set	0.00	Hdlbp	0.02
Sec24c	0.00	Sec24c	0.00	Ube2d3	0.02
Sec23b	0.00	Sec23b	0.00	Crim1	0.02
Sec22b	0.00	Sec22b	0.00	Hnrnpg	0.02
Sdk2	0.00	Sdc	0.00	Atp1a1	0.02

Selenocysteine lyase	Scly	0.000	0.000	0.059
Squamous cell carcinoma antigen recognized by	Sart3	0.000	0.000	0.036
Scaffold attachment factor B2	Safb2	0.000	0.000	0.001
Protein S100-A6	S100a6	0.000	0.022	0.171
Protein S100-A13	S100a13	0.000	0.000	0.152
RuvB-like 2	Ruvbl2	0.000	0.000	0.033
RuvB-like 1	Ruvbl1	0.000	0.002	0.148
Runt-related transcription factor 3	Runx3	0.000	0.000	0.007
40S ribosomal protein S27	Rps27	0.000	0.000	0.051
40S ribosomal protein S14	Rps14	0.000	0.000	0.028
40S ribosomal protein S13	Rps13	0.000	0.000	0.007
Regulation of nuclear pre-mRNA domain-contair	Rprd1b	0.000	0.003	0.142
60S ribosomal protein L7a	Rpl7a	0.000	0.000	0.063
60S ribosomal protein L6	Rpl6l	0.000	0.000	0.001
60S ribosomal protein L5	Rpl5	0.000	0.000	0.007
60S ribosomal protein L11	Rpl11	0.000	0.000	0.011
Replication protein A 14 kDa subunit	Rpa3	0.000	0.000	0.044
Regulator of differentiation 1	Rod1	0.000	0.000	0.002
RecName: Full	RecName: Full	0.000	0.032	0.437
Putative RNA-binding protein 19	Rbm19	0.000	0.000	0.002
GTP-binding nuclear protein Ran	Ran	0.000	0.000	0.005
Ras-related protein Rab-6A (Fragment)	Rab6a	0.000	0.010	0.010
Ras-related protein Rab-5C	Rab5c	0.000	0.000	0.005
Glutaminyl-tRNA synthetase	Qars	0.000	0.000	0.028
Partner of Y14 and mago	Pym1	0.000	0.000	0.005
Poly(U)-binding-splicing factor PUF60	Puf60	0.000	0.000	0.009
Poly(U)-binding-splicing factor PUF60	Puf60	0.000	0.000	0.047
Receptor-type tyrosine-protein phosphatase eta	Ptprj	0.000	0.003	0.000
Tyrosine-protein phosphatase non-receptor type	Ptpn23	0.000	0.000	0.002
Prostaglandin E synthase 3 (Fragment)	Ptges3	0.000	0.000	0.133
Proteasome assembly chaperone 4	Psmg4	0.000	0.000	0.059
Proteasome activator complex subunit 3	PSME3	0.000	0.008	0.124
26S proteasome non-ATPase regulatory subunit	Psmd7	0.000	0.003	0.053
26S protease regulatory subunit 8	Psmc5	0.000	0.000	0.015
Pre-mRNA-processing factor 19	Prpf19	0.000	0.000	0.061
Protein arginine N-methyltransferase 5	Prmt5	0.000	0.000	0.061
Thioredoxin-dependent peroxide reductase, mitc	Prdx3	0.000	0.000	0.008
Protein phosphatase 1F	Ppm1f	0.000	0.000	0.061
Peptidyl-prolyl cis-trans isomerase	Ppi	0.000	0.000	0.006
Pleckstrin-likey domain-containing family G men	Plekhg2	0.000	0.004	0.000
Pyruvate kinase	Pkm	0.000	0.000	0.005
Peptidyl-prolyl cis-trans isomerase NIMA-interac	Pin1	0.000	0.000	0.030
Phosphoacetylglucosamine mutase	Pgm3	0.000	0.000	0.041
6-phosphogluconolactonase	Pgls	0.000	0.000	0.211
Phosducin-like protein 3	Pdcl3	0.000	0.000	0.031
28 kDa heat-and acid-stable phosphoprotein	Pdap1	0.000	0.000	0.053
Protocadherin beta-6	Pcdhb6	0.000	0.001	0.000
Poly(RC)-binding protein 3	Pcbp3	0.000	0.000	0.006
Poly(RC)-binding protein 2	Pcbp2	0.000	0.000	0.003
Poly(RC)-binding protein 2	Pcbp2	0.000	0.003	0.000
Serine/threonine-protein kinase PAK 2	Pak2	0.000	0.002	0.004
Platelet-activating factor acetylhydrolase IB subu	Pafah1b1	0.000	0.000	0.070
Platelet-activating factor acetylhydrolase IB subu	Pafah1b1	0.000	0.009	0.139
Ubiquitin thioesterase OTUB1	Otub1	0.000	0.004	0.126
Nuclear ubiquitous casein and cyclin-dependent	Nucks	0.000	0.000	0.030
Nuclease domain-containing protein 1	Nucb1	0.000	0.001	0.144
Cytosolic purine 5'-nucleotidase	Nt5c2	0.000	0.000	0.046
Putative uncharacterized protein	Nrg1	0.000	0.000	0.036
Ribosyldihydronicotinamide dehydrogenase [qui	Nqo2	0.000	0.000	0.032
Non-POU domain-containing octamer-binding p	Nono	0.000	0.000	0.034
Neuroligin-3	Nlgn3	0.000	0.001	0.000
Nuclear receptor coactivator 1	Ncoa1	0.000	0.000	0.001
Alpha-soluble NSF attachment protein	Napa	0.000	0.000	0.020
UPF0160 protein MYG1, mitochondrial	Myg1	0.000	0.000	0.098
Myb-binding protein 1A	Mybbp1a	0.000	0.000	0.025
S-adenosylmethionine synthase	Mat	0.000	0.000	0.003
Microtubule-associated protein	Map	0.000	0.000	0.040
Cell growth-regulating nucleolar protein	Lyar	0.000	0.000	0.008
Lupus brain antigen 1 (Fragment)	Lba1	0.000	0.000	0.001
U6 snRNA-associated Sm-like protein LSm8	Lsm8	0.000	0.000	0.210
Leucine-rich repeat flightless-interacting protein	Lrrfip1	0.000	0.000	0.007
Lamin-L(I)	lmnl1	0.000	0.002	0.059
Lin-7-like B	Lin7b	0.000	0.000	0.010
L-lactate dehydrogenase A chain	Ldha	0.000	0.000	0.007
L-lactate dehydrogenase A chain	Ldha	0.000	0.000	0.010
Putative leucyl-tRNA synthetase, mitochondrial	Lars2	0.000	0.001	0.003
Keratin, type II cuticular Hb2	Krt82	0.000	0.004	0.000
Keratin, type II cytoskeletal 79	Krt79	0.000	0.000	0.002
Keratin, type II cytoskeletal 2 oral	Krt76	0.000	0.000	0.003
Importin subunit alpha	Kpna2	0.000	0.000	0.032
Kelch domain-containing protein 4	Klhl4	0.000	0.000	0.005
LisH domain and HEAT repeat-containing protei	Kiaa1468	0.000	0.001	0.001
Far upstream element-binding protein 2	Khsrp	0.000	0.009	0.069
Lysine--tRNA ligase	Kars	0.000	0.002	0.000
Pre-mRNA-splicing factor ISY1-like	Isy1	0.000	0.000	0.018
Isochorismatase domain-containing protein 1	Isoc1	0.000	0.000	0.145

Scly	0.00	Scly	0.00	Napa	0.02
Sart3	0.00	Sart3	0.00	Tsn	0.02
Safb2	0.00	Safb2	0.00	Eif4ebp1	0.02
S100a6	0.00	S100a13	0.00	Tubb5	0.02
S100a13	0.00	Ruvbl2	0.00	Rpl12	0.02
Ruvbl2	0.00	Runx3	0.00	Asns	0.02
Ruvbl1	0.00	Rps27	0.00	Isy1	0.02
Runx3	0.00	Rps2	0.00	Aldh9a1	0.02
Rps27	0.00	Rps14	0.00	Pdxk	0.02
Rps14	0.00	Rps13	0.00	Snrnp200	0.02
Rps13	0.00	Rplp1	0.00	Trappc3	0.02
Rprd1b	0.00	Rpl7a	0.00	Sec22b	0.02
Rpl7a	0.00	Rpl7	0.00	Epb41l2	0.02
Rpl6l	0.00	Rpl5	0.00	Pld3	0.02
Rpl5	0.00	Rpl27a	0.00	Psmc5	0.02
Rpl11	0.00	Rpl12	0.00	Atp1b3	0.01
Rpa3	0.00	Rpl11	0.00	Arpc1b	0.01
Rod1	0.00	Rpa3	0.00	Zc3h15	0.01
RecName: Full	0.00	Rpa2	0.00	C1qbp	0.01
Rbm19	0.00	Rod1	0.00	Bat1	0.01
Ran	0.00	Rnpep	0.00	Set	0.01
Rab6a	0.00	Rbm19	0.00	Adsl	0.01
Rab5c	0.00	Ran	0.00	Dctn1	0.01
Qars	0.00	Rab5c	0.00	Acp1	0.01
Pym1	0.00	Qars	0.00	Tlr9	0.01
Puf60	0.00	Pym1	0.00	Tceb2	0.01
Puf60	0.00	Puf60	0.00	Atxn7l3	0.01
Ptprj	0.00	Puf60	0.00	Ap1m1	0.01
Ptpn23	0.00	Ptpn23	0.00	Hspa2	0.01
Ptges3	0.00	Ptges3	0.00	Cul4b	0.01
Psmg4	0.00	Psmg4	0.00	Rpl11	0.01
PSME3	0.00	Psmc5	0.00	Layn	0.01
Psmd7	0.00	Prpf19	0.00	Gnb2	0.01
Psmc5	0.00	Protein C15orf2	0.00	Vbp1	0.01
Prpf19	0.00	Prmt5	0.00	Sept8	0.01
Prmt5	0.00	Prdx3	0.00	Tnks1bp1	0.01
Prdx3	0.00	Ppm1f	0.00	Baiap2	0.01
Ppm1f	0.00	Ppi	0.00	Lin7b	0.01
Ppi	0.00	Ppcs	0.00	Ldha	0.01
Plekhg2	0.00	Pld3	0.00	Farsa	0.01
Pkm	0.00	Pkm	0.00	Glb1	0.01
Pin1	0.00	Pin1	0.00	Stip1	0.01
Pgm3	0.00	Pgm3	0.00	Uncharacterized p	0.01
Pgls	0.00	Pgls	0.00	Rab6a	0.01
Pdcl3	0.00	Pfdn6	0.00	Cpox	0.01
Pdap1	0.00	Pdcl3	0.00	Puf60	0.01
Pcdhb6	0.00	Pdap1	0.00	Snx2	0.01
Pcbp3	0.00	Pcbp3	0.00	Lyar	0.01
Pcbp2	0.00	Pcbp2	0.00	Gmps	0.01
Pcbp2	0.00	Pafah1b1	0.00	Prdx3	0.01
Pak2	0.00	Nucks	0.00	Echdc1	0.01
Pafah1b1	0.00	Nt5c2	0.00	Ddx39	0.01
Pafah1b1	0.00	Nrg1	0.00	Lrrfip1	0.01
Otub1	0.00	Nrg1	0.00	Nkg2d4	0.01
Nucks	0.00	Nqo2	0.00	Gapdh	0.01
Nucb1	0.00	Nono	0.00	Runx3	0.01
Nt5c2	0.00	Ncoa1	0.00	Dlg1	0.01
Nrg1	0.00	Napa	0.00	Rps13	0.01
Nqo2	0.00	Myo18a	0.00	Arfip2	0.01
Nono	0.00	Myh10	0.00	Rpl5	0.01
Nlgn3	0.00	Myg1	0.00	Ldha	0.01
Ncoa1	0.00	Mybbp1a	0.00	Taldo1	0.01
Napa	0.00	Mdp1	0.00	Uncharacterized p	0.01
Myg1	0.00	Mdh1	0.00	Vwa5a	0.01
Mybbp1a	0.00	Mat	0.00	Ppi	0.01
Mat	0.00	Masp1	0.00	Hmgb1	0.01
Map	0.00	Map	0.00	Snrpb2	0.01
Lyar	0.00	Lyar	0.00	Fer	0.01
Lupus brain antige	0.00	Lupus brain antige	0.00	Pcbp3	0.01
Lsm8	0.00	Ltn1	0.00	Gspt2	0.01
Lrrfip1	0.00	Lsm8	0.00	Myh10	0.01
lmnl1	0.00	Lrrfip1	0.00	Rab5c	0.01
Lin7b	0.00	Lin7b	0.00	Klhl4	0.01
Ldha	0.00	Ldha	0.00	Sept5	0.01
Ldha	0.00	Ldha	0.00	Hist1h4	0.01
Lars2	0.00	Lars	0.00	Srrt	0.01
Krt82	0.00	Krt8	0.00	Hspa8	0.01
Krt79	0.00	Krt79	0.00	Pkm	0.00
Krt76	0.00	Krt76	0.00	Pym1	0.00
Kpna2	0.00	Kpna2	0.00	Hdac	0.00
Klhl4	0.00	Klhl4	0.00	Ran	0.00
Kiaa1468	0.00	Isy1	0.00	Krt2	0.00
Khsrp	0.00	Isoc1	0.00	Pak2	0.00
Kars	0.00	Iqgap2	0.00	Ddx5	0.00
Isy1	0.00	Impdh2	0.00	Cab39	0.00
Isoc1	0.00	Iah1	0.00	H2-L	0.00

Ras GTPase-activating-like protein IQGAP2	Iqgap2	0.000	0.000	0.001
Ras GTPase-activating protein-binding protein 2	Iqgap1	0.000	0.005	0.083
Inosine-5'-monophosphate dehydrogenase 2	Impdh2	0.000	0.000	0.173
E3 ubiquitin-protein ligase HUWE1 (Fragment)	Huwe1	0.000	0.001	0.000
Serine protease HTRA2, mitochondrial	Htra2	0.000	0.000	0.066
Heat shock 70 kDa protein 1L	Hspa1l	0.000	0.000	0.003
Heat shock factor protein 4	Hsf4	0.000	0.000	0.002
Heterogeneous nuclear ribonucleoprotein L-like	Hnrnpll	0.000	0.000	0.073
Heterogeneous nuclear ribonucleoprotein D-like	Hnrnpdl	0.000	0.000	0.028
Heterogeneous nuclear ribonucleoprotein A3-lik	Hnrnpa3	0.000	0.000	0.143
Heme oxygenase 1	Hmox1	0.000	0.000	0.324
High mobility group protein B1	Hmgb1	0.000	0.000	0.006
Histone H4	Hist1h4	0.000	0.005	0.005
Histone H2B	Hist1h2b	0.000	0.008	0.000
Protein HEXIM1	Hexim1	0.000	0.000	0.027
Vigilin	Hdlbp	0.000	0.000	0.022
Histone deacetylase	Hdac	0.000	0.000	0.005
Histone H3.3	H3f3a	0.000	0.000	0.003
H-2 class I histocompatibility antigen, L-D alpha	H2-L	0.000	0.000	0.003
Glutathione S-transferase theta-2	Gstt2	0.000	0.000	0.035
Eukaryotic peptide chain release factor GTP-bind	Gspt2	0.000	0.000	0.005
Growth factor receptor-bound protein 2	Grb2	0.000	0.000	0.132
Glucosamine-6-phosphate isomerase 1 (Fragmen	Gnpda1	0.000	0.004	0.030
GMP synthase [glutamine-hydrolyzing]	Gmps	0.000	0.000	0.008
Glia maturation factor beta	Gmfb	0.000	0.000	0.068
Glyceraldehyde-3-phosphate dehydrogenase	Gapdh	0.000	0.005	0.000
Gamma-aminobutyric acid receptor-associated p	Gabarapl2	0.000	0.000	0.099
RNA-binding protein FUS	Fus	0.000	0.000	0.036
Far upstream element-binding protein 1	Fubp1	0.000	0.012	0.111
Filamin-C	Flnc	0.000	0.000	0.001
Peptidyl-prolyl cis-trans isomerase A	Fkbp1a	0.000	0.010	0.000
Four and a half LIM domains protein 1	Fhl1	0.000	0.000	0.023
Fermitin family-like 2	FERMT2	0.000	0.006	0.070
Ferritin	Fer	0.000	0.000	0.006
Phenylalanyl-tRNA synthetase alpha chain	Farsa	0.000	0.000	0.010
Protein FAM98B	Fam98b	0.000	0.000	0.034
Fumarylacetoacetase	Fah	0.000	0.019	0.098
Eukaryotic peptide chain release factor subunit 1	ETF1	0.000	0.000	0.121
Band 4.1-like protein 2	Epb41l2	0.000	0.001	0.015
Eukaryotic translation initiation factor 4E-binding	Eif4ebp1	0.000	0.000	0.019
Eukaryotic initiation factor 4A-II	Eif4a2	0.000	0.000	0.044
Eukaryotic translation initiation factor 3 subunit	Eif3e	0.000	0.000	0.097
Eukaryotic translation initiation factor 3 subunit	Eif3c	0.000	0.000	0.036
Eukaryotic translation elongation factor 1 epsilon	Eef1e1	0.000	0.000	0.090
Elongation factor 1-alpha	Eef1a1	0.000	0.000	0.003
Enoyl-CoA hydratase domain-containing protein	Echdc1	0.000	0.000	0.008
Protein Dr1	Dr1	0.000	0.000	0.042
2'-deoxynucleoside 5'-phosphate N-hydrolase 1	Dnph1	0.000	0.000	0.097
Dynamin	Dnm	0.000	0.000	0.036
DnaJ-like subfamily C member 8	Dnajc8	0.000	0.000	0.083
Disks large-like 1	Dlg1	0.000	0.000	0.007
Deoxyhypusine synthase	Dhps	0.000	0.000	0.104
Desmin	Des	0.000	0.003	0.000
Putative deoxyribose-phosphate aldolase	Dera	0.000	0.000	0.034
ATP-dependent RNA helicase DDX39	Ddx39	0.000	0.000	0.008
Nucleolar RNA helicase 2	Ddx21	0.000	0.000	0.003
dCTP pyrophosphatase 1	Dctpp1	0.000	0.000	0.048
Dynactin subunit 1	Dctn1	0.000	0.000	0.013
Cytoplasmic FMR1-interacting protein 1	Cyfip1	0.000	0.000	0.002
Cytochrome c	Cycs	0.000	0.000	0.062
Cullin-4B (Fragment)	Cul4b	0.000	0.000	0.011
Casein kinase II subunit beta	Csnk2b	0.000	0.000	0.079
Beta/gamma crystallin domain-containing protei	Crybg3	0.000	0.001	0.000
Cysteine-rich protein 2	Crip2	0.000	0.000	0.090
Coproporphyrinogen-III oxidase, mitochondrial	Cpox	0.000	0.000	0.009
Coatomer subunit alpha	Copa	0.000	0.000	0.049
CB1 cannabinoid receptor-interacting protein 1	Cnrip1	0.000	0.000	0.046
Cellular nucleic acid-binding protein	Cnbp	0.000	0.000	0.030
Clathrin light chain A	Clta	0.000	0.000	0.098
CAP-Gly domain-containing linker protein 2	Clip2	0.000	0.000	0.001
Cysteine and histidine-rich domain-containing p	Chordc1	0.000	0.000	0.086
Chromodomain-helicase-DNA-binding protein 6	Chd6	0.000	0.001	0.001
Calmodulin	Calm	0.000	0.000	0.104
Voltage-dependent N-type calcium channel subu	Cacna1b	0.000	0.001	0.000
Calcium-binding protein 39	Cab39	0.000	0.000	0.004
Complement component C7	C7	0.000	0.001	0.003
Complement component 1 Q subcomponent-bin	C1qbp	0.000	0.000	0.015
Breast cancer type 2 susceptibility protein-like	Brca2	0.000	0.001	0.000
Tyrosine-protein kinase BLK	Blk	0.000	0.000	0.002
Myc box-dependent-interacting protein 1 (Fragm	Bin1	0.000	0.000	0.025
BH3-interacting domain death agonist	Bid	0.000	0.000	0.109
Spliceosome RNA helicase BAT1	Bat1	0.000	0.000	0.014
V-type proton ATPase subunit E 1	Atp6v1e1	0.000	0.000	0.049
V-type proton ATPase subunit D	Atp6v1d	0.000	0.000	0.029
V-type proton ATPase subunit B, brain isoform (	Atp6v1b2	0.000	0.000	0.049
Sodium/potassium-transporting ATPase subunit	Atp1b3	0.000	0.000	0.015

Iqgap2	0.00	Htra2	0.00	Adamts18	0.00
Iqgap1	0.00	Hspa8	0.00	Krt76	0.00
Impdh2	0.00	Hspa2	0.00	Hspa1l	0.00
Huwe1	0.00	Hspa1l	0.00	Pcbp2	0.00
Htra2	0.00	Hsf4	0.00	Ptx3	0.00
Hspa1l	0.00	Hp1g	0.00	C7	0.00
Hsf4	0.00	Hnrnpll	0.00	St3gal	0.00
Hnrnpll	0.00	Hnrnpdl	0.00	Ttc15	0.00
Hnrnpdl	0.00	Hnrnpa3	0.00	Ddx21	0.00
Hnrnpa3	0.00	Hmox1	0.00	H3f3a	0.00
Hmox1	0.00	Hmgb1	0.00	Ap3b1	0.00
Hmgb1	0.00	Hist3h3	0.00	Mat	0.00
Hist1h4	0.00	Hexim1	0.00	Lpl	0.00
Hist1h2b	0.00	Hdlbp	0.00	Ces4	0.00
Hexim1	0.00	Hdac	0.00	Lars2	0.00
Hdlbp	0.00	H3f3a	0.00	Eef1a1	0.00
Hdac	0.00	H2-L	0.00	Ubiquitin	0.00
H3f3a	0.00	Gstt2	0.00	Vim	0.00
H2-L	0.00	Gspt2	0.00	Tubb4	0.00
Gstt2	0.00	Grb2	0.00	Hsf4	0.00
Gspt2	0.00	Gnb2	0.00	Col7a1	0.00
Grb2	0.00	Gmps	0.00	Rbm19	0.00
Gnpda1	0.00	Gmfb	0.00	Blk	0.00
Gmps	0.00	GLT25D1	0.00	SGSH	0.00
Gmfb	0.00	Gart	0.00	Rod1	0.00
Gapdh	0.00	Gapdh	0.00	Ptpn23	0.00
Gabarapl2	0.00	Gapdh	0.00	Krt79	0.00
Fus	0.00	Gabarapl2	0.00	FAT1	0.00
Fubp1	0.00	Fus	0.00	Os9	0.00
Flnc	0.00	Fkbp14	0.00	Adam10	0.00
Fkbp1a	0.00	Fhl1	0.00	0.000	0.00
Fhl1	0.00	Fh	0.00	Cyfip1	0.00
FERMT2	0.00	Fer	0.00	Ythdc2	0.00
Fer	0.00	Fbn1	0.00	Myo18a	0.00
Farsa	0.00	Farsa	0.00	Brd2	0.00
Fam98b	0.00	Fam98b	0.00	Trap1	0.00
Fah	0.00	ETF1	0.00	Ncoa1	0.00
ETF1	0.00	Eif4ebp1	0.00	Kiaa1468	0.00
Epb41l2	0.00	Eif4a2	0.00	Tubb3	0.00
Eif4ebp1	0.00	Eif3e	0.00	H2-D1	0.00
Eif4a2	0.00	Eif3c	0.00	Actn3	0.00
Eif3e	0.00	Eef1e1	0.00	Rpl6l	0.00
Eif3c	0.00	Eef1a1	0.00	Tp53bp1	0.00
Eef1e1	0.00	Eef1a1	0.00	Clip2	0.00
Eef1a1	0.00	Echdc1	0.00	Lupus brain antige	0.00
Echdc1	0.00	Dr1	0.00	Chd6	0.00
Dr1	0.00	Dnph1	0.00	Tdrd7	0.00
Dnph1	0.00	Dnm	0.00	Acacb	0.00
Dnm	0.00	Dnajc8	0.00	Slf2	0.00
Dnajc8	0.00	Dlg1	0.00	Safb2	0.00
Dlg1	0.00	Dhps	0.00	Flnc	0.00
Dhps	0.00	Dera	0.00	Tenm4	0.00
Des	0.00	Ddx39	0.00	Iqgap2	0.00
Dera	0.00	Ddx21	0.00	Brca2	0.00
Ddx39	0.00	Dctpp1	0.00	Golgb1	0.00
Ddx21	0.00	Dctn1	0.00	Huwe1	0.00
Dctpp1	0.00	Dbnl	0.00	Ntn4	0.00
Dctn1	0.00	Cyfip1	0.00	Zeb2	0.00
Cyfip1	0.00	Cycs	0.00	Tuba8	0.00
Cycs	0.00	Cul4b	0.00	St8sia4	0.00
Cul4b	0.00	Csnk2b	0.00	Setdb1	0.00
Csnk2b	0.00	Crip2	0.00	Sepn	0.00
Crybg3	0.00	Cpox	0.00	Sdk2	0.00
Crip2	0.00	Copa	0.00	Sdc	0.00
Cpox	0.00	Cnrip1	0.00	Rps2	0.00
Copa	0.00	Cnbp	0.00	Rplp1	0.00
Cnrip1	0.00	Clta	0.00	Rpl7	0.00
Cnbp	0.00	Clip2	0.00	Ptprj	0.00
Clta	0.00	Chordc1	0.00	Protein C15orf2	0.00
Clip2	0.00	Cg050	0.00	Plekhg2	0.00
Chordc1	0.00	Ces4	0.00	Pcdhb6	0.00
Chd6	0.00	Calm	0.00	Pcbp2	0.00
Calm	0.00	Cadps2	0.00	Otub1	0.00
Cacna1b	0.00	Cab39	0.00	Nrg1	0.00
Cab39	0.00	C1qbp	0.00	Nlgn3	0.00
C7	0.00	Blk	0.00	Masp1	0.00
C1qbp	0.00	Bin1	0.00	Ltn1	0.00
Brca2	0.00	Bid	0.00	LOC100689010	0.00
Blk	0.00	Bat1	0.00	Krt82	0.00
Bin1	0.00	Baiap2	0.00	Krt8	0.00
Bid	0.00	Bai2	0.00	Kars	0.00
Bat1	0.00	Atp6v1e1	0.00	Il1rl1	0.00
Atp6v1e1	0.00	Atp6v1d	0.00	Hist3h3	0.00
Atp6v1d	0.00	Atp6v1b2	0.00	Hist1h2b	0.00
Atp6v1b2	0.00	Atp1b3	0.00	H2-K1	0.00
Atp1b3	0.00	Atp1a2	0.00	GLT25D1	0.00

Asparagine synthetase [glutamine-hydrolyzing]	Asns	0.000	0.002	0.019
Actin-related protein 2/3 complex subunit 1B	Arpc1b	0.000	0.000	0.015
Arfaptin-2	Arfip2	0.000	0.000	0.007
ADP-ribosylation factor GTPase-activating protei	Arfgap3	0.000	0.000	0.024
Apolipoprotein B-100	Apob	0.000	0.001	0.000
AP-3 complex subunit beta-1	Ap3b1	0.000	0.001	0.003
AP-1 complex subunit mu-1	Ap1m1	0.000	0.000	0.012
AP-1 complex subunit gamma-1	Ap1g1	0.000	0.000	0.040
Mutant adenylosuccinate lyase	Adsl	0.000	0.000	0.013
[Protein ADP-ribosylarginine] hydrolase	Adprh	0.000	0.000	0.064
1,2-dihydroxy-3-keto-5-methylthiopentene dioxy	Adi1	0.000	0.000	0.029
Adenosine deaminase	Ada	0.000	0.000	0.076
Actin-related protein 2	Actr2	0.000	0.000	0.035
Alpha-actinin-3	Actn3	0.000	0.000	0.001
Low molecular weight phosphotyrosine protein	Acp1	0.000	0.000	0.013
ATP-citrate synthase	Acly	0.000	0.000	0.084
Abhydrolase domain-containing protein 14B	Abhd14b	0.000	0.000	0.121
Septin-8	Sept8	0.000	0.000	0.011
Septin-5	Sept5	0.000	0.000	0.005

Asns	0.00	Arpc1b	0.00	Gart	0.00
Arpc1b	0.00	Arfip2	0.00	Gapdh	0.00
Arfip2	0.00	Arfgap3	0.00	Gapdh	0.00
Arfgap3	0.00	Ap1m1	0.00	Fkbp1a	0.00
Apob	0.00	Ap1g1	0.00	Fbn1	0.00
Ap3b1	0.00	Aldh9a1	0.00	Efemp2	0.00
Ap1m1	0.00	Adsl	0.00	Eef1a1	0.00
Ap1g1	0.00	Adprh	0.00	Des	0.00
Adsl	0.00	Adi1	0.00	Crybg3	0.00
Adprh	0.00	Ada	0.00	COL6A1	0.00
Adi1	0.00	Actr2	0.00	Cntn1	0.00
Ada	0.00	Actn3	0.00	Ccl7	0.00
Actr2	0.00	Acp1	0.00	Cadps2	0.00
Actn3	0.00	Acly	0.00	Cacna1b	0.00
Acp1	0.00	Acacb	0.00	Bai2	0.00
Acly	0.00	Abhd14b	0.00	Atrx	0.00
Abhd14b	0.00	Sept8	0.00	Atp1a2	0.00
Sept8	0.00	Sept5	0.00	Arf4	0.00
Sept5	0.00	0.000	0.00	Apob	0.00

Day3_Top 30_CC				
Term	Count	%	P-Value	Benjamini
extracellular region	20	74.1	2.60E-14	1.90E-12
extracellular region part	14	51.9	2.40E-11	9.00E-10
proteinaceous extracellular matrix	7	25.9	5.40E-06	1.30E-04
extracellular matrix	7	25.9	6.80E-06	1.30E-04
extracellular space	8	29.6	1.00E-05	1.50E-04
lysosome	5	18.5	1.60E-04	2.00E-03
lytic vacuole	5	18.5	1.70E-04	1.70E-03
basement membrane	4	14.8	2.00E-04	1.90E-03
vacuole	5	18.5	2.70E-04	2.20E-03
extracellular matrix part	4	14.8	4.00E-04	2.90E-03
cell surface	5	18.5	1.20E-03	8.30E-03
external side of plasma membrane	4	14.8	4.00E-03	2.50E-02

Day5_Top 30_CC				
Term	Count	%	P-Value	Benjamini
extracellular region	20	74.1	2.60E-14	2.40E-12
extracellular region part	13	48.1	4.90E-10	2.20E-08
proteinaceous extracellular matrix	6	22.2	8.70E-05	2.60E-03
extracellular matrix	6	22.2	1.00E-04	2.40E-03
extracellular space	7	25.9	1.10E-04	2.10E-03
basement membrane	4	14.8	2.00E-04	3.00E-03
extracellular matrix part	4	14.8	4.00E-04	5.10E-03
pigment granule	3	11.1	7.90E-03	8.60E-02
melanosome	3	11.1	7.90E-03	8.60E-02
cell surface	4	14.8	1.20E-02	1.10E-01
lysosome	3	11.1	3.20E-02	2.50E-01
lytic vacuole	3	11.1	3.20E-02	2.40E-01
vacuole	3	11.1	4.10E-02	2.70E-01
external side of plasma membrane	3	11.1	4.10E-02	2.60E-01

Day8_Top 30_CC				
Term	Count	%	P-Value	Benjamini
extracellular region	13	59.1	4.80E-08	3.50E-06
extracellular region part	8	36.4	1.90E-05	7.20E-04
pigment granule	4	18.2	1.40E-04	3.30E-03
melanosome	4	18.2	1.40E-04	3.30E-03
proteinaceous extracellular matrix	5	22.7	3.70E-04	6.90E-03
extracellular matrix	5	22.7	4.40E-04	6.40E-03
lysosome	4	18.2	1.20E-03	1.40E-02
lytic vacuole	4	18.2	1.20E-03	1.30E-02
vacuole	4	18.2	1.70E-03	1.60E-02
basement membrane	3	13.6	3.40E-03	2.80E-02
extracellular matrix part	3	13.6	5.30E-03	3.90E-02
cell surface	4	18.2	5.40E-03	3.60E-02
cytoplasmic membrane-bounded vesicle	4	18.2	1.30E-02	7.50E-02
membrane-bounded vesicle	4	18.2	1.30E-02	7.20E-02
cytoplasmic vesicle	4	18.2	2.20E-02	1.10E-01
extracellular space	4	18.2	2.20E-02	1.00E-01
vesicle	4	18.2	2.30E-02	1.00E-01
cytoplasmic part	10	45.5	4.00E-02	1.60E-01

Clustering analysis				
Cluster 1	Protein ID	day3	day5	day8
	Hmox1	0.000	0.000	0.324
	St13	0.000	0.000	0.282
	Pgls	0.000	0.000	0.211
	Lsm8	0.000	0.000	0.210
	Impdh2	0.000	0.000	0.173
	S100a13	0.000	0.000	0.152

Sub1	0.000	0.000	0.145
Isoc1	0.000	0.000	0.145
Hnrnpa3	0.000	0.000	0.143
Ptges3	0.000	0.000	0.133
Grb2	0.000	0.000	0.132
Abhd14b	0.000	0.000	0.121
ETF1	0.000	0.000	0.121
Tardbp	0.000	0.000	0.111
Bid	0.000	0.000	0.109
Calm	0.000	0.000	0.104
Dhps	0.000	0.000	0.104
Gabarapl2	0.000	0.000	0.099
Clta	0.000	0.000	0.098
Myg1	0.000	0.000	0.098
Eif3e	0.000	0.000	0.097
Dnph1	0.000	0.000	0.097
Sirt1	0.000	0.000	0.092
Vbp1	0.000	0.000	0.091
Crip2	0.000	0.000	0.090
Eef1e1	0.000	0.000	0.090
Chordc1	0.000	0.000	0.086
Sri	0.000	0.000	0.086
Acly	0.000	0.000	0.084
Dnajc8	0.000	0.000	0.083
Csnk2b	0.000	0.000	0.079
Ada	0.000	0.000	0.076
Hnrnpll	0.000	0.000	0.073
Pafah1b1	0.000	0.000	0.070
Gmfb	0.000	0.000	0.068
Uso1	0.000	0.000	0.067
Htra2	0.000	0.000	0.066
Adprh	0.000	0.000	0.064
Rpl7a	0.000	0.000	0.063
Cycs	0.000	0.000	0.062
Prpf19	0.000	0.000	0.061
Ppm1f	0.000	0.000	0.061
Prmt5	0.000	0.000	0.061
Scly	0.000	0.000	0.059
Psmg4	0.000	0.000	0.059
Zpr1	0.000	0.000	0.059
Rnpep	0.000	0.000	0.059
Pdap1	0.000	0.000	0.053
Sf1	0.000	0.000	0.053
Rps27	0.000	0.000	0.051
Atp6v1b2	0.000	0.000	0.049
Atp6v1e1	0.000	0.000	0.049
Copa	0.000	0.000	0.049
Dctpp1	0.000	0.000	0.048
Puf60	0.000	0.000	0.047
Cnrip1	0.000	0.000	0.046
Nt5c2	0.000	0.000	0.046
Eif4a2	0.000	0.000	0.044
Snx5	0.000	0.000	0.044
Rpa3	0.000	0.000	0.044
Dr1	0.000	0.000	0.042
Pgm3	0.000	0.000	0.041
Ap1g1	0.000	0.000	0.040
Map	0.000	0.000	0.040
Dbnl	0.000	0.000	0.040
Eif3c	0.000	0.000	0.036
Fus	0.000	0.000	0.036
Nrg1	0.000	0.000	0.036
Sec24c	0.000	0.000	0.036
Dnm	0.000	0.000	0.036
Sart3	0.000	0.000	0.036
Gstt2	0.000	0.000	0.035
Actr2	0.000	0.000	0.035
Nono	0.000	0.000	0.034
Dera	0.000	0.000	0.034
Fam98b	0.000	0.000	0.034
Trip10	0.000	0.000	0.033
Vps26a	0.000	0.000	0.033
Ruvbl2	0.000	0.000	0.033
Nqo2	0.000	0.000	0.032
Thumpd1	0.000	0.000	0.032
Kpna2	0.000	0.000	0.032
Pdcl3	0.000	0.000	0.031
Nucks	0.000	0.000	0.030
Cnbp	0.000	0.000	0.030
Slc2a1	0.000	0.000	0.030
Pin1	0.000	0.000	0.030
Atp6v1d	0.000	0.000	0.029
Adi1	0.000	0.000	0.029
Qars	0.000	0.000	0.028
Sf3b1	0.000	0.000	0.028
Rps14	0.000	0.000	0.028

Thtpa	0.000	0.000	0.028
Lars	0.000	0.000	0.028
Hnrnpdl	0.000	0.000	0.028
Hexim1	0.000	0.000	0.027
Sptbn1	0.000	0.000	0.026
Mybbp1a	0.000	0.000	0.025
Bin1	0.000	0.000	0.025
Arfgap3	0.000	0.000	0.024
Sec23b	0.000	0.000	0.024
Fhl1	0.000	0.000	0.023
Hdlbp	0.000	0.000	0.022
Ube2d3	0.000	0.000	0.022
Napa	0.000	0.000	0.020
Tsn	0.000	0.000	0.019
Eif4ebp1	0.000	0.000	0.019
Isy1	0.000	0.000	0.018
Aldh9a1	0.000	0.000	0.017
Snrnp200	0.000	0.000	0.017
Trappc3	0.000	0.000	0.017
Sec22b	0.000	0.000	0.016
Pld3	0.000	0.000	0.015
Psmc5	0.000	0.000	0.015
Atp1b3	0.000	0.000	0.015
Arpc1b	0.000	0.000	0.015
Zc3h15	0.000	0.000	0.015
C1qbp	0.000	0.000	0.015
Bat1	0.000	0.000	0.014
Adsl	0.000	0.000	0.013
Dctn1	0.000	0.000	0.013
Acp1	0.000	0.000	0.013
Tlr9	0.000	0.000	0.013
Tceb2	0.000	0.000	0.012
Ap1m1	0.000	0.000	0.012
Hspa2	0.000	0.000	0.012
Cul4b	0.000	0.000	0.011
Rpl11	0.000	0.000	0.011
Vbp1	0.000	0.000	0.011
Sept8	0.000	0.000	0.011
Baiap2	0.000	0.000	0.010
Lin7b	0.000	0.000	0.010
Ldha	0.000	0.000	0.010
Farsa	0.000	0.000	0.010
Stip1	0.000	0.000	0.010
Uncharacterized	0.000	0.000	0.010
Cpox	0.000	0.000	0.009
Puf60	0.000	0.000	0.009
Snx2	0.000	0.000	0.008
Lyar	0.000	0.000	0.008
Gmps	0.000	0.000	0.008
Prdx3	0.000	0.000	0.008
Echdc1	0.000	0.000	0.008
Ddx39	0.000	0.000	0.008
Lrrfip1	0.000	0.000	0.007
Runx3	0.000	0.000	0.007
Dlg1	0.000	0.000	0.007
Rps13	0.000	0.000	0.007
Arfip2	0.000	0.000	0.007
Rpl5	0.000	0.000	0.007
Ldha	0.000	0.000	0.007
Taldo1	0.000	0.000	0.007
Uncharacterized	0.000	0.000	0.006
Ppi	0.000	0.000	0.006
Hmgb1	0.000	0.000	0.006
Snrpb2	0.000	0.000	0.006
Fer	0.000	0.000	0.006
Pcbp3	0.000	0.000	0.006
Gspt2	0.000	0.000	0.005
Myh10	0.000	0.000	0.005
Rab5c	0.000	0.000	0.005
Klhl4	0.000	0.000	0.005
Sept5	0.000	0.000	0.005
Srrt	0.000	0.000	0.005
Pkm	0.000	0.000	0.005
Pym1	0.000	0.000	0.005
Hdac	0.000	0.000	0.005
Ran	0.000	0.000	0.005
Cab39	0.000	0.000	0.004
H2-L	0.000	0.000	0.003
Krt76	0.000	0.000	0.003
Hspa1l	0.000	0.000	0.003
Pcbp2	0.000	0.000	0.003
St3gal	0.000	0.000	0.003
Ttc15	0.000	0.000	0.003
Ddx21	0.000	0.000	0.003
H3f3a	0.000	0.000	0.003
Mat	0.000	0.000	0.003

	Eef1a1	0.000	0.000	0.003
	Ubiquitin	0.000	0.000	0.002
	Vim	0.000	0.000	0.002
	Tubb4	0.000	0.000	0.002
	Hsf4	0.000	0.000	0.002
	Rbm19	0.000	0.000	0.002
	Blk	0.000	0.000	0.002
	Rod1	0.000	0.000	0.002
	Ptpn23	0.000	0.000	0.002
	Krt79	0.000	0.000	0.002
	Tubb4	0.000	0.000	0.002
	Cyfip1	0.000	0.000	0.002
	Myo18a	0.000	0.000	0.002
	Trap1	0.000	0.000	0.002
	Ncoa1	0.000	0.000	0.001
	Tubb3	0.000	0.000	0.001
	Actn3	0.000	0.000	0.001
	Rpl6l	0.000	0.000	0.001
	Tp53bp1	0.000	0.000	0.001
	Clip2	0.000	0.000	0.001
	Lupus brain antic	0.000	0.000	0.001
	Acacb	0.000	0.000	0.001
	Slf2	0.000	0.000	0.001
	Safb2	0.000	0.000	0.001
	Flnc	0.000	0.000	0.001
	Tenm4	0.000	0.000	0.001
	Iqgap2	0.000	0.000	0.001
	Fbn1	0.000	0.000	0.000
	Atp1a2	0.000	0.000	0.000
	Gart	0.000	0.000	0.000
	Ltn1	0.000	0.000	0.000
	Bai2	0.000	0.000	0.000
	Masp1	0.000	0.000	0.000
	Setdb1	0.000	0.000	0.000
	Krt8	0.000	0.000	0.000
	Eef1a1	0.000	0.000	0.000
	C15orf2	0.000	0.000	0.000
Cluster 2	Protein ID	day3	day5	day8
	Mdh1	0.001	0.000	0.234
	Pfdn6	0.002	0.000	0.251
	Ppcs	0.001	0.000	0.088
	Nucb1	0.000	0.001	0.144
	Fh	0.001	0.000	0.094
	Atp6v1a	0.000	0.002	0.141
	Iah1	0.001	0.000	0.121
	Psmd2	0.001	0.001	0.046
	Fkbp14	0.002	0.000	0.131
	Ruvbl1	0.000	0.002	0.148
	Sf3a1	0.000	0.001	0.057
	Mdp1	0.001	0.000	0.065
	Nap1l4	0.001	0.003	0.092
	Rprd1b	0.000	0.003	0.142
	Cg050	0.001	0.000	0.040
	Plaa	0.000	0.001	0.037
	Fgfr	0.001	0.003	0.059
	Kcnab2	0.001	0.003	0.089
	Rpl27a	0.001	0.000	0.042
	Nudt5	0.001	0.004	0.113
	Otub1	0.000	0.004	0.126
	Hp1g	0.003	0.000	0.099
	Tars	0.000	0.004	0.132
	lmnl1	0.000	0.002	0.059
	Strap	0.001	0.003	0.069
	Blmh	0.000	0.004	0.102
	Vps35	0.000	0.001	0.038
	Fasn	0.005	0.012	0.188
	Bag3	0.000	0.004	0.095
	Rpa2	0.002	0.000	0.039
	Ap1b1	0.001	0.004	0.051
	Dars	0.000	0.004	0.077
	Tbca	0.003	0.028	0.538
	Txndc17	0.001	0.006	0.103
	Pdlim1	0.001	0.004	0.071
	Glrx3	0.001	0.012	0.192
	Cyb5	0.001	0.013	0.211
	Psmd7	0.000	0.003	0.053
	Cltb	0.001	0.006	0.086
	Gnb2	0.001	0.000	0.011
	Tnks1bp1	0.000	0.001	0.010
	Iqgap1	0.000	0.005	0.083
	Set	0.001	0.000	0.014
	PSME3	0.000	0.008	0.124
	Arcn1	0.000	0.007	0.104
	Yars	0.002	0.014	0.197
	Pafah1b1	0.000	0.009	0.139
	Epb41l2	0.000	0.001	0.015

RecName: Full	0.000	0.032	0.437
Lap3	0.004	0.025	0.282
FERMT2	0.000	0.006	0.070
Prkar1a	0.001	0.008	0.081
Hspa8	0.001	0.000	0.005
Uap1	0.006	0.016	0.125
Rpl12	0.002	0.000	0.019
Pdia4	0.017	0.064	0.552
Asns	0.000	0.002	0.019
Ssb	0.000	0.003	0.028
Pacsin2	0.004	0.011	0.081
Ak2	0.027	0.072	0.468
Nit2	0.001	0.019	0.177
Fubp1	0.000	0.012	0.111
Thop1	0.008	0.029	0.195
Chid1	0.001	0.010	0.080
Atp1a1	0.000	0.003	0.020
Adamts18	0.001	0.001	0.003
S100a6	0.000	0.022	0.171
Gnpda1	0.000	0.004	0.030
Khsrp	0.000	0.009	0.069
Fkbp9	0.013	0.035	0.166
Psma7	0.027	0.089	0.461
Hnrnpa2b1	0.010	0.060	0.353
Gapdh	0.001	0.000	0.007
Ranbp1	0.010	0.033	0.145
Gss	0.016	0.094	0.457
Flna	0.003	0.035	0.174
Ast	0.022	0.096	0.425
Nsfl1c	0.018	0.075	0.316
Eif2s2	0.004	0.019	0.082
Vwa5a	0.000	0.001	0.006
Fah	0.000	0.019	0.098
Hsp90b1	0.046	0.165	0.643
Ldh	0.031	0.094	0.345
6Pgd	0.032	0.131	0.516
Pcbp1	0.006	0.028	0.113
PSAT	0.040	0.192	0.758
Atxn7l3	0.001	0.003	0.012
Pdxk	0.003	0.006	0.017
Pkm	0.111	0.399	1.411
Tkt	0.043	0.208	0.785
Akr1b1	0.036	0.176	0.668
Calr	0.003	0.017	0.066
Ube2n	0.013	0.075	0.288
Ywhae	0.175	0.778	2.821
Prkcsh	0.014	0.044	0.141
Gsto1	0.023	0.074	0.244
Eef1g	0.029	0.103	0.343
Hsp90aa1	0.016	0.073	0.253
Prdx1	0.114	0.612	2.176
Nudc	0.026	0.098	0.323
Arhgdia	0.032	0.253	0.939
Ctsz	0.139	0.302	0.803
Gstp	0.040	0.194	0.665
Hspa5	0.077	0.302	0.986
C7	0.000	0.001	0.003
VAT1L	0.018	0.158	0.572
Clns1a	0.001	0.021	0.081
Pgk	0.045	0.237	0.794
Tmpo	0.006	0.032	0.106
Capg	0.013	0.111	0.383
Ces4	0.001	0.000	0.003
Sort1	0.003	0.017	0.054
Pdi	0.045	0.133	0.368
Cdc42	0.025	0.099	0.289
Agrn	0.033	0.052	0.099
Cd63	0.012	0.035	0.092
Naca	0.022	0.084	0.235
Hsp90ab1	0.054	0.210	0.581
Rplp2	0.064	0.375	1.076
Actn1	0.021	0.118	0.336
Txnrd1	0.022	0.111	0.299
Rcn3	0.026	0.099	0.251
Ppa	0.018	0.103	0.279
Ncl	0.024	0.140	0.375
Snrpd3	0.002	0.075	0.214
Ganab	0.026	0.086	0.193
Ap3b1	0.000	0.001	0.003
Cfl1	0.093	0.723	1.843
Lrp	0.016	0.048	0.105
Anx	0.041	0.280	0.680
Ywhaq	0.033	0.137	0.298
Tdrd7	0.000	0.001	0.001
Eno1	0.036	0.190	0.415
Krt2	0.001	0.002	0.005

	Tpi1	0.041	0.190	0.402
	Lars2	0.000	0.001	0.003
	Kiaa1468	0.000	0.001	0.001
	Pebp1	0.057	0.329	0.696
	Ctsd	0.064	0.224	0.439
	Ctsa	0.070	0.271	0.531
	Plec	0.002	0.011	0.023
	Serpinb1a	0.044	0.215	0.432
	Nme1	0.041	0.174	0.339
	Eif3i	0.010	0.058	0.117
	Pak2	0.000	0.002	0.004
	Layn	0.001	0.006	0.011
	Calu	0.016	0.071	0.130
	Glul	0.022	0.163	0.313
	Ythdc2	0.000	0.001	0.002
	Lgals1	0.088	0.515	0.948
	Vnn1	0.017	0.053	0.088
	Hspa8	0.018	0.085	0.150
Cluster 3	Protein ID	day3	day5	day8
	Anx	0.077	0.464	0.779
	Asah1	0.062	0.197	0.302
	Hnrnpg	0.001	0.013	0.022
	Ctsl	0.062	0.226	0.328
	Npc2	0.077	0.340	0.482
	Twsg1	0.030	0.116	0.157
	Col7a1	0.000	0.002	0.002
	Tubb5	0.003	0.014	0.019
	Plod1	0.044	0.162	0.207
	Psap	0.193	0.904	1.158
	Tnfsf9	0.017	0.132	0.165
	Rps3a	0.020	0.102	0.124
	Atp6ap1	0.045	0.191	0.223
	Lamc1	0.104	0.264	0.297
	Glb1	0.005	0.009	0.010
	H2-D1	0.001	0.001	0.001
	Col12a1	0.015	0.042	0.047
	App	0.072	0.440	0.493
	Nucb2	0.137	0.594	0.650
	Ctsb	0.118	0.619	0.679
	Lamb1	0.081	0.234	0.252
	Lgmn	0.245	0.882	0.951
	Ppi	0.116	0.677	0.736
	Cadps2	0.001	0.000	0.000
	Icam1	0.047	0.199	0.212
	Hist3h3	0.001	0.000	0.000
	Ppi	0.071	0.252	0.265
	Zeb2	0.001	0.000	0.000
	Gaa	0.058	0.297	0.309
	Rpl7	0.001	0.000	0.000
	Scpep1	0.044	0.144	0.148
	Chd6	0.000	0.001	0.001
	Ddx5	0.000	0.004	0.004
	Crim1	0.005	0.021	0.022
	Cxcr3	0.072	0.378	0.388
	Gapdh	0.002	0.000	0.000
	Aga	0.132	0.580	0.593
	Rplp1	0.002	0.000	0.000
	Rps2	0.002	0.000	0.000
	Aplp2	0.049	0.208	0.205
	Gla	0.027	0.097	0.096
	GLT25D1	0.003	0.000	0.000
	Rab6a	0.000	0.010	0.010
	Sdc	0.005	0.000	0.000
	Nrg1	0.008	0.000	0.000
Cluster 4	Protein ID	day3	day5	day8
	B2m	0.373	1.726	1.328
	Clu	0.385	1.569	1.162
	Timp1	0.346	1.750	1.148
	MFGE8	0.255	1.405	0.999
	Sparc	0.315	1.545	0.871
	DCN	0.167	0.873	0.796
	Sdc	0.223	0.913	0.681
	DKK3	0.222	1.031	0.675
	Nid1	0.198	0.910	0.653
	PCOLCE	0.244	1.029	0.639
	Ccl2	0.192	0.691	0.544
	Htra1	0.082	0.541	0.474
	Nid1	0.112	0.500	0.452
	Cspg4	0.191	0.733	0.424
	Lgals3bp	0.137	0.778	0.423
	C1ra	0.156	0.682	0.420
	Rnaset2	0.152	0.560	0.408
	Nucb1	0.106	0.664	0.406
	Hspg2	0.084	0.464	0.404
	ATP6AP2	0.069	0.422	0.375
	Tinagl1	0.121	0.435	0.374

Mmp9	0.078	0.404	0.360
Lpl	0.127	0.749	0.348
Sbsn	0.116	0.588	0.336
QSOX	0.113	0.658	0.331
Timp2	0.134	0.730	0.330
C1qtnf5	0.103	0.693	0.330
PLTP	0.083	0.522	0.325
Itih5	0.086	0.374	0.318
Gpc1	0.117	0.388	0.317
MFGE8	0.078	0.382	0.315
Pxdnl	0.104	0.416	0.289
Lama5	0.102	0.299	0.274
IGFBP4	0.128	0.637	0.261
Efemp1	0.103	0.507	0.255
I79_001431	0.086	0.482	0.223
pam-b	0.039	0.247	0.194
Sema3b	0.066	0.354	0.179
Gpr56	0.068	0.343	0.179
Alcam	0.083	0.229	0.177
Oaf	0.046	0.337	0.175
LOXL1	0.045	0.311	0.174
SDF4	0.086	0.403	0.173
Gusb	0.049	0.208	0.167
Dag1	0.041	0.185	0.159
Fstl1	0.079	0.318	0.147
Thbs1	0.109	0.583	0.135
Cant1	0.071	0.307	0.133
Cyr61	0.064	0.177	0.127
Vasn	0.047	0.209	0.121
GLG1	0.026	0.192	0.117
Emilin1	0.050	0.186	0.115
Clstn1	0.052	0.238	0.114
Pdpk1	0.039	0.268	0.109
C3	0.075	0.410	0.106
Sema3c	0.079	0.219	0.104
Hexb	0.067	0.254	0.102
ECM1	0.060	0.300	0.097
Mamdc2	0.070	0.258	0.088
BMP1	0.041	0.212	0.084
Sema3e	0.046	0.155	0.084
Ptgfrn	0.018	0.113	0.071
B3GNT	0.026	0.165	0.069
LOXL3	0.027	0.164	0.061
Man2a1	0.019	0.142	0.059
Pdi	0.012	0.059	0.057
Galnt7	0.029	0.129	0.057
Ccdc80	0.023	0.179	0.054
COL3A1	0.015	0.213	0.054
Fuca1	0.031	0.137	0.048
Cd44	0.010	0.044	0.043
Cd109	0.013	0.070	0.043
Man2b1	0.021	0.129	0.042
Mpv17l	0.022	0.043	0.038
Siae	0.027	0.137	0.031
LOXL4	0.051	0.077	0.024
Nkg2d4	0.006	0.029	0.007
Hist1h4	0.000	0.005	0.005
Ptx3	0.022	0.013	0.003
Lpl	0.012	0.037	0.003
SGSH	0.000	0.053	0.002
FAT1	0.007	0.020	0.002
Os9	0.007	0.033	0.002
Adam10	0.011	0.067	0.002
Brd2	0.001	0.003	0.002
Brca2	0.000	0.001	0.000
Golgb1	0.000	0.001	0.000
Huwe1	0.000	0.001	0.000
B4galt1	0.007	0.030	0.000
Pcdhb6	0.000	0.001	0.000
Atrx	0.000	0.001	0.000
Apob	0.000	0.001	0.000
Crybg3	0.000	0.001	0.000
Sdk2	0.000	0.001	0.000
Nlgn3	0.000	0.001	0.000
Cacna1b	0.000	0.001	0.000
Kars	0.000	0.002	0.000
Ntn4	0.002	0.002	0.000
Tuba8	0.000	0.002	0.000
Cntn1	0.001	0.002	0.000
H2-K1	0.001	0.003	0.000
Ptprj	0.000	0.003	0.000
Des	0.000	0.003	0.000
Sepn	0.004	0.003	0.000
Pcbp2	0.000	0.003	0.000
Krt82	0.000	0.004	0.000
Plekhg2	0.000	0.004	0.000

St8sia4	0.000	0.004	0.000
Gapdh	0.000	0.005	0.000
Arf4	0.001	0.006	0.000
Hist1h2b	0.000	0.008	0.000
Fkbp1a	0.000	0.010	0.000
Otub1	0.001	0.012	0.000
COL6A1	0.001	0.019	0.000
Efemp2	0.007	0.031	0.000
LOC100689010	0.009	0.036	0.000
Il1rl1	0.051	0.123	0.000
Ccl7	0.129	0.316	0.000

Fed-batch culture				
Identified Proteins	Protein ID	day3	day8	day12
Beta-catenin-like protein 1	Ctnnbl1	0.000	0.000	0.037
Beta-catenin-like protein 1	Aox	0.000	0.000	0.002
THO complex subunit 2	Thoc2	0.000	0.000	0.003
Kinesin heavy chain isoform 5C	Kif5c	0.000	0.000	0.005
Methionine synthase	MTR	0.000	0.000	0.009
Histone deacetylase	Hdac1	0.000	0.000	0.011
Tubulin beta-4 chain	Tubb4a	0.000	0.000	0.012
Protein NDRG3	Ndrg3	0.000	0.000	0.008
Poly(RC)-binding protein 3	Pcbp3	0.000	0.000	0.021
Zinc finger homeobox protein 3	Zfhx3	0.000	0.000	0.002
Vascular endothelial growth factor receptor 2	Kdr	0.000	0.000	0.002
Protein FAM38B	Piezo2	0.000	0.000	0.002
Rab effector MyRIP	Myrip	0.000	0.000	0.003
Neuron navigator 1	Nav1	0.000	0.000	0.003
Heat shock 70 kDa protein 1L	Hspa1l	0.000	0.000	0.004
Olfactory receptor 2T2	OR2T2	0.000	0.000	0.005
Ubiquilin-2	Ubqln2	0.000	0.000	0.005
Heterogeneous nuclear ribonucleoprotein H2	HNRNPH2	0.000	0.000	0.006
Beta-centractin	Actr1b	0.000	0.000	0.006
Heterogeneous nuclear ribonucleoprotein H (Fra	HNRNPH	0.000	0.000	0.006
Leucine-rich repeat and fibronectin type-III dom	Lrfn2	0.000	0.000	0.007
TATA-binding protein-associated factor 2N	TAF15	0.000	0.000	0.007
Lipid-transfer protein CERTL	CERTL	0.000	0.000	0.008
Microtubule-associated protein RP/EB family me	Mapre3	0.000	0.000	0.009
Caspase 3	Casp3	0.000	0.000	0.009
Putative bifunctional methylenetetrahydrofolate	Mthfd2	0.000	0.000	0.010
Glycylpeptide N-tetradecanoyltransferase	Nmt	0.000	0.000	0.012
ADP-ribosylation factor 4	Arf4	0.000	0.000	0.015
Peptidyl-prolyl cis-trans isomerase E	Ppie	0.000	0.000	0.018
Eukaryotic translation initiation factor 4E	Eif4e	0.000	0.000	0.022
Serine/threonine-protein phosphatase 6 catalytic	PPP6C	0.000	0.000	0.023
Protein FAM107B	Fam107b	0.000	0.000	0.032
Eukaryotic translation initiation factor 5	EIF5	0.000	0.000	0.036
PDZ and LIM domain protein 5	Pdlim5	0.000	0.000	0.037
Ubiquitin-conjugating enzyme E2 variant 2	Ube2v2	0.000	0.000	0.042
Protein CTLA-2-beta	Ctla2b	0.000	0.000	0.055
Histone deacetylase	Hdac1	0.000	0.000	0.046
Transportin-1	Tnpo1	0.000	0.000	0.035
Heat shock factor protein 4	Hsf4	0.000	0.000	0.034
Myosin-Vb	Myo5b	0.000	0.000	0.013
Eukaryotic translation initiation factor 3 subunit	Eif3e	0.000	0.000	0.141
Actin-related protein 2/3 complex subunit 1B	Arpc1b	0.000	0.000	0.092
Diphthamide biosynthesis protein 1	Dph1	0.000	0.000	0.032
Glutathione S-transferase Mu 5	Gstm5	0.000	0.000	0.093
Treacle protein	Tcof1	0.000	0.000	0.013
Liprin-alpha-1	Ppfia1	0.000	0.000	0.018
Serine/threonine-protein phosphatase 2A 55 kDa	Ppp2r2b	0.000	0.000	0.029
Heterogeneous nuclear ribonucleoprotein D-like	Hnrnpdl	0.000	0.000	0.116
Suppressor of tumorigenicity 14 protein homolo	St14	0.025	0.101	0.022
Src substrate cortactin	Cttn	0.000	0.000	0.053
Dynamin	Dnm	0.000	0.000	0.021
Protein FAM98B	Fam98b	0.000	0.000	0.059
Carbonyl reductase 2	Cbr2	0.000	0.000	0.048
Metalloproteinase inhibitor 2	Timp2	0.152	1.313	0.842
Glucosamine-6-phosphate isomerase 2	Gnpda2	0.000	0.000	0.169
NEDD8-conjugating enzyme Ubc12	Ube2m	0.000	0.000	0.131
UPF0368 protein Cxorf26-like	Cxorf26-like	0.000	0.000	0.093
Ras-related protein Rap-1A	Rap1a	0.000	0.000	0.072
Serine/threonine-protein phosphatase	Ppp	0.000	0.000	0.036
28 kDa heat-and acid-stable phosphoprotein	Pdap1	0.000	0.000	0.147
Apoptotic chromatin condensation inducer in th	Acin1	0.000	0.000	0.013
Charged multivesicular body protein 2b (Fragme	Chmp2b	0.000	0.000	0.065
Vigilin	Hdlbp	0.000	0.000	0.033
NudC domain-containing protein 2	Nudcd2	0.000	0.000	0.167
Arfaptin-2	Arfip2	0.000	0.000	0.046
WD repeat-containing protein 6	Wdr6	0.000	0.000	0.029
MAM domain-containing protein 2	Mamdc2	0.070	0.371	0.219
Leucyl-tRNA synthetase, cytoplasmic	Lars	0.000	0.000	0.211
26S protease regulatory subunit 7	Psmc2	0.000	0.000	0.323
U5 small nuclear ribonucleoprotein 200 kDa heli	Snrnp200	0.000	0.000	0.088
Renin receptor	ATP6AP2	0.067	0.623	0.504

Protein ID	day3	Protein ID	day8	Protein ID	day12
Clu	0.38	Clu	2.57	Ywhae	5.90
Timp1	0.34	Timp1	2.16	Prdx1	4.52
Sparc	0.32	Sparc	2.06	Psap	3.83
MFGE8	0.27	PCOLCE	1.98	Pkm	3.39
PCOLCE	0.26	Psap	1.67	Cfl1	3.30
Lgmn	0.25	MFGE8	1.64	Clu	3.13
Psap	0.23	Ccl2	1.58	Timp1	3.11
Sdc	0.23	DKK3	1.52	RPLP2	3.06
DKK3	0.23	DCN	1.46	Hspa5	2.63
Ccl2	0.22	Ywhae	1.41	Pdia6	2.26
Ywhae	0.20	Timp2	1.31	C1qtnf5	2.01
Nid1	0.19	C1qtnf5	1.29	Aldo	1.99
Cspg4	0.19	Lgmn	1.26	DCN	1.94
Rnaset2	0.17	Prdx1	1.20	Sprr1a	1.90
DCN	0.17	Lgals3bp	1.16	PSAT	1.87
Nucb2	0.16	Nid1	1.05	PARK7	1.86
C1ra	0.15	Sdc	1.03	Arhgdia	1.80
Timp2	0.15	Htra1	0.99	PLTP	1.69
Lgals3bp	0.15	Hspg2	0.98	Pgk	1.67
Ctsz	0.14	Cspg4	0.97	Tkt	1.67
IGFBP4	0.13	Pkm	0.90	Gstp	1.65
Ppi	0.12	Rnaset2	0.89	Hsp90b1	1.64
Lpl	0.12	Tinagl1	0.86	Nid1	1.61
Ccl7	0.12	Nid1	0.84	Pgam1	1.59
Nid1	0.12	Ppi	0.84	Ppi	1.51
Cfl1	0.12	Cfl1	0.83	DKK3	1.51
QSOX	0.12	PLTP	0.82	Actb	1.50
Htra1	0.12	Nucb1	0.77	ANX	1.44
Tinagl1	0.12	ANX	0.74	PCOLCE	1.41
Gpc1	0.12	QSOX	0.74	Pgk	1.38
Pkm	0.11	RPLP2	0.73	Eef1b	1.35
Sbsn	0.11	Lpl	0.71	ANX	1.31
Prdx1	0.11	Sbsn	0.68	Cxcr3	1.31
C1qtnf5	0.11	Efemp1	0.67	Ccl2	1.28
Efemp1	0.11	Itih5	0.65	Nucb2	1.23
Nucb1	0.11	C1ra	0.64	Lgmn	1.21
Pxdnl	0.11	App	0.63	Rps8	1.19
Lamc1	0.10	Pxdnl	0.63	Mmp9	1.16
Thbs1	0.10	Nucb2	0.63	Gss	1.16
Lama5	0.10	Pebp1	0.62	Sparc	1.16
Hspg2	0.10	Lama5	0.62	Tinagl1	1.15
Alcam	0.09	ATP6AP2	0.62	Ncl	1.13
Itih5	0.09	Lamc1	0.62	Ywhaz	1.12
PLTP	0.08	Gpc1	0.61	GNB2L1	1.09
ANX	0.08	IGFBP4	0.60	Lamc1	1.08
Hspa5	0.08	Arhgdia	0.59	Nucb1	1.06
MFGE8	0.08	Hspa5	0.59	Eno1	1.06
Sema3c	0.08	Thbs1	0.58	Lgals3bp	1.05
Lamb1	0.08	Mmp9	0.54	Mdh	1.04
Ctsl	0.08	Ctsz	0.52	Ctsd	1.03
C3	0.08	Pgam1	0.51	Aprt	1.03
App	0.08	Gaa	0.51	Ctsa	1.02
Mmp9	0.08	Sema3b	0.50	Hspg2	1.02
Fstl1	0.08	Ddt	0.49	Htra1	1.01
Ctsa	0.07	PSAT	0.48	Npm1	1.01
Gpr56	0.07	ANX	0.45	Ddt	1.01
Cant1	0.07	ECM1	0.44	Hsp90ab1	0.99
Cxcr3	0.07	Lamb1	0.44	Pebp1	0.96
Mamdc2	0.07	PARK7	0.44	MFGE8	0.96
ATP6AP2	0.07	Serpinb1a	0.43	Eef1d	0.95
ECM1	0.07	Ctsd	0.43	Ast	0.93
Mmp19	0.06	MFGE8	0.43	Ilf2	0.92
Sema3b	0.06	Fstl1	0.43	Akr1b1	0.89
Gaa	0.06	Sema3c	0.43	Vcl	0.86
Ctsd	0.06	Alcam	0.42	Ctsz	0.86
Serpinb1a	0.06	Ywhaz	0.41	LMNA	0.85
Pdia6	0.06	Gpr56	0.40	Timp2	0.84
RPLP2	0.06	C3	0.39	Rps24	0.82
Mamdc2	0.06	Tkt	0.38	Snrpa1	0.79
Hexb	0.06	Cant1	0.38	Sbsn	0.77
Clstn1	0.05	Clstn1	0.37	Mmp19	0.77

Far upstream element-binding protein 1	Fubp1	0.000	0.000	0.201
26S protease regulatory subunit 8	Psmc5	0.000	0.000	0.168
Aldehyde dehydrogenase, mitochondrial	Aldh2	0.000	0.000	0.163
Nucleolar RNA helicase 2	Ddx21	0.000	0.000	0.157
Ribosomal L1 domain-containing protein 1	Rsl1d1	0.000	0.000	0.150
EGF-containing fibulin-like extracellular matrix pr	Efemp1	0.106	0.672	0.551
Extracellular matrix protein 1	ECM1	0.066	0.445	0.230
Acidic leucine-rich nuclear phosphoprotein 32 fa	Anp32e	0.000	0.000	0.426
Putative pre-mRNA-splicing factor ATP-depende	Dhx15	0.000	0.000	0.105
ATP synthase subunit beta	Atp5b	0.000	0.000	0.224
Splicing factor 3B subunit 1	Sf3b1	0.000	0.000	0.041
Mannosyl-oligosaccharide 1,2-alpha-mannosidas	Man1a1	0.027	0.208	0.102
AMP deaminase 2	Ampd2	0.000	0.000	0.060
Semaphorin-4B	Sema4b	0.033	0.178	0.176
CAP-Gly domain-containing linker protein 1	Clip1	0.000	0.000	0.043
PEST proteolytic signal-containing nuclear prote	Pcnp	0.000	0.000	0.210
Cysteine and histidine-rich domain-containing p	Chordc1	0.000	0.000	0.150
3-hydroxyacyl-CoA dehydrogenase type-2	Hsd17b10	0.000	0.000	0.170
Cullin-5	Cul5	0.000	0.000	0.098
U1 small nuclear ribonucleoprotein 70 kDa	Snrnp70	0.000	0.000	0.089
Rho GTPase-activating protein 1	Arhgap1	0.000	0.000	0.170
Acyl-coenzyme A thioesterase 2, mitochondrial	Acot2	0.000	0.000	0.122
Agrin	Agrn	0.033	0.183	0.188
Cell growth-regulating nucleolar protein	Lyar	0.000	0.000	0.200
Nitrilase-like 1	Nit1	0.000	0.000	0.299
Nucleobindin-2	Nucb2	0.160	0.626	1.228
V-type proton ATPase subunit S1	Atp6ap1	0.048	0.221	0.374
Serine protease HTRA1	Htra1	0.117	0.988	1.007
Interleukin enhancer-binding factor 2	Ilf2	0.000	0.000	0.915
Protein RCC2	Rcc2	0.000	0.000	0.145
Chromobox protein-like 5	Cbx5	0.000	0.000	0.195
Non-POU domain-containing octamer-binding p	Nono	0.000	0.000	0.095
SEC23-interacting protein	Sec23ip	0.000	0.000	0.042
Signal recognition particle 72 kDa protein	Srp72	0.000	0.000	0.083
Cytoplasmic dynein 1 light intermediate chain 2	Dync1i2	0.000	0.000	0.060
Isoleucyl-tRNA synthetase, cytoplasmic	Iars	0.000	0.000	0.121
Heat shock protein HSP 90-beta	Hsp90ab1	0.049	0.312	0.989
Amyloid-like protein 2	Aplp2	0.048	0.294	0.335
Developmentally-regulated GTP-binding protein	Drg1	0.000	0.000	0.308
HEAT repeat-containing protein 3	Heatr3	0.000	0.000	0.104
Scaffold attachment factor B1	Safb	0.000	0.000	0.037
Coatomer subunit gamma	Copg	0.000	0.000	0.106
Alpha-mannosidase 2	Man2a1	0.020	0.192	0.101
Cathepsin L1	Ctsl	0.078	0.343	0.522
Squamous cell carcinoma antigen recognized by	Sart3	0.000	0.000	0.075
Large proline-rich protein BAT3	Bat3	0.000	0.000	0.042
Eukaryotic translation initiation factor 4 gamma	Eif4g1	0.000	0.000	0.030
Lactadherin	MFGE8	0.267	1.638	0.961
40S ribosomal protein S27	Rps27	0.000	0.000	0.252
Leucyl-tRNA synthetase, cytoplasmic	Lars	0.000	0.000	0.134
Glutaminyl-tRNA synthetase	Qars	0.000	0.000	0.154
Putative ribosomal RNA methyltransferase NOP2	Nop2	0.000	0.000	0.137
Protein transport protein Sec23A	Sec23a	0.000	0.000	0.118
Inosine-5'-monophosphate dehydrogenase	Impdh	0.000	0.000	0.078
Cleavage and polyadenylation specificity factor s	Cpsf6	0.000	0.000	0.068
Insulin-like growth factor-binding protein 4	IGFBP4	0.130	0.603	0.432
Succinyl-CoA:3-ketoacid-coenzyme A transferase	Oxct1	0.000	0.000	0.056
Retinal dehydrogenase 1	Aldh1a1	0.000	0.000	0.047
U6 snRNA-associated Sm-like protein LSm4	Lsm4	0.000	0.000	0.149
Prostaglandin F2 receptor negative regulator	Ptgfrn	0.018	0.192	0.105
Splicing factor, arginine/serine-rich 1 (Fragment)	Srsf1	0.000	0.000	0.265
Semaphorin-3C	Sema3c	0.081	0.426	0.194
Golgin subfamily A member 5	Golga5	0.000	0.000	0.025
GTP:AMP phosphotransferase mitochondrial	Ak3	0.000	0.000	0.139
Dynamin-like 120 kDa protein, mitochondrial	Opa1	0.000	0.000	0.023
Suprabasin	Sbsn	0.113	0.682	0.772
40S ribosomal protein S17	Rps17	0.000	0.000	0.413
26S proteasome non-ATPase regulatory subunit	Psmd3	0.000	0.000	0.401
Semaphorin-3E	Sema3e	0.044	0.242	0.101
Cytoskeleton-associated protein 5	Ckap5	0.000	0.000	0.019
tRNA (Cytosine-5-)-methyltransferase NSUN2	Nsun2	0.000	0.000	0.081
Protein arginine N-methyltransferase 5	Prmt5	0.000	0.000	0.213
C-C motif chemokine 2	Ccl2	0.221	1.575	1.282
Cytoplasmic FMR1-interacting protein 1	Cyfip1	0.000	0.000	0.032
Poly(U)-binding-splicing factor PUF60	Puf60	0.000	0.000	0.147
Focal adhesion kinase 1	Fak1	0.000	0.000	0.036
Kinesin-1 heavy chain	Kif5b	0.000	0.000	0.081
Sorting nexin-6 (Fragment)	Snx6	0.000	0.000	0.104
THUMP domain-containing protein 1	Thumpd1	0.000	0.000	0.133
Small nuclear ribonucleoprotein F	Snrpf	0.000	0.000	0.321
Calmodulin	Calm	0.000	0.000	0.182
Dual specificity mitogen-activated protein kinase	Map2k1	0.000	0.000	0.033
Phosphoribosylaminoimidazole carboxylase/ pho	Paics	0.000	0.000	0.031
26S protease regulatory subunit 6B (Fragment)	Psmc4	0.000	0.000	0.064
GDP-L-fucose synthase	Tsta3	0.000	0.000	0.049
Huntingtin-interacting protein K	Hypk	0.000	0.000	0.130

Ywhaz	0.05	Mamdc2	0.37	Capzb	0.76
Emilin1	0.05	Icam1	0.37	Nid1	0.75
Icam1	0.05	LOXL1	0.36	Pgls	0.73
Pgk	0.05	Mamdc2	0.35	Psma	0.73
Hsp90ab1	0.05	Ctsl	0.34	Actn1	0.72
Srpx	0.05	Cxcr3	0.34	Capg	0.72
Tkt	0.05	Aldo	0.34	Sdc	0.69
Arhgdia	0.05	Actb	0.34	Hprt1	0.68
Atp6ap1	0.05	Pgk	0.34	Ywhaq	0.68
Aplp2	0.05	Ccl7	0.33	Hmox1	0.67
Ddt	0.05	Dag1	0.33	Pdi	0.67
Gstp	0.05	Ctsa	0.33	Lama5	0.67
Pebp1	0.05	pam-b	0.32	Nudc	0.66
Dbi	0.05	Plod1	0.32	Lamb1	0.66
Plod1	0.05	Srpx	0.32	Gaa	0.64
pam-b	0.05	Ast	0.32	Serpinb1a	0.63
LOXL1	0.04	Gstp	0.32	Nsfl1c	0.63
Dag1	0.04	Dbi	0.31	Dbi	0.62
Sema3e	0.04	Hsp90ab1	0.31	Pfdn5	0.62
ANX	0.04	Hexb	0.31	Cspg4	0.61
Hsp90b1	0.04	Akr1b1	0.31	Bola1	0.60
Pdi	0.04	Mmp19	0.30	EIF6	0.59
Aldo	0.04	Hsp90b1	0.30	QSOX	0.59
BMP1	0.04	Aplp2	0.29	Timm13	0.58
PSAT	0.04	Pgk	0.29	Pa2g4	0.56
Gm2a	0.04	Pdia6	0.29	Efemp1	0.55
Pgam1	0.04	GNB2L1	0.28	Gpc1	0.55
Naga	0.04	Emilin1	0.28	Plod1	0.54
Akr1b1	0.04	Ywhaq	0.28	App	0.54
PARK7	0.04	Eno1	0.28	Ctsl	0.52
Eno1	0.04	Pdi	0.26	C1ra	0.51
Actb	0.03	Psma	0.26	Ruvbl1	0.51
Sema4b	0.03	Eef1b	0.25	ATP6AP2	0.50
Npm1	0.03	Akr1b8	0.25	LOXL1	0.50
Agrn	0.03	Hprt1	0.25	Rps3a	0.50
B3GNT	0.03	Sema3e	0.24	Pxdnl	0.50
Rpl12	0.03	Ccdc80	0.24	Rnaset2	0.48
Twsg1	0.03	Rpl12	0.24	Icam1	0.47
Eef1d	0.03	GLG1	0.23	Acat2	0.46
Akr1b8	0.03	Atp6ap1	0.22	Hexb	0.44
Tnfsf9	0.03	M오	0.22	IGFBP4	0.43
Ywhaq	0.03	Ncl	0.22	Anp32e	0.43
Rcn3	0.03	Eef1d	0.22	Psmb	0.42
Man1a1	0.03	Acat2	0.21	Cope	0.42
M오	0.03	Capzb	0.21	Rps17	0.41
Smpd	0.03	Man1a1	0.21	Snrpd3	0.41
Ccdc80	0.03	Npm1	0.21	Psmd3	0.40
Eef1b	0.03	Slc3a2	0.20	Cyb5	0.40
GLG1	0.03	Gm2a	0.20	MFGE8	0.40
Ggh	0.03	Plbd2	0.19	C3	0.39
Sepp1	0.03	Gdi2	0.19	Pfdn2	0.39
Plbd2	0.02	Man2a1	0.19	Mapre1	0.38
St14	0.02	Ptgfrn	0.19	Rcn3	0.38
Ncl	0.02	Nudc	0.19	Rpl12	0.38
GNB2L1	0.02	Psmb	0.19	Rpl13	0.38
Klk1b3	0.02	LMNA	0.19	Atp6ap1	0.37
Calcrl	0.02	BMP1	0.18	Sema3b	0.37
Gdi2	0.02	Agrn	0.18	Tgm2	0.36
Capzb	0.02	B3GNT	0.18	Naga	0.35
Actn1	0.02	Tnfsf9	0.18	Arpc4	0.35
Gsto1	0.02	Gss	0.18	Emilin1	0.34
Ganab	0.02	Sema4b	0.18	Itih5	0.34
Ast	0.02	Actn1	0.17	EIF3M	0.34
C19orf10-like	0.02	Ggh	0.17	Hspa8	0.34
Cln5	0.02	Sepp1	0.17	Aplp2	0.33
Pgk	0.02	Lgals	0.17	Thbs1	0.33
Man2a1	0.02	Twsg1	0.17	Akr1b8	0.33
Hsp90aa1	0.02	Capg	0.17	Glrx3	0.33
Acat2	0.02	Nsfl1c	0.16	Psmc2	0.32
Hprt1	0.02	Rps8	0.15	PSME3	0.32
Rps3a	0.02	Rcn3	0.15	Snrpf	0.32
Hspa8	0.02	Rps24	0.15	Fstl1	0.32
Ptgfrn	0.02	Hspa8	0.14	Dag1	0.32
LMNA	0.02	Naga	0.14	Hnrnpab	0.31
Nsfl1c	0.02	Smpd	0.14	Drg1	0.31
Nudc	0.02	Gsto1	0.13	Plbd2	0.31
Gns	0.02	Calcrl	0.13	Gns	0.31
Anf	0.02	Hsp90aa1	0.12	Stx12	0.31
Tgfbr3	0.02	Vcl	0.12	Clta	0.30
Prkcsh	0.02	Fn1	0.11	Cs	0.30
Gss	0.02	St14	0.10	Nucb1	0.30
Thbs3	0.02	Pitpna	0.10	Imnl1	0.30
COL3A1	0.02	Hnrnpab	0.10	Prmt1	0.30
Rps8	0.01	Nrp1	0.10	Nit1	0.30
Capg	0.01	Rps3a	0.10	Anf	0.30
EIF6	0.01	ITM2C	0.09	Cltc	0.29

DnaJ-like subfamily C member 9	Dnajc9	0.000	0.000	0.049
Putative proline racemase	prdF	0.000	0.000	0.028
Cellular nucleic acid-binding protein	Cnbp	0.000	0.000	0.090
Copine-3	Cpne3	0.000	0.000	0.030
Ubiquilin-1	Ubqln1	0.000	0.000	0.142
Importin subunit alpha	Kpna2	0.000	0.000	0.017
Collagen alpha-5(VI) chain	Col6a5	0.000	0.000	0.002
Ras GTPase-activating-like protein IQGAP2	Iqgap2	0.000	0.000	0.002
Thrombospondin-4 (Fragment)	THBS4	0.000	0.000	0.003
Protein phosphatase 1 regulatory subunit 12	PPP1R12	0.000	0.000	0.003
Chromodomain-helicase-DNA-binding protein 2	Chd2	0.000	0.000	0.003
Glucocorticoid receptor DNA-binding factor 1	GRLF1	0.000	0.000	0.004
C-jun-amino-terminal kinase-interacting protein	Mapk8ip4	0.000	0.000	0.005
Trifunctional purine biosynthetic protein adenosi	Gart	0.000	0.000	0.021
Tubulin alpha-8 chain	Tuba8	0.000	0.000	0.006
Aspartyl-tRNA synthetase, cytoplasmic	DARS	0.000	0.000	0.006
Cell division protein kinase 9	CDK9	0.000	0.000	0.007
Ferritin	Fth1	0.000	0.000	0.008
Poly(RC)-binding protein 2	Pcbp2	0.000	0.000	0.008
Oxysterol-binding protein-related protein 6	Osbpl6	0.000	0.000	0.009
Neurabin-2	Ppp1r9b	0.000	0.000	0.009
Protein FAM26D	Fam26d	0.000	0.000	0.011
Bcl-2-associated transcription factor 1	Bclaf1	0.000	0.000	0.013
Protein ENL	MLLT1	0.000	0.000	0.013
Bone marrow stromal antigen 2	Bst2	0.000	0.000	0.014
Isocitrate dehydrogenase [NAD] subunit alpha, n	Idh3a	0.000	0.000	0.037
NFU1 iron-sulfur cluster scaffold-like, mitochond	NFU1	0.000	0.000	0.016
Glycine cleavage system H protein, mitochondria	GCSH	0.000	0.000	0.016
60S ribosomal protein L12	Rpl12	0.000	0.000	0.016
Protein SET	SET	0.000	0.000	0.018
Ubiquitin-conjugating enzyme E2 variant 1	Ube2v1	0.000	0.000	0.025
Splicing factor, arginine/serine-rich 7 (Fragment)	Srsf7	0.000	0.000	0.025
Mitogen-activated protein kinase 1	Mapk1	0.000	0.000	0.042
Transaldolase	Taldo1	0.000	0.010	0.000
Fibronectin	Fn1	0.011	0.109	0.000
Programmed cell death protein 5	PDCD5	0.000	0.000	0.081
Cathepsin Z	Ctsz	0.138	0.523	0.857
Basement membrane-specific heparan sulfate pr	Hspg2	0.101	0.979	1.023
Dystroglycan	Dag1	0.044	0.329	0.320
Phospholipid transfer protein	PLTP	0.085	0.825	1.689
Phosphoserine aminotransferase	PSAT	0.040	0.477	1.871
78 kDa glucose-regulated protein	Hspa5	0.083	0.586	2.629
Decorin	DCN	0.173	1.457	1.945
Glucosidase 2 subunit beta	Prkcsh	0.016	0.074	0.251
Laminin subunit beta-1	Lamb1	0.081	0.445	0.658
Matrix metalloproteinase-19	Mmp19	0.065	0.298	0.768
Laminin subunit alpha-5	Lama5	0.102	0.623	0.666
Phosphoglycerate kinase	Pgk	0.050	0.336	1.669
CTP synthase	Ctps	0.000	0.000	0.027
Uridine 5'-monophosphate synthase	Umps	0.000	0.000	0.066
Cysteine-rich with EGF-like domain protein 2	Creld2	0.004	0.013	0.046
Cell division protein kinase 6	Cdk6	0.000	0.000	0.058
Alpha-soluble NSF attachment protein	Napa	0.000	0.000	0.154
RecName: Full	RecName: Full	0.000	0.000	0.064
AH receptor-interacting protein	Aip	0.000	0.000	0.112
Sushi repeat-containing protein SRPX	Srpx	0.049	0.318	0.194
Collagen alpha-1(III) chain	COL3A1	0.015	0.066	0.232
Heat shock protein 75 kDa, mitochondrial	Trap1	0.000	0.000	0.082
Glycylpeptide N-tetradecanoyltransferase	Nmt	0.000	0.000	0.032
LIM domain and actin-binding protein 1	Lima1	0.000	0.000	0.031
WD repeat-containing protein 61	Wdr61	0.000	0.000	0.200
CD166 antigen	Alcam	0.091	0.416	0.236
Procollagen C-endopeptidase enhancer 1	PCOLCE	0.262	1.980	1.413
Clusterin	Clu	0.381	2.565	3.132
Dickkopf-related protein 3	DKK3	0.228	1.517	1.511
G-protein coupled receptor 56	Gpr56	0.073	0.400	0.258
Annexin	ANX	0.043	0.452	1.311
Glypican-1	Gpc1	0.116	0.611	0.547
Lysosomal alpha-glucosidase	Gaa	0.065	0.505	0.638
Glutathione S-transferase P	Gstp	0.047	0.316	1.650
Leukocyte elastase inhibitor A	Serpinb1a	0.058	0.435	0.634
Protein transport protein Sec24C	Sec24C	0.000	0.000	0.148
Peroxidasin-like	Pxdnl	0.106	0.630	0.496
Inter-alpha-trypsin inhibitor heavy chain H5	Itih5	0.091	0.646	0.341
Pyruvate kinase	Pkm	0.114	0.905	3.392
Metalloproteinase inhibitor 1	Timp1	0.344	2.162	3.111
Lipoprotein lipase	Lpl	0.124	0.709	0.264
RuvB-like 1	Ruvbl1	0.000	0.000	0.508
Legumain	Lgmn	0.253	1.258	1.211
4F2 cell-surface antigen heavy chain	Slc3a2	0.011	0.203	0.261
Ubiquitin thioesterase OTUB1	Otub1	0.000	0.000	0.253
Matrix metalloproteinase-9	Mmp9	0.075	0.542	1.158
Tubulointerstitial nephritis antigen-like	Tinagl1	0.117	0.863	1.146
Xanthine dehydrogenase/oxidase	Xdh	0.000	0.000	0.061
Semaphorin-3B	Sema3b	0.065	0.499	0.373
SPARC	Sparc	0.316	2.061	1.157

Sep15	0.01	Timm13	0.09	EIF3F	0.28
Nrp1	0.01	Ganab	0.09	Shmt1	0.28
Vcl	0.01	FKBP9	0.09	C19orf10-like	0.27
Lgals	0.01	C19orf10-like	0.08	Srsf1	0.26
FKBP9	0.01	Pfdn5	0.08	Lpl	0.26
Slc3a2	0.01	Ezr	0.08	Smpd	0.26
Fn1	0.01	Tgfbr3	0.08	Cops4	0.26
Tnxb	0.01	EIF6	0.08	Slc3a2	0.26
Psmb	0.01	Cln5	0.07	Gpr56	0.26
Hsp90aa1	0.01	Prkcsh	0.07	Gsto1	0.26
Psma	0.01	Thbs3	0.07	Gstm1	0.26
Pitpna	0.01	Vim	0.07	Fkbp10	0.25
Arpc4	0.01	Prmt1	0.07	Otub1	0.25
Hnrnpab	0.01	Snrpd3	0.07	Hadh	0.25
B4galt1	0.01	Tnxb	0.07	Rps27	0.25
Prmt1	0.01	COL3A1	0.07	Prkcsh	0.25
FAT1	0.01	Sep15	0.06	Twsg1	0.25
Vcp	0.01	Tpm1	0.06	Pitpna	0.25
Ezr	0.01	Sprr1a	0.06	Cant1	0.25
Rps24	0.01	Cltc	0.06	Hsp90aa1	0.24
Gp	0.00	Arpc4	0.06	Alcam	0.24
Anp32b	0.00	Ccdc85a	0.05	Idi1	0.23
Creld2	0.00	Klk1b3	0.05	COL3A1	0.23
RCN1	0.00	Gns	0.05	Ezr	0.23
Glb1	0.00	B4galt1	0.05	ECM1	0.23
Serpinh1	0.00	Anf	0.05	GLG1	0.23
ITM2C	0.00	Hsp90aa1	0.05	Anp32b	0.22
Vim	0.00	Atp1a1	0.05	Atp5b	0.22
Pdxk	0.00	Glb1	0.04	pam-b	0.22
Cltc	0.00	Idi1	0.04	Mamdc2	0.22
Sprr1a	0.00	Arpc2	0.04	NARS	0.22
G6pd	0.00	FAT1	0.03	Dkc1	0.22
Ppia	0.00	Imnl1	0.03	Gdi2	0.21
Stip1	0.00	Sptan1	0.03	FKBP9	0.21
Rpsa	0.00	Psmb6	0.03	Prmt5	0.21
Crim1	0.00	Pacsin2	0.03	Vcp	0.21
Rem1	0.00	Rras2	0.02	Lars	0.21
Plxnb2	0.00	Anp32b	0.02	Pcnp	0.21
Tpm1	0.00	Rpl13	0.02	Ganab	0.21
Pdlim1	0.00	Ehd3	0.02	Asns	0.21
Atp1a1	0.00	Vcp	0.02	Cse1l	0.21
Pacsin2	0.00	Slc7a5	0.02	Zpr1	0.20
Rhoc	0.00	Mapre1	0.02	Ssrp1	0.20
Pfdn2	0.00	Arf5	0.02	Acly	0.20
Pa2g4	0.00	Cyb5	0.02	Fubp1	0.20
Cnpy4	0.00	Enpp6	0.02	Lyar	0.20
Fus	0.00	Plxnb2	0.01	Wdr61	0.20
Sptan1	0.00	Actr2	0.01	Rtcb	0.20
Mapre1	0.00	Rps21	0.01	Tnfsf9	0.20
Krt2	0.00	Snrpa1	0.01	DAK	0.20
Casp3	0.00	Cnpy4	0.01	Akr7a2	0.20
Cbr1	0.00	Creld2	0.01	Bub3	0.20
Arpc2	0.00	Atp2b1	0.01	Acy1a	0.19
Eef1a1	0.00	Pa2g4	0.01	Cbx5	0.19
Eno	0.00	Serpinh1	0.01	Srpx	0.19
Stx12	0.00	Aprt	0.01	U2af2	0.19
Incenp	0.00	Plxnb2	0.01	MARS	0.19
Cd276	0.00	Rab8b	0.01	Sema3c	0.19
Bub3	0.00	Rps2	0.01	Agrn	0.19
Col5a3	0.00	Zpr1	0.01	Syncrip	0.19
Krt15	0.00	Pdxk	0.01	Btf3l4	0.18
U2af2	0.00	Sema6c	0.01	Hnrnpul2	0.18
Krt1b	0.00	Pdlim1	0.01	EIF3D	0.18
Impdh2	0.00	Ndrg1	0.01	Calm	0.18
Tank	0.00	Taldo1	0.01	Klk1b3	0.18
Eno2	0.00	Ldha	0.01	Man2c1	0.18
Ppm1f	0.00	Ppm1f	0.01	Sema4b	0.18
Antxr2	0.00	LASP1	0.01	Vim	0.18
Mmp12	0.00	Gp	0.01	Mybbp1a	0.17
Ehd3	0.00	RCN1	0.01	Map	0.17
EIF3D	0.00	Acy1a	0.01	Ppm1f	0.17
Pkm	0.00	His2A	0.01	Cfdp1	0.17
C6	0.00	Syncrip	0.01	Hsd17b10	0.17
Tgm2	0.00	Rab35	0.01	Arhgap1	0.17
Syncrip	0.00	Asns	0.01	Gnpda2	0.17
Fkbp10	0.00	Stx12	0.01	Psmc5	0.17
Hnrnpul2	0.00	Dcaf12	0.01	Psmb6	0.17
Kdm2b	0.00	Rps2	0.01	Ggct	0.17
Arhgef7	0.00	Btf3l4	0.01	Nudcd2	0.17
Krt76	0.00	Rhoc	0.01	Calcrl	0.17
DMP4	0.00	NARS	0.01	Aldh2	0.16
Pdxdc1	0.00	Ggct	0.01	LASP1	0.16
Ap1g1	0.00	Pgls	0.01	Hsp90aa1	0.16
Supt16h	0.00	Gstm1	0.01	Ddx21	0.16
Cse1l	0.00	G6pd	0.01	Kpna2	0.15
Akap8l	0.00	Antxr2	0.01	Napa	0.15

Laminin subunit gamma-1	Lamc1	0.105	0.616	1.080
EMILIN-1	Emilin1	0.050	0.280	0.343
Complement C1r-A subcomponent	C1ra	0.154	0.642	0.512
Sulfhydryl oxidase	QSOX	0.118	0.739	0.588
Syndecan	Sdc	0.228	1.028	0.693
Alpha-actinin-1	Actn1	0.023	0.173	0.719
Nucleobindin-1	Nucb1	0.106	0.770	1.061
Chondroitin sulfate proteoglycan 4	Cspg4	0.192	0.967	0.614
Nucleophosmin	Npm1	0.033	0.207	1.007
Alpha-N-acetylglucosaminidase	Naglu	0.000	0.000	0.118
Follistatin-related protein 1	Fstl1	0.075	0.427	0.321
Nidogen-1	Nid1	0.194	1.049	1.609
Lysyl oxidase-like 1	LOXL1	0.045	0.361	0.501
Sulfated glycoprotein 1	Psap	0.228	1.666	3.833
Coiled-coil domain-containing protein 80	Ccdc80	0.026	0.242	0.139
Aminoacylase-1A	Acy1a	0.000	0.009	0.195
Complement C3	C3	0.078	0.388	0.389
Golgi apparatus protein 1	GLG1	0.026	0.227	0.229
Sialic acid synthase	Nans	0.000	0.000	0.154
Septin-6	Sept6	0.000	0.000	0.134
Nck-associated protein 1 (Fragment)	Nckap1	0.000	0.000	0.055
Nidogen-1	Nid1	0.121	0.837	0.753
Macrophage-capping protein	Capg	0.014	0.167	0.718
BolA-like protein 1	Bola1	0.000	0.000	0.595
Complement C1q tumor necrosis factor-related	C1qtnf5	0.112	1.288	2.010
Ezrin	Ezr	0.006	0.079	0.231
Myb-binding protein 1A	Mybbp1a	0.000	0.000	0.173
H/ACA ribonucleoprotein complex subunit 4	Dkc1	0.000	0.000	0.215
Importin subunit alpha	Kpna2	0.000	0.000	0.155
Vimentin	Vim	0.003	0.071	0.175
Thrombospondin-1	Thbs1	0.104	0.581	0.332
Intercellular adhesion molecule 1	Icam1	0.050	0.367	0.467
Beta-hexosaminidase	Hexb	0.056	0.312	0.444
Procollagen-lysine,2-oxoglutarate 5-dioxygenase	Plod1	0.045	0.321	0.544
116 kDa U5 small nuclear ribonucleoprotein con	Eftud2	0.000	0.000	0.117
Aflatoxin B1 aldehyde reductase member 2	Akr7a2	0.000	0.000	0.196
Hydroxyacyl-coenzyme A dehydrogenase, mitoch	Hadh	0.000	0.000	0.253
Phosphoglycolate phosphatase	Pgp	0.000	0.000	0.103
Clathrin light chain A	Clta	0.000	0.000	0.304
Galectin-3-binding protein	Lgals3bp	0.147	1.162	1.053
Nuclear migration protein nudC	Nudc	0.018	0.189	0.664
Aconitate hydratase, mitochondrial	Aco2	0.000	0.000	0.146
Peptidyl-glycine alpha-amidating monooxygenas	pam-b	0.045	0.324	0.224
Putative phospholipase B-like 2	Plbd2	0.025	0.195	0.308
Vinculin	Vcl	0.012	0.118	0.861
Beta-galactosidase (Fragment)	Glb1	0.004	0.044	0.073
Peripheral-type benzodiazepine receptor-associa	Bzrap1	0.000	0.001	0.001
Complement component C7	C7	0.000	0.005	0.002
F-box only protein 43	Fbxo43	0.000	0.002	0.002
Adenosylhomocysteinase	Ahcy	0.000	0.003	0.004
40S ribosomal protein S21	Rps21	0.000	0.014	0.031
FACT complex subunit SSRP1	Ssrp1	0.000	0.003	0.204
Coatomer subunit beta'	Copb2	0.000	0.002	0.144
Small nuclear ribonucleoprotein Sm D3	Snrpd3	0.000	0.067	0.406
Adenosine kinase	ADK	0.000	0.007	0.102
Mitochondrial import inner membrane transloca	Timm13	0.000	0.092	0.580
Peflin	Pef1	0.000	0.005	0.106
Kinectin	Ktn1	0.000	0.002	0.025
Kinesin light chain 1	Klc1	0.000	0.002	0.030
Microtubule-associated protein 1A	Map1a	0.000	0.001	0.008
Serine/threonine-protein phosphatase (Fragment	Ppp5c	0.000	0.004	0.038
Glutaredoxin-3	Glrx3	0.000	0.003	0.325
Nucleoprotein TPR	Tpr	0.000	0.001	0.045
ATP-citrate synthase	Acly	0.000	0.003	0.201
Nuclease domain-containing protein 1	Nucb1	0.000	0.006	0.302
Tumor necrosis factor ligand superfamily membe	Tnfsf9	0.029	0.180	0.198
Heterogeneous nuclear ribonucleoprotein U	Hnrnpu	0.000	0.002	0.118
AP-2 complex subunit alpha-2	Ap2a2	0.000	0.001	0.062
6-phosphogluconolactonase	Pgls	0.000	0.007	0.735
Eukaryotic translation initiation factor 3 subunit	EIF3F	0.000	0.006	0.285
Methionine aminopeptidase 2	Metap2	0.000	0.002	0.132
60S ribosomal protein L12	Rpl12	0.032	0.240	0.377
Annexin	ANX	0.000	0.003	0.106
Thrombospondin-3	Thbs3	0.016	0.073	0.103
COP9 signalosome complex subunit 4	Cops4	0.000	0.006	0.262
60S ribosomal protein L13	Rpl13	0.000	0.022	0.375
Calcitonin gene-related peptide type 1 receptor	Calcrl	0.024	0.129	0.166
Zinc finger protein ZPR1	Zpr1	0.000	0.011	0.205
FK506-binding protein 9	FKBP9	0.011	0.086	0.214
Neutral alpha-glucosidase AB	Ganab	0.023	0.087	0.209
tRNA-splicing ligase RtcB homolog	Rtcb	0.000	0.003	0.198
Coatomer subunit epsilon	Cope	0.000	0.004	0.420
U2 small nuclear ribonucleoprotein A'	Snrpa1	0.000	0.013	0.785
Importin-9	Ipo9	0.000	0.001	0.136
Sphingomyelin phosphodiesterase	Smpd	0.027	0.139	0.263
N-acetyllactosaminide beta-1,3-N-acetylglucosan	B3GNT	0.033	0.182	0.139

PDS5A	0.00	Cd276	0.01	Qars	0.15
Abca1	0.00	Tgm2	0.01	Nans	0.15
Gmps	0.00	MAGED2	0.01	Sepp1	0.15
DGK	0.00	Adprh	0.01	Pacsin2	0.15
Flnc	0.00	Cnrip1	0.01	Rsl1d1	0.15
Ncoa2	0.00	ADK	0.01	Chordc1	0.15
Eppk1	0.00	Krt2	0.01	Lsm4	0.15
Xirp2	0.00	Nucb1	0.01	Impdh2	0.15
Myh10	0.00	Hist1h4a	0.01	Sec24C	0.15
Apc	0.00	EIF3F	0.01	Pdap1	0.15
Ryr2	0.00	Cops4	0.01	Puf60	0.15
Srrm2	0.00	C7	0.01	Aco2	0.15
Csmd2	0.00	U2af2	0.01	Pdlim1	0.14
Birc6	0.00	Ppp	0.01	Rcc2	0.14
Pclo	0.00	Phf1	0.01	Copb2	0.14
Ctnnbl1	0.00	Rab2b	0.01	Clstn1	0.14
Aox	0.00	Rpl7	0.01	Ubqln1	0.14
Thoc2	0.00	Rpsa	0.00	Eif3e	0.14
Kif5c	0.00	LDH	0.00	BMP1	0.14
MTR	0.00	Ppcs	0.00	B3GNT	0.14
Hdac1	0.00	Hist1h3a	0.00	Ccdc80	0.14
Tubb4a	0.00	Pef1	0.00	Ak3	0.14
Ndrg3	0.00	C6	0.00	Gm2a	0.14
Pcbp3	0.00	PSME3	0.00	Nop2	0.14
Zfhx3	0.00	SH3RF3	0.00	Ipo9	0.14
Kdr	0.00	Cfdp1	0.00	Ccl7	0.13
Piezo2	0.00	Cd74	0.00	Adprh	0.13
Myrip	0.00	Hmox1	0.00	Lars	0.13
Nav1	0.00	EIF3M	0.00	Sept6	0.13
Hspa1l	0.00	Ppp5c	0.00	Cnpy4	0.13
OR2T2	0.00	Rpl6	0.00	Eno2	0.13
Ubqln2	0.00	Lamp1	0.00	Copb1	0.13
HNRNPH2	0.00	Rps2	0.00	Thumpd1	0.13
Actr1b	0.00	Cope	0.00	Arpc2	0.13
HNRNPH	0.00	Krt1b	0.00	Metap2	0.13
Lrfn2	0.00	Cs	0.00	Ube2m	0.13
TAF15	0.00	Psmd7	0.00	Hypk	0.13
CERTL	0.00	Dync2h1	0.00	Cln5	0.13
Mapre3	0.00	Eef1a	0.00	Sep15	0.12
Casp3	0.00	Glrx3	0.00	Acot2	0.12
Mthfd2	0.00	DUS3L	0.00	Rab2b	0.12
Nmt	0.00	Asxl1	0.00	Iars	0.12
Arf4	0.00	Ppp4r3b	0.00	Naglu	0.12
Ppie	0.00	Ssrp1	0.00	Hnrnpu	0.12
Eif4e	0.00	Acly	0.00	Sec23a	0.12
PPP6C	0.00	Actg2	0.00	Eftud2	0.12
Fam107b	0.00	Inpp4a	0.00	Hnrnpdl	0.12
EIF5	0.00	Stip1	0.00	RCN1	0.12
Pdlim5	0.00	PRDM15	0.00	Supt16h	0.11
Ube2v2	0.00	Rtcb	0.00	Aip	0.11
Ctla2b	0.00	Cyp4f4	0.00	ANX	0.11
Hdac1	0.00	Ssb	0.00	Copg	0.11
Tnpo1	0.00	Pkm	0.00	Pef1	0.11
Hsf4	0.00	Copb1	0.00	Ptgfrn	0.11
Myo5b	0.00	DAK	0.00	Dhx15	0.10
Eif3e	0.00	Shc1	0.00	Heatr3	0.10
Arpc1b	0.00	ANX	0.00	Snx6	0.10
Dph1	0.00	Ahcy	0.00	Pgp	0.10
Gstm5	0.00	Ncoa2	0.00	Thbs3	0.10
Tcof1	0.00	Metap2	0.00	ADK	0.10
Ppfia1	0.00	Shmt1	0.00	Man1a1	0.10
Ppp2r2b	0.00	Hnrnpu	0.00	Sema3e	0.10
Hnrnpdl	0.00	Fbxo43	0.00	Man2a1	0.10
Cttn	0.00	Mybpc1	0.00	Tpm1	0.10
Dnm	0.00	Ktn1	0.00	Cul5	0.10
Fam98b	0.00	Klc1	0.00	Rhoc	0.10
Cbr2	0.00	PFK	0.00	Nono	0.09
Gnpda2	0.00	YES1	0.00	Mamdc2	0.09
Ube2m	0.00	Stip1	0.00	Gstm5	0.09
Cxorf26-like	0.00	Fkbp10	0.00	Cxorf26-like	0.09
Rap1a	0.00	Copb2	0.00	Arpc1b	0.09
Ppp	0.00	ABCB1	0.00	Cnbp	0.09
Pdap1	0.00	MARS	0.00	Snrnp70	0.09
Acin1	0.00	Cdh23	0.00	Snrnp200	0.09
Chmp2b	0.00	VARS	0.00	Actr2	0.09
Hdlbp	0.00	DMP4	0.00	Sptan1	0.09
Nudcd2	0.00	Spata5	0.00	Ap1g1	0.08
Arfip2	0.00	Ap1g1	0.00	Srp72	0.08
Wdr6	0.00	Bzrap1	0.00	Trap1	0.08
Lars	0.00	Ap2a2	0.00	Cnrip1	0.08
Psmc2	0.00	Apc	0.00	Nsun2	0.08
Snrnp200	0.00	Kdm2a	0.00	Gmps	0.08
Fubp1	0.00	Tpr	0.00	Kif5b	0.08
Psmc5	0.00	Man2c1	0.00	PDCD5	0.08
Aldh2	0.00	Ipo9	0.00	VARS	0.08
Ddx21	0.00	Xirp2	0.00	Impdh	0.08

Craniofacial development protein 1	Cfdp1	0.000	0.004	0.170
Citrate synthase	Cs	0.000	0.004	0.304
Coatomer subunit beta	Copb1	0.000	0.003	0.133
Phosphopantothenate--cysteine ligase	Ppcs	0.000	0.005	0.068
Transcription factor BTF3-like 4	Btf3l4	0.000	0.008	0.184
Eukaryotic translation initiation factor 3 subunit	EIF3M	0.000	0.004	0.337
Isopentenyl-diphosphate Delta-isomerase 1	Idi1	0.000	0.037	0.233
Prefoldin subunit 5	Pfdn5	0.000	0.080	0.618
LIM and SH3 domain protein 1	LASP1	0.000	0.010	0.161
Ras-related protein Rab-2B	Rab2b	0.000	0.005	0.121
Proteasome subunit beta type-6	Psmb6	0.000	0.026	0.168
Cytochrome b5	Cyb5	0.000	0.016	0.400
26S proteasome non-ATPase regulatory subunit	Psmd7	0.000	0.004	0.058
[Protein ADP-ribosylarginine] hydrolase	Adprh	0.000	0.007	0.135
Large neutral amino acids transporter small subu	Slc7a5	0.000	0.019	0.026
A-kinase anchor protein 13	Akap13	0.000	0.000	0.003
6-phosphofructokinase	PFK	0.000	0.002	0.047
Actin-related protein 2	Actr2	0.000	0.014	0.088
Proteasome-associated protein ECM29-like	ECM29	0.000	0.001	0.013
Spermatogenesis-associated protein 5	Spata5	0.000	0.001	0.003
SHC-transforming protein 1	Shc1	0.000	0.003	0.009
60S ribosomal protein L7	Rpl7	0.000	0.005	0.042
Serine/threonine-protein phosphatase	Ppp	0.000	0.005	0.042
CB1 cannabinoid receptor-interacting protein 1	Cnrip1	0.000	0.007	0.082
Cytoplasmic dynein 2 heavy chain 1	Dync2h1	0.000	0.004	0.000
ADP-ribosylation factor 5	Arf5	0.000	0.017	0.000
Melanoma-associated antigen D2	MAGED2	0.000	0.007	0.000
60S ribosomal protein L6	Rpl6	0.000	0.001	0.000
Cadherin-23	Cdh23	0.000	0.002	0.000
Type I inositol-3,4-bisphosphate 4-phosphatase	Inpp4a	0.000	0.003	0.000
Elongation factor 1-alpha	Eef1a	0.000	0.004	0.000
Lysosome-associated membrane glycoprotein 1	Lamp1	0.000	0.004	0.000
H-2 class II histocompatibility antigen gamma ch	Cd74	0.000	0.004	0.000
SH3 domain-containing RING finger protein 3 (F	SH3RF3	0.000	0.004	0.000
L-lactate dehydrogenase	LDH	0.000	0.005	0.000
PHD finger protein 1	Phf1	0.000	0.005	0.000
40S ribosomal protein S2	Rps2	0.000	0.008	0.000
WD repeat-containing protein 40A (Fragment)	Dcaf12	0.000	0.008	0.000
Ras-related protein Rab-35	Rab35	0.000	0.009	0.000
Plexin-B2	Plxnb2	0.000	0.015	0.000
Ectonucleotide pyrophosphatase/phosphodiester	Enpp6	0.000	0.016	0.000
Plasma membrane calcium-transporting ATPase	Atp2b1	0.000	0.013	0.000
Envelope glycoprotein	Gp	0.004	0.010	0.000
Beta-1,4-galactosyltransferase 1	B4galt1	0.008	0.052	0.000
Multidrug resistance protein 1	ABCB1	0.000	0.002	0.000
Serine/threonine-protein phosphatase 4 regulato	Ppp4r3b	0.000	0.003	0.000
Ras-related protein Rab-8B	Rab8b	0.000	0.012	0.000
Coiled-coil domain-containing protein 85A	Ccdc85a	0.000	0.054	0.000
Protein NDRG1	Ndrg1	0.000	0.010	0.000
Semaphorin-6C	Sema6c	0.000	0.011	0.000
Ras-related protein R-Ras2 (Fragment)	Rras2	0.000	0.023	0.000
Lysine-specific demethylase 2A	Kdm2a	0.000	0.001	0.000
Stress-induced-phosphoprotein 1	Stip1	0.000	0.002	0.000
Proto-oncogene tyrosine-protein kinase Yes	YES1	0.000	0.002	0.000
Myosin-binding protein C, slow-type	Mybpc1	0.000	0.002	0.000
Lupus La protein-like	Ssb	0.000	0.003	0.000
Cytochrome P450 4F4	Cyp4f4	0.000	0.003	0.000
PR domain zinc finger protein 15	PRDM15	0.000	0.003	0.000
Putative Polycomb group protein ASXL1	Asxl1	0.000	0.003	0.000
tRNA-dihydrouridine(47) synthase [NAD(P)(+)]	DUS3L	0.000	0.003	0.000
40S ribosomal protein S2	Rps2	0.000	0.004	0.000
60S ribosomal protein L6	Rpl6	0.000	0.004	0.000
Histone H3	Hist1h3a	0.000	0.005	0.000
Histone H4	Hist1h4a	0.000	0.006	0.000
Histone H2A	His2A	0.000	0.009	0.000
L-lactate dehydrogenase A chain	Ldha	0.000	0.010	0.000
40S ribosomal protein S2	Rps2	0.000	0.011	0.000
Adenine phosphoribosyltransferase	Aprt	0.000	0.012	1.028
Gamma-glutamylcyclotransferase	Ggct	0.000	0.008	0.167
Actin, gamma-enteric smooth muscle (Fragment)	Actg2	0.000	0.003	0.022
Heme oxygenase 1	Hmox1	0.000	0.004	0.674
Guanine nucleotide-binding protein subunit beta	GNB2L1	0.024	0.282	1.093
C-X-C motif chemokine 3	Cxcr3	0.071	0.343	1.310
Elongation factor 1-beta	Eef1b	0.026	0.254	1.349
Asparagine synthetase [glutamine-hydrolyzing]	Asns	0.000	0.008	0.207
14-3-3 protein epsilon	Ywhae	0.200	1.409	5.898
Proteasome activator complex subunit 3	PSME3	0.000	0.005	0.322
Lamin-L(I)	lmnl1	0.000	0.032	0.301
Alpha-mannosidase 2C1	Man2c1	0.000	0.001	0.179
Glutathione S-transferase omega-1	Gsto1	0.023	0.130	0.256
Methionyl-tRNA synthetase, cytoplasmic	MARS	0.000	0.002	0.194
Valyl-tRNA synthetase	VARS	0.000	0.002	0.080
Microtubule-associated protein	Map	0.000	0.001	0.173
Serine hydroxymethyltransferase	Shmt1	0.000	0.002	0.283
Asparaginyl-tRNA synthetase, cytoplasmic	NARS	0.000	0.008	0.219
Bifunctional ATP-dependent dihydroxyacetone ki	DAK	0.000	0.003	0.197

Rsl1d1	0.00	ECM29	0.00	Ggh	0.08
Anp32e	0.00	Col5a3	0.00	Fus	0.08
Dhx15	0.00	Map	0.00	Sart3	0.08
Atp5b	0.00	Csmd2	0.00	Atp1a1	0.08
Sf3b1	0.00	Pclo	0.00	Glb1	0.07
Ampd2	0.00	Birc6	0.00	Rap1a	0.07
Clip1	0.00	Myh10	0.00	Cpsf6	0.07
Pcnp	0.00	Map1a	0.00	Ppcs	0.07
Chordc1	0.00	Rpl6	0.00	Umps	0.07
Hsd17b10	0.00	Flnc	0.00	Chmp2b	0.07
Cul5	0.00	Akap13	0.00	Psmc4	0.06
Snrnp70	0.00	Ctnnbl1	0.00	Myh10	0.06
Arhgap1	0.00	Aox	0.00	RecName: Full	0.06
Acot2	0.00	Thoc2	0.00	Ap2a2	0.06
Lyar	0.00	Kif5c	0.00	Xdh	0.06
Nit1	0.00	MTR	0.00	Dync1i2	0.06
Ilf2	0.00	Hdac1	0.00	Ampd2	0.06
Rcc2	0.00	Tubb4a	0.00	Fam98b	0.06
Cbx5	0.00	Ndrg3	0.00	Cdk6	0.06
Nono	0.00	Pcbp3	0.00	Psmd7	0.06
Sec23ip	0.00	Zfhx3	0.00	Mmp12	0.06
Srp72	0.00	Kdr	0.00	Oxct1	0.06
Dync1i2	0.00	Piezo2	0.00	Nckap1	0.06
Iars	0.00	Myrip	0.00	Ctla2b	0.06
Drg1	0.00	Nav1	0.00	Rpsa	0.05
Heatr3	0.00	Hspa1l	0.00	Cttn	0.05
Safb	0.00	OR2T2	0.00	Dnajc9	0.05
Copg	0.00	Ubqln2	0.00	Tsta3	0.05
Sart3	0.00	HNRNPH2	0.00	Cbr2	0.05
Bat3	0.00	Actr1b	0.00	Aldh1a1	0.05
Eif4g1	0.00	HNRNPH	0.00	PFK	0.05
Rps27	0.00	Lrfn2	0.00	Arfip2	0.05
Lars	0.00	TAF15	0.00	Hdac1	0.05
Qars	0.00	CERTL	0.00	Creld2	0.05
Nop2	0.00	Mapre3	0.00	Tpr	0.04
Sec23a	0.00	Casp3	0.00	Clip1	0.04
Impdh	0.00	Mthfd2	0.00	Sec23ip	0.04
Cpsf6	0.00	Nmt	0.00	Ppp	0.04
Oxct1	0.00	Arf4	0.00	Ube2v2	0.04
Aldh1a1	0.00	Ppie	0.00	Bat3	0.04
Lsm4	0.00	Eif4e	0.00	Mapk1	0.04
Srsf1	0.00	PPP6C	0.00	Rpl7	0.04
Golga5	0.00	Fam107b	0.00	Sf3b1	0.04
Ak3	0.00	EIF5	0.00	Ppp5c	0.04
Opa1	0.00	Pdlim5	0.00	Safb	0.04
Rps17	0.00	Ube2v2	0.00	Pdlim5	0.04
Psmd3	0.00	Ctla2b	0.00	Idh3a	0.04
Ckap5	0.00	Hdac1	0.00	Ctnnbl1	0.04
Nsun2	0.00	Tnpo1	0.00	Ppp	0.04
Prmt5	0.00	Hsf4	0.00	EIF5	0.04
Cyfip1	0.00	Myo5b	0.00	Fak1	0.04
Puf60	0.00	Eif3e	0.00	Tnpo1	0.03
Fak1	0.00	Arpc1b	0.00	Hsf4	0.03
Kif5b	0.00	Dph1	0.00	Hdlbp	0.03
Snx6	0.00	Gstm5	0.00	Map2k1	0.03
Thumpd1	0.00	Tcof1	0.00	Dph1	0.03
Snrpf	0.00	Ppfia1	0.00	Nmt	0.03
Calm	0.00	Ppp2r2b	0.00	Fam107b	0.03
Map2k1	0.00	Hnrnpdl	0.00	Cyfip1	0.03
Paics	0.00	Cttn	0.00	Lima1	0.03
Psmc4	0.00	Dnm	0.00	Nrp1	0.03
Tsta3	0.00	Fam98b	0.00	Paics	0.03
Hypk	0.00	Cbr2	0.00	Rps21	0.03
Dnajc9	0.00	Gnpda2	0.00	Cpne3	0.03
prdF	0.00	Ube2m	0.00	Klc1	0.03
Cnbp	0.00	Cxorf26-like	0.00	Eif4g1	0.03
Cpne3	0.00	Rap1a	0.00	Wdr6	0.03
Ubqln1	0.00	Ppp	0.00	Ehd3	0.03
Kpna2	0.00	Pdap1	0.00	Ppp2r2b	0.03
Col6a5	0.00	Acin1	0.00	prdF	0.03
Iqgap2	0.00	Chmp2b	0.00	Ctps	0.03
THBS4	0.00	Hdlbp	0.00	Slc7a5	0.03
PPP1R12	0.00	Nudcd2	0.00	Golga5	0.03
Chd2	0.00	Arfip2	0.00	Serpinh1	0.03
GRLF1	0.00	Wdr6	0.00	Srsf7	0.03
Mapk8ip4	0.00	Lars	0.00	Ktn1	0.02
Gart	0.00	Psmc2	0.00	Ube2v1	0.02
Tuba8	0.00	Snrnp200	0.00	Stip1	0.02
DARS	0.00	Fubp1	0.00	Opa1	0.02
CDK9	0.00	Psmc5	0.00	PPP6C	0.02
Fth1	0.00	Aldh2	0.00	St14	0.02
Pcbp2	0.00	Ddx21	0.00	Actg2	0.02
Osbpl6	0.00	Rsl1d1	0.00	Eif4e	0.02
Ppp1r9b	0.00	Anp32e	0.00	Gart	0.02
Fam26d	0.00	Dhx15	0.00	Dnm	0.02
Bclaf1	0.00	Atp5b	0.00	Pcbp3	0.02

Aldose reductase-related protein 2	Akr1b8	0.029	0.248	0.326
Glutathione S-transferase Mu 1	Gstm1	0.000	0.007	0.255
Sister chromatid cohesion protein PDS5-like A	PDS5A	0.000	0.000	0.005
Ryanodine receptor 2	Ryr2	0.000	0.000	0.002
Rho guanine nucleotide exchange factor 7	Arhgef7	0.000	0.000	0.004
Pyridoxal-dependent decarboxylase domain-cont	Pdxdc1	0.000	0.000	0.020
MAM domain-containing protein 2	Mamdc2	0.057	0.354	0.094
Serine/arginine repetitive matrix protein 2	Srrm2	0.000	0.000	0.016
Transketolase	Tkt	0.049	0.382	1.668
Exportin-2	Cse1l	0.000	0.000	0.206
FACT complex subunit SPT16	Supt16h	0.000	0.000	0.112
Soluble calcium-activated nucleotidase 1	Cant1	0.071	0.375	0.250
Lactadherin (Fragment)	MFGE8	0.083	0.432	0.398
Heterogeneous nuclear ribonucleoprotein U-like	Hnrnpul2	0.000	0.000	0.183
Actin, cytoplasmic 1	Actb	0.034	0.338	1.505
NSFL1 cofactor p47	Nsfl1c	0.018	0.158	0.628
Gamma-enolase	Eno2	0.000	0.000	0.133
Atrial natriuretic factor	Anf	0.017	0.052	0.298
PDZ and LIM domain protein 1	Pdlim1	0.001	0.011	0.145
Heat shock protein HSP 90-alpha	Hsp90aa1	0.020	0.123	0.241
Lamin-A/C	LMNA	0.018	0.186	0.845
GMP synthase [glutamine-hydrolyzing]	Gmps	0.000	0.000	0.081
Amyloid beta A4 protein	App	0.076	0.634	0.538
Mitotic checkpoint protein BUB3	Bub3	0.001	0.000	0.195
Reticulocalbin-3	Rcn3	0.027	0.151	0.382
Heat shock protein HSP 90-alpha	Hsp90aa1	0.010	0.051	0.158
Macrophage metalloelastase	Mmp12	0.000	0.000	0.058
Aldose reductase	Akr1b1	0.038	0.310	0.890
Bone morphogenetic protein 1	BMP1	0.040	0.185	0.139
Prefoldin subunit 2	Pfdn2	0.001	0.000	0.387
Ribonuclease T2	Rnaset2	0.175	0.891	0.481
Inosine-5'-monophosphate dehydrogenase 2	Impdh2	0.000	0.000	0.148
C-C motif chemokine 7	Ccl7	0.124	0.335	0.135
A-kinase anchor protein 8-like	Akap8l	0.000	0.000	0.003
Diacylglycerol kinase	DGK	0.000	0.000	0.005
Inner centromere protein	Incenp	0.001	0.000	0.000
Keratin, type II cytoskeletal 2 oral	Krt76	0.000	0.000	0.000
Lysine-specific demethylase 2B	Kdm2b	0.000	0.000	0.000
TRAF family member-associated NF-kappa-B act	Tank	0.000	0.000	0.000
Enolase	Eno	0.001	0.000	0.000
Elongation factor 1-alpha 1	Eef1a1	0.001	0.000	0.000
Peptidyl-prolyl cis-trans isomerase A	Ppia	0.002	0.000	0.000
Cysteine-rich motor neuron 1 protein	Crim1	0.001	0.000	0.000
Epiplakin	Eppk1	0.000	0.000	0.000
ATP-binding cassette sub-family A member 1	Abca1	0.000	0.000	0.000
Keratin, type I cytoskeletal 15	Krt15	0.001	0.000	0.000
Carbonyl reductase [NADPH] 1	Cbr1	0.001	0.000	0.000
Caspase 3	Casp3	0.001	0.000	0.000
GTP-binding protein REM 1	Rem1	0.001	0.000	0.000
Pyridoxal kinase	Pdxk	0.003	0.011	0.000
Galectin	Lgals	0.012	0.168	0.000
Protocadherin Fat 1	FAT1	0.007	0.034	0.000
Peroxiredoxin-1	Prdx1	0.113	1.198	4.524
Alpha-enolase	Eno1	0.037	0.275	1.059
F-actin-capping protein subunit beta	Capzb	0.024	0.209	0.757
RNA-binding protein FUS	Fus	0.001	0.000	0.077
Cofilin-1	Cfl1	0.121	0.831	3.296
Endoplasmin	Hsp90b1	0.041	0.297	1.644
Rho GDP-dissociation inhibitor 1	Arhgdia	0.048	0.591	1.799
Clathrin heavy chain	Cltc	0.002	0.060	0.289
14-3-3 protein zeta/delta	Ywhaz	0.053	0.412	1.122
Glutathione synthetase	Gss	0.016	0.178	1.157
Phosphatidylethanolamine-binding protein 1	Pebp1	0.046	0.625	0.962
Phosphoglycerate mutase 1	Pgam1	0.039	0.509	1.586
Calsyntenin-1	Clstn1	0.054	0.374	0.143
Eukaryotic translation initiation factor 3 subunit	EIF3D	0.000	0.000	0.182
Peptidyl-prolyl cis-trans isomerase	Ppi	0.125	0.835	1.511
60S acidic ribosomal protein P2	RPLP2	0.057	0.726	3.060
Phosphoglycerate kinase	Pgk	0.020	0.292	1.375
Nucleolin	Ncl	0.025	0.216	1.134
Heat shock cognate 71 kDa protein	Hspa8	0.019	0.140	0.335
Annexin	ANX	0.084	0.739	1.436
Protein disulfide-isomerase	Pdi	0.041	0.264	0.667
Malate dehydrogenase	M오	0.027	0.218	1.038
Glucose-6-phosphate 1-dehydrogenase	G6pd	0.002	0.007	0.009
Serpin H1	Serpinh1	0.003	0.012	0.025
Xin actin-binding repeat-containing protein 2	Xirp2	0.000	0.001	0.001
Adenomatous polyposis coli protein	Apc	0.000	0.001	0.001
Tenascin-X	Tnxb	0.011	0.067	0.002
Protein-glutamine gamma-glutamyltransferase 2	Tgm2	0.000	0.007	0.363
40S ribosomal protein S3a	Rps3a	0.019	0.095	0.500
15 kDa selenoprotein	Sep15	0.012	0.064	0.124
Acidic leucine-rich nuclear phosphoprotein 32 fa	Anp32b	0.004	0.023	0.225
Ganglioside GM2 activator	Gm2a	0.039	0.198	0.138
EH domain-containing protein 3	Ehd3	0.000	0.022	0.029
Stress-induced-phosphoprotein 1	Stip1	0.002	0.003	0.025

MLLT1	0.00	Sf3b1	0.00	Pdxdc1	0.02
Bst2	0.00	Ampd2	0.00	Ckap5	0.02
Idh3a	0.00	Clip1	0.00	Ppie	0.02
NFU1	0.00	Pcnp	0.00	Ppfia1	0.02
GCSH	0.00	Chordc1	0.00	SET	0.02
Rpl12	0.00	Hsd17b10	0.00	Kpna2	0.02
SET	0.00	Cul5	0.00	Rpl12	0.02
Ube2v1	0.00	Snrnp70	0.00	Srrm2	0.02
Srsf7	0.00	Arhgap1	0.00	GCSH	0.02
Mapk1	0.00	Acot2	0.00	NFU1	0.02
Taldo1	0.00	Lyar	0.00	Arf4	0.02
PDCD5	0.00	Nit1	0.00	Bst2	0.01
Ctps	0.00	Ilf2	0.00	MLLT1	0.01
Umps	0.00	Rcc2	0.00	Myo5b	0.01
Cdk6	0.00	Cbx5	0.00	Tcof1	0.01
Napa	0.00	Nono	0.00	ECM29	0.01
RecName: Full	0.00	Sec23ip	0.00	Acin1	0.01
Aip	0.00	Srp72	0.00	Bclaf1	0.01
Trap1	0.00	Dync1i2	0.00	Tubb4a	0.01
Nmt	0.00	Iars	0.00	Nmt	0.01
Lima1	0.00	Drg1	0.00	Hdac1	0.01
Wdr61	0.00	Heatr3	0.00	Fam26d	0.01
Sec24C	0.00	Safb	0.00	Mthfd2	0.01
Ruvbl1	0.00	Copg	0.00	Shc1	0.01
Otub1	0.00	Sart3	0.00	Casp3	0.01
Xdh	0.00	Bat3	0.00	Mapre3	0.01
Naglu	0.00	Eif4g1	0.00	Ppp1r9b	0.01
Acy1a	0.00	Rps27	0.00	Osbpl6	0.01
Nans	0.00	Lars	0.00	G6pd	0.01
Sept6	0.00	Qars	0.00	MTR	0.01
Nckap1	0.00	Nop2	0.00	CERTL	0.01
Bola1	0.00	Sec23a	0.00	Map1a	0.01
Mybbp1a	0.00	Impdh	0.00	Pcbp2	0.01
Dkc1	0.00	Cpsf6	0.00	Fth1	0.01
Kpna2	0.00	Oxct1	0.00	Ndrg3	0.01
Eftud2	0.00	Aldh1a1	0.00	TAF15	0.01
Akr7a2	0.00	Lsm4	0.00	Lrfn2	0.01
Hadh	0.00	Srsf1	0.00	CDK9	0.01
Pgp	0.00	Golga5	0.00	DARS	0.01
Clta	0.00	Ak3	0.00	Antxr2	0.01
Aco2	0.00	Opa1	0.00	HNRNPH	0.01
Bzrap1	0.00	Rps17	0.00	Actr1b	0.01
C7	0.00	Psmd3	0.00	Tuba8	0.01
Fbxo43	0.00	Ckap5	0.00	HNRNPH2	0.01
Ahcy	0.00	Nsun2	0.00	Kif5c	0.01
Rps21	0.00	Prmt5	0.00	Mapk8ip4	0.01
Ssrp1	0.00	Cyfip1	0.00	Ubqln2	0.01
Copb2	0.00	Puf60	0.00	PDS5A	0.00
Snrpd3	0.00	Fak1	0.00	DGK	0.00
ADK	0.00	Kif5b	0.00	OR2T2	0.00
Timm13	0.00	Snx6	0.00	Ahcy	0.00
Pef1	0.00	Thumpd1	0.00	Hspa1l	0.00
Ktn1	0.00	Snrpf	0.00	Arhgef7	0.00
Klc1	0.00	Calm	0.00	GRLF1	0.00
Map1a	0.00	Map2k1	0.00	DMP4	0.00
Ppp5c	0.00	Paics	0.00	Thoc2	0.00
Glrx3	0.00	Psmc4	0.00	Plxnb2	0.00
Tpr	0.00	Tsta3	0.00	Nav1	0.00
Acly	0.00	Hypk	0.00	Spata5	0.00
Nucb1	0.00	Dnajc9	0.00	Myrip	0.00
Hnrnpu	0.00	prdF	0.00	Chd2	0.00
Ap2a2	0.00	Cnbp	0.00	Akap13	0.00
Pgls	0.00	Cpne3	0.00	PPP1R12	0.00
EIF3F	0.00	Ubqln1	0.00	THBS4	0.00
Metap2	0.00	Kpna2	0.00	Akap8l	0.00
ANX	0.00	Col6a5	0.00	Piezo2	0.00
Cops4	0.00	Iqgap2	0.00	Kdr	0.00
Rpl13	0.00	THBS4	0.00	Tnxb	0.00
Zpr1	0.00	PPP1R12	0.00	Ryr2	0.00
Rtcb	0.00	Chd2	0.00	Fbxo43	0.00
Cope	0.00	GRLF1	0.00	Zfhx3	0.00
Snrpa1	0.00	Mapk8ip4	0.00	C7	0.00
Ipo9	0.00	Gart	0.00	Iqgap2	0.00
Cfdp1	0.00	Tuba8	0.00	Aox	0.00
Cs	0.00	DARS	0.00	Col6a5	0.00
Copb1	0.00	CDK9	0.00	Apc	0.00
Ppcs	0.00	Fth1	0.00	Bzrap1	0.00
Btf3l4	0.00	Pcbp2	0.00	Xirp2	0.00
EIF3M	0.00	Osbpl6	0.00	Taldo1	0.00
Idi1	0.00	Ppp1r9b	0.00	Fn1	0.00
Pfdn5	0.00	Fam26d	0.00	Dync2h1	0.00
LASP1	0.00	Bclaf1	0.00	Arf5	0.00
Rab2b	0.00	MLLT1	0.00	MAGED2	0.00
Psmb6	0.00	Bst2	0.00	Rpl6	0.00
Cyb5	0.00	Idh3a	0.00	Cdh23	0.00
Psmd7	0.00	NFU1	0.00	Inpp4a	0.00

UPF0556 protein C19orf10-like	C19orf10-like	0.021	0.083	0.272
Gamma-glutamyl hydrolase	Ggh	0.025	0.172	0.078
Protein canopy-like 4	Cnpy4	0.001	0.013	0.133
Protein kinase C and casein kinase substrate in r	Pacsin2	0.001	0.025	0.151
40S ribosomal protein SA	Rpsa	0.002	0.005	0.054
Splicing factor U2AF 65 kDa subunit	U2af2	0.001	0.005	0.194
Proteasome subunit beta type	Psmb	0.010	0.188	0.421
Elongation factor 1-delta	Eef1d	0.031	0.215	0.953
Sodium/potassium-transporting ATPase subunit	Atp1a1	0.001	0.046	0.075
Protein disulfide-isomerase A6	Pdia6	0.058	0.287	2.263
Rab GDP dissociation inhibitor beta	Gdi2	0.024	0.193	0.214
Acyl-CoA-binding protein	Dbi	0.046	0.313	0.625
Protein phosphatase 1F	Ppm1f	0.000	0.010	0.172
Actin-related protein 2/3 complex subunit 2	Arpc2	0.001	0.036	0.133
Heterogeneous nuclear ribonucleoprotein Q	Syncrip	0.000	0.009	0.187
14-3-3 protein theta	Ywhaq	0.028	0.279	0.676
Myosin-10	Myh10	0.000	0.001	0.064
Twisted gastrulation protein-like 1	Twsg1	0.032	0.167	0.251
Heterogeneous nuclear ribonucleoprotein A/B	Hnrnpab	0.008	0.096	0.312
Ceroid-lipofuscinosis neuronal protein 5	Cln5	0.020	0.075	0.126
D-dopachrome decarboxylase	Ddt	0.047	0.487	1.005
Syntaxin-12	Stx12	0.001	0.008	0.306
AP-1 complex subunit gamma-1	Ap1g1	0.000	0.001	0.084
Cornifin-A	Sprr1a	0.002	0.062	1.900
Selenoprotein P	Sepp1	0.025	0.170	0.153
N-acetylglucosamine-6-sulfatase	Gns	0.018	0.053	0.306
Protein arginine N-methyltransferase 1	Prmt1	0.007	0.070	0.301
Rho-related GTP-binding protein RhoC	Rhoc	0.001	0.008	0.096
Spectrin alpha chain, brain	Sptan1	0.001	0.030	0.087
Neuropilin-1	Nrp1	0.012	0.096	0.031
Reticulocalbin-1 (Fragment)	RCN1	0.004	0.010	0.116
Plexin-B2	Plxnb2	0.001	0.012	0.003
Dentin matrix protein 4	DMP4	0.000	0.002	0.003
Antrax toxin receptor 2 (Fragment)	Antxr2	0.000	0.007	0.006
Protein piccolo	Pclo	0.000	0.001	0.000
Keratin, type II cytoskeletal 1b	Krt1b	0.001	0.004	0.000
CUB and sushi domain-containing protein 2 (Fra	Csmd2	0.000	0.001	0.000
Pyruvate kinase	Pkm	0.000	0.003	0.000
Keratin, type II cytoskeletal 2 epidermal	Krt2	0.001	0.006	0.000
TGF-beta receptor type III	Tgfbr3	0.017	0.079	0.000
Integral membrane protein 2C	ITM2C	0.003	0.095	0.000
Nuclear receptor coactivator 2	Ncoa2	0.000	0.002	0.000
Filamin-C	Flnc	0.000	0.000	0.000
Baculoviral IAP repeat-containing protein 6	Birc6	0.000	0.001	0.000
Collagen alpha-3(V) chain (Fragment)	Col5a3	0.001	0.001	0.000
Complement component C6	C6	0.000	0.005	0.000
CD276 antigen	Cd276	0.001	0.007	0.000
Tropomyosin alpha-1 chain	Tpm1	0.001	0.064	0.100
Acetyl-CoA acetyltransferase, cytosolic	Acat2	0.020	0.211	0.460
Fructose-bisphosphate aldolase	Aldo	0.041	0.339	1.987
40S ribosomal protein S8	Rps8	0.015	0.152	1.191
Proteasome subunit alpha type	Psma	0.009	0.263	0.735
Protein DJ-1	PARK7	0.038	0.441	1.859
Proliferation-associated protein 2G4	Pa2g4	0.001	0.013	0.557
Alpha-N-acetylgalactosaminidase	Naga	0.039	0.139	0.350
Aspartate aminotransferase	Ast	0.023	0.317	0.930
Cathepsin D	Ctsd	0.061	0.433	1.032
Lysosomal protective protein	Ctsa	0.073	0.326	1.025
Transitional endoplasmic reticulum ATPase	Vcp	0.007	0.021	0.212
40S ribosomal protein S24	Rps24	0.005	0.145	0.821
Microtubule-associated protein RP/EB family me	Mapre1	0.001	0.018	0.384
Hypoxanthine-guanine phosphoribosyltransferas	Hprt1	0.020	0.247	0.678
Eukaryotic translation initiation factor 6	EIF6	0.012	0.077	0.595
Salivary plasminogen activator alpha 2	Klk1b3	0.024	0.054	0.180
FK506-binding protein 10	Fkbp10	0.000	0.002	0.254
Phosphatidylinositol transfer protein alpha isofor	Pitpna	0.009	0.098	0.250
Actin-related protein 2/3 complex subunit 4	Arpc4	0.009	0.056	0.346

Adprh	0.00	GCSH	0.00	Eef1a	0.00
Slc7a5	0.00	Rpl12	0.00	Lamp1	0.00
Akap13	0.00	SET	0.00	Cd74	0.00
PFK	0.00	Ube2v1	0.00	SH3RF3	0.00
Actr2	0.00	Srsf7	0.00	LDH	0.00
ECM29	0.00	Mapk1	0.00	Phf1	0.00
Spata5	0.00	PDCD5	0.00	Rps2	0.00
Shc1	0.00	Ctps	0.00	Dcaf12	0.00
Rpl7	0.00	Umps	0.00	Rab35	0.00
Ppp	0.00	Cdk6	0.00	Plxnb2	0.00
Cnrip1	0.00	Napa	0.00	Enpp6	0.00
Dync2h1	0.00	RecName: Full	0.00	Atp2b1	0.00
Arf5	0.00	Aip	0.00	Gp	0.00
MAGED2	0.00	Trap1	0.00	B4galt1	0.00
Rpl6	0.00	Nmt	0.00	ABCB1	0.00
Cdh23	0.00	Lima1	0.00	Ppp4r3b	0.00
Inpp4a	0.00	Wdr61	0.00	Rab8b	0.00
Eef1a	0.00	Sec24C	0.00	Ccdc85a	0.00
Lamp1	0.00	Ruvbl1	0.00	Ndrg1	0.00
Cd74	0.00	Otub1	0.00	Sema6c	0.00
SH3RF3	0.00	Xdh	0.00	Rras2	0.00
LDH	0.00	Naglu	0.00	Kdm2a	0.00
Phf1	0.00	Nans	0.00	Stip1	0.00
Rps2	0.00	Sept6	0.00	YES1	0.00
Dcaf12	0.00	Nckap1	0.00	Mybpc1	0.00
Rab35	0.00	Bola1	0.00	Ssb	0.00
Plxnb2	0.00	Mybbp1a	0.00	Cyp4f4	0.00
Enpp6	0.00	Dkc1	0.00	PRDM15	0.00
Atp2b1	0.00	Kpna2	0.00	Asxl1	0.00
ABCB1	0.00	Eftud2	0.00	DUS3L	0.00
Ppp4r3b	0.00	Akr7a2	0.00	Rps2	0.00
Rab8b	0.00	Hadh	0.00	Rpl6	0.00
Ccdc85a	0.00	Pgp	0.00	Hist1h3a	0.00
Ndrg1	0.00	Clta	0.00	Hist1h4a	0.00
Sema6c	0.00	Aco2	0.00	His2A	0.00
Rras2	0.00	PDS5A	0.00	Ldha	0.00
Kdm2a	0.00	Ryr2	0.00	Rps2	0.00
Stip1	0.00	Arhgef7	0.00	Incenp	0.00
YES1	0.00	Pdxdc1	0.00	Krt76	0.00
Mybpc1	0.00	Srrm2	0.00	Kdm2b	0.00
Ssb	0.00	Cse1l	0.00	Tank	0.00
Cyp4f4	0.00	Supt16h	0.00	Eno	0.00
PRDM15	0.00	Hnrnpul2	0.00	Eef1a1	0.00
Asxl1	0.00	Eno2	0.00	Ppia	0.00
DUS3L	0.00	Gmps	0.00	Crim1	0.00
Rps2	0.00	Bub3	0.00	Eppk1	0.00
Rpl6	0.00	Mmp12	0.00	Abca1	0.00
Hist1h3a	0.00	Pfdn2	0.00	Krt15	0.00
Hist1h4a	0.00	Impdh2	0.00	Cbr1	0.00
His2A	0.00	Akap8l	0.00	Casp3	0.00
Ldha	0.00	DGK	0.00	Rem1	0.00
Rps2	0.00	Incenp	0.00	Pdxk	0.00
Aprt	0.00	Krt76	0.00	Lgals	0.00
Ggct	0.00	Kdm2b	0.00	FAT1	0.00
Actg2	0.00	Tank	0.00	Pclo	0.00
Hmox1	0.00	Eno	0.00	Krt1b	0.00
Asns	0.00	Eef1a1	0.00	Csmd2	0.00
PSME3	0.00	Ppia	0.00	Pkm	0.00
Imnl1	0.00	Crim1	0.00	Krt2	0.00
Man2c1	0.00	Eppk1	0.00	Tgfbr3	0.00
MARS	0.00	Abca1	0.00	ITM2C	0.00
VARS	0.00	Krt15	0.00	Ncoa2	0.00
Map	0.00	Cbr1	0.00	Flnc	0.00
Shmt1	0.00	Casp3	0.00	Birc6	0.00
NARS	0.00	Rem1	0.00	Col5a3	0.00
DAK	0.00	Fus	0.00	C6	0.00
Gstm1	0.00	EIF3D	0.00	Cd276	0.00

Day3_Top 30_CC				
Term	Count	%	P-Value	Benjamini
extracellular region	19	76	5.20E-14	4.40E-12
extracellular region part	14	56	5.90E-12	2.50E-10
proteinaceous extracellular matrix	7	28	3.20E-06	8.90E-05
extracellular matrix	7	28	4.00E-06	8.40E-05
extracellular space	8	32	5.40E-06	9.10E-05
basement membrane	4	16	1.60E-04	2.20E-03
extracellular matrix part	4	16	3.10E-04	3.70E-03
cell surface	4	16	9.40E-03	9.40E-02
lysosome	3	12	2.70E-02	2.30E-01
lytic vacuole	3	12	2.70E-02	2.10E-01
vacuole	3	12	3.50E-02	2.40E-01
external side of plasma membrane	3	12	3.60E-02	2.20E-01

Day8_Top 30_CC

Term	Count	%	P-Value	Benjamini
extracellular region	18	75	3.90E-13	2.60E-11
extracellular region part	11	45.8	2.70E-08	9.20E-07
proteinaceous extracellular matrix	7	29.2	2.40E-06	5.30E-05
basement membrane	5	20.8	2.80E-06	4.80E-05
extracellular matrix	7	29.2	3.00E-06	4.00E-05
extracellular matrix part	5	20.8	7.20E-06	8.00E-05
extracellular space	4	16.7	3.30E-02	2.70E-01
cell surface	3	12.5	6.60E-02	4.40E-01

Day12_Top 30				
Term	Count	%	P-Value	Benjamini
melanosome	5	21.7	2.80E-06	1.90E-04
pigment granule	5	21.7	2.80E-06	1.90E-04
extracellular region	11	47.8	6.50E-06	2.20E-04
cytoplasmic membrane-bounded vesicle	5	21.7	1.30E-03	2.80E-02
membrane-bounded vesicle	5	21.7	1.40E-03	2.20E-02
cytoplasmic vesicle	5	21.7	2.80E-03	3.60E-02
vesicle	5	21.7	3.00E-03	3.20E-02
endoplasmic reticulum lumen	3	13	3.30E-03	3.10E-02
extracellular region part	5	21.7	1.20E-02	9.60E-02
cytoplasmic part	11	47.8	1.30E-02	9.30E-02
endoplasmic reticulum part	3	13	3.10E-02	1.90E-01
cytoplasm	13	56.5	4.10E-02	2.20E-01
proteinaceous extracellular matrix	3	13	4.80E-02	2.40E-01
cortical cytoskeleton	2	8.7	5.00E-02	2.30E-01
extracellular matrix	3	13	5.20E-02	2.20E-01
cell cortex part	2	8.7	8.40E-02	3.20E-01
basement membrane	2	8.7	8.40E-02	3.20E-01

Clustering analysis				
Cluster 1	Protein ID	day3	day8	day12
	Ctnnbl1	0.000	0.000	0.037
	Aox	0.000	0.000	0.002
	Thoc2	0.000	0.000	0.003
	Kif5c	0.000	0.000	0.005
	MTR	0.000	0.000	0.009
	Hdac1	0.000	0.000	0.011
	Tubb4a	0.000	0.000	0.012
	Ndrg3	0.000	0.000	0.008
	Pcbp3	0.000	0.000	0.021
	Zfhx3	0.000	0.000	0.002
	Kdr	0.000	0.000	0.002
	Piezo2	0.000	0.000	0.002
	Myrip	0.000	0.000	0.003
	Nav1	0.000	0.000	0.003
	Hspa1l	0.000	0.000	0.004
	OR2T2	0.000	0.000	0.005
	Ubqln2	0.000	0.000	0.005
	HNRNPH2	0.000	0.000	0.006
	Actr1b	0.000	0.000	0.006
	HNRNPH	0.000	0.000	0.006
	Lrfn2	0.000	0.000	0.007
	TAF15	0.000	0.000	0.007
	CERTL	0.000	0.000	0.008
	Mapre3	0.000	0.000	0.009
	Casp3	0.000	0.000	0.009
	Mthfd2	0.000	0.000	0.010
	Nmt	0.000	0.000	0.012
	Arf4	0.000	0.000	0.015
	Ppie	0.000	0.000	0.018
	Eif4e	0.000	0.000	0.022
	PPP6C	0.000	0.000	0.023
	Fam107b	0.000	0.000	0.032
	EIF5	0.000	0.000	0.036
	Pdlim5	0.000	0.000	0.037
	Ube2v2	0.000	0.000	0.042
	Ctla2b	0.000	0.000	0.055
	Hdac1	0.000	0.000	0.046
	Tnpo1	0.000	0.000	0.035
	Hsf4	0.000	0.000	0.034
	Myo5b	0.000	0.000	0.013
	Eif3e	0.000	0.000	0.141
	Arpc1b	0.000	0.000	0.092
	Dph1	0.000	0.000	0.032
	Gstm5	0.000	0.000	0.093
	Tcof1	0.000	0.000	0.013
	Ppfia1	0.000	0.000	0.018
	Ppp2r2b	0.000	0.000	0.029
	Hnrnpdl	0.000	0.000	0.116
	Cttn	0.000	0.000	0.053
	Dnm	0.000	0.000	0.021
	Fam98b	0.000	0.000	0.059
	Cbr2	0.000	0.000	0.048

Gnpda2	0.000	0.000	0.169
Ube2m	0.000	0.000	0.131
Cxorf26-like	0.000	0.000	0.093
Rap1a	0.000	0.000	0.072
Ppp	0.000	0.000	0.036
Pdap1	0.000	0.000	0.147
Acin1	0.000	0.000	0.013
Chmp2b	0.000	0.000	0.065
Hdlbp	0.000	0.000	0.033
Nudcd2	0.000	0.000	0.167
Arfip2	0.000	0.000	0.046
Wdr6	0.000	0.000	0.029
Lars	0.000	0.000	0.211
Psmc2	0.000	0.000	0.323
Snrnp200	0.000	0.000	0.088
Fubp1	0.000	0.000	0.201
Psmc5	0.000	0.000	0.168
Aldh2	0.000	0.000	0.163
Ddx21	0.000	0.000	0.157
Rsl1d1	0.000	0.000	0.150
Anp32e	0.000	0.000	0.426
Dhx15	0.000	0.000	0.105
Atp5b	0.000	0.000	0.224
Sf3b1	0.000	0.000	0.041
Ampd2	0.000	0.000	0.060
Clip1	0.000	0.000	0.043
Pcnp	0.000	0.000	0.210
Chordc1	0.000	0.000	0.150
Hsd17b10	0.000	0.000	0.170
Cul5	0.000	0.000	0.098
Snrnp70	0.000	0.000	0.089
Arhgap1	0.000	0.000	0.170
Acot2	0.000	0.000	0.122
Lyar	0.000	0.000	0.200
Nit1	0.000	0.000	0.299
Ilf2	0.000	0.000	0.915
Rcc2	0.000	0.000	0.145
Cbx5	0.000	0.000	0.195
Nono	0.000	0.000	0.095
Sec23ip	0.000	0.000	0.042
Srp72	0.000	0.000	0.083
Dync1i2	0.000	0.000	0.060
Iars	0.000	0.000	0.121
Drg1	0.000	0.000	0.308
Heatr3	0.000	0.000	0.104
Safb	0.000	0.000	0.037
Copg	0.000	0.000	0.106
Sart3	0.000	0.000	0.075
Bat3	0.000	0.000	0.042
Eif4g1	0.000	0.000	0.030
Rps27	0.000	0.000	0.252
Lars	0.000	0.000	0.134
Qars	0.000	0.000	0.154
Nop2	0.000	0.000	0.137
Sec23a	0.000	0.000	0.118
Impdh	0.000	0.000	0.078
Cpsf6	0.000	0.000	0.068
Oxct1	0.000	0.000	0.056
Aldh1a1	0.000	0.000	0.047
Lsm4	0.000	0.000	0.149
Srsf1	0.000	0.000	0.265
Golga5	0.000	0.000	0.025
Ak3	0.000	0.000	0.139
Opa1	0.000	0.000	0.023
Rps17	0.000	0.000	0.413
Psmd3	0.000	0.000	0.401
Ckap5	0.000	0.000	0.019
Nsun2	0.000	0.000	0.081
Prmt5	0.000	0.000	0.213
Cyfip1	0.000	0.000	0.032
Puf60	0.000	0.000	0.147
Fak1	0.000	0.000	0.036
Kif5b	0.000	0.000	0.081
Snx6	0.000	0.000	0.104
Thumpd1	0.000	0.000	0.133
Snrpf	0.000	0.000	0.321
Calm	0.000	0.000	0.182
Map2k1	0.000	0.000	0.033
Paics	0.000	0.000	0.031
Psmc4	0.000	0.000	0.064
Tsta3	0.000	0.000	0.049
Hypk	0.000	0.000	0.130
Dnajc9	0.000	0.000	0.049
prdF	0.000	0.000	0.028
Cnbp	0.000	0.000	0.090
Cpne3	0.000	0.000	0.030

	Ubqln1	0.000	0.000	0.142
	Kpna2	0.000	0.000	0.017
	Col6a5	0.000	0.000	0.002
	Iqgap2	0.000	0.000	0.002
	THBS4	0.000	0.000	0.003
	PPP1R12	0.000	0.000	0.003
	Chd2	0.000	0.000	0.003
	GRLF1	0.000	0.000	0.004
	Mapk8ip4	0.000	0.000	0.005
	Gart	0.000	0.000	0.021
	Tuba8	0.000	0.000	0.006
	DARS	0.000	0.000	0.006
	CDK9	0.000	0.000	0.007
	Fth1	0.000	0.000	0.008
	Pcbp2	0.000	0.000	0.008
	Osbpl6	0.000	0.000	0.009
	Ppp1r9b	0.000	0.000	0.009
	Fam26d	0.000	0.000	0.011
	Bclaf1	0.000	0.000	0.013
	MLLT1	0.000	0.000	0.013
	Bst2	0.000	0.000	0.014
	Idh3a	0.000	0.000	0.037
	NFU1	0.000	0.000	0.016
	GCSH	0.000	0.000	0.016
	Rpl12	0.000	0.000	0.016
	SET	0.000	0.000	0.018
	Ube2v1	0.000	0.000	0.025
	Srsf7	0.000	0.000	0.025
	Mapk1	0.000	0.000	0.042
	PDCD5	0.000	0.000	0.081
	Ctps	0.000	0.000	0.027
	Umps	0.000	0.000	0.066
	Cdk6	0.000	0.000	0.058
	Napa	0.000	0.000	0.154
	RecName: Full	0.000	0.000	0.064
	Aip	0.000	0.000	0.112
	Trap1	0.000	0.000	0.082
	Nmt	0.000	0.000	0.032
	Lima1	0.000	0.000	0.031
	Wdr61	0.000	0.000	0.200
	Sec24C	0.000	0.000	0.148
	Ruvbl1	0.000	0.000	0.508
	Otub1	0.000	0.000	0.253
	Xdh	0.000	0.000	0.061
	Naglu	0.000	0.000	0.118
	Nans	0.000	0.000	0.154
	Sept6	0.000	0.000	0.134
	Nckap1	0.000	0.000	0.055
	Bola1	0.000	0.000	0.595
	Mybbp1a	0.000	0.000	0.173
	Dkc1	0.000	0.000	0.215
	Kpna2	0.000	0.000	0.155
	Eftud2	0.000	0.000	0.117
	Akr7a2	0.000	0.000	0.196
	Hadh	0.000	0.000	0.253
	Pgp	0.000	0.000	0.103
	Clta	0.000	0.000	0.304
	Aco2	0.000	0.000	0.146
	Srrm2	0.000	0.000	0.016
	Ryr2	0.000	0.000	0.002
	Eppk1	0.000	0.000	0.000
	DGK	0.000	0.000	0.005
	Gmps	0.000	0.000	0.081
	Abca1	0.000	0.000	0.000
	PDS5A	0.000	0.000	0.005
	Akap8l	0.000	0.000	0.003
	Cse1l	0.000	0.000	0.206
	Supt16h	0.000	0.000	0.112
	Pdxdc1	0.000	0.000	0.020
	Krt76	0.000	0.000	0.000
	Arhgef7	0.000	0.000	0.004
	Kdm2b	0.000	0.000	0.000
	Hnrnpul2	0.000	0.000	0.183
	EIF3D	0.000	0.000	0.182
	Mmp12	0.000	0.000	0.058
	Eno2	0.000	0.000	0.133
	Tank	0.000	0.000	0.000
	Impdh2	0.000	0.000	0.148
	Akap13	0.000	0.000	0.003
	Flnc	0.000	0.000	0.000
Cluster 2	Protein ID	day3	day8	day12
	Pfdn2	0.001	0.000	0.387
	Bub3	0.001	0.000	0.195
	Map	0.000	0.001	0.173
	Man2c1	0.000	0.001	0.179
	Fkbp10	0.000	0.002	0.254

Hmox1	0.000	0.004	0.674
Ipo9	0.000	0.001	0.136
Myh10	0.000	0.001	0.064
Shmt1	0.000	0.002	0.283
MARS	0.000	0.002	0.194
Cope	0.000	0.004	0.420
Pgls	0.000	0.007	0.735
Glrx3	0.000	0.003	0.325
Aprt	0.000	0.012	1.028
Fus	0.001	0.000	0.077
Cs	0.000	0.004	0.304
EIF3M	0.000	0.004	0.337
DAK	0.000	0.003	0.197
Copb2	0.000	0.002	0.144
Ap1g1	0.000	0.001	0.084
PSME3	0.000	0.005	0.322
Rtcb	0.000	0.003	0.198
Ssrp1	0.000	0.003	0.204
Acly	0.000	0.003	0.201
Snrpa1	0.000	0.013	0.785
Metap2	0.000	0.002	0.132
Tgm2	0.000	0.007	0.363
VARS	0.000	0.002	0.080
Hnrnpu	0.000	0.002	0.118
Copb1	0.000	0.003	0.133
Ap2a2	0.000	0.001	0.062
EIF3F	0.000	0.006	0.285
Pa2g4	0.001	0.013	0.557
Cops4	0.000	0.006	0.262
Nucb1	0.000	0.006	0.302
ANX	0.000	0.003	0.106
Stx12	0.001	0.008	0.306
U2af2	0.001	0.005	0.194
Tpr	0.000	0.001	0.045
Cfdp1	0.000	0.004	0.170
Gstm1	0.000	0.007	0.255
Sprr1a	0.002	0.062	1.900
NARS	0.000	0.008	0.219
Asns	0.000	0.008	0.207
Cyb5	0.000	0.016	0.400
Rab2b	0.000	0.005	0.121
Btf3l4	0.000	0.008	0.184
Pef1	0.000	0.005	0.106
Mapre1	0.001	0.018	0.384
PFK	0.000	0.002	0.047
Ggct	0.000	0.008	0.167
Syncrip	0.000	0.009	0.187
Acy1a	0.000	0.009	0.195
Adprh	0.000	0.007	0.135
RCN1	0.004	0.010	0.116
Zpr1	0.000	0.011	0.205
Ppm1f	0.000	0.010	0.172
Rpl13	0.000	0.022	0.375
LASP1	0.000	0.010	0.161
Stip1	0.002	0.003	0.025
Psmd7	0.000	0.004	0.058
Rpsa	0.002	0.005	0.054
Pdlim1	0.001	0.011	0.145
ADK	0.000	0.007	0.102
Klc1	0.000	0.002	0.030
Rhoc	0.001	0.008	0.096
Ppcs	0.000	0.005	0.068
Vcp	0.007	0.021	0.212
ECM29	0.000	0.001	0.013
Map1a	0.000	0.001	0.008
Cnrip1	0.000	0.007	0.082
Ktn1	0.000	0.002	0.025
Anp32b	0.004	0.023	0.225
Cnpy4	0.001	0.013	0.133
Pdia6	0.058	0.287	2.263
Imnl1	0.000	0.032	0.301
Ppp5c	0.000	0.004	0.038
EIF6	0.012	0.077	0.595
Rps8	0.015	0.152	1.191
Rpl7	0.000	0.005	0.042
Gns	0.018	0.053	0.306
Ppp	0.000	0.005	0.042
Anf	0.017	0.052	0.298
Vcl	0.012	0.118	0.861
Pfdn5	0.000	0.080	0.618
Arpc4	0.009	0.056	0.346
Actg2	0.000	0.003	0.022
Gss	0.016	0.178	1.157
Aldo	0.041	0.339	1.987
Psmb6	0.000	0.026	0.168
Rps3a	0.019	0.095	0.500

Timm13	0.000	0.092	0.580
Actr2	0.000	0.014	0.088
Hsp90b1	0.041	0.297	1.644
Idi1	0.000	0.037	0.233
Pacsin2	0.001	0.025	0.151
Snrpd3	0.000	0.067	0.406
Gstp	0.047	0.316	1.650
Rps24	0.005	0.145	0.821
Eef1b	0.026	0.254	1.349
Ncl	0.025	0.216	1.134
Pgk	0.050	0.336	1.669
Npm1	0.033	0.207	1.007
Klk1b3	0.024	0.054	0.180
M오	0.027	0.218	1.038
Hspa5	0.083	0.586	2.629
Eef1d	0.031	0.215	0.953
Pgk	0.020	0.292	1.375
Cltc	0.002	0.060	0.289
LMNA	0.018	0.186	0.845
Tkt	0.049	0.382	1.668
Actb	0.034	0.338	1.505
Ywhae	0.200	1.409	5.898
Prmt1	0.007	0.070	0.301
Actn1	0.023	0.173	0.719
Capg	0.014	0.167	0.718
Creld2	0.004	0.013	0.046
Cxcr3	0.071	0.343	1.310
Ldh	0.015	0.169	0.715
PARK7	0.038	0.441	1.859
RPLP2	0.057	0.726	3.060
Cfl1	0.121	0.831	3.296
Nsfl1c	0.018	0.158	0.628
Eno1	0.037	0.275	1.059
COL3A1	0.015	0.066	0.232
PSAT	0.040	0.477	1.871
GNB2L1	0.024	0.282	1.093
Pkm	0.114	0.905	3.392
C19orf10-like	0.021	0.083	0.272
Prdx1	0.113	1.198	4.524
Prkcsh	0.016	0.074	0.251
Capzb	0.024	0.209	0.757
Ctsa	0.073	0.326	1.025
Nudc	0.018	0.189	0.664
Arpc2	0.001	0.036	0.133
Shc1	0.000	0.003	0.009
Hsp90aa1	0.010	0.051	0.158
Hsp90ab1	0.049	0.312	0.989
Hnrnpab	0.008	0.096	0.312
Pgam1	0.039	0.509	1.586
Arhgdia	0.048	0.591	1.799
Akr1b1	0.038	0.310	0.890
ANX	0.043	0.452	1.311
Naga	0.039	0.139	0.350
Ast	0.023	0.317	0.930
Ezr	0.006	0.079	0.231
Mmp19	0.065	0.298	0.768
Ywhaz	0.053	0.412	1.122
Sptan1	0.001	0.030	0.087
Hprt1	0.020	0.247	0.678
Ganab	0.023	0.087	0.209
Rcn3	0.027	0.151	0.382
Psma	0.009	0.263	0.735
Pdi	0.041	0.264	0.667
FKBP9	0.011	0.086	0.214
Pitpna	0.009	0.098	0.250
Hspa8	0.019	0.140	0.335
Ctsd	0.061	0.433	1.032
Ywhaq	0.028	0.279	0.676
DMP4	0.000	0.002	0.003
Vim	0.003	0.071	0.175
Psap	0.228	1.666	3.833
Serpinh1	0.003	0.012	0.025
Mmp9	0.075	0.542	1.158
Psmb	0.010	0.188	0.421
Spata5	0.000	0.001	0.003
Acat2	0.020	0.211	0.460
Nucb2	0.160	0.626	1.228
Rps21	0.000	0.014	0.031
Ddt	0.047	0.487	1.005
Gsto1	0.023	0.130	0.256
PLTP	0.085	0.825	1.689
Dbi	0.046	0.313	0.625
Sep15	0.012	0.064	0.124
Hsp90aa1	0.020	0.123	0.241
Smpd	0.027	0.139	0.263
ANX	0.084	0.739	1.436

	Ppi	0.125	0.835	1.511
Cluster 3	Protein ID	day3	day8	day12
	Cln5	0.020	0.075	0.126
	Lamc1	0.105	0.616	1.080
	Atp6ap1	0.048	0.221	0.374
	Ctsz	0.138	0.523	0.857
	Plod1	0.045	0.321	0.544
	Glb1	0.004	0.044	0.073
	Ctsl	0.078	0.343	0.522
	Plbd2	0.025	0.195	0.308
	Rpl12	0.032	0.240	0.377
	Nid1	0.194	1.049	1.609
	Atp1a1	0.001	0.046	0.075
	Ahcy	0.000	0.003	0.004
	Twsg1	0.032	0.167	0.251
	C1qtnf5	0.112	1.288	2.010
	Lamb1	0.081	0.445	0.658
	Pebp1	0.046	0.625	0.962
	Tpm1	0.001	0.064	0.100
	Serpinb1a	0.058	0.435	0.634
	Thbs3	0.016	0.073	0.103
	Timp1	0.344	2.162	3.111
	Hexb	0.056	0.312	0.444
	LOXL1	0.045	0.361	0.501
	Nucb1	0.106	0.770	1.061
	Slc7a5	0.000	0.019	0.026
	Tinagl1	0.117	0.863	1.146
	DCN	0.173	1.457	1.945
	Calcrl	0.024	0.129	0.166
	Akr1b8	0.029	0.248	0.326
	Ehd3	0.000	0.022	0.029
	Icam1	0.050	0.367	0.467
	Slc3a2	0.011	0.203	0.261
	Gaa	0.065	0.505	0.638
	Emilin1	0.050	0.280	0.343
	Krt15	0.001	0.000	0.000
	Clu	0.381	2.565	3.132
	G6pd	0.002	0.007	0.009
	Incenp	0.001	0.000	0.000
	Eno	0.001	0.000	0.000
	Eef1a1	0.001	0.000	0.000
	Cbr1	0.001	0.000	0.000
	Casp3	0.001	0.000	0.000
	Aplp2	0.048	0.294	0.335
	Sbsn	0.113	0.682	0.772
	Gdi2	0.024	0.193	0.214
	Tnfsf9	0.029	0.180	0.198
	Rem1	0.001	0.000	0.000
	Crim1	0.001	0.000	0.000
	Lama5	0.102	0.623	0.666
	Ppia	0.002	0.000	0.000
	Hspg2	0.101	0.979	1.023
	Agrn	0.033	0.183	0.188
	Htra1	0.117	0.988	1.007
	Xirp2	0.000	0.001	0.001
	GLG1	0.026	0.227	0.229
	C3	0.078	0.388	0.389
Cluster 4	Protein ID	day3	day8	day12
	DKK3	0.228	1.517	1.511
	Sema4b	0.033	0.178	0.176
	Apc	0.000	0.001	0.001
	Dag1	0.044	0.329	0.320
	Lgmn	0.253	1.258	1.211
	MFGE8	0.083	0.432	0.398
	Fbxo43	0.000	0.002	0.002
	Lgals3bp	0.147	1.162	1.053
	Sepp1	0.025	0.170	0.153
	Nid1	0.121	0.837	0.753
	Gpc1	0.116	0.611	0.547
	Bzrap1	0.000	0.001	0.001
	Antxr2	0.000	0.007	0.006
	App	0.076	0.634	0.538
	Efemp1	0.106	0.672	0.551
	Ccl2	0.221	1.575	1.282
	ATP6AP2	0.067	0.623	0.504
	C1ra	0.154	0.642	0.512
	QSOX	0.118	0.739	0.588
	Pxdnl	0.106	0.630	0.496
	B3GNT	0.033	0.182	0.139
	BMP1	0.040	0.185	0.139
	Fstl1	0.075	0.427	0.321
	Sema3b	0.065	0.499	0.373
	IGFBP4	0.130	0.603	0.432
	PCOLCE	0.262	1.980	1.413
	Gm2a	0.039	0.198	0.138
	pam-b	0.045	0.324	0.224

Sdc	0.228	1.028	0.693
Cant1	0.071	0.375	0.250
Gpr56	0.073	0.400	0.258
Timp2	0.152	1.313	0.842
Cspg4	0.192	0.967	0.614
Srpx	0.049	0.318	0.194
Mamdc2	0.070	0.371	0.219
MFGE8	0.267	1.638	0.961
Ccdc80	0.026	0.242	0.139
Thbs1	0.104	0.581	0.332
Alcam	0.091	0.416	0.236
Sparc	0.316	2.061	1.157
Ptgfrn	0.018	0.192	0.105
Rnaset2	0.175	0.891	0.481
Itih5	0.091	0.646	0.341
Man2a1	0.020	0.192	0.101
ECM1	0.066	0.445	0.230
Man1a1	0.027	0.208	0.102
Sema3c	0.081	0.426	0.194
Ggh	0.025	0.172	0.078
Sema3e	0.044	0.242	0.101
Ccl7	0.124	0.335	0.135
Clstn1	0.054	0.374	0.143
Lpl	0.124	0.709	0.264
C7	0.000	0.005	0.002
Rpl6	0.000	0.001	0.000
Nrp1	0.012	0.096	0.031
Plxnb2	0.001	0.012	0.003
Birc6	0.000	0.001	0.000
Mamdc2	0.057	0.354	0.094
Pclo	0.000	0.001	0.000
Csmd2	0.000	0.001	0.000
Col5a3	0.001	0.001	0.000
St14	0.025	0.101	0.022
Kdm2a	0.000	0.001	0.000
Cdh23	0.000	0.002	0.000
ABCB1	0.000	0.002	0.000
Stip1	0.000	0.002	0.000
YES1	0.000	0.002	0.000
Mybpc1	0.000	0.002	0.000
Ncoa2	0.000	0.002	0.000
Pkm	0.000	0.003	0.000
Ssb	0.000	0.003	0.000
Cyp4f4	0.000	0.003	0.000
PRDM15	0.000	0.003	0.000
Inpp4a	0.000	0.003	0.000
Ppp4r3b	0.000	0.003	0.000
Asxl1	0.000	0.003	0.000
DUS3L	0.000	0.003	0.000
Eef1a	0.000	0.004	0.000
Dync2h1	0.000	0.004	0.000
Krt1b	0.001	0.004	0.000
Rps2	0.000	0.004	0.000
Lamp1	0.000	0.004	0.000
Rpl6	0.000	0.004	0.000
Cd74	0.000	0.004	0.000
SH3RF3	0.000	0.004	0.000
C6	0.000	0.005	0.000
Hist1h3a	0.000	0.005	0.000
LDH	0.000	0.005	0.000
Phf1	0.000	0.005	0.000
Tnxb	0.011	0.067	0.002
Hist1h4a	0.000	0.006	0.000
Krt2	0.001	0.006	0.000
MAGED2	0.000	0.007	0.000
Cd276	0.001	0.007	0.000
Rps2	0.000	0.008	0.000
Dcaf12	0.000	0.008	0.000
Rab35	0.000	0.009	0.000
His2A	0.000	0.009	0.000
Gp	0.004	0.010	0.000
Ldha	0.000	0.010	0.000
Taldo1	0.000	0.010	0.000
Ndrg1	0.000	0.010	0.000
Sema6c	0.000	0.011	0.000
Pdxk	0.003	0.011	0.000
Rps2	0.000	0.011	0.000
Rab8b	0.000	0.012	0.000
Atp2b1	0.000	0.013	0.000
Plxnb2	0.000	0.015	0.000
Enpp6	0.000	0.016	0.000
Arf5	0.000	0.017	0.000
Rras2	0.000	0.023	0.000
FAT1	0.007	0.034	0.000
B4galt1	0.008	0.052	0.000
Ccdc85a	0.000	0.054	0.000

Tgfbr3	0.017	0.079	0.000
ITM2C	0.003	0.095	0.000
Fn1	0.011	0.109	0.000
Lgals	0.012	0.168	0.000

Supplementary Table S8. Proteases and glycosidases affecting quality of therapeutic proteins from rCHO cells.

Protein	Product	Effect	References
β-galactosidase	mAb	Degalactosylation	35
Sialidase<br>β-galactosidase<br>β-hexosaminidase<br>fucosidase	Glycoproteins	Deglycoslylation	37
Metalloprotenases (MMP-3, 10, 12)	Recombinant factor VIII (r-FVIII)	Product degradation	8
Gelatinase-B (MMP-9)	Interferon-beta (IFN-b)	Degradation of IFN-b	38
Carboxypeptidase-B	IgG, hEPO, tPA	Removal of C-terminal Lys or Arg	39, 40
Serine and cysteine proteases	Recombinant Fc-fusion protein, VEGFR1	Partial cleavage of therapeutic proteins	42
Lysosomal phospholipase A2 isomer X1	mAb	Degradation of polysorbates	45
Cathepsin D	Fc-fusion protein<br>mAb	Fc fragmentation	12
